# Impact of scaling up prenatal nutrition interventions on human capital outcomes in low- and middle-income countries: a modeling analysis

**DOI:** 10.1093/ajcn/nqab234

**Published:** 2021-07-28

**Authors:** Nandita Perumal, Mia M Blakstad, Günther Fink, Mark Lambiris, Lilia Bliznashka, Goodarz Danaei, Christopher R Sudfeld

**Affiliations:** Department of Global Health and Population, Harvard T.H. Chan School of Public Health, Boston, MA, USA; Department of Global Health and Population, Harvard T.H. Chan School of Public Health, Boston, MA, USA; Department of Epidemiology and Public Health, Swiss Tropical and Public Health Institute, Basel, Switzerland; University of Basel, Basel, Switzerland; Department of Epidemiology and Public Health, Swiss Tropical and Public Health Institute, Basel, Switzerland; University of Basel, Basel, Switzerland; Department of Global Health and Population, Harvard T.H. Chan School of Public Health, Boston, MA, USA; Department of Global Health and Population, Harvard T.H. Chan School of Public Health, Boston, MA, USA; Department of Epidemiology, Harvard T.H. Chan School of Public Health, Boston, MA, USA; Department of Global Health and Population, Harvard T.H. Chan School of Public Health, Boston, MA, USA; Department of Nutrition, Harvard T.H. Chan School of Public Health, Boston, MA, USA

**Keywords:** prenatal nutrition interventions, iron-folic acid, calcium, multiple micronutrients, balanced energy protein, schooling, income, human capital

## Abstract

**Background:**

Prenatal nutrition interventions can lead to improved birth outcomes, which in turn are associated with better education and human capital outcomes later in life.

**Objective:**

We estimated the impact of scaling up iron–folic acid (IFA), calcium, multiple micronutrient (MMS), and balanced energy protein (BEP) supplementation for pregnant women, on human capital outcomes in low- and middle-income countries (LMIC).

**Methods:**

We used mathematical modeling with proportional reductions in adverse birth outcomes to estimate the potential gains in school years and lifetime income due to scaling up each prenatal nutrition intervention. Estimates of intervention effects on birth outcomes were derived from meta-analyses of randomized trials. Estimates of the associations between birth outcomes and schooling and lifetime income were derived from *de novo* meta-analyses of observational studies.

**Results:**

Across 132 LMIC, scaling up prenatal nutrition interventions to 90% coverage was estimated to increase school years and lifetime income per 5-yr birth cohort by: 2.28 million y (95% uncertainty intervals (UI): −0.44, 6.26) and $8.26 billion (95% UI: −1.60, 22.4) for IFA; 4.08 million y (95% UI: 0.12, 9.68) and $18.9 billion (95% UI: 0.59, 44.6) for calcium; 5.02 million y (95% UI: 1.07, 11.0) and $18.1 billion (95% UI: 3.88, 39.1) for MMS; and 0.53 million y (95% UI: −0.49, 1.70) and $1.34 billion (95% UI: −1.10, 3.10 billion) for BEP supplementation. South Asia and Sub-Saharan Africa tended to have the largest estimated regional gains in school years for scaling up each intervention due to the large population size and high burden of poor birth outcomes. Absolute income benefits for each intervention were estimated to be the largest in Latin America, where returns to education and incomes are higher relative to other regions.

**Conclusion:**

Increasing coverage of prenatal nutrition interventions in LMIC may lead to substantial gains in schooling and lifetime income. Decision makers should consider the potential long-term human capital returns of investments in maternal nutrition.

## Introduction

Child survival has dramatically improved over the past few decades with deaths among children <5 y of age declining from 12.6 million in 1990 to 5.6 million in 2016 (1). However, more than 250 million children in low- and middle- income countries (LMIC) are estimated to not reach their developmental potential, with over a quarter of children aged 3–5 y estimated to not meet minimum cognitive and socioemotional milestones (2, 3). Suboptimal development in early childhood is associated with lower educational and income attainment, which increases the risk of poorer health and socioeconomic position throughout the life course (4–6). Nutritional deficiencies during conception and pregnancy can lead to adverse birth outcomes, such as neural tube defects, low birthweight (LBW; <2500 g at birth), and preterm birth (birth before 37 wk gestational age), which in turn are associated with neurological disability, suboptimal early child development, and lower schooling achievement later in life (7–10). Interventions to improve nutrition in the first 1000 days, between conception and a child's second birthday, a period of rapid brain development, may therefore provide neurocognitive benefits throughout the life course (4, 5, 7).

The 2013 Lancet Maternal and Child Nutrition Series identified several nutrition-specific interventions to reduce the burden of maternal and child mortality and morbidity due to undernutrition in LMIC, and estimated the costs and cost-effectiveness of scaling these interventions to 90% coverage based on the number of lives saved (11, 12). The potential broader human capital benefits of scaling up maternal nutrition interventions during pregnancy due to reductions in adverse birth outcomes or improvements in child neurodevelopment, however, are unclear. In this study, we estimated the potential global, regional, and national gains in school years and lifetime income attributable to scaling up prenatal nutrition interventions in 132 LMIC.

## Methods

### Identifying maternal prenatal nutrition interventions and analytical approach

We conducted a landscape evidence review to identify prenatal nutrition interventions with a convincing level of evidence of a positive effect on birth outcomes, such as LBW and preterm birth, or on human capital outcomes, such as child neurodevelopment, disability, educational attainment, or lifetime income (**Supplemental Table 1**). A convincing level of evidence was defined as evidence of an intervention effect based on multiple randomized controlled trials or robust cohort studies (13). Of the 13 maternal prenatal nutrition interventions examined, 4 interventions had a convincing level of evidence of a beneficial effect on birth outcomes: *1*) iron–folic acid supplementation (IFA), *2*) calcium supplementation, *3*) multiple micronutrient supplementation (MMS), and *4*) balanced energy protein supplementation (BEP) among undernourished women. Although we initially considered quantifying the benefits of scaling up prenatal nutrition interventions using evidence of intervention effects directly on human capital outcomes, we did not find convincing level of evidence due to lack of data for direct intervention effects on human capital (Supplemental Table 1).

**FIGURE 1:**
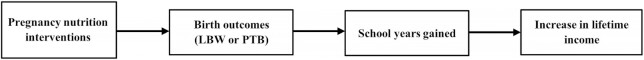
Conceptual modeling framework for assessing the impact of maternal micronutrient supplementation on human capital and income gains. LBW, low birthweight; PTB, preterm birth.

We therefore estimated the potential human capital benefits of scaling up prenatal nutrition interventions with convincing level of evidence of an effect on adverse birth outcomes as mediators on the pathway to schooling and lifetime income (**Figure 1**, **Table 1**). We did not restrict our analysis to only interventions with statistically significant effects because the 95% CIs of pooled estimates from randomized controlled trials reflect, in part, the heterogeneity due to study-specific characteristics, such as sample size, which can be refined as additional data become available. Furthermore, IFA and BEP supplementation are currently recommended by the WHO for pregnant women in all or in context-specific settings, respectively; therefore the potential human capital benefits of scaling up these interventions are important to estimate. For each intervention, we selected a single mediator; for IFA, BEP, and MMS, we used LBW as the mediator as there is a convincing level of evidence of an intervention effect on LBW or birthweight. We also considered small for gestational age (SGA; defined as birthweight for gestational age and sex <10th percentile of the standard reference population) as a potential mediator; however, meta-analyses of randomized controlled trials suggest a null effect of IFA, calcium, and BEP supplementation on SGA at birth (14–16). The most recent Cochrane review determined that MMS reduced the risk of SGA birth (17); however, the quality of evidence was rated as moderate compared with the high-quality of evidence on LBW. In addition, there is a convincing level of evidence that MMS reduces the risk of LBW for both anemic and non-anemic women. As such, we used LBW as the mediator for MMS as well. For calcium supplementation, we used preterm birth as the mediator as there is a convincing level of evidence of an effect on preterm birth, but not on LBW or SGA (15).

### Quantities estimated

We used population-based mathematical models to estimate 3 quantities to predict the potential human capital benefits of scaling up each prenatal nutrition intervention in a linear deterministic fashion. These were: *1*) the absolute reduction in LBW/preterm birth prevalence as a function of scaling up nutrition interventions, *2*) the increase in school years due to reductions in the LBW/preterm birth prevalence, and *3*) the increase in lifetime income due to gains in schooling. Linear deterministic models quantify the average behavior of a population given a set of parameter values assuming that the system does not dynamically change with downstream consequences (18). We estimated the potential gains of scaling up prenatal nutrition interventions from the current coverage level to 90% for IFA, and to 50% and 90% target coverage for calcium, BEP, and MMS supplementation in 132 LMIC. For IFA supplementation, we only estimated the impact of scaling up to 90% target coverage as several countries have reported current coverage >50%. We selected 50% target coverage levels to model intervention benefits if half of the population received the intervention, and 90% target coverage to model intervention benefits for an “ideal” scenario in which almost all pregnant women received the intervention. In the case of BEP supplementation, the intervention coverage scenarios were scaled by the proportion of pregnant women estimated to be underweight.

**TABLE 1:** Summary of effect sizes and sources of data for modeling the impact of prenatal nutrition interventions on relative reduction in adverse birth outcomes^1^

Maternal interventions	Mediator	Intervention effect size on birth outcomes	Source of prevalence data for birth outcomes	Sources of prevalence data for other parameters considered to estimate population-attributable fractions
Iron-folic acid supplementation (IFA)	LBW	Effect of IFA (vs. placebo) on LBW (*n* = 11): RR 0.84 (95% CI: 0.69, 1.03) (19)	National estimates of LBW in 2015, and 2010 for countries missing 2015 estimates (20, 21).	Current coverage of IFA supplementation abstracted from the most recent DHS. Indicator used: % women in the past 5 y who took iron tablets or syrup for >90 d.
Calcium supplementation	PTB	Effect of high-dose calcium (≥1g daily of calcium) vs. placebo: PTB (*n* = 11): RR 0.76 (95% CI: 0.60, 0.97) (15)	National estimates on PTB in 2015 (22).	
Balanced energy protein supplementation (BEP)	LBW	A meta-analysis of individual studies included in the most recent Cochrane review, which were conducted among undernourished women only (Supplemental Methods 2, page 4) (16). The pooled effect of BEP (vs. placebo) on LBW using a random effects meta-analysis was (*n* = 4): RR 0.83 (95% CI: 0.61, 1.12).	National estimates of LBW in 2015, and 2010 for countries missing 2015 estimates (20, 21).	Estimates of underweight (BMI <18.5) among women of reproductive age were derived from the 2016 Noncommunicable Disease risk factor collaboration (23)
Multiple micronutrient supplementation (MMS)	LBW	Effect size of MMS (vs. IFA) on LBW as modified by maternal anemia status (24): Among anemic women (Hb <110 g/L): RR 0.81 (95% CI: 0.74, 0.89)Among nonanemic women (Hb ≥110 g/L): RR 0.91 (95% CI: 0.85, 0.98)	National estimates of LBW in 2015 and 2010 for countries missing 2015 estimates (20, 21).	Country-specific national anemia prevalence (25).

^1^BEP, balanced protein energy supplementation; BMI, body mass index; DHS, Demographic and Health Surveys; Hb, hemoglobin status; IFA, iron–folic acid supplementation; LBW, low birthweight; MMS, multiple micronutrient supplementation; PTB, preterm birth.

#### Absolute reductions in adverse birth outcomes

We estimated the proportional reduction in LBW and preterm birth prevalence due to scaling up each prenatal nutrition intervention using the following formula:

(1) $$\begin{eqnarray*}
PA{F_{Birth\ outcome}} = \frac{{\left( {{P_{target\ coverage}} - {P_{current\ coverage}}} \right)\ x\ \left( {1 - RR} \right)}}{{1 + \ {P_{current\ coverage}}\ x\ \left( {RR - 1} \right)}}\nonumber\\
\end{eqnarray*}$$

where PAF is the population attributable fraction, RR is the relative risk of adverse birth outcome (LBW or preterm birth) among women who received the nutrition intervention compared with controls, and P_target coverage_ is the intervention coverage scenario (**Supplemental Methods 1**, page 3). We used pooled relative risks from recently published meta-analyses of randomized controlled trials of intervention effects on birth outcomes (Table 1). For BEP supplementation, a pooled relative risk was estimated based on random-effects meta-analysis of four BEP trials conducted among undernourished women only (**Supplemental Methods 2**, pages 4–5; **Supplemental Table 2**).

We selected 2015 as the base year for model parameterization given availability of data for LBW and preterm birth prevalence. We accounted for current coverage of interventions where possible. For IFA supplements, we estimated baseline coverage using data on self-reported intake of IFA supplements for >90 d from the most recent Demographic and Health Surveys (DHS) in each country. For calcium, BEP, and MMS supplementation, we assumed no baseline coverage in 2015. For BEP supplementation, we estimated the impact of scaling up the intervention on human capital outcomes among the proportion of women in the general population with a body mass index (BMI) of <18.5 kg/m^2^ (i.e., underweight women) (23). Notably, this approach differs from that presented in the current World Health Organization (WHO) guidelines, which recommend BEP supplementation for all individuals in populations where the prevalence of underweight women is >20% (26). We decided to model the impact for individuals rather than populations with low BMI because evidence from randomized controlled trials suggests individual-level benefits of BEP supplementation in reducing the risk of LBW among “undernourished” women; the definition of undernourished, however, varies between trials. In line with the WHO guidelines, we used low BMI as the criterion to define the target population for BEP supplementation (16). For MMS supplementation, we accounted for the differential effect of MMS among anemic (hemoglobin <110 g/dL) and non-anemic women using stratified effect sizes based on a recent individual patient data meta-analysis of randomized controlled trials (27) and the most recent country-specific national estimates of anemia prevalence (25). Given that most trials have assessed the efficacy of MMS relative to IFA, we modeled the effect of switching from IFA supplementation, based on the country-specific coverage of IFA, to MMS using the pooled relative risk from trials. For the remaining proportion of the population estimated to not receive IFA supplements, we assumed a multiplicative effect of receiving both IFA and MMS supplements, accounting for uncertainty in both effect sizes. We then used a weighted average of the stratified impact of MMS supplementation among anemic and non-anemic women to obtain the overall relative reduction in LBW attributable to scaling up MMS within each country.

Finally, to estimate the absolute reduction in adverse birth outcomes, we multiplied the estimated proportional reduction in LBW or preterm birth attributable to scaling up each intervention by the most recent 2015 country-specific estimates for LBW and preterm birth prevalence (22, 20). These national prevalence estimates for LBW and preterm birth were derived based on global modeling studies using national or nationally representative population-based datasets, accounting for variations in data quality. Countries with high coverage of civil registration and high-quality administrative data, primarily from upper-middle income or high-income countries, tended to have narrower uncertainty ranges around the prevalence point estimates. For countries for which LBW or preterm birth prevalence estimates were unavailable from 2015, we used the prevalence estimates from 2010 where available (21). For the few countries for which data for LBW or preterm birth prevalence were unavailable, we imputed the Global Burden of Disease (GBD) database regional average using a random-effects meta-analysis to pool national prevalence estimates and associated variance for countries within a given GBD subregion.

#### Quantifying the effect of reductions in adverse birth outcome on schooling

Given minimal data on the association between LBW or preterm birth with adult income in LMIC, we conducted a *de novo* systematic review and meta-analysis of the economics literature to examine the link between birthweight and human capital outcomes, including schooling and adult income (**Supplemental Methods 3**, page 6). The review identified evidence only from high-income country settings, given that studies from the economics literature rely primarily on sibling and twin designs, to identify the causal effect of being born LBW on adult schooling and income. As such, to account for all available evidence on the link between birth outcomes and schooling, we used random effects meta-analysis to pool estimates from the *de novo* systematic review with estimates of the associations between LBW and educational attainment based on longitudinal data from 5 major cohort studies [reanalysis of data based on the COHORTS collaboration (8, 9); Supplemental Methods 3, page 6]. The pooled estimate based on evidence from both high- and low-income settings suggests that LBW is associated with 0.29 fewer school years (95% CI: −0.48, −0.10). Estimates for the association between preterm birth and educational attainment, were only available from the COHORTS collaboration and suggest that preterm birth is associated with 0.32 fewer school years (95% CI: −0.57, −0.06).

To obtain average gains in schooling per child attributable to reductions in adverse birth outcomes, we multiplied the estimated absolute reduction in LBW or preterm birth prevalence by the effect sizes linking birth outcomes to years of schooling. We further estimated the population-level gains in schooling by multiplying the gains in schooling per child by the 2015-2020 birth cohort size of each country and the probability of survival up to age 25 to account for delayed completion of secondary school in many LMIC. Demographic data for each country were abstracted from the United Nations World Population Prospects 2019 Revision (28). Although we included all LMIC based on World Bank July 2019 income classifications in our initial analysis, 5 countries (Dominica, Republic of Kosovo, Marshall Islands, Nauru, and Tuvalu) were excluded from the final estimation as key demographic data on numbers of live births and survival probabilities were unavailable.

In addition, we estimated the potential gains in the number of youths (aged 20–24 y) who would complete secondary school due to the predicted population-level increases in school attendance. Assuming a 12-y education system, theoretically we would expect only grade 11 students to be eligible for crossing the threshold to secondary school completion through a marginal increase in school attendance. We derived the additional number of youths expected to complete secondary school by multiplying the proportion of the population with incomplete secondary school in the penultimate year of secondary school (i.e., grade 11) by the average gains in school years per child for each country. We used the country-specific prevalence of incomplete secondary school among youth (20–24 y) from the Barro-Lee education database and assumed an equal distribution of students among all secondary grades to estimate the proportion of the population in grade 11 (29).

#### Quantifying the net present value of future income

To estimate gains in adult income, we multiplied the number of school years gained at the population level by the previously published country-specific estimates for returns in adult income for each additional year of schooling (30, 31). Country-specific estimates of returns to annual income for each additional school year were based on a global review of the literature on returns to education, which used 197 estimates from 88 LMIC (31). The net present value of lifetime income was estimated by summing the country-specific discounted annual income over a 40-y working period (i.e., 20–59 y) (30). Given that average income data are not available for most countries, we followed previous work in assuming that average annual income corresponds to two-thirds of gross domestic product (GDP) in each country (32). The country-specific sum of discounted lifetime income was calculated in 2010 constant US dollars based on a 3% discounting rate, assuming an annual real wage growth of 2% and country-specific survival probabilities for each working year from 20–59 y based on Institute for Health Metrics gender-averaged lifetables. The World Development Indicators database was used to obtain information on per capita GDP for each country (32). To ensure comparability across countries and interventions, we used GDP in 2010 constant US dollars in primary analyses to generate unified benefit estimates which can be directly compared with costs of the interventions. However, the relative local value of income may vary due to local differences in purchasing power parity. Therefore, in sensitivity analyses, we estimated the gains in lifetime income, adjusted for purchasing power (reference year 2011). To understand what these total economic benefits would mean at the individual level, we further estimated the returns in lifetime income per child by dividing the total income gains per birth cohort by the number of children born to women targeted to receive the intervention.

#### Uncertainty intervals

We quantified parameter uncertainty by bootstrapping the SEs of each data input and effect size parameter used in each quantity, and propagated uncertainty using 1000 independently drawn simulations (**Supplemental Methods 4**, page 7). Uncertainty estimates were available from published estimates for prevalence of baseline population characteristics (i.e., low BMI, anemia, preterm birth, and LBW), the effect sizes for each prenatal nutrition intervention on LBW/preterm birth, the association between preterm birth/LBW to education gains, and the returns to income per additional year of education. The 95% uncertainty intervals (UI) around the final estimates were calculated by using the 2.5th and 97.5th percentiles of the 1000 simulations. All estimates were generated using the Stata 14 Statistical Software package (StataCorp LP).

#### Role of the funding source

The funders of the study had no role in the study design, data collection, analysis, and interpretation, writing of the report, or the decision to submit the manuscript for publication.

## Results

Across 132 LMIC, scaling up IFA supplementation coverage from current levels to 90% was estimated to result in an increase of 2.28 million (95% UI: −0.44, 6.26) school years and an additional 75.1 thousand youths (95% UI: −14.5, 203) completing secondary school per birth cohort (**Table 2**). Increasing coverage of calcium supplementation to 90% target coverage was estimated to result in 4.08 million (95% UI: 0.12, 9.68) additional school years and an increase of 154 thousand youths (95% UI: 4.58, 367) completing secondary school per birth cohort. Similarly, scaling up MMS to 90% coverage in LMIC was estimated to contribute to 5.02 million (95% UI: 1.07, 11.0) additional school years and an increase of 168 thousand youths (95% UI: 35.8, 369) completing secondary school per birth cohort. Finally, scaling up BEP supplementation among undernourished women to 90% coverage was estimated to result in an additional 0.53 million (95% UI: −0.49, 1.70) school years and 17.8 thousand youths (95% UI: −16.7, 57.6) completing secondary school per birth cohort in LMIC. Regionally, the estimated impact of scaling up each nutrition intervention on schooling outcomes was largest in South Asia and Sub-Saharan Africa for all interventions; however, estimated gains for IFA and BEP supplementation crossed the null.

**TABLE 2:** Total years of schooling gained and number of youths who would complete secondary school due to improvements in LBW or preterm birth attributable to scaling up maternal prenatal nutrition interventions at target coverage per 5-yr birth cohort (in thousands)^1^

Intervention	Target coverage (%)	Central Europe, Eastern Europe, Central Asia (*n* = 20)	Latin America and Caribbean (*n* = 24)	North Africa and Middle East (*n* = 14)	South Asia (*n* = 5)	Sub-Saharan Africa *(n* = 47)	Southeast Asia, East Asia, and Oceania (*n* = 22)	All LMIC (*n* = 132)
School years gained								
IFA	90%	45.5 (−8.66, 120)	104 (−18.4, 281)	175 (−28.8, 464)	1018 (−177, 2858)	691 (−130, 1834)	231 (−36.8, 635)	2277 (−441, 6261)
Calcium	50%	71.4 (2.81, 203)	165 (4.85, 392)	217 (8.64, 559)	713 (20.7, 1776)	665 (19.5, 1540)	420 (12.2, 1004)	2264 (68.3, 5378)
	90%	128 (5.06, 366)	297 (8.73, 705)	390 (15.6, 686)	1283 (37.2, 3196)	1197 (35.1, 2773)	756 (12.9, 1806)	4075 (123, 9681)
MMS	50%	41.5 (7.88, 89.4)	95.3 (21.4, 193)	162 (32.4, 354)	1068 (235, 2384)	679 (153, 1432)	210 (45.5, 438)	2248 (518, 4800)
	90%	80.4 (14.3, 174)	226 (45.1, 485)	330 (64.0, 738)	2399 (507, 5531)	1406 (285, 2986)	536 (111, 1160)	5021 (1065, 11,031)
BEP	50%	0.79 (−0.76, 2.58)	2.17 (−2.19, 7.21)	6.77 (−7.69, 20)	204 (−193, 685)	53.7 (−49.6, 162)	25.2 (−24.4, 77)	292 (−274, 942)
	90%	1.41 (−1.37, 4.64)	3.91 (−3.95, 13.0)	12.2 (−13.8, 36)	368 (−347, 1234)	94.8 (−89.4, 292)	45.4 (−43.9, 91)	525 (−494, 1695)
Secondary school completion among individuals aged 20–24 y								
IFA	90%	1.09 (−0.21, 2.87)	4.87 (−0.87, 13.2)	5.35 (−0.84, 14.5)	34.6 (−6.0, 100)	15.1 (−2.83, 39.5)	13.5 (−2.34, 37.0)	75.1 (−14.5, 203)
Calcium	50%	1.77 (0.06, 4.60)	7.95 (0.23, 19.1)	7.29 (0.29, 18.8)	24.9 (0.73, 62.3)	16.2 (0.47, 37.9)	27.2 (0.78, 64.7)	85.8 (2.54, 204)
	90%	3.19 (0.11, 8.28)	14.3 (0.42, 34.4)	13.1 (0.53, 33.9)	44.9 (1.31, 112)	29.2 (0.84, 68.2)	48.9 (1.40, 116)	154 (4.58, 367)
MMS	50%	1.01 (0.19, 2.21)	4.45 (1.08, 9.05)	4.90 (1.02, 10.8)	36.2 (7.91, 81.7)	15.0 (3.44, 31.4)	11.5 (2.44, 24.3)	73.0 (16.8, 156)
	90%	1.92 (0.34, 4.14)	10.4 (2.06, 22.2)	10.2 (1.93, 23.3)	81.5 (17.4, 190)	32.4 (6.58, 68.0)	30.0 (6.31, 66.2)	168 (35.8, 369)
BEP	50%	0.02 (−0.02, 0.07)	0.11 (−0.11, 0.35)	0.18 (−0.19, 0.53)	7.13 (−6.80, 23.9)	1.21 (−1.11, 3.63)	1.17 (−1.15, 3.86)	9.88 (−9.30, 32.0)
	90%	0.04 (−0.03, 0.12)	0.19 (−0.19, 0.64)	0.32 (−0.34, 0.96)	12.8 (−12.2, 43.1)	2.17 (−2.0, 6.53)	2.10 (−2.07, 6.96)	17.8 (−16.7, 57.6)

1 Total estimated educational benefits by birth cohort. Values in parentheses are 95% uncertainty intervals based on bootstrapped SEs. BEP, balanced energy protein supplementation; DHS, Demographic and Health Surveys; IFA, iron–folic acid supplementation; LBW, low birthweight; LMIC, low- and middle-income countries; MMS, multiple micronutrient supplementation.

Cumulative increase in lifetime income for scaling up each intervention to 90% coverage in LMIC was estimated to be $8.26 billion (95% UI: −1.60, 22.4) for IFA supplementation, $18.9 billion (95% UI: 0.59, 44.6) for calcium supplementation, $18.1 billion (95% UI: 3.88, 39.1) for MMS supplementation, and $1.34 billion (95% UI: −1.10, 3.10) for BEP supplementation among undernourished women (**Table 3**). At the regional level, absolute gains in lifetime income per birth cohort were larger for scaling up calcium supplementation to 90% coverage in Latin America ($5.73 billion; 95% UI: 0.19, 13.7) and scaling up MMS to 90% coverage in South Asia ($5.31 billion; 95% UI: 1.13, 12.7). Using purchasing power parity-adjusted income rates, compared with nominal rates, increased the estimated global economic benefits to $19.9 billion (95% UI: −3.88, 54.6) for IFA, $41.9 billion (95% UI: 1.31, 99.8) for calcium, $44.2 billion (95% UI: 9.67, 96.0) for MMS, and $3.96 billion (95% UI: −3.70, 12.7) for BEP supplementation when scaled to 90% target coverage (**Supplemental Table 3**).

**TABLE 3:** Net present value of lifetime income due to improvements in LBW or preterm birth attributable to scaling up maternal prenatal nutrition interventions at target coverage per 5-yr birth cohort (in US $ billions)^1^

Interventions	Target coverage (%)	Central Europe, Eastern Europe, Central Asia (*n* = 20)	Latin America and Caribbean (*n* = 24)	North Africa and Middle East (*n* = 14)	South Asia (*n* = 5)	Sub-Saharan Africa (*n* = 47)	Southeast Asia, East Asia, and Oceania (*n* = 22)	All LMIC (*n* = 132)
IFA	90%	0.38 (−0.07, 1.02)	1.94 (−0.35, 5.35)	0.95 (−0.16, 2.61)	2.26 (−0.41, 6.66)	1.56 (−0.29, 4.14)	1.09 (−0.17, 3.09)	8.26 (−1.60, 22.4)
Calcium	50%	0.53 (0.001, 1.94)	3.18 (0.10, 7.59)	1.26 (0.05, 3.23)	1.60 (0.05, 4.00)	1.79 (0.06, 4.29)	2.03 (0.06, 4.98)	10.5 (0.33, 24.8)
	90%	0.94 (0.002, 3.50)	5.73 (0.19, 13.7)	2.27 (0.08, 5.82)	2.87 (0.09, 7.20)	3.21 (0.10, 7.72)	3.65 (0.10, 8.97)	18.9 (0.59, 44.6)
MMS	50%	0.34 (0.07, 0.74)	1.73 (0.40, 3.71)	0.87 (0.18, 1.21)	2.33 (0.50, 5.42)	1.51 (0.33, 3.16)	0.98 (0.22, 2.15)	7.78 (1.80, 10.5)
	90%	0.68 (0.12, 0.12)	4.02 (0.85, 9.11)	1.83 (1.83, 4.13)	5.31 (1.13, 12.7)	3.37 (0.70, 7.36)	2.57 (0.50, 5.79)	18.1 (3.88, 39.1)
BEP	50%	0.006 (−0.006, 0.02)	0.04 (−0.04, 0.13)	0.02 (−0.02, 0.06)	0.46 (−0.44, 1.58)	0.10 (−0.09, 0.32)	0.11 (−0.10, 0.34)	0.75 (−0.70, 2.38)
	90%	0.011 (−0.011, 0.04)	0.07 (−0.07, 0.23)	0.03 (−0.03, 0.11)	0.84 (−0.79, 2.84)	0.18 (−0.17, 0.57)	0.20 (−0.19, 0.62)	1.34 (−1.10, 3.10)

1 Total estimated educational benefits by birth cohort. Values in parentheses are 95% uncertainty intervals based on bootstrapped standard errors. BEP, balanced energy protein supplementation; IFA, iron–folic acid supplementation; LBW, low birthweight; LMIC, low- and middle-income countries; MMS, multiple micronutrient supplementation.

**FIGURE 2:**
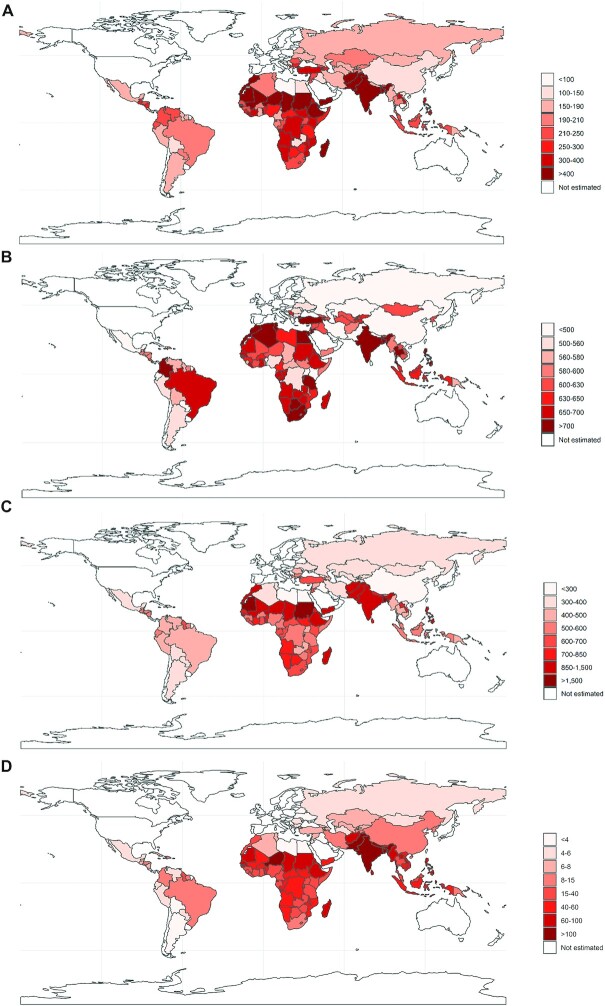
Population-averaged gains in school years per 100,000 children for scaling up (A) iron-folic acid supplementation, (B) calcium supplementation, (C) multiple micronutrient supplementation, and (D) balanced energy protein supplementation to 90% target coverage in 132 low- and middle-income countries.

**FIGURE 3:**
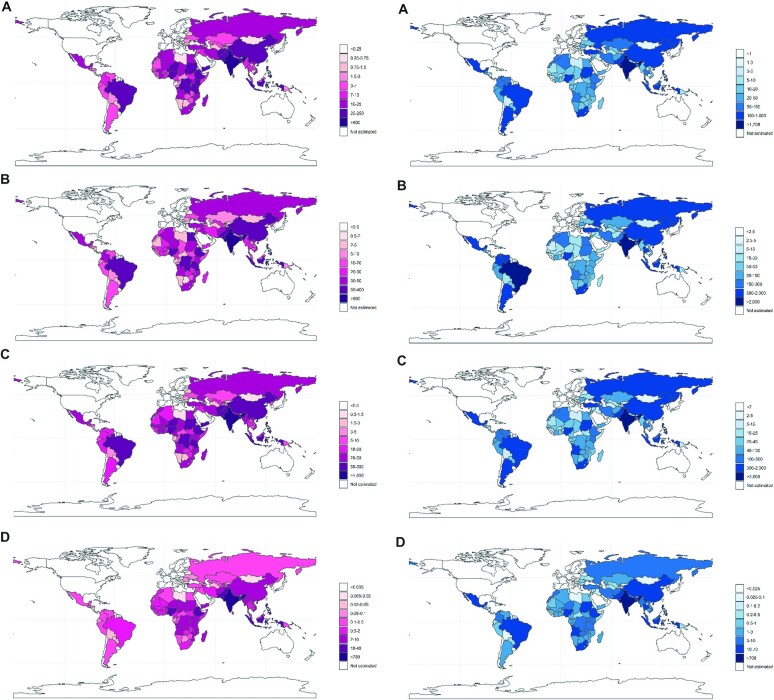
Gains in school years (in thousands; left column, pink) and lifetime income (in millions; right column, blue) per 5-yr birth cohort for scaling up (A) iron-folic acid supplementation, (B) calcium supplementation, (C) multiple micronutrient supplementation, and (D) balanced energy protein supplementation, to 90% target coverage in 132 low- and middle-income countries.

Countries with the largest population-averaged gains in school years per 100,000 children were in Sub-Saharan Africa and in South Asia (**Figure 2**), whereas countries with the largest absolute gains in school years and lifetime income per birth cohort were primarily in South Asia and Latin America (**Figure 3**). The 3 countries with the largest expected absolute gains in school years were India (0.70 million; 95% UI: −0.12, 2.10), Pakistan (0.20 million; 95% UI: −0.04, 0.60), and Ethiopia (0.12 million; 95% UI: −0.02, 0.31) for IFA supplementation; India (0.95 million; 95% UI: 0.03, 2.39), China (0.36 million; 95% UI: 0.01, 0.86) and Nigeria (0.20 million; 95% UI: 0.01, 0.53) for calcium supplementation; India (1.70 million; 95% UI: 0.36, 4.15), Pakistan (0.42 million; 95% UI: 0.08, 0.99) and Bangladesh (0.23 million; 95% UI: 0.04, 0.53) for MMS supplementation; and India (0.29 million; 95% UI: −0.28, 0.98), Pakistan (0.04 million; 95% UI: −0.04, 0.14), and Bangladesh (0.03 million; 95% UI: −0.03, 0.12) for BEP supplementation. Countries with the largest absolute gains in lifetime income for scaling up IFA supplementation to 90% coverage were India ($1.76 billion; 95% UI: −0.30, 5.37), Brazil ($0.81 billion; 95% UI: −0.15, 2.18), and Mexico ($0.54 billion; 95% UI: −0.08, 1.72); for calcium supplementation were Brazil ($2.73 billion; 95% UI: 0.08, 6.76), India ($2.40 billion; 95% UI: 0.08, 5.99), and China ($1.72 billion; 95% UI: 0.05, 4.23); for MMS supplementation were India ($4.33 billion; 95% UI: 0.91, 10.5), Brazil ($1.72 billion; 95% UI: 0.35, 3.76) and Mexico ($1.10 billion; 95% UI: 0.19, 2.92); and for BEP supplementation were India ($0.72 billion; 95% UI: −0.70, 2.48), Pakistan ($0.06 billion; 95% UI: −0.06, 0.24) and Indonesia ($0.06 billion; 95% UI: −0.06, 0.25).

**FIGURE 4:**
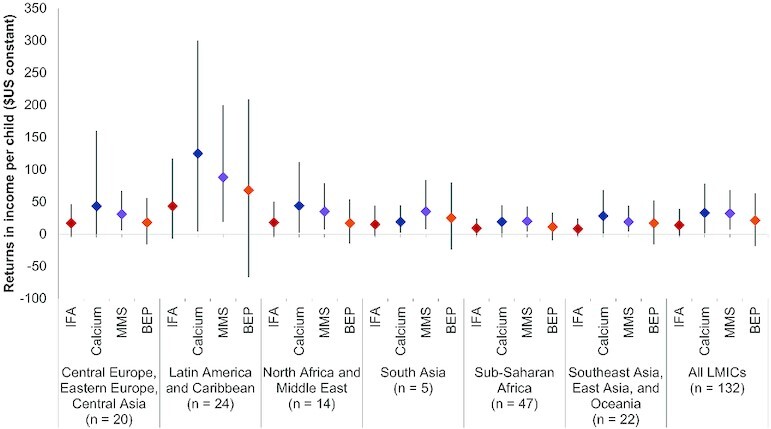
Global and regional estimated returns in the net present value of lifetime income per child born to women who received prenatal nutrition intervention at 90% target coverage in 2010 constant US dollars. BEP, balanced energy protein supplementation; Calcium, calcium supplementation; IFA, iron–folic acid supplementation; MMS, multiple micronutrient supplementation.

At the individual level, the absolute gain in future income per child born to pregnant women targeted for supplementation was estimated to be $14 (95% UI: −2.78, 39) for IFA, $33 (95% UI: 1.03, 78) for calcium, $32 (95% UI: 6.77, 68) for MMS, and $21 (95% UI: −19, 63) for BEP; with the largest regional gains in absolute income expected in Latin America and the Caribbean (**Figure 4**; **Supplemental Table 4**). Country-specific estimated gains in human capital and lifetime income for scaling up each intervention at 50% and 90% target coverage scenarios are summarized in the **Supplemental file online** (pages 11–142).

## Discussion

Our findings suggest that scaling up calcium and MMS supplementation to 90% coverage would yield large absolute gains in educational attainment and lifetime income. Gains in school years were estimated to be approximately 4 million school years for calcium supplementation and 5 million school years for MMS supplementation, leading to an estimated annual increase of approximately $18 billion in lifetime income for each intervention. Scaling up IFA supplementation from current levels to 90% coverage was estimated to lead to an increase of 2.28 million school years and $8.3 billion dollars in net present value of future earnings, and scaling up BEP supplementation among undernourished women was estimated to increase number of school years by 0.53 million years and $1.3 billion in lifetime earnings; the uncertainty intervals, however, included the null as the efficacy effect sizes of these interventions for reducing the risk of LBW crossed the null. Across all interventions, gains in educational attainment were largest for South Asia and Sub-Saharan Africa given the large population size and high prevalence of adverse birth outcomes. The estimated economic benefits of scaling up prenatal nutrition interventions in absolute terms in contrast were largest for Latin America, owing both to the larger returns for additional school years and the higher annual incomes relative to other regions.

This study, to our knowledge, provides the first global and national estimates of the potential human capital benefits of scaling up prenatal nutrition interventions across LMIC. Prior modeling studies have made an investment case for maternal nutrition interventions based on the number of lives saved and cost-effectiveness of implementing packages of nutrition-specific interventions in high-burden LMIC (11). However, only 2 studies have previously estimated the potential economic losses associated with poor child nutrition across several countries. Findings from these studies suggest substantial human capital losses associated with child linear growth faltering (∼69 million school years and US $177 billion in lifetime income lost per birth cohort across 137 LMIC) (30) and with inadequate breastfeeding (US $1.6 billion income losses and increase in healthcare expenditure costs of US $293 million across 7 Southeast Asian countries annually) (33). Whereas these estimates provide key data on the potential economic losses associated with broad indicators of poor health and suboptimal nutrition, estimating the potential economic benefits of specific interventions is a distinctly different approach (34). A case study in Egypt is among the only studies to previously estimate the potential cumulative economic gains of scaling up IFA flour fortification, which was estimated to be ∼US $886 million per year due to reductions in population prevalence of neural tube defects, perinatal mortality, child anemia, and adult productivity (35). Our study therefore provides a novel framework for modeling and quantifying the potential long-term human capital benefits of scaling up early life nutrition interventions globally.

The WHO guidelines for a positive pregnancy experience recommend IFA supplements for all pregnant women, and calcium supplementation in populations with low dietary intake of calcium (26). Our findings align with these recommendations as scaling up IFA and calcium supplementation may confer substantial estimated gains in educational attainment and lifetime earnings, even though estimates for human capital gains for IFA included the null. MMS supplementation during pregnancy, on the other hand, is currently a context-specific recommendation (36–38). Substantial evidence suggests, however, additional benefits of MMS compared with IFA supplements in reducing the risk of LBW, and therefore our models estimated large returns to scaling up MMS (17, 27). Decisionmakers should consider the potentially large returns of providing MMS during pregnancy in antenatal care programs. Similarly, although current guidelines recommend providing BEP supplements in undernourished populations, defined as those with a prevalence of low BMI >20% (26), the potential gains in schooling and lifetime income of providing BEP to individual women with low BMI in contexts where the prevalence of BMI is <20% should be taken into consideration. Even in the context of nutrition transition, which many LMIC are undergoing, the potential benefits to human capital outcomes of providing BEP supplements to underweight pregnant women are expected to be transferable. Future changes in population-level BMI distribution, however, will affect the number of women targeted for supplementation.

Furthermore, although our estimates suggest substantial human capital returns to scaling up each prenatal nutrition intervention, it is important to note that the estimated gains in school years and lifetime earnings attributable to each individual prenatal nutrition intervention cannot be summed to estimate the cumulative impact of scaling up a package of multiple prenatal nutrition interventions. Further work is needed to account for correlations in intervention coverage and the joint distribution of the magnitude of effect on birth outcomes when combining interventions to estimate the impact. Nevertheless, scaling up of a package of 4 prenatal nutrition interventions, including MMS, calcium, and BEP supplementation, to 90% coverage in 34 high-burden LMIC has previously been estimated to be highly cost-effective, with the cost per life saved estimated to be $571 (11). Accounting for the additional potential human capital benefits of scaling up these prenatal nutrition interventions is likely to increase the cost-effectiveness of individual interventions as well as intervention packages, particularly in contexts with a high burden of adverse birth outcomes. Future studies should aim to evaluate the cost-effectiveness of scaling up prenatal nutrition interventions individually and in a multicomponent package based on a wide range of health, nutrition, and human capital outcomes, and country-specific implementation costs for scaling up these interventions to target coverage.

Our study has several strengths and important limitations. We used a population-based linear deterministic model to estimate the impact of prenatal maternal nutrition interventions on long-term human capital outcomes using the most recent, robust evidence from randomized controlled trials or longitudinal cohort studies. However, evidence for the associations between birth outcomes and schooling and between schooling and lifetime income were based on observational evidence, which may be at risk of confounding and bias. Ideally, we would be able to parameterize the model solely based on causal evidence from long-term follow-up of randomized controlled trials. However, such evidence for schooling and lifetime income, for the prenatal interventions examined in this study, is not currently available largely due to the substantial time and resource investments required for extending trial follow-up into adulthood. New data from long-term follow-up studies of randomized controlled trials and high-quality observational cohorts in LMIC are likely to improve the validity and precision of parameter estimates used in this study. In addition, current evidence for BEP supplementation is complicated by the substantial heterogeneity in the nutritional composition of the supplements and the definition of “undernourished” participants used in previous trials. We used the most recent estimates for the effect of BEP supplementation on LBW among studies conducted in LMIC; however, additional data and synthesis of BEP trials, which more consistently define the nutritional composition of BEP supplements and the target populations that may benefit from this intervention, are needed to re-evaluate the intervention effect on LBW. The large uncertainties in the estimated years of schooling and lifetime income gains therefore reflect the uncertainties in the model parameters that may be refined with new evidence on intervention effect sizes and model parameters. Of note, although we used the most recent 2015 LBW and preterm birth estimates available, updated SGA estimates for the same year would allow for greater potential to generate estimates through specific pathways of earlier timing and intrauterine growth restriction. We used uncertainty propagation methods to account for uncertainties in all model parameters, including: *1*) intervention effect sizes on birth outcomes; *2*) prevalence estimates of baseline population characteristics, including LBW and preterm birth; and *3*) the effect sizes for associations between birth outcomes and schooling, and between schooling and lifetime income. However, we did not incorporate modeling uncertainty, which is not easily quantifiable; therefore, our uncertainty intervals may be considered as a lower boundary. It is important to note that we used a population-level modeling approach and therefore our population-level estimates of schooling and human capital benefits should not be interpreted to affect all births equally in a country. The absolute gains in future income per child born to pregnant women targeted by an intervention reflects the average gain for a child given country-level population characteristics. In addition, we estimated the human capital impacts of prenatal nutrition interventions through one specific pathway—reductions in adverse birth outcome and gains in educational attainment—and therefore may have underestimated the overall benefits that may accrue through other pathways. For example, since the uncertainty intervals of the estimated human capital benefits of scaling up IFA and BEP crossed the null, it is unclear if scaling up these interventions will improve schooling and lifetime incomes through their effect on LBW; nevertheless, it is important to note that there may be other potential pathways through which human capital benefits may accrue in addition to benefits of these interventions for other maternal and child health outcomes. As such, future studies should consider estimating the economic benefits of prenatal nutrition interventions through additional pathways, such as improved socioemotional and cognitive development, increased adult productivity, or lower healthcare costs associated with lower risk of adverse birth outcomes. We were also unable to account for education quality as we did not have data on learning-adjusted school years. Finally, our analysis focused on maternal nutritional supplements; therefore, research is needed to examine the potential role of other population-based nutrition-sensitive and nutrition-specific interventions, such as large-scale food fortification, on human capital gains throughout the life course.

Overall, our findings suggest that scaling up prenatal nutrition interventions may lead to substantial gains in schooling and lifetime incomes in LMIC, with large benefits expected in countries with a high burden of adverse birth outcomes, greater estimated returns to education, and higher annual wages. Researchers, funders, and policy makers should consider the broader human capital returns that may be attained through greater investments in scaling up prenatal nutrition interventions.

## Supplementary Material

nqab234_Supplemental_File

## Data Availability

Data described in the manuscript is publicly available. The codebook and analytic code will be made available upon request.

## Notes

Supported by funding from the Bill and Melinda Gates Foundation (grant mumber OPP1198520). The funders had no role in the design, implementation, analysis or interpretation of the data. NP was partially supported by the Canadian Institutes of Health Research Fellowship.

Supplemental Tables 1–4 and Supplemental Methods are available from the “Supplementary data” link in the online posting of the article and from the same link in the online table of contents at https://academic.oup.com/ajcn/.

Abbreviations used: BEP, balanced energy protein supplementation; DHS, Demographic and Health Surveys; IFA, iron–folic acid supplementation; LBW, low birthweight; LMIC, low- and middle-income countries; MMS, multiple micronutrient supplementation; SGA, small-for-gestational age.
