# Correction to: Perumal et al. Impact of scaling up prenatal nutrition interventions on human capital outcomes in low- and middle-income countries: a modeling analysis

**DOI:** 10.1093/ajcn/nqac282

**Published:** 2022-11-15

This is a correction to: Perumal et al. Impact of scaling up prenatal nutrition interventions on human capital outcomes in low- and middle-income countries: a modeling analysis. Am J Clin Nutr 2021;114:1708–18.

In the originally published version of this manuscript, the 2015 fertility data from the World Population Prospectus 2019 were used for the number of birth, which presents birth cohort data in five-year intervals. This may not have been clear to some readers. Therefore, “5-year” has been added to the text to clarify the birth cohort size in the abstract, table/figure titles, and methods. These changes have been made online. The authors apologize for any confusion.

