# Supplementary material for: Impact of scaling up prenatal nutrition interventions on human capital outcomes in low- and middle-income countries: a modeling analysis
Source: Am J Clin Nutr. 2021 Jul 28;114(5):1708–18. doi: 10.1093/ajcn/nqab234 (PMC8574629; doi:10.1093/ajcn/nqab234)
Supplement: nqab234_Supplemental_File [file nqab234_supplemental_file.pdf]

Online Supplementary Material

Nandita Perumal, Mia Blakstad, Günther Fink, Mark Lambiris, Lilia Bliznashka, Goodarz Danaei, Christopher R. Sudfeld

Supplementary Table 1: Maternal prenatal nutrition interventions examined and the level of evidence on the direct and indirect[1].

Pregnancy Nutrition Interventions	Level of evidence on child development, disability, schooling or lifetime income	Level of evidence for the effect on mediators of human capital outcomes (e.g. birth outcomes, child anemia)
Iron-Folic acid supplementation	Limited-suggestive	Convincing
Calcium supplementation	No evidence	Convincing
Multiple micronutrient supplements	Limited-suggestive	Convincing
Maternal folic acid supplementation	Convincing	Limited-suggestive
Balanced energy-protein supplements	Limited-suggestive	Convincing
Iodine supplementation	Limited-suggestive	Limited-suggestive
Maternal zinc supplementation	No evidence	Probable
Choline supplementation	No evidence	No evidence
Vitamin A supplementation	No evidence	Probable
Deworming intervention	No evidence	Limited-suggestive
Lipid-based nutrient supplements	No evidence	Probable
Maternal dietary interventions	No evidence	Limited-no conclusion
Fatty acids supplementation	No evidence	Limited-suggestive

[1]Criteria for level of evidence adapted from: Danaei G, Andrews KG, Sudfeld CR, Fink G, McCoy DC, Peet E, et al. (2016) Risk Factors for Childhood Stunting in 137 Developing Countries: A Comparative Risk Assessment Analysis at Global, Regional, and Country Levels. PLoS Med 13(11):e1002164.
Level of evidence criteria: 'Convincing' - evidence from more than one study type (e.g. randomized trials and cohort studies); 'Probable' - evidence from at least two independent randomized trials, cohort studies, or at least five case-control or cross sectional studies; 'Limited-suggestive' - evidence from at least two independent randomized trials or cohort studies or at least five case-control or cross-sectional studies; 'Limited-no conclusion' - evidence is so limited that no firm conclusion can be made.

Supplementary Methods 1: Considerations for estimating intervention-specific relative reduction in birth outcomes due to scaling up prenatal nutrition interventions to target coverage.

We used the following general formula to estimate the population attributable fraction (PAF) of each outcome.

$$PAF_{Birth\ outcome} = \frac{(P_{target\ coverage} - P_{current\ coverage})\ x\ (1 - RR)}{1 +\ P_{current\ coverage}\ x\ (RR - 1)}$$

All four selected interventions had a protective effect on birth outcomes; as such all relative risks were <1.0. For iron-folic acid (IFA) supplementation, we accounted for baseline coverage of IFA supplements in each country. For calcium, balanced energy protein (BEP) supplementation, and multiple micronutrient supplementation (MMS), the baseline coverage of the intervention was assumed to be 0. For BEP, we further scaled the PAF by the proportion of the female population of reproductive age estimated to have low body-mass-index (BMI).

For MMS, we first accounted for baseline IFA supplement coverage and then used stratified risk estimates for the effect of MMS on low birthweight by maternal anemia separately. When the baseline IFA coverage was above target coverage of MMS (e.g. baseline coverage of IFA was greater than 50% target coverage scenario), we estimated the PAF for shifting the population from IFA to MMS using stratum-specific relative risk of low birthweight among anemic vs. non-anemic women:

$$PAF_{Low\ birthweight} = [Prevalence_{Anemia} * \left[\left(P_{target\ coverageMMS}\right) x\ (1 - RR_{MMSanemic})\right] +$$
$$[(1 - Prevalence_{Anemia}) * \left[\left(P_{target\ coverageMMS}\right) x\ (1 -\ RR_{MMSnot\ anemic})\right]$$

When baseline coverage of IFA supplements was below target coverage of MMS, we estimated the relative reduction in low birthweight assuming a multiplicative impact of receiving MMS and IFA based on stratum-specific RRs for MMS intervention effect among anemic and non-anemic women. We assumed that the proportion of women who are anemic is the same among women currently receiving IFA supplements and not receiving IFA supplements.

Supplementary Methods 2: Effect of balanced protein energy (BEP) intake on low birthweight among undernourished women

We conducted a *de novo* random-effects meta-analysis of a subset of studies, included in the most recent meta-analysis (Ota et al., 2015), which were conducted among 'undernourished' women only. This is because the most recent guidelines from the World Health Organization recommend balanced protein energy supplementation in populations where the population of undernourished women, as defined by body-mass-index <18.5 kg/m^2, is above 20%[1]. The characteristics of the included studies are briefly summarized in the table below. Random-effects meta-analysis was used to derive a pooled effect size for the association between BEP supplementation and low birthweight (<2500 grams).

Supplementary Table 2: Characteristics of studies on the effect of balanced protein energy supplementation during pregnancy included in the meta-analysis.

Study, year	Settings	Study design	Participant description	Description of 'undernourished'	Intervention	Comparison
Blackwell et al., 1973[2]	Taiwan	RCT	Well-nourished rural Taiwanese women with 'marginal' diets and who were married and planned to have at least one more child were recruited in the last trimester of pregnancy.	By low socioeconomic status (defined as a lack of electric appliances in the home) and consuming baseline protein <40g/day (had a 'marginal diet') based on a preliminary food survey in 1965 in this area (estimated a daily energy intake of approximately 2000 kcal and protein intake <= 40 g for adult women)	Chocolate-flavoured liquid supplement given twice daily beginning after prior birth and continuing during index pregnancy until 15 months postpartum; supplement contained 40 g protein and 800 kcal energy plus vitamins/minerals. (114 in intervention group)	Control: supplement containing vitamins and minerals only, but given at the same time and for the same duration. (111 in control group).
Kardjati et al., 1988[3]	East Java	RCT	747 women in 3 rural villages at 26-28 weeks' gestation.	Study was conducted in an area known to be 'nutritionally vulnerable' based on a previous nutrition survey conducted. Total mean ± SD pre-pregnant BMI was 18.7 ± 2.0 kg/m2.	Supplement containing a dry powder (50% fat, 10% casein, and 40% glucose) providing 465 kcal energy and 7.1 g protein ('high energy').	Control: Supplement containing 52 kcal energy and 6.2 g protein ('low energy')
Ceesay et al., 1997[4]	Gambia	Cluster-RCT	Women (15-45 yrs) from 28 villages with "chronically" marginal nutrition. The mean maternal BMI measured after delivery was 20.7 ± 2.3 kg/m2 in the control group and 21.3 ± 2.8 kg/m 2 in the intervention group.	Undernutrition more pronounced from June to October (the 'hungry' season involving low food supply and heavy agricultural work) than from November to May (the dry harvest season with adequate food supply and less strenuous work).	Experimental villages: 2 supplement biscuits containing roasted groundnuts, rice flour, sugar, and groundnut oil (4250 kJ (1017 kcal) energy, 22 g protein, 56 g fat, 47 mg calcium, and 1.8 mg iron) consumed daily in presence of birth attendants.	Control villages: no supplement.
Huybregts et al., 2009[5]	Burkina Faso	RCT	1296 pregnant women in 2 villages.	None. However, approximately 13% of participants overall had BMI <18.5 kg/m2 and ~40% had hemoglobin <11.5g/dL. BMI at entry for intervention group was 20.8 ± 2.2 kg/m2, and control group was 21.0 ± 2.2 kg/m2.	Prenatal multiple micronutrient + fortified food supplement.	Control: multiple micronutrient supplement.

Effect of balanced protein energy supplementation on low birthweight among undernourished women

	BEP		Placebo/Standard diet			Risk Ratio	
**Study or Subgroup**	**Events**	**Total**	**Events**	**Total**	**Weight**	**M-H, Random, 95% CI**	**Year**
Blackwell 1973	7	143	11	138	8.8%	0.61 [0.25, 1.54]	1973
Kardjati 1988	29	258	24	252	20.3%	1.18 [0.71, 1.97]	1988
Ceesay 1997	112	1010	176	1037	39.0%	0.65 [0.52, 0.81]	1997
Huybregts 2009	67	523	67	497	31.9%	0.95 [0.69, 1.30]	2009
**Total (95% CI)**		**1934**		**1924**	**100.0%**	**0.83 [0.61, 1.12]**	
Total events	215		278				

Heterogeneity: $Tau^2 = 0.05$; $Chi^2 = 6.74$, df = 3 (P = 0.08); $I^2 = 55\%$
Test for overall effect: Z = 1.25 (P = 0.21)

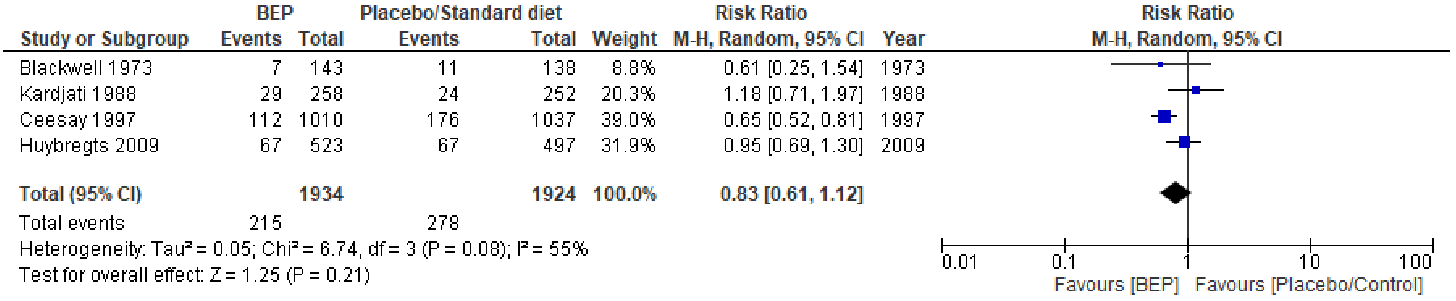

Supplementary Methods 3: *De novo* systematic review and meta-analysis combining evidence from the economics literature and birth cohort studies in the low- and middle-income countries.

To quantify the relationship between birthweight and human capital and labour market outcomes, we conducted a systematic review of the economics literature up to February 2019 using EconLit. Studies were included in the review if they: (i) used individual-level data in the analysis; (ii) the exposure was birth weight either defined as a continuous variable or categorically (e.g. LBW or extremely low birth weight); (iii) the outcome was measured at age ten or above; (iv) outcomes were related to education, schooling, “human capital”, “labor force” or unemployment, and (v) were published in English language. Studies were excluded if only outcomes related to parental investment in their children, adult health (but not economic) outcomes, or behavioural measures or measures of mental health, were examined.

From the 145 abstracts screened, 29 studies that used individual-level data to estimate the effect of birthweight on cognitive ability, educational attainment, labour force participation, and wages were included. Random-effects meta-analyses were used to estimate the average relationship between low birthweight and human capital outcomes. Studies from the economics literature rely primarily on sibling and twin designs to identify the causal effect of being born low birthweight (<2500 grams) on schooling and income. However, of the 29 studies eligible for inclusion in the review, only one study was from a lower-middle income country; the remaining studies were from high-income countries. Estimates for the relationship between low birthweight and schooling based on studies conducted in high-income countries are not directly applicable to low and middle income countries. Therefore, we generated a pooled estimate combining evidence from the economics literature[6–10] and data from the five birth cohorts in low- and middle-income countries[11]. Summarized in the figure below, the combined evidence suggests that being born low birthweight (<2500g) compared to normal birthweight reduces educational attainment by 0.29 years (95%CI -0.49, -0.096).

Estimated Association of Low birthweight (<2500g) with Educational Attainment

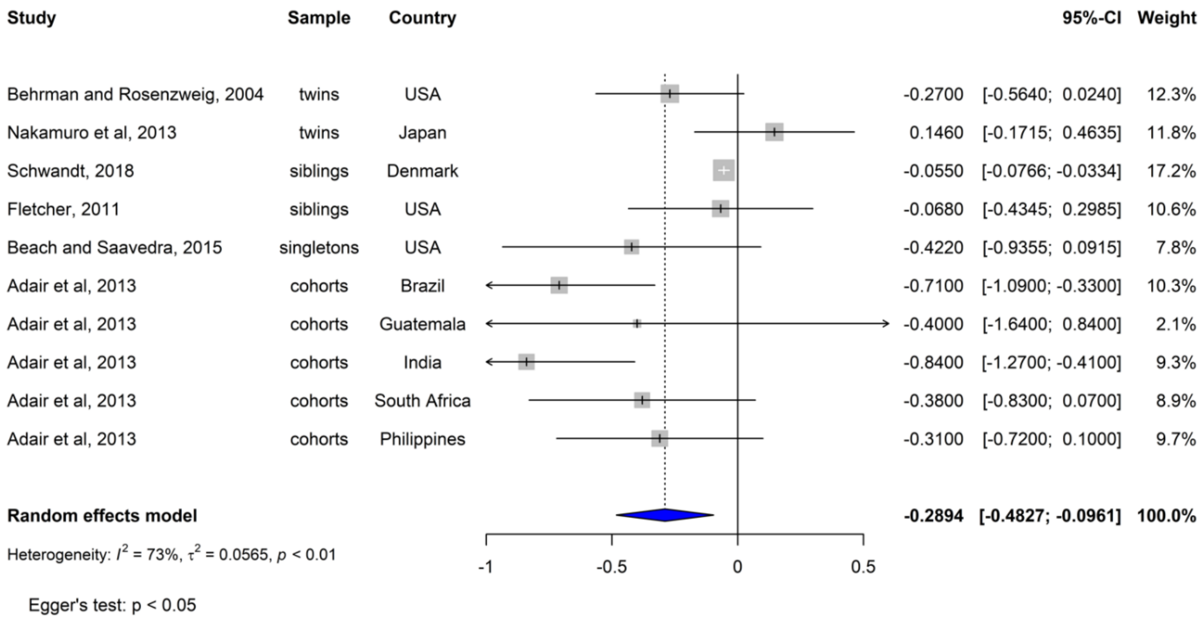

Supplementary Methods 4: Methods for uncertainty interval estimation

We used bootstrapping to account for parameter uncertainty in each step of the quantification and propagated uncertainty using 1,000 independently drawn simulations. Uncertainty estimates were available from published estimates for prevalence of baseline population characteristics (i.e. low BMI, anemia, preterm birth, and LBW), the effect sizes for each prenatal nutrition intervention on LBW/preterm birth, and returns to income per additional year of education. For any country for which baseline prevalence of population characteristics was unavailable, we used random-effects meta-analysis to impute the GBD sub-regional average with SE estimates. For countries for which the 2010 low birthweight prevalence estimates were used, which were published without variance estimation, we used a conservative approach and borrowed the largest variance (i.e. standard error) from a country from the same GBD subregion to estimate uncertainty around the 2010 low birthweight prevalence estimates. Standardized mean differences were assumed to be normally distributed and relative risks were assumed to be log-normal. All parameters were assumed to be independent as they are derived from different studies (e.g. global modeling of LBW versus meta-analysis of LBW and schooling).

Supplementary Table 3: Net present value of lifetime income gains due to improvements in low birthweight or preterm birth attributable to scaling up maternal prenatal nutrition interventions at target coverage per birth cohort (in International $, billions).[1]

Intervention	Target Coverage (%)	Central Europe, Eastern Europe, Central Asia (n = 20)	Latin America and Caribbean (n = 24)	North Africa and Middle East (n = 14)	South Asia (n = 5)	Sub-Saharan Africa (n = 47)	Southeast Asia, East Asia, and Oceania (n = 22)	All LMICs (n = 132)
Iron/Folic Acid	90%	0.86 (-0.16, 2.29)	3.03 (-0.55, 8.45)	2.15 (-0.37, 5.90)	7.69 (-1.39, 22.7)	3.39 (-0.64, 9.03)	2·68 (-0.41, 7.55)	19.9 (-3.88, 54.6)
Calcium	50%	1.19 (0.01, 4.37)	4.88 (0.16, 11.6)	2.96 (0.12, 7.75)	5.34 (0.16, 13.4)	3.68 (0.11, 8.83)	4·86 (0.13, 12.0)	23.3 (0.73, 55.5)
	90%	2.14 (0.02, 7.86)	8.78 (0.29, 20.9)	5.33 (0.21, 13.9)	9.61 (0.30, 24.1)	6.62 (0.20, 15.9)	8·76 (0.24, 21.5)	41.9 (1.31, 99.8)
Multiple Micronutrient Supplements	50%	0.78 (0.16, 1.68)	2.71 (0.62, 5.84)	1.95 (0.41, 4.21)	7.94 (1.72, 18.2)	3.28 (0.72, 6.85)	2·43 (0.50, 5.30)	19.2 (4.40, 41.1)
	90%	1.54 (0.28, 3.35)	6.31 (1.32, 14.2)	4.16 (0.78, 9.19)	18.0 (3.84, 42.8)	7.19 (1.50, 15.5)	6·31 (1.24, 14.1)	44.2 (9.67, 96.0)
Balanced Energy Protein	50%	0.014 (-0.013, 0.045)	0.06 (-0.006, 0.20)	0.05 (-0.05, 0.15)	1.55 (-1.47, 5.26)	0.23 (-0.21, 0.71)	0·28 (-0.27, 0.89)	2.20 (-2.05, 7.07)
	90%	0.024 (-0.024, 0.081)	0.10 (-0.11, 0.35)	0.86 (-0.88, 0.28)	2.79 (-2.64, 9.46)	0.41 (-0.38, 1.28)	0·51 (-0.48, 1.60)	3.96 (-3.70, 12.7)

[1]Total estimated educational benefits by birth cohort. Values in parentheses are 95% uncertainty intervals based on bootstrapped SEs.

Supplementary Table 4: Returns in the net present value of lifetime income per child born to women who received the intervention at target coverage per birth cohort.[1]

Intervention	Target Coverage (%)	Central Europe, Eastern Europe, Central Asia (n = 20)	Latin America and Caribbean (n = 24)	North Africa and Middle East (n = 14)	South Asia (n = 5)	Sub-Saharan Africa (n = 47)	Southeast Asia, East Asia, and Oceania (n = 22)	All LMICs (n = 132)
**2010 US dollars**								
Iron-folic Acid	90%	17 (-3.21, 46)	43 (-7.67, 117)	18 (-3.16, 50)	15 (-2.67, 44)	9·2 (-1.73, 24)	8·2 (-1.27, 24)	14 (-2.78, 39)
Calcium	50%	43 (0.11, 160)	126 (4.12, 300)	44 (1.63, 112)	19 (0.58, 47)	19 (0.59, 45)	28 (0.76, 68)	33 (1.03, 78)
	90%	43 (0.11, 160)	125 (4.12, 300)	44 (1.63, 112)	19 (1.63, 45)	19 (0.59, 45)	28 (0.76, 68)	33 (1.03, 78)
Multiple Micronutrient Supplements	50%	28 (5.66, 61)	69 (16, 147)	30 (6.21, 65)	28 (5.94, 64)	16 (3.52, 33)	13 (2.82, 29)	24 (5.66, 53)
	90%	31 (5.61, 67)	88 (19, 200)	35 (6.87, 79)	35 (7.47, 84)	20 (4.12, 43)	19 (3.82, 44)	32 (6.77, 68)
Balanced Energy Protein	50%	18 (-16, 56)	68 (-67, 209)	17 (-15, 54)	25 (-24, 80)	11 (-9·83, 33)	17 (-16, 52)	20 (-19, 63)
	90%	18 (-16, 56)	68 (-67, 209)	17 (-15, 54)	25 (-24, 80)	11 (-9·83, 33)	17 (-16, 52)	21 (-19, 63)
**2011 International dollars**								
Iron-folic Acid	90%	39 (-7.28, 104)	67 (-12, 186)	41 (-7.03, 113)	51 (-9.16, 149)	20 (-3.79, 53)	20 (-3.08, 57)	35 (-6.76, 95)
Calcium	50%	97 (1.06, 358)	193 (6.44, 460)	102 (4.10, 268)	63 (1.94, 159)	40 (1.18, 94)	66 (1.82, 163)	73 (2.28, 174)
	90%	97 (1.06, 358)	193 (6.44, 460)	102 (4.10, 268)	63 (1.94, 159)	40 (1.82, 94)	66 (1.82, 163)	73 (2.28, 174)
Multiple Micronutrient Supplements	50%	64 (13, 138)	107 (24, 231)	67 (14, 146)	94 (20, 216)	35 (7.66, 73)	33 (6·80, 72)	60 (14, 129)
	90%	70 (13, 153)	139 (29, 313)	80 (15, 177)	119 (25, 282)	42 (8.85, 91)	48 (9.38, 107)	77 (17, 167)
Balanced Energy Protein	50%	41 (-37, 127)	104 (-99, 319)	41 (-39, 131)	83 (-81, 267)	24 (-22, 74)	43 (-42, 134)	59 (-55, 189)
	90%	41 (-37, 127)	104 (-99, 319)	41 (-39, 131)	83 (-81, 267)	24 (-22, 74)	43 (-42, 134)	59 (-55, 189)

[1]Values in parentheses are 95% uncertainty intervals based on bootstrapped SEs.

References

1 World Health Organization. WHO recommendations on antenatal care for a positive pregnancy experience. Geneva, 2016 DOI:10.1017/CBO9781107415324.004.

2 Blackwell R, Chow B, Chinn K, Blackwell B, Hsu S. Prospective maternal nutrition study in Taiwan: rationale, study design, feasibility, and preliminary findings. *Nutr Rep Int* 1973; **7**: 517–32.

3 Kardjati S, Kusin JA, Schofield WM, De With C. Energy supplementation in the last trimester of pregnancy in East Java, Indonesia: Effect on maternal anthropometry. *Am J Clin Nutr* 1990; **52**: 987–94.

4 Ceesay SM, Prentice AM, Cole TJ, *et al.* Effects on Birth Weight and Perinatal Mortality of Maternal Dietary Supplements in Rural Gambia: 5 Year Randomised Controlled Trial. *Br Med J* 1997; **315**: 786–90.

5 Huybregts L, Roberfroid D, Lanou H, *et al.* Prenatal food supplementation fortified with multiple micronutrients increases birth length: A randomized controlled trial in rural Burkina Faso. *Am J Clin Nutr* 2009; **90**: 1593–600.

6 Behrman J, Rosenzweig M. Returns to Birthweight. *Rev Econ Stat* 2004; **86**: 586–601.

7 Nakamuro M, Uzuki Y, Inui T. The effects of birth weight: Does fetal origin really matter for long-run outcomes? *Econ Lett* 2013; **121**: 53–8.

8 Schwandt H. The Lasting Legacy of Seasonal Influenza: In-Utero Exposure and Labor Market Outcomes. 2018.

9 Fletcher M. The Medium Term Schooling and Health Effects of Low Birth Weight: Evidence from Siblings. *Econ Educ Rev* 2011; **30**: 517–27.

10 Beach B, Saavedra M. Mitigating the effects of low birth weight: Evidence from randomly assigned adoptees. *Am J Heal Econ* 2015; **1**: 275–96.

11 Adair LS, Fall CHD, Osmond C, *et al.* Associations of linear growth and relative weight gain during early life with adult health and human capital in countries of low and middle income: Findings from five birth cohort studies. *Lancet* 2013; **382**: 525–34.

Afghanistan

Region: North Africa and Middle East**; Sub-region:** North Africa and Middle East

Low birthweight prevalence[1]: 20.0% (95% CI: 16.0, 24.0)

Preterm birth prevalence[2]: 10.4% (95% CI: 8.7, 11.9)

Number of births[3]: 6,024,000

Returns to education[4]: 7.8% (95% CI: 7.0, 8.6)

GDP per capita 2010 US$ (estimated annual wage)[5]: $574 ($383/year)

GDP per capita 2011 International $ (estimated annual wage)[5]: $1767 ($1178/year)

Prevalence of low BMI[6]: 16.0% (95% CI: 7.4, 26.4)

Prevalence of anemia[7]: 44.3% (95% CI: 25.0, 65.1)

Baseline coverage of IFA[8]: 6.8%

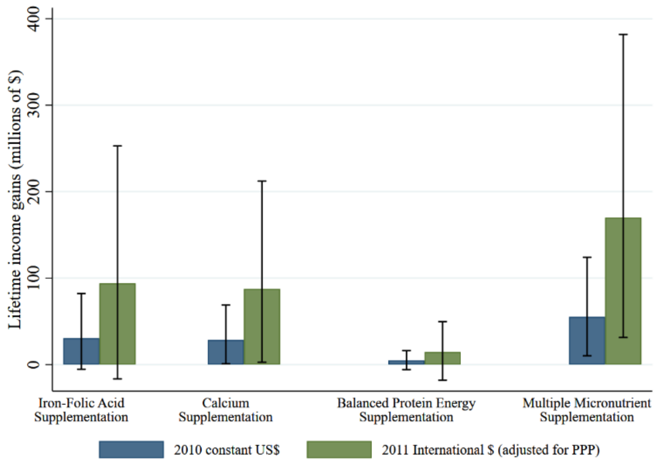

Figure: Benefits by birth cohort size for estimated gains in lifetime wages attributable to scaling up nutrition interventions to 90% target coverage

Table: Impact of maternal prenatal nutrition intervention on human capital and labour market outcomes, through improvements in low birthweight or preterm birth and schooling in Afghanistan.

Intervention	Target Coverage (%)	Absolute reduction in birth outcome (%)	Benefits by cohorts: School years gained (in 1000 s)	No. of additional students completing secondary school	Benefits by cohorts: Lifetime wages		Returns in lifetime earnings per child born to a targeted pregnant woman	
					in US $ millions	in International $ millions	in US $	in International $
**Iron/Folic Acid Suppl.**	90%	2.72 (-0.48, 5.34)	37.72 (-6.64, 102.71)	694 (-122, 1890)	30.52 (-5.38, 82.19)	93.90 (-16.56, 252.86)	5.63 (-0.99, 15.16)	17.32 (-3.05, 46.64)
**Calcium Suppl.**	50%	1.24 (0.28, 2.10)	19.77 (0.62, 46.91)	364 (11, 863)	15.82 (0.48, 38.31)	48.67 (1.48, 117.87)	5.25 (0.16, 12.72)	16.16 (0.49, 39.13)
	90%	2.23 (0.51, 3.77)	35.58 (1.11, 84.44)	655 (20, 1554)	28.47 (0.86, 68.96)	87.60 (2.66, 212.16)	5.25 (0.16, 12.72)	16.16 (0.49, 39.13)
**Multiple Micronutrient Suppl.**	50%	2.53 (1.08, 3.90)	37.06 (7.14, 82.39)	682 (131, 1516)	29.41 (5.62, 66.39)	90.50 (17.29, 204.27)	9.77 (1.87, 22.04)	30.05 (5.74, 67.82)
	90%	4.73 (1.91, 7.34)	69.34 (12.81, 154.09)	1276 (236, 2835)	55.23 (10.19, 124.12)	169.93 (31.36, 381.87)	10.19 (1.88, 22.89)	31.34 (5.78, 70.43)
**Balanced Protein Suppl.**	50%	0.25 (-0.27, 0.63)	3.32 (-4.12, 11.06)	61 (-76, 203)	2.63 (-3.25, 8.98)	8.10 (-10.00, 27.64)	6.05 (-7.01, 16.41)	18.61 (-21.58, 50.50)
	90%	0.46 (-0.48, 1.13)	5.98 (-7.41, 19.91)	110 (-136, 366)	4.74 (-5.85, 16.17)	14.58 (-18.00, 49.75)	6.05 (-7.01, 16.41)	18.61 (-21.58, 50.50)

References for Data Inputs

[1] Blencowe H, Krasevec J, Onis M De, et al. Articles National , regional , and worldwide estimates of low birthweight in 2015 , with trends from 2000: a systematic analysis. Lancet Glob Heal. 2019;(18):1-12.

[2] Chawanpaiboon S, Vogel JP, Moller AB, et al. Global, regional, and national estimates of levels of preterm birth in 2014: a systematic review and modelling analysis. Lancet Glob Heal. 2019;7(1):e37-e46.

[3] United National Population Division World Population Prospects 2019.

[4] Fink G, Peet E, Danaei G, et al. Schooling and wage income losses due to early-childhood growth faltering in developing countries: National, regional, and global estimates. Am J Clin Nutr. 2016;104(1):104-112.

[5] Country specific annual wage data from World Indicators Database. Average yearly wage was estimated to be 2/3 of the gross domestic product in 2010 constant US dollars and 2011 International dollars, adjusted for purchasing power parity.

[6] NCD Risk Factor Collaboration. Trends in adult body-mass index in 200 countries from 1975 to 2014: a pooled analysis of 1698 population-based measurement studies with 19·2 million participants. Lancet. 2016;387(10026):1377-1396.

[7] Stevens GA, Finucane MM, De-Regil LM, et al. Global, regional, and national trends in haemoglobin concentration and prevalence of total and severe anaemia in children and pregnant and non-pregnant women for 1995-2011: A systematic analysis of population-representative data. Lancet Glob Heal. 2013;1(1):16-25.

[8] Coverage of iron-folic acid supplementation abstracted from the most recent Demographic Health Survey or imputed based on sub-regional average. Indicator used: % women in the past five years who took iron tablets or syrup for >90 days.

Albania

Region: Central Europe, Eastern Europe, Central Asia**; Sub-region:** Central Europe

Low birthweight prevalence[1]: 4.6% (95% CI: 3.6, 5.8)

Preterm birth prevalence[2]: 8.7% (95% CI: 6.3, 13.3)

Number of births[3]: 170,000

Returns to education[4]: 6.3% (95% CI: 5.8, 6.8)

GDP per capita 2010 US$ (estimated annual wage)[5]: $4525 ($3016/year)

GDP per capita 2011 International $ (estimated annual wage)[5]: $10971 ($7314/year)

Prevalence of low BMI[6]: 2.0% (95% CI: 0.7, 4.3)

Prevalence of anemia[7]: 22.0% (95% CI: 14.9, 31.8)

Baseline coverage of IFA[8]: 18.5%

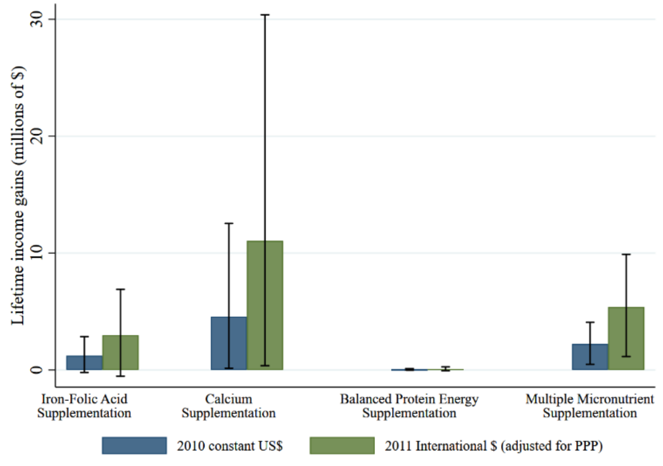

Figure: Benefits by birth cohort size for estimated gains in lifetime wages attributable to scaling up nutrition interventions to 90% target coverage

Table: Impact of maternal prenatal nutrition intervention on human capital and labour market outcomes, through improvements in low birthweight or preterm birth and schooling in Albania.

Intervention	Target Coverage (%)	Absolute reduction in birth outcome (%)	Benefits by cohorts: School years gained (in 1000 s)	No. of additional students completing secondary school	Benefits by cohorts: Lifetime wages		Returns in lifetime earnings per child born to a targeted pregnant woman	
					in US $ millions	in International $ millions	in US $	in International $
Iron/Folic Acid Suppl.	90%	0.55 (-0.10, 1.15)	0.24 (-0.04, 0.55)	17 (-3, 39)	1.23 (-0.22, 2.84)	2.97 (-0.53, 6.89)	8.01 (-1.44, 18.58)	19.42 (-3.48, 45.05)
Calcium Suppl.	50%	1.02 (0.21, 1.96)	0.48 (0.02, 1.35)	35 (1, 97)	2.53 (0.08, 6.96)	6.13 (0.20, 16.88)	29.75 (0.97, 81.91)	72.14 (2.35, 198.61)
	90%	1.84 (0.38, 3.53)	0.87 (0.03, 2.42)	63 (2, 175)	4.55 (0.15, 12.53)	11.04 (0.36, 30.39)	29.75 (0.97, 81.91)	72.14 (2.35, 198.61)
Multiple Micronutrient Suppl.	50%	0.46 (0.20, 0.71)	0.21 (0.05, 0.37)	15 (4, 27)	1.10 (0.26, 1.94)	2.66 (0.63, 4.69)	12.92 (3.05, 22.77)	31.32 (7.39, 55.22)
	90%	0.93 (0.34, 1.50)	0.43 (0.09, 0.78)	31 (7, 56)	2.22 (0.47, 4.08)	5.38 (1.14, 9.89)	14.50 (3.08, 26.66)	35.17 (7.47, 64.64)
Balanced Protein Suppl.	50%	0.01 (-0.01, 0.02)	0.00 (-0.00, 0.01)	0 (-0, 1)	0.02 (-0.02, 0.06)	0.04 (-0.04, 0.15)	10.26 (-8.55, 27.33)	24.87 (-20.72, 66.27)
	90%	0.01 (-0.01, 0.04)	0.01 (-0.01, 0.02)	0 (-0, 2)	0.03 (-0.03, 0.11)	0.07 (-0.07, 0.27)	10.26 (-8.55, 27.33)	24.87 (-20.72, 66.27)

References for Data Inputs

[1] Blencowe H, Krasevec J, Onis M De, et al. Articles National , regional , and worldwide estimates of low birthweight in 2015 , with trends from 2000: a systematic analysis. Lancet Glob Heal. 2019;(18):1-12.
[2] Chawanpaiboon S, Vogel JP, Moller AB, et al. Global, regional, and national estimates of levels of preterm birth in 2014: a systematic review and modelling analysis. Lancet Glob Heal. 2019;7(1):e37-e46.
[3] United National Population Division World Population Prospects 2019.
[4] Fink G, Peet E, Danaei G, et al. Schooling and wage income losses due to early-childhood growth faltering in developing countries: National, regional, and global estimates. Am J Clin Nutr. 2016;104(1):104-112.
[5] Country specific annual wage data from World Indicators Database. Average yearly wage was estimated to be 2/3 of the gross domestic product in 2010 constant US dollars and 2011 International dollars, adjusted for purchasing power parity.
[6] NCD Risk Factor Collaboration. Trends in adult body-mass index in 200 countries from 1975 to 2014: a pooled analysis of 1698 population-based measurement studies with 19·2 million participants. Lancet. 2016;387(10026):1377-1396.
[7] Stevens GA, Finucane MM, De-Regil LM, et al. Global, regional, and national trends in haemoglobin concentration and prevalence of total and severe anaemia in children and pregnant and non-pregnant women for 1995-2011: A systematic analysis of population-representative data. Lancet Glob Heal. 2013;1(1):16-25.
[8] Coverage of iron-folic acid supplementation abstracted from the most recent Demographic Health Survey or imputed based on sub-regional average. Indicator used: % women in the past five years who took iron tablets or syrup for >90 days.

Algeria

Region: North Africa and Middle East**; Sub-region:** North Africa and Middle East

Low birthweight prevalence[1]: 7.3% (95% CI: 5.7, 9.6)

Preterm birth prevalence[2]: 13.4% (95% CI: 6.3, 30.9)

Number of births[3]: 5,159,000

Returns to education[4]: 6.7% (95% CI: 4.1, 9.4)

GDP per capita 2010 US$ (estimated annual wage)[5]: $4777 ($3185/year)

GDP per capita 2011 International $ (estimated annual wage)[5]: $13774 ($9183/year)

Prevalence of low BMI[6]: 3.3% (95% CI: 1.4, 6.5)

Prevalence of anemia[7]: 31.6% (95% CI: 15.9, 56.3)

Baseline coverage of IFA[8]: 24.3%

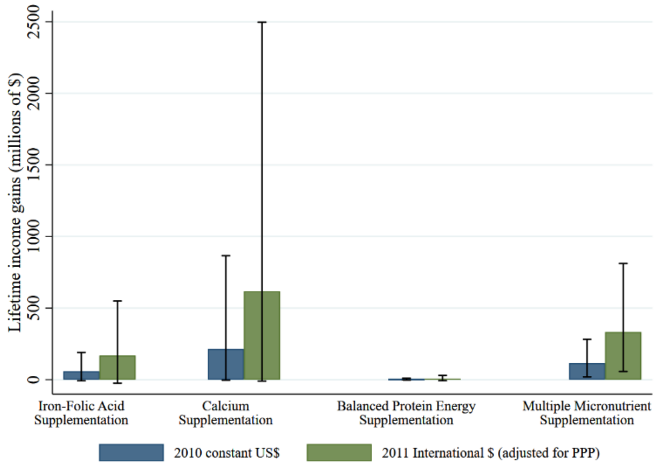

Figure: Benefits by birth cohort size for estimated gains in lifetime wages attributable to scaling up nutrition interventions to 90% target coverage

Table: Impact of maternal prenatal nutrition intervention on human capital and labour market outcomes, through improvements in low birthweight or preterm birth and schooling in Algeria.

Intervention	Target Coverage (%)	Absolute reduction in birth outcome (%)	Benefits by cohorts: School years gained (in 1000 s)	No. of additional students completing secondary school	Benefits by cohorts: Lifetime wages		Returns in lifetime earnings per child born to a targeted pregnant woman	
					in US $ millions	in International $ millions	in US $	in International $
Iron/Folic Acid Suppl.	90%	0.78 (-0.14, 1.76)	10.27 (-1.55, 30.29)	105 (-16, 309)	58.25 (-8.50, 190.46)	167.97 (-24.50, 549.22)	12.55 (-1.83, 41.02)	36.18 (-5.28, 118.29)
Calcium Suppl.	50%	1.45 (0.03, 3.87)	20.78 (-0.39, 78.76)	212 (-4, 803)	118.83 (-1.95, 481.02)	342.66 (-5.62, 1387.07)	46.07 (-0.76, 186.48)	132.84 (-2.18, 537.73)
	90%	2.61 (0.05, 6.97)	37.40 (-0.70, 141.77)	381 (-7, 1446)	213.90 (-3.51, 865.84)	616.79 (-10.11, 2496.73)	46.07 (-0.76, 186.48)	132.84 (-2.18, 537.73)
Multiple Micronutrient Suppl.	50%	0.69 (0.37, 1.01)	9.37 (2.16, 20.06)	96 (22, 205)	53.99 (11.11, 127.50)	155.68 (32.03, 367.65)	20.93 (4.31, 49.43)	60.35 (12.42, 142.53)
	90%	1.46 (0.67, 2.30)	19.88 (4.04, 44.59)	203 (41, 455)	115.31 (19.70, 281.36)	332.51 (56.81, 811.34)	24.84 (4.24, 60.60)	71.61 (12.24, 174.74)
Balanced Protein Suppl.	50%	0.02 (-0.02, 0.06)	0.21 (-0.27, 0.97)	2 (-3, 10)	1.19 (-1.52, 5.75)	3.44 (-4.39, 16.58)	16.76 (-15.71, 57.69)	48.34 (-45.31, 166.36)
	90%	0.03 (-0.03, 0.11)	0.38 (-0.48, 1.75)	4 (-5, 18)	2.15 (-2.74, 10.35)	6.20 (-7.91, 29.84)	16.76 (-15.71, 57.69)	48.34 (-45.31, 166.36)

References for Data Inputs

[1] Blencowe H, Krasevec J, Onis M De, et al. Articles National , regional , and worldwide estimates of low birthweight in 2015 , with trends from 2000: a systematic analysis. Lancet Glob Heal. 2019;(18):1-12.

[2] Chawanpaiboon S, Vogel JP, Moller AB, et al. Global, regional, and national estimates of levels of preterm birth in 2014: a systematic review and modelling analysis. Lancet Glob Heal. 2019;7(1):e37-e46.

[3] United National Population Division World Population Prospects 2019.

[4] Fink G, Peet E, Danaei G, et al. Schooling and wage income losses due to early-childhood growth faltering in developing countries: National, regional, and global estimates. Am J Clin Nutr. 2016;104(1):104-112.

[5] Country specific annual wage data from World Indicators Database. Average yearly wage was estimated to be 2/3 of the gross domestic product in 2010 constant US dollars and 2011 International dollars, adjusted for purchasing power parity.

[6] NCD Risk Factor Collaboration. Trends in adult body-mass index in 200 countries from 1975 to 2014: a pooled analysis of 1698 population-based measurement studies with 19·2 million participants. Lancet. 2016;387(10026):1377-1396.

[7] Stevens GA, Finucane MM, De-Regil LM, et al. Global, regional, and national trends in haemoglobin concentration and prevalence of total and severe anaemia in children and pregnant and non-pregnant women for 1995-2011: A systematic analysis of population-representative data. Lancet Glob Heal. 2013;1(1):16-25.

[8] Coverage of iron-folic acid supplementation abstracted from the most recent Demographic Health Survey or imputed based on sub-regional average. Indicator used: % women in the past five years who took iron tablets or syrup for >90 days.

Angola

Region: Sub-Saharan Africa**; Sub-region:** Central Sub-Saharan Africa

Low birthweight prevalence[1]: 15.3% (95% CI: 11.8, 21.4)

Preterm birth prevalence[2]: 12.0% (95% CI: 8.6, 16.7)

Number of births[3]: 6,215,000

Returns to education[4]: 9.9% (95% CI: 7.0, 12.9)

GDP per capita 2010 US$ (estimated annual wage)[5]: $3748 ($2499/year)

GDP per capita 2011 International $ (estimated annual wage)[5]: $6645 ($4430/year)

Prevalence of low BMI[6]: 10.8% (95% CI: 3.8, 20.9)

Prevalence of anemia[7]: 47.8% (95% CI: 31.9, 62.6)

Baseline coverage of IFA[8]: 32.1%

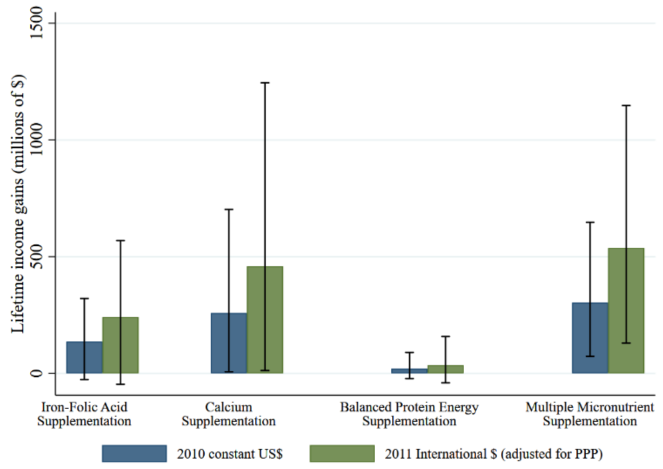

Figure: Benefits by birth cohort size for estimated gains in lifetime wages attributable to scaling up nutrition interventions to 90% target coverage

Table: Impact of maternal prenatal nutrition intervention on human capital and labour market outcomes, through improvements in low birthweight or preterm birth and schooling in Angola.

Intervention	Target Coverage (%)	Absolute reduction in birth outcome (%)	Benefits by cohorts: School years gained (in 1000 s)	No. of additional students completing secondary school	Benefits by cohorts: Lifetime wages		Returns in lifetime earnings per child born to a targeted pregnant woman	
					in US $ millions	in International $ millions	in US $	in International $
Iron/Folic Acid Suppl.	90%	1.50 (-0.36, 2.10)	20.88 (-4.98, 45.53)	1507 (-360, 3287)	136.00 (-26.34, 320.77)	241.10 (-46.69, 568.64)	24.31 (-4.71, 57.35)	43.10 (-8.35, 101.66)
Calcium Suppl.	50%	1.41 (0.31, 2.62)	22.63 (0.56, 56.63)	1634 (40, 4089)	143.77 (3.87, 390.10)	254.86 (6.87, 691.55)	46.26 (1.25, 125.53)	82.02 (2.21, 222.54)
	90%	2.54 (0.56, 4.71)	40.73 (1.01, 101.94)	2941 (73, 7360)	258.78 (6.97, 702.18)	458.75 (12.36, 1244.79)	46.26 (1.25, 125.53)	82.02 (2.21, 222.54)
Multiple Micronutrient Suppl.	50%	1.35 (0.96, 1.75)	20.88 (5.03, 41.73)	1508 (363, 3013)	137.14 (31.63, 300.44)	243.11 (56.08, 532.61)	44.13 (10.18, 96.68)	78.23 (18.05, 171.40)
	90%	3.10 (1.88, 3.76)	46.14 (10.80, 92.55)	3331 (780, 6682)	302.96 (72.98, 647.05)	537.07 (129.38, 1147.06)	54.16 (13.05, 115.68)	96.02 (23.13, 205.07)
Balanced Protein Suppl.	50%	0.12 (-0.10, 0.42)	1.67 (-1.76, 7.31)	121 (-127, 527)	11.02 (-12.67, 49.56)	19.54 (-22.46, 87.86)	35.02 (-38.23, 126.98)	62.08 (-67.78, 225.10)
	90%	0.22 (-0.18, 0.75)	3.01 (-3.18, 13.15)	217 (-229, 949)	19.84 (-22.81, 89.21)	35.18 (-40.44, 158.15)	35.02 (-38.23, 126.98)	62.08 (-67.78, 225.10)

References for Data Inputs

[1] Blencowe H, Krasevec J, Onis M De, et al. Articles National , regional , and worldwide estimates of low birthweight in 2015 , with trends from 2000: a systematic analysis. Lancet Glob Heal. 2019;(18):1-12.

[2] Chawanpaiboon S, Vogel JP, Moller AB, et al. Global, regional, and national estimates of levels of preterm birth in 2014: a systematic review and modelling analysis. Lancet Glob Heal. 2019;7(1):e37-e46.

[3] United National Population Division World Population Prospects 2019.

[4] Fink G, Peet E, Danaei G, et al. Schooling and wage income losses due to early-childhood growth faltering in developing countries: National, regional, and global estimates. Am J Clin Nutr. 2016;104(1):104-112.

[5] Country specific annual wage data from World Indicators Database. Average yearly wage was estimated to be 2/3 of the gross domestic product in 2010 constant US dollars and 2011 International dollars, adjusted for purchasing power parity.

[6] NCD Risk Factor Collaboration. Trends in adult body-mass index in 200 countries from 1975 to 2014: a pooled analysis of 1698 population-based measurement studies with 19·2 million participants. Lancet. 2016;387(10026):1377-1396.

[7] Stevens GA, Finucane MM, De-Regil LM, et al. Global, regional, and national trends in haemoglobin concentration and prevalence of total and severe anaemia in children and pregnant and non-pregnant women for 1995-2011: A systematic analysis of population-representative data. Lancet Glob Heal. 2013;1(1):16-25.

[8] Coverage of iron-folic acid supplementation abstracted from the most recent Demographic Health Survey or imputed based on sub-regional average. Indicator used: % women in the past five years who took iron tablets or syrup for >90 days.

Argentina

Region: Latin America and Caribbean**; Sub-region:** Southern Latin America

Low birthweight prevalence[1]: 7.3% (95% CI: 7.1, 7.6)

Preterm birth prevalence[2]: 8.4% (95% CI: 6.7, 10.2)

Number of births[3]: 3,776,000

Returns to education[4]: 12.1% (95% CI: 10.2, 14.1)

GDP per capita 2010 US$ (estimated annual wage)[5]: $10568 ($7045/year)

GDP per capita 2011 International $ (estimated annual wage)[5]: $19244 ($12829/year)

Prevalence of low BMI[6]: 1.2% (95% CI: 0.5, 2.3)

Prevalence of anemia[7]: 28.2% (95% CI: 10.7, 53.5)

Baseline coverage of IFA[8]: 34.2%

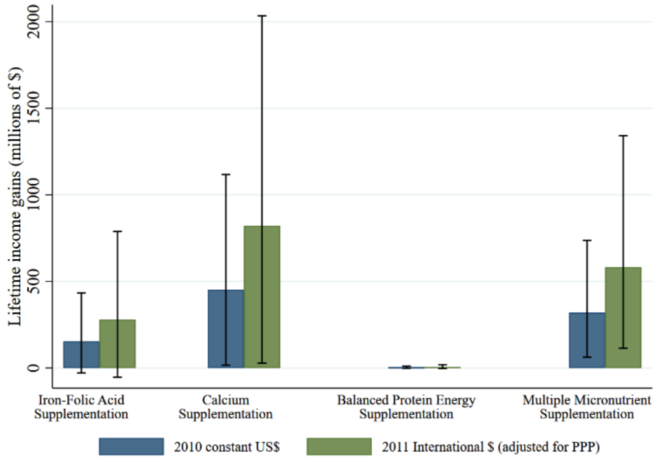

Figure: Benefits by birth cohort size for estimated gains in lifetime wages attributable to scaling up nutrition interventions to 90% target coverage

Table: Impact of maternal prenatal nutrition intervention on human capital and labour market outcomes, through improvements in low birthweight or preterm birth and schooling in Argentina.

Intervention	Target Coverage (%)	Absolute reduction in birth outcome (%)	Benefits by cohorts: School years gained (in 1000 s)	No. of additional students completing secondary school	Benefits by cohorts: Lifetime wages		Returns in lifetime earnings per child born to a targeted pregnant woman	
					in US $ millions	in International $ millions	in US $	in International $
Iron/Folic Acid Suppl.	90%	0.69 (-0.13, 1.42)	6.71 (-1.18, 18.28)	130 (-23, 355)	153.53 (-29.18, 433.06)	279.56 (-53.13, 788.57)	45.18 (-8.59, 127.43)	82.26 (-15.63, 232.04)
Calcium Suppl.	50%	1.00 (0.22, 1.73)	10.92 (0.36, 26.45)	212 (7, 513)	250.76 (8.68, 620.78)	456.60 (15.81, 1130.37)	132.82 (4.60, 328.80)	241.84 (8.37, 598.71)
	90%	1.79 (0.39, 3.12)	19.66 (0.65, 47.62)	381 (13, 924)	451.36 (15.62, 1117.40)	821.89 (28.45, 2034.67)	132.82 (4.60, 328.80)	241.84 (8.37, 598.71)
Multiple Micronutrient Suppl.	50%	0.58 (0.32, 0.81)	5.95 (1.38, 12.28)	115 (27, 238)	135.85 (30.97, 295.28)	247.37 (56.40, 537.67)	71.96 (16.41, 156.40)	131.02 (29.87, 284.78)
	90%	1.38 (0.63, 2.12)	13.78 (2.78, 30.38)	267 (54, 589)	319.69 (62.49, 737.18)	582.12 (113.79, 1342.34)	94.07 (18.39, 216.92)	171.29 (33.48, 394.99)
Balanced Protein Suppl.	50%	0.01 (-0.01, 0.02)	0.06 (-0.05, 0.25)	1 (-1, 5)	1.43 (-1.19, 5.86)	2.60 (-2.17, 10.66)	70.45 (-64.32, 215.86)	128.27 (-117.12, 393.06)
	90%	0.01 (-0.01, 0.04)	0.11 (-0.10, 0.44)	2 (-2, 9)	2.57 (-2.15, 10.54)	4.68 (-3.91, 19.19)	70.45 (-64.32, 215.86)	128.27 (-117.12, 393.06)

References for Data Inputs
[1] Blencowe H, Krasevec J, Onis M De, et al. Articles National , regional , and worldwide estimates of low birthweight in 2015 , with trends from 2000: a systematic analysis. Lancet Glob Heal. 2019;(18):1-12.
[2] Chawanpaiboon S, Vogel JP, Moller AB, et al. Global, regional, and national estimates of levels of preterm birth in 2014: a systematic review and modelling analysis. Lancet Glob Heal. 2019;7(1):e37-e46.
[3] United National Population Division World Population Prospects 2019.
[4] Fink G, Peet E, Danaei G, et al. Schooling and wage income losses due to early-childhood growth faltering in developing countries: National, regional, and global estimates. Am J Clin Nutr. 2016;104(1):104-112.
[5] Country specific annual wage data from World Indicators Database. Average yearly wage was estimated to be 2/3 of the gross domestic product in 2010 constant US dollars and 2011 International dollars, adjusted for purchasing power parity.
[6] NCD Risk Factor Collaboration. Trends in adult body-mass index in 200 countries from 1975 to 2014: a pooled analysis of 1698 population-based measurement studies with 19·2 million participants. Lancet. 2016;387(10026):1377-1396.
[7] Stevens GA, Finucane MM, De-Regil LM, et al. Global, regional, and national trends in haemoglobin concentration and prevalence of total and severe anaemia in children and pregnant and non-pregnant women for 1995-2011: A systematic analysis of population-representative data. Lancet Glob Heal. 2013;1(1):16-25.
[8] Coverage of iron-folic acid supplementation abstracted from the most recent Demographic Health Survey or imputed based on sub-regional average. Indicator used: % women in the past five years who took iron tablets or syrup for >90 days.

Armenia

Region: Central Europe, Eastern Europe, Central Asia**; Sub-region:** Central Asia

Low birthweight prevalence[1]: 9.0% (95% CI: 8.2, 9.7)

Preterm birth prevalence[2]: 10.4% (95% CI: 8.7, 11.9)

Number of births[3]: 209,000

Returns to education[4]: 5.1% (95% CI: 2.4, 7.8)

GDP per capita 2010 US$ (estimated annual wage)[5]: $3924 ($2616/year)

GDP per capita 2011 International $ (estimated annual wage)[5]: $8172 ($5448/year)

Prevalence of low BMI[6]: 3.3% (95% CI: 1.4, 6.2)

Prevalence of anemia[7]: 27.2% (95% CI: 15.7, 46.7)

Baseline coverage of IFA[8]: 4.5%

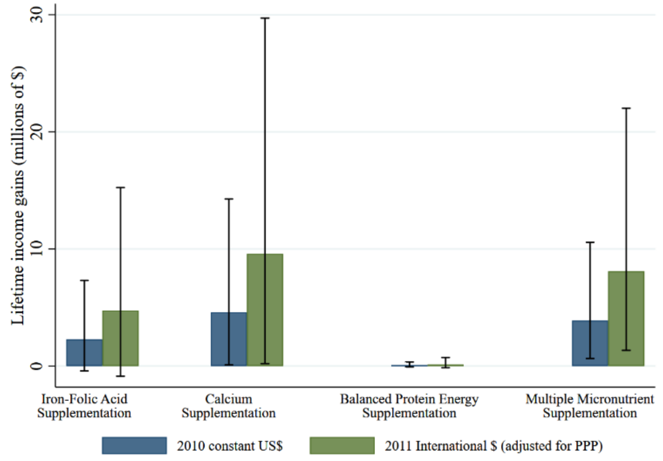

Figure: Benefits by birth cohort size for estimated gains in lifetime wages attributable to scaling up nutrition interventions to 90% target coverage

Table: Impact of maternal prenatal nutrition intervention on human capital and labour market outcomes, through improvements in low birthweight or preterm birth and schooling in Armenia.

Intervention	Target Coverage (%)	Absolute reduction in birth outcome (%)	Benefits by cohorts: School years gained (in 1000 s)	No. of additional students completing secondary school	Benefits by cohorts: Lifetime wages		Returns in lifetime earnings per child born to a targeted pregnant woman	
					in US $ millions	in International $ millions	in US $	in International $
Iron/Folic Acid Suppl.	90%	1.25 (-0.23, 2.40)	0.67 (-0.12, 1.73)	10 (-2, 26)	2.28 (-0.41, 7.32)	4.75 (-0.86, 15.24)	12.12 (-2.20, 38.89)	25.24 (-4.58, 81.00)
Calcium Suppl.	50%	1.26 (0.28, 2.13)	0.74 (0.02, 1.80)	11 (0, 27)	2.56 (0.06, 7.93)	5.32 (0.12, 16.51)	24.47 (0.54, 75.85)	50.96 (1.13, 157.97)
	90%	2.26 (0.50, 3.83)	1.34 (0.04, 3.25)	20 (1, 49)	4.60 (0.10, 14.27)	9.58 (0.21, 29.71)	24.47 (0.54, 75.85)	50.96 (1.13, 157.97)
Multiple Micronutrient Suppl.	50%	1.11 (0.40, 1.68)	0.61 (0.10, 1.33)	9 (2, 20)	2.11 (0.35, 5.73)	4.39 (0.73, 11.92)	20.18 (3.35, 54.79)	42.02 (6.97, 114.11)
	90%	2.05 (0.73, 3.12)	1.13 (0.18, 2.46)	17 (3, 37)	3.89 (0.64, 10.57)	8.09 (1.34, 22.02)	20.66 (3.42, 56.20)	43.02 (7.13, 117.05)
Balanced Protein Suppl.	50%	0.02 (-0.02, 0.07)	0.01 (-0.01, 0.05)	0 (-0, 1)	0.04 (-0.04, 0.20)	0.08 (-0.08, 0.41)	12.70 (-13.03, 51.06)	26.45 (-27.15, 106.34)
	90%	0.04 (-0.04, 0.13)	0.02 (-0.02, 0.08)	0 (-0, 1)	0.07 (-0.07, 0.35)	0.14 (-0.15, 0.73)	12.70 (-13.03, 51.06)	26.45 (-27.15, 106.34)

References for Data Inputs

[1] Blencowe H, Krasevec J, Onis M De, et al. Articles National , regional , and worldwide estimates of low birthweight in 2015 , with trends from 2000: a systematic analysis. Lancet Glob Heal. 2019;(18):1-12.
[2] Chawanpaiboon S, Vogel JP, Moller AB, et al. Global, regional, and national estimates of levels of preterm birth in 2014: a systematic review and modelling analysis. Lancet Glob Heal. 2019;7(1):e37-e46.
[3] United National Population Division World Population Prospects 2019.
[4] Fink G, Peet E, Danaei G, et al. Schooling and wage income losses due to early-childhood growth faltering in developing countries: National, regional, and global estimates. Am J Clin Nutr. 2016;104(1):104-112.
[5] Country specific annual wage data from World Indicators Database. Average yearly wage was estimated to be 2/3 of the gross domestic product in 2010 constant US dollars and 2011 International dollars, adjusted for purchasing power parity.
[6] NCD Risk Factor Collaboration. Trends in adult body-mass index in 200 countries from 1975 to 2014: a pooled analysis of 1698 population-based measurement studies with 19·2 million participants. Lancet. 2016;387(10026):1377-1396.
[7] Stevens GA, Finucane MM, De-Regil LM, et al. Global, regional, and national trends in haemoglobin concentration and prevalence of total and severe anaemia in children and pregnant and non-pregnant women for 1995-2011: A systematic analysis of population-representative data. Lancet Glob Heal. 2013;1(1):16-25.
[8] Coverage of iron-folic acid supplementation abstracted from the most recent Demographic Health Survey or imputed based on sub-regional average. Indicator used: % women in the past five years who took iron tablets or syrup for >90 days.

Azerbaijan

Region: Central Europe, Eastern Europe, Central Asia**; Sub-region:** Central Asia

Low birthweight prevalence[1]: 7.3% (95% CI: 6.4, 8.2)

Preterm birth prevalence[2]: 10.4% (95% CI: 8.7, 11.9)

Number of births[3]: 844,000

Returns to education[4]: 3.9% (95% CI: 3.3, 4.4)

GDP per capita 2010 US$ (estimated annual wage)[5]: $6064 ($4042/year)

GDP per capita 2011 International $ (estimated annual wage)[5]: $16829 ($11219/year)

Prevalence of low BMI[6]: 2.9% (95% CI: 1.1, 6.1)

Prevalence of anemia[7]: 31.1% (95% CI: 19.5, 46.1)

Baseline coverage of IFA[8]: 3.0%

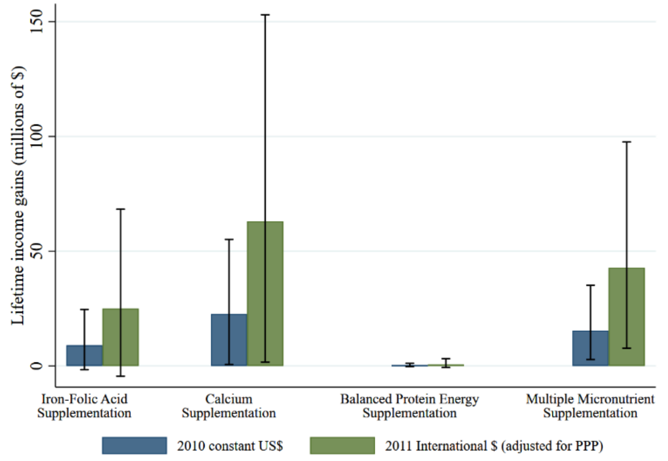

Figure: Benefits by birth cohort size for estimated gains in lifetime wages attributable to scaling up nutrition interventions to 90% target coverage

Table: Impact of maternal prenatal nutrition intervention on human capital and labour market outcomes, through improvements in low birthweight or preterm birth and schooling in Azerbaijan.

Intervention	Target Coverage (%)	Absolute reduction in birth outcome (%)	Benefits by cohorts: School years gained (in 1000 s)	No. of additional students completing secondary school	Benefits by cohorts: Lifetime wages		Returns in lifetime earnings per child born to a targeted pregnant woman	
					in US $ millions	in International $ millions	in US $	in International $
Iron/Folic Acid Suppl.	90%	1.02 (-0.19, 1.99)	2.14 (-0.39, 5.65)	210 (-38, 554)	9.00 (-1.61, 24.60)	24.97 (-4.46, 68.28)	11.85 (-2.11, 32.39)	32.88 (-5.87, 89.89)
Calcium Suppl.	50%	1.24 (0.27, 2.09)	2.95 (0.09, 7.19)	289 (9, 706)	12.62 (0.34, 30.64)	35.02 (0.94, 85.03)	29.90 (0.80, 72.60)	82.98 (2.22, 201.49)
	90%	2.23 (0.49, 3.76)	5.30 (0.16, 12.95)	521 (16, 1272)	22.71 (0.61, 55.15)	63.03 (1.69, 153.05)	29.90 (0.80, 72.60)	82.98 (2.22, 201.49)
Multiple Micronutrient Suppl.	50%	0.92 (0.34, 1.40)	2.01 (0.36, 4.49)	197 (35, 441)	8.44 (1.52, 19.19)	23.41 (4.22, 53.26)	19.99 (3.60, 45.47)	55.49 (9.99, 126.20)
	90%	1.68 (0.61, 2.57)	3.67 (0.66, 8.27)	361 (65, 812)	15.42 (2.79, 35.17)	42.80 (7.73, 97.60)	20.30 (3.67, 46.30)	56.35 (10.18, 128.49)
Balanced Protein Suppl.	50%	0.02 (-0.02, 0.05)	0.03 (-0.03, 0.15)	3 (-3, 15)	0.13 (-0.14, 0.63)	0.36 (-0.40, 1.75)	12.70 (-12.29, 38.87)	35.24 (-34.11, 107.88)
	90%	0.03 (-0.03, 0.10)	0.06 (-0.06, 0.28)	6 (-6, 27)	0.24 (-0.26, 1.13)	0.65 (-0.71, 3.15)	12.70 (-12.29, 38.87)	35.24 (-34.11, 107.88)

References for Data Inputs

[1] Blencowe H, Krasevec J, Onis M De, et al. Articles National , regional , and worldwide estimates of low birthweight in 2015 , with trends from 2000: a systematic analysis. Lancet Glob Heal. 2019;(18):1-12.

[2] Chawanpaiboon S, Vogel JP, Moller AB, et al. Global, regional, and national estimates of levels of preterm birth in 2014: a systematic review and modelling analysis. Lancet Glob Heal. 2019;7(1):e37-e46.

[3] United National Population Division World Population Prospects 2019.

[4] Fink G, Peet E, Danaei G, et al. Schooling and wage income losses due to early-childhood growth faltering in developing countries: National, regional, and global estimates. Am J Clin Nutr. 2016;104(1):104-112.

[5] Country specific annual wage data from World Indicators Database. Average yearly wage was estimated to be 2/3 of the gross domestic product in 2010 constant US dollars and 2011 International dollars, adjusted for purchasing power parity.

[6] NCD Risk Factor Collaboration. Trends in adult body-mass index in 200 countries from 1975 to 2014: a pooled analysis of 1698 population-based measurement studies with 19·2 million participants. Lancet. 2016;387(10026):1377-1396.

[7] Stevens GA, Finucane MM, De-Regil LM, et al. Global, regional, and national trends in haemoglobin concentration and prevalence of total and severe anaemia in children and pregnant and non-pregnant women for 1995-2011: A systematic analysis of population-representative data. Lancet Glob Heal. 2013;1(1):16-25.

[8] Coverage of iron-folic acid supplementation abstracted from the most recent Demographic Health Survey or imputed based on sub-regional average. Indicator used: % women in the past five years who took iron tablets or syrup for >90 days.

Bangladesh

Region: South Asia**; Sub-region:** South Asia

Low birthweight prevalence[1]: 27.8% (95% CI: 19.6, 38.5)

Preterm birth prevalence[2]: 19.1% (95% CI: 13.2, 26.2)

Number of births[3]: 14,732,000

Returns to education[4]: 7.1% (95% CI: 6.7, 7.5)

GDP per capita 2010 US$ (estimated annual wage)[5]: $1002 ($668/year)

GDP per capita 2011 International $ (estimated annual wage)[5]: $3232 ($2154/year)

Prevalence of low BMI[6]: 23.3% (95% CI: 16.6, 30.3)

Prevalence of anemia[7]: 48.2% (95% CI: 38.3, 57.6)

Baseline coverage of IFA[8]: 26.0%

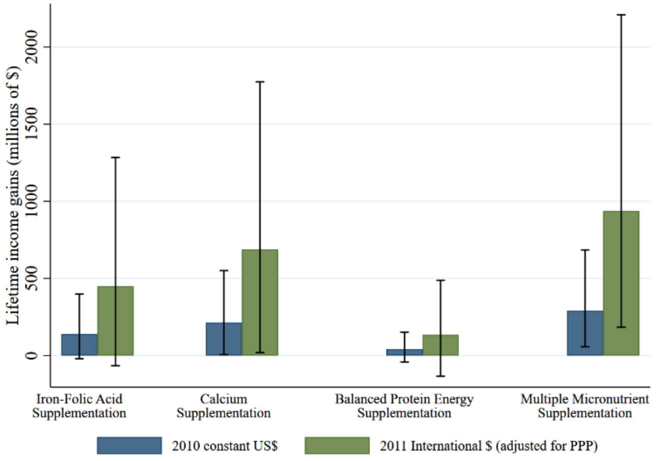

Figure: Benefits by birth cohort size for estimated gains in lifetime wages attributable to scaling up nutrition interventions to 90% target coverage

Table: Impact of maternal prenatal nutrition intervention on human capital and labour market outcomes, through improvements in low birthweight or preterm birth and schooling in Bangladesh.

Intervention	Target Coverage (%)	Absolute reduction in birth outcome (%)	Benefits by cohorts: School years gained (in 1000 s)	No. of additional students completing secondary school	Benefits by cohorts: Lifetime wages		Returns in lifetime earnings per child born to a targeted pregnant woman	
					in US $ millions	in International $ millions	in US $	in International $
Iron/Folic Acid Suppl.	90%	2.94 (-0.54, 6.52)	108.74 (-16.10, 311.85)	4415 (-654, 12661)	139.46 (-20.48, 398.31)	449.60 (-66.02, 1284.15)	10.52 (-1.54, 30.04)	33.91 (-4.98, 96.85)
Calcium Suppl.	50%	2.23 (0.43, 4.24)	91.81 (2.66, 239.80)	3728 (108, 9736)	118.65 (3.33, 305.72)	382.54 (10.74, 985.64)	16.11 (0.45, 41.50)	51.93 (1.46, 133.81)
	90%	4.02 (0.78, 7.64)	165.26 (4.79, 431.65)	6710 (195, 17525)	213.58 (5.99, 550.30)	688.57 (19.33, 1774.16)	16.11 (0.45, 41.50)	51.93 (1.46, 133.81)
Multiple Micronutrient Suppl.	50%	2.74 (1.36, 4.32)	105.45 (21.97, 241.86)	4281 (892, 9820)	134.67 (28.23, 315.88)	434.19 (91.00, 1018.41)	18.28 (3.83, 42.88)	58.95 (12.35, 138.26)
	90%	5.90 (2.55, 9.68)	224.93 (44.10, 530.07)	9132 (1790, 21521)	290.78 (57.03, 685.29)	937.46 (183.86, 2209.38)	21.93 (4.30, 51.69)	70.70 (13.87, 166.64)
Balanced Protein Suppl.	50%	0.53 (-0.42, 1.36)	18.23 (-17.67, 65.67)	740 (-717, 2666)	23.18 (-23.00, 84.01)	74.75 (-74.16, 270.84)	14.28 (-13.68, 47.54)	46.04 (-44.11, 153.28)
	90%	0.96 (-0.75, 2.45)	32.81 (-31.80, 118.21)	1332 (-1291, 4799)	41.73 (-41.40, 151.22)	134.54 (-133.49, 487.52)	14.28 (-13.68, 47.54)	46.04 (-44.11, 153.28)

References for Data Inputs
[1] Blencowe H, Krasevec J, Onis M De, et al. Articles National , regional , and worldwide estimates of low birthweight in 2015 , with trends from 2000: a systematic analysis. Lancet Glob Heal. 2019;(18):1-12.
[2] Chawanpaiboon S, Vogel JP, Moller AB, et al. Global, regional, and national estimates of levels of preterm birth in 2014: a systematic review and modelling analysis. Lancet Glob Heal. 2019;7(1):e37-e46.
[3] United National Population Division World Population Prospects 2019.
[4] Fink G, Peet E, Danaei G, et al. Schooling and wage income losses due to early-childhood growth faltering in developing countries: National, regional, and global estimates. Am J Clin Nutr. 2016;104(1):104-112.
[5] Country specific annual wage data from World Indicators Database. Average yearly wage was estimated to be 2/3 of the gross domestic product in 2010 constant US dollars and 2011 International dollars, adjusted for purchasing power parity.
[6] NCD Risk Factor Collaboration. Trends in adult body-mass index in 200 countries from 1975 to 2014: a pooled analysis of 1698 population-based measurement studies with 19·2 million participants. Lancet. 2016;387(10026):1377-1396.
[7] Stevens GA, Finucane MM, De-Regil LM, et al. Global, regional, and national trends in haemoglobin concentration and prevalence of total and severe anaemia in children and pregnant and non-pregnant women for 1995-2011: A systematic analysis of population-representative data. Lancet Glob Heal. 2013;1(1):16-25.
[8] Coverage of iron-folic acid supplementation abstracted from the most recent Demographic Health Survey or imputed based on sub-regional average. Indicator used: % women in the past five years who took iron tablets or syrup for >90 days.

Belarus

Region: Central Europe, Eastern Europe, Central Asia**; Sub-region:** Eastern Europe

Low birthweight prevalence[1]: 5.1% (95% CI: 4.6, 5.5)

Preterm birth prevalence[2]: 4.3% (95% CI: 3.4, 5.4)

Number of births[3]: 559,000

Returns to education[4]: 6.7% (95% CI: 5.3, 8.1)

GDP per capita 2010 US$ (estimated annual wage)[5]: $6546 ($4364/year)

GDP per capita 2011 International $ (estimated annual wage)[5]: $17219 ($11479/year)

Prevalence of low BMI[6]: 2.0% (95% CI: 0.7, 4.4)

Prevalence of anemia[7]: 24.6% (95% CI: 11.0, 47.5)

Baseline coverage of IFA[8]: 18.5%

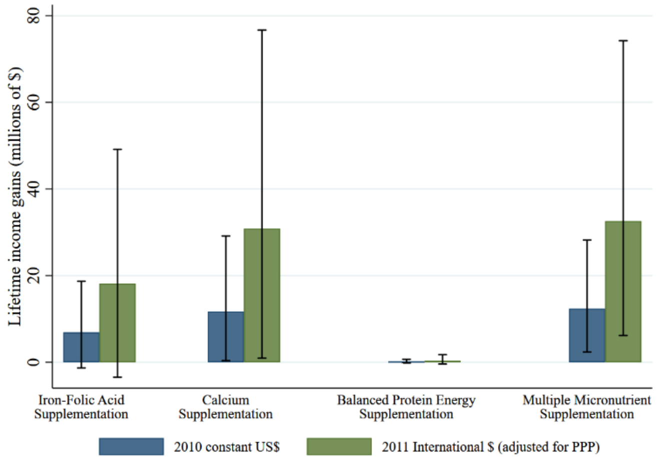

Figure: Benefits by birth cohort size for estimated gains in lifetime wages attributable to scaling up nutrition interventions to 90% target coverage

Table: Impact of maternal prenatal nutrition intervention on human capital and labour market outcomes, through improvements in low birthweight or preterm birth and schooling in Belarus.

Intervention	Target Coverage (%)	Absolute reduction in birth outcome (%)	Benefits by cohorts: School years gained (in 1000 s)	No. of additional students completing secondary school	Benefits by cohorts: Lifetime wages		Returns in lifetime earnings per child born to a targeted pregnant woman	
					in US $ millions	in International $ millions	in US $	in International $
Iron/Folic Acid Suppl.	90%	0.60 (-0.11, 1.21)	0.87 (-0.15, 2.30)	20 (-3, 51)	6.90 (-1.31, 18.69)	18.15 (-3.44, 49.16)	13.72 (-2.60, 37.15)	36.09 (-6.84, 97.72)
Calcium Suppl.	50%	0.52 (0.10, 0.91)	0.83 (0.03, 2.04)	19 (1, 45)	6.51 (0.20, 16.19)	17.14 (0.52, 42.60)	23.31 (0.71, 57.94)	61.31 (1.86, 152.41)
	90%	0.94 (0.19, 1.64)	1.50 (0.05, 3.66)	33 (1, 82)	11.73 (0.36, 29.15)	30.85 (0.94, 76.68)	23.31 (0.71, 57.94)	61.31 (1.86, 152.41)
Multiple Micronutrient Suppl.	50%	0.51 (0.21, 0.78)	0.77 (0.15, 1.68)	17 (3, 38)	6.06 (1.18, 13.62)	15.94 (3.10, 35.83)	21.68 (4.22, 48.73)	57.02 (11.10, 128.18)
	90%	1.05 (0.39, 1.64)	1.59 (0.29, 3.46)	36 (6, 77)	12.39 (2.35, 28.22)	32.59 (6.18, 74.23)	24.63 (4.67, 56.09)	64.78 (12.28, 147.55)
Balanced Protein Suppl.	50%	0.01 (-0.01, 0.03)	0.01 (-0.01, 0.05)	0 (-0, 1)	0.08 (-0.09, 0.37)	0.20 (-0.23, 0.96)	17.38 (-15.07, 54.15)	45.71 (-39.64, 142.45)
	90%	0.01 (-0.01, 0.05)	0.02 (-0.02, 0.08)	0 (-0, 2)	0.14 (-0.16, 0.66)	0.36 (-0.42, 1.73)	17.38 (-15.07, 54.15)	45.71 (-39.64, 142.45)

References for Data Inputs

[1] Blencowe H, Krasevec J, Onis M De, et al. Articles National , regional , and worldwide estimates of low birthweight in 2015 , with trends from 2000: a systematic analysis. Lancet Glob Heal. 2019;(18):1-12.

[2] Chawanpaiboon S, Vogel JP, Moller AB, et al. Global, regional, and national estimates of levels of preterm birth in 2014: a systematic review and modelling analysis. Lancet Glob Heal. 2019;7(1):e37-e46.

[3] United National Population Division World Population Prospects 2019.

[4] Fink G, Peet E, Danaei G, et al. Schooling and wage income losses due to early-childhood growth faltering in developing countries: National, regional, and global estimates. Am J Clin Nutr. 2016;104(1):104-112.

[5] Country specific annual wage data from World Indicators Database. Average yearly wage was estimated to be 2/3 of the gross domestic product in 2010 constant US dollars and 2011 International dollars, adjusted for purchasing power parity.

[6] NCD Risk Factor Collaboration. Trends in adult body-mass index in 200 countries from 1975 to 2014: a pooled analysis of 1698 population-based measurement studies with 19·2 million participants. Lancet. 2016;387(10026):1377-1396.

[7] Stevens GA, Finucane MM, De-Regil LM, et al. Global, regional, and national trends in haemoglobin concentration and prevalence of total and severe anaemia in children and pregnant and non-pregnant women for 1995-2011: A systematic analysis of population-representative data. Lancet Glob Heal. 2013;1(1):16-25.

[8] Coverage of iron-folic acid supplementation abstracted from the most recent Demographic Health Survey or imputed based on sub-regional average. Indicator used: % women in the past five years who took iron tablets or syrup for >90 days.

Belize

Region: Latin America and Caribbean**; Sub-region:** Caribbean

Low birthweight prevalence[1]: 8.6% (95% CI: 6.8, 11.1)

Preterm birth prevalence[2]: 9.8% (95% CI: 8.6, 11.3)

Number of births[3]: 40,000

Returns to education[4]: 9.8% (95% CI: 8.1, 11.5)

GDP per capita 2010 US$ (estimated annual wage)[5]: $4402 ($2935/year)

GDP per capita 2011 International $ (estimated annual wage)[5]: $7977 ($5318/year)

Prevalence of low BMI[6]: 2.8% (95% CI: 1.0, 5.9)

Prevalence of anemia[7]: 27.3% (95% CI: 12.1, 50.2)

Baseline coverage of IFA[8]: 62.3%

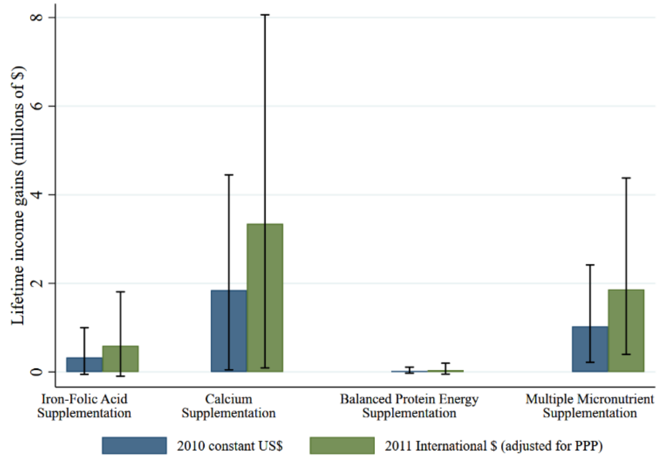

Figure: Benefits by birth cohort size for estimated gains in lifetime wages attributable to scaling up nutrition interventions to 90% target coverage

Table: Impact of maternal prenatal nutrition intervention on human capital and labour market outcomes, through improvements in low birthweight or preterm birth and schooling in Belize.

Intervention	Target Coverage (%)	Absolute reduction in birth outcome (%)	Benefits by cohorts: School years gained (in 1000 s)	No. of additional students completing secondary school	Benefits by cohorts: Lifetime wages		Returns in lifetime earnings per child born to a targeted pregnant woman	
					in US $ millions	in International $ millions	in US $	in International $
**Iron/Folic Acid Suppl.**	90%	0.42 (-0.07, 0.96)	0.04 (-0.01, 0.13)	3 (-0, 9)	0.33 (-0.05, 1.00)	0.59 (-0.09, 1.81)	9.06 (-1.45, 27.73)	16.42 (-2.63, 50.25)
**Calcium Suppl.**	50%	1.18 (0.27, 1.96)	0.13 (0.00, 0.32)	10 (0, 24)	1.03 (0.03, 2.47)	1.86 (0.05, 4.48)	51.29 (1.40, 123.61)	92.95 (2.54, 224.01)
	90%	2.12 (0.48, 3.53)	0.24 (0.01, 0.57)	18 (0, 43)	1.85 (0.05, 4.45)	3.35 (0.09, 8.06)	51.29 (1.40, 123.61)	92.95 (2.54, 224.01)
**Multiple Micronutrient Suppl.**	50%	0.50 (0.22, 0.79)	0.05 (0.01, 0.13)	4 (1, 9)	0.42 (0.08, 1.01)	0.77 (0.14, 1.82)	21.13 (3.79, 50.26)	38.30 (6.86, 91.08)
	90%	1.24 (0.63, 2.00)	0.13 (0.03, 0.30)	10 (2, 23)	1.03 (0.22, 2.42)	1.86 (0.40, 4.38)	28.57 (6.09, 67.11)	51.78 (11.03, 121.62)
**Balanced Protein Suppl.**	50%	0.02 (-0.02, 0.06)	0.00 (-0.00, 0.01)	0 (-0, 1)	0.01 (-0.02, 0.06)	0.02 (-0.03, 0.11)	27.18 (-29.22, 88.49)	49.26 (-52.96, 160.35)
	90%	0.03 (-0.03, 0.11)	0.00 (-0.00, 0.01)	0 (-0, 1)	0.02 (-0.03, 0.11)	0.04 (-0.05, 0.20)	27.18 (-29.22, 88.49)	49.26 (-52.96, 160.35)

References for Data Inputs
[1] Blencowe H, Krasevec J, Onis M De, et al. Articles National , regional , and worldwide estimates of low birthweight in 2015 , with trends from 2000: a systematic analysis. Lancet Glob Heal. 2019;(18):1-12.
[2] Chawanpaiboon S, Vogel JP, Moller AB, et al. Global, regional, and national estimates of levels of preterm birth in 2014: a systematic review and modelling analysis. Lancet Glob Heal. 2019;7(1):e37-e46.
[3] United National Population Division World Population Prospects 2019.
[4] Fink G, Peet E, Danaei G, et al. Schooling and wage income losses due to early-childhood growth faltering in developing countries: National, regional, and global estimates. Am J Clin Nutr. 2016;104(1):104-112.
[5] Country specific annual wage data from World Indicators Database. Average yearly wage was estimated to be 2/3 of the gross domestic product in 2010 constant US dollars and 2011 International dollars, adjusted for purchasing power parity.
[6] NCD Risk Factor Collaboration. Trends in adult body-mass index in 200 countries from 1975 to 2014: a pooled analysis of 1698 population-based measurement studies with 19·2 million participants. Lancet. 2016;387(10026):1377-1396.
[7] Stevens GA, Finucane MM, De-Regil LM, et al. Global, regional, and national trends in haemoglobin concentration and prevalence of total and severe anaemia in children and pregnant and non-pregnant women for 1995-2011: A systematic analysis of population-representative data. Lancet Glob Heal. 2013;1(1):16-25.
[8] Coverage of iron-folic acid supplementation abstracted from the most recent Demographic Health Survey or imputed based on sub-regional average. Indicator used: % women in the past five years who took iron tablets or syrup for >90 days.

Benin

Region: Sub-Saharan Africa**; Sub-region:** Western Sub-Saharan Africa

Low birthweight prevalence[1]: 16.9% (95% CI: 13.3, 21.3)

Preterm birth prevalence[2]: 9.3% (95% CI: 6.0, 13.6)

Number of births[3]: 2,066,000

Returns to education[4]: 6.3% (95% CI: 4.5, 8.1)

GDP per capita 2010 US$ (estimated annual wage)[5]: $828 ($552/year)

GDP per capita 2011 International $ (estimated annual wage)[5]: $1987 ($1325/year)

Prevalence of low BMI[6]: 8.5% (95% CI: 4.4, 14.2)

Prevalence of anemia[7]: 64.6% (95% CI: 55.6, 71.3)

Baseline coverage of IFA[8]: 47.1%

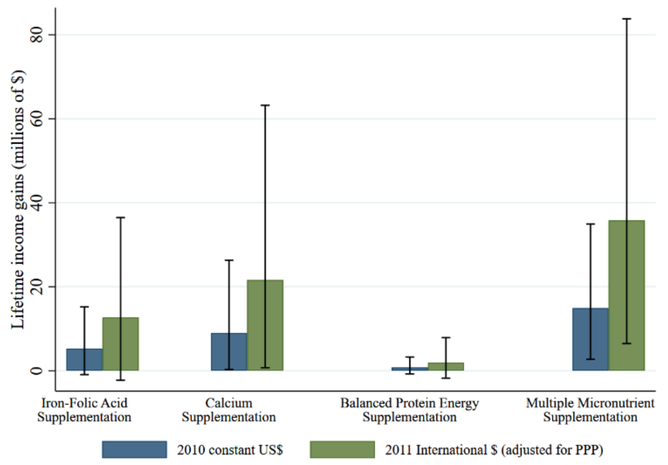

Figure: Benefits by birth cohort size for estimated gains in lifetime wages attributable to scaling up nutrition interventions to 90% target coverage

Table: Impact of maternal prenatal nutrition intervention on human capital and labour market outcomes, through improvements in low birthweight or preterm birth and schooling in Benin.

Intervention	Target Coverage (%)	Absolute reduction in birth outcome (%)	Benefits by cohorts: School years gained (in 1000 s)	No. of additional students completing secondary school	Benefits by cohorts: Lifetime wages		Returns in lifetime earnings per child born to a targeted pregnant woman	
					in US $ millions	in International $ millions	in US $	in International $
Iron/Folic Acid Suppl.	90%	1.26 (-0.22, 2.70)	5.75 (-0.97, 16.07)	0 (-0, 0)	5.29 (-0.93, 15.19)	12.69 (-2.24, 36.47)	2.84 (-0.50, 8.17)	6.82 (-1.21, 19.61)
Calcium Suppl.	50%	1.09 (0.22, 2.05)	5.42 (0.24, 15.12)	0 (0, 0)	5.00 (0.17, 14.63)	12.01 (0.41, 35.12)	4.84 (0.17, 14.16)	11.63 (0.40, 34.00)
	90%	1.95 (0.40, 3.69)	9.76 (0.42, 27.21)	0 (0, 0)	9.01 (0.31, 26.34)	21.62 (0.74, 63.22)	4.84 (0.17, 14.16)	11.63 (0.40, 34.00)
Multiple Micronutrient Suppl.	50%	1.26 (0.75, 1.79)	6.27 (1.29, 13.62)	0 (0, 0)	5.75 (1.06, 13.21)	13.80 (2.55, 31.71)	5.56 (1.03, 12.79)	13.36 (2.47, 30.70)
	90%	3.35 (1.88, 4.98)	16.30 (2.95, 36.62)	0 (0, 0)	14.91 (2.70, 34.92)	35.80 (6.48, 83.83)	8.02 (1.45, 18.78)	19.25 (3.48, 45.09)
Balanced Protein Suppl.	50%	0.11 (-0.10, 0.32)	0.48 (-0.50, 1.91)	0 (-0, 0)	0.45 (-0.41, 1.82)	1.08 (-0.98, 4.37)	5.54 (-5.38, 18.64)	13.30 (-12.91, 44.74)
	90%	0.20 (-0.18, 0.58)	0.87 (-0.91, 3.43)	0 (-0, 0)	0.81 (-0.73, 3.28)	1.95 (-1.76, 7.87)	5.54 (-5.38, 18.64)	13.30 (-12.91, 44.74)

References for Data Inputs

[1] Blencowe H, Krasevec J, Onis M De, et al. Articles National , regional , and worldwide estimates of low birthweight in 2015 , with trends from 2000: a systematic analysis. Lancet Glob Heal. 2019;(18):1-12.
[2] Chawanpaiboon S, Vogel JP, Moller AB, et al. Global, regional, and national estimates of levels of preterm birth in 2014: a systematic review and modelling analysis. Lancet Glob Heal. 2019;7(1):e37-e46.
[3] United National Population Division World Population Prospects 2019.
[4] Fink G, Peet E, Danaei G, et al. Schooling and wage income losses due to early-childhood growth faltering in developing countries: National, regional, and global estimates. Am J Clin Nutr. 2016;104(1):104-112.
[5] Country specific annual wage data from World Indicators Database. Average yearly wage was estimated to be 2/3 of the gross domestic product in 2010 constant US dollars and 2011 International dollars, adjusted for purchasing power parity.
[6] NCD Risk Factor Collaboration. Trends in adult body-mass index in 200 countries from 1975 to 2014: a pooled analysis of 1698 population-based measurement studies with 19·2 million participants. Lancet. 2016;387(10026):1377-1396.
[7] Stevens GA, Finucane MM, De-Regil LM, et al. Global, regional, and national trends in haemoglobin concentration and prevalence of total and severe anaemia in children and pregnant and non-pregnant women for 1995-2011: A systematic analysis of population-representative data. Lancet Glob Heal. 2013;1(1):16-25.
[8] Coverage of iron-folic acid supplementation abstracted from the most recent Demographic Health Survey or imputed based on sub-regional average. Indicator used: % women in the past five years who took iron tablets or syrup for >90 days.

Bhutan

Region: South Asia**; Sub-region:** South Asia

Low birthweight prevalence[1]: 11.7% (95% CI: 8.2, 18.5)

Preterm birth prevalence[2]: 10.4% (95% CI: 8.7, 11.9)

Number of births[3]: 66,000

Returns to education[4]: 7.8% (95% CI: 7.0, 8.6)

GDP per capita 2010 US$ (estimated annual wage)[5]: $2844 ($1896/year)

GDP per capita 2011 International $ (estimated annual wage)[5]: $8380 ($5587/year)

Prevalence of low BMI[6]: 10.7% (95% CI: 5.0, 18.2)

Prevalence of anemia[7]: 46.4% (95% CI: 26.5, 66.6)

Baseline coverage of IFA[8]: 39.4%

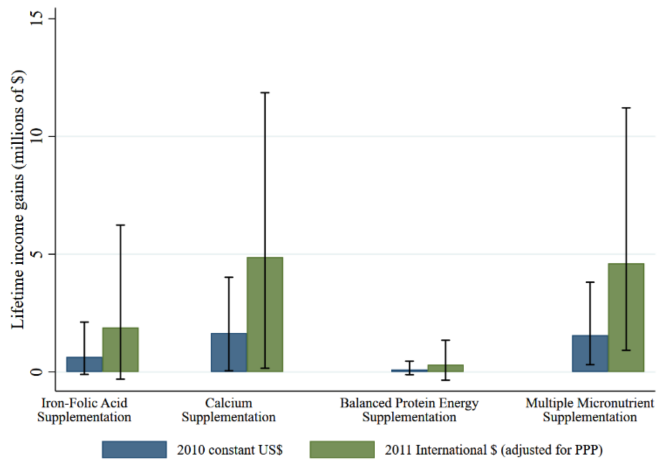

Figure: Benefits by birth cohort size for estimated gains in lifetime wages attributable to scaling up nutrition interventions to 90% target coverage

Table: Impact of maternal prenatal nutrition intervention on human capital and labour market outcomes, through improvements in low birthweight or preterm birth and schooling in Bhutan.

Intervention	Target Coverage (%)	Absolute reduction in birth outcome (%)	Benefits by cohorts: School years gained (in 1000 s)	No. of additional students completing secondary school	Benefits by cohorts: Lifetime wages		Returns in lifetime earnings per child born to a targeted pregnant woman	
					in US $ millions	in International $ millions	in US $	in International $
**Iron/Folic Acid Suppl.**	90%	0.99 (-0.18, 2.35)	0.16 (-0.03, 0.53)	6 (-1, 19)	0.64 (-0.10, 2.12)	1.89 (-0.31, 6.24)	10.80 (-1.76, 35.63)	31.81 (-5.18, 104.98)
**Calcium Suppl.**	50%	1.24 (0.29, 2.14)	0.23 (0.01, 0.55)	8 (0, 20)	0.92 (0.03, 2.24)	2.71 (0.09, 6.59)	27.86 (0.90, 67.76)	82.10 (2.65, 199.65)
	90%	2.22 (0.51, 3.85)	0.41 (0.01, 0.99)	15 (0, 35)	1.66 (0.05, 4.03)	4.88 (0.16, 11.86)	27.86 (0.90, 67.76)	82.10 (2.65, 199.65)
**Multiple Micronutrient Suppl.**	50%	0.91 (0.46, 1.48)	0.16 (0.03, 0.38)	6 (1, 14)	0.65 (0.13, 1.53)	1.91 (0.37, 4.52)	19.63 (3.79, 46.50)	57.84 (11.17, 137.01)
	90%	2.25 (1.01, 4.03)	0.39 (0.08, 0.97)	14 (3, 35)	1.57 (0.31, 3.81)	4.61 (0.92, 11.22)	26.36 (5.25, 64.10)	77.66 (15.48, 188.84)
**Balanced Protein Suppl.**	50%	0.09 (-0.08, 0.31)	0.01 (-0.02, 0.06)	1 (-1, 2)	0.06 (-0.07, 0.25)	0.17 (-0.19, 0.75)	18.32 (-16.73, 62.69)	53.98 (-49.29, 184.70)
	90%	0.17 (-0.14, 0.56)	0.03 (-0.03, 0.12)	1 (-1, 4)	0.10 (-0.12, 0.46)	0.31 (-0.35, 1.35)	18.32 (-16.73, 62.69)	53.98 (-49.29, 184.70)

References for Data Inputs

[1] Blencowe H, Krasevec J, Onis M De, et al. Articles National , regional , and worldwide estimates of low birthweight in 2015 , with trends from 2000: a systematic analysis. Lancet Glob Heal. 2019;(18):1-12.
[2] Chawanpaiboon S, Vogel JP, Moller AB, et al. Global, regional, and national estimates of levels of preterm birth in 2014: a systematic review and modelling analysis. Lancet Glob Heal. 2019;7(1):e37-e46.
[3] United National Population Division World Population Prospects 2019.
[4] Fink G, Peet E, Danaei G, et al. Schooling and wage income losses due to early-childhood growth faltering in developing countries: National, regional, and global estimates. Am J Clin Nutr. 2016;104(1):104-112.
[5] Country specific annual wage data from World Indicators Database. Average yearly wage was estimated to be 2/3 of the gross domestic product in 2010 constant US dollars and 2011 International dollars, adjusted for purchasing power parity.
[6] NCD Risk Factor Collaboration. Trends in adult body-mass index in 200 countries from 1975 to 2014: a pooled analysis of 1698 population-based measurement studies with 19·2 million participants. Lancet. 2016;387(10026):1377-1396.
[7] Stevens GA, Finucane MM, De-Regil LM, et al. Global, regional, and national trends in haemoglobin concentration and prevalence of total and severe anaemia in children and pregnant and non-pregnant women for 1995-2011: A systematic analysis of population-representative data. Lancet Glob Heal. 2013;1(1):16-25.
[8] Coverage of iron-folic acid supplementation abstracted from the most recent Demographic Health Survey or imputed based on sub-regional average. Indicator used: % women in the past five years who took iron tablets or syrup for >90 days.

Bolivia

Region: Latin America and Caribbean**; Sub-region:** Andean Latin America

Low birthweight prevalence[1]: 7.2% (95% CI: 5.7, 9.3)

Preterm birth prevalence[2]: 9.8% (95% CI: 8.6, 11.3)

Number of births[3]: 1,233,000

Returns to education[4]: 6.7% (95% CI: 5.5, 8.0)

GDP per capita 2010 US$ (estimated annual wage)[5]: $2361 ($1574/year)

GDP per capita 2011 International $ (estimated annual wage)[5]: $6444 ($4296/year)

Prevalence of low BMI[6]: 1.3% (95% CI: 0.4, 2.9)

Prevalence of anemia[7]: 37.4% (95% CI: 25.2, 51.0)

Baseline coverage of IFA[8]: 44.7%

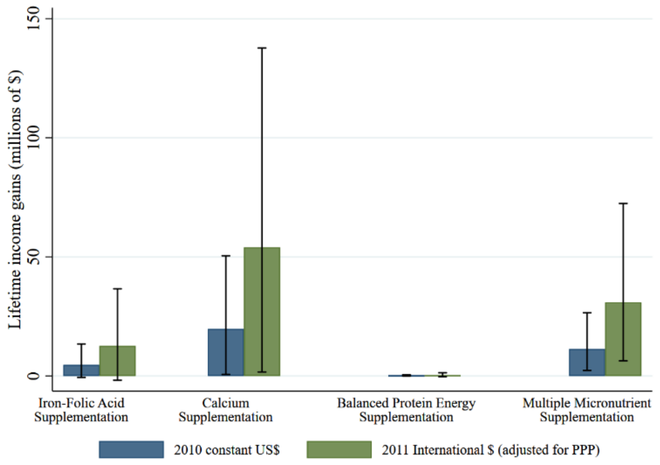

Figure: Benefits by birth cohort size for estimated gains in lifetime wages attributable to scaling up nutrition interventions to 90% target coverage

Table: Impact of maternal prenatal nutrition intervention on human capital and labour market outcomes, through improvements in low birthweight or preterm birth and schooling in Bolivia.

Intervention	Target Coverage (%)	Absolute reduction in birth outcome (%)	Benefits by cohorts: School years gained (in 1000 s)	No. of additional students completing secondary school	Benefits by cohorts: Lifetime wages		Returns in lifetime earnings per child born to a targeted pregnant woman	
					in US $ millions	in International $ millions	in US $	in International $
Iron/Folic Acid Suppl.	90%	0.55 (-0.09, 1.23)	1.62 (-0.22, 4.54)	74 (-10, 206)	4.64 (-0.65, 13.40)	12.67 (-1.76, 36.58)	4.18 (-0.58, 12.08)	11.42 (-1.59, 32.97)
Calcium Suppl.	50%	1.16 (0.27, 1.99)	3.91 (0.11, 9.07)	177 (5, 412)	10.99 (0.34, 28.03)	30.00 (0.93, 76.51)	17.83 (0.55, 45.47)	48.67 (1.50, 124.10)
	90%	2.09 (0.48, 3.58)	7.03 (0.20, 16.32)	319 (9, 741)	19.79 (0.61, 50.45)	54.01 (1.67, 137.71)	17.83 (0.55, 45.47)	48.67 (1.50, 124.10)
Multiple Micronutrient Suppl.	50%	0.47 (0.28, 0.69)	1.49 (0.30, 3.39)	68 (14, 154)	4.31 (0.85, 9.63)	11.76 (2.31, 26.29)	6.99 (1.38, 15.62)	19.07 (3.75, 42.64)
	90%	1.27 (0.61, 1.99)	3.99 (0.84, 9.25)	181 (38, 420)	11.33 (2.32, 26.54)	30.92 (6.34, 72.45)	10.21 (2.09, 23.92)	27.87 (5.72, 65.29)
Balanced Protein Suppl.	50%	0.01 (-0.01, 0.02)	0.02 (-0.02, 0.09)	1 (-1, 4)	0.05 (-0.06, 0.27)	0.14 (-0.16, 0.74)	7.66 (-7.64, 25.18)	20.91 (-20.85, 68.72)
	90%	0.01 (-0.01, 0.04)	0.03 (-0.04, 0.16)	1 (-2, 7)	0.09 (-0.11, 0.49)	0.25 (-0.29, 1.33)	7.66 (-7.64, 25.18)	20.91 (-20.85, 68.72)

References for Data Inputs

[1] Blencowe H, Krasevec J, Onis M De, et al. Articles National , regional , and worldwide estimates of low birthweight in 2015 , with trends from 2000: a systematic analysis. Lancet Glob Heal. 2019;(18):1-12.

[2] Chawanpaiboon S, Vogel JP, Moller AB, et al. Global, regional, and national estimates of levels of preterm birth in 2014: a systematic review and modelling analysis. Lancet Glob Heal. 2019;7(1):e37-e46.

[3] United National Population Division World Population Prospects 2019.

[4] Fink G, Peet E, Danaei G, et al. Schooling and wage income losses due to early-childhood growth faltering in developing countries: National, regional, and global estimates. Am J Clin Nutr. 2016;104(1):104-112.

[5] Country specific annual wage data from World Indicators Database. Average yearly wage was estimated to be 2/3 of the gross domestic product in 2010 constant US dollars and 2011 International dollars, adjusted for purchasing power parity.

[6] NCD Risk Factor Collaboration. Trends in adult body-mass index in 200 countries from 1975 to 2014: a pooled analysis of 1698 population-based measurement studies with 19·2 million participants. Lancet. 2016;387(10026):1377-1396.

[7] Stevens GA, Finucane MM, De-Regil LM, et al. Global, regional, and national trends in haemoglobin concentration and prevalence of total and severe anaemia in children and pregnant and non-pregnant women for 1995-2011: A systematic analysis of population-representative data. Lancet Glob Heal. 2013;1(1):16-25.

[8] Coverage of iron-folic acid supplementation abstracted from the most recent Demographic Health Survey or imputed based on sub-regional average. Indicator used: % women in the past five years who took iron tablets or syrup for >90 days.

Bosnia

Region: Central Europe, Eastern Europe, Central Asia**; Sub-region:** Central Europe

Low birthweight prevalence[1]: 3.4% (95% CI: 2.7, 4.2)

Preterm birth prevalence[2]: 6.7% (95% CI: 2.2, 15.6)

Number of births[3]: 137,000

Returns to education[4]: 8.2% (95% CI: 7.6, 8.8)

GDP per capita 2010 US$ (estimated annual wage)[5]: $5352 ($3568/year)

GDP per capita 2011 International $ (estimated annual wage)[5]: $11274 ($7516/year)

Prevalence of low BMI[6]: 2.4% (95% CI: 1.0, 4.4)

Prevalence of anemia[7]: 26.8% (95% CI: 12.2, 49.9)

Baseline coverage of IFA[8]: 18.5%

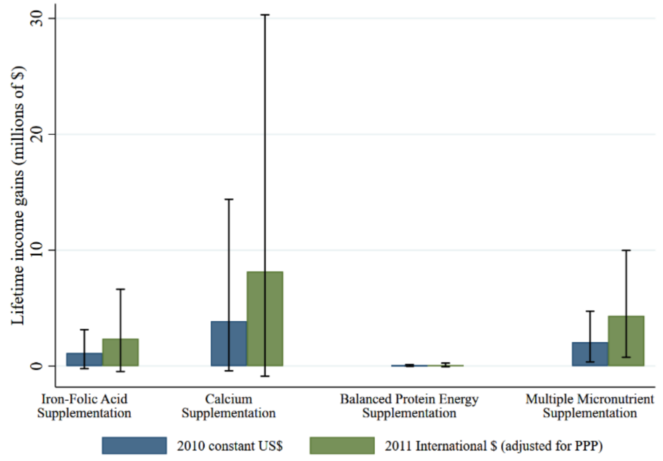

Figure: Benefits by birth cohort size for estimated gains in lifetime wages attributable to scaling up nutrition interventions to 90% target coverage

Table: Impact of maternal prenatal nutrition intervention on human capital and labour market outcomes, through improvements in low birthweight or preterm birth and schooling in Bosnia.

Intervention	Target Coverage (%)	Absolute reduction in birth outcome (%)	Benefits by cohorts: School years gained (in 1000 s)	No. of additional students completing secondary school	Benefits by cohorts: Lifetime wages		Returns in lifetime earnings per child born to a targeted pregnant woman	
					in US $ millions	in International $ millions	in US $	in International $
Iron/Folic Acid Suppl.	90%	0.40 (-0.08, 0.83)	0.14 (-0.03, 0.39)	6 (-1, 17)	1.12 (-0.22, 3.14)	2.37 (-0.47, 6.62)	9.12 (-1.80, 25.51)	19.20 (-3.80, 53.72)
Calcium Suppl.	50%	0.69 (-0.03, 2.07)	0.27 (-0.03, 1.02)	12 (-1, 45)	2.15 (-0.23, 8.00)	4.53 (-0.48, 16.84)	31.38 (-3.36, 116.75)	66.09 (-7.07, 245.91)
	90%	1.25 (-0.06, 3.73)	0.49 (-0.05, 1.83)	22 (-2, 81)	3.87 (-0.41, 14.40)	8.15 (-0.87, 30.32)	31.38 (-3.36, 116.75)	66.09 (-7.07, 245.91)
Multiple Micronutrient Suppl.	50%	0.35 (0.15, 0.55)	0.13 (0.02, 0.29)	6 (1, 13)	1.02 (0.20, 2.30)	2.15 (0.41, 4.85)	14.92 (2.86, 33.62)	31.43 (6.02, 70.81)
	90%	0.70 (0.27, 1.13)	0.26 (0.05, 0.61)	11 (2, 27)	2.06 (0.36, 4.74)	4.33 (0.76, 9.98)	16.67 (2.91, 38.42)	35.12 (6.13, 80.93)
Balanced Protein Suppl.	50%	0.01 (-0.01, 0.02)	0.00 (-0.00, 0.01)	0 (-0, 0)	0.02 (-0.02, 0.07)	0.03 (-0.03, 0.14)	11.26 (-10.90, 34.88)	23.71 (-22.96, 73.46)
	90%	0.01 (-0.01, 0.03)	0.00 (-0.00, 0.02)	0 (-0, 1)	0.03 (-0.03, 0.12)	0.06 (-0.06, 0.25)	11.26 (-10.90, 34.88)	23.71 (-22.96, 73.46)

References for Data Inputs

[1] Blencowe H, Krasevec J, Onis M De, et al. Articles National , regional , and worldwide estimates of low birthweight in 2015 , with trends from 2000: a systematic analysis. Lancet Glob Heal. 2019;(18):1-12.

[2] Chawanpaiboon S, Vogel JP, Moller AB, et al. Global, regional, and national estimates of levels of preterm birth in 2014: a systematic review and modelling analysis. Lancet Glob Heal. 2019;7(1):e37-e46.

[3] United National Population Division World Population Prospects 2019.

[4] Fink G, Peet E, Danaei G, et al. Schooling and wage income losses due to early-childhood growth faltering in developing countries: National, regional, and global estimates. Am J Clin Nutr. 2016;104(1):104-112.

[5] Country specific annual wage data from World Indicators Database. Average yearly wage was estimated to be 2/3 of the gross domestic product in 2010 constant US dollars and 2011 International dollars, adjusted for purchasing power parity.

[6] NCD Risk Factor Collaboration. Trends in adult body-mass index in 200 countries from 1975 to 2014: a pooled analysis of 1698 population-based measurement studies with 19·2 million participants. Lancet. 2016;387(10026):1377-1396.

[7] Stevens GA, Finucane MM, De-Regil LM, et al. Global, regional, and national trends in haemoglobin concentration and prevalence of total and severe anaemia in children and pregnant and non-pregnant women for 1995-2011: A systematic analysis of population-representative data. Lancet Glob Heal. 2013;1(1):16-25.

[8] Coverage of iron-folic acid supplementation abstracted from the most recent Demographic Health Survey or imputed based on sub-regional average. Indicator used: % women in the past five years who took iron tablets or syrup for >90 days.

Botswana

Region: Sub-Saharan Africa**; Sub-region:** Southern Sub-Saharan Africa

Low birthweight prevalence[1]: 15.6% (95% CI: 12.3, 20.6)

Preterm birth prevalence[2]: 12.0% (95% CI: 8.6, 16.7)

Number of births[3]: 281,000

Returns to education[4]: 16.0% (95% CI: 15.2, 16.8)

GDP per capita 2010 US$ (estimated annual wage)[5]: $7614 ($5076/year)

GDP per capita 2011 International $ (estimated annual wage)[5]: $15660 ($10440/year)

Prevalence of low BMI[6]: 6.3% (95% CI: 2.8, 11.4)

Prevalence of anemia[7]: 32.0% (95% CI: 14.5, 55.3)

Baseline coverage of IFA[8]: 45.0%

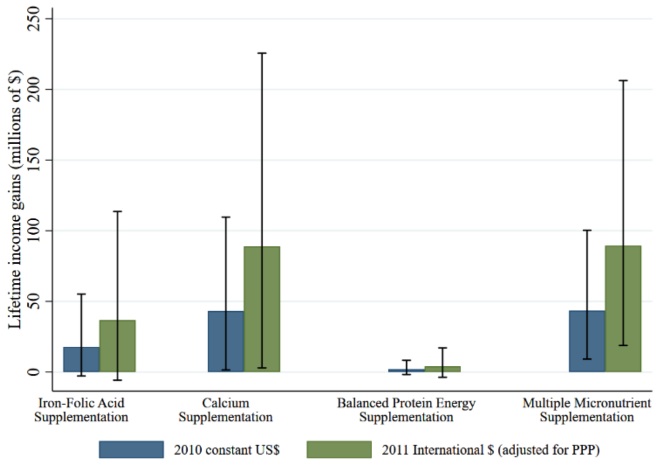

Figure: Benefits by birth cohort size for estimated gains in lifetime wages attributable to scaling up nutrition interventions to 90% target coverage

Table: Impact of maternal prenatal nutrition intervention on human capital and labour market outcomes, through improvements in low birthweight or preterm birth and schooling in Botswana.

Intervention	Target Coverage (%)	Absolute reduction in birth outcome (%)	Benefits by cohorts: School years gained (in 1000 s)	No. of additional students completing secondary school	Benefits by cohorts: Lifetime wages		Returns in lifetime earnings per child born to a targeted pregnant woman	
					in US $ millions	in International $ millions	in US $	in International $
Iron/Folic Acid Suppl.	90%	1.21 (-0.19, 2.65)	0.82 (-0.13, 2.50)	98 (-16, 298)	17.86 (-2.79, 55.20)	36.73 (-5.73, 113.54)	70.62 (-11.02, 218.29)	145.24 (-22.68, 448.97)
Calcium Suppl.	50%	1.42 (0.31, 2.62)	1.09 (0.04, 2.75)	130 (4, 328)	24.00 (0.81, 60.93)	49.35 (1.66, 125.32)	170.79 (5.76, 433.66)	351.28 (11.84, 891.96)
	90%	2.55 (0.57, 4.71)	1.97 (0.07, 4.95)	235 (8, 590)	43.19 (1.46, 109.67)	88.84 (2.99, 225.58)	170.79 (5.76, 433.66)	351.28 (11.84, 891.96)
Multiple Micronutrient Suppl.	50%	0.98 (0.54, 1.49)	0.72 (0.16, 1.64)	86 (19, 195)	15.93 (3.46, 36.72)	32.77 (7.12, 75.53)	113.39 (24.65, 261.37)	233.22 (50.71, 537.59)
	90%	2.73 (1.28, 4.34)	1.99 (0.42, 4.58)	237 (49, 545)	43.49 (9.13, 100.29)	89.46 (18.77, 206.27)	171.98 (36.09, 396.55)	353.74 (74.22, 815.63)
Balanced Protein Suppl.	50%	0.08 (-0.06, 0.23)	0.05 (-0.05, 0.21)	6 (-6, 25)	1.09 (-1.00, 4.63)	2.23 (-2.05, 9.53)	138.39 (-126.93, 445.65)	284.63 (-261.07, 916.62)
	90%	0.14 (-0.11, 0.42)	0.09 (-0.08, 0.38)	11 (-10, 45)	1.95 (-1.80, 8.34)	4.02 (-3.69, 17.15)	138.39 (-126.93, 445.65)	284.63 (-261.07, 916.62)

References for Data Inputs

[1] Blencowe H, Krasevec J, Onis M De, et al. Articles National , regional , and worldwide estimates of low birthweight in 2015 , with trends from 2000: a systematic analysis. Lancet Glob Heal. 2019;(18):1-12.
[2] Chawanpaiboon S, Vogel JP, Moller AB, et al. Global, regional, and national estimates of levels of preterm birth in 2014: a systematic review and modelling analysis. Lancet Glob Heal. 2019;7(1):e37-e46.
[3] United National Population Division World Population Prospects 2019.
[4] Fink G, Peet E, Danaei G, et al. Schooling and wage income losses due to early-childhood growth faltering in developing countries: National, regional, and global estimates. Am J Clin Nutr. 2016;104(1):104-112.
[5] Country specific annual wage data from World Indicators Database. Average yearly wage was estimated to be 2/3 of the gross domestic product in 2010 constant US dollars and 2011 International dollars, adjusted for purchasing power parity.
[6] NCD Risk Factor Collaboration. Trends in adult body-mass index in 200 countries from 1975 to 2014: a pooled analysis of 1698 population-based measurement studies with 19·2 million participants. Lancet. 2016;387(10026):1377-1396.
[7] Stevens GA, Finucane MM, De-Regil LM, et al. Global, regional, and national trends in haemoglobin concentration and prevalence of total and severe anaemia in children and pregnant and non-pregnant women for 1995-2011: A systematic analysis of population-representative data. Lancet Glob Heal. 2013;1(1):16-25.
[8] Coverage of iron-folic acid supplementation abstracted from the most recent Demographic Health Survey or imputed based on sub-regional average. Indicator used: % women in the past five years who took iron tablets or syrup for >90 days.

Brazil

Region: Latin America and Caribbean**; Sub-region:** Tropical Latin America

Low birthweight prevalence[1]: 8.4% (95% CI: 8.3, 8.5)

Preterm birth prevalence[2]: 11.2% (95% CI: 9.4, 12.9)

Number of births[3]: 14,672,000

Returns to education[4]: 13.3% (95% CI: 11.6, 15.0)

GDP per capita 2010 US$ (estimated annual wage)[5]: $11431 ($7621/year)

GDP per capita 2011 International $ (estimated annual wage)[5]: $14807 ($9871/year)

Prevalence of low BMI[6]: 3.2% (95% CI: 1.9, 4.9)

Prevalence of anemia[7]: 32.4% (95% CI: 12.0, 61.1)

Baseline coverage of IFA[8]: 34.2%

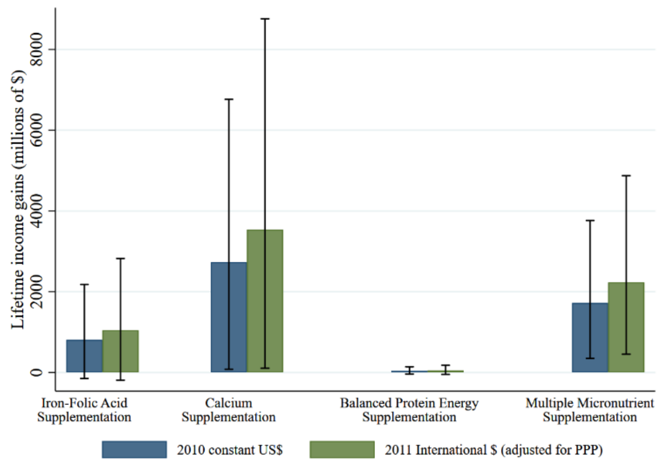

Figure: Benefits by birth cohort size for estimated gains in lifetime wages attributable to scaling up nutrition interventions to 90% target coverage

Table: Impact of maternal prenatal nutrition intervention on human capital and labour market outcomes, through improvements in low birthweight or preterm birth and schooling in Brazil.

Intervention	Target Coverage (%)	Absolute reduction in birth outcome (%)	Benefits by cohorts: School years gained (in 1000 s)	No. of additional students completing secondary school	Benefits by cohorts: Lifetime wages		Returns in lifetime earnings per child born to a targeted pregnant woman	
					in US $ millions	in International $ millions	in US $	in International $
Iron/Folic Acid Suppl.	90%	0.80 (-0.14, 1.62)	29.54 (-5.21, 80.30)	2104 (-371, 5717)	809.16 (-147.07, 2180.43)	1048.13 (-190.50, 2824.40)	61.28 (-11.14, 165.12)	79.38 (-14.43, 213.89)
Calcium Suppl.	50%	1.33 (0.28, 2.28)	55.35 (1.66, 136.44)	3941 (118, 9714)	1516.97 (44.62, 3757.56)	1964.99 (57.79, 4867.31)	206.78 (6.08, 512.21)	267.86 (7.88, 663.48)
	90%	2.40 (0.51, 4.10)	99.64 (2.98, 245.58)	7094 (212, 17486)	2730.54 (80.31, 6763.60)	3536.98 (104.03, 8761.16)	206.78 (6.08, 512.21)	267.86 (7.88, 663.48)
Multiple Micronutrient Suppl.	50%	0.68 (0.37, 0.95)	26.76 (6.30, 55.59)	1906 (448, 3958)	730.20 (161.67, 1560.95)	945.86 (209.41, 2021.95)	99.54 (22.04, 212.78)	128.93 (28.55, 275.62)
	90%	1.62 (0.72, 2.43)	61.90 (13.06, 136.67)	4407 (930, 9731)	1722.58 (349.86, 3763.19)	2231.33 (453.19, 4874.61)	130.45 (26.50, 284.99)	168.98 (34.32, 369.15)
Balanced Protein Suppl.	50%	0.02 (-0.02, 0.06)	0.75 (-0.78, 2.87)	53 (-56, 204)	20.73 (-21.29, 76.28)	26.85 (-27.57, 98.81)	94.96 (-92.31, 280.12)	123.00 (-119.58, 362.85)
	90%	0.04 (-0.03, 0.11)	1.35 (-1.41, 5.16)	96 (-100, 368)	37.31 (-38.31, 137.30)	48.33 (-49.63, 177.85)	94.96 (-92.31, 280.12)	123.00 (-119.58, 362.85)

References for Data Inputs
[1] Blencowe H, Krasevec J, Onis M De, et al. Articles National , regional , and worldwide estimates of low birthweight in 2015 , with trends from 2000: a systematic analysis. Lancet Glob Heal. 2019;(18):1-12.
[2] Chawanpaiboon S, Vogel JP, Moller AB, et al. Global, regional, and national estimates of levels of preterm birth in 2014: a systematic review and modelling analysis. Lancet Glob Heal. 2019;7(1):e37-e46.
[3] United National Population Division World Population Prospects 2019.
[4] Fink G, Peet E, Danaei G, et al. Schooling and wage income losses due to early-childhood growth faltering in developing countries: National, regional, and global estimates. Am J Clin Nutr. 2016;104(1):104-112.
[5] Country specific annual wage data from World Indicators Database. Average yearly wage was estimated to be 2/3 of the gross domestic product in 2010 constant US dollars and 2011 International dollars, adjusted for purchasing power parity.
[6] NCD Risk Factor Collaboration. Trends in adult body-mass index in 200 countries from 1975 to 2014: a pooled analysis of 1698 population-based measurement studies with 19·2 million participants. Lancet. 2016;387(10026):1377-1396.
[7] Stevens GA, Finucane MM, De-Regil LM, et al. Global, regional, and national trends in haemoglobin concentration and prevalence of total and severe anaemia in children and pregnant and non-pregnant women for 1995-2011: A systematic analysis of population-representative data. Lancet Glob Heal. 2013;1(1):16-25.
[8] Coverage of iron-folic acid supplementation abstracted from the most recent Demographic Health Survey or imputed based on sub-regional average. Indicator used: % women in the past five years who took iron tablets or syrup for >90 days.

Bulgaria

Region: Central Europe, Eastern Europe, Central Asia**; Sub-region:** Central Europe

Low birthweight prevalence[1]: 9.6% (95% CI: 9.1, 10.0)

Preterm birth prevalence[2]: 9.0% (95% CI: 6.8, 11.6)

Number of births[3]: 317,000

Returns to education[4]: 4.6% (95% CI: 4.2, 5.0)

GDP per capita 2010 US$ (estimated annual wage)[5]: $7612 ($5074/year)

GDP per capita 2011 International $ (estimated annual wage)[5]: $16999 ($11333/year)

Prevalence of low BMI[6]: 1.8% (95% CI: 0.6, 3.9)

Prevalence of anemia[7]: 25.9% (95% CI: 12.7, 48.3)

Baseline coverage of IFA[8]: 18.5%

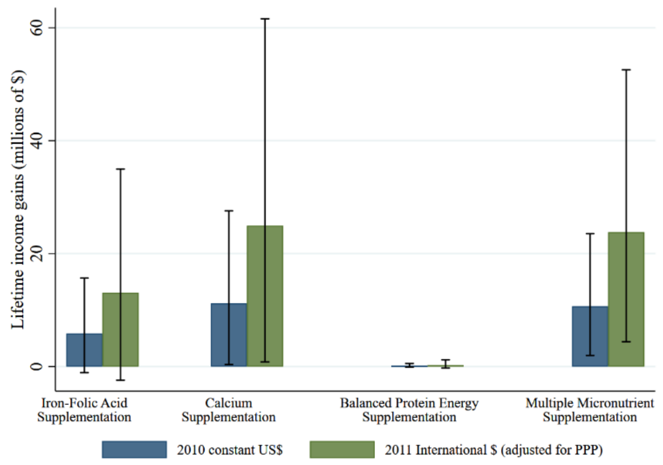

Figure: Benefits by birth cohort size for estimated gains in lifetime wages attributable to scaling up nutrition interventions to 90% target coverage

Table: Impact of maternal prenatal nutrition intervention on human capital and labour market outcomes, through improvements in low birthweight or preterm birth and schooling in Bulgaria.

Intervention	Target Coverage (%)	Absolute reduction in birth outcome (%)	Benefits by cohorts: School years gained (in 1000 s)	No. of additional students completing secondary school	Benefits by cohorts: Lifetime wages		Returns in lifetime earnings per child born to a targeted pregnant woman	
					in US $ millions	in International $ millions	in US $	in International $
Iron/Folic Acid Suppl.	90%	1.14 (-0.22, 2.26)	0.93 (-0.17, 2.45)	23 (-4, 62)	5.85 (-1.08, 15.66)	13.06 (-2.41, 34.98)	20.50 (-3.79, 54.90)	45.78 (-8.46, 122.60)
Calcium Suppl.	50%	1.06 (0.23, 1.89)	0.97 (0.03, 2.48)	25 (1, 62)	6.21 (0.20, 15.32)	13.86 (0.45, 34.22)	39.16 (1.28, 96.68)	87.47 (2.85, 215.93)
	90%	1.91 (0.42, 3.40)	1.75 (0.06, 4.46)	44 (1, 112)	11.17 (0.36, 27.58)	24.95 (0.81, 61.60)	39.16 (1.28, 96.68)	87.47 (2.85, 215.93)
Multiple Micronutrient Suppl.	50%	0.97 (0.42, 1.44)	0.83 (0.17, 1.78)	21 (4, 45)	5.24 (1.04, 11.46)	11.70 (2.32, 25.60)	33.06 (6.56, 72.31)	73.84 (14.66, 161.49)
	90%	2.00 (0.78, 3.01)	1.69 (0.30, 3.72)	43 (7, 94)	10.66 (1.97, 23.53)	23.80 (4.40, 52.56)	37.36 (6.90, 82.49)	83.43 (15.41, 184.23)
Balanced Protein Suppl.	50%	0.01 (-0.01, 0.04)	0.01 (-0.01, 0.05)	0 (-0, 1)	0.06 (-0.07, 0.30)	0.14 (-0.15, 0.66)	25.69 (-23.90, 79.37)	57.37 (-53.38, 177.25)
	90%	0.02 (-0.02, 0.08)	0.02 (-0.02, 0.09)	0 (-0, 2)	0.11 (-0.12, 0.54)	0.25 (-0.26, 1.20)	25.69 (-23.90, 79.37)	57.37 (-53.38, 177.25)

References for Data Inputs

[1] Blencowe H, Krasevec J, Onis M De, et al. Articles National , regional , and worldwide estimates of low birthweight in 2015 , with trends from 2000: a systematic analysis. Lancet Glob Heal. 2019;(18):1-12.
[2] Chawanpaiboon S, Vogel JP, Moller AB, et al. Global, regional, and national estimates of levels of preterm birth in 2014: a systematic review and modelling analysis. Lancet Glob Heal. 2019;7(1):e37-e46.
[3] United National Population Division World Population Prospects 2019.
[4] Fink G, Peet E, Danaei G, et al. Schooling and wage income losses due to early-childhood growth faltering in developing countries: National, regional, and global estimates. Am J Clin Nutr. 2016;104(1):104-112.
[5] Country specific annual wage data from World Indicators Database. Average yearly wage was estimated to be 2/3 of the gross domestic product in 2010 constant US dollars and 2011 International dollars, adjusted for purchasing power parity.
[6] NCD Risk Factor Collaboration. Trends in adult body-mass index in 200 countries from 1975 to 2014: a pooled analysis of 1698 population-based measurement studies with 19·2 million participants. Lancet. 2016;387(10026):1377-1396.
[7] Stevens GA, Finucane MM, De-Regil LM, et al. Global, regional, and national trends in haemoglobin concentration and prevalence of total and severe anaemia in children and pregnant and non-pregnant women for 1995-2011: A systematic analysis of population-representative data. Lancet Glob Heal. 2013;1(1):16-25.
[8] Coverage of iron-folic acid supplementation abstracted from the most recent Demographic Health Survey or imputed based on sub-regional average. Indicator used: % women in the past five years who took iron tablets or syrup for >90 days.

Burkina Faso

Region: Sub-Saharan Africa**; Sub-region:** Western Sub-Saharan Africa

Low birthweight prevalence[1]: 13.1% (95% CI: 9.7, 16.3)

Preterm birth prevalence[2]: 12.0% (95% CI: 8.6, 16.7)

Number of births[3]: 3,725,000

Returns to education[4]: 6.3% (95% CI: 4.5, 8.1)

GDP per capita 2010 US$ (estimated annual wage)[5]: $645 ($430/year)

GDP per capita 2011 International $ (estimated annual wage)[5]: $1596 ($1064/year)

Prevalence of low BMI[6]: 12.7% (95% CI: 7.3, 19.3)

Prevalence of anemia[7]: 58.1% (95% CI: 50.1, 64.2)

Baseline coverage of IFA[8]: 50.2%

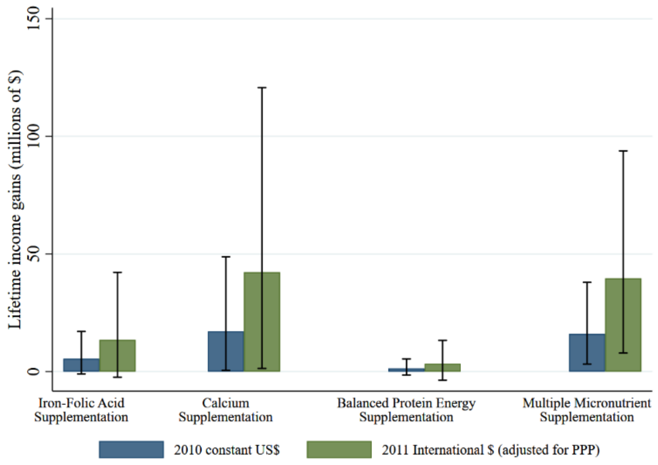

Figure: Benefits by birth cohort size for estimated gains in lifetime wages attributable to scaling up nutrition interventions to 90% target coverage

Table: Impact of maternal prenatal nutrition intervention on human capital and labour market outcomes, through improvements in low birthweight or preterm birth and schooling in Burkina Faso.

Intervention	Target Coverage (%)	Absolute reduction in birth outcome (%)	Benefits by cohorts: School years gained (in 1000 s)	No. of additional students completing secondary school	Benefits by cohorts: Lifetime wages		Returns in lifetime earnings per child born to a targeted pregnant woman	
					in US $ millions	in International $ millions	in US $	in International $
**Iron/Folic Acid Suppl.**	90%	0.88 (-0.15, 1.95)	7.55 (-1.16, 22.39)	246 (-38, 731)	5.46 (-0.96, 17.06)	13.51 (-2.38, 42.21)	1.63 (-0.29, 5.09)	4.03 (-0.71, 12.59)
**Calcium Suppl.**	50%	1.41 (0.27, 2.56)	13.25 (0.44, 34.59)	433 (14, 1130)	9.49 (0.30, 27.11)	23.48 (0.74, 67.06)	5.10 (0.16, 14.56)	12.61 (0.40, 36.01)
	90%	2.55 (0.48, 4.61)	23.85 (0.79, 62.26)	779 (26, 2033)	17.08 (0.54, 48.80)	42.26 (1.33, 120.71)	5.10 (0.16, 14.56)	12.61 (0.40, 36.01)
**Multiple Micronutrient Suppl.**	50%	0.95 (0.53, 1.40)	8.75 (1.60, 19.69)	286 (52, 643)	6.31 (1.15, 14.99)	15.61 (2.84, 37.09)	3.39 (0.62, 8.05)	8.38 (1.52, 19.91)
	90%	2.46 (1.35, 3.75)	21.97 (4.20, 48.92)	718 (137, 1598)	16.02 (3.19, 37.94)	39.63 (7.88, 93.83)	4.78 (0.95, 11.32)	11.82 (2.35, 27.99)
**Balanced Protein Suppl.**	50%	0.13 (-0.11, 0.36)	1.04 (-1.06, 3.83)	34 (-35, 125)	0.75 (-0.82, 2.98)	1.87 (-2.02, 7.38)	3.36 (-3.10, 11.65)	8.30 (-7.66, 28.81)
	90%	0.24 (-0.19, 0.64)	1.88 (-1.92, 6.90)	61 (-63, 225)	1.36 (-1.47, 5.37)	3.36 (-3.63, 13.29)	3.36 (-3.10, 11.65)	8.30 (-7.66, 28.81)

References for Data Inputs

[1] Blencowe H, Krasevec J, Onis M De, et al. Articles National , regional , and worldwide estimates of low birthweight in 2015 , with trends from 2000: a systematic analysis. Lancet Glob Heal. 2019;(18):1-12.
[2] Chawanpaiboon S, Vogel JP, Moller AB, et al. Global, regional, and national estimates of levels of preterm birth in 2014: a systematic review and modelling analysis. Lancet Glob Heal. 2019;7(1):e37-e46.
[3] United National Population Division World Population Prospects 2019.
[4] Fink G, Peet E, Danaei G, et al. Schooling and wage income losses due to early-childhood growth faltering in developing countries: National, regional, and global estimates. Am J Clin Nutr. 2016;104(1):104-112.
[5] Country specific annual wage data from World Indicators Database. Average yearly wage was estimated to be 2/3 of the gross domestic product in 2010 constant US dollars and 2011 International dollars, adjusted for purchasing power parity.
[6] NCD Risk Factor Collaboration. Trends in adult body-mass index in 200 countries from 1975 to 2014: a pooled analysis of 1698 population-based measurement studies with 19·2 million participants. Lancet. 2016;387(10026):1377-1396.
[7] Stevens GA, Finucane MM, De-Regil LM, et al. Global, regional, and national trends in haemoglobin concentration and prevalence of total and severe anaemia in children and pregnant and non-pregnant women for 1995-2011: A systematic analysis of population-representative data. Lancet Glob Heal. 2013;1(1):16-25.
[8] Coverage of iron-folic acid supplementation abstracted from the most recent Demographic Health Survey or imputed based on sub-regional average. Indicator used: % women in the past five years who took iron tablets or syrup for >90 days.

Burundi

Region: Sub-Saharan Africa**; Sub-region:** Eastern Sub-Saharan Africa

Low birthweight prevalence[1]: 15.1% (95% CI: 10.9, 19.4)

Preterm birth prevalence[2]: 12.0% (95% CI: 8.6, 16.7)

Number of births[3]: 2,164,000

Returns to education[4]: 11.3% (95% CI: 9.7, 12.9)

GDP per capita 2010 US$ (estimated annual wage)[5]: $228 ($152/year)

GDP per capita 2011 International $ (estimated annual wage)[5]: $716 ($477/year)

Prevalence of low BMI[6]: 11.1% (95% CI: 4.7, 19.7)

Prevalence of anemia[7]: 32.7% (95% CI: 23.9, 45.5)

Baseline coverage of IFA[8]: 1.4%

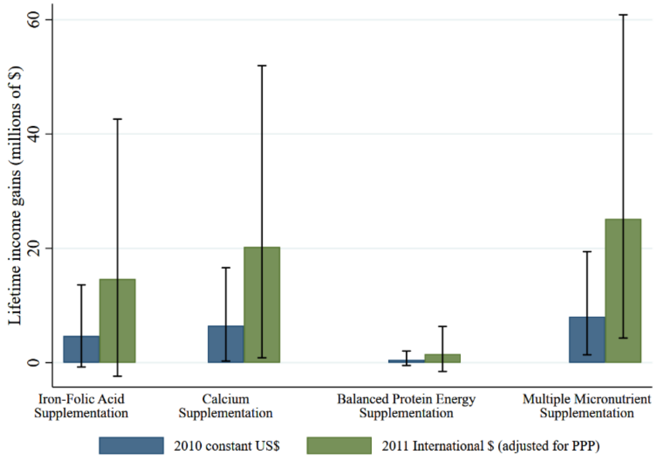

Figure: Benefits by birth cohort size for estimated gains in lifetime wages attributable to scaling up nutrition interventions to 90% target coverage

Table: Impact of maternal prenatal nutrition intervention on human capital and labour market outcomes, through improvements in low birthweight or preterm birth and schooling in Burundi.

Intervention	Target Coverage (%)	Absolute reduction in birth outcome (%)	Benefits by cohorts: School years gained (in 1000 s)	No. of additional students completing secondary school	Benefits by cohorts: Lifetime wages		Returns in lifetime earnings per child born to a targeted pregnant woman	
					in US $ millions	in International $ millions	in US $	in International $
Iron/Folic Acid Suppl.	90%	2.11 (-0.41, 4.53)	10.14 (-1.69, 29.01)	77 (-13, 221)	4.67 (-0.76, 13.60)	14.64 (-2.37, 42.61)	2.40 (-0.39, 6.99)	7.52 (-1.21, 21.88)
Calcium Suppl.	50%	1.40 (0.31, 2.64)	7.73 (0.31, 19.57)	59 (2, 149)	3.59 (0.15, 9.22)	11.24 (0.48, 28.88)	3.32 (0.14, 8.52)	10.39 (0.44, 26.69)
	90%	2.52 (0.56, 4.75)	13.91 (0.56, 35.22)	106 (4, 268)	6.46 (0.27, 16.59)	20.23 (0.86, 51.98)	3.32 (0.14, 8.52)	10.39 (0.44, 26.69)
Multiple Micronutrient Suppl.	50%	1.89 (0.66, 3.25)	9.52 (1.66, 22.73)	72 (13, 173)	4.43 (0.76, 10.71)	13.87 (2.37, 33.55)	4.09 (0.70, 9.90)	12.82 (2.19, 31.01)
	90%	3.42 (1.18, 5.90)	17.26 (3.01, 41.33)	131 (23, 314)	8.03 (1.37, 19.43)	25.16 (4.29, 60.88)	4.12 (0.70, 9.98)	12.92 (2.20, 31.26)
Balanced Protein Suppl.	50%	0.12 (-0.10, 0.41)	0.59 (-0.59, 2.39)	4 (-5, 18)	0.27 (-0.27, 1.12)	0.84 (-0.85, 3.51)	2.47 (-2.26, 8.06)	7.74 (-7.07, 25.24)
	90%	0.22 (-0.19, 0.74)	1.06 (-1.07, 4.30)	8 (-8, 33)	0.48 (-0.49, 2.02)	1.52 (-1.54, 6.33)	2.47 (-2.26, 8.06)	7.74 (-7.07, 25.24)

References for Data Inputs

[1] Blencowe H, Krasevec J, Onis M De, et al. Articles National , regional , and worldwide estimates of low birthweight in 2015 , with trends from 2000: a systematic analysis. Lancet Glob Heal. 2019;(18):1-12.
[2] Chawanpaiboon S, Vogel JP, Moller AB, et al. Global, regional, and national estimates of levels of preterm birth in 2014: a systematic review and modelling analysis. Lancet Glob Heal. 2019;7(1):e37-e46.
[3] United National Population Division World Population Prospects 2019.
[4] Fink G, Peet E, Danaei G, et al. Schooling and wage income losses due to early-childhood growth faltering in developing countries: National, regional, and global estimates. Am J Clin Nutr. 2016;104(1):104-112.
[5] Country specific annual wage data from World Indicators Database. Average yearly wage was estimated to be 2/3 of the gross domestic product in 2010 constant US dollars and 2011 International dollars, adjusted for purchasing power parity.
[6] NCD Risk Factor Collaboration. Trends in adult body-mass index in 200 countries from 1975 to 2014: a pooled analysis of 1698 population-based measurement studies with 19·2 million participants. Lancet. 2016;387(10026):1377-1396.
[7] Stevens GA, Finucane MM, De-Regil LM, et al. Global, regional, and national trends in haemoglobin concentration and prevalence of total and severe anaemia in children and pregnant and non-pregnant women for 1995-2011: A systematic analysis of population-representative data. Lancet Glob Heal. 2013;1(1):16-25.
[8] Coverage of iron-folic acid supplementation abstracted from the most recent Demographic Health Survey or imputed based on sub-regional average. Indicator used: % women in the past five years who took iron tablets or syrup for >90 days.

Cabo Verde

Region: Sub-Saharan Africa**; Sub-region:** Western Sub-Saharan Africa

Low birthweight prevalence[1]: 6.0% (95% CI: 0.7, 11.4)

Preterm birth prevalence[2]: 12.0% (95% CI: 8.6, 16.7)

Number of births[3]: 53,000

Returns to education[4]: 6.3% (95% CI: 4.5, 8.1)

GDP per capita 2010 US$ (estimated annual wage)[5]: $3415 ($2276/year)

GDP per capita 2011 International $ (estimated annual wage)[5]: $6007 ($4005/year)

Prevalence of low BMI[6]: 7.0% (95% CI: 2.7, 13.4)

Prevalence of anemia[7]: 43.1% (95% CI: 22.3, 64.4)

Baseline coverage of IFA[8]: 36.8%

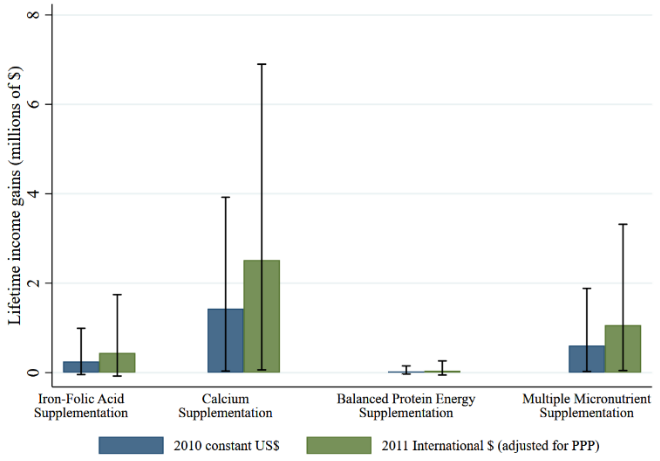

Figure: Benefits by birth cohort size for estimated gains in lifetime wages attributable to scaling up nutrition interventions to 90% target coverage

Table: Impact of maternal prenatal nutrition intervention on human capital and labour market outcomes, through improvements in low birthweight or preterm birth and schooling in Cabo Verde.

Intervention	Target Coverage (%)	Absolute reduction in birth outcome (%)	Benefits by cohorts: School years gained (in 1000 s)	No. of additional students completing secondary school	Benefits by cohorts: Lifetime wages		Returns in lifetime earnings per child born to a targeted pregnant woman	
					in US $ millions	in International $ millions	in US $	in International $
Iron/Folic Acid Suppl.	90%	0.48 (-0.08, 1.48)	0.07 (-0.01, 0.26)	2 (-0, 10)	0.25 (-0.04, 0.99)	0.44 (-0.08, 1.75)	5.23 (-0.90, 20.81)	9.19 (-1.58, 36.62)
Calcium Suppl.	50%	1.40 (0.32, 2.59)	0.21 (0.01, 0.57)	8 (0, 21)	0.80 (0.02, 2.18)	1.40 (0.04, 3.83)	30.01 (0.75, 82.23)	52.79 (1.32, 144.67)
	90%	2.52 (0.57, 4.66)	0.38 (0.01, 1.03)	14 (0, 38)	1.43 (0.04, 3.92)	2.52 (0.06, 6.90)	30.01 (0.75, 82.23)	52.79 (1.32, 144.67)
Multiple Micronutrient Suppl.	50%	0.48 (0.04, 0.99)	0.06 (0.00, 0.20)	2 (0, 7)	0.25 (0.01, 0.77)	0.44 (0.02, 1.35)	9.46 (0.48, 29.01)	16.65 (0.85, 51.04)
	90%	1.13 (0.09, 2.53)	0.16 (0.01, 0.49)	6 (0, 18)	0.60 (0.03, 1.89)	1.06 (0.05, 3.32)	12.64 (0.55, 39.54)	22.23 (0.97, 69.56)
Balanced Protein Suppl.	50%	0.03 (-0.03, 0.12)	0.00 (-0.00, 0.02)	0 (-0, 1)	0.01 (-0.02, 0.08)	0.02 (-0.03, 0.15)	8.09 (-9.20, 38.86)	14.23 (-16.18, 68.36)
	90%	0.05 (-0.06, 0.22)	0.01 (-0.01, 0.04)	0 (-0, 1)	0.02 (-0.03, 0.15)	0.04 (-0.06, 0.26)	8.09 (-9.20, 38.86)	14.23 (-16.18, 68.36)

References for Data Inputs

[1] Blencowe H, Krasevec J, Onis M De, et al. Articles National , regional , and worldwide estimates of low birthweight in 2015 , with trends from 2000: a systematic analysis. Lancet Glob Heal. 2019;(18):1-12.

[2] Chawanpaiboon S, Vogel JP, Moller AB, et al. Global, regional, and national estimates of levels of preterm birth in 2014: a systematic review and modelling analysis. Lancet Glob Heal. 2019;7(1):e37-e46.

[3] United National Population Division World Population Prospects 2019.

[4] Fink G, Peet E, Danaei G, et al. Schooling and wage income losses due to early-childhood growth faltering in developing countries: National, regional, and global estimates. Am J Clin Nutr. 2016;104(1):104-112.

[5] Country specific annual wage data from World Indicators Database. Average yearly wage was estimated to be 2/3 of the gross domestic product in 2010 constant US dollars and 2011 International dollars, adjusted for purchasing power parity.

[6] NCD Risk Factor Collaboration. Trends in adult body-mass index in 200 countries from 1975 to 2014: a pooled analysis of 1698 population-based measurement studies with 19·2 million participants. Lancet. 2016;387(10026):1377-1396.

[7] Stevens GA, Finucane MM, De-Regil LM, et al. Global, regional, and national trends in haemoglobin concentration and prevalence of total and severe anaemia in children and pregnant and non-pregnant women for 1995-2011: A systematic analysis of population-representative data. Lancet Glob Heal. 2013;1(1):16-25.

[8] Coverage of iron-folic acid supplementation abstracted from the most recent Demographic Health Survey or imputed based on sub-regional average. Indicator used: % women in the past five years who took iron tablets or syrup for >90 days.

Cambodia

Region: Southeast Asia, East Asia, and Oceania**; Sub-region:** Southeast Asia

Low birthweight prevalence[1]: 12.1% (95% CI: 7.6, 16.4)

Preterm birth prevalence[2]: 10.4% (95% CI: 8.7, 11.9)

Number of births[3]: 1,832,000

Returns to education[4]: 6.5% (95% CI: 2.1, 10.9)

GDP per capita 2010 US$ (estimated annual wage)[5]: $1025 ($683/year)

GDP per capita 2011 International $ (estimated annual wage)[5]: $3290 ($2193/year)

Prevalence of low BMI[6]: 14.1% (95% CI: 7.9, 21.4)

Prevalence of anemia[7]: 52.0% (95% CI: 42.4, 60.7)

Baseline coverage of IFA[8]: 75.5%

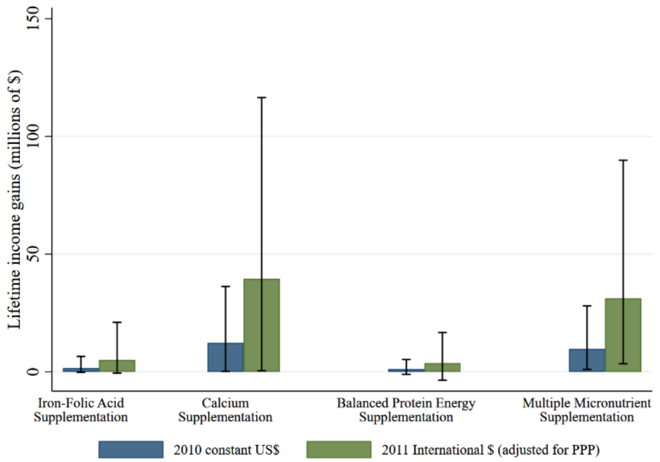

Figure: Benefits by birth cohort size for estimated gains in lifetime wages attributable to scaling up nutrition interventions to 90% target coverage

Table: Impact of maternal prenatal nutrition intervention on human capital and labour market outcomes, through improvements in low birthweight or preterm birth and schooling in Cambodia.

Intervention	Target Coverage (%)	Absolute reduction in birth outcome (%)	Benefits by cohorts: School years gained (in 1000 s)	No. of additional students completing secondary school	Benefits by cohorts: Lifetime wages		Returns in lifetime earnings per child born to a targeted pregnant woman	
					in US $ millions	in International $ millions	in US $	in International $
Iron/Folic Acid Suppl.	90%	0.31 (-0.05, 0.77)	1.37 (-0.18, 4.59)	47 (-6, 157)	1.56 (-0.18, 6.54)	5.02 (-0.58, 21.00)	0.95 (-0.11, 3.97)	3.04 (-0.35, 12.73)
Calcium Suppl.	50%	1.23 (0.26, 2.07)	6.26 (0.18, 15.21)	214 (6, 520)	6.83 (0.08, 20.16)	21.94 (0.25, 64.72)	7.46 (0.08, 22.00)	23.95 (0.27, 70.66)
	90%	2.22 (0.48, 3.73)	11.27 (0.33, 27.38)	385 (11, 936)	12.30 (0.14, 36.28)	39.49 (0.45, 116.50)	7.46 (0.08, 22.00)	23.95 (0.27, 70.66)
Multiple Micronutrient Suppl.	50%	0.83 (0.43, 1.38)	4.06 (0.77, 10.27)	139 (26, 351)	4.63 (0.47, 13.52)	14.88 (1.51, 43.40)	5.06 (0.51, 14.76)	16.25 (1.65, 47.38)
	90%	1.77 (0.93, 2.82)	8.59 (1.69, 20.55)	294 (58, 703)	9.72 (1.06, 28.00)	31.21 (3.39, 89.91)	5.89 (0.64, 16.98)	18.93 (2.06, 54.53)
Balanced Protein Suppl.	50%	0.13 (-0.10, 0.41)	0.60 (-0.54, 2.27)	20 (-19, 78)	0.63 (-0.61, 2.89)	2.02 (-1.97, 9.29)	5.14 (-4.77, 21.13)	16.50 (-15.30, 67.85)
	90%	0.24 (-0.18, 0.73)	1.07 (-0.97, 4.09)	37 (-33, 140)	1.13 (-1.11, 5.21)	3.64 (-3.55, 16.72)	5.14 (-4.77, 21.13)	16.50 (-15.30, 67.85)

References for Data Inputs
[1] Blencowe H, Krasevec J, Onis M De, et al. Articles National , regional , and worldwide estimates of low birthweight in 2015 , with trends from 2000: a systematic analysis. Lancet Glob Heal. 2019;(18):1-12.
[2] Chawanpaiboon S, Vogel JP, Moller AB, et al. Global, regional, and national estimates of levels of preterm birth in 2014: a systematic review and modelling analysis. Lancet Glob Heal. 2019;7(1):e37-e46.
[3] United National Population Division World Population Prospects 2019.
[4] Fink G, Peet E, Danaei G, et al. Schooling and wage income losses due to early-childhood growth faltering in developing countries: National, regional, and global estimates. Am J Clin Nutr. 2016;104(1):104-112.
[5] Country specific annual wage data from World Indicators Database. Average yearly wage was estimated to be 2/3 of the gross domestic product in 2010 constant US dollars and 2011 International dollars, adjusted for purchasing power parity.
[6] NCD Risk Factor Collaboration. Trends in adult body-mass index in 200 countries from 1975 to 2014: a pooled analysis of 1698 population-based measurement studies with 19·2 million participants. Lancet. 2016;387(10026):1377-1396.
[7] Stevens GA, Finucane MM, De-Regil LM, et al. Global, regional, and national trends in haemoglobin concentration and prevalence of total and severe anaemia in children and pregnant and non-pregnant women for 1995-2011: A systematic analysis of population-representative data. Lancet Glob Heal. 2013;1(1):16-25.
[8] Coverage of iron-folic acid supplementation abstracted from the most recent Demographic Health Survey or imputed based on sub-regional average. Indicator used: % women in the past five years who took iron tablets or syrup for >90 days.

Cameroon

Region: Sub-Saharan Africa**; Sub-region:** Western Sub-Saharan Africa

Low birthweight prevalence[1]: 12.0% (95% CI: 9.3, 15.2)

Preterm birth prevalence[2]: 12.0% (95% CI: 8.6, 16.7)

Number of births[3]: 4,437,000

Returns to education[4]: 6.3% (95% CI: 4.5, 8.1)

GDP per capita 2010 US$ (estimated annual wage)[5]: $1440 ($960/year)

GDP per capita 2011 International $ (estimated annual wage)[5]: $3224 ($2149/year)

Prevalence of low BMI[6]: 6.1% (95% CI: 3.1, 10.2)

Prevalence of anemia[7]: 50.0% (95% CI: 42.3, 58.1)

Baseline coverage of IFA[8]: 54.2%

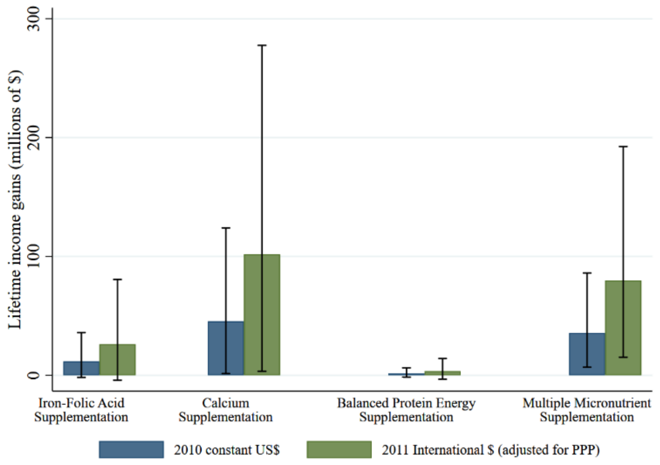

Figure: Benefits by birth cohort size for estimated gains in lifetime wages attributable to scaling up nutrition interventions to 90% target coverage

Table: Impact of maternal prenatal nutrition intervention on human capital and labour market outcomes, through improvements in low birthweight or preterm birth and schooling in Cameroon.

Intervention	Target Coverage (%)	Absolute reduction in birth outcome (%)	Benefits by cohorts: School years gained (in 1000 s)	No. of additional students completing secondary school	Benefits by cohorts: Lifetime wages		Returns in lifetime earnings per child born to a targeted pregnant woman	
					in US $ millions	in International $ millions	in US $	in International $
Iron/Folic Acid Suppl.	90%	0.73 (-0.12, 1.67)	7.36 (-1.10, 21.71)	157 (-24, 465)	11.71 (-1.82, 36.01)	26.22 (-4.08, 80.59)	2.93 (-0.46, 9.02)	6.57 (-1.02, 20.18)
Calcium Suppl.	50%	1.40 (0.30, 2.66)	15.75 (0.47, 39.82)	337 (10, 852)	25.26 (0.83, 68.91)	56.53 (1.85, 154.21)	11.38 (0.37, 31.06)	25.48 (0.84, 69.51)
	90%	2.51 (0.54, 4.80)	28.36 (0.85, 71.68)	607 (18, 1534)	45.46 (1.49, 124.03)	101.75 (3.34, 277.58)	11.38 (0.37, 31.06)	25.48 (0.84, 69.51)
Multiple Micronutrient Suppl.	50%	0.83 (0.47, 1.21)	8.90 (1.56, 19.73)	191 (33, 422)	14.31 (2.51, 33.99)	32.02 (5.61, 76.07)	6.45 (1.13, 15.32)	14.43 (2.53, 34.29)
	90%	2.11 (1.15, 3.17)	22.23 (4.29, 48.74)	476 (92, 1043)	35.66 (6.79, 86.02)	79.80 (15.20, 192.50)	8.93 (1.70, 21.54)	19.98 (3.81, 48.21)
Balanced Protein Suppl.	50%	0.06 (-0.05, 0.17)	0.56 (-0.54, 2.06)	12 (-12, 44)	0.87 (-0.83, 3.52)	1.94 (-1.86, 7.88)	6.85 (-6.52, 23.77)	15.32 (-14.58, 53.20)
	90%	0.10 (-0.08, 0.31)	1.00 (-0.98, 3.72)	21 (-21, 80)	1.56 (-1.50, 6.34)	3.50 (-3.35, 14.18)	6.85 (-6.52, 23.77)	15.32 (-14.58, 53.20)

References for Data Inputs

[1] Blencowe H, Krasevec J, Onis M De, et al. Articles National , regional , and worldwide estimates of low birthweight in 2015 , with trends from 2000: a systematic analysis. Lancet Glob Heal. 2019;(18):1-12.
[2] Chawanpaiboon S, Vogel JP, Moller AB, et al. Global, regional, and national estimates of levels of preterm birth in 2014: a systematic review and modelling analysis. Lancet Glob Heal. 2019;7(1):e37-e46.
[3] United National Population Division World Population Prospects 2019.
[4] Fink G, Peet E, Danaei G, et al. Schooling and wage income losses due to early-childhood growth faltering in developing countries: National, regional, and global estimates. Am J Clin Nutr. 2016;104(1):104-112.
[5] Country specific annual wage data from World Indicators Database. Average yearly wage was estimated to be 2/3 of the gross domestic product in 2010 constant US dollars and 2011 International dollars, adjusted for purchasing power parity.
[6] NCD Risk Factor Collaboration. Trends in adult body-mass index in 200 countries from 1975 to 2014: a pooled analysis of 1698 population-based measurement studies with 19·2 million participants. Lancet. 2016;387(10026):1377-1396.
[7] Stevens GA, Finucane MM, De-Regil LM, et al. Global, regional, and national trends in haemoglobin concentration and prevalence of total and severe anaemia in children and pregnant and non-pregnant women for 1995-2011: A systematic analysis of population-representative data. Lancet Glob Heal. 2013;1(1):16-25.
[8] Coverage of iron-folic acid supplementation abstracted from the most recent Demographic Health Survey or imputed based on sub-regional average. Indicator used: % women in the past five years who took iron tablets or syrup for >90 days.

Chad

Region: Sub-Saharan Africa**; Sub-region:** Western Sub-Saharan Africa

Low birthweight prevalence[1]: 21.7% (95% CI: 16.4, 27.1)

Preterm birth prevalence[2]: 12.0% (95% CI: 8.6, 16.7)

Number of births[3]: 3,239,000

Returns to education[4]: 6.3% (95% CI: 4.5, 8.1)

GDP per capita 2010 US$ (estimated annual wage)[5]: $956 ($637/year)

GDP per capita 2011 International $ (estimated annual wage)[5]: $2052 ($1368/year)

Prevalence of low BMI[6]: 13.2% (95% CI: 6.9, 21.1)

Prevalence of anemia[7]: 51.5% (95% CI: 30.9, 68.8)

Baseline coverage of IFA[8]: 11.0%

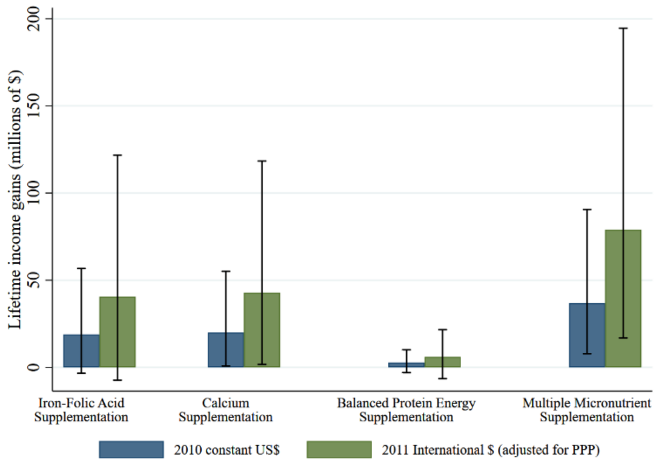

Figure: Benefits by birth cohort size for estimated gains in lifetime wages attributable to scaling up nutrition interventions to 90% target coverage

Table: Impact of maternal prenatal nutrition intervention on human capital and labour market outcomes, through improvements in low birthweight or preterm birth and schooling in Chad.

Intervention	Target Coverage (%)	Absolute reduction in birth outcome (%)	Benefits by cohorts: School years gained (in 1000 s)	No. of additional students completing secondary school	Benefits by cohorts: Lifetime wages		Returns in lifetime earnings per child born to a targeted pregnant woman	
					in US $ millions	in International $ millions	in US $	in International $
Iron/Folic Acid Suppl.	90%	2.75 (-0.53, 5.86)	17.77 (-3.24, 51.02)	875 (-159, 2513)	18.88 (-3.42, 56.68)	40.54 (-7.35, 121.71)	6.48 (-1.17, 19.44)	13.91 (-2.52, 41.75)
Calcium Suppl.	50%	1.40 (0.30, 2.54)	10.31 (0.38, 27.01)	508 (19, 1330)	11.08 (0.44, 30.62)	23.79 (0.95, 65.74)	6.84 (0.27, 18.90)	14.69 (0.59, 40.59)
	90%	2.52 (0.55, 4.58)	18.56 (0.69, 48.62)	914 (34, 2395)	19.94 (0.80, 55.11)	42.82 (1.71, 118.33)	6.84 (0.27, 18.90)	14.69 (0.59, 40.59)
Multiple Micronutrient Suppl.	50%	2.65 (1.22, 4.14)	18.11 (3.64, 41.54)	892 (179, 2046)	19.38 (4.13, 46.90)	41.61 (8.88, 100.71)	11.97 (2.55, 28.96)	25.69 (5.48, 62.18)
	90%	5.09 (2.22, 8.08)	34.67 (6.84, 80.30)	1708 (337, 3955)	36.77 (7.84, 90.61)	78.96 (16.83, 194.57)	12.61 (2.69, 31.08)	27.09 (5.77, 66.75)
Balanced Protein Suppl.	50%	0.24 (-0.21, 0.64)	1.48 (-1.50, 5.40)	73 (-74, 266)	1.55 (-1.67, 5.59)	3.33 (-3.59, 12.00)	7.60 (-6.92, 24.87)	16.33 (-14.86, 53.40)
	90%	0.44 (-0.38, 1.16)	2.66 (-2.69, 9.71)	131 (-133, 478)	2.79 (-3.01, 10.06)	6.00 (-6.46, 21.61)	7.60 (-6.92, 24.87)	16.33 (-14.86, 53.40)

References for Data Inputs

[1] Blencowe H, Krasevec J, Onis M De, et al. Articles National , regional , and worldwide estimates of low birthweight in 2015 , with trends from 2000: a systematic analysis. Lancet Glob Heal. 2019;(18):1-12.

[2] Chawanpaiboon S, Vogel JP, Moller AB, et al. Global, regional, and national estimates of levels of preterm birth in 2014: a systematic review and modelling analysis. Lancet Glob Heal. 2019;7(1):e37-e46.

[3] United National Population Division World Population Prospects 2019.

[4] Fink G, Peet E, Danaei G, et al. Schooling and wage income losses due to early-childhood growth faltering in developing countries: National, regional, and global estimates. Am J Clin Nutr. 2016;104(1):104-112.

[5] Country specific annual wage data from World Indicators Database. Average yearly wage was estimated to be 2/3 of the gross domestic product in 2010 constant US dollars and 2011 International dollars, adjusted for purchasing power parity.

[6] NCD Risk Factor Collaboration. Trends in adult body-mass index in 200 countries from 1975 to 2014: a pooled analysis of 1698 population-based measurement studies with 19·2 million participants. Lancet. 2016;387(10026):1377-1396.

[7] Stevens GA, Finucane MM, De-Regil LM, et al. Global, regional, and national trends in haemoglobin concentration and prevalence of total and severe anaemia in children and pregnant and non-pregnant women for 1995-2011: A systematic analysis of population-representative data. Lancet Glob Heal. 2013;1(1):16-25.

[8] Coverage of iron-folic acid supplementation abstracted from the most recent Demographic Health Survey or imputed based on sub-regional average. Indicator used: % women in the past five years who took iron tablets or syrup for >90 days.

China

Region: Southeast Asia, East Asia, and Oceania**; Sub-region:** East Asia

Low birthweight prevalence[1]: 5.0% (95% CI: 3.9, 6.4)

Preterm birth prevalence[2]: 6.9% (95% CI: 5.8, 7.9)

Number of births[3]: 84,890,000

Returns to education[4]: 4.1% (95% CI: 3.3, 5.0)

GDP per capita 2010 US$ (estimated annual wage)[5]: $6484 ($4323/year)

GDP per capita 2011 International $ (estimated annual wage)[5]: $13535 ($9023/year)

Prevalence of low BMI[6]: 6.2% (95% CI: 4.1, 8.7)

Prevalence of anemia[7]: 21.7% (95% CI: 10.7, 36.8)

Baseline coverage of IFA[8]: 44.2%

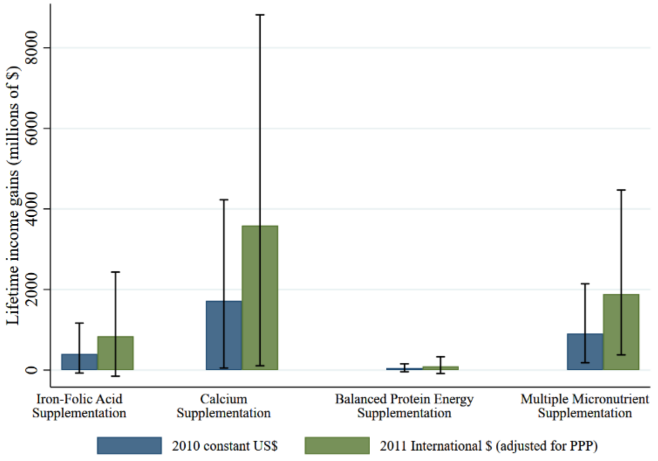

Figure: Benefits by birth cohort size for estimated gains in lifetime wages attributable to scaling up nutrition interventions to 90% target coverage

Table: Impact of maternal prenatal nutrition intervention on human capital and labour market outcomes, through improvements in low birthweight or preterm birth and schooling in China.

Intervention	Target Coverage (%)	Absolute reduction in birth outcome (%)	Benefits by cohorts: School years gained (in 1000 s)	No. of additional students completing secondary school	Benefits by cohorts: Lifetime wages		Returns in lifetime earnings per child born to a targeted pregnant woman	
					in US $ millions	in International $ millions	in US $	in International $
Iron/Folic Acid Suppl.	90%	0.39 (-0.07, 0.87)	85.08 (-16.57, 244.05)	7930 (-1544, 22745)	401.44 (-72.45, 1164.92)	837.93 (-151.23, 2431.51)	5.25 (-0.95, 15.25)	10.97 (-1.98, 31.83)
Calcium Suppl.	50%	0.82 (0.18, 1.41)	202.19 (6.00, 479.83)	18844 (559, 44720)	955.08 (28.28, 2348.35)	1993.53 (59.03, 4901.66)	22.50 (0.67, 55.33)	46.97 (1.39, 115.48)
	90%	1.47 (0.32, 2.53)	363.95 (10.80, 863.70)	33920 (1006, 80497)	1719.15 (50.90, 4227.02)	3588.35 (106.25, 8822.99)	22.50 (0.67, 55.33)	46.97 (1.39, 115.48)
Multiple Micronutrient Suppl.	50%	0.30 (0.15, 0.46)	69.85 (14.19, 157.63)	6510 (1322, 14691)	332.89 (68.26, 771.23)	694.84 (142.49, 1609.78)	7.84 (1.61, 18.17)	16.37 (3.36, 37.93)
	90%	0.84 (0.38, 1.36)	189.99 (39.44, 422.31)	17707 (3676, 39360)	904.59 (180.45, 2142.69)	1888.14 (376.66, 4472.39)	11.84 (2.36, 28.05)	24.71 (4.93, 58.54)
Balanced Protein Suppl.	50%	0.03 (-0.02, 0.07)	5.29 (-4.89, 17.88)	493 (-456, 1666)	25.43 (-23.14, 87.52)	53.09 (-48.29, 182.68)	10.08 (-9.32, 33.14)	21.04 (-19.46, 69.17)
	90%	0.05 (-0.04, 0.12)	9.52 (-8.81, 32.18)	887 (-821, 2999)	45.78 (-41.65, 157.54)	95.56 (-86.93, 328.82)	10.08 (-9.32, 33.14)	21.04 (-19.46, 69.17)

References for Data Inputs

[1] Blencowe H, Krasevec J, Onis M De, et al. Articles National , regional , and worldwide estimates of low birthweight in 2015 , with trends from 2000: a systematic analysis. Lancet Glob Heal. 2019;(18):1-12.
[2] Chawanpaiboon S, Vogel JP, Moller AB, et al. Global, regional, and national estimates of levels of preterm birth in 2014: a systematic review and modelling analysis. Lancet Glob Heal. 2019;7(1):e37-e46.
[3] United National Population Division World Population Prospects 2019.
[4] Fink G, Peet E, Danaei G, et al. Schooling and wage income losses due to early-childhood growth faltering in developing countries: National, regional, and global estimates. Am J Clin Nutr. 2016;104(1):104-112.
[5] Country specific annual wage data from World Indicators Database. Average yearly wage was estimated to be 2/3 of the gross domestic product in 2010 constant US dollars and 2011 International dollars, adjusted for purchasing power parity.
[6] NCD Risk Factor Collaboration. Trends in adult body-mass index in 200 countries from 1975 to 2014: a pooled analysis of 1698 population-based measurement studies with 19·2 million participants. Lancet. 2016;387(10026):1377-1396.
[7] Stevens GA, Finucane MM, De-Regil LM, et al. Global, regional, and national trends in haemoglobin concentration and prevalence of total and severe anaemia in children and pregnant and non-pregnant women for 1995-2011: A systematic analysis of population-representative data. Lancet Glob Heal. 2013;1(1):16-25.
[8] Coverage of iron-folic acid supplementation abstracted from the most recent Demographic Health Survey or imputed based on sub-regional average. Indicator used: % women in the past five years who took iron tablets or syrup for >90 days.

Central African Republic

Region: Sub-Saharan Africa**; Sub-region:** Central Sub-Saharan Africa

Low birthweight prevalence[1]: 14.5% (95% CI: 11.3, 18.1)

Preterm birth prevalence[2]: 12.0% (95% CI: 8.6, 16.7)

Number of births[3]: 825,000

Returns to education[4]: 9.9% (95% CI: 7.0, 12.9)

GDP per capita 2010 US$ (estimated annual wage)[5]: $349 ($233/year)

GDP per capita 2011 International $ (estimated annual wage)[5]: $703 ($469/year)

Prevalence of low BMI[6]: 12.2% (95% CI: 5.4, 21.3)

Prevalence of anemia[7]: 51.2% (95% CI: 32.9, 66.3)

Baseline coverage of IFA[8]: 34.1%

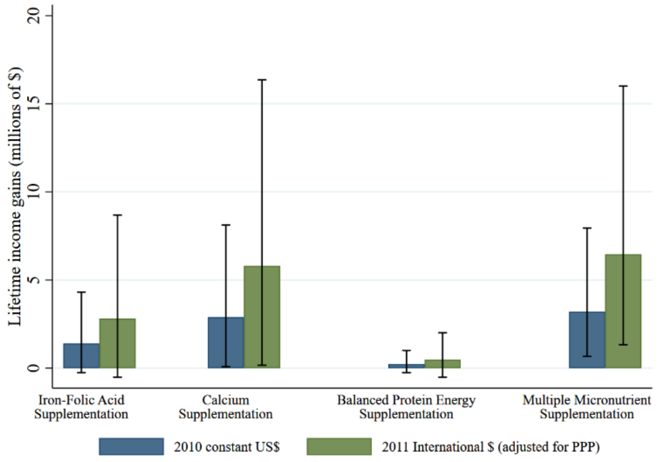

Figure: Benefits by birth cohort size for estimated gains in lifetime wages attributable to scaling up nutrition interventions to 90% target coverage

Table: Impact of maternal prenatal nutrition intervention on human capital and labour market outcomes, through improvements in low birthweight or preterm birth and schooling in Central African Republic.

Intervention	Target Coverage (%)	Absolute reduction in birth outcome (%)	Benefits by cohorts: School years gained (in 1000 s)	No. of additional students completing secondary school	Benefits by cohorts: Lifetime wages		Returns in lifetime earnings per child born to a targeted pregnant woman	
					in US $ millions	in International $ millions	in US $	in International $
**Iron/Folic Acid Suppl.**	90%	1.38 (-0.27, 2.92)	2.37 (-0.50, 6.61)	38 (-8, 107)	1.39 (-0.26, 4.31)	2.81 (-0.51, 8.68)	1.88 (-0.34, 5.81)	3.78 (-0.69, 11.70)
**Calcium Suppl.**	50%	1.39 (0.29, 2.66)	2.58 (0.08, 7.06)	42 (1, 114)	1.60 (0.04, 4.51)	3.23 (0.09, 9.09)	3.89 (0.11, 10.94)	7.82 (0.21, 22.03)
	90%	2.51 (0.52, 4.79)	4.65 (0.14, 12.70)	75 (2, 206)	2.89 (0.08, 8.12)	5.81 (0.16, 16.36)	3.89 (0.11, 10.94)	7.82 (0.21, 22.03)
**Multiple Micronutrient Suppl.**	50%	1.27 (0.74, 1.85)	2.37 (0.51, 5.19)	38 (8, 84)	1.41 (0.31, 3.34)	2.84 (0.62, 6.73)	3.43 (0.74, 8.10)	6.90 (1.50, 16.31)
	90%	2.96 (1.47, 4.58)	5.38 (1.15, 12.22)	87 (19, 198)	3.21 (0.66, 7.95)	6.45 (1.33, 16.01)	4.32 (0.89, 10.71)	8.69 (1.79, 21.56)
**Balanced Protein Suppl.**	50%	0.14 (-0.12, 0.43)	0.22 (-0.22, 0.91)	4 (-4, 15)	0.13 (-0.14, 0.55)	0.27 (-0.29, 1.11)	2.88 (-2.86, 9.60)	5.81 (-5.76, 19.32)
	90%	0.25 (-0.21, 0.77)	0.40 (-0.40, 1.64)	7 (-6, 27)	0.24 (-0.26, 1.00)	0.48 (-0.51, 2.01)	2.88 (-2.86, 9.60)	5.81 (-5.76, 19.32)

References for Data Inputs

[1] Blencowe H, Krasevec J, Onis M De, et al. Articles National , regional , and worldwide estimates of low birthweight in 2015 , with trends from 2000: a systematic analysis. Lancet Glob Heal. 2019;(18):1-12.
[2] Chawanpaiboon S, Vogel JP, Moller AB, et al. Global, regional, and national estimates of levels of preterm birth in 2014: a systematic review and modelling analysis. Lancet Glob Heal. 2019;7(1):e37-e46.
[3] United National Population Division World Population Prospects 2019.
[4] Fink G, Peet E, Danaei G, et al. Schooling and wage income losses due to early-childhood growth faltering in developing countries: National, regional, and global estimates. Am J Clin Nutr. 2016;104(1):104-112.
[5] Country specific annual wage data from World Indicators Database. Average yearly wage was estimated to be 2/3 of the gross domestic product in 2010 constant US dollars and 2011 International dollars, adjusted for purchasing power parity.
[6] NCD Risk Factor Collaboration. Trends in adult body-mass index in 200 countries from 1975 to 2014: a pooled analysis of 1698 population-based measurement studies with 19·2 million participants. Lancet. 2016;387(10026):1377-1396.
[7] Stevens GA, Finucane MM, De-Regil LM, et al. Global, regional, and national trends in haemoglobin concentration and prevalence of total and severe anaemia in children and pregnant and non-pregnant women for 1995-2011: A systematic analysis of population-representative data. Lancet Glob Heal. 2013;1(1):16-25.
[8] Coverage of iron-folic acid supplementation abstracted from the most recent Demographic Health Survey or imputed based on sub-regional average. Indicator used: % women in the past five years who took iron tablets or syrup for >90 days.

Colombia

Region: Latin America and Caribbean**; Sub-region:** Central Latin America

Low birthweight prevalence[1]: 10.0% (95% CI: 7.7, 13.2)

Preterm birth prevalence[2]: 14.5% (95% CI: 11.4, 18.2)

Number of births[3]: 3,697,000

Returns to education[4]: 9.5% (95% CI: 7.5, 11.6)

GDP per capita 2010 US$ (estimated annual wage)[5]: $7572 ($5048/year)

GDP per capita 2011 International $ (estimated annual wage)[5]: $13115 ($8743/year)

Prevalence of low BMI[6]: 2.5% (95% CI: 1.3, 4.1)

Prevalence of anemia[7]: 31.4% (95% CI: 19.4, 47.3)

Baseline coverage of IFA[8]: 33.3%

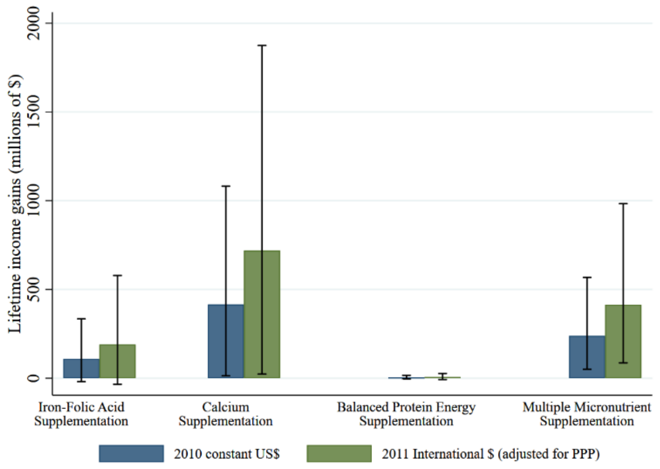

Figure: Benefits by birth cohort size for estimated gains in lifetime wages attributable to scaling up nutrition interventions to 90% target coverage

Table: Impact of maternal prenatal nutrition intervention on human capital and labour market outcomes, through improvements in low birthweight or preterm birth and schooling in Colombia.

Intervention	Target Coverage (%)	Absolute reduction in birth outcome (%)	Benefits by cohorts: School years gained (in 1000 s)	No. of additional students completing secondary school	Benefits by cohorts: Lifetime wages		Returns in lifetime earnings per child born to a targeted pregnant woman	
					in US $ millions	in International $ millions	in US $	in International $
Iron/Folic Acid Suppl.	90%	0.95 (-0.19, 2.04)	8.75 (-1.47, 25.14)	280 (-47, 804)	109.52 (-19.70, 334.07)	189.68 (-34.12, 578.58)	32.92 (-5.92, 100.40)	57.01 (-10.25, 173.89)
Calcium Suppl.	50%	1.73 (0.37, 3.07)	18.31 (0.56, 46.30)	586 (18, 1482)	230.66 (7.36, 601.15)	399.48 (12.75, 1041.13)	124.78 (3.98, 325.21)	216.11 (6.90, 563.23)
	90%	3.11 (0.67, 5.53)	32.95 (1.01, 83.35)	1055 (32, 2667)	415.19 (13.25, 1082.07)	719.06 (22.95, 1874.03)	124.78 (3.98, 325.21)	216.11 (6.90, 563.23)
Multiple Micronutrient Suppl.	50%	0.82 (0.45, 1.26)	8.06 (1.78, 17.06)	258 (57, 546)	104.65 (23.39, 235.06)	181.25 (40.50, 407.09)	56.61 (12.65, 127.16)	98.05 (21.91, 220.23)
	90%	1.92 (0.81, 3.08)	18.42 (3.79, 41.69)	589 (121, 1334)	239.08 (49.87, 567.60)	414.06 (86.36, 983.02)	71.85 (14.99, 170.59)	124.44 (25.96, 295.44)
Balanced Protein Suppl.	50%	0.02 (-0.02, 0.06)	0.17 (-0.19, 0.67)	5 (-6, 21)	2.22 (-2.42, 8.58)	3.85 (-4.20, 14.86)	51.41 (-50.45, 175.68)	89.04 (-87.38, 304.27)
	90%	0.03 (-0.03, 0.11)	0.31 (-0.34, 1.20)	10 (-11, 39)	4.00 (-4.36, 15.44)	6.92 (-7.56, 26.75)	51.41 (-50.45, 175.68)	89.04 (-87.38, 304.27)

References for Data Inputs
[1] Blencowe H, Krasevec J, Onis M De, et al. Articles National , regional , and worldwide estimates of low birthweight in 2015 , with trends from 2000: a systematic analysis. Lancet Glob Heal. 2019;(18):1-12.
[2] Chawanpaiboon S, Vogel JP, Moller AB, et al. Global, regional, and national estimates of levels of preterm birth in 2014: a systematic review and modelling analysis. Lancet Glob Heal. 2019;7(1):e37-e46.
[3] United National Population Division World Population Prospects 2019.
[4] Fink G, Peet E, Danaei G, et al. Schooling and wage income losses due to early-childhood growth faltering in developing countries: National, regional, and global estimates. Am J Clin Nutr. 2016;104(1):104-112.
[5] Country specific annual wage data from World Indicators Database. Average yearly wage was estimated to be 2/3 of the gross domestic product in 2010 constant US dollars and 2011 International dollars, adjusted for purchasing power parity.
[6] NCD Risk Factor Collaboration. Trends in adult body-mass index in 200 countries from 1975 to 2014: a pooled analysis of 1698 population-based measurement studies with 19·2 million participants. Lancet. 2016;387(10026):1377-1396.
[7] Stevens GA, Finucane MM, De-Regil LM, et al. Global, regional, and national trends in haemoglobin concentration and prevalence of total and severe anaemia in children and pregnant and non-pregnant women for 1995-2011: A systematic analysis of population-representative data. Lancet Glob Heal. 2013;1(1):16-25.
[8] Coverage of iron-folic acid supplementation abstracted from the most recent Demographic Health Survey or imputed based on sub-regional average. Indicator used: % women in the past five years who took iron tablets or syrup for >90 days.

Comoros

Region: Sub-Saharan Africa**; Sub-region:** Eastern Sub-Saharan Africa

Low birthweight prevalence[1]: 23.7% (95% CI: 18.9, 29.8)

Preterm birth prevalence[2]: 12.0% (95% CI: 8.6, 16.7)

Number of births[3]: 132,000

Returns to education[4]: 11.3% (95% CI: 9.7, 12.9)

GDP per capita 2010 US$ (estimated annual wage)[5]: $1352 ($901/year)

GDP per capita 2011 International $ (estimated annual wage)[5]: $2494 ($1663/year)

Prevalence of low BMI[6]: 8.4% (95% CI: 3.9, 14.6)

Prevalence of anemia[7]: 35.7% (95% CI: 19.4, 56.9)

Baseline coverage of IFA[8]: 12.6%

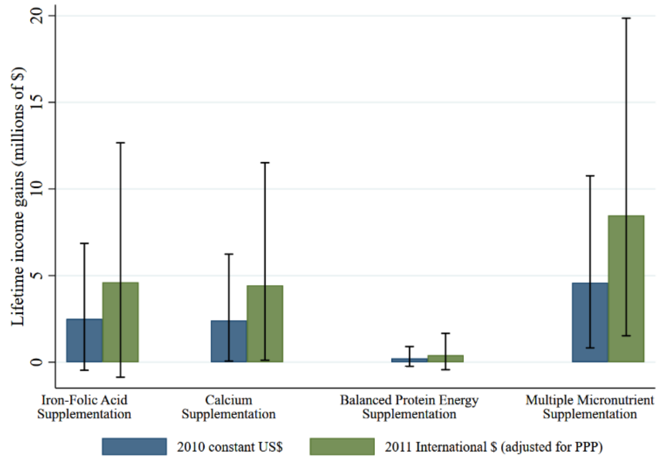

Figure: Benefits by birth cohort size for estimated gains in lifetime wages attributable to scaling up nutrition interventions to 90% target coverage

Table: Impact of maternal prenatal nutrition intervention on human capital and labour market outcomes, through improvements in low birthweight or preterm birth and schooling in Comoros.

Intervention	Target Coverage (%)	Absolute reduction in birth outcome (%)	Benefits by cohorts: School years gained (in 1000 s)	No. of additional students completing secondary school	Benefits by cohorts: Lifetime wages		Returns in lifetime earnings per child born to a targeted pregnant woman	
					in US $ millions	in International $ millions	in US $	in International $
Iron/Folic Acid Suppl.	90%	2.99 (-0.58, 6.21)	0.90 (-0.18, 2.49)	47 (-10, 132)	2.50 (-0.47, 6.86)	4.61 (-0.86, 12.66)	21.05 (-3.93, 57.78)	38.83 (-7.26, 106.59)
Calcium Suppl.	50%	1.41 (0.27, 2.55)	0.50 (0.01, 1.28)	26 (1, 68)	1.34 (0.04, 3.47)	2.46 (0.07, 6.40)	20.24 (0.55, 52.53)	37.34 (1.01, 96.90)
	90%	2.53 (0.48, 4.59)	0.90 (0.03, 2.31)	48 (1, 122)	2.40 (0.06, 6.24)	4.44 (0.12, 11.51)	20.24 (0.55, 52.53)	37.34 (1.01, 96.90)
Multiple Micronutrient Suppl.	50%	2.67 (1.09, 4.25)	0.87 (0.16, 2.01)	46 (9, 106)	2.38 (0.45, 5.55)	4.39 (0.84, 10.25)	36.03 (6.86, 84.15)	66.46 (12.66, 155.23)
	90%	5.23 (1.90, 8.39)	1.68 (0.30, 3.91)	89 (16, 206)	4.59 (0.83, 10.76)	8.47 (1.53, 19.85)	38.66 (6.98, 90.60)	71.32 (12.87, 167.12)
Balanced Protein Suppl.	50%	0.15 (-0.14, 0.45)	0.05 (-0.05, 0.18)	2 (-2, 10)	0.12 (-0.13, 0.50)	0.23 (-0.24, 0.92)	24.58 (-24.42, 77.44)	45.34 (-45.05, 142.86)
	90%	0.27 (-0.24, 0.81)	0.08 (-0.08, 0.33)	4 (-4, 18)	0.22 (-0.23, 0.90)	0.41 (-0.43, 1.66)	24.58 (-24.42, 77.44)	45.34 (-45.05, 142.86)

References for Data Inputs

[1] Blencowe H, Krasevec J, Onis M De, et al. Articles National , regional , and worldwide estimates of low birthweight in 2015 , with trends from 2000: a systematic analysis. Lancet Glob Heal. 2019;(18):1-12.

[2] Chawanpaiboon S, Vogel JP, Moller AB, et al. Global, regional, and national estimates of levels of preterm birth in 2014: a systematic review and modelling analysis. Lancet Glob Heal. 2019;7(1):e37-e46.

[3] United National Population Division World Population Prospects 2019.

[4] Fink G, Peet E, Danaei G, et al. Schooling and wage income losses due to early-childhood growth faltering in developing countries: National, regional, and global estimates. Am J Clin Nutr. 2016;104(1):104-112.

[5] Country specific annual wage data from World Indicators Database. Average yearly wage was estimated to be 2/3 of the gross domestic product in 2010 constant US dollars and 2011 International dollars, adjusted for purchasing power parity.

[6] NCD Risk Factor Collaboration. Trends in adult body-mass index in 200 countries from 1975 to 2014: a pooled analysis of 1698 population-based measurement studies with 19·2 million participants. Lancet. 2016;387(10026):1377-1396.

[7] Stevens GA, Finucane MM, De-Regil LM, et al. Global, regional, and national trends in haemoglobin concentration and prevalence of total and severe anaemia in children and pregnant and non-pregnant women for 1995-2011: A systematic analysis of population-representative data. Lancet Glob Heal. 2013;1(1):16-25.

[8] Coverage of iron-folic acid supplementation abstracted from the most recent Demographic Health Survey or imputed based on sub-regional average. Indicator used: % women in the past five years who took iron tablets or syrup for >90 days.

Congo

Region: Sub-Saharan Africa**; Sub-region:** Central Sub-Saharan Africa

Low birthweight prevalence[1]: 13.1% (95% CI: 8.3, 17.9)

Preterm birth prevalence[2]: 12.0% (95% CI: 8.6, 16.7)

Number of births[3]: 857,000

Returns to education[4]: 9.9% (95% CI: 7.0, 12.9)

GDP per capita 2010 US$ (estimated annual wage)[5]: $3010 ($2006/year)

GDP per capita 2011 International $ (estimated annual wage)[5]: $5248 ($3499/year)

Prevalence of low BMI[6]: 11.2% (95% CI: 6.0, 18.2)

Prevalence of anemia[7]: 60.1% (95% CI: 50.3, 68.4)

Baseline coverage of IFA[8]: 42.9%

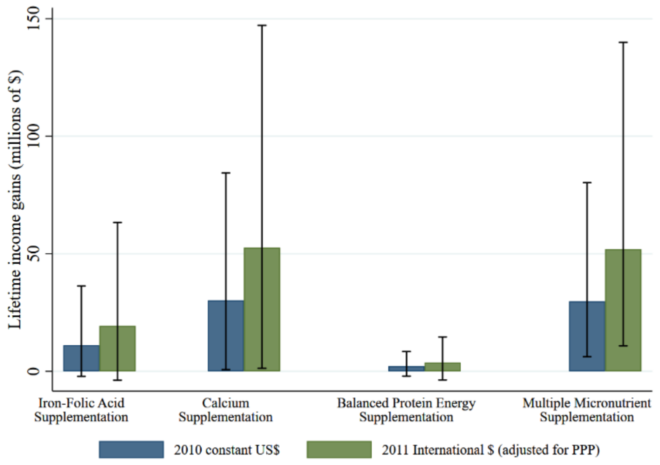

Figure: Benefits by birth cohort size for estimated gains in lifetime wages attributable to scaling up nutrition interventions to 90% target coverage

Table: Impact of maternal prenatal nutrition intervention on human capital and labour market outcomes, through improvements in low birthweight or preterm birth and schooling in Congo.

Intervention	Target Coverage (%)	Absolute reduction in birth outcome (%)	Benefits by cohorts: School years gained (in 1000 s)	No. of additional students completing secondary school	Benefits by cohorts: Lifetime wages		Returns in lifetime earnings per child born to a targeted pregnant woman	
					in US $ millions	in International $ millions	in US $	in International $
Iron/Folic Acid Suppl.	90%	1.02 (-0.20, 2.40)	2.08 (-0.36, 6.32)	180 (-31, 546)	11.09 (-2.16, 36.30)	19.34 (-3.77, 63.30)	14.38 (-2.80, 47.06)	25.08 (-4.88, 82.07)
Calcium Suppl.	50%	1.42 (0.32, 2.62)	3.23 (0.10, 8.32)	279 (8, 719)	16.76 (0.40, 46.88)	29.22 (0.70, 81.75)	39.11 (0.93, 109.41)	68.20 (1.62, 190.78)
	90%	2.55 (0.58, 4.72)	5.81 (0.17, 14.98)	502 (15, 1294)	30.16 (0.72, 84.38)	52.60 (1.25, 147.15)	39.11 (0.93, 109.41)	68.20 (1.62, 190.78)
Multiple Micronutrient Suppl.	50%	1.03 (0.54, 1.61)	2.26 (0.43, 5.44)	195 (37, 470)	12.01 (2.22, 30.31)	20.94 (3.88, 52.85)	28.02 (5.19, 70.73)	48.86 (9.05, 123.33)
	90%	2.60 (1.34, 4.31)	5.62 (1.15, 13.95)	485 (100, 1205)	29.77 (6.22, 80.23)	51.92 (10.84, 139.91)	38.60 (8.06, 104.03)	67.31 (14.06, 181.40)
Balanced Protein Suppl.	50%	0.12 (-0.10, 0.33)	0.23 (-0.22, 0.86)	19 (-19, 75)	1.20 (-1.17, 4.66)	2.10 (-2.04, 8.12)	26.51 (-27.15, 91.43)	46.24 (-47.34, 159.44)
	90%	0.21 (-0.18, 0.60)	0.41 (-0.40, 1.55)	35 (-35, 134)	2.16 (-2.10, 8.39)	3.77 (-3.67, 14.62)	26.51 (-27.15, 91.43)	46.24 (-47.34, 159.44)

References for Data Inputs

[1] Blencowe H, Krasevec J, Onis M De, et al. Articles National , regional , and worldwide estimates of low birthweight in 2015 , with trends from 2000: a systematic analysis. Lancet Glob Heal. 2019;(18):1-12.

[2] Chawanpaiboon S, Vogel JP, Moller AB, et al. Global, regional, and national estimates of levels of preterm birth in 2014: a systematic review and modelling analysis. Lancet Glob Heal. 2019;7(1):e37-e46.

[3] United National Population Division World Population Prospects 2019.

[4] Fink G, Peet E, Danaei G, et al. Schooling and wage income losses due to early-childhood growth faltering in developing countries: National, regional, and global estimates. Am J Clin Nutr. 2016;104(1):104-112.

[5] Country specific annual wage data from World Indicators Database. Average yearly wage was estimated to be 2/3 of the gross domestic product in 2010 constant US dollars and 2011 International dollars, adjusted for purchasing power parity.

[6] NCD Risk Factor Collaboration. Trends in adult body-mass index in 200 countries from 1975 to 2014: a pooled analysis of 1698 population-based measurement studies with 19·2 million participants. Lancet. 2016;387(10026):1377-1396.

[7] Stevens GA, Finucane MM, De-Regil LM, et al. Global, regional, and national trends in haemoglobin concentration and prevalence of total and severe anaemia in children and pregnant and non-pregnant women for 1995-2011: A systematic analysis of population-representative data. Lancet Glob Heal. 2013;1(1):16-25.

[8] Coverage of iron-folic acid supplementation abstracted from the most recent Demographic Health Survey or imputed based on sub-regional average. Indicator used: % women in the past five years who took iron tablets or syrup for >90 days.

Costa Rica

Region: Latin America and Caribbean**; Sub-region:** Central Latin America

Low birthweight prevalence[1]: 7.5% (95% CI: 7.4, 7.5)

Preterm birth prevalence[2]: 9.8% (95% CI: 8.6, 11.3)

Number of births[3]: 351,000

Returns to education[4]: 9.5% (95% CI: 7.5, 11.6)

GDP per capita 2010 US$ (estimated annual wage)[5]: $9219 ($6146/year)

GDP per capita 2011 International $ (estimated annual wage)[5]: $14617 ($9745/year)

Prevalence of low BMI[6]: 2.2% (95% CI: 0.9, 4.1)

Prevalence of anemia[7]: 26.3% (95% CI: 10.9, 49.1)

Baseline coverage of IFA[8]: 33.3%

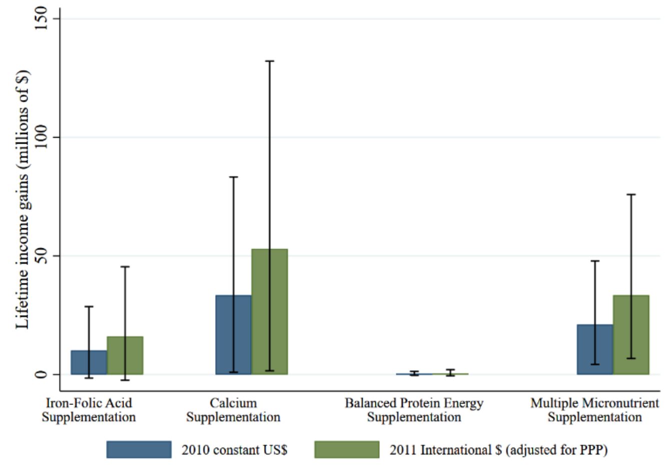

Figure: Benefits by birth cohort size for estimated gains in lifetime wages attributable to scaling up nutrition interventions to 90% target coverage

Table: Impact of maternal prenatal nutrition intervention on human capital and labour market outcomes, through improvements in low birthweight or preterm birth and schooling in Costa Rica.

Intervention	Target Coverage (%)	Absolute reduction in birth outcome (%)	Benefits by cohorts: School years gained (in 1000 s)	No. of additional students completing secondary school	Benefits by cohorts: Lifetime wages		Returns in lifetime earnings per child born to a targeted pregnant woman	
					in US $ millions	in International $ millions	in US $	in International $
Iron/Folic Acid Suppl.	90%	0.72 (-0.13, 1.46)	0.65 (-0.12, 1.77)	14 (-3, 39)	10.18 (-1.50, 28.65)	16.14 (-2.37, 45.42)	32.22 (-4.73, 90.68)	51.08 (-7.51, 143.77)
Calcium Suppl.	50%	1.16 (0.26, 2.01)	1.20 (0.04, 2.82)	26 (1, 62)	18.58 (0.56, 46.28)	29.46 (0.88, 73.38)	105.89 (3.17, 263.73)	167.89 (5.03, 418.14)
	90%	2.09 (0.48, 3.62)	2.16 (0.07, 5.08)	47 (1, 112)	33.45 (1.00, 83.31)	53.04 (1.59, 132.09)	105.89 (3.17, 263.73)	167.89 (5.03, 418.14)
Multiple Micronutrient Suppl.	50%	0.60 (0.30, 0.84)	0.57 (0.12, 1.22)	13 (3, 27)	9.04 (2.01, 19.76)	14.34 (3.19, 31.33)	51.53 (11.48, 112.60)	81.71 (18.20, 178.53)
	90%	1.42 (0.58, 2.17)	1.32 (0.26, 2.91)	29 (6, 64)	21.10 (4.28, 47.86)	33.45 (6.78, 75.88)	66.78 (13.54, 151.49)	105.88 (21.47, 240.19)
Balanced Protein Suppl.	50%	0.01 (-0.01, 0.04)	0.01 (-0.01, 0.04)	0 (-0, 1)	0.16 (-0.19, 0.72)	0.26 (-0.30, 1.14)	48.42 (-45.22, 162.04)	76.76 (-71.70, 256.91)
	90%	0.02 (-0.02, 0.07)	0.02 (-0.02, 0.08)	0 (-0, 2)	0.29 (-0.34, 1.30)	0.47 (-0.54, 2.06)	48.42 (-45.22, 162.04)	76.76 (-71.70, 256.91)

References for Data Inputs

[1] Blencowe H, Krasevec J, Onis M De, et al. Articles National , regional , and worldwide estimates of low birthweight in 2015 , with trends from 2000: a systematic analysis. Lancet Glob Heal. 2019;(18):1-12.

[2] Chawanpaiboon S, Vogel JP, Moller AB, et al. Global, regional, and national estimates of levels of preterm birth in 2014: a systematic review and modelling analysis. Lancet Glob Heal. 2019;7(1):e37-e46.

[3] United National Population Division World Population Prospects 2019.

[4] Fink G, Peet E, Danaei G, et al. Schooling and wage income losses due to early-childhood growth faltering in developing countries: National, regional, and global estimates. Am J Clin Nutr. 2016;104(1):104-112.

[5] Country specific annual wage data from World Indicators Database. Average yearly wage was estimated to be 2/3 of the gross domestic product in 2010 constant US dollars and 2011 International dollars, adjusted for purchasing power parity.

[6] NCD Risk Factor Collaboration. Trends in adult body-mass index in 200 countries from 1975 to 2014: a pooled analysis of 1698 population-based measurement studies with 19·2 million participants. Lancet. 2016;387(10026):1377-1396.

[7] Stevens GA, Finucane MM, De-Regil LM, et al. Global, regional, and national trends in haemoglobin concentration and prevalence of total and severe anaemia in children and pregnant and non-pregnant women for 1995-2011: A systematic analysis of population-representative data. Lancet Glob Heal. 2013;1(1):16-25.

[8] Coverage of iron-folic acid supplementation abstracted from the most recent Demographic Health Survey or imputed based on sub-regional average. Indicator used: % women in the past five years who took iron tablets or syrup for >90 days.

Cote d'Ivoire

Region: Sub-Saharan Africa**; Sub-region:** Western Sub-Saharan Africa

Low birthweight prevalence[1]: 15.5% (95% CI: 13.7, 22.4)

Preterm birth prevalence[2]: 12.0% (95% CI: 8.6, 16.7)

Number of births[3]: 4,451,000

Returns to education[4]: 6.8% (95% CI: 3.6, 10.1)

GDP per capita 2010 US$ (estimated annual wage)[5]: $1462 ($975/year)

GDP per capita 2011 International $ (estimated annual wage)[5]: $3225 ($2150/year)

Prevalence of low BMI[6]: 7.4% (95% CI: 3.7, 12.8)

Prevalence of anemia[7]: 57.0% (95% CI: 41.9, 68.6)

Baseline coverage of IFA[8]: 25.0%

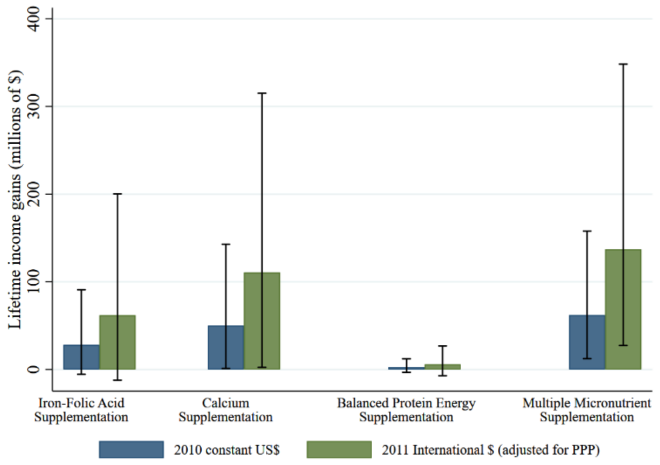

Figure: Benefits by birth cohort size for estimated gains in lifetime wages attributable to scaling up nutrition interventions to 90% target coverage

Table: Impact of maternal prenatal nutrition intervention on human capital and labour market outcomes, through improvements in low birthweight or preterm birth and schooling in Cote d'Ivoire.

Intervention	Target Coverage (%)	Absolute reduction in birth outcome (%)	Benefits by cohorts: School years gained (in 1000 s)	No. of additional students completing secondary school	Benefits by cohorts: Lifetime wages		Returns in lifetime earnings per child born to a targeted pregnant woman	
					in US $ millions	in International $ millions	in US $	in International $
Iron/Folic Acid Suppl.	90%	1.67 (-0.33, 3.55)	16.24 (-3.04, 46.20)	195 (-36, 554)	28.04 (-5.53, 90.82)	61.84 (-12.20, 200.31)	7.00 (-1.38, 22.67)	15.44 (-3.04, 50.00)
Calcium Suppl.	50%	1.41 (0.27, 2.59)	15.27 (0.49, 38.99)	183 (6, 468)	27.86 (0.60, 79.33)	61.45 (1.33, 174.96)	12.52 (0.27, 35.64)	27.61 (0.60, 78.62)
	90%	2.53 (0.49, 4.67)	27.49 (0.88, 70.19)	330 (11, 842)	50.15 (1.09, 142.79)	110.62 (2.40, 314.93)	12.52 (0.27, 35.64)	27.61 (0.60, 78.62)
Multiple Micronutrient Suppl.	50%	1.61 (0.88, 2.44)	16.86 (3.55, 38.02)	202 (43, 456)	29.87 (6.07, 72.99)	65.88 (13.39, 160.98)	13.42 (2.73, 32.80)	29.60 (6.02, 72.34)
	90%	3.43 (1.62, 5.35)	35.48 (7.79, 80.93)	426 (93, 971)	62.13 (12.36, 157.85)	137.03 (27.27, 348.14)	15.51 (3.09, 39.40)	34.21 (6.81, 86.91)
Balanced Protein Suppl.	50%	0.09 (-0.09, 0.27)	0.83 (-0.81, 3.36)	10 (-10, 40)	1.48 (-1.79, 6.76)	3.27 (-3.94, 14.91)	9.82 (-9.79, 34.13)	21.65 (-21.58, 75.28)
	90%	0.16 (-0.15, 0.49)	1.50 (-1.45, 6.05)	18 (-17, 73)	2.67 (-3.22, 12.17)	5.89 (-7.10, 26.85)	9.82 (-9.79, 34.13)	21.65 (-21.58, 75.28)

References for Data Inputs

[1] Blencowe H, Krasevec J, Onis M De, et al. Articles National , regional , and worldwide estimates of low birthweight in 2015 , with trends from 2000: a systematic analysis. Lancet Glob Heal. 2019;(18):1-12.
[2] Chawanpaiboon S, Vogel JP, Moller AB, et al. Global, regional, and national estimates of levels of preterm birth in 2014: a systematic review and modelling analysis. Lancet Glob Heal. 2019;7(1):e37-e46.
[3] United National Population Division World Population Prospects 2019.
[4] Fink G, Peet E, Danaei G, et al. Schooling and wage income losses due to early-childhood growth faltering in developing countries: National, regional, and global estimates. Am J Clin Nutr. 2016;104(1):104-112.
[5] Country specific annual wage data from World Indicators Database. Average yearly wage was estimated to be 2/3 of the gross domestic product in 2010 constant US dollars and 2011 International dollars, adjusted for purchasing power parity.
[6] NCD Risk Factor Collaboration. Trends in adult body-mass index in 200 countries from 1975 to 2014: a pooled analysis of 1698 population-based measurement studies with 19·2 million participants. Lancet. 2016;387(10026):1377-1396.
[7] Stevens GA, Finucane MM, De-Regil LM, et al. Global, regional, and national trends in haemoglobin concentration and prevalence of total and severe anaemia in children and pregnant and non-pregnant women for 1995-2011: A systematic analysis of population-representative data. Lancet Glob Heal. 2013;1(1):16-25.
[8] Coverage of iron-folic acid supplementation abstracted from the most recent Demographic Health Survey or imputed based on sub-regional average. Indicator used: % women in the past five years who took iron tablets or syrup for >90 days.

Cuba

Region: Latin America and Caribbean**; Sub-region:** Caribbean

Low birthweight prevalence[1]: 5.3% (95% CI: 5.2, 5.4)

Preterm birth prevalence[2]: 6.0% (95% CI: 2.0, 10.1)

Number of births[3]: 579,000

Returns to education[4]: 9.8% (95% CI: 8.1, 11.5)

GDP per capita 2010 US$ (estimated annual wage)[5]: $6523 ($4348/year)

GDP per capita 2011 International $ (estimated annual wage)[5]: $18083 ($12055/year)

Prevalence of low BMI[6]: 4.4% (95% CI: 2.1, 7.7)

Prevalence of anemia[7]: 28.1% (95% CI: 12.7, 51.0)

Baseline coverage of IFA[8]: 62.3%

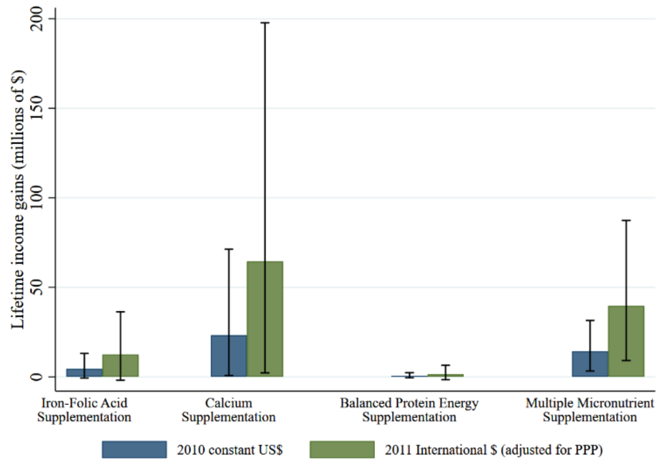

Figure: Benefits by birth cohort size for estimated gains in lifetime wages attributable to scaling up nutrition interventions to 90% target coverage

Table: Impact of maternal prenatal nutrition intervention on human capital and labour market outcomes, through improvements in low birthweight or preterm birth and schooling in Cuba.

Intervention	Target Coverage (%)	Absolute reduction in birth outcome (%)	Benefits by cohorts: School years gained (in 1000 s)	No. of additional students completing secondary school	Benefits by cohorts: Lifetime wages		Returns in lifetime earnings per child born to a targeted pregnant woman	
					in US $ millions	in International $ millions	in US $	in International $
Iron/Folic Acid Suppl.	90%	0.26 (-0.05, 0.57)	0.40 (-0.07, 1.11)	21 (-3, 58)	4.51 (-0.66, 13.10)	12.49 (-1.84, 36.31)	8.65 (-1.27, 25.13)	23.97 (-3.53, 69.68)
Calcium Suppl.	50%	0.67 (0.09, 1.57)	1.10 (0.04, 3.43)	57 (2, 178)	12.92 (0.44, 39.62)	35.83 (1.23, 109.83)	44.64 (1.53, 136.85)	123.76 (4.24, 379.38)
	90%	1.21 (0.16, 2.82)	1.98 (0.06, 6.17)	103 (3, 320)	23.26 (0.80, 71.31)	64.49 (2.21, 197.70)	44.64 (1.53, 136.85)	123.76 (4.24, 379.38)
Multiple Micronutrient Suppl.	50%	0.32 (0.15, 0.46)	0.50 (0.10, 1.11)	26 (5, 58)	5.76 (1.14, 13.24)	15.97 (3.17, 36.71)	19.90 (3.95, 45.74)	55.16 (10.96, 126.81)
	90%	0.78 (0.41, 1.12)	1.24 (0.28, 2.72)	64 (14, 141)	14.31 (3.29, 31.51)	39.67 (9.11, 87.36)	27.46 (6.30, 60.47)	76.13 (17.48, 167.65)
Balanced Protein Suppl.	50%	0.02 (-0.02, 0.06)	0.03 (-0.03, 0.11)	1 (-2, 5)	0.29 (-0.32, 1.29)	0.81 (-0.88, 3.58)	25.85 (-24.66, 80.12)	71.66 (-68.37, 222.11)
	90%	0.03 (-0.03, 0.10)	0.05 (-0.05, 0.19)	2 (-3, 10)	0.52 (-0.57, 2.32)	1.45 (-1.58, 6.44)	25.85 (-24.66, 80.12)	71.66 (-68.37, 222.11)

References for Data Inputs

[1] Blencowe H, Krasevec J, Onis M De, et al. Articles National , regional , and worldwide estimates of low birthweight in 2015 , with trends from 2000: a systematic analysis. Lancet Glob Heal. 2019;(18):1-12.
[2] Chawanpaiboon S, Vogel JP, Moller AB, et al. Global, regional, and national estimates of levels of preterm birth in 2014: a systematic review and modelling analysis. Lancet Glob Heal. 2019;7(1):e37-e46.
[3] United National Population Division World Population Prospects 2019.
[4] Fink G, Peet E, Danaei G, et al. Schooling and wage income losses due to early-childhood growth faltering in developing countries: National, regional, and global estimates. Am J Clin Nutr. 2016;104(1):104-112.
[5] Country specific annual wage data from World Indicators Database. Average yearly wage was estimated to be 2/3 of the gross domestic product in 2010 constant US dollars and 2011 International dollars, adjusted for purchasing power parity.
[6] NCD Risk Factor Collaboration. Trends in adult body-mass index in 200 countries from 1975 to 2014: a pooled analysis of 1698 population-based measurement studies with 19·2 million participants. Lancet. 2016;387(10026):1377-1396.
[7] Stevens GA, Finucane MM, De-Regil LM, et al. Global, regional, and national trends in haemoglobin concentration and prevalence of total and severe anaemia in children and pregnant and non-pregnant women for 1995-2011: A systematic analysis of population-representative data. Lancet Glob Heal. 2013;1(1):16-25.
[8] Coverage of iron-folic acid supplementation abstracted from the most recent Demographic Health Survey or imputed based on sub-regional average. Indicator used: % women in the past five years who took iron tablets or syrup for >90 days.

Democratic Republic of Congo

Region: Sub-Saharan Africa**; Sub-region:** Central Sub-Saharan Africa

Low birthweight prevalence[1]: 10.8% (95% CI: 8.5, 15.1)

Preterm birth prevalence[2]: 9.8% (95% CI: 7.1, 13.2)

Number of births[3]: 17,169,000

Returns to education[4]: 9.9% (95% CI: 7.0, 12.9)

GDP per capita 2010 US$ (estimated annual wage)[5]: $411 ($274/year)

GDP per capita 2011 International $ (estimated annual wage)[5]: $812 ($541/year)

Prevalence of low BMI[6]: 13.1% (95% CI: 6.7, 21.0)

Prevalence of anemia[7]: 50.1% (95% CI: 36.6, 62.0)

Baseline coverage of IFA[8]: 4.7%

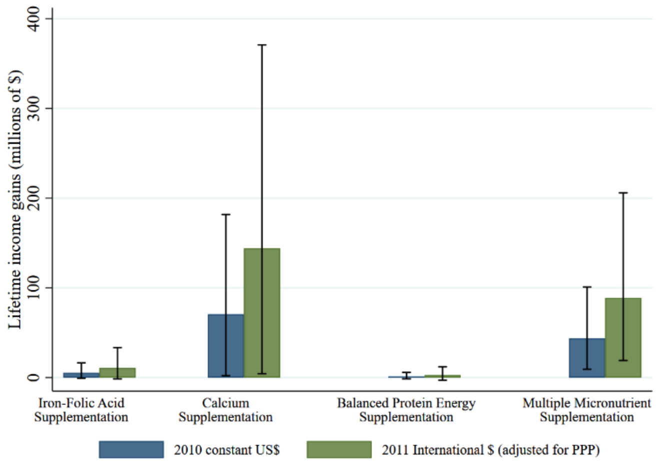

Figure: Benefits by birth cohort size for estimated gains in lifetime wages attributable to scaling up nutrition interventions to 90% target coverage

Table: Impact of maternal prenatal nutrition intervention on human capital and labour market outcomes, through improvements in low birthweight or preterm birth and schooling in Democratic Republic of Congo.

Intervention	Target Coverage (%)	Absolute reduction in birth outcome (%)	Benefits by cohorts: School years gained (in 1000 s)	No. of additional students completing secondary school	Benefits by cohorts: Lifetime wages		Returns in lifetime earnings per child born to a targeted pregnant woman	
					in US $ millions	in International $ millions	in US $	in International $
**Iron/Folic Acid Suppl.**	90%	1.43 (-0.26, 3.11)	55.15 (-10.74, 153.32)	982 (-191, 2729)	39.11 (-7.07, 119.03)	77.25 (-13.96, 235.13)	2.53 (-0.46, 7.70)	5.00 (-0.90, 15.22)
**Calcium Suppl.**	50%	1.16 (0.24, 2.16)	49.57 (1.26, 122.70)	882 (22, 2184)	34.45 (0.85, 96.18)	68.04 (1.68, 189.99)	4.01 (0.10, 11.20)	7.93 (0.20, 22.13)
	90%	2.08 (0.44, 3.88)	89.23 (2.26, 220.86)	1588 (40, 3931)	62.00 (1.53, 173.12)	122.47 (3.02, 341.97)	4.01 (0.10, 11.20)	7.93 (0.20, 22.13)
**Multiple Micronutrient Suppl.**	50%	1.41 (0.56, 2.32)	54.45 (9.97, 126.46)	969 (178, 2251)	38.67 (6.79, 98.94)	76.39 (13.42, 195.44)	4.50 (0.79, 11.53)	8.90 (1.56, 22.77)
	90%	2.60 (1.02, 4.30)	100.75 (17.91, 234.10)	1793 (319, 4167)	71.41 (12.36, 183.20)	141.06 (24.42, 361.88)	4.62 (0.80, 11.86)	9.13 (1.58, 23.42)
**Balanced Protein Suppl.**	50%	0.11 (-0.09, 0.32)	3.96 (-4.34, 14.49)	71 (-77, 258)	2.78 (-2.81, 11.15)	5.49 (-5.55, 22.02)	2.68 (-2.60, 9.03)	5.29 (-5.14, 17.84)
	90%	0.20 (-0.17, 0.58)	7.14 (-7.82, 26.09)	127 (-139, 464)	5.00 (-5.06, 20.07)	9.88 (-9.99, 39.64)	2.68 (-2.60, 9.03)	5.29 (-5.14, 17.84)

References for Data Inputs
[1] Blencowe H, Krasevec J, Onis M De, et al. Articles National , regional , and worldwide estimates of low birthweight in 2015 , with trends from 2000: a systematic analysis. Lancet Glob Heal. 2019;(18):1-12.
[2] Chawanpaiboon S, Vogel JP, Moller AB, et al. Global, regional, and national estimates of levels of preterm birth in 2014: a systematic review and modelling analysis. Lancet Glob Heal. 2019;7(1):e37-e46.
[3] United National Population Division World Population Prospects 2019.
[4] Fink G, Peet E, Danaei G, et al. Schooling and wage income losses due to early-childhood growth faltering in developing countries: National, regional, and global estimates. Am J Clin Nutr. 2016;104(1):104-112.
[5] Country specific annual wage data from World Indicators Database. Average yearly wage was estimated to be 2/3 of the gross domestic product in 2010 constant US dollars and 2011 International dollars, adjusted for purchasing power parity.
[6] NCD Risk Factor Collaboration. Trends in adult body-mass index in 200 countries from 1975 to 2014: a pooled analysis of 1698 population-based measurement studies with 19·2 million participants. Lancet. 2016;387(10026):1377-1396.
[7] Stevens GA, Finucane MM, De-Regil LM, et al. Global, regional, and national trends in haemoglobin concentration and prevalence of total and severe anaemia in children and pregnant and non-pregnant women for 1995-2011: A systematic analysis of population-representative data. Lancet Glob Heal. 2013;1(1):16-25.
[8] Coverage of iron-folic acid supplementation abstracted from the most recent Demographic Health Survey or imputed based on sub-regional average. Indicator used: % women in the past five years who took iron tablets or syrup for >90 days.

People's Republic of Korea

Region: Southeast Asia, East Asia, and Oceania**; Sub-region:** East Asia

Low birthweight prevalence[1]: 6.7% (95% CI: 5.5, 8.0)

Preterm birth prevalence[2]: 10.4% (95% CI: 8.7, 11.9)

Number of births[3]: 1,776,000

Returns to education[4]: 4.1% (95% CI: 3.3, 5.0)

GDP per capita 2010 US$ (estimated annual wage)[5]: $523 ($349/year)

GDP per capita 2011 International $ (estimated annual wage)[5]: $1559 ($1039/year)

Prevalence of low BMI[6]: 6.0% (95% CI: 4.0, 9.0)

Prevalence of anemia[7]: 27.4% (95% CI: 15.9, 45.7)

Baseline coverage of IFA[8]: 44.2%

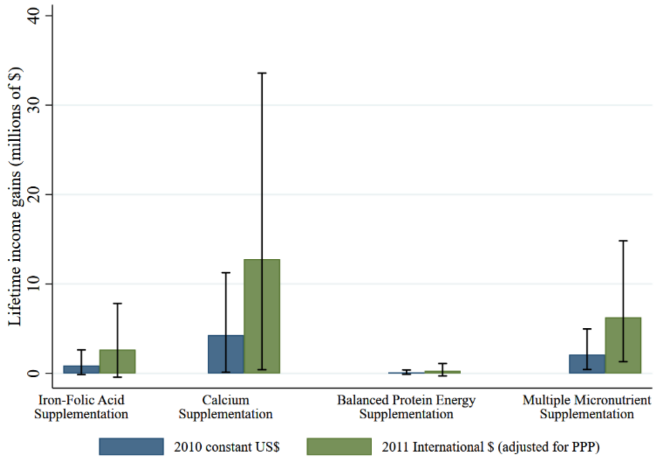

Figure: Benefits by birth cohort size for estimated gains in lifetime wages attributable to scaling up nutrition interventions to 90% target coverage

Table: Impact of maternal prenatal nutrition intervention on human capital and labour market outcomes, through improvements in low birthweight or preterm birth and schooling in People's Republic of Korea.

Intervention	Target Coverage (%)	Absolute reduction in birth outcome (%)	Benefits by cohorts: School years gained (in 1000 s)	No. of additional students completing secondary school	Benefits by cohorts: Lifetime wages		Returns in lifetime earnings per child born to a targeted pregnant woman	
					in US $ millions	in International $ millions	in US $	in International $
**Iron/Folic Acid Suppl.**	90%	0.53 (-0.09, 1.12)	2.35 (-0.41, 6.61)	219 (-38, 616)	0.89 (-0.14, 2.62)	2.65 (-0.42, 7.83)	0.56 (-0.09, 1.64)	1.66 (-0.26, 4.90)
**Calcium Suppl.**	50%	1.24 (0.29, 2.10)	6.14 (0.19, 15.13)	572 (18, 1410)	2.38 (0.08, 6.26)	7.09 (0.23, 18.66)	2.68 (0.09, 7.05)	7.99 (0.26, 21.01)
	90%	2.23 (0.52, 3.78)	11.06 (0.35, 27.24)	1030 (33, 2539)	4.28 (0.14, 11.26)	12.77 (0.41, 33.59)	2.68 (0.09, 7.05)	7.99 (0.26, 21.01)
**Multiple Micronutrient Suppl.**	50%	0.42 (0.23, 0.61)	2.01 (0.44, 4.48)	187 (41, 417)	0.77 (0.16, 1.71)	2.30 (0.48, 5.11)	0.87 (0.18, 1.93)	2.60 (0.54, 5.75)
	90%	1.15 (0.53, 1.81)	5.41 (1.21, 12.48)	504 (113, 1163)	2.10 (0.44, 4.97)	6.27 (1.32, 14.83)	1.31 (0.28, 3.11)	3.92 (0.82, 9.28)
**Balanced Protein Suppl.**	50%	0.03 (-0.03, 0.09)	0.14 (-0.14, 0.50)	13 (-13, 47)	0.05 (-0.05, 0.21)	0.16 (-0.16, 0.61)	1.08 (-1.02, 3.37)	3.23 (-3.03, 10.05)
	90%	0.06 (-0.05, 0.16)	0.25 (-0.26, 0.91)	24 (-24, 85)	0.10 (-0.10, 0.37)	0.28 (-0.29, 1.11)	1.08 (-1.02, 3.37)	3.23 (-3.03, 10.05)

References for Data Inputs

[1] Blencowe H, Krasevec J, Onis M De, et al. Articles National , regional , and worldwide estimates of low birthweight in 2015 , with trends from 2000: a systematic analysis. Lancet Glob Heal. 2019;(18):1-12.
[2] Chawanpaiboon S, Vogel JP, Moller AB, et al. Global, regional, and national estimates of levels of preterm birth in 2014: a systematic review and modelling analysis. Lancet Glob Heal. 2019;7(1):e37-e46.
[3] United National Population Division World Population Prospects 2019.
[4] Fink G, Peet E, Danaei G, et al. Schooling and wage income losses due to early-childhood growth faltering in developing countries: National, regional, and global estimates. Am J Clin Nutr. 2016;104(1):104-112.
[5] Country specific annual wage data from World Indicators Database. Average yearly wage was estimated to be 2/3 of the gross domestic product in 2010 constant US dollars and 2011 International dollars, adjusted for purchasing power parity.
[6] NCD Risk Factor Collaboration. Trends in adult body-mass index in 200 countries from 1975 to 2014: a pooled analysis of 1698 population-based measurement studies with 19·2 million participants. Lancet. 2016;387(10026):1377-1396.
[7] Stevens GA, Finucane MM, De-Regil LM, et al. Global, regional, and national trends in haemoglobin concentration and prevalence of total and severe anaemia in children and pregnant and non-pregnant women for 1995-2011: A systematic analysis of population-representative data. Lancet Glob Heal. 2013;1(1):16-25.
[8] Coverage of iron-folic acid supplementation abstracted from the most recent Demographic Health Survey or imputed based on sub-regional average. Indicator used: % women in the past five years who took iron tablets or syrup for >90 days.

Djibouti

Region: Sub-Saharan Africa**; Sub-region:** Eastern Sub-Saharan Africa

Low birthweight prevalence[1]: 20.0% (95% CI: 14.4, 25.6)

Preterm birth prevalence[2]: 12.0% (95% CI: 8.6, 16.7)

Number of births[3]: 103,000

Returns to education[4]: 11.3% (95% CI: 9.7, 12.9)

GDP per capita 2010 US$ (estimated annual wage)[5]: $1343 ($896/year)

GDP per capita 2011 International $ (estimated annual wage)[5]: $10215 ($6810/year)

Prevalence of low BMI[6]: 7.1% (95% CI: 2.5, 14.7)

Prevalence of anemia[7]: 32.5% (95% CI: 15.5, 54.6)

Baseline coverage of IFA[8]: 19.2%

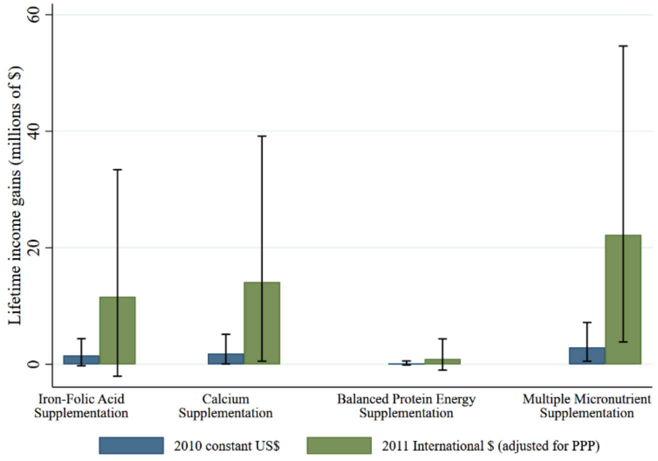

Figure: Benefits by birth cohort size for estimated gains in lifetime wages attributable to scaling up nutrition interventions to 90% target coverage

Table: Impact of maternal prenatal nutrition intervention on human capital and labour market outcomes, through improvements in low birthweight or preterm birth and schooling in Djibouti.

Intervention	Target Coverage (%)	Absolute reduction in birth outcome (%)	Benefits by cohorts: School years gained (in 1000 s)	No. of additional students completing secondary school	Benefits by cohorts: Lifetime wages		Returns in lifetime earnings per child born to a targeted pregnant woman	
					in US $ millions	in International $ millions	in US $	in International $
**Iron/Folic Acid Suppl.**	90%	2.35 (-0.42, 4.97)	0.57 (-0.10, 1.59)	16 (-3, 44)	1.52 (-0.27, 4.39)	11.60 (-2.04, 33.40)	16.45 (-2.89, 47.38)	125.09 (-21.96, 360.33)
**Calcium Suppl.**	50%	1.39 (0.28, 2.59)	0.38 (0.01, 1.02)	10 (0, 28)	1.03 (0.04, 2.86)	7.84 (0.29, 21.76)	20.02 (0.74, 55.56)	152.27 (5.61, 422.53)
	90%	2.50 (0.51, 4.65)	0.68 (0.03, 1.83)	19 (1, 50)	1.86 (0.07, 5.15)	14.12 (0.52, 39.17)	20.02 (0.74, 55.56)	152.27 (5.61, 422.53)
**Multiple Micronutrient Suppl.**	50%	2.06 (0.94, 3.29)	0.53 (0.10, 1.23)	15 (3, 34)	1.43 (0.27, 3.38)	10.88 (2.04, 25.71)	27.78 (5.21, 65.63)	211.25 (39.61, 499.13)
	90%	4.22 (1.64, 6.88)	1.07 (0.18, 2.54)	29 (5, 70)	2.92 (0.51, 7.18)	22.21 (3.85, 54.62)	31.50 (5.46, 77.48)	239.56 (41.49, 589.24)
**Balanced Protein Suppl.**	50%	0.11 (-0.09, 0.40)	0.02 (-0.03, 0.12)	1 (-1, 3)	0.07 (-0.07, 0.32)	0.51 (-0.55, 2.42)	21.68 (-21.26, 69.51)	164.84 (-161.68, 528.63)
	90%	0.19 (-0.16, 0.72)	0.04 (-0.05, 0.22)	1 (-1, 6)	0.12 (-0.13, 0.57)	0.92 (-0.99, 4.36)	21.68 (-21.26, 69.51)	164.84 (-161.68, 528.63)

References for Data Inputs

[1] Blencowe H, Krasevec J, Onis M De, et al. Articles National , regional , and worldwide estimates of low birthweight in 2015 , with trends from 2000: a systematic analysis. Lancet Glob Heal. 2019;(18):1-12.
[2] Chawanpaiboon S, Vogel JP, Moller AB, et al. Global, regional, and national estimates of levels of preterm birth in 2014: a systematic review and modelling analysis. Lancet Glob Heal. 2019;7(1):e37-e46.
[3] United National Population Division World Population Prospects 2019.
[4] Fink G, Peet E, Danaei G, et al. Schooling and wage income losses due to early-childhood growth faltering in developing countries: National, regional, and global estimates. Am J Clin Nutr. 2016;104(1):104-112.
[5] Country specific annual wage data from World Indicators Database. Average yearly wage was estimated to be 2/3 of the gross domestic product in 2010 constant US dollars and 2011 International dollars, adjusted for purchasing power parity.
[6] NCD Risk Factor Collaboration. Trends in adult body-mass index in 200 countries from 1975 to 2014: a pooled analysis of 1698 population-based measurement studies with 19·2 million participants. Lancet. 2016;387(10026):1377-1396.
[7] Stevens GA, Finucane MM, De-Regil LM, et al. Global, regional, and national trends in haemoglobin concentration and prevalence of total and severe anaemia in children and pregnant and non-pregnant women for 1995-2011: A systematic analysis of population-representative data. Lancet Glob Heal. 2013;1(1):16-25.
[8] Coverage of iron-folic acid supplementation abstracted from the most recent Demographic Health Survey or imputed based on sub-regional average. Indicator used: % women in the past five years who took iron tablets or syrup for >90 days.

Dominican Republic

Region: Latin America and Caribbean**; Sub-region:** Caribbean

Low birthweight prevalence[1]: 11.3% (95% CI: 8.7, 15.0)

Preterm birth prevalence[2]: 9.8% (95% CI: 8.6, 11.3)

Number of births[3]: 1,040,000

Returns to education[4]: 9.8% (95% CI: 8.1, 11.5)

GDP per capita 2010 US$ (estimated annual wage)[5]: $6720 ($4480/year)

GDP per capita 2011 International $ (estimated annual wage)[5]: $13717 ($9145/year)

Prevalence of low BMI[6]: 3.1% (95% CI: 1.3, 5.9)

Prevalence of anemia[7]: 32.2% (95% CI: 15.1, 56.8)

Baseline coverage of IFA[8]: 81.5%

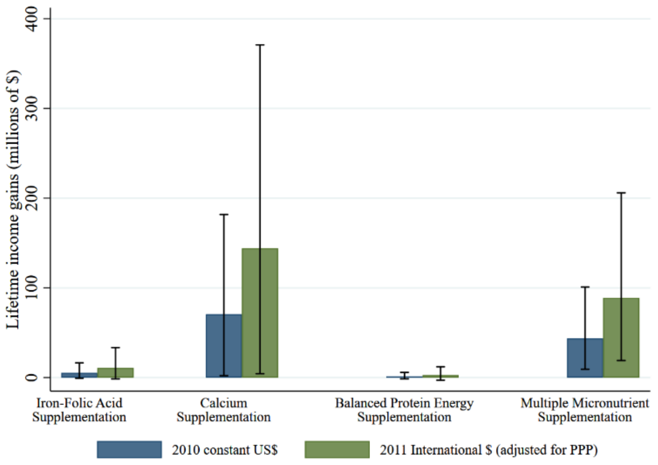

Figure: Benefits by birth cohort size for estimated gains in lifetime wages attributable to scaling up nutrition interventions to 90% target coverage

Table: Impact of maternal prenatal nutrition intervention on human capital and labour market outcomes, through improvements in low birthweight or preterm birth and schooling in Dominican Republic.

Intervention	Target Coverage (%)	Absolute reduction in birth outcome (%)	Benefits by cohorts: School years gained (in 1000 s)	No. of additional students completing secondary school	Benefits by cohorts: Lifetime wages		Returns in lifetime earnings per child born to a targeted pregnant woman	
					in US $ millions	in International $ millions	in US $	in International $
**Iron/Folic Acid Suppl.**	90%	0.18 (-0.03, 0.42)	0.44 (-0.07, 1.33)	24 (-4, 71)	5.27 (-0.74, 16.40)	10.76 (-1.50, 33.47)	5.63 (-0.79, 17.52)	11.50 (-1.61, 35.76)
**Calcium Suppl.**	50%	1.17 (0.27, 1.96)	3.38 (0.10, 8.13)	180 (5, 434)	39.20 (1.19, 100.96)	80.01 (2.42, 206.07)	75.38 (2.28, 194.15)	153.86 (4.65, 396.30)
	90%	2.11 (0.49, 3.52)	6.08 (0.17, 14.63)	325 (9, 781)	70.55 (2.13, 181.72)	144.01 (4.36, 370.93)	75.38 (2.28, 194.15)	153.86 (4.65, 396.30)
**Multiple Micronutrient Suppl.**	50%	0.66 (0.33, 1.08)	1.86 (0.38, 4.37)	99 (20, 233)	21.95 (4.40, 52.98)	44.81 (8.99, 108.14)	42.22 (8.47, 101.88)	86.18 (17.29, 207.96)
	90%	1.32 (0.72, 2.11)	3.73 (0.76, 8.41)	199 (41, 449)	43.60 (9.30, 100.89)	88.99 (18.99, 205.93)	46.58 (9.94, 107.79)	95.08 (20.29, 220.01)
**Balanced Protein Suppl.**	50%	0.03 (-0.02, 0.09)	0.06 (-0.07, 0.27)	3 (-3, 15)	0.76 (-0.78, 3.25)	1.55 (-1.60, 6.64)	52.90 (-45.55, 166.33)	107.98 (-92.97, 339.51)
	90%	0.05 (-0.04, 0.16)	0.12 (-0.12, 0.49)	6 (-6, 26)	1.37 (-1.41, 5.86)	2.79 (-2.88, 11.96)	52.90 (-45.55, 166.33)	107.98 (-92.97, 339.51)

References for Data Inputs

[1] Blencowe H, Krasevec J, Onis M De, et al. Articles National , regional , and worldwide estimates of low birthweight in 2015 , with trends from 2000: a systematic analysis. Lancet Glob Heal. 2019;(18):1-12.

[2] Chawanpaiboon S, Vogel JP, Moller AB, et al. Global, regional, and national estimates of levels of preterm birth in 2014: a systematic review and modelling analysis. Lancet Glob Heal. 2019;7(1):e37-e46.

[3] United National Population Division World Population Prospects 2019.

[4] Fink G, Peet E, Danaei G, et al. Schooling and wage income losses due to early-childhood growth faltering in developing countries: National, regional, and global estimates. Am J Clin Nutr. 2016;104(1):104-112.

[5] Country specific annual wage data from World Indicators Database. Average yearly wage was estimated to be 2/3 of the gross domestic product in 2010 constant US dollars and 2011 International dollars, adjusted for purchasing power parity.

[6] NCD Risk Factor Collaboration. Trends in adult body-mass index in 200 countries from 1975 to 2014: a pooled analysis of 1698 population-based measurement studies with 19·2 million participants. Lancet. 2016;387(10026):1377-1396.

[7] Stevens GA, Finucane MM, De-Regil LM, et al. Global, regional, and national trends in haemoglobin concentration and prevalence of total and severe anaemia in children and pregnant and non-pregnant women for 1995-2011: A systematic analysis of population-representative data. Lancet Glob Heal. 2013;1(1):16-25.

[8] Coverage of iron-folic acid supplementation abstracted from the most recent Demographic Health Survey or imputed based on sub-regional average. Indicator used: % women in the past five years who took iron tablets or syrup for >90 days.

Ecuador

Region: Latin America and Caribbean**; Sub-region:** Andean Latin America

Low birthweight prevalence[1]: 11.2% (95% CI: 8.7, 14.2)

Preterm birth prevalence[2]: 8.0% (95% CI: 4.3, 13.3)

Number of births[3]: 1,681,000

Returns to education[4]: 6.7% (95% CI: 5.5, 8.0)

GDP per capita 2010 US$ (estimated annual wage)[5]: $5331 ($3554/year)

GDP per capita 2011 International $ (estimated annual wage)[5]: $10704 ($7136/year)

Prevalence of low BMI[6]: 1.2% (95% CI: 0.4, 2.8)

Prevalence of anemia[7]: 29.8% (95% CI: 13.4, 51.6)

Baseline coverage of IFA[8]: 44.7%

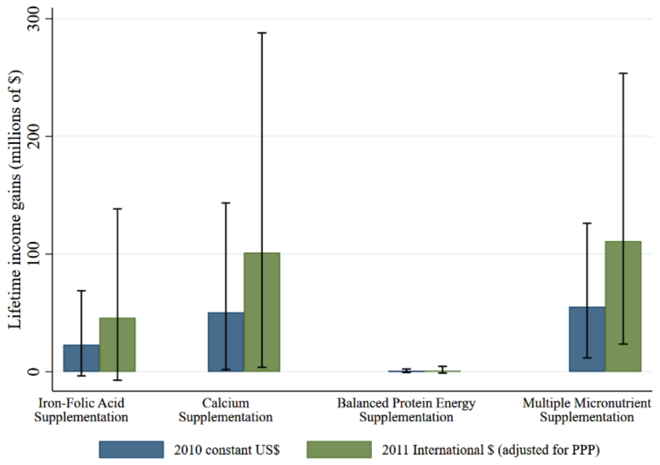

Figure: Benefits by birth cohort size for estimated gains in lifetime wages attributable to scaling up nutrition interventions to 90% target coverage

Table: Impact of maternal prenatal nutrition intervention on human capital and labour market outcomes, through improvements in low birthweight or preterm birth and schooling in Ecuador.

Intervention	Target Coverage (%)	Absolute reduction in birth outcome (%)	Benefits by cohorts: School years gained (in 1000 s)	No. of additional students completing secondary school	Benefits by cohorts: Lifetime wages		Returns in lifetime earnings per child born to a targeted pregnant woman	
					in US $ millions	in International $ millions	in US $	in International $
**Iron/Folic Acid Suppl.**	90%	0.86 (-0.14, 1.89)	3.65 (-0.51, 10.57)	48 (-7, 140)	22.88 (-3.56, 68.89)	45.94 (-7.16, 138.34)	15.12 (-2.36, 45.54)	30.37 (-4.73, 91.44)
**Calcium Suppl.**	50%	0.91 (0.16, 1.92)	4.31 (0.15, 12.48)	57 (2, 165)	27.99 (1.03, 79.68)	56.21 (2.08, 160.00)	33.30 (1.23, 94.80)	66.88 (2.47, 190.37)
	90%	1.64 (0.30, 3.45)	7.77 (0.27, 22.46)	103 (4, 297)	50.39 (1.86, 143.42)	101.18 (3.74, 288.00)	33.30 (1.23, 94.80)	66.88 (2.47, 190.37)
**Multiple Micronutrient Suppl.**	50%	0.70 (0.38, 1.06)	3.25 (0.68, 6.92)	43 (9, 91)	20.74 (4.64, 46.17)	41.65 (9.31, 92.71)	24.68 (5.52, 54.93)	49.55 (11.07, 110.31)
	90%	1.97 (0.89, 3.09)	8.70 (1.86, 19.58)	115 (25, 258)	55.32 (11.70, 126.25)	111.08 (23.50, 253.52)	36.56 (7.73, 83.45)	73.42 (15.53, 167.57)
**Balanced Protein Suppl.**	50%	0.01 (-0.01, 0.04)	0.04 (-0.05, 0.19)	1 (-1, 3)	0.25 (-0.33, 1.28)	0.50 (-0.65, 2.56)	28.63 (-28.05, 93.73)	57.50 (-56.32, 188.21)
	90%	0.02 (-0.02, 0.07)	0.07 (-0.09, 0.35)	1 (-1, 5)	0.45 (-0.59, 2.30)	0.90 (-1.18, 4.61)	28.63 (-28.05, 93.73)	57.50 (-56.32, 188.21)

References for Data Inputs

[1] Blencowe H, Krasevec J, Onis M De, et al. Articles National , regional , and worldwide estimates of low birthweight in 2015 , with trends from 2000: a systematic analysis. Lancet Glob Heal. 2019;(18):1-12.

[2] Chawanpaiboon S, Vogel JP, Moller AB, et al. Global, regional, and national estimates of levels of preterm birth in 2014: a systematic review and modelling analysis. Lancet Glob Heal. 2019;7(1):e37-e46.

[3] United National Population Division World Population Prospects 2019.

[4] Fink G, Peet E, Danaei G, et al. Schooling and wage income losses due to early-childhood growth faltering in developing countries: National, regional, and global estimates. Am J Clin Nutr. 2016;104(1):104-112.

[5] Country specific annual wage data from World Indicators Database. Average yearly wage was estimated to be 2/3 of the gross domestic product in 2010 constant US dollars and 2011 International dollars, adjusted for purchasing power parity.

[6] NCD Risk Factor Collaboration. Trends in adult body-mass index in 200 countries from 1975 to 2014: a pooled analysis of 1698 population-based measurement studies with 19·2 million participants. Lancet. 2016;387(10026):1377-1396.

[7] Stevens GA, Finucane MM, De-Regil LM, et al. Global, regional, and national trends in haemoglobin concentration and prevalence of total and severe anaemia in children and pregnant and non-pregnant women for 1995-2011: A systematic analysis of population-representative data. Lancet Glob Heal. 2013;1(1):16-25.

[8] Coverage of iron-folic acid supplementation abstracted from the most recent Demographic Health Survey or imputed based on sub-regional average. Indicator used: % women in the past five years who took iron tablets or syrup for >90 days.

Egypt

Region: North Africa and Middle East**; Sub-region:** North Africa and Middle East

Low birthweight prevalence[1]: 6.0% (95% CI: 2.0, 10.0)

Preterm birth prevalence[2]: 13.4% (95% CI: 6.3, 30.9)

Number of births[3]: 12,922,000

Returns to education[4]: 5.2% (95% CI: 4.8, 5.5)

GDP per capita 2010 US$ (estimated annual wage)[5]: $2704 ($1802/year)

GDP per capita 2011 International $ (estimated annual wage)[5]: $10243 ($6828/year)

Prevalence of low BMI[6]: 1.0% (95% CI: 0.5, 1.8)

Prevalence of anemia[7]: 30.4% (95% CI: 19.7, 45.6)

Baseline coverage of IFA[8]: 36.1%

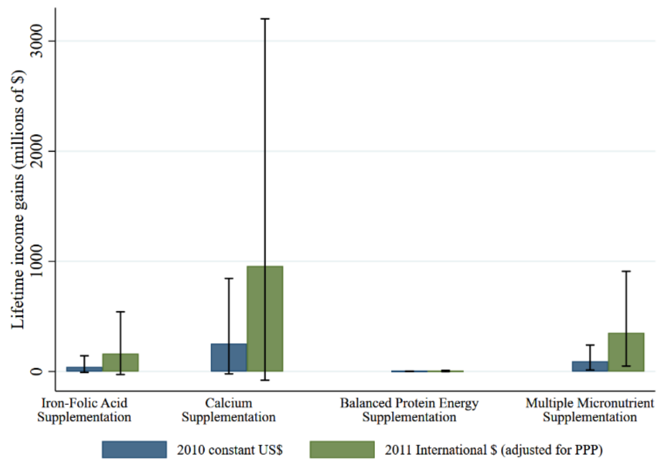

Figure: Benefits by birth cohort size for estimated gains in lifetime wages attributable to scaling up nutrition interventions to 90% target coverage

Table: Impact of maternal prenatal nutrition intervention on human capital and labour market outcomes, through improvements in low birthweight or preterm birth and schooling in Egypt.

Intervention	Target Coverage (%)	Absolute reduction in birth outcome (%)	Benefits by cohorts: School years gained (in 1000 s)	No. of additional students completing secondary school	Benefits by cohorts: Lifetime wages		Returns in lifetime earnings per child born to a targeted pregnant woman	
					in US $ millions	in International $ millions	in US $	in International $
Iron/Folic Acid Suppl.	90%	0.51 (-0.09, 1.39)	17.02 (-2.96, 56.43)	807 (-140, 2675)	42.99 (-7.35, 143.02)	162.85 (-27.84, 541.80)	3.70 (-0.63, 12.30)	14.00 (-2.39, 46.59)
Calcium Suppl.	50%	1.52 (-0.04, 3.76)	55.54 (-4.72, 184.61)	2633 (-224, 8750)	140.45 (-11.52, 469.69)	532.07 (-43.63, 1779.32)	21.74 (-1.78, 72.70)	82.35 (-6.75, 275.39)
	90%	2.73 (-0.07, 6.77)	99.98 (-8.50, 332.30)	4739 (-403, 15751)	252.81 (-20.73, 845.45)	957.73 (-78.53, 3202.78)	21.74 (-1.78, 72.70)	82.35 (-6.75, 275.39)
Multiple Micronutrient Suppl.	50%	0.45 (0.12, 0.82)	15.61 (2.10, 40.85)	740 (100, 1936)	39.37 (5.38, 101.91)	149.14 (20.36, 386.06)	6.09 (0.83, 15.77)	23.08 (3.15, 59.75)
	90%	1.10 (0.24, 2.14)	36.82 (5.11, 97.40)	1745 (242, 4617)	92.83 (13.18, 240.05)	351.65 (49.92, 909.38)	7.98 (1.13, 20.64)	30.24 (4.29, 78.19)
Balanced Protein Suppl.	50%	0.00 (-0.00, 0.02)	0.13 (-0.15, 0.61)	6 (-7, 29)	0.33 (-0.38, 1.55)	1.27 (-1.43, 5.86)	5.70 (-6.40, 21.64)	21.59 (-24.24, 81.97)
	90%	0.01 (-0.01, 0.03)	0.24 (-0.26, 1.09)	11 (-12, 52)	0.60 (-0.68, 2.79)	2.28 (-2.58, 10.55)	5.70 (-6.40, 21.64)	21.59 (-24.24, 81.97)

References for Data Inputs

[1] Blencowe H, Krasevec J, Onis M De, et al. Articles National , regional , and worldwide estimates of low birthweight in 2015 , with trends from 2000: a systematic analysis. Lancet Glob Heal. 2019;(18):1-12.
[2] Chawanpaiboon S, Vogel JP, Moller AB, et al. Global, regional, and national estimates of levels of preterm birth in 2014: a systematic review and modelling analysis. Lancet Glob Heal. 2019;7(1):e37-e46.
[3] United National Population Division World Population Prospects 2019.
[4] Fink G, Peet E, Danaei G, et al. Schooling and wage income losses due to early-childhood growth faltering in developing countries: National, regional, and global estimates. Am J Clin Nutr. 2016;104(1):104-112.
[5] Country specific annual wage data from World Indicators Database. Average yearly wage was estimated to be 2/3 of the gross domestic product in 2010 constant US dollars and 2011 International dollars, adjusted for purchasing power parity.
[6] NCD Risk Factor Collaboration. Trends in adult body-mass index in 200 countries from 1975 to 2014: a pooled analysis of 1698 population-based measurement studies with 19·2 million participants. Lancet. 2016;387(10026):1377-1396.
[7] Stevens GA, Finucane MM, De-Regil LM, et al. Global, regional, and national trends in haemoglobin concentration and prevalence of total and severe anaemia in children and pregnant and non-pregnant women for 1995-2011: A systematic analysis of population-representative data. Lancet Glob Heal. 2013;1(1):16-25.
[8] Coverage of iron-folic acid supplementation abstracted from the most recent Demographic Health Survey or imputed based on sub-regional average. Indicator used: % women in the past five years who took iron tablets or syrup for >90 days.

El Salvador

Region: Latin America and Caribbean**; Sub-region:** Central Latin America

Low birthweight prevalence[1]: 10.3% (95% CI: 8.0, 13.3)

Preterm birth prevalence[2]: 9.6% (95% CI: 7.4, 12.1)

Number of births[3]: 589,000

Returns to education[4]: 9.5% (95% CI: 7.5, 11.6)

GDP per capita 2010 US$ (estimated annual wage)[5]: $3315 ($2210/year)

GDP per capita 2011 International $ (estimated annual wage)[5]: $6979 ($4653/year)

Prevalence of low BMI[6]: 2.0% (95% CI: 0.8, 4.1)

Prevalence of anemia[7]: 28.8% (95% CI: 13.3, 50.9)

Baseline coverage of IFA[8]: 33.3%

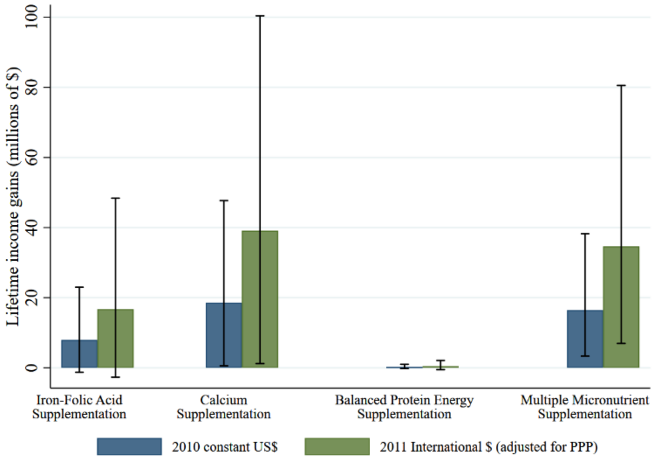

Figure: Benefits by birth cohort size for estimated gains in lifetime wages attributable to scaling up nutrition interventions to 90% target coverage

Table: Impact of maternal prenatal nutrition intervention on human capital and labour market outcomes, through improvements in low birthweight or preterm birth and schooling in El Salvador.

Intervention	Target Coverage (%)	Absolute reduction in birth outcome (%)	Benefits by cohorts: School years gained (in 1000 s)	No. of additional students completing secondary school	Benefits by cohorts: Lifetime wages		Returns in lifetime earnings per child born to a targeted pregnant woman	
					in US $ millions	in International $ millions	in US $	in International $
**Iron/Folic Acid Suppl.**	90%	0.97 (-0.17, 2.09)	1.43 (-0.22, 3.89)	56 (-9, 153)	7.94 (-1.27, 22.98)	16.71 (-2.67, 48.38)	14.97 (-2.39, 43.35)	31.53 (-5.03, 91.27)
**Calcium Suppl.**	50%	1.15 (0.25, 1.99)	1.88 (0.06, 4.75)	74 (2, 186)	10.32 (0.32, 26.50)	21.72 (0.67, 55.79)	35.03 (1.08, 89.98)	73.76 (2.28, 189.44)
	90%	2.07 (0.46, 3.58)	3.38 (0.11, 8.55)	133 (4, 335)	18.57 (0.57, 47.70)	39.10 (1.21, 100.42)	35.03 (1.08, 89.98)	73.76 (2.28, 189.44)
**Multiple Micronutrient Suppl.**	50%	0.82 (0.44, 1.21)	1.28 (0.29, 2.71)	50 (11, 106)	7.14 (1.67, 15.74)	15.04 (3.52, 33.14)	24.25 (5.68, 53.44)	51.06 (11.97, 112.52)
	90%	1.93 (0.80, 3.11)	2.97 (0.59, 6.58)	116 (23, 258)	16.45 (3.32, 38.25)	34.63 (6.98, 80.53)	31.02 (6.26, 72.16)	65.32 (13.17, 151.92)
**Balanced Protein Suppl.**	50%	0.02 (-0.01, 0.05)	0.02 (-0.02, 0.09)	1 (-1, 4)	0.11 (-0.14, 0.55)	0.24 (-0.29, 1.16)	22.65 (-24.29, 75.84)	47.69 (-51.14, 159.67)
	90%	0.03 (-0.03, 0.09)	0.04 (-0.04, 0.17)	1 (-2, 7)	0.20 (-0.25, 0.99)	0.43 (-0.53, 2.09)	22.65 (-24.29, 75.84)	47.69 (-51.14, 159.67)

References for Data Inputs

[1] Blencowe H, Krasevec J, Onis M De, et al. Articles National , regional , and worldwide estimates of low birthweight in 2015 , with trends from 2000: a systematic analysis. Lancet Glob Heal. 2019;(18):1-12.

[2] Chawanpaiboon S, Vogel JP, Moller AB, et al. Global, regional, and national estimates of levels of preterm birth in 2014: a systematic review and modelling analysis. Lancet Glob Heal. 2019;7(1):e37-e46.

[3] United National Population Division World Population Prospects 2019.

[4] Fink G, Peet E, Danaei G, et al. Schooling and wage income losses due to early-childhood growth faltering in developing countries: National, regional, and global estimates. Am J Clin Nutr. 2016;104(1):104-112.

[5] Country specific annual wage data from World Indicators Database. Average yearly wage was estimated to be 2/3 of the gross domestic product in 2010 constant US dollars and 2011 International dollars, adjusted for purchasing power parity.

[6] NCD Risk Factor Collaboration. Trends in adult body-mass index in 200 countries from 1975 to 2014: a pooled analysis of 1698 population-based measurement studies with 19·2 million participants. Lancet. 2016;387(10026):1377-1396.

[7] Stevens GA, Finucane MM, De-Regil LM, et al. Global, regional, and national trends in haemoglobin concentration and prevalence of total and severe anaemia in children and pregnant and non-pregnant women for 1995-2011: A systematic analysis of population-representative data. Lancet Glob Heal. 2013;1(1):16-25.

[8] Coverage of iron-folic acid supplementation abstracted from the most recent Demographic Health Survey or imputed based on sub-regional average. Indicator used: % women in the past five years who took iron tablets or syrup for >90 days.

Equatorial Guinea

Region: Sub-Saharan Africa**; Sub-region:** Central Sub-Saharan Africa

Low birthweight prevalence[1]: 13.6% (95% CI: 8.8, 18.4)

Preterm birth prevalence[2]: 11.7% (95% CI: 10.6, 12.8)

Number of births[3]: 215,000

Returns to education[4]: 9.9% (95% CI: 7.0, 12.9)

GDP per capita 2010 US$ (estimated annual wage)[5]: $14080 ($9387/year)

GDP per capita 2011 International $ (estimated annual wage)[5]: $27709 ($18472/year)

Prevalence of low BMI[6]: 10.8% (95% CI: 3.0, 18.6)

Prevalence of anemia[7]: 52.3% (95% CI: 34.6, 69.9)

Baseline coverage of IFA[8]: 34.1%

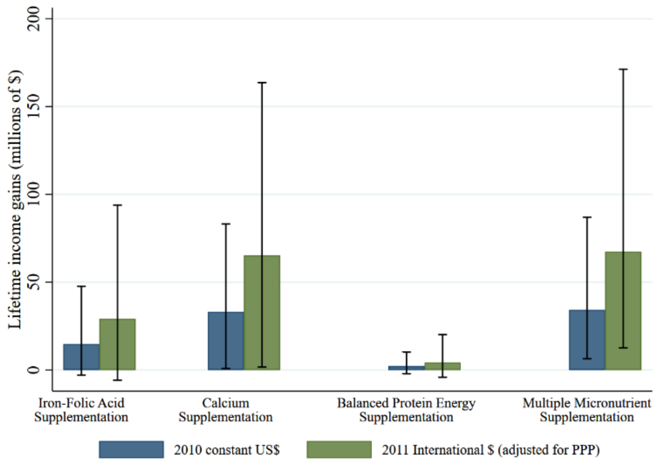

Figure: Benefits by birth cohort size for estimated gains in lifetime wages attributable to scaling up nutrition interventions to 90% target coverage

Table: Impact of maternal prenatal nutrition intervention on human capital and labour market outcomes, through improvements in low birthweight or preterm birth and schooling in Equatorial Guinea.

Intervention	Target Coverage (%)	Absolute reduction in birth outcome (%)	Benefits by cohorts: School years gained (in 1000 s)	No. of additional students completing secondary school	Benefits by cohorts: Lifetime wages		Returns in lifetime earnings per child born to a targeted pregnant woman	
					in US $ millions	in International $ millions	in US $	in International $
Iron/Folic Acid Suppl.	90%	1.26 (-0.26, 2.91)	0.60 (-0.10, 1.82)	28 (-5, 86)	14.81 (-2.94, 47.70)	29.14 (-5.79, 93.86)	76.52 (-15.21, 246.50)	150.59 (-29.92, 485.09)
Calcium Suppl.	50%	1.41 (0.30, 2.35)	0.75 (0.02, 1.81)	35 (1, 85)	18.42 (0.46, 46.17)	36.24 (0.91, 90.86)	171.33 (4.32, 429.50)	337.16 (8.49, 845.23)
	90%	2.54 (0.54, 4.23)	1.35 (0.04, 3.25)	64 (2, 153)	33.15 (0.83, 83.11)	65.24 (1.64, 163.55)	171.33 (4.32, 429.50)	337.16 (8.49, 845.23)
Multiple Micronutrient Suppl.	50%	1.21 (0.63, 1.82)	0.61 (0.13, 1.42)	29 (6, 67)	14.95 (3.12, 36.77)	29.41 (6.15, 72.37)	139.03 (29.06, 342.08)	273.60 (57.18, 673.20)
	90%	2.77 (1.29, 4.50)	1.38 (0.28, 3.32)	65 (13, 157)	34.23 (6.41, 87.02)	67.35 (12.62, 171.24)	176.87 (33.15, 449.69)	348.07 (65.24, 884.96)
Balanced Protein Suppl.	50%	0.11 (-0.09, 0.36)	0.05 (-0.05, 0.22)	2 (-2, 10)	1.22 (-1.17, 5.68)	2.41 (-2.30, 11.18)	119.17 (-112.23, 410.74)	234.52 (-220.87, 808.31)
	90%	0.20 (-0.17, 0.65)	0.09 (-0.08, 0.39)	4 (-4, 19)	2.20 (-2.10, 10.23)	4.33 (-4.14, 20.13)	119.17 (-112.23, 410.74)	234.52 (-220.87, 808.31)

References for Data Inputs

[1] Blencowe H, Krasevec J, Onis M De, et al. Articles National , regional , and worldwide estimates of low birthweight in 2015 , with trends from 2000: a systematic analysis. Lancet Glob Heal. 2019;(18):1-12.

[2] Chawanpaiboon S, Vogel JP, Moller AB, et al. Global, regional, and national estimates of levels of preterm birth in 2014: a systematic review and modelling analysis. Lancet Glob Heal. 2019;7(1):e37-e46.

[3] United National Population Division World Population Prospects 2019.

[4] Fink G, Peet E, Danaei G, et al. Schooling and wage income losses due to early-childhood growth faltering in developing countries: National, regional, and global estimates. Am J Clin Nutr. 2016;104(1):104-112.

[5] Country specific annual wage data from World Indicators Database. Average yearly wage was estimated to be 2/3 of the gross domestic product in 2010 constant US dollars and 2011 International dollars, adjusted for purchasing power parity.

[6] NCD Risk Factor Collaboration. Trends in adult body-mass index in 200 countries from 1975 to 2014: a pooled analysis of 1698 population-based measurement studies with 19·2 million participants. Lancet. 2016;387(10026):1377-1396.

[7] Stevens GA, Finucane MM, De-Regil LM, et al. Global, regional, and national trends in haemoglobin concentration and prevalence of total and severe anaemia in children and pregnant and non-pregnant women for 1995-2011: A systematic analysis of population-representative data. Lancet Glob Heal. 2013;1(1):16-25.

[8] Coverage of iron-folic acid supplementation abstracted from the most recent Demographic Health Survey or imputed based on sub-regional average. Indicator used: % women in the past five years who took iron tablets or syrup for >90 days.

Eritrea

Region: Sub-Saharan Africa**; Sub-region:** Eastern Sub-Saharan Africa

Low birthweight prevalence[1]: 14.1% (95% CI: 8.5, 19.7)

Preterm birth prevalence[2]: 12.0% (95% CI: 8.6, 16.7)

Number of births[3]: 528,000

Returns to education[4]: 11.3% (95% CI: 9.7, 12.9)

GDP per capita 2010 US$ (estimated annual wage)[5]: $668 ($445/year)

GDP per capita 2011 International $ (estimated annual wage)[5]: $1962 ($1308/year)

Prevalence of low BMI[6]: 16.9% (95% CI: 9.9, 25.2)

Prevalence of anemia[7]: 37.3% (95% CI: 20.5, 58.4)

Baseline coverage of IFA[8]: 19.2%

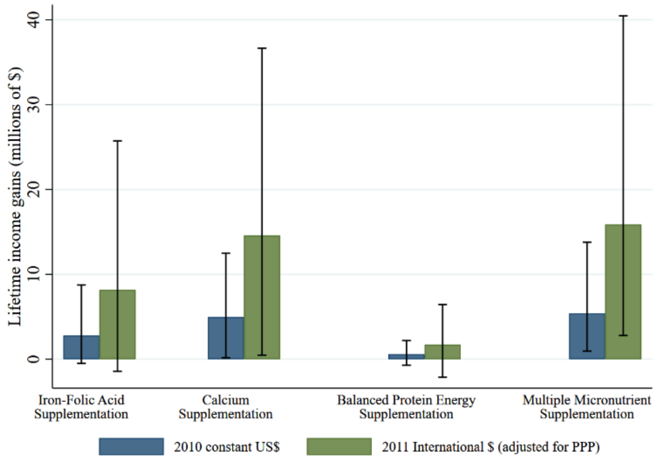

Figure: Benefits by birth cohort size for estimated gains in lifetime wages attributable to scaling up nutrition interventions to 90% target coverage

Table: Impact of maternal prenatal nutrition intervention on human capital and labour market outcomes, through improvements in low birthweight or preterm birth and schooling in Eritrea.

Intervention	Target Coverage (%)	Absolute reduction in birth outcome (%)	Benefits by cohorts: School years gained (in 1000 s)	No. of additional students completing secondary school	Benefits by cohorts: Lifetime wages		Returns in lifetime earnings per child born to a targeted pregnant woman	
					in US $ millions	in International $ millions	in US $	in International $
Iron/Folic Acid Suppl.	90%	1.58 (-0.28, 3.66)	2.09 (-0.38, 6.37)	57 (-10, 175)	2.78 (-0.49, 8.75)	8.17 (-1.44, 25.72)	5.85 (-1.03, 18.42)	17.19 (-3.03, 54.12)
Calcium Suppl.	50%	1.39 (0.28, 2.62)	2.03 (0.07, 5.05)	56 (2, 138)	2.76 (0.09, 6.93)	8.10 (0.26, 20.37)	10.45 (0.34, 26.26)	30.69 (0.99, 77.15)
	90%	2.51 (0.50, 4.72)	3.66 (0.12, 9.09)	100 (3, 249)	4.96 (0.16, 12.48)	14.58 (0.47, 36.66)	10.45 (0.34, 26.26)	30.69 (0.99, 77.15)
Multiple Micronutrient Suppl.	50%	1.45 (0.64, 2.51)	1.96 (0.36, 4.85)	54 (10, 133)	2.67 (0.47, 6.73)	7.85 (1.37, 19.76)	10.13 (1.77, 25.48)	29.75 (5.20, 74.85)
	90%	2.96 (1.12, 5.28)	3.99 (0.69, 10.00)	110 (19, 274)	5.41 (0.95, 13.78)	15.88 (2.80, 40.48)	11.38 (2.00, 29.00)	33.43 (5.88, 85.19)
Balanced Protein Suppl.	50%	0.19 (-0.18, 0.57)	0.24 (-0.29, 0.91)	6 (-8, 25)	0.32 (-0.40, 1.22)	0.95 (-1.18, 3.58)	7.41 (-8.11, 24.83)	21.76 (-23.82, 72.93)
	90%	0.35 (-0.32, 1.02)	0.43 (-0.52, 1.64)	12 (-14, 45)	0.58 (-0.72, 2.19)	1.70 (-2.12, 6.44)	7.41 (-8.11, 24.83)	21.76 (-23.82, 72.93)

References for Data Inputs

[1] Blencowe H, Krasevec J, Onis M De, et al. Articles National , regional , and worldwide estimates of low birthweight in 2015 , with trends from 2000: a systematic analysis. Lancet Glob Heal. 2019;(18):1-12.
[2] Chawanpaiboon S, Vogel JP, Moller AB, et al. Global, regional, and national estimates of levels of preterm birth in 2014: a systematic review and modelling analysis. Lancet Glob Heal. 2019;7(1):e37-e46.
[3] United National Population Division World Population Prospects 2019.
[4] Fink G, Peet E, Danaei G, et al. Schooling and wage income losses due to early-childhood growth faltering in developing countries: National, regional, and global estimates. Am J Clin Nutr. 2016;104(1):104-112.
[5] Country specific annual wage data from World Indicators Database. Average yearly wage was estimated to be 2/3 of the gross domestic product in 2010 constant US dollars and 2011 International dollars, adjusted for purchasing power parity.
[6] NCD Risk Factor Collaboration. Trends in adult body-mass index in 200 countries from 1975 to 2014: a pooled analysis of 1698 population-based measurement studies with 19·2 million participants. Lancet. 2016;387(10026):1377-1396.
[7] Stevens GA, Finucane MM, De-Regil LM, et al. Global, regional, and national trends in haemoglobin concentration and prevalence of total and severe anaemia in children and pregnant and non-pregnant women for 1995-2011: A systematic analysis of population-representative data. Lancet Glob Heal. 2013;1(1):16-25.
[8] Coverage of iron-folic acid supplementation abstracted from the most recent Demographic Health Survey or imputed based on sub-regional average. Indicator used: % women in the past five years who took iron tablets or syrup for >90 days.

Eswatini

Region: Sub-Saharan Africa**; Sub-region:** Southern Sub-Saharan Africa

Low birthweight prevalence[1]: 10.3% (95% CI: 7.9, 13.7)

Preterm birth prevalence[2]: 12.1% (95% CI: 10.2, 13.9)

Number of births[3]: 151,000

Returns to education[4]: 16.1% (95% CI: 15.3, 16.8)

GDP per capita 2010 US$ (estimated annual wage)[5]: $4688 ($3125/year)

GDP per capita 2011 International $ (estimated annual wage)[5]: $9269 ($6179/year)

Prevalence of low BMI[6]: 5.0% (95% CI: 2.0, 9.8)

Prevalence of anemia[7]: 31.4% (95% CI: 18.5, 47.2)

Baseline coverage of IFA[8]: 45.0%

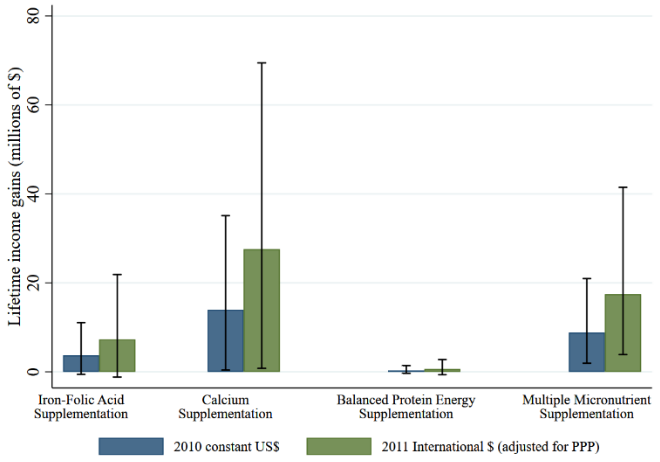

Figure: Benefits by birth cohort size for estimated gains in lifetime wages attributable to scaling up nutrition interventions to 90% target coverage

Table: Impact of maternal prenatal nutrition intervention on human capital and labour market outcomes, through improvements in low birthweight or preterm birth and schooling in Eswatini.

Intervention	Target Coverage (%)	Absolute reduction in birth outcome (%)	Benefits by cohorts: School years gained (in 1000 s)	No. of additional students completing secondary school	Benefits by cohorts: Lifetime wages		Returns in lifetime earnings per child born to a targeted pregnant woman	
					in US $ millions	in International $ millions	in US $	in International $
Iron/Folic Acid Suppl.	90%	0.78 (-0.14, 1.77)	0.27 (-0.04, 0.81)	15 (-2, 44)	3.70 (-0.59, 11.06)	7.31 (-1.16, 21.86)	27.19 (-4.33, 81.37)	53.76 (-8.57, 160.88)
Calcium Suppl.	50%	1.44 (0.32, 2.48)	0.57 (0.02, 1.44)	31 (1, 78)	7.74 (0.21, 19.51)	15.31 (0.42, 38.57)	102.56 (2.84, 258.39)	202.79 (5.61, 510.91)
	90%	2.60 (0.57, 4.47)	1.03 (0.03, 2.59)	56 (2, 141)	13.94 (0.39, 35.12)	27.56 (0.76, 69.43)	102.56 (2.84, 258.39)	202.79 (5.61, 510.91)
Multiple Micronutrient Suppl.	50%	0.64 (0.35, 0.98)	0.24 (0.05, 0.53)	13 (3, 29)	3.30 (0.70, 7.13)	6.53 (1.39, 14.09)	43.77 (9.31, 94.38)	86.55 (18.40, 186.62)
	90%	1.77 (0.84, 2.95)	0.66 (0.15, 1.55)	36 (8, 84)	8.82 (1.95, 20.97)	17.44 (3.86, 41.46)	64.89 (14.37, 154.29)	128.30 (28.40, 305.07)
Balanced Protein Suppl.	50%	0.04 (-0.03, 0.13)	0.01 (-0.01, 0.06)	1 (-1, 3)	0.17 (-0.19, 0.77)	0.34 (-0.37, 1.53)	53.02 (-50.34, 160.25)	104.84 (-99.53, 316.86)
	90%	0.07 (-0.06, 0.23)	0.02 (-0.02, 0.10)	1 (-1, 6)	0.31 (-0.34, 1.39)	0.62 (-0.67, 2.75)	53.02 (-50.34, 160.25)	104.84 (-99.53, 316.86)

References for Data Inputs

[1] Blencowe H, Krasevec J, Onis M De, et al. Articles National , regional , and worldwide estimates of low birthweight in 2015 , with trends from 2000: a systematic analysis. Lancet Glob Heal. 2019;(18):1-12.
[2] Chawanpaiboon S, Vogel JP, Moller AB, et al. Global, regional, and national estimates of levels of preterm birth in 2014: a systematic review and modelling analysis. Lancet Glob Heal. 2019;7(1):e37-e46.
[3] United National Population Division World Population Prospects 2019.
[4] Fink G, Peet E, Danaei G, et al. Schooling and wage income losses due to early-childhood growth faltering in developing countries: National, regional, and global estimates. Am J Clin Nutr. 2016;104(1):104-112.
[5] Country specific annual wage data from World Indicators Database. Average yearly wage was estimated to be 2/3 of the gross domestic product in 2010 constant US dollars and 2011 International dollars, adjusted for purchasing power parity.
[6] NCD Risk Factor Collaboration. Trends in adult body-mass index in 200 countries from 1975 to 2014: a pooled analysis of 1698 population-based measurement studies with 19·2 million participants. Lancet. 2016;387(10026):1377-1396.
[7] Stevens GA, Finucane MM, De-Regil LM, et al. Global, regional, and national trends in haemoglobin concentration and prevalence of total and severe anaemia in children and pregnant and non-pregnant women for 1995-2011: A systematic analysis of population-representative data. Lancet Glob Heal. 2013;1(1):16-25.
[8] Coverage of iron-folic acid supplementation abstracted from the most recent Demographic Health Survey or imputed based on sub-regional average. Indicator used: % women in the past five years who took iron tablets or syrup for >90 days.

Ethiopia

Region: Sub-Saharan Africa**; Sub-region:** Eastern Sub-Saharan Africa

Low birthweight prevalence[1]: 20.3% (95% CI: 14.7, 25.9)

Preterm birth prevalence[2]: 12.0% (95% CI: 8.6, 16.7)

Number of births[3]: 17,572,000

Returns to education[4]: 12.5% (95% CI: 8.4, 16.6)

GDP per capita 2010 US$ (estimated annual wage)[5]: $483 ($322/year)

GDP per capita 2011 International $ (estimated annual wage)[5]: $1518 ($1012/year)

Prevalence of low BMI[6]: 14.4% (95% CI: 7.7, 22.8)

Prevalence of anemia[7]: 24.4% (95% CI: 19.1, 31.7)

Baseline coverage of IFA[8]: 5.1%

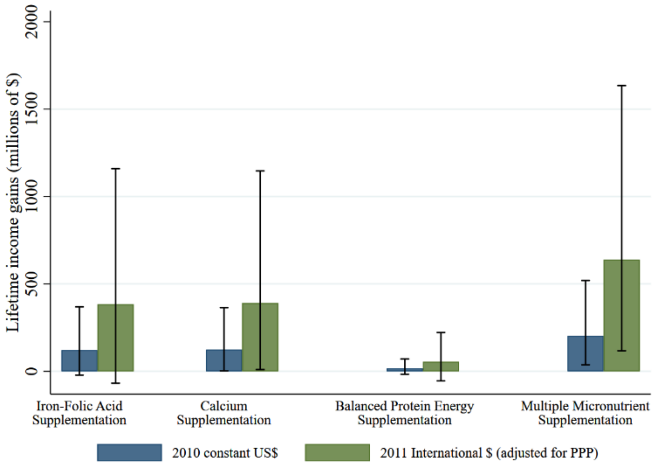

Figure: Benefits by birth cohort size for estimated gains in lifetime wages attributable to scaling up nutrition interventions to 90% target coverage

Table: Impact of maternal prenatal nutrition intervention on human capital and labour market outcomes, through improvements in low birthweight or preterm birth and schooling in Ethiopia.

Intervention	Target Coverage (%)	Absolute reduction in birth outcome (%)	Benefits by cohorts: School years gained (in 1000 s)	No. of additional students completing secondary school	Benefits by cohorts: Lifetime wages		Returns in lifetime earnings per child born to a targeted pregnant woman	
					in US $ millions	in International $ millions	in US $	in International $
Iron/Folic Acid Suppl.	90%	2.70 (-0.49, 5.76)	113.76 (-19.64, 314.40)	2002 (-346, 5533)	122.00 (-21.52, 368.43)	383.83 (-67.72, 1159.16)	7.71 (-1.36, 23.30)	24.27 (-4.28, 73.30)
Calcium Suppl.	50%	1.41 (0.30, 2.68)	65.05 (2.10, 171.58)	1145 (37, 3020)	68.99 (1.78, 202.51)	217.04 (5.60, 637.12)	7.85 (0.20, 23.05)	24.70 (0.64, 72.52)
	90%	2.54 (0.55, 4.83)	117.10 (3.78, 308.84)	2061 (67, 5436)	124.17 (3.20, 364.51)	390.68 (10.08, 1146.82)	7.85 (0.20, 23.05)	24.70 (0.64, 72.52)
Multiple Micronutrient Suppl.	50%	2.41 (0.82, 4.00)	103.74 (18.19, 237.91)	1826 (320, 4187)	109.87 (20.95, 279.54)	345.69 (65.91, 879.47)	12.51 (2.38, 31.82)	39.34 (7.50, 100.10)
	90%	4.47 (1.45, 7.48)	191.58 (33.41, 440.73)	3372 (588, 7757)	203.17 (37.51, 519.48)	639.22 (118.03, 1634.37)	12.85 (2.37, 32.85)	40.42 (7.46, 103.34)
Balanced Protein Suppl.	50%	0.22 (-0.21, 0.64)	8.95 (-10.16, 34.66)	157 (-179, 610)	9.63 (-9.70, 39.29)	30.28 (-30.51, 123.61)	8.14 (-7.57, 28.70)	25.61 (-23.81, 90.28)
	90%	0.40 (-0.38, 1.16)	16.10 (-18.29, 62.39)	283 (-322, 1098)	17.33 (-17.46, 70.72)	54.51 (-54.92, 222.50)	8.14 (-7.57, 28.70)	25.61 (-23.81, 90.28)

References for Data Inputs

[1] Blencowe H, Krasevec J, Onis M De, et al. Articles National , regional , and worldwide estimates of low birthweight in 2015 , with trends from 2000: a systematic analysis. Lancet Glob Heal. 2019;(18):1-12.

[2] Chawanpaiboon S, Vogel JP, Moller AB, et al. Global, regional, and national estimates of levels of preterm birth in 2014: a systematic review and modelling analysis. Lancet Glob Heal. 2019;7(1):e37-e46.

[3] United National Population Division World Population Prospects 2019.

[4] Fink G, Peet E, Danaei G, et al. Schooling and wage income losses due to early-childhood growth faltering in developing countries: National, regional, and global estimates. Am J Clin Nutr. 2016;104(1):104-112.

[5] Country specific annual wage data from World Indicators Database. Average yearly wage was estimated to be 2/3 of the gross domestic product in 2010 constant US dollars and 2011 International dollars, adjusted for purchasing power parity.

[6] NCD Risk Factor Collaboration. Trends in adult body-mass index in 200 countries from 1975 to 2014: a pooled analysis of 1698 population-based measurement studies with 19·2 million participants. Lancet. 2016;387(10026):1377-1396.

[7] Stevens GA, Finucane MM, De-Regil LM, et al. Global, regional, and national trends in haemoglobin concentration and prevalence of total and severe anaemia in children and pregnant and non-pregnant women for 1995-2011: A systematic analysis of population-representative data. Lancet Glob Heal. 2013;1(1):16-25.

[8] Coverage of iron-folic acid supplementation abstracted from the most recent Demographic Health Survey or imputed based on sub-regional average. Indicator used: % women in the past five years who took iron tablets or syrup for >90 days.

Fiji

Region: Southeast Asia, East Asia, and Oceania**; Sub-region:** Oceania

Low birthweight prevalence[1]: 8.1% (95% CI: 5.5, 10.8)

Preterm birth prevalence[2]: 10.0% (95% CI: 9.2, 10.8)

Number of births[3]: 95,000

Returns to education[4]: 6.1% (95% CI: 2.7, 9.6)

GDP per capita 2010 US$ (estimated annual wage)[5]: $4359 ($2906/year)

GDP per capita 2011 International $ (estimated annual wage)[5]: $8776 ($5851/year)

Prevalence of low BMI[6]: 1.4% (95% CI: -1.3, 4.2)

Prevalence of anemia[7]: 29.8% (95% CI: 6.3, 53.3)

Baseline coverage of IFA[8]: 44.2%

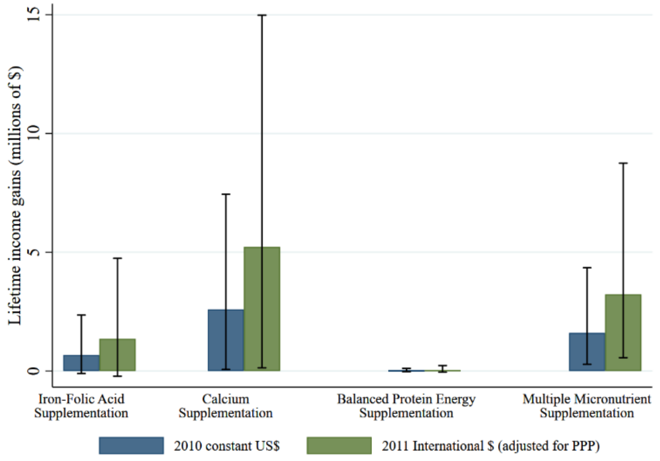

Figure: Benefits by birth cohort size for estimated gains in lifetime wages attributable to scaling up nutrition interventions to 90% target coverage

Table: Impact of maternal prenatal nutrition intervention on human capital and labour market outcomes, through improvements in low birthweight or preterm birth and schooling in Fiji.

Intervention	Target Coverage (%)	Absolute reduction in birth outcome (%)	Benefits by cohorts: School years gained (in 1000 s)	No. of additional students completing secondary school	Benefits by cohorts: Lifetime wages		Returns in lifetime earnings per child born to a targeted pregnant woman	
					in US $ millions	in International $ millions	in US $	in International $
Iron/Folic Acid Suppl.	90%	0.61 (-0.11, 1.43)	0.15 (-0.02, 0.44)	2 (-0, 7)	0.67 (-0.11, 2.36)	1.36 (-0.22, 4.74)	7.88 (-1.26, 27.55)	15.87 (-2.54, 55.47)
Calcium Suppl.	50%	1.20 (0.26, 1.97)	0.31 (0.01, 0.74)	5 (0, 12)	1.44 (0.04, 4.13)	2.90 (0.07, 8.32)	30.33 (0.77, 87.03)	61.06 (1.54, 175.21)
	90%	2.15 (0.46, 3.55)	0.57 (0.02, 1.34)	9 (0, 21)	2.59 (0.07, 7.44)	5.22 (0.13, 14.98)	30.33 (0.77, 87.03)	61.06 (1.54, 175.21)
Multiple Micronutrient Suppl.	50%	0.51 (0.27, 0.81)	0.13 (0.03, 0.29)	2 (0, 5)	0.60 (0.10, 1.54)	1.20 (0.20, 3.10)	12.59 (2.05, 32.46)	25.35 (4.13, 65.34)
	90%	1.40 (0.64, 2.37)	0.34 (0.07, 0.82)	5 (1, 13)	1.60 (0.28, 4.35)	3.23 (0.56, 8.75)	18.74 (3.26, 50.82)	37.73 (6.57, 102.31)
Balanced Protein Suppl.	50%	0.01 (-0.01, 0.05)	0.00 (-0.00, 0.01)	0 (-0, 0)	0.01 (-0.01, 0.06)	0.01 (-0.03, 0.13)	14.35 (-13.60, 57.76)	28.89 (-27.37, 116.27)
	90%	0.01 (-0.02, 0.08)	0.00 (-0.01, 0.02)	0 (-0, 0)	0.01 (-0.03, 0.11)	0.02 (-0.05, 0.23)	14.35 (-13.60, 57.76)	28.89 (-27.37, 116.27)

References for Data Inputs

1 Blencowe H, Krasevec J, Onis M De, et al. Articles National , regional , and worldwide estimates of low birthweight in 2015 , with trends from 2000: a systematic analysis. Lancet Glob Heal. 2019;(18):1-12.

2 Chawanpaiboon S, Vogel JP, Moller AB, et al. Global, regional, and national estimates of levels of preterm birth in 2014: a systematic review and modelling analysis. Lancet Glob Heal. 2019;7(1):e37-e46.

3 United National Population Division World Population Prospects 2019.

4 Fink G, Peet E, Danaei G, et al. Schooling and wage income losses due to early-childhood growth faltering in developing countries: National, regional, and global estimates. Am J Clin Nutr. 2016;104(1):104-112.

5 Country specific annual wage data from World Indicators Database. Average yearly wage was estimated to be 2/3 of the gross domestic product in 2010 constant US dollars and 2011 International dollars, adjusted for purchasing power parity.

6 NCD Risk Factor Collaboration. Trends in adult body-mass index in 200 countries from 1975 to 2014: a pooled analysis of 1698 population-based measurement studies with 19·2 million participants. Lancet. 2016;387(10026):1377-1396.

7 Stevens GA, Finucane MM, De-Regil LM, et al. Global, regional, and national trends in haemoglobin concentration and prevalence of total and severe anaemia in children and pregnant and non-pregnant women for 1995-2011: A systematic analysis of population-representative data. Lancet Glob Heal. 2013;1(1):16-25.

8 Coverage of iron-folic acid supplementation abstracted from the most recent Demographic Health Survey or imputed based on sub-regional average. Indicator used: % women in the past five years who took iron tablets or syrup for >90 days.

Gabon

Region: Sub-Saharan Africa**; Sub-region:** Central Sub-Saharan Africa

Low birthweight prevalence[1]: 14.2% (95% CI: 11.2, 18.8)

Preterm birth prevalence[2]: 12.0% (95% CI: 8.6, 16.7)

Number of births[3]: 334,000

Returns to education[4]: 9.9% (95% CI: 7.0, 12.9)

GDP per capita 2010 US$ (estimated annual wage)[5]: $9512 ($6341/year)

GDP per capita 2011 International $ (estimated annual wage)[5]: $16685 ($11123/year)

Prevalence of low BMI[6]: 6.6% (95% CI: 2.7, 12.5)

Prevalence of anemia[7]: 52.3% (95% CI: 34.6, 69.9)

Baseline coverage of IFA[8]: 56.8%

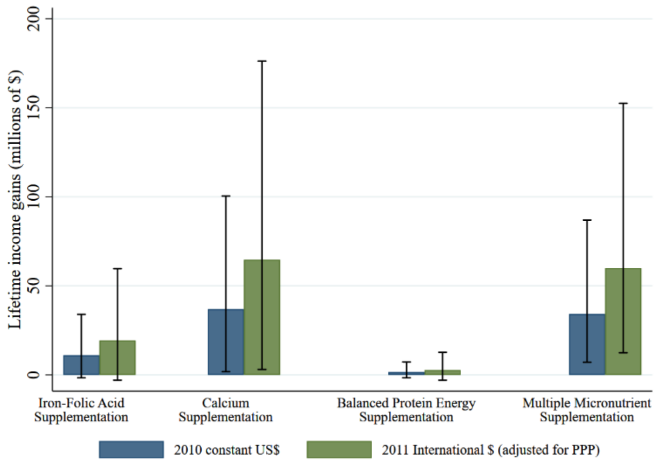

Figure: Benefits by birth cohort size for estimated gains in lifetime wages attributable to scaling up nutrition interventions to 90% target coverage

Table: Impact of maternal prenatal nutrition intervention on human capital and labour market outcomes, through improvements in low birthweight or preterm birth and schooling in Gabon.

Intervention	Target Coverage (%)	Absolute reduction in birth outcome (%)	Benefits by cohorts: School years gained (in 1000 s)	No. of additional students completing secondary school	Benefits by cohorts: Lifetime wages		Returns in lifetime earnings per child born to a targeted pregnant woman	
					in US $ millions	in International $ millions	in US $	in International $
Iron/Folic Acid Suppl.	90%	0.83 (-0.14, 1.83)	0.65 (-0.10, 1.93)	28 (-4, 84)	11.01 (-1.68, 33.98)	19.32 (-2.95, 59.60)	36.64 (-5.59, 113.04)	64.27 (-9.80, 198.28)
Calcium Suppl.	50%	1.40 (0.27, 2.62)	1.24 (0.05, 3.30)	54 (2, 144)	20.49 (0.98, 55.80)	35.93 (1.72, 97.87)	122.67 (5.86, 334.10)	215.17 (10.27, 586.06)
	90%	2.51 (0.49, 4.71)	2.23 (0.09, 5.94)	97 (4, 259)	36.87 (1.76, 100.43)	64.68 (3.09, 176.17)	122.67 (5.86, 334.10)	215.17 (10.27, 586.06)
Multiple Micronutrient Suppl.	50%	0.99 (0.55, 1.51)	0.85 (0.15, 2.02)	37 (7, 88)	13.92 (2.61, 37.35)	24.42 (4.58, 65.52)	83.37 (15.62, 223.66)	146.23 (27.40, 392.33)
	90%	2.47 (1.31, 3.83)	2.05 (0.40, 4.81)	89 (18, 210)	34.16 (7.07, 86.96)	59.91 (12.40, 152.54)	113.63 (23.53, 289.28)	199.31 (41.27, 507.44)
Balanced Protein Suppl.	50%	0.07 (-0.06, 0.23)	0.05 (-0.06, 0.24)	2 (-3, 10)	0.86 (-0.94, 4.02)	1.51 (-1.65, 7.05)	91.08 (-84.99, 294.46)	159.76 (-149.08, 516.51)
	90%	0.12 (-0.11, 0.42)	0.10 (-0.10, 0.43)	4 (-5, 19)	1.55 (-1.69, 7.24)	2.72 (-2.96, 12.70)	91.08 (-84.99, 294.46)	159.76 (-149.08, 516.51)

References for Data Inputs

[1] Blencowe H, Krasevec J, Onis M De, et al. Articles National , regional , and worldwide estimates of low birthweight in 2015 , with trends from 2000: a systematic analysis. Lancet Glob Heal. 2019;(18):1-12.
[2] Chawanpaiboon S, Vogel JP, Moller AB, et al. Global, regional, and national estimates of levels of preterm birth in 2014: a systematic review and modelling analysis. Lancet Glob Heal. 2019;7(1):e37-e46.
[3] United National Population Division World Population Prospects 2019.
[4] Fink G, Peet E, Danaei G, et al. Schooling and wage income losses due to early-childhood growth faltering in developing countries: National, regional, and global estimates. Am J Clin Nutr. 2016;104(1):104-112.
[5] Country specific annual wage data from World Indicators Database. Average yearly wage was estimated to be 2/3 of the gross domestic product in 2010 constant US dollars and 2011 International dollars, adjusted for purchasing power parity.
[6] NCD Risk Factor Collaboration. Trends in adult body-mass index in 200 countries from 1975 to 2014: a pooled analysis of 1698 population-based measurement studies with 19·2 million participants. Lancet. 2016;387(10026):1377-1396.
[7] Stevens GA, Finucane MM, De-Regil LM, et al. Global, regional, and national trends in haemoglobin concentration and prevalence of total and severe anaemia in children and pregnant and non-pregnant women for 1995-2011: A systematic analysis of population-representative data. Lancet Glob Heal. 2013;1(1):16-25.
[8] Coverage of iron-folic acid supplementation abstracted from the most recent Demographic Health Survey or imputed based on sub-regional average. Indicator used: % women in the past five years who took iron tablets or syrup for >90 days.

Gambia

Region: Sub-Saharan Africa**; Sub-region:** Western Sub-Saharan Africa

Low birthweight prevalence[1]: 16.8% (95% CI: 13.5, 21.0)

Preterm birth prevalence[2]: 12.0% (95% CI: 8.6, 16.7)

Number of births[3]: 436,000

Returns to education[4]: 6.8% (95% CI: 5.8, 7.8)

GDP per capita 2010 US$ (estimated annual wage)[5]: $507 ($338/year)

GDP per capita 2011 International $ (estimated annual wage)[5]: $1481 ($988/year)

Prevalence of low BMI[6]: 9.4% (95% CI: 4.7, 15.5)

Prevalence of anemia[7]: 57.1% (95% CI: 32.9, 71.8)

Baseline coverage of IFA[8]: 44.6%

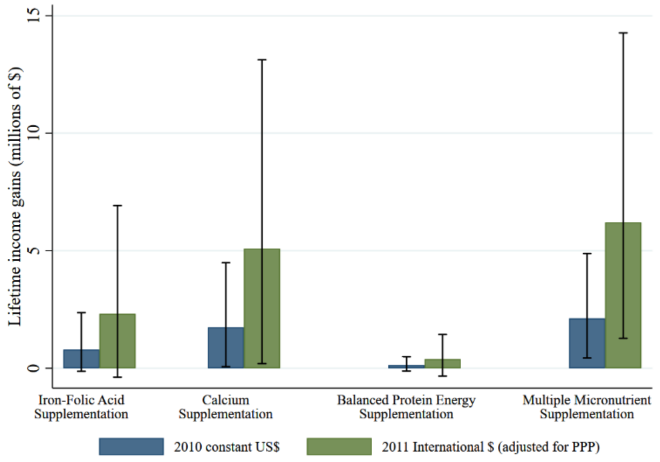

Figure: Benefits by birth cohort size for estimated gains in lifetime wages attributable to scaling up nutrition interventions to 90% target coverage

Table: Impact of maternal prenatal nutrition intervention on human capital and labour market outcomes, through improvements in low birthweight or preterm birth and schooling in Gambia.

Intervention	Target Coverage (%)	Absolute reduction in birth outcome (%)	Benefits by cohorts: School years gained (in 1000 s)	No. of additional students completing secondary school	Benefits by cohorts: Lifetime wages		Returns in lifetime earnings per child born to a targeted pregnant woman	
					in US $ millions	in International $ millions	in US $	in International $
Iron/Folic Acid Suppl.	90%	1.32 (-0.22, 2.80)	1.30 (-0.22, 3.81)	115 (-20, 338)	0.79 (-0.13, 2.37)	2.32 (-0.38, 6.92)	2.02 (-0.33, 6.03)	5.90 (-0.96, 17.64)
Calcium Suppl.	50%	1.38 (0.33, 2.55)	1.57 (0.05, 4.05)	139 (5, 359)	0.97 (0.04, 2.49)	2.83 (0.11, 7.29)	4.44 (0.17, 11.44)	12.98 (0.50, 33.44)
	90%	2.49 (0.59, 4.59)	2.82 (0.10, 7.28)	250 (9, 647)	1.74 (0.07, 4.49)	5.09 (0.20, 13.12)	4.44 (0.17, 11.44)	12.98 (0.50, 33.44)
Multiple Micronutrient Suppl.	50%	1.26 (0.78, 1.83)	1.38 (0.27, 3.03)	123 (24, 269)	0.85 (0.17, 1.88)	2.49 (0.49, 5.50)	3.91 (0.77, 8.63)	11.44 (2.25, 25.23)
	90%	3.30 (1.76, 5.04)	3.47 (0.69, 7.74)	308 (61, 688)	2.12 (0.44, 4.88)	6.20 (1.28, 14.27)	5.41 (1.11, 12.44)	15.81 (3.25, 36.37)
Balanced Protein Suppl.	50%	0.12 (-0.10, 0.36)	0.12 (-0.11, 0.44)	11 (-10, 39)	0.07 (-0.06, 0.27)	0.22 (-0.19, 0.80)	3.79 (-3.66, 12.14)	11.08 (-10.69, 35.48)
	90%	0.22 (-0.17, 0.64)	0.22 (-0.20, 0.78)	19 (-18, 70)	0.13 (-0.12, 0.49)	0.39 (-0.34, 1.44)	3.79 (-3.66, 12.14)	11.08 (-10.69, 35.48)

References for Data Inputs

[1] Blencowe H, Krasevec J, Onis M De, et al. Articles National , regional , and worldwide estimates of low birthweight in 2015 , with trends from 2000: a systematic analysis. Lancet Glob Heal. 2019;(18):1-12.
[2] Chawanpaiboon S, Vogel JP, Moller AB, et al. Global, regional, and national estimates of levels of preterm birth in 2014: a systematic review and modelling analysis. Lancet Glob Heal. 2019;7(1):e37-e46.
[3] United National Population Division World Population Prospects 2019.
[4] Fink G, Peet E, Danaei G, et al. Schooling and wage income losses due to early-childhood growth faltering in developing countries: National, regional, and global estimates. Am J Clin Nutr. 2016;104(1):104-112.
[5] Country specific annual wage data from World Indicators Database. Average yearly wage was estimated to be 2/3 of the gross domestic product in 2010 constant US dollars and 2011 International dollars, adjusted for purchasing power parity.
[6] NCD Risk Factor Collaboration. Trends in adult body-mass index in 200 countries from 1975 to 2014: a pooled analysis of 1698 population-based measurement studies with 19·2 million participants. Lancet. 2016;387(10026):1377-1396.
[7] Stevens GA, Finucane MM, De-Regil LM, et al. Global, regional, and national trends in haemoglobin concentration and prevalence of total and severe anaemia in children and pregnant and non-pregnant women for 1995-2011: A systematic analysis of population-representative data. Lancet Glob Heal. 2013;1(1):16-25.
[8] Coverage of iron-folic acid supplementation abstracted from the most recent Demographic Health Survey or imputed based on sub-regional average. Indicator used: % women in the past five years who took iron tablets or syrup for >90 days.

Georgia

Region: Central Europe, Eastern Europe, Central Asia**; Sub-region:** Central Asia

Low birthweight prevalence[1]: 6.0% (95% CI: 4.6, 7.5)

Preterm birth prevalence[2]: 10.0% (95% CI: 9.2, 10.9)

Number of births[3]: 272,000

Returns to education[4]: 5.1% (95% CI: 2.4, 7.8)

GDP per capita 2010 US$ (estimated annual wage)[5]: $3965 ($2643/year)

GDP per capita 2011 International $ (estimated annual wage)[5]: $9005 ($6004/year)

Prevalence of low BMI[6]: 3.6% (95% CI: 1.6, 5.6)

Prevalence of anemia[7]: 29.0% (95% CI: 9.8, 49.0)

Baseline coverage of IFA[8]: 3.0%

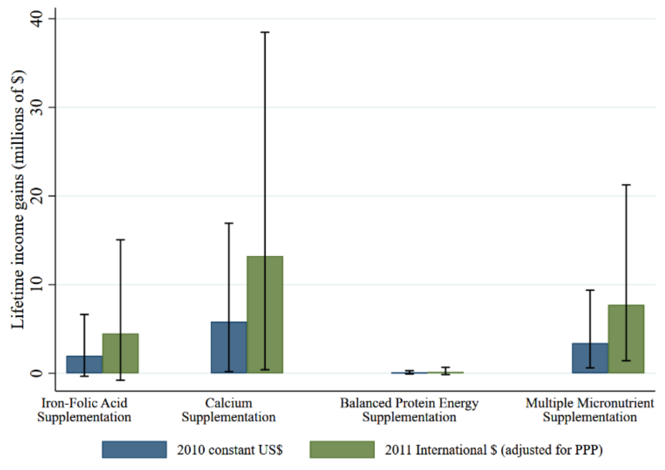

Figure: Benefits by birth cohort size for estimated gains in lifetime wages attributable to scaling up nutrition interventions to 90% target coverage

Table: Impact of maternal prenatal nutrition intervention on human capital and labour market outcomes, through improvements in low birthweight or preterm birth and schooling in Georgia.

Intervention	Target Coverage (%)	Absolute reduction in birth outcome (%)	Benefits by cohorts: School years gained (in 1000 s)	No. of additional students completing secondary school	Benefits by cohorts: Lifetime wages		Returns in lifetime earnings per child born to a targeted pregnant woman	
					in US $ millions	in International $ millions	in US $	in International $
Iron/Folic Acid Suppl.	90%	0.86 (-0.15, 1.68)	0.58 (-0.10, 1.60)	18 (-3, 50)	1.98 (-0.34, 6.63)	4.49 (-0.77, 15.06)	8.08 (-1.39, 27.09)	18.35 (-3.16, 61.53)
Calcium Suppl.	50%	1.21 (0.29, 2.03)	0.94 (0.03, 2.23)	30 (1, 70)	3.24 (0.10, 9.41)	7.35 (0.22, 21.37)	23.79 (0.71, 69.18)	54.03 (1.62, 157.14)
	90%	2.18 (0.53, 3.66)	1.69 (0.05, 4.01)	53 (2, 126)	5.82 (0.17, 16.94)	13.23 (0.40, 38.47)	23.79 (0.71, 69.18)	54.03 (1.62, 157.14)
Multiple Micronutrient Suppl.	50%	0.76 (0.27, 1.22)	0.54 (0.09, 1.26)	17 (3, 40)	1.86 (0.34, 5.12)	4.23 (0.77, 11.62)	13.68 (2.50, 37.62)	31.08 (5.67, 85.45)
	90%	1.39 (0.49, 2.25)	0.98 (0.17, 2.30)	31 (5, 72)	3.40 (0.62, 9.36)	7.73 (1.41, 21.27)	13.90 (2.54, 38.25)	31.57 (5.77, 86.88)
Balanced Protein Suppl.	50%	0.02 (-0.01, 0.05)	0.01 (-0.01, 0.04)	0 (-0, 1)	0.04 (-0.04, 0.16)	0.09 (-0.08, 0.37)	8.47 (-8.90, 32.01)	19.25 (-20.21, 72.72)
	90%	0.03 (-0.02, 0.09)	0.02 (-0.02, 0.08)	1 (-1, 2)	0.07 (-0.07, 0.30)	0.16 (-0.15, 0.67)	8.47 (-8.90, 32.01)	19.25 (-20.21, 72.72)

References for Data Inputs

[1] Blencowe H, Krasevec J, Onis M De, et al. Articles National , regional , and worldwide estimates of low birthweight in 2015 , with trends from 2000: a systematic analysis. Lancet Glob Heal. 2019;(18):1-12.
[2] Chawanpaiboon S, Vogel JP, Moller AB, et al. Global, regional, and national estimates of levels of preterm birth in 2014: a systematic review and modelling analysis. Lancet Glob Heal. 2019;7(1):e37-e46.
[3] United National Population Division World Population Prospects 2019.
[4] Fink G, Peet E, Danaei G, et al. Schooling and wage income losses due to early-childhood growth faltering in developing countries: National, regional, and global estimates. Am J Clin Nutr. 2016;104(1):104-112.
[5] Country specific annual wage data from World Indicators Database. Average yearly wage was estimated to be 2/3 of the gross domestic product in 2010 constant US dollars and 2011 International dollars, adjusted for purchasing power parity.
[6] NCD Risk Factor Collaboration. Trends in adult body-mass index in 200 countries from 1975 to 2014: a pooled analysis of 1698 population-based measurement studies with 19·2 million participants. Lancet. 2016;387(10026):1377-1396.
[7] Stevens GA, Finucane MM, De-Regil LM, et al. Global, regional, and national trends in haemoglobin concentration and prevalence of total and severe anaemia in children and pregnant and non-pregnant women for 1995-2011: A systematic analysis of population-representative data. Lancet Glob Heal. 2013;1(1):16-25.
[8] Coverage of iron-folic acid supplementation abstracted from the most recent Demographic Health Survey or imputed based on sub-regional average. Indicator used: % women in the past five years who took iron tablets or syrup for >90 days.

Ghana

Region: Sub-Saharan Africa**; Sub-region:** Western Sub-Saharan Africa

Low birthweight prevalence[1]: 14.2% (95% CI: 11.3, 18.5)

Preterm birth prevalence[2]: 12.0% (95% CI: 8.6, 16.7)

Number of births[3]: 4,356,000

Returns to education[4]: 5.4% (95% CI: 3.8, 7.0)

GDP per capita 2010 US$ (estimated annual wage)[5]: $1625 ($1083/year)

GDP per capita 2011 International $ (estimated annual wage)[5]: $3787 ($2525/year)

Prevalence of low BMI[6]: 6.9% (95% CI: 4.1, 10.7)

Prevalence of anemia[7]: 62.3% (95% CI: 52.3, 69.2)

Baseline coverage of IFA[8]: 59.4%

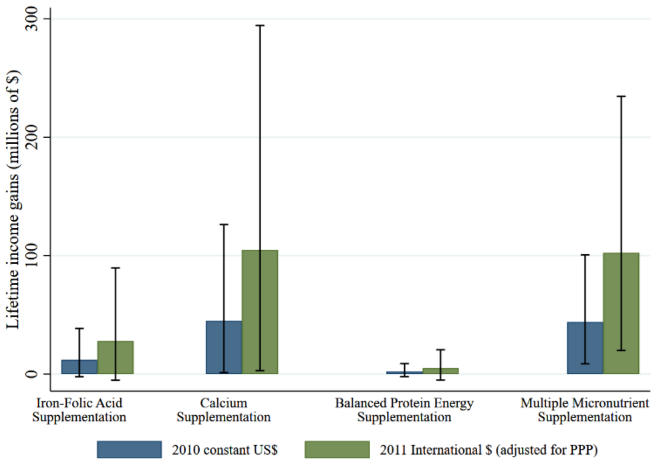

Figure: Benefits by birth cohort size for estimated gains in lifetime wages attributable to scaling up nutrition interventions to 90% target coverage

Table: Impact of maternal prenatal nutrition intervention on human capital and labour market outcomes, through improvements in low birthweight or preterm birth and schooling in Ghana.

Intervention	Target Coverage (%)	Absolute reduction in birth outcome (%)	Benefits by cohorts: School years gained (in 1000 s)	No. of additional students completing secondary school	Benefits by cohorts: Lifetime wages		Returns in lifetime earnings per child born to a targeted pregnant woman	
					in US $ millions	in International $ millions	in US $	in International $
Iron/Folic Acid Suppl.	90%	0.76 (-0.14, 1.69)	7.58 (-1.17, 22.78)	500 (-77, 1503)	12.00 (-2.21, 38.44)	27.97 (-5.15, 89.60)	3.06 (-0.56, 9.81)	7.13 (-1.31, 22.86)
Calcium Suppl.	50%	1.38 (0.27, 2.58)	16.08 (0.50, 39.57)	1061 (33, 2612)	24.97 (0.68, 70.14)	58.21 (1.59, 163.48)	11.47 (0.31, 32.20)	26.73 (0.73, 75.06)
	90%	2.48 (0.49, 4.64)	28.95 (0.90, 71.23)	1911 (59, 4701)	44.95 (1.23, 126.25)	104.78 (2.87, 294.26)	11.47 (0.31, 32.20)	26.73 (0.73, 75.06)
Multiple Micronutrient Suppl.	50%	1.06 (0.63, 1.61)	11.92 (2.05, 27.75)	787 (135, 1832)	18.33 (3.19, 43.26)	42.72 (7.43, 100.83)	8.42 (1.46, 19.86)	19.61 (3.41, 46.29)
	90%	2.51 (1.49, 3.82)	27.68 (5.50, 64.73)	1827 (363, 4272)	43.89 (8.55, 100.59)	102.29 (19.93, 234.46)	11.19 (2.18, 25.66)	26.09 (5.08, 59.81)
Balanced Protein Suppl.	50%	0.08 (-0.07, 0.22)	0.78 (-0.77, 3.00)	51 (-51, 198)	1.20 (-1.19, 4.89)	2.80 (-2.78, 11.39)	8.27 (-8.13, 27.30)	19.28 (-18.95, 63.62)
	90%	0.15 (-0.12, 0.40)	1.40 (-1.38, 5.41)	93 (-91, 357)	2.16 (-2.15, 8.79)	5.04 (-5.00, 20.50)	8.27 (-8.13, 27.30)	19.28 (-18.95, 63.62)

References for Data Inputs

[1] Blencowe H, Krasevec J, Onis M De, et al. Articles National , regional , and worldwide estimates of low birthweight in 2015 , with trends from 2000: a systematic analysis. Lancet Glob Heal. 2019;(18):1-12.
[2] Chawanpaiboon S, Vogel JP, Moller AB, et al. Global, regional, and national estimates of levels of preterm birth in 2014: a systematic review and modelling analysis. Lancet Glob Heal. 2019;7(1):e37-e46.
[3] United National Population Division World Population Prospects 2019.
[4] Fink G, Peet E, Danaei G, et al. Schooling and wage income losses due to early-childhood growth faltering in developing countries: National, regional, and global estimates. Am J Clin Nutr. 2016;104(1):104-112.
[5] Country specific annual wage data from World Indicators Database. Average yearly wage was estimated to be 2/3 of the gross domestic product in 2010 constant US dollars and 2011 International dollars, adjusted for purchasing power parity.
[6] NCD Risk Factor Collaboration. Trends in adult body-mass index in 200 countries from 1975 to 2014: a pooled analysis of 1698 population-based measurement studies with 19·2 million participants. Lancet. 2016;387(10026):1377-1396.
[7] Stevens GA, Finucane MM, De-Regil LM, et al. Global, regional, and national trends in haemoglobin concentration and prevalence of total and severe anaemia in children and pregnant and non-pregnant women for 1995-2011: A systematic analysis of population-representative data. Lancet Glob Heal. 2013;1(1):16-25.
[8] Coverage of iron-folic acid supplementation abstracted from the most recent Demographic Health Survey or imputed based on sub-regional average. Indicator used: % women in the past five years who took iron tablets or syrup for >90 days.

Grenada

Region: Latin America and Caribbean**; Sub-region:** Caribbean

Low birthweight prevalence[1]: 7.5% (95% CI: 3.8, 11.3)

Preterm birth prevalence[2]: 9.8% (95% CI: 8.6, 16.7)

Number of births[3]: 9,000

Returns to education[4]: 9.8% (95% CI: 8.1, 11.5)

GDP per capita 2010 US$ (estimated annual wage)[5]: $8194 ($5463/year)

GDP per capita 2011 International $ (estimated annual wage)[5]: $12436 ($8290/year)

Prevalence of low BMI[6]: 3.3% (95% CI: 1.2, 7.0)

Prevalence of anemia[7]: 31.5% (95% CI: 15.0, 55.3)

Baseline coverage of IFA[8]: 62.3%

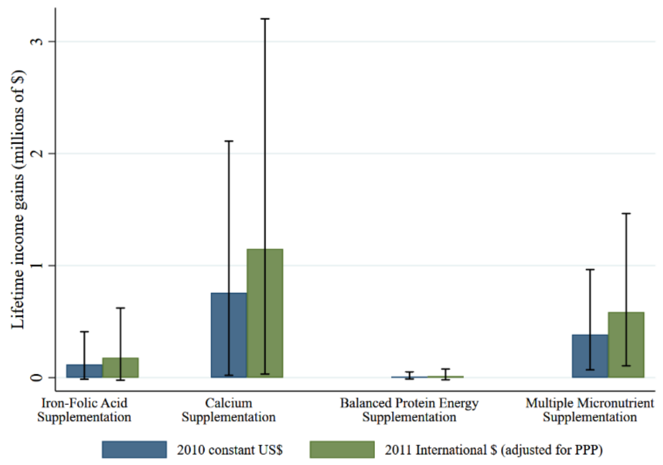

Figure: Benefits by birth cohort size for estimated gains in lifetime wages attributable to scaling up nutrition interventions to 90% target coverage

Table: Impact of maternal prenatal nutrition intervention on human capital and labour market outcomes, through improvements in low birthweight or preterm birth and schooling in Grenada.

Intervention	Target Coverage (%)	Absolute reduction in birth outcome (%)	Benefits by cohorts: School years gained (in 1000 s)	No. of additional students completing secondary school	Benefits by cohorts: Lifetime wages		Returns in lifetime earnings per child born to a targeted pregnant woman	
					in US $ millions	in International $ millions	in US $	in International $
Iron/Folic Acid Suppl.	90%	0.35 (-0.05, 0.92)	0.01 (-0.00, 0.03)	0 (-0, 1)	0.12 (-0.01, 0.41)	0.18 (-0.02, 0.62)	14.49 (-1.80, 50.59)	21.99 (-2.74, 76.78)
Calcium Suppl.	50%	1.13 (0.25, 2.25)	0.03 (0.00, 0.08)	2 (0, 4)	0.42 (0.01, 1.17)	0.64 (0.02, 1.78)	93.60 (2.66, 260.62)	142.04 (4.03, 395.51)
	90%	2.03 (0.44, 4.06)	0.05 (0.00, 0.14)	3 (0, 7)	0.76 (0.02, 2.11)	1.15 (0.03, 3.20)	93.60 (2.66, 260.62)	142.04 (4.03, 395.51)
Multiple Micronutrient Suppl.	50%	0.44 (0.17, 0.78)	0.01 (0.00, 0.03)	1 (0, 1)	0.15 (0.02, 0.40)	0.23 (0.04, 0.61)	34.04 (5.55, 89.84)	51.65 (8.43, 136.35)
	90%	1.10 (0.47, 1.91)	0.03 (0.01, 0.07)	1 (0, 3)	0.39 (0.07, 0.97)	0.59 (0.11, 1.46)	47.62 (8.62, 119.16)	72.26 (13.08, 180.83)
Balanced Protein Suppl.	50%	0.02 (-0.02, 0.06)	0.00 (-0.00, 0.00)	0 (-0, 0)	0.01 (-0.01, 0.03)	0.01 (-0.01, 0.04)	43.90 (-40.70, 152.05)	66.62 (-61.76, 230.74)
	90%	0.03 (-0.03, 0.12)	0.00 (-0.00, 0.00)	0 (-0, 0)	0.01 (-0.01, 0.05)	0.01 (-0.02, 0.08)	43.90 (-40.70, 152.05)	66.62 (-61.76, 230.74)

References for Data Inputs

[1] Blencowe H, Krasevec J, Onis M De, et al. Articles National , regional , and worldwide estimates of low birthweight in 2015 , with trends from 2000: a systematic analysis. Lancet Glob Heal. 2019;(18):1-12.

[2] Chawanpaiboon S, Vogel JP, Moller AB, et al. Global, regional, and national estimates of levels of preterm birth in 2014: a systematic review and modelling analysis. Lancet Glob Heal. 2019;7(1):e37-e46.

[3] United National Population Division World Population Prospects 2019.

[4] Fink G, Peet E, Danaei G, et al. Schooling and wage income losses due to early-childhood growth faltering in developing countries: National, regional, and global estimates. Am J Clin Nutr. 2016;104(1):104-112.

[5] Country specific annual wage data from World Indicators Database. Average yearly wage was estimated to be 2/3 of the gross domestic product in 2010 constant US dollars and 2011 International dollars, adjusted for purchasing power parity.

[6] NCD Risk Factor Collaboration. Trends in adult body-mass index in 200 countries from 1975 to 2014: a pooled analysis of 1698 population-based measurement studies with 19·2 million participants. Lancet. 2016;387(10026):1377-1396.

[7] Stevens GA, Finucane MM, De-Regil LM, et al. Global, regional, and national trends in haemoglobin concentration and prevalence of total and severe anaemia in children and pregnant and non-pregnant women for 1995-2011: A systematic analysis of population-representative data. Lancet Glob Heal. 2013;1(1):16-25.

[8] Coverage of iron-folic acid supplementation abstracted from the most recent Demographic Health Survey or imputed based on sub-regional average. Indicator used: % women in the past five years who took iron tablets or syrup for >90 days.

Guatemala

Region: Latin America and Caribbean**; Sub-region:** Central Latin America

Low birthweight prevalence[1]: 11.0% (95% CI: 8.3, 14.0)

Preterm birth prevalence[2]: 9.8% (95% CI: 8.6, 11.3)

Number of births[3]: 2,115,000

Returns to education[4]: 9.3% (95% CI: 7.1, 11.6)

GDP per capita 2010 US$ (estimated annual wage)[5]: $3069 ($2046/year)

GDP per capita 2011 International $ (estimated annual wage)[5]: $7293 ($4862/year)

Prevalence of low BMI[6]: 1.7% (95% CI: 0.7, 3.4)

Prevalence of anemia[7]: 30.9% (95% CI: 16.3, 52.1)

Baseline coverage of IFA[8]: 29.3%

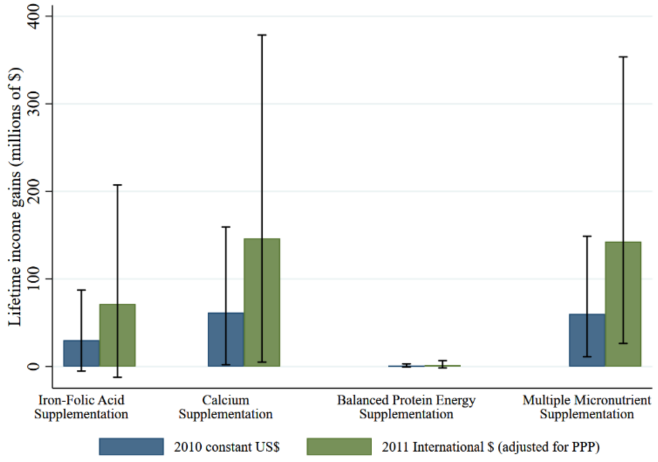

Figure: Benefits by birth cohort size for estimated gains in lifetime wages attributable to scaling up nutrition interventions to 90% target coverage

Table: Impact of maternal prenatal nutrition intervention on human capital and labour market outcomes, through improvements in low birthweight or preterm birth and schooling in Guatemala.

Intervention	Target Coverage (%)	Absolute reduction in birth outcome (%)	Benefits by cohorts: School years gained (in 1000 s)	No. of additional students completing secondary school	Benefits by cohorts: Lifetime wages		Returns in lifetime earnings per child born to a targeted pregnant woman	
					in US $ millions	in International $ millions	in US $	in International $
Iron/Folic Acid Suppl.	90%	1.10 (-0.18, 2.37)	5.85 (-0.92, 16.36)	192 (-30, 537)	30.05 (-5.17, 87.26)	71.40 (-12.28, 207.34)	15.79 (-2.71, 45.84)	37.51 (-6.45, 108.93)
Calcium Suppl.	50%	1.16 (0.26, 1.97)	6.87 (0.21, 16.94)	225 (7, 556)	34.19 (1.17, 88.50)	81.24 (2.77, 210.30)	32.33 (1.10, 83.69)	76.82 (2.62, 198.87)
	90%	2.10 (0.46, 3.55)	12.37 (0.37, 30.49)	406 (12, 1000)	61.54 (2.10, 159.31)	146.23 (4.99, 378.55)	32.33 (1.10, 83.69)	76.82 (2.62, 198.87)
Multiple Micronutrient Suppl.	50%	0.95 (0.46, 1.46)	5.24 (1.09, 12.08)	172 (36, 396)	27.05 (5.56, 65.25)	64.27 (13.20, 155.05)	25.58 (5.25, 61.70)	60.78 (12.49, 146.62)
	90%	2.16 (0.84, 3.47)	11.62 (2.31, 27.91)	381 (76, 916)	60.04 (11.11, 148.74)	142.67 (26.40, 353.45)	31.54 (5.84, 78.14)	74.95 (13.87, 185.68)
Balanced Protein Suppl.	50%	0.01 (-0.01, 0.05)	0.07 (-0.07, 0.30)	2 (-2, 10)	0.38 (-0.37, 1.57)	0.90 (-0.87, 3.73)	22.23 (-21.19, 71.89)	52.82 (-50.34, 170.82)
	90%	0.02 (-0.02, 0.08)	0.13 (-0.13, 0.54)	4 (-4, 18)	0.68 (-0.66, 2.82)	1.62 (-1.57, 6.71)	22.23 (-21.19, 71.89)	52.82 (-50.34, 170.82)

References for Data Inputs

[1] Blencowe H, Krasevec J, Onis M De, et al. Articles National , regional , and worldwide estimates of low birthweight in 2015 , with trends from 2000: a systematic analysis. Lancet Glob Heal. 2019;(18):1-12.
[2] Chawanpaiboon S, Vogel JP, Moller AB, et al. Global, regional, and national estimates of levels of preterm birth in 2014: a systematic review and modelling analysis. Lancet Glob Heal. 2019;7(1):e37-e46.
[3] United National Population Division World Population Prospects 2019.
[4] Fink G, Peet E, Danaei G, et al. Schooling and wage income losses due to early-childhood growth faltering in developing countries: National, regional, and global estimates. Am J Clin Nutr. 2016;104(1):104-112.
[5] Country specific annual wage data from World Indicators Database. Average yearly wage was estimated to be 2/3 of the gross domestic product in 2010 constant US dollars and 2011 International dollars, adjusted for purchasing power parity.
[6] NCD Risk Factor Collaboration. Trends in adult body-mass index in 200 countries from 1975 to 2014: a pooled analysis of 1698 population-based measurement studies with 19·2 million participants. Lancet. 2016;387(10026):1377-1396.
[7] Stevens GA, Finucane MM, De-Regil LM, et al. Global, regional, and national trends in haemoglobin concentration and prevalence of total and severe anaemia in children and pregnant and non-pregnant women for 1995-2011: A systematic analysis of population-representative data. Lancet Glob Heal. 2013;1(1):16-25.
[8] Coverage of iron-folic acid supplementation abstracted from the most recent Demographic Health Survey or imputed based on sub-regional average. Indicator used: % women in the past five years who took iron tablets or syrup for >90 days.

Guinea Bissau

Region: Sub-Saharan Africa**; Sub-region:** Western Sub-Saharan Africa

Low birthweight prevalence[1]: 21.1% (95% CI: 16.7, 27.4)

Preterm birth prevalence[2]: 12.0% (95% CI: 8.6, 16.7)

Number of births[3]: 328,000

Returns to education[4]: 6.3% (95% CI: 4.5, 8.1)

GDP per capita 2010 US$ (estimated annual wage)[5]: $574 ($383/year)

GDP per capita 2011 International $ (estimated annual wage)[5]: $1474 ($983/year)

Prevalence of low BMI[6]: 8.9% (95% CI: 3.6, 16.0)

Prevalence of anemia[7]: 50.0% (95% CI: 29.1, 67.8)

Baseline coverage of IFA[8]: 36.8%

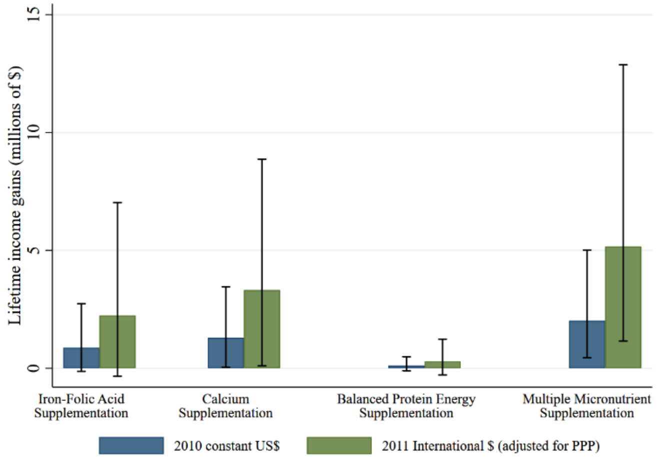

Figure: Benefits by birth cohort size for estimated gains in lifetime wages attributable to scaling up nutrition interventions to 90% target coverage

Table: Impact of maternal prenatal nutrition intervention on human capital and labour market outcomes, through improvements in low birthweight or preterm birth and schooling in Guinea Bissau.

Intervention	Target Coverage (%)	Absolute reduction in birth outcome (%)	Benefits by cohorts: School years gained (in 1000 s)	No. of additional students completing secondary school	Benefits by cohorts: Lifetime wages		Returns in lifetime earnings per child born to a targeted pregnant woman	
					in US $ millions	in International $ millions	in US $	in International $
Iron/Folic Acid Suppl.	90%	1.92 (-0.33, 4.00)	1.37 (-0.23, 4.01)	50 (-8, 148)	0.87 (-0.13, 2.74)	2.24 (-0.34, 7.03)	2.96 (-0.44, 9.28)	7.59 (-1.14, 23.81)
Calcium Suppl.	50%	1.42 (0.30, 2.57)	1.15 (0.03, 2.85)	42 (1, 105)	0.72 (0.02, 1.92)	1.84 (0.06, 4.93)	4.38 (0.13, 11.71)	11.23 (0.34, 30.05)
	90%	2.55 (0.54, 4.63)	2.07 (0.06, 5.12)	76 (2, 189)	1.29 (0.04, 3.46)	3.31 (0.10, 8.87)	4.38 (0.13, 11.71)	11.23 (0.34, 30.05)
Multiple Micronutrient Suppl.	50%	1.77 (1.04, 2.58)	1.38 (0.31, 3.04)	51 (12, 112)	0.87 (0.19, 2.08)	2.23 (0.50, 5.33)	5.31 (1.18, 12.67)	13.63 (3.04, 32.50)
	90%	4.25 (2.08, 6.59)	3.22 (0.68, 7.38)	119 (25, 272)	2.01 (0.45, 5.02)	5.17 (1.15, 12.87)	6.82 (1.52, 16.99)	17.50 (3.91, 43.61)
Balanced Protein Suppl.	50%	0.15 (-0.13, 0.45)	0.10 (-0.09, 0.40)	4 (-3, 15)	0.06 (-0.06, 0.27)	0.16 (-0.16, 0.69)	4.66 (-4.54, 16.20)	11.97 (-11.66, 41.58)
	90%	0.26 (-0.23, 0.80)	0.18 (-0.16, 0.72)	7 (-6, 27)	0.11 (-0.11, 0.48)	0.29 (-0.29, 1.24)	4.66 (-4.54, 16.20)	11.97 (-11.66, 41.58)

References for Data Inputs

[1] Blencowe H, Krasevec J, Onis M De, et al. Articles National , regional , and worldwide estimates of low birthweight in 2015 , with trends from 2000: a systematic analysis. Lancet Glob Heal. 2019;(18):1-12.
[2] Chawanpaiboon S, Vogel JP, Moller AB, et al. Global, regional, and national estimates of levels of preterm birth in 2014: a systematic review and modelling analysis. Lancet Glob Heal. 2019;7(1):e37-e46.
[3] United National Population Division World Population Prospects 2019.
[4] Fink G, Peet E, Danaei G, et al. Schooling and wage income losses due to early-childhood growth faltering in developing countries: National, regional, and global estimates. Am J Clin Nutr. 2016;104(1):104-112.
[5] Country specific annual wage data from World Indicators Database. Average yearly wage was estimated to be 2/3 of the gross domestic product in 2010 constant US dollars and 2011 International dollars, adjusted for purchasing power parity.
[6] NCD Risk Factor Collaboration. Trends in adult body-mass index in 200 countries from 1975 to 2014: a pooled analysis of 1698 population-based measurement studies with 19·2 million participants. Lancet. 2016;387(10026):1377-1396.
[7] Stevens GA, Finucane MM, De-Regil LM, et al. Global, regional, and national trends in haemoglobin concentration and prevalence of total and severe anaemia in children and pregnant and non-pregnant women for 1995-2011: A systematic analysis of population-representative data. Lancet Glob Heal. 2013;1(1):16-25.
[8] Coverage of iron-folic acid supplementation abstracted from the most recent Demographic Health Survey or imputed based on sub-regional average. Indicator used: % women in the past five years who took iron tablets or syrup for >90 days.

Guinea

Region: Sub-Saharan Africa**; Sub-region:** Western Sub-Saharan Africa

Low birthweight prevalence[1]: 12.2% (95% CI: 6.8, 17.6)

Preterm birth prevalence[2]: 12.0% (95% CI: 8.6, 16.7)

Number of births[3]: 2,245,000

Returns to education[4]: 6.3% (95% CI: 4.5, 8.1)

GDP per capita 2010 US$ (estimated annual wage)[5]: $750 ($500/year)

GDP per capita 2011 International $ (estimated annual wage)[5]: $1860 ($1240/year)

Prevalence of low BMI[6]: 10.0% (95% CI: 5.0, 16.6)

Prevalence of anemia[7]: 61.1% (95% CI: 53.3, 68.0)

Baseline coverage of IFA[8]: 41.5%

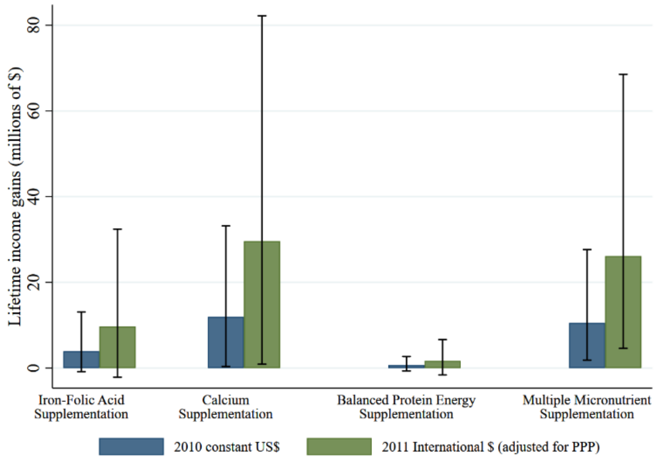

Figure: Benefits by birth cohort size for estimated gains in lifetime wages attributable to scaling up nutrition interventions to 90% target coverage

Table: Impact of maternal prenatal nutrition intervention on human capital and labour market outcomes, through improvements in low birthweight or preterm birth and schooling in Guinea.

Intervention	Target Coverage (%)	Absolute reduction in birth outcome (%)	Benefits by cohorts: School years gained (in 1000 s)	No. of additional students completing secondary school	Benefits by cohorts: Lifetime wages		Returns in lifetime earnings per child born to a targeted pregnant woman	
					in US $ millions	in International $ millions	in US $	in International $
**Iron/Folic Acid Suppl.**	90%	0.94 (-0.19, 2.36)	4.75 (-0.91, 14.81)	202 (-39, 631)	3.91 (-0.86, 13.08)	9.70 (-2.13, 32.42)	1.94 (-0.42, 6.47)	4.80 (-1.05, 16.04)
**Calcium Suppl.**	50%	1.45 (0.29, 2.55)	7.95 (0.24, 21.75)	339 (10, 927)	6.64 (0.20, 18.43)	16.45 (0.50, 45.68)	5.91 (0.18, 16.42)	14.66 (0.44, 40.69)
	90%	2.60 (0.53, 4.60)	14.31 (0.42, 39.16)	609 (18, 1668)	11.95 (0.36, 33.17)	29.62 (0.90, 82.22)	5.91 (0.18, 16.42)	14.66 (0.44, 40.69)
**Multiple Micronutrient Suppl.**	50%	0.95 (0.46, 1.57)	5.28 (0.91, 12.77)	225 (39, 544)	4.38 (0.69, 11.57)	10.85 (1.71, 28.67)	3.90 (0.62, 10.31)	9.66 (1.53, 25.54)
	90%	2.39 (0.98, 4.18)	12.87 (2.51, 30.24)	548 (107, 1288)	10.54 (1.85, 27.66)	26.12 (4.59, 68.55)	5.22 (0.92, 13.69)	12.93 (2.27, 33.93)
**Balanced Protein Suppl.**	50%	0.09 (-0.08, 0.29)	0.44 (-0.42, 1.86)	19 (-18, 79)	0.38 (-0.36, 1.49)	0.93 (-0.90, 3.68)	3.47 (-3.14, 12.76)	8.61 (-7.79, 31.63)
	90%	0.16 (-0.14, 0.51)	0.79 (-0.75, 3.34)	34 (-32, 142)	0.68 (-0.65, 2.67)	1.68 (-1.62, 6.63)	3.47 (-3.14, 12.76)	8.61 (-7.79, 31.63)

References for Data Inputs

[1] Blencowe H, Krasevec J, Onis M De, et al. Articles National , regional , and worldwide estimates of low birthweight in 2015 , with trends from 2000: a systematic analysis. Lancet Glob Heal. 2019;(18):1-12.

[2] Chawanpaiboon S, Vogel JP, Moller AB, et al. Global, regional, and national estimates of levels of preterm birth in 2014: a systematic review and modelling analysis. Lancet Glob Heal. 2019;7(1):e37-e46.

[3] United National Population Division World Population Prospects 2019.

[4] Fink G, Peet E, Danaei G, et al. Schooling and wage income losses due to early-childhood growth faltering in developing countries: National, regional, and global estimates. Am J Clin Nutr. 2016;104(1):104-112.

[5] Country specific annual wage data from World Indicators Database. Average yearly wage was estimated to be 2/3 of the gross domestic product in 2010 constant US dollars and 2011 International dollars, adjusted for purchasing power parity.

[6] NCD Risk Factor Collaboration. Trends in adult body-mass index in 200 countries from 1975 to 2014: a pooled analysis of 1698 population-based measurement studies with 19·2 million participants. Lancet. 2016;387(10026):1377-1396.

[7] Stevens GA, Finucane MM, De-Regil LM, et al. Global, regional, and national trends in haemoglobin concentration and prevalence of total and severe anaemia in children and pregnant and non-pregnant women for 1995-2011: A systematic analysis of population-representative data. Lancet Glob Heal. 2013;1(1):16-25.

[8] Coverage of iron-folic acid supplementation abstracted from the most recent Demographic Health Survey or imputed based on sub-regional average. Indicator used: % women in the past five years who took iron tablets or syrup for >90 days.

Guyana

Region: Latin America and Caribbean**; Sub-region:** Caribbean

Low birthweight prevalence[1]: 15.6% (95% CI: 12.2, 19.6)

Preterm birth prevalence[2]: 9.8% (95% CI: 8.6, 11.3)

Number of births[3]: 78,000

Returns to education[4]: 9.8% (95% CI: 8.1, 11.5)

GDP per capita 2010 US$ (estimated annual wage)[5]: $3690 ($2460/year)

GDP per capita 2011 International $ (estimated annual wage)[5]: $7087 ($4725/year)

Prevalence of low BMI[6]: 4.3% (95% CI: 1.5, 8.9)

Prevalence of anemia[7]: 30.9% (95% CI: 22.7, 41.6)

Baseline coverage of IFA[8]: 62.3%

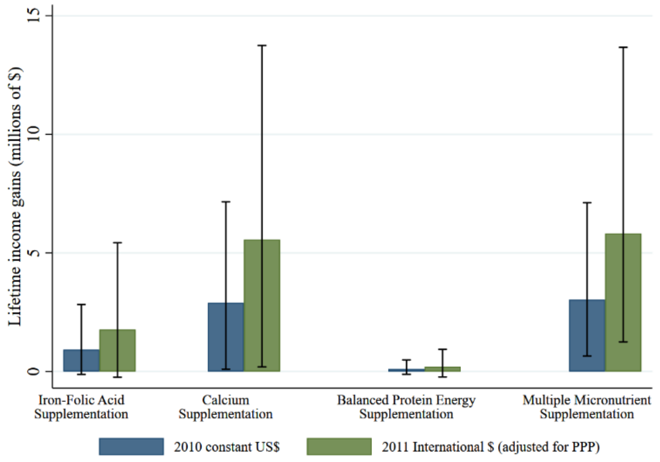

Figure: Benefits by birth cohort size for estimated gains in lifetime wages attributable to scaling up nutrition interventions to 90% target coverage

Table: Impact of maternal prenatal nutrition intervention on human capital and labour market outcomes, through improvements in low birthweight or preterm birth and schooling in Guyana.

Intervention	Target Coverage (%)	Absolute reduction in birth outcome (%)	Benefits by cohorts: School years gained (in 1000 s)	No. of additional students completing secondary school	Benefits by cohorts: Lifetime wages		Returns in lifetime earnings per child born to a targeted pregnant woman	
					in US $ millions	in International $ millions	in US $	in International $
Iron/Folic Acid Suppl.	90%	0.76 (-0.13, 1.67)	0.15 (-0.02, 0.43)	8 (-1, 25)	0.92 (-0.12, 2.83)	1.77 (-0.24, 5.43)	13.11 (-1.77, 40.25)	25.18 (-3.40, 77.30)
Calcium Suppl.	50%	1.16 (0.27, 1.98)	0.25 (0.01, 0.60)	14 (0, 35)	1.61 (0.06, 3.98)	3.09 (0.11, 7.64)	41.28 (1.42, 101.95)	79.27 (2.72, 195.80)
	90%	2.09 (0.49, 3.56)	0.44 (0.01, 1.07)	26 (1, 62)	2.90 (0.10, 7.16)	5.57 (0.19, 13.74)	41.28 (1.42, 101.95)	79.27 (2.72, 195.80)
Multiple Micronutrient Suppl.	50%	0.94 (0.46, 1.42)	0.19 (0.04, 0.44)	11 (2, 25)	1.23 (0.25, 2.92)	2.37 (0.49, 5.61)	31.63 (6.52, 74.94)	60.74 (12.52, 143.92)
	90%	2.33 (1.24, 3.57)	0.47 (0.10, 1.06)	28 (6, 62)	3.03 (0.65, 7.12)	5.82 (1.25, 13.66)	43.17 (9.26, 101.35)	82.91 (17.77, 194.65)
Balanced Protein Suppl.	50%	0.05 (-0.04, 0.17)	0.01 (-0.01, 0.04)	1 (-1, 2)	0.06 (-0.07, 0.27)	0.11 (-0.13, 0.52)	39.91 (-37.58, 123.02)	76.65 (-72.17, 236.27)
	90%	0.09 (-0.08, 0.30)	0.02 (-0.02, 0.07)	1 (-1, 4)	0.11 (-0.12, 0.49)	0.20 (-0.23, 0.94)	39.91 (-37.58, 123.02)	76.65 (-72.17, 236.27)

References for Data Inputs
[1] Blencowe H, Krasevec J, Onis M De, et al. Articles National , regional , and worldwide estimates of low birthweight in 2015 , with trends from 2000: a systematic analysis. Lancet Glob Heal. 2019;(18):1-12.
[2] Chawanpaiboon S, Vogel JP, Moller AB, et al. Global, regional, and national estimates of levels of preterm birth in 2014: a systematic review and modelling analysis. Lancet Glob Heal. 2019;7(1):e37-e46.
[3] United National Population Division World Population Prospects 2019.
[4] Fink G, Peet E, Danaei G, et al. Schooling and wage income losses due to early-childhood growth faltering in developing countries: National, regional, and global estimates. Am J Clin Nutr. 2016;104(1):104-112.
[5] Country specific annual wage data from World Indicators Database. Average yearly wage was estimated to be 2/3 of the gross domestic product in 2010 constant US dollars and 2011 International dollars, adjusted for purchasing power parity.
[6] NCD Risk Factor Collaboration. Trends in adult body-mass index in 200 countries from 1975 to 2014: a pooled analysis of 1698 population-based measurement studies with 19·2 million participants. Lancet. 2016;387(10026):1377-1396.
[7] Stevens GA, Finucane MM, De-Regil LM, et al. Global, regional, and national trends in haemoglobin concentration and prevalence of total and severe anaemia in children and pregnant and non-pregnant women for 1995-2011: A systematic analysis of population-representative data. Lancet Glob Heal. 2013;1(1):16-25.
[8] Coverage of iron-folic acid supplementation abstracted from the most recent Demographic Health Survey or imputed based on sub-regional average. Indicator used: % women in the past five years who took iron tablets or syrup for >90 days.

Haiti

Region: Latin America and Caribbean**; Sub-region:** Caribbean

Low birthweight prevalence[1]: 24.6% (95% CI: 20.9, 28.4)

Preterm birth prevalence[2]: 9.8% (95% CI: 8.6, 11.3)

Number of births[3]: 1,355,000

Returns to education[4]: 9.8% (95% CI: 8.1, 11.5)

GDP per capita 2010 US$ (estimated annual wage)[5]: $729 ($486/year)

GDP per capita 2011 International $ (estimated annual wage)[5]: $1654 ($1102/year)

Prevalence of low BMI[6]: 4.7% (95% CI: 2.0, 9.1)

Prevalence of anemia[7]: 47.9% (95% CI: 36.9, 58.2)

Baseline coverage of IFA[8]: 43.2%

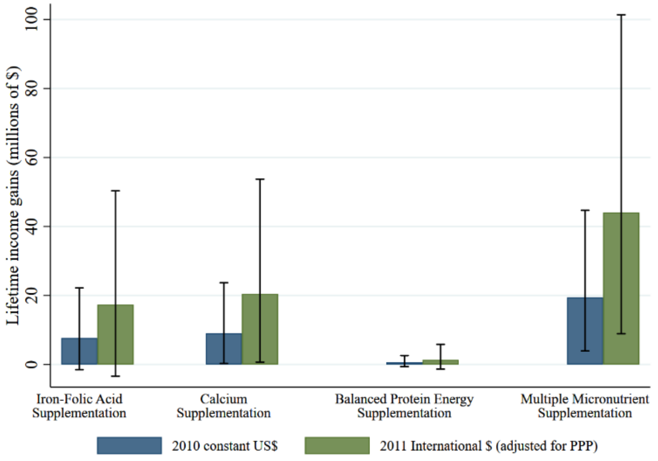

Figure: Benefits by birth cohort size for estimated gains in lifetime wages attributable to scaling up nutrition interventions to 90% target coverage

Table: Impact of maternal prenatal nutrition intervention on human capital and labour market outcomes, through improvements in low birthweight or preterm birth and schooling in Haiti.

Intervention	Target Coverage (%)	Absolute reduction in birth outcome (%)	Benefits by cohorts: School years gained (in 1000 s)	No. of additional students completing secondary school	Benefits by cohorts: Lifetime wages		Returns in lifetime earnings per child born to a targeted pregnant woman	
					in US $ millions	in International $ millions	in US $	in International $
Iron/Folic Acid Suppl.	90%	1.99 (-0.36, 4.11)	5.93 (-1.01, 16.72)	109 (-19, 308)	7.66 (-1.49, 22.19)	17.37 (-3.38, 50.32)	6.28 (-1.22, 18.19)	14.24 (-2.77, 41.26)
Calcium Suppl.	50%	1.17 (0.25, 2.04)	3.99 (0.12, 9.71)	73 (2, 179)	5.02 (0.17, 13.16)	11.39 (0.39, 29.85)	7.42 (0.25, 19.43)	16.82 (0.57, 44.06)
	90%	2.10 (0.44, 3.67)	7.17 (0.21, 17.47)	132 (4, 322)	9.04 (0.31, 23.69)	20.51 (0.70, 53.74)	7.42 (0.25, 19.43)	16.82 (0.57, 44.06)
Multiple Micronutrient Suppl.	50%	1.80 (1.14, 2.48)	6.02 (1.25, 12.67)	111 (23, 233)	7.69 (1.68, 17.09)	17.45 (3.82, 38.76)	11.35 (2.49, 25.22)	25.75 (5.64, 57.21)
	90%	4.70 (2.48, 6.99)	15.13 (3.26, 33.34)	278 (60, 613)	19.43 (3.92, 44.70)	44.06 (8.90, 101.38)	15.93 (3.22, 36.66)	36.13 (7.30, 83.14)
Balanced Protein Suppl.	50%	0.09 (-0.07, 0.27)	0.26 (-0.27, 1.13)	5 (-5, 21)	0.33 (-0.33, 1.42)	0.75 (-0.75, 3.23)	11.39 (-11.03, 37.06)	25.83 (-25.03, 84.06)
	90%	0.16 (-0.13, 0.49)	0.47 (-0.49, 2.03)	9 (-9, 37)	0.60 (-0.59, 2.56)	1.35 (-1.35, 5.82)	11.39 (-11.03, 37.06)	25.83 (-25.03, 84.06)

References for Data Inputs

[1] Blencowe H, Krasevec J, Onis M De, et al. Articles National , regional , and worldwide estimates of low birthweight in 2015 , with trends from 2000: a systematic analysis. Lancet Glob Heal. 2019;(18):1-12.
[2] Chawanpaiboon S, Vogel JP, Moller AB, et al. Global, regional, and national estimates of levels of preterm birth in 2014: a systematic review and modelling analysis. Lancet Glob Heal. 2019;7(1):e37-e46.
[3] United National Population Division World Population Prospects 2019.
[4] Fink G, Peet E, Danaei G, et al. Schooling and wage income losses due to early-childhood growth faltering in developing countries: National, regional, and global estimates. Am J Clin Nutr. 2016;104(1):104-112.
[5] Country specific annual wage data from World Indicators Database. Average yearly wage was estimated to be 2/3 of the gross domestic product in 2010 constant US dollars and 2011 International dollars, adjusted for purchasing power parity.
[6] NCD Risk Factor Collaboration. Trends in adult body-mass index in 200 countries from 1975 to 2014: a pooled analysis of 1698 population-based measurement studies with 19·2 million participants. Lancet. 2016;387(10026):1377-1396.
[7] Stevens GA, Finucane MM, De-Regil LM, et al. Global, regional, and national trends in haemoglobin concentration and prevalence of total and severe anaemia in children and pregnant and non-pregnant women for 1995-2011: A systematic analysis of population-representative data. Lancet Glob Heal. 2013;1(1):16-25.
[8] Coverage of iron-folic acid supplementation abstracted from the most recent Demographic Health Survey or imputed based on sub-regional average. Indicator used: % women in the past five years who took iron tablets or syrup for >90 days.

Honduras

Region: Latin America and Caribbean**; Sub-region:** Central Latin America

Low birthweight prevalence[1]: 10.9% (95% CI: 8.6, 13.8)

Preterm birth prevalence[2]: 9.8% (95% CI: 8.6, 11.3)

Number of births[3]: 1,037,000

Returns to education[4]: 11.1% (95% CI: 3.7, 18.4)

GDP per capita 2010 US$ (estimated annual wage)[5]: $2053 ($1369/year)

GDP per capita 2011 International $ (estimated annual wage)[5]: $4247 ($2832/year)

Prevalence of low BMI[6]: 2.5% (95% CI: 1.0, 4.8)

Prevalence of anemia[7]: 21.9% (95% CI: 12.4, 36.3)

Baseline coverage of IFA[8]: 37.4%

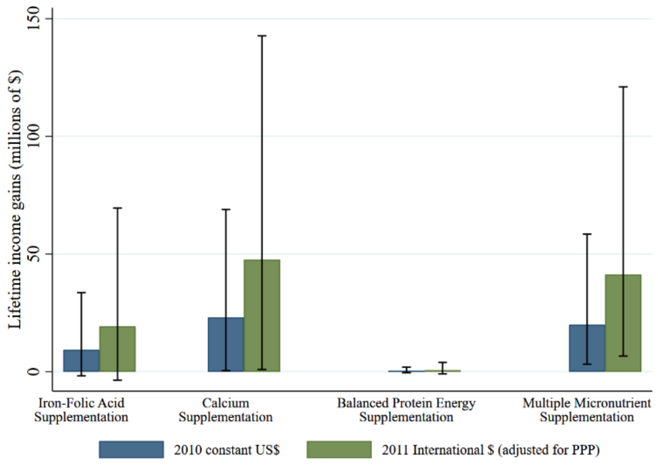

Figure: Benefits by birth cohort size for estimated gains in lifetime wages attributable to scaling up nutrition interventions to 90% target coverage

Table: Impact of maternal prenatal nutrition intervention on human capital and labour market outcomes, through improvements in low birthweight or preterm birth and schooling in Honduras.

Intervention	Target Coverage (%)	Absolute reduction in birth outcome (%)	Benefits by cohorts: School years gained (in 1000 s)	No. of additional students completing secondary school	Benefits by cohorts: Lifetime wages		Returns in lifetime earnings per child born to a targeted pregnant woman	
					in US $ millions	in International $ millions	in US $	in International $
Iron/Folic Acid Suppl.	90%	0.97 (-0.17, 2.08)	2.53 (-0.41, 7.10)	108 (-17, 303)	9.31 (-1.73, 33.62)	19.26 (-3.59, 69.56)	9.97 (-1.86, 36.02)	20.63 (-3.85, 74.53)
Calcium Suppl.	50%	1.17 (0.26, 1.95)	3.38 (0.11, 8.11)	144 (5, 345)	12.80 (0.26, 38.33)	26.47 (0.54, 79.30)	24.68 (0.50, 73.93)	51.06 (1.04, 152.94)
	90%	2.11 (0.46, 3.50)	6.09 (0.19, 14.60)	259 (8, 622)	23.03 (0.47, 68.99)	47.65 (0.97, 142.74)	24.68 (0.50, 73.93)	51.06 (1.04, 152.94)
Multiple Micronutrient Suppl.	50%	0.77 (0.41, 1.16)	2.15 (0.47, 4.66)	92 (20, 198)	8.17 (1.37, 22.16)	16.90 (2.84, 45.84)	15.75 (2.65, 42.74)	32.59 (5.48, 88.41)
	90%	1.94 (0.78, 3.11)	5.25 (1.02, 12.09)	224 (44, 515)	19.96 (3.20, 58.51)	41.29 (6.62, 121.05)	21.39 (3.43, 62.69)	44.24 (7.09, 129.70)
Balanced Protein Suppl.	50%	0.02 (-0.02, 0.07)	0.05 (-0.06, 0.23)	2 (-2, 10)	0.18 (-0.24, 1.06)	0.37 (-0.50, 2.19)	16.31 (-18.29, 64.57)	33.75 (-37.84, 133.58)
	90%	0.04 (-0.03, 0.12)	0.09 (-0.10, 0.41)	4 (-4, 17)	0.32 (-0.43, 1.91)	0.67 (-0.89, 3.95)	16.31 (-18.29, 64.57)	33.75 (-37.84, 133.58)

References for Data Inputs

[1] Blencowe H, Krasevec J, Onis M De, et al. Articles National , regional , and worldwide estimates of low birthweight in 2015 , with trends from 2000: a systematic analysis. Lancet Glob Heal. 2019;(18):1-12.

[2] Chawanpaiboon S, Vogel JP, Moller AB, et al. Global, regional, and national estimates of levels of preterm birth in 2014: a systematic review and modelling analysis. Lancet Glob Heal. 2019;7(1):e37-e46.

[3] United National Population Division World Population Prospects 2019.

[4] Fink G, Peet E, Danaei G, et al. Schooling and wage income losses due to early-childhood growth faltering in developing countries: National, regional, and global estimates. Am J Clin Nutr. 2016;104(1):104-112.

[5] Country specific annual wage data from World Indicators Database. Average yearly wage was estimated to be 2/3 of the gross domestic product in 2010 constant US dollars and 2011 International dollars, adjusted for purchasing power parity.

[6] NCD Risk Factor Collaboration. Trends in adult body-mass index in 200 countries from 1975 to 2014: a pooled analysis of 1698 population-based measurement studies with 19·2 million participants. Lancet. 2016;387(10026):1377-1396.

[7] Stevens GA, Finucane MM, De-Regil LM, et al. Global, regional, and national trends in haemoglobin concentration and prevalence of total and severe anaemia in children and pregnant and non-pregnant women for 1995-2011: A systematic analysis of population-representative data. Lancet Glob Heal. 2013;1(1):16-25.

[8] Coverage of iron-folic acid supplementation abstracted from the most recent Demographic Health Survey or imputed based on sub-regional average. Indicator used: % women in the past five years who took iron tablets or syrup for >90 days.

India

Region: South Asia**; Sub-region:** South Asia

Low birthweight prevalence[1]: 27.6% (95% CI: 18.1, 37.0)

Preterm birth prevalence[2]: 13.6% (95% CI: 11.1, 16.1)

Number of births[3]: 121,189,000

Returns to education[4]: 8.0% (95% CI: 7.5, 8.6)

GDP per capita 2010 US$ (estimated annual wage)[5]: $1752 ($1168/year)

GDP per capita 2011 International $ (estimated annual wage)[5]: $5743 ($3829/year)

Prevalence of low BMI[6]: 24.1% (95% CI: 19.1, 29.5)

Prevalence of anemia[7]: 53.9% (95% CI: 39.4, 66.0)

Baseline coverage of IFA[8]: 38.7%

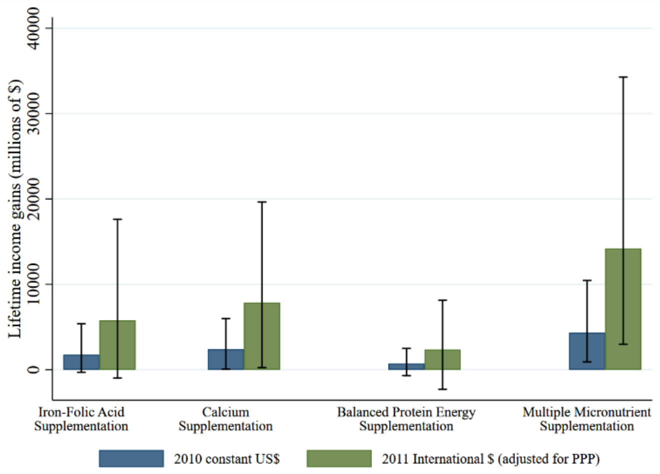

Figure: Benefits by birth cohort size for estimated gains in lifetime wages attributable to scaling up nutrition interventions to 90% target coverage

Table: Impact of maternal prenatal nutrition intervention on human capital and labour market outcomes, through improvements in low birthweight or preterm birth and schooling in India.

Intervention	Target Coverage (%)	Absolute reduction in birth outcome (%)	Benefits by cohorts: School years gained (in 1000 s)	No. of additional students completing secondary school	Benefits by cohorts: Lifetime wages		Returns in lifetime earnings per child born to a targeted pregnant woman	
					in US $ millions	in International $ millions	in US $	in International $
Iron/Folic Acid Suppl.	90%	2.38 (-0.44, 5.39)	702.44 (-118.48, 2102.51)	25007 (-4218, 74849)	1764.23 (-299.62, 5370)	5784.63 (-982, 17609)	16.18 (-2.75, 49.24)	53.04 (-9.01, 161)
Calcium Suppl.	50%	1.62 (0.35, 2.79)	529.29 (15.93, 1329.55)	18843 (567, 47332)	1331.41 (42.04, 3329)	4365.48 (138, 10916)	21.97 (0.69, 54.94)	72.04 (2.27, 180)
	90%	2.91 (0.63, 5.03)	952.72 (28.68, 2393.18)	33917 (1021, 85197)	2396.54 (75.66, 5993)	7857.86 (248, 19649)	21.97 (0.69, 54.94)	72.04 (2.27, 180)
Multiple Micronutrient Suppl.	50%	2.26 (1.25, 3.50)	717.71 (150.06, 1707.03)	25550 (5342, 60770)	1809.64 (378, 4384)	5933.51 (1238, 14373)	29.86 (6.23, 72.34)	97.92 (20.43, 237)
	90%	5.50 (2.54, 8.95)	1699.80 (361.22, 4153.42)	60513 (12859, 147862)	4332.43 (909, 10454)	14205.34 (2980, 34278)	39.72 (8.33, 95.85)	130.24 (27.33, 314)
Balanced Protein Suppl.	50%	0.56 (-0.44, 1.38)	158.71 (-154.54, 544.13)	5650 (-5501, 19371)	398.13 (-388, 1380)	1305.40 (-1273, 4524)	27.48 (-27.21, 89.01)	90.11 (-89.23, 292)
	90%	1.00 (-0.79, 2.48)	285.68 (-278.16, 979.44)	10170 (-9903, 34868)	716.63 (-699, 2483.55)	2349.72 (-2291, 8143)	27.48 (-27.21, 89.01)	90.11 (-89.23, 292)

References for Data Inputs

[1] Blencowe H, Krasevec J, Onis M De, et al. Articles National , regional , and worldwide estimates of low birthweight in 2015 , with trends from 2000: a systematic analysis. Lancet Glob Heal. 2019;(18):1-12.
[2] Chawanpaiboon S, Vogel JP, Moller AB, et al. Global, regional, and national estimates of levels of preterm birth in 2014: a systematic review and modelling analysis. Lancet Glob Heal. 2019;7(1):e37-e46.
[3] United National Population Division World Population Prospects 2019.
[4] Fink G, Peet E, Danaei G, et al. Schooling and wage income losses due to early-childhood growth faltering in developing countries: National, regional, and global estimates. Am J Clin Nutr. 2016;104(1):104-112.
[5] Country specific annual wage data from World Indicators Database. Average yearly wage was estimated to be 2/3 of the gross domestic product in 2010 constant US dollars and 2011 International dollars, adjusted for purchasing power parity.
[6] NCD Risk Factor Collaboration. Trends in adult body-mass index in 200 countries from 1975 to 2014: a pooled analysis of 1698 population-based measurement studies with 19·2 million participants. Lancet. 2016;387(10026):1377-1396.
[7] Stevens GA, Finucane MM, De-Regil LM, et al. Global, regional, and national trends in haemoglobin concentration and prevalence of total and severe anaemia in children and pregnant and non-pregnant women for 1995-2011: A systematic analysis of population-representative data. Lancet Glob Heal. 2013;1(1):16-25.
[8] Coverage of iron-folic acid supplementation abstracted from the most recent Demographic Health Survey or imputed based on sub-regional average. Indicator used: % women in the past five years who took iron tablets or syrup for >90 days.

Indonesia

Region: Southeast Asia, East Asia, and Oceania**; Sub-region:** Southeast Asia

Low birthweight prevalence[1]: 10.0% (95% CI: 7.4, 12.7)

Preterm birth prevalence[2]: 10.4% (95% CI: 8.7, 11.9)

Number of births[3]: 24,208,000

Returns to education[4]: 8.0% (95% CI: 2.8, 13.2)

GDP per capita 2010 US$ (estimated annual wage)[5]: $3824 ($2550/year)

GDP per capita 2011 International $ (estimated annual wage)[5]: $10359 ($6906/year)

Prevalence of low BMI[6]: 12.7% (95% CI: 7.8, 18.5)

Prevalence of anemia[7]: 30.4% (95% CI: 18.4, 50.7)

Baseline coverage of IFA[8]: 32.7%

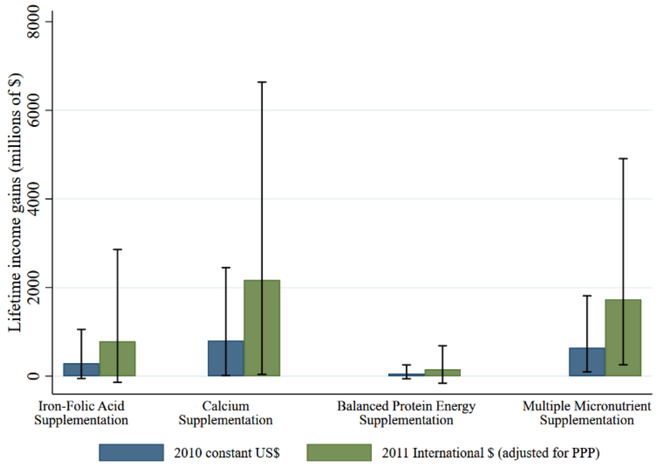

Figure: Benefits by birth cohort size for estimated gains in lifetime wages attributable to scaling up nutrition interventions to 90% target coverage

Table: Impact of maternal prenatal nutrition intervention on human capital and labour market outcomes, through improvements in low birthweight or preterm birth and schooling in Indonesia.

Intervention	Target Coverage (%)	Absolute reduction in birth outcome (%)	Benefits by cohorts: School years gained (in 1000 s)	No. of additional students completing secondary school	Benefits by cohorts: Lifetime wages		Returns in lifetime earnings per child born to a targeted pregnant woman	
					in US $ millions	in International $ millions	in US $	in International $
Iron/Folic Acid Suppl.	90%	0.95 (-0.18, 2.07)	56.19 (-8.45, 160.31)	2641 (-397, 7535)	290.16 (-52.06, 1055.71)	785.97 (-141.03, 2860)	13.32 (-2.39, 48.46)	36.07 (-6.47, 131)
Calcium Suppl.	50%	1.26 (0.27, 2.14)	85.33 (2.48, 207.37)	4011 (117, 9746)	445.17 (8.07, 1361.46)	1205.84 (21.85, 3688)	36.78 (0.67, 112.48)	99.62 (1.81, 305)
	90%	2.26 (0.49, 3.85)	153.60 (4.46, 373.27)	7219 (210, 17544)	801.31 (14.52, 2450.63)	2170.51 (39.33, 6638)	36.78 (0.67, 112.48)	99.62 (1.81, 305)
Multiple Micronutrient Suppl.	50%	0.82 (0.43, 1.22)	52.57 (11.05, 111.26)	2471 (519, 5229)	277.33 (44.83, 742.78)	751.20 (121.44, 2012)	22.91 (3.70, 61.37)	62.06 (10.03, 166)
	90%	1.89 (0.78, 3.12)	121.24 (25.17, 275.11)	5698 (1183, 12930)	639.65 (95.21, 1812.65)	1732.62 (257.88, 4910)	29.36 (4.37, 83.20)	79.52 (11.84, 225)
Balanced Protein Suppl.	50%	0.10 (-0.09, 0.29)	5.92 (-6.03, 22.02)	278 (-283, 1035)	31.13 (-32.50, 140.40)	84.32 (-88.03, 380)	20.54 (-22.66, 83.92)	55.64 (-61.38, 227)
	90%	0.19 (-0.16, 0.53)	10.66 (-10.85, 39.63)	501 (-510, 1863)	56.03 (-58.50, 252.72)	151.77 (-158.45, 684)	20.54 (-22.66, 83.92)	55.64 (-61.38, 227)

References for Data Inputs

[1] Blencowe H, Krasevec J, Onis M De, et al. Articles National , regional , and worldwide estimates of low birthweight in 2015 , with trends from 2000: a systematic analysis. Lancet Glob Heal. 2019;(18):1-12.

[2] Chawanpaiboon S, Vogel JP, Moller AB, et al. Global, regional, and national estimates of levels of preterm birth in 2014: a systematic review and modelling analysis. Lancet Glob Heal. 2019;7(1):e37-e46.

[3] United National Population Division World Population Prospects 2019.

[4] Fink G, Peet E, Danaei G, et al. Schooling and wage income losses due to early-childhood growth faltering in developing countries: National, regional, and global estimates. Am J Clin Nutr. 2016;104(1):104-112.

[5] Country specific annual wage data from World Indicators Database. Average yearly wage was estimated to be 2/3 of the gross domestic product in 2010 constant US dollars and 2011 International dollars, adjusted for purchasing power parity.

[6] NCD Risk Factor Collaboration. Trends in adult body-mass index in 200 countries from 1975 to 2014: a pooled analysis of 1698 population-based measurement studies with 19·2 million participants. Lancet. 2016;387(10026):1377-1396.

[7] Stevens GA, Finucane MM, De-Regil LM, et al. Global, regional, and national trends in haemoglobin concentration and prevalence of total and severe anaemia in children and pregnant and non-pregnant women for 1995-2011: A systematic analysis of population-representative data. Lancet Glob Heal. 2013;1(1):16-25.

[8] Coverage of iron-folic acid supplementation abstracted from the most recent Demographic Health Survey or imputed based on sub-regional average. Indicator used: % women in the past five years who took iron tablets or syrup for >90 days.

Iran

Region: North Africa and Middle East**; Sub-region:** North Africa and Middle East

Low birthweight prevalence[1]: 7.2% (95% CI: 3.2, 11.2)

Preterm birth prevalence[2]: 6.0% (95% CI: 4.0, 8.7)

Number of births[3]: 7,758,000

Returns to education[4]: 7.8% (95% CI: 6.9, 8.8)

GDP per capita 2010 US$ (estimated annual wage)[5]: $6073 ($4049/year)

GDP per capita 2011 International $ (estimated annual wage)[5]: $16683 ($11122/year)

Prevalence of low BMI[6]: 3.6% (95% CI: 2.3, 5.2)

Prevalence of anemia[7]: 26.1% (95% CI: 14.9, 42.7)

Baseline coverage of IFA[8]: 24.3%

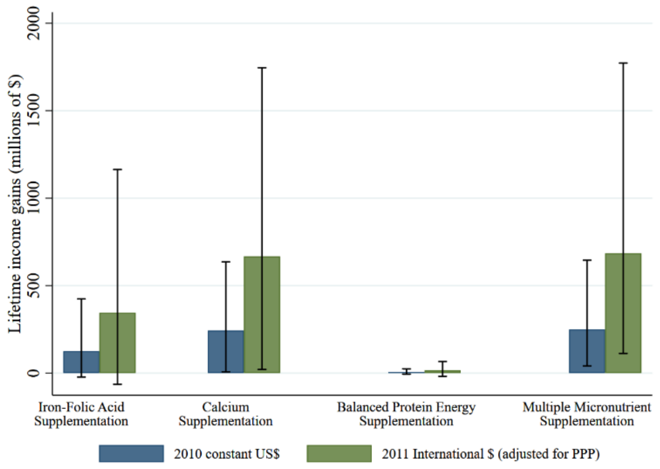

Figure: Benefits by birth cohort size for estimated gains in lifetime wages attributable to scaling up nutrition interventions to 90% target coverage

Table: Impact of maternal prenatal nutrition intervention on human capital and labour market outcomes, through improvements in low birthweight or preterm birth and schooling in Iran.

Intervention	Target Coverage (%)	Absolute reduction in birth outcome (%)	Benefits by cohorts: School years gained (in 1000 s)	No. of additional students completing secondary school	Benefits by cohorts: Lifetime wages		Returns in lifetime earnings per child born to a targeted pregnant woman	
					in US $ millions	in International $ millions	in US $	in International $
**Iron/Folic Acid Suppl.**	90%	0.74 (-0.14, 1.88)	14.67 (-2.70, 49.35)	566 (-104, 1905)	125.65 (-23.25, 423.65)	345.16 (-63.86, 1163.73)	18.00 (-3.33, 60.68)	49.43 (-9.15, 167)
**Calcium Suppl.**	50%	0.70 (0.16, 1.30)	15.29 (0.47, 39.96)	590 (18, 1542)	134.88 (4.10, 353.04)	370.52 (11.27, 969.78)	34.77 (1.06, 91.01)	95.52 (2.91, 250)
	90%	1.25 (0.28, 2.34)	27.52 (0.84, 71.93)	1062 (33, 2776)	242.79 (7.38, 635.47)	666.93 (20.28, 1745.61)	34.77 (1.06, 91.01)	95.52 (2.91, 250)
**Multiple Micronutrient Suppl.**	50%	0.65 (0.25, 1.19)	13.41 (2.39, 33.91)	518 (92, 1309)	116.32 (20.19, 297.17)	319.53 (55.46, 816.31)	29.99 (5.20, 76.61)	82.38 (14.30, 210)
	90%	1.39 (0.44, 2.68)	28.66 (4.65, 72.89)	1106 (180, 2814)	249.23 (40.99, 645.20)	684.62 (112.59, 1772.32)	35.70 (5.87, 92.41)	98.05 (16.12, 254)
**Balanced Protein Suppl.**	50%	0.02 (-0.02, 0.06)	0.37 (-0.44, 1.56)	14 (-17, 60)	3.15 (-3.72, 13.36)	8.66 (-10.22, 36.70)	23.26 (-23.00, 88.30)	63.88 (-63.19, 243)
	90%	0.04 (-0.03, 0.11)	0.66 (-0.80, 2.81)	26 (-31, 108)	5.68 (-6.70, 24.05)	15.59 (-18.39, 66.07)	23.26 (-23.00, 88.30)	63.88 (-63.19, 243)

References for Data Inputs

[1] Blencowe H, Krasevec J, Onis M De, et al. Articles National , regional , and worldwide estimates of low birthweight in 2015 , with trends from 2000: a systematic analysis. Lancet Glob Heal. 2019;(18):1-12.
[2] Chawanpaiboon S, Vogel JP, Moller AB, et al. Global, regional, and national estimates of levels of preterm birth in 2014: a systematic review and modelling analysis. Lancet Glob Heal. 2019;7(1):e37-e46.
[3] United National Population Division World Population Prospects 2019.
[4] Fink G, Peet E, Danaei G, et al. Schooling and wage income losses due to early-childhood growth faltering in developing countries: National, regional, and global estimates. Am J Clin Nutr. 2016;104(1):104-112.
[5] Country specific annual wage data from World Indicators Database. Average yearly wage was estimated to be 2/3 of the gross domestic product in 2010 constant US dollars and 2011 International dollars, adjusted for purchasing power parity.
[6] NCD Risk Factor Collaboration. Trends in adult body-mass index in 200 countries from 1975 to 2014: a pooled analysis of 1698 population-based measurement studies with 19·2 million participants. Lancet. 2016;387(10026):1377-1396.
[7] Stevens GA, Finucane MM, De-Regil LM, et al. Global, regional, and national trends in haemoglobin concentration and prevalence of total and severe anaemia in children and pregnant and non-pregnant women for 1995-2011: A systematic analysis of population-representative data. Lancet Glob Heal. 2013;1(1):16-25.
[8] Coverage of iron-folic acid supplementation abstracted from the most recent Demographic Health Survey or imputed based on sub-regional average. Indicator used: % women in the past five years who took iron tablets or syrup for >90 days.

Iraq

Region: North Africa and Middle East**; Sub-region:** North Africa and Middle East

Low birthweight prevalence[1]: 5.6% (95% CI: 1.6, 9.6)

Preterm birth prevalence[2]: 10.4% (95% CI: 8.7, 11.9)

Number of births[3]: 5,520,000

Returns to education[4]: 0.7% (95% CI: 0.2, 1.1)

GDP per capita 2010 US$ (estimated annual wage)[5]: $5298 ($3532/year)

GDP per capita 2011 International $ (estimated annual wage)[5]: $14964 ($9976/year)

Prevalence of low BMI[6]: 2.0% (95% CI: 0.8, 4.1)

Prevalence of anemia[7]: 31.1% (95% CI: 15.0, 55.2)

Baseline coverage of IFA[8]: 24.3%

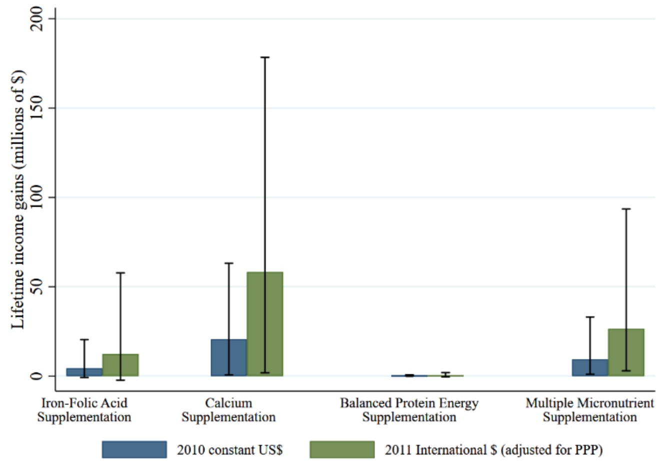

Figure: Benefits by birth cohort size for estimated gains in lifetime wages attributable to scaling up nutrition interventions to 90% target coverage

Table: Impact of maternal prenatal nutrition intervention on human capital and labour market outcomes, through improvements in low birthweight or preterm birth and schooling in Iraq.

Intervention	Target Coverage (%)	Absolute reduction in birth outcome (%)	Benefits by cohorts: School years gained (in 1000 s)	No. of additional students completing secondary school	Benefits by cohorts: Lifetime wages		Returns in lifetime earnings per child born to a targeted pregnant woman	
					in US $ millions	in International $ millions	in US $	in International $
Iron/Folic Acid Suppl.	90%	0.57 (-0.10, 1.53)	7.89 (-1.20, 26.18)	185 (-28, 613)	4.38 (-0.80, 20.44)	12.37 (-2.26, 57.74)	0.88 (-0.16, 4.12)	2.49 (-0.46, 11.62)
Calcium Suppl.	50%	1.24 (0.28, 2.13)	18.96 (0.51, 46.80)	444 (12, 1095)	11.47 (0.36, 35.10)	32.40 (1.01, 99.13)	4.16 (0.13, 12.72)	11.74 (0.37, 35.92)
	90%	2.23 (0.51, 3.83)	34.12 (0.91, 84.24)	798 (21, 1971)	20.65 (0.65, 63.17)	58.31 (1.83, 178.43)	4.16 (0.13, 12.72)	11.74 (0.37, 35.92)
Multiple Micronutrient Suppl.	50%	0.51 (0.14, 1.06)	7.36 (1.13, 20.30)	172 (26, 475)	4.47 (0.52, 15.17)	12.64 (1.46, 42.85)	1.62 (0.19, 5.50)	4.58 (0.53, 15.53)
	90%	1.08 (0.26, 2.33)	15.52 (2.20, 43.27)	363 (51, 1013)	9.40 (1.06, 33.11)	26.54 (2.98, 93.51)	1.89 (0.21, 6.66)	5.34 (0.60, 18.82)
Balanced Protein Suppl.	50%	0.01 (-0.01, 0.03)	0.10 (-0.12, 0.51)	2 (-3, 12)	0.06 (-0.08, 0.39)	0.16 (-0.21, 1.09)	1.20 (-1.44, 5.92)	3.40 (-4.05, 16.71)
	90%	0.01 (-0.01, 0.06)	0.18 (-0.22, 0.92)	4 (-5, 22)	0.10 (-0.14, 0.69)	0.29 (-0.38, 1.96)	1.20 (-1.44, 5.92)	3.40 (-4.05, 16.71)

References for Data Inputs

[1] Blencowe H, Krasevec J, Onis M De, et al. Articles National , regional , and worldwide estimates of low birthweight in 2015 , with trends from 2000: a systematic analysis. Lancet Glob Heal. 2019;(18):1-12.

[2] Chawanpaiboon S, Vogel JP, Moller AB, et al. Global, regional, and national estimates of levels of preterm birth in 2014: a systematic review and modelling analysis. Lancet Glob Heal. 2019;7(1):e37-e46.

[3] United National Population Division World Population Prospects 2019.

[4] Fink G, Peet E, Danaei G, et al. Schooling and wage income losses due to early-childhood growth faltering in developing countries: National, regional, and global estimates. Am J Clin Nutr. 2016;104(1):104-112.

[5] Country specific annual wage data from World Indicators Database. Average yearly wage was estimated to be 2/3 of the gross domestic product in 2010 constant US dollars and 2011 International dollars, adjusted for purchasing power parity.

[6] NCD Risk Factor Collaboration. Trends in adult body-mass index in 200 countries from 1975 to 2014: a pooled analysis of 1698 population-based measurement studies with 19·2 million participants. Lancet. 2016;387(10026):1377-1396.

[7] Stevens GA, Finucane MM, De-Regil LM, et al. Global, regional, and national trends in haemoglobin concentration and prevalence of total and severe anaemia in children and pregnant and non-pregnant women for 1995-2011: A systematic analysis of population-representative data. Lancet Glob Heal. 2013;1(1):16-25.

[8] Coverage of iron-folic acid supplementation abstracted from the most recent Demographic Health Survey or imputed based on sub-regional average. Indicator used: % women in the past five years who took iron tablets or syrup for >90 days.

Jamaica

Region: Latin America and Caribbean**; Sub-region:** Caribbean

Low birthweight prevalence[1]: 14.6% (95% CI: 11.4, 18.9)

Preterm birth prevalence[2]: 9.8% (95% CI: 8.6, 11.3)

Number of births[3]: 237,000

Returns to education[4]: 9.8% (95% CI: 8.1, 11.5)

GDP per capita 2010 US$ (estimated annual wage)[5]: $4714 ($3142/year)

GDP per capita 2011 International $ (estimated annual wage)[5]: $8047 ($5365/year)

Prevalence of low BMI[6]: 3.2% (95% CI: 1.4, 5.9)

Prevalence of anemia[7]: 30.4% (95% CI: 13.5, 54.1)

Baseline coverage of IFA[8]: 62.3%

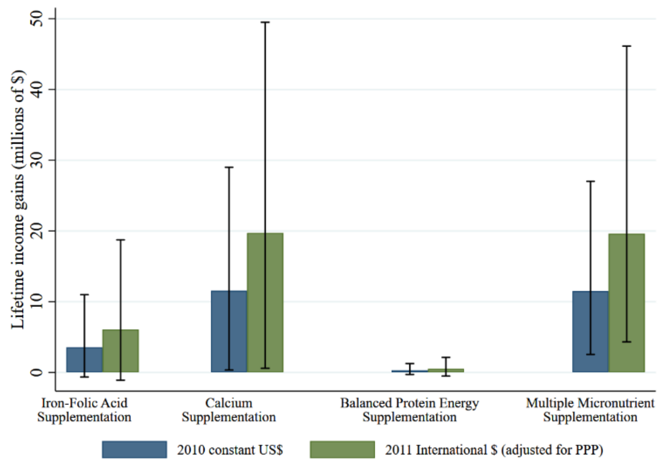

Figure: Benefits by birth cohort size for estimated gains in lifetime wages attributable to scaling up nutrition interventions to 90% target coverage

Table: Impact of maternal prenatal nutrition intervention on human capital and labour market outcomes, through improvements in low birthweight or preterm birth and schooling in Jamaica.

Intervention	Target Coverage (%)	Absolute reduction in birth outcome (%)	Benefits by cohorts: School years gained (in 1000 s)	No. of additional students completing secondary school	Benefits by cohorts: Lifetime wages		Returns in lifetime earnings per child born to a targeted pregnant woman	
					in US $ millions	in International $ millions	in US $	in International $
Iron/Folic Acid Suppl.	90%	0.72 (-0.12, 1.58)	0.43 (-0.08, 1.30)	23 (-4, 72)	3.55 (-0.64, 10.98)	6.07 (-1.09, 18.74)	16.66 (-3.00, 51.48)	28.43 (-5.12, 87.88)
Calcium Suppl.	50%	1.17 (0.25, 1.98)	0.78 (0.02, 1.90)	43 (1, 104)	6.42 (0.19, 16.11)	10.95 (0.32, 27.50)	54.15 (1.58, 135.94)	92.44 (2.70, 232.07)
	90%	2.11 (0.45, 3.57)	1.40 (0.04, 3.42)	77 (2, 187)	11.55 (0.34, 29.00)	19.72 (0.57, 49.50)	54.15 (1.58, 135.94)	92.44 (2.70, 232.07)
Multiple Micronutrient Suppl.	50%	0.87 (0.39, 1.41)	0.56 (0.11, 1.31)	31 (6, 72)	4.69 (0.82, 11.24)	8.01 (1.40, 19.19)	39.58 (6.94, 94.88)	67.56 (11.84, 161.98)
	90%	2.17 (1.15, 3.40)	1.38 (0.32, 3.17)	76 (17, 174)	11.49 (2.53, 27.02)	19.62 (4.32, 46.12)	53.88 (11.87, 126.66)	91.99 (20.26, 216.24)
Balanced Protein Suppl.	50%	0.04 (-0.03, 0.11)	0.02 (-0.02, 0.08)	1 (-1, 4)	0.17 (-0.17, 0.69)	0.29 (-0.29, 1.18)	50.00 (-48.88, 162.88)	85.36 (-83.44, 278.06)
	90%	0.06 (-0.06, 0.20)	0.04 (-0.04, 0.15)	2 (-2, 8)	0.30 (-0.30, 1.25)	0.52 (-0.52, 2.13)	50.00 (-48.88, 162.88)	85.36 (-83.44, 278.06)

References for Data Inputs

[1] Blencowe H, Krasevec J, Onis M De, et al. Articles National , regional , and worldwide estimates of low birthweight in 2015 , with trends from 2000: a systematic analysis. Lancet Glob Heal. 2019;(18):1-12.

[2] Chawanpaiboon S, Vogel JP, Moller AB, et al. Global, regional, and national estimates of levels of preterm birth in 2014: a systematic review and modelling analysis. Lancet Glob Heal. 2019;7(1):e37-e46.

[3] United National Population Division World Population Prospects 2019.

[4] Fink G, Peet E, Danaei G, et al. Schooling and wage income losses due to early-childhood growth faltering in developing countries: National, regional, and global estimates. Am J Clin Nutr. 2016;104(1):104-112.

[5] Country specific annual wage data from World Indicators Database. Average yearly wage was estimated to be 2/3 of the gross domestic product in 2010 constant US dollars and 2011 International dollars, adjusted for purchasing power parity.

[6] NCD Risk Factor Collaboration. Trends in adult body-mass index in 200 countries from 1975 to 2014: a pooled analysis of 1698 population-based measurement studies with 19·2 million participants. Lancet. 2016;387(10026):1377-1396.

[7] Stevens GA, Finucane MM, De-Regil LM, et al. Global, regional, and national trends in haemoglobin concentration and prevalence of total and severe anaemia in children and pregnant and non-pregnant women for 1995-2011: A systematic analysis of population-representative data. Lancet Glob Heal. 2013;1(1):16-25.

[8] Coverage of iron-folic acid supplementation abstracted from the most recent Demographic Health Survey or imputed based on sub-regional average. Indicator used: % women in the past five years who took iron tablets or syrup for >90 days.

Jordan

Region: North Africa and Middle East**; Sub-region:** North Africa and Middle East

Low birthweight prevalence[1]: 13.8% (95% CI: 11.0, 18.0)

Preterm birth prevalence[2]: 17.8% (95% CI: 12.6, 23.9)

Number of births[3]: 1,073,000

Returns to education[4]: 6.7% (95% CI: 4.1, 9.4)

GDP per capita 2010 US$ (estimated annual wage)[5]: $3275 ($2184/year)

GDP per capita 2011 International $ (estimated annual wage)[5]: $8408 ($5605/year)

Prevalence of low BMI[6]: 1.2% (95% CI: 0.6, 2.2)

Prevalence of anemia[7]: 27.6% (95% CI: 21.0, 35.8)

Baseline coverage of IFA[8]: 48.9%

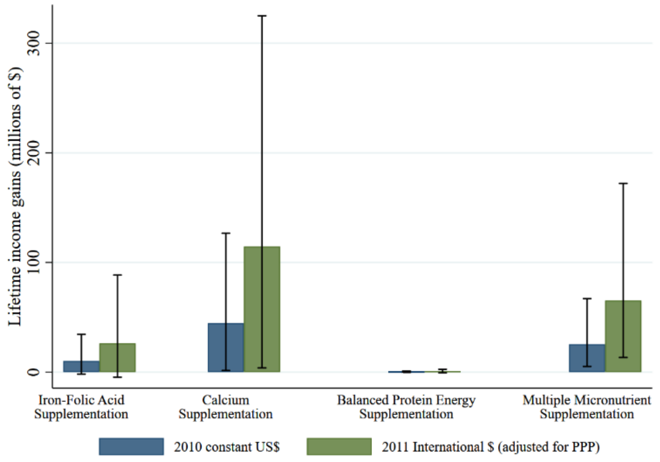

Figure: Benefits by birth cohort size for estimated gains in lifetime wages attributable to scaling up nutrition interventions to 90% target coverage

Table: Impact of maternal prenatal nutrition intervention on human capital and labour market outcomes, through improvements in low birthweight or preterm birth and schooling in Jordan.

Intervention	Target Coverage (%)	Absolute reduction in birth outcome (%)	Benefits by cohorts: School years gained (in 1000 s)	No. of additional students completing secondary school	Benefits by cohorts: Lifetime wages		Returns in lifetime earnings per child born to a targeted pregnant woman	
					in US $ millions	in International $ millions	in US $	in International $
Iron/Folic Acid Suppl.	90%	0.99 (-0.16, 2.15)	2.63 (-0.45, 7.95)	194 (-33, 585)	10.20 (-1.79, 34.49)	26.18 (-4.60, 88.53)	10.56 (-1.85, 35.71)	27.11 (-4.76, 91.67)
Calcium Suppl.	50%	2.11 (0.43, 3.73)	6.46 (0.21, 16.07)	475 (15, 1182)	24.79 (0.84, 70.36)	63.63 (2.16, 180.60)	46.20 (1.57, 131.14)	118.60 (4.03, 336.63)
	90%	3.79 (0.77, 6.72)	11.62 (0.37, 28.92)	855 (27, 2128)	44.62 (1.52, 126.64)	114.53 (3.89, 325.09)	46.20 (1.57, 131.14)	118.60 (4.03, 336.63)
Multiple Micronutrient Suppl.	50%	0.75 (0.39, 1.19)	2.24 (0.46, 5.13)	165 (34, 378)	8.57 (1.68, 22.09)	22.00 (4.32, 56.71)	15.98 (3.14, 41.18)	41.01 (8.06, 105.71)
	90%	2.27 (1.10, 3.64)	6.51 (1.40, 14.94)	479 (103, 1099)	25.42 (5.21, 67.07)	65.25 (13.37, 172.17)	26.32 (5.39, 69.45)	67.57 (13.85, 178.29)
Balanced Protein Suppl.	50%	0.01 (-0.01, 0.04)	0.04 (-0.03, 0.13)	3 (-2, 10)	0.14 (-0.13, 0.56)	0.35 (-0.35, 1.45)	21.74 (-22.30, 78.85)	55.82 (-57.25, 202.41)
	90%	0.02 (-0.02, 0.07)	0.06 (-0.06, 0.24)	5 (-4, 18)	0.24 (-0.24, 1.02)	0.63 (-0.62, 2.61)	21.74 (-22.30, 78.85)	55.82 (-57.25, 202.41)

References for Data Inputs

[1] Blencowe H, Krasevec J, Onis M De, et al. Articles National , regional , and worldwide estimates of low birthweight in 2015 , with trends from 2000: a systematic analysis. Lancet Glob Heal. 2019;(18):1-12.

[2] Chawanpaiboon S, Vogel JP, Moller AB, et al. Global, regional, and national estimates of levels of preterm birth in 2014: a systematic review and modelling analysis. Lancet Glob Heal. 2019;7(1):e37-e46.

[3] United National Population Division World Population Prospects 2019.

[4] Fink G, Peet E, Danaei G, et al. Schooling and wage income losses due to early-childhood growth faltering in developing countries: National, regional, and global estimates. Am J Clin Nutr. 2016;104(1):104-112.

[5] Country specific annual wage data from World Indicators Database. Average yearly wage was estimated to be 2/3 of the gross domestic product in 2010 constant US dollars and 2011 International dollars, adjusted for purchasing power parity.

[6] NCD Risk Factor Collaboration. Trends in adult body-mass index in 200 countries from 1975 to 2014: a pooled analysis of 1698 population-based measurement studies with 19·2 million participants. Lancet. 2016;387(10026):1377-1396.

[7] Stevens GA, Finucane MM, De-Regil LM, et al. Global, regional, and national trends in haemoglobin concentration and prevalence of total and severe anaemia in children and pregnant and non-pregnant women for 1995-2011: A systematic analysis of population-representative data. Lancet Glob Heal. 2013;1(1):16-25.

[8] Coverage of iron-folic acid supplementation abstracted from the most recent Demographic Health Survey or imputed based on sub-regional average. Indicator used: % women in the past five years who took iron tablets or syrup for >90 days.

Kazakhstan

Region: Central Europe, Eastern Europe, Central Asia**; Sub-region:** Central Asia

Low birthweight prevalence[1]: 5.4% (95% CI: 5.0, 5.9)

Preterm birth prevalence[2]: 5.2% (95% CI: 2.8, 8.8)

Number of births[3]: 1,943,000

Returns to education[4]: 8.0% (95% CI: 7.2, 8.8)

GDP per capita 2010 US$ (estimated annual wage)[5]: $10617 ($7078/year)

GDP per capita 2011 International $ (estimated annual wage)[5]: $23524 ($15683/year)

Prevalence of low BMI[6]: 3.5% (95% CI: 1.7, 6.2)

Prevalence of anemia[7]: 27.6% (95% CI: 15.7, 46.4)

Baseline coverage of IFA[8]: 3.0%

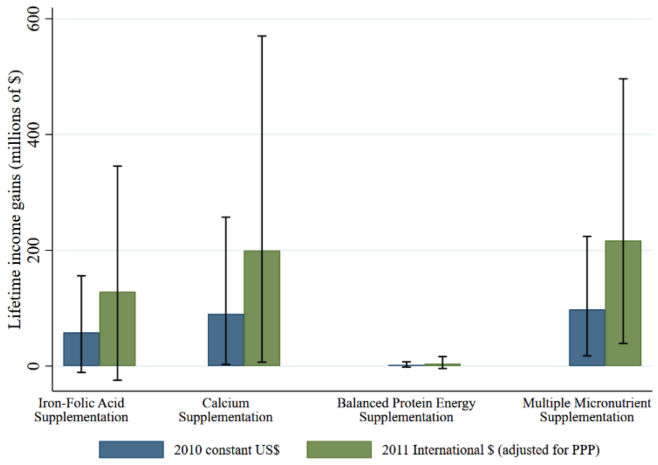

Figure: Benefits by birth cohort size for estimated gains in lifetime wages attributable to scaling up nutrition interventions to 90% target coverage

Table: Impact of maternal prenatal nutrition intervention on human capital and labour market outcomes, through improvements in low birthweight or preterm birth and schooling in Kazakhstan.

Intervention	Target Coverage (%)	Absolute reduction in birth outcome (%)	Benefits by cohorts: School years gained (in 1000 s)	No. of additional students completing secondary school	Benefits by cohorts: Lifetime wages		Returns in lifetime earnings per child born to a targeted pregnant woman	
					in US $ millions	in International $ millions	in US $	in International $
Iron/Folic Acid Suppl.	90%	0.76 (-0.15, 1.45)	3.79 (-0.73, 9.89)	22 (-4, 57)	58.11 (-10.99, 155.92)	128.75 (-24.36, 345.45)	33.23 (-6.29, 89.16)	73.62 (-13.93, 197.55)
Calcium Suppl.	50%	0.60 (0.11, 1.31)	3.24 (0.09, 9.38)	19 (1, 54)	50.06 (1.65, 142.97)	110.92 (3.65, 316.76)	51.53 (1.69, 147.16)	114.18 (3.75, 326.05)
	90%	1.09 (0.19, 2.36)	5.84 (0.17, 16.89)	34 (1, 98)	90.12 (2.96, 257.34)	199.66 (6.56, 570.17)	51.53 (1.69, 147.16)	114.18 (3.75, 326.05)
Multiple Micronutrient Suppl.	50%	0.67 (0.25, 1.04)	3.49 (0.62, 7.87)	20 (4, 46)	53.53 (9.60, 121.92)	118.60 (21.28, 270.12)	55.10 (9.89, 125.49)	122.08 (21.90, 278.04)
	90%	1.23 (0.45, 1.90)	6.39 (1.15, 14.46)	37 (7, 84)	97.79 (17.63, 223.98)	216.67 (39.07, 496.25)	55.92 (10.08, 128.08)	123.91 (22.34, 283.78)
Balanced Protein Suppl.	50%	0.01 (-0.01, 0.04)	0.07 (-0.07, 0.27)	0 (-0, 2)	1.07 (-1.08, 4.12)	2.37 (-2.39, 9.13)	34.19 (-30.85, 103.39)	75.74 (-68.34, 229.07)
	90%	0.03 (-0.02, 0.08)	0.12 (-0.13, 0.49)	1 (-1, 3)	1.93 (-1.94, 7.42)	4.27 (-4.30, 16.43)	34.19 (-30.85, 103.39)	75.74 (-68.34, 229.07)

References for Data Inputs

[1] Blencowe H, Krasevec J, Onis M De, et al. Articles National , regional , and worldwide estimates of low birthweight in 2015 , with trends from 2000: a systematic analysis. Lancet Glob Heal. 2019;(18):1-12.

[2] Chawanpaiboon S, Vogel JP, Moller AB, et al. Global, regional, and national estimates of levels of preterm birth in 2014: a systematic review and modelling analysis. Lancet Glob Heal. 2019;7(1):e37-e46.

[3] United National Population Division World Population Prospects 2019.

[4] Fink G, Peet E, Danaei G, et al. Schooling and wage income losses due to early-childhood growth faltering in developing countries: National, regional, and global estimates. Am J Clin Nutr. 2016;104(1):104-112.

[5] Country specific annual wage data from World Indicators Database. Average yearly wage was estimated to be 2/3 of the gross domestic product in 2010 constant US dollars and 2011 International dollars, adjusted for purchasing power parity.

[6] NCD Risk Factor Collaboration. Trends in adult body-mass index in 200 countries from 1975 to 2014: a pooled analysis of 1698 population-based measurement studies with 19·2 million participants. Lancet. 2016;387(10026):1377-1396.

[7] Stevens GA, Finucane MM, De-Regil LM, et al. Global, regional, and national trends in haemoglobin concentration and prevalence of total and severe anaemia in children and pregnant and non-pregnant women for 1995-2011: A systematic analysis of population-representative data. Lancet Glob Heal. 2013;1(1):16-25.

[8] Coverage of iron-folic acid supplementation abstracted from the most recent Demographic Health Survey or imputed based on sub-regional average. Indicator used: % women in the past five years who took iron tablets or syrup for >90 days.

Kenya

Region: Sub-Saharan Africa**; Sub-region:** Eastern Sub-Saharan Africa

Low birthweight prevalence[1]: 11.5% (95% CI: 8.9, 14.5)

Preterm birth prevalence[2]: 8.6% (95% CI: 6.3, 11.3)

Number of births[3]: 7,345,000

Returns to education[4]: 11.3% (95% CI: 9.7, 12.9)

GDP per capita 2010 US$ (estimated annual wage)[5]: $1093 ($729/year)

GDP per capita 2011 International $ (estimated annual wage)[5]: $2798 ($1865/year)

Prevalence of low BMI[6]: 9.6% (95% CI: 5.1, 15.4)

Prevalence of anemia[7]: 37.5% (95% CI: 12.7, 61.5)

Baseline coverage of IFA[8]: 7.5%

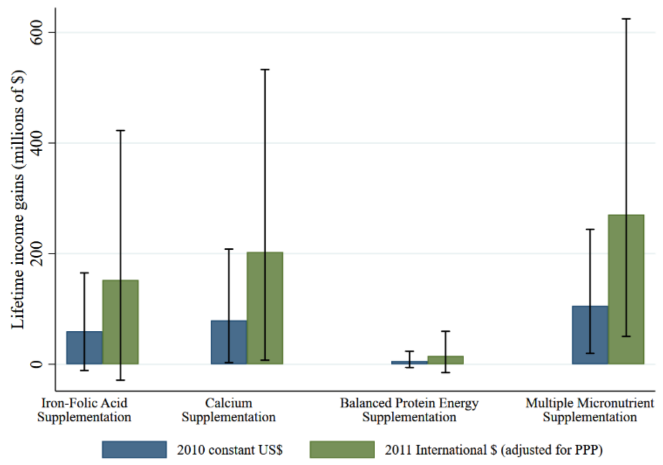

Figure: Benefits by birth cohort size for estimated gains in lifetime wages attributable to scaling up nutrition interventions to 90% target coverage

Table: Impact of maternal prenatal nutrition intervention on human capital and labour market outcomes, through improvements in low birthweight or preterm birth and schooling in Kenya.

Intervention	Target Coverage (%)	Absolute reduction in birth outcome (%)	Benefits by cohorts: School years gained (in 1000 s)	No. of additional students completing secondary school	Benefits by cohorts: Lifetime wages		Returns in lifetime earnings per child born to a targeted pregnant woman	
					in US $ millions	in International $ millions	in US $	in International $
Iron/Folic Acid Suppl.	90%	1.53 (-0.29, 3.07)	26.48 (-5.15, 72.56)	535 (-104, 1466)	59.55 (-11.23, 165.14)	152.42 (-28.74, 422.67)	9.01 (-1.70, 24.98)	23.06 (-4.35, 63.94)
Calcium Suppl.	50%	1.02 (0.22, 1.85)	19.97 (0.61, 52.78)	403 (12, 1066)	44.07 (1.62, 115.73)	112.81 (4.16, 296.22)	12.00 (0.44, 31.51)	30.72 (1.13, 80.66)
	90%	1.83 (0.40, 3.33)	35.95 (1.10, 95.01)	726 (22, 1919)	79.33 (2.92, 208.32)	203.06 (7.48, 533.19)	12.00 (0.44, 31.51)	30.72 (1.13, 80.66)
Multiple Micronutrient Suppl.	50%	1.41 (0.59, 2.25)	25.59 (5.09, 57.99)	517 (103, 1171)	56.12 (10.61, 130.22)	143.63 (27.17, 333.29)	15.28 (2.89, 35.46)	39.11 (7.40, 90.75)
	90%	2.66 (1.07, 4.25)	47.77 (9.56, 109.18)	965 (193, 2206)	105.79 (19.64, 244.13)	270.78 (50.27, 624.85)	16.00 (2.97, 36.93)	40.96 (7.60, 94.52)
Balanced Protein Suppl.	50%	0.09 (-0.08, 0.26)	1.48 (-1.44, 5.92)	30 (-29, 120)	3.26 (-3.27, 12.92)	8.33 (-8.36, 33.06)	9.93 (-10.07, 31.66)	25.41 (-25.79, 81.04)
	90%	0.16 (-0.14, 0.48)	2.66 (-2.60, 10.65)	54 (-53, 215)	5.86 (-5.88, 23.25)	15.00 (-15.06, 59.52)	9.93 (-10.07, 31.66)	25.41 (-25.79, 81.04)

References for Data Inputs
[1] Blencowe H, Krasevec J, Onis M De, et al. Articles National , regional , and worldwide estimates of low birthweight in 2015 , with trends from 2000: a systematic analysis. Lancet Glob Heal. 2019;(18):1-12.
[2] Chawanpaiboon S, Vogel JP, Moller AB, et al. Global, regional, and national estimates of levels of preterm birth in 2014: a systematic review and modelling analysis. Lancet Glob Heal. 2019;7(1):e37-e46.
[3] United National Population Division World Population Prospects 2019.
[4] Fink G, Peet E, Danaei G, et al. Schooling and wage income losses due to early-childhood growth faltering in developing countries: National, regional, and global estimates. Am J Clin Nutr. 2016;104(1):104-112.
[5] Country specific annual wage data from World Indicators Database. Average yearly wage was estimated to be 2/3 of the gross domestic product in 2010 constant US dollars and 2011 International dollars, adjusted for purchasing power parity.
[6] NCD Risk Factor Collaboration. Trends in adult body-mass index in 200 countries from 1975 to 2014: a pooled analysis of 1698 population-based measurement studies with 19·2 million participants. Lancet. 2016;387(10026):1377-1396.
[7] Stevens GA, Finucane MM, De-Regil LM, et al. Global, regional, and national trends in haemoglobin concentration and prevalence of total and severe anaemia in children and pregnant and non-pregnant women for 1995-2011: A systematic analysis of population-representative data. Lancet Glob Heal. 2013;1(1):16-25.
[8] Coverage of iron-folic acid supplementation abstracted from the most recent Demographic Health Survey or imputed based on sub-regional average. Indicator used: % women in the past five years who took iron tablets or syrup for >90 days.

Kiribati

Region: Southeast Asia, East Asia, and Oceania**; Sub-region:** Oceania

Low birthweight prevalence[1]: 8.2% (95% CI: 5.5, 10.9)

Preterm birth prevalence[2]: 10.0% (95% CI: 7.9, 12.7)

Number of births[3]: 16,000

Returns to education[4]: 6.1% (95% CI: 2.7, 9.6)

GDP per capita 2010 US$ (estimated annual wage)[5]: $1711 ($1140/year)

GDP per capita 2011 International $ (estimated annual wage)[5]: $1976 ($1317/year)

Prevalence of low BMI[6]: 1.1% (95% CI: 0.3, 2.6)

Prevalence of anemia[7]: 29.2% (95% CI: 10.9, 56.2)

Baseline coverage of IFA[8]: 44.2%

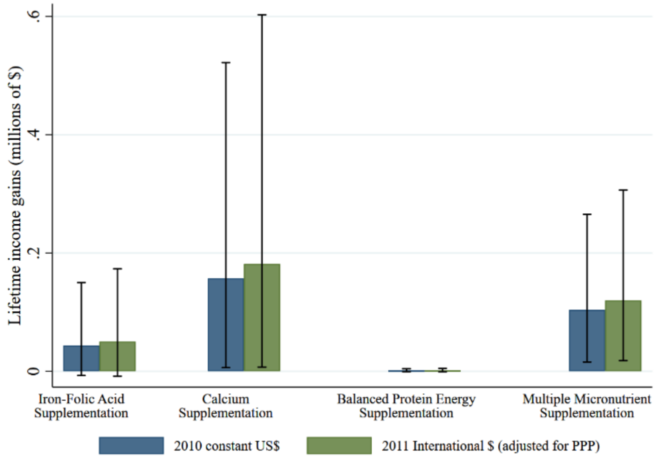

Figure: Benefits by birth cohort size for estimated gains in lifetime wages attributable to scaling up nutrition interventions to 90% target coverage

Table: Impact of maternal prenatal nutrition intervention on human capital and labour market outcomes, through improvements in low birthweight or preterm birth and schooling in Kiribati.

Intervention	Target Coverage (%)	Absolute reduction in birth outcome (%)	Benefits by cohorts: School years gained (in 1000 s)	No. of additional students completing secondary school	Benefits by cohorts: Lifetime wages		Returns in lifetime earnings per child born to a targeted pregnant woman	
					in US $ millions	in International $ millions	in US $	in International $
Iron/Folic Acid Suppl.	90%	0.63 (-0.11, 1.39)	0.02 (-0.00, 0.07)	1 (-0, 2)	0.04 (-0.01, 0.15)	0.05 (-0.01, 0.17)	3.02 (-0.50, 10.42)	3.49 (-0.57, 12.03)
Calcium Suppl.	50%	1.20 (0.25, 2.07)	0.05 (0.00, 0.13)	1 (0, 3)	0.09 (0.00, 0.29)	0.10 (0.00, 0.33)	10.90 (0.42, 36.25)	12.59 (0.49, 41.86)
	90%	2.15 (0.45, 3.72)	0.09 (0.00, 0.24)	2 (0, 5)	0.16 (0.01, 0.52)	0.18 (0.01, 0.60)	10.90 (0.42, 36.25)	12.59 (0.49, 41.86)
Multiple Micronutrient Suppl.	50%	0.51 (0.25, 0.80)	0.02 (0.00, 0.05)	0 (0, 1)	0.04 (0.01, 0.10)	0.04 (0.01, 0.12)	4.81 (0.78, 12.46)	5.55 (0.90, 14.39)
	90%	1.41 (0.59, 2.32)	0.06 (0.01, 0.13)	1 (0, 3)	0.10 (0.02, 0.27)	0.12 (0.02, 0.31)	7.20 (1.09, 18.43)	8.32 (1.26, 21.28)
Balanced Protein Suppl.	50%	0.01 (-0.01, 0.03)	0.00 (-0.00, 0.00)	0 (-0, 0)	0.00 (-0.00, 0.00)	0.00 (-0.00, 0.00)	5.25 (-5.48, 22.44)	6.06 (-6.33, 25.92)
	90%	0.01 (-0.01, 0.05)	0.00 (-0.00, 0.00)	0 (-0, 0)	0.00 (-0.00, 0.00)	0.00 (-0.00, 0.00)	5.25 (-5.48, 22.44)	6.06 (-6.33, 25.92)

References for Data Inputs
[1] Blencowe H, Krasevec J, Onis M De, et al. Articles National , regional , and worldwide estimates of low birthweight in 2015 , with trends from 2000: a systematic analysis. Lancet Glob Heal. 2019;(18):1-12.
[2] Chawanpaiboon S, Vogel JP, Moller AB, et al. Global, regional, and national estimates of levels of preterm birth in 2014: a systematic review and modelling analysis. Lancet Glob Heal. 2019;7(1):e37-e46.
[3] United National Population Division World Population Prospects 2019.
[4] Fink G, Peet E, Danaei G, et al. Schooling and wage income losses due to early-childhood growth faltering in developing countries: National, regional, and global estimates. Am J Clin Nutr. 2016;104(1):104-112.
[5] Country specific annual wage data from World Indicators Database. Average yearly wage was estimated to be 2/3 of the gross domestic product in 2010 constant US dollars and 2011 International dollars, adjusted for purchasing power parity.
[6] NCD Risk Factor Collaboration. Trends in adult body-mass index in 200 countries from 1975 to 2014: a pooled analysis of 1698 population-based measurement studies with 19·2 million participants. Lancet. 2016;387(10026):1377-1396.
[7] Stevens GA, Finucane MM, De-Regil LM, et al. Global, regional, and national trends in haemoglobin concentration and prevalence of total and severe anaemia in children and pregnant and non-pregnant women for 1995-2011: A systematic analysis of population-representative data. Lancet Glob Heal. 2013;1(1):16-25.
[8] Coverage of iron-folic acid supplementation abstracted from the most recent Demographic Health Survey or imputed based on sub-regional average. Indicator used: % women in the past five years who took iron tablets or syrup for >90 days.

Kyrgyzstan

Region: Central Europe, Eastern Europe, Central Asia**; Sub-region:** Central Asia

Low birthweight prevalence[1]: 5.5% (95% CI: 5.2, 5.8)

Preterm birth prevalence[2]: 10.4% (95% CI: 8.7, 11.9)

Number of births[3]: 774,000

Returns to education[4]: 5.2% (95% CI: 2.7, 7.7)

GDP per capita 2010 US$ (estimated annual wage)[5]: $1020 ($680/year)

GDP per capita 2011 International $ (estimated annual wage)[5]: $3234 ($2156/year)

Prevalence of low BMI[6]: 3.8% (95% CI: 1.6, 7.2)

Prevalence of anemia[7]: 30.3% (95% CI: 18.0, 48.7)

Baseline coverage of IFA[8]: 2.3%

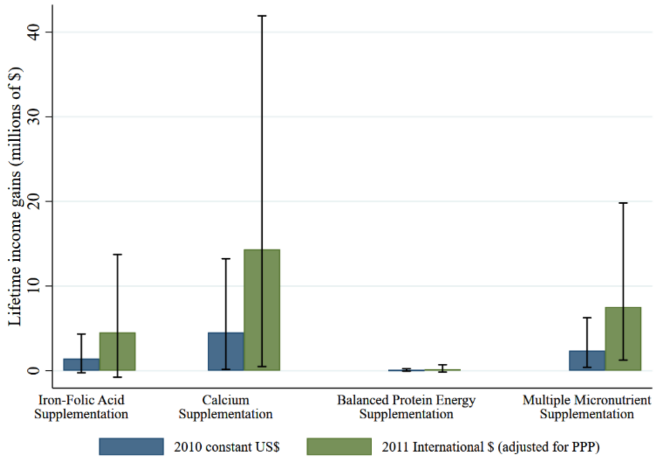

Figure: Benefits by birth cohort size for estimated gains in lifetime wages attributable to scaling up nutrition interventions to 90% target coverage

Table: Impact of maternal prenatal nutrition intervention on human capital and labour market outcomes, through improvements in low birthweight or preterm birth and schooling in Kyrgyzstan.

Intervention	Target Coverage (%)	Absolute reduction in birth outcome (%)	Benefits by cohorts: School years gained (in 1000 s)	No. of additional students completing secondary school	Benefits by cohorts: Lifetime wages		Returns in lifetime earnings per child born to a targeted pregnant woman	
					in US $ millions	in International $ millions	in US $	in International $
Iron/Folic Acid Suppl.	90%	0.78 (-0.15, 1.51)	1.54 (-0.29, 3.94)	21 (-4, 54)	1.43 (-0.24, 4.33)	4.52 (-0.76, 13.73)	2.05 (-0.35, 6.22)	6.49 (-1.10, 19.71)
Calcium Suppl.	50%	1.25 (0.28, 2.10)	2.77 (0.09, 6.64)	38 (1, 90)	2.51 (0.09, 7.35)	7.96 (0.27, 23.30)	6.49 (0.22, 18.99)	20.57 (0.71, 60.21)
	90%	2.24 (0.51, 3.78)	4.98 (0.16, 11.95)	68 (2, 162)	4.52 (0.16, 13.23)	14.33 (0.49, 41.95)	6.49 (0.22, 18.99)	20.57 (0.71, 60.21)
Multiple Micronutrient Suppl.	50%	0.70 (0.25, 1.07)	1.43 (0.25, 3.12)	19 (3, 42)	1.31 (0.22, 3.42)	4.14 (0.70, 10.85)	3.38 (0.57, 8.84)	10.71 (1.81, 28.03)
	90%	1.28 (0.45, 1.95)	2.61 (0.45, 5.71)	35 (6, 78)	2.38 (0.40, 6.25)	7.53 (1.26, 19.82)	3.41 (0.57, 8.97)	10.81 (1.81, 28.45)
Balanced Protein Suppl.	50%	0.02 (-0.01, 0.05)	0.03 (-0.03, 0.13)	0 (-0, 2)	0.03 (-0.03, 0.12)	0.09 (-0.09, 0.39)	2.03 (-2.06, 7.18)	6.43 (-6.52, 22.77)
	90%	0.03 (-0.02, 0.09)	0.05 (-0.06, 0.23)	1 (-1, 3)	0.05 (-0.05, 0.22)	0.16 (-0.16, 0.71)	2.03 (-2.06, 7.18)	6.43 (-6.52, 22.77)

References for Data Inputs

[1] Blencowe H, Krasevec J, Onis M De, et al. Articles National , regional , and worldwide estimates of low birthweight in 2015 , with trends from 2000: a systematic analysis. Lancet Glob Heal. 2019;(18):1-12.
[2] Chawanpaiboon S, Vogel JP, Moller AB, et al. Global, regional, and national estimates of levels of preterm birth in 2014: a systematic review and modelling analysis. Lancet Glob Heal. 2019;7(1):e37-e46.
[3] United National Population Division World Population Prospects 2019.
[4] Fink G, Peet E, Danaei G, et al. Schooling and wage income losses due to early-childhood growth faltering in developing countries: National, regional, and global estimates. Am J Clin Nutr. 2016;104(1):104-112.
[5] Country specific annual wage data from World Indicators Database. Average yearly wage was estimated to be 2/3 of the gross domestic product in 2010 constant US dollars and 2011 International dollars, adjusted for purchasing power parity.
[6] NCD Risk Factor Collaboration. Trends in adult body-mass index in 200 countries from 1975 to 2014: a pooled analysis of 1698 population-based measurement studies with 19·2 million participants. Lancet. 2016;387(10026):1377-1396.
[7] Stevens GA, Finucane MM, De-Regil LM, et al. Global, regional, and national trends in haemoglobin concentration and prevalence of total and severe anaemia in children and pregnant and non-pregnant women for 1995-2011: A systematic analysis of population-representative data. Lancet Glob Heal. 2013;1(1):16-25.
[8] Coverage of iron-folic acid supplementation abstracted from the most recent Demographic Health Survey or imputed based on sub-regional average. Indicator used: % women in the past five years who took iron tablets or syrup for >90 days.

Laos People's Democratic Republic

Region: Southeast Asia, East Asia, and Oceania**; Sub-region:** Southeast Asia

Low birthweight prevalence[1]: 17.3% (95% CI: 7.3, 11.7)

Preterm birth prevalence[2]: 10.4% (95% CI: 8.7, 11.9)

Number of births[3]: 835,000

Returns to education[4]: 6.5% (95% CI: 2.1, 10.9)

GDP per capita 2010 US$ (estimated annual wage)[5]: $1539 ($1026/year)

GDP per capita 2011 International $ (estimated annual wage)[5]: $5689 ($3793/year)

Prevalence of low BMI[6]: 11.4% (95% CI: 5.9, 18.3)

Prevalence of anemia[7]: 37.1% (95% CI: 22.5, 55.6)

Baseline coverage of IFA[8]: 46.2%

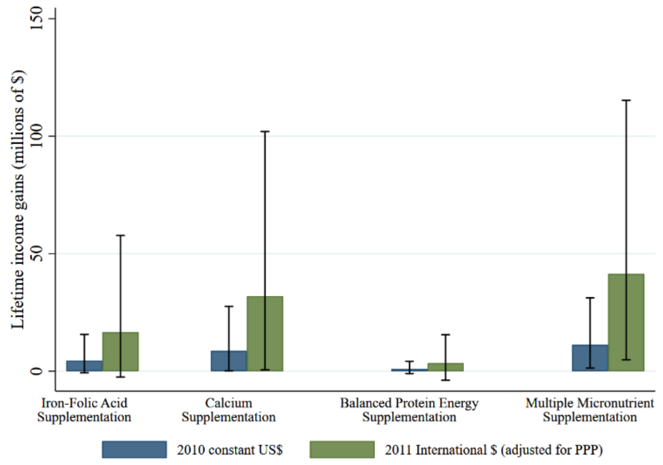

Figure: Benefits by birth cohort size for estimated gains in lifetime wages attributable to scaling up nutrition interventions to 90% target coverage

Table: Impact of maternal prenatal nutrition intervention on human capital and labour market outcomes, through improvements in low birthweight or preterm birth and schooling in Laos People's Democratic Republic.

Intervention	Target Coverage (%)	Absolute reduction in birth outcome (%)	Benefits by cohorts: School years gained (in 1000 s)	No. of additional students completing secondary school	Benefits by cohorts: Lifetime wages		Returns in lifetime earnings per child born to a targeted pregnant woman	
					in US $ millions	in International $ millions	in US $	in International $
Iron/Folic Acid Suppl.	90%	1.29 (-0.23, 2.85)	2.62 (-0.39, 7.51)	172 (-26, 494)	4.49 (-0.67, 15.63)	16.59 (-2.48, 57.80)	5.97 (-0.89, 20.80)	22.08 (-3.30, 76.91)
Calcium Suppl.	50%	1.24 (0.28, 2.10)	2.86 (0.09, 6.79)	188 (6, 447)	4.80 (0.08, 15.33)	17.75 (0.29, 56.67)	11.50 (0.19, 36.71)	42.52 (0.70, 135.73)
	90%	2.23 (0.51, 3.78)	5.15 (0.17, 12.23)	339 (11, 805)	8.64 (0.14, 27.59)	31.95 (0.53, 102.00)	11.50 (0.19, 36.71)	42.52 (0.70, 135.73)
Multiple Micronutrient Suppl.	50%	1.09 (0.66, 1.57)	2.38 (0.50, 5.34)	156 (33, 351)	4.01 (0.53, 11.09)	14.83 (1.96, 41.00)	9.61 (1.27, 26.56)	35.53 (4.70, 98.20)
	90%	3.05 (1.51, 4.65)	6.53 (1.41, 14.50)	430 (93, 954)	11.20 (1.31, 31.18)	41.41 (4.86, 115.29)	14.91 (1.75, 41.49)	55.11 (6.47, 153.41)
Balanced Protein Suppl.	50%	0.16 (-0.14, 0.42)	0.30 (-0.31, 1.07)	20 (-21, 70)	0.51 (-0.57, 2.33)	1.89 (-2.13, 8.60)	11.51 (-11.21, 45.27)	42.56 (-41.46, 167.37)
	90%	0.28 (-0.26, 0.76)	0.54 (-0.56, 1.92)	35 (-37, 126)	0.92 (-1.03, 4.19)	3.40 (-3.83, 15.48)	11.51 (-11.21, 45.27)	42.56 (-41.46, 167.37)

References for Data Inputs
[1] Blencowe H, Krasevec J, Onis M De, et al. Articles National , regional , and worldwide estimates of low birthweight in 2015 , with trends from 2000: a systematic analysis. Lancet Glob Heal. 2019;(18):1-12.
[2] Chawanpaiboon S, Vogel JP, Moller AB, et al. Global, regional, and national estimates of levels of preterm birth in 2014: a systematic review and modelling analysis. Lancet Glob Heal. 2019;7(1):e37-e46.
[3] United National Population Division World Population Prospects 2019.
[4] Fink G, Peet E, Danaei G, et al. Schooling and wage income losses due to early-childhood growth faltering in developing countries: National, regional, and global estimates. Am J Clin Nutr. 2016;104(1):104-112.
[5] Country specific annual wage data from World Indicators Database. Average yearly wage was estimated to be 2/3 of the gross domestic product in 2010 constant US dollars and 2011 International dollars, adjusted for purchasing power parity.
[6] NCD Risk Factor Collaboration. Trends in adult body-mass index in 200 countries from 1975 to 2014: a pooled analysis of 1698 population-based measurement studies with 19·2 million participants. Lancet. 2016;387(10026):1377-1396.
[7] Stevens GA, Finucane MM, De-Regil LM, et al. Global, regional, and national trends in haemoglobin concentration and prevalence of total and severe anaemia in children and pregnant and non-pregnant women for 1995-2011: A systematic analysis of population-representative data. Lancet Glob Heal. 2013;1(1):16-25.
[8] Coverage of iron-folic acid supplementation abstracted from the most recent Demographic Health Survey or imputed based on sub-regional average. Indicator used: % women in the past five years who took iron tablets or syrup for >90 days.

Lebanon

Region: North Africa and Middle East**; Sub-region:** North Africa and Middle East

Low birthweight prevalence[1]: 9.2% (95% CI: 7.3, 11.7)

Preterm birth prevalence[2]: 9.0% (95% CI: 5.3, 14.3)

Number of births[3]: 587,000

Returns to education[4]: 6.7% (95% CI: 4.1, 9.4)

GDP per capita 2010 US$ (estimated annual wage)[5]: $6401 ($4267/year)

GDP per capita 2011 International $ (estimated annual wage)[5]: $11887 ($7925/year)

Prevalence of low BMI[6]: 2.2% (95% CI: 0.9, 4.4)

Prevalence of anemia[7]: 31.1% (95% CI: 22.2, 41.4)

Baseline coverage of IFA[8]: 24.3%

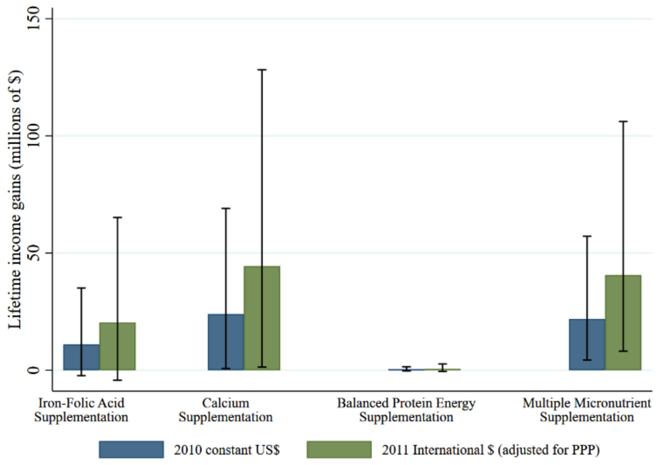

Figure: Benefits by birth cohort size for estimated gains in lifetime wages attributable to scaling up nutrition interventions to 90% target coverage

Table: Impact of maternal prenatal nutrition intervention on human capital and labour market outcomes, through improvements in low birthweight or preterm birth and schooling in Lebanon.

Intervention	Target Coverage (%)	Absolute reduction in birth outcome (%)	Benefits by cohorts: School years gained (in 1000 s)	No. of additional students completing secondary school	Benefits by cohorts: Lifetime wages		Returns in lifetime earnings per child born to a targeted pregnant woman	
					in US $ millions	in International $ millions	in US $	in International $
Iron/Folic Acid Suppl.	90%	0.99 (-0.18, 2.15)	1.50 (-0.26, 4.29)	51 (-9, 146)	10.98 (-2.29, 35.09)	20.40 (-4.25, 65.17)	20.79 (-4.33, 66.43)	38.61 (-8.05, 123.37)
Calcium Suppl.	50%	1.03 (0.15, 2.13)	1.75 (0.06, 5.10)	60 (2, 173)	13.28 (0.39, 38.35)	24.66 (0.73, 71.23)	45.23 (1.33, 130.68)	84.01 (2.47, 242.70)
	90%	1.86 (0.27, 3.83)	3.15 (0.10, 9.17)	107 (3, 312)	23.90 (0.70, 69.04)	44.38 (1.31, 128.22)	45.23 (1.33, 130.68)	84.01 (2.47, 242.70)
Multiple Micronutrient Suppl.	50%	0.86 (0.41, 1.32)	1.36 (0.30, 3.04)	46 (10, 103)	10.32 (2.10, 26.13)	19.17 (3.90, 48.53)	35.17 (7.16, 89.03)	65.31 (13.29, 165.34)
	90%	1.84 (0.78, 3.00)	2.89 (0.61, 6.69)	98 (21, 228)	21.86 (4.38, 57.15)	40.60 (8.13, 106.13)	41.38 (8.29, 108.17)	76.85 (15.39, 200.90)
Balanced Protein Suppl.	50%	0.02 (-0.01, 0.06)	0.02 (-0.02, 0.10)	1 (-1, 3)	0.17 (-0.17, 0.79)	0.31 (-0.32, 1.47)	28.40 (-29.47, 101.58)	52.74 (-54.74, 188.66)
	90%	0.03 (-0.03, 0.10)	0.04 (-0.04, 0.18)	1 (-1, 6)	0.30 (-0.31, 1.43)	0.55 (-0.57, 2.65)	28.40 (-29.47, 101.58)	52.74 (-54.74, 188.66)

References for Data Inputs

[1] Blencowe H, Krasevec J, Onis M De, et al. Articles National , regional , and worldwide estimates of low birthweight in 2015 , with trends from 2000: a systematic analysis. Lancet Glob Heal. 2019;(18):1-12.
[2] Chawanpaiboon S, Vogel JP, Moller AB, et al. Global, regional, and national estimates of levels of preterm birth in 2014: a systematic review and modelling analysis. Lancet Glob Heal. 2019;7(1):e37-e46.
[3] United National Population Division World Population Prospects 2019.
[4] Fink G, Peet E, Danaei G, et al. Schooling and wage income losses due to early-childhood growth faltering in developing countries: National, regional, and global estimates. Am J Clin Nutr. 2016;104(1):104-112.
[5] Country specific annual wage data from World Indicators Database. Average yearly wage was estimated to be 2/3 of the gross domestic product in 2010 constant US dollars and 2011 International dollars, adjusted for purchasing power parity.
[6] NCD Risk Factor Collaboration. Trends in adult body-mass index in 200 countries from 1975 to 2014: a pooled analysis of 1698 population-based measurement studies with 19·2 million participants. Lancet. 2016;387(10026):1377-1396.
[7] Stevens GA, Finucane MM, De-Regil LM, et al. Global, regional, and national trends in haemoglobin concentration and prevalence of total and severe anaemia in children and pregnant and non-pregnant women for 1995-2011: A systematic analysis of population-representative data. Lancet Glob Heal. 2013;1(1):16-25.
[8] Coverage of iron-folic acid supplementation abstracted from the most recent Demographic Health Survey or imputed based on sub-regional average. Indicator used: % women in the past five years who took iron tablets or syrup for >90 days.

Lesotho

Region: Sub-Saharan Africa**; Sub-region:** Southern Sub-Saharan Africa

Low birthweight prevalence[1]: 14.6% (95% CI: 11.5, 19.3)

Preterm birth prevalence[2]: 12.0% (95% CI: 8.6, 16.7)

Number of births[3]: 284,000

Returns to education[4]: 16.1% (95% CI: 15.3, 16.8)

GDP per capita 2010 US$ (estimated annual wage)[5]: $1403 ($935/year)

GDP per capita 2011 International $ (estimated annual wage)[5]: $2867 ($1911/year)

Prevalence of low BMI[6]: 4.4% (95% CI: 1.9, 8.2)

Prevalence of anemia[7]: 53.7% (95% CI: 36.2, 68.1)

Baseline coverage of IFA[8]: 51.4%

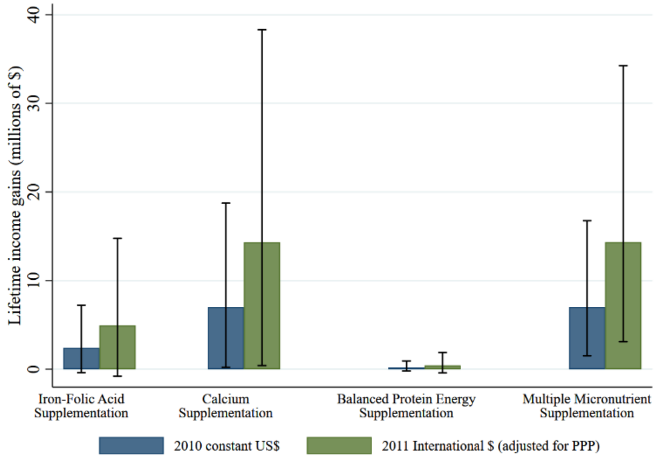

Figure: Benefits by birth cohort size for estimated gains in lifetime wages attributable to scaling up nutrition interventions to 90% target coverage

Table: Impact of maternal prenatal nutrition intervention on human capital and labour market outcomes, through improvements in low birthweight or preterm birth and schooling in Lesotho.

Intervention	Target Coverage (%)	Absolute reduction in birth outcome (%)	Benefits by cohorts: School years gained (in 1000 s)	No. of additional students completing secondary school	Benefits by cohorts: Lifetime wages		Returns in lifetime earnings per child born to a targeted pregnant woman	
					in US $ millions	in International $ millions	in US $	in International $
Iron/Folic Acid Suppl.	90%	0.96 (-0.16, 2.22)	0.60 (-0.10, 1.80)	12 (-2, 37)	2.41 (-0.38, 7.22)	4.93 (-0.78, 14.76)	9.45 (-1.50, 28.26)	19.31 (-3.07, 57.75)
Calcium Suppl.	50%	1.41 (0.27, 2.63)	0.96 (0.03, 2.58)	20 (1, 53)	3.89 (0.11, 10.41)	7.95 (0.23, 21.28)	27.39 (0.80, 73.32)	55.98 (1.63, 149.87)
	90%	2.53 (0.49, 4.73)	1.72 (0.05, 4.65)	35 (1, 96)	7.00 (0.20, 18.74)	14.31 (0.42, 38.31)	27.39 (0.80, 73.32)	55.98 (1.63, 149.87)
Multiple Micronutrient Suppl.	50%	1.02 (0.55, 1.58)	0.70 (0.13, 1.65)	14 (3, 34)	2.79 (0.51, 6.62)	5.69 (1.03, 13.53)	19.62 (3.57, 46.61)	40.10 (7.29, 95.28)
	90%	2.63 (1.40, 4.14)	1.74 (0.38, 4.16)	36 (8, 86)	7.01 (1.52, 16.76)	14.32 (3.10, 34.26)	27.41 (5.94, 65.57)	56.03 (12.14, 134.03)
Balanced Protein Suppl.	50%	0.05 (-0.04, 0.16)	0.03 (-0.03, 0.13)	1 (-1, 3)	0.12 (-0.11, 0.51)	0.24 (-0.23, 1.05)	20.47 (-18.58, 65.98)	41.85 (-37.98, 134.86)
	90%	0.09 (-0.08, 0.29)	0.05 (-0.05, 0.23)	1 (-1, 5)	0.21 (-0.20, 0.92)	0.42 (-0.41, 1.88)	20.47 (-18.58, 65.98)	41.85 (-37.98, 134.86)

References for Data Inputs

[1] Blencowe H, Krasevec J, Onis M De, et al. Articles National , regional , and worldwide estimates of low birthweight in 2015 , with trends from 2000: a systematic analysis. Lancet Glob Heal. 2019;(18):1-12.
[2] Chawanpaiboon S, Vogel JP, Moller AB, et al. Global, regional, and national estimates of levels of preterm birth in 2014: a systematic review and modelling analysis. Lancet Glob Heal. 2019;7(1):e37-e46.
[3] United National Population Division World Population Prospects 2019.
[4] Fink G, Peet E, Danaei G, et al. Schooling and wage income losses due to early-childhood growth faltering in developing countries: National, regional, and global estimates. Am J Clin Nutr. 2016;104(1):104-112.
[5] Country specific annual wage data from World Indicators Database. Average yearly wage was estimated to be 2/3 of the gross domestic product in 2010 constant US dollars and 2011 International dollars, adjusted for purchasing power parity.
[6] NCD Risk Factor Collaboration. Trends in adult body-mass index in 200 countries from 1975 to 2014: a pooled analysis of 1698 population-based measurement studies with 19·2 million participants. Lancet. 2016;387(10026):1377-1396.
[7] Stevens GA, Finucane MM, De-Regil LM, et al. Global, regional, and national trends in haemoglobin concentration and prevalence of total and severe anaemia in children and pregnant and non-pregnant women for 1995-2011: A systematic analysis of population-representative data. Lancet Glob Heal. 2013;1(1):16-25.
[8] Coverage of iron-folic acid supplementation abstracted from the most recent Demographic Health Survey or imputed based on sub-regional average. Indicator used: % women in the past five years who took iron tablets or syrup for >90 days.

Liberia

Region: Sub-Saharan Africa**; Sub-region:** Western Sub-Saharan Africa

Low birthweight prevalence[1]: 13.7% (95% CI: 8.3, 19.1)

Preterm birth prevalence[2]: 12.0% (95% CI: 8.6, 16.7)

Number of births[3]: 791,000

Returns to education[4]: 6.3% (95% CI: 4.5, 8.1)

GDP per capita 2010 US$ (estimated annual wage)[5]: $571 ($381/year)

GDP per capita 2011 International $ (estimated annual wage)[5]: $1226 ($817/year)

Prevalence of low BMI[6]: 7.7% (95% CI: 3.5, 13.5)

Prevalence of anemia[7]: 28.7% (95% CI: 13.2, 52.3)

Baseline coverage of IFA[8]: 21.2%

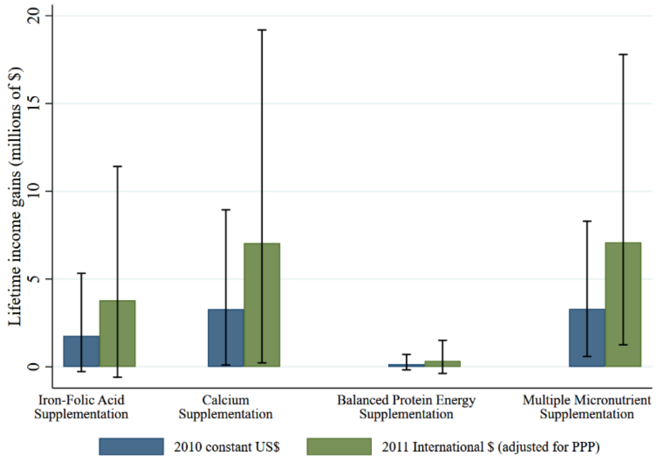

Figure: Benefits by birth cohort size for estimated gains in lifetime wages attributable to scaling up nutrition interventions to 90% target coverage

Table: Impact of maternal prenatal nutrition intervention on human capital and labour market outcomes, through improvements in low birthweight or preterm birth and schooling in Liberia.

Intervention	Target Coverage (%)	Absolute reduction in birth outcome (%)	Benefits by cohorts: School years gained (in 1000 s)	No. of additional students completing secondary school	Benefits by cohorts: Lifetime wages		Returns in lifetime earnings per child born to a targeted pregnant woman	
					in US $ millions	in International $ millions	in US $	in International $
Iron/Folic Acid Suppl.	90%	1.52 (-0.31, 3.46)	2.74 (-0.43, 8.16)	72 (-11, 215)	1.76 (-0.27, 5.32)	3.78 (-0.59, 11.42)	2.48 (-0.39, 7.48)	5.32 (-0.83, 16.04)
Calcium Suppl.	50%	1.41 (0.29, 2.58)	2.86 (0.07, 7.57)	76 (2, 200)	1.82 (0.06, 4.97)	3.91 (0.13, 10.66)	4.61 (0.15, 12.57)	9.90 (0.32, 26.96)
	90%	2.54 (0.53, 4.65)	5.16 (0.13, 13.62)	136 (3, 360)	3.28 (0.11, 8.95)	7.04 (0.23, 19.19)	4.61 (0.15, 12.57)	9.90 (0.32, 26.96)
Multiple Micronutrient Suppl.	50%	1.31 (0.53, 2.28)	2.55 (0.50, 6.07)	67 (13, 160)	1.60 (0.33, 3.95)	3.44 (0.71, 8.47)	4.06 (0.84, 9.98)	8.70 (1.80, 21.42)
	90%	2.74 (1.00, 4.94)	5.24 (0.96, 12.63)	138 (25, 333)	3.31 (0.59, 8.29)	7.10 (1.26, 17.79)	4.65 (0.82, 11.65)	9.97 (1.77, 24.99)
Balanced Protein Suppl.	50%	0.08 (-0.08, 0.25)	0.14 (-0.15, 0.59)	4 (-4, 15)	0.09 (-0.10, 0.39)	0.19 (-0.21, 0.84)	3.27 (-2.92, 11.48)	7.02 (-6.27, 24.62)
	90%	0.14 (-0.14, 0.45)	0.25 (-0.28, 1.05)	7 (-7, 28)	0.16 (-0.18, 0.70)	0.34 (-0.38, 1.51)	3.27 (-2.92, 11.48)	7.02 (-6.27, 24.62)

References for Data Inputs

[1] Blencowe H, Krasevec J, Onis M De, et al. Articles National , regional , and worldwide estimates of low birthweight in 2015 , with trends from 2000: a systematic analysis. Lancet Glob Heal. 2019;(18):1-12.

[2] Chawanpaiboon S, Vogel JP, Moller AB, et al. Global, regional, and national estimates of levels of preterm birth in 2014: a systematic review and modelling analysis. Lancet Glob Heal. 2019;7(1):e37-e46.

[3] United National Population Division World Population Prospects 2019.

[4] Fink G, Peet E, Danaei G, et al. Schooling and wage income losses due to early-childhood growth faltering in developing countries: National, regional, and global estimates. Am J Clin Nutr. 2016;104(1):104-112.

[5] Country specific annual wage data from World Indicators Database. Average yearly wage was estimated to be 2/3 of the gross domestic product in 2010 constant US dollars and 2011 International dollars, adjusted for purchasing power parity.

[6] NCD Risk Factor Collaboration. Trends in adult body-mass index in 200 countries from 1975 to 2014: a pooled analysis of 1698 population-based measurement studies with 19·2 million participants. Lancet. 2016;387(10026):1377-1396.

[7] Stevens GA, Finucane MM, De-Regil LM, et al. Global, regional, and national trends in haemoglobin concentration and prevalence of total and severe anaemia in children and pregnant and non-pregnant women for 1995-2011: A systematic analysis of population-representative data. Lancet Glob Heal. 2013;1(1):16-25.

[8] Coverage of iron-folic acid supplementation abstracted from the most recent Demographic Health Survey or imputed based on sub-regional average. Indicator used: % women in the past five years who took iron tablets or syrup for >90 days.

Libya

Region: North Africa and Middle East**; Sub-region:** North Africa and Middle East

Low birthweight prevalence[1]: 4.0% (95% CI: 0.0, 8.0)

Preterm birth prevalence[2]: 10.3% (95% CI: 9.1, 11.6)

Number of births[3]: 631,000

Returns to education[4]: 6.7% (95% CI: 4.1, 9.4)

GDP per capita 2010 US$ (estimated annual wage)[5]: $5900 ($3933/year)

GDP per capita 2011 International $ (estimated annual wage)[5]: $14423 ($9615/year)

Prevalence of low BMI[6]: 1.7% (95% CI: 0.6, 3.6)

Prevalence of anemia[7]: 25.8% (95% CI: 14.6, 45.2)

Baseline coverage of IFA[8]: 24.3%

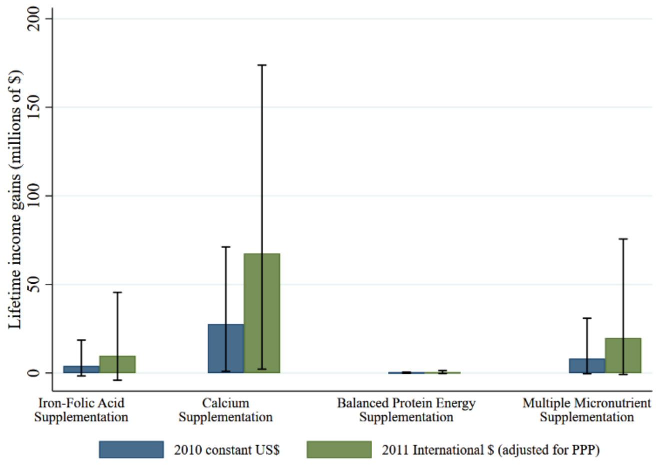

Figure: Benefits by birth cohort size for estimated gains in lifetime wages attributable to scaling up nutrition interventions to 90% target coverage

Table: Impact of maternal prenatal nutrition intervention on human capital and labour market outcomes, through improvements in low birthweight or preterm birth and schooling in Libya.

Intervention	Target Coverage (%)	Absolute reduction in birth outcome (%)	Benefits by cohorts: School years gained (in 1000 s)	No. of additional students completing secondary school	Benefits by cohorts: Lifetime wages		Returns in lifetime earnings per child born to a targeted pregnant woman	
					in US $ millions	in International $ millions	in US $	in International $
Iron/Folic Acid Suppl.	90%	0.38 (-0.14, 1.24)	0.57 (-0.22, 2.47)	7 (-3, 30)	3.99 (-1.64, 18.65)	9.75 (-4.01, 45.59)	7.03 (-2.89, 32.84)	17.17 (-7.05, 80.29)
Calcium Suppl.	50%	1.24 (0.28, 2.08)	2.22 (0.07, 5.31)	27 (1, 64)	15.34 (0.51, 39.50)	37.51 (1.24, 96.57)	48.63 (1.60, 125.21)	118.89 (3.92, 306.09)
	90%	2.22 (0.50, 3.75)	4.00 (0.12, 9.57)	48 (1, 115)	27.62 (0.91, 71.11)	67.52 (2.23, 173.83)	48.63 (1.60, 125.21)	118.89 (3.92, 306.09)
Multiple Micronutrient Suppl.	50%	0.35 (-0.01, 0.82)	0.56 (-0.02, 1.83)	7 (-0, 22)	3.90 (-0.16, 14.37)	9.53 (-0.39, 35.14)	12.35 (-0.51, 45.56)	30.19 (-1.25, 111.38)
	90%	0.75 (-0.02, 1.84)	1.17 (-0.04, 3.99)	14 (-0, 48)	8.10 (-0.34, 30.94)	19.80 (-0.84, 75.64)	14.26 (-0.60, 54.48)	34.87 (-1.48, 133.19)
Balanced Protein Suppl.	50%	0.00 (-0.00, 0.03)	0.01 (-0.01, 0.04)	0 (-0, 1)	0.04 (-0.06, 0.33)	0.10 (-0.15, 0.80)	9.24 (-12.88, 50.11)	22.60 (-31.48, 122.51)
	90%	0.01 (-0.01, 0.05)	0.01 (-0.01, 0.08)	0 (-0, 1)	0.07 (-0.11, 0.59)	0.18 (-0.28, 1.44)	9.24 (-12.88, 50.11)	22.60 (-31.48, 122.51)

References for Data Inputs

[1] Blencowe H, Krasevec J, Onis M De, et al. Articles National , regional , and worldwide estimates of low birthweight in 2015 , with trends from 2000: a systematic analysis. Lancet Glob Heal. 2019;(18):1-12.

[2] Chawanpaiboon S, Vogel JP, Moller AB, et al. Global, regional, and national estimates of levels of preterm birth in 2014: a systematic review and modelling analysis. Lancet Glob Heal. 2019;7(1):e37-e46.

[3] United National Population Division World Population Prospects 2019.

[4] Fink G, Peet E, Danaei G, et al. Schooling and wage income losses due to early-childhood growth faltering in developing countries: National, regional, and global estimates. Am J Clin Nutr. 2016;104(1):104-112.

[5] Country specific annual wage data from World Indicators Database. Average yearly wage was estimated to be 2/3 of the gross domestic product in 2010 constant US dollars and 2011 International dollars, adjusted for purchasing power parity.

[6] NCD Risk Factor Collaboration. Trends in adult body-mass index in 200 countries from 1975 to 2014: a pooled analysis of 1698 population-based measurement studies with 19·2 million participants. Lancet. 2016;387(10026):1377-1396.

[7] Stevens GA, Finucane MM, De-Regil LM, et al. Global, regional, and national trends in haemoglobin concentration and prevalence of total and severe anaemia in children and pregnant and non-pregnant women for 1995-2011: A systematic analysis of population-representative data. Lancet Glob Heal. 2013;1(1):16-25.

[8] Coverage of iron-folic acid supplementation abstracted from the most recent Demographic Health Survey or imputed based on sub-regional average. Indicator used: % women in the past five years who took iron tablets or syrup for >90 days.

Macedonia

Region: Central Europe, Eastern Europe, Central Asia**; Sub-region:** Central Europe

Low birthweight prevalence[1]: 9.1% (95% CI: 8.4, 9.7)

Preterm birth prevalence[2]: 8.7% (95% CI: 7.2, 10.2)

Number of births[3]: 113,000

Returns to education[4]: 6.7% (95% CI: 5.3, 8.1)

GDP per capita 2010 US$ (estimated annual wage)[5]: $5105 ($3404/year)

GDP per capita 2011 International $ (estimated annual wage)[5]: $12761 ($8508/year)

Prevalence of low BMI[6]: 2.3% (95% CI: 0.7, 5.0)

Prevalence of anemia[7]: 34.6% (95% CI: 26.4, 44.7)

Baseline coverage of IFA[8]: 18.5%

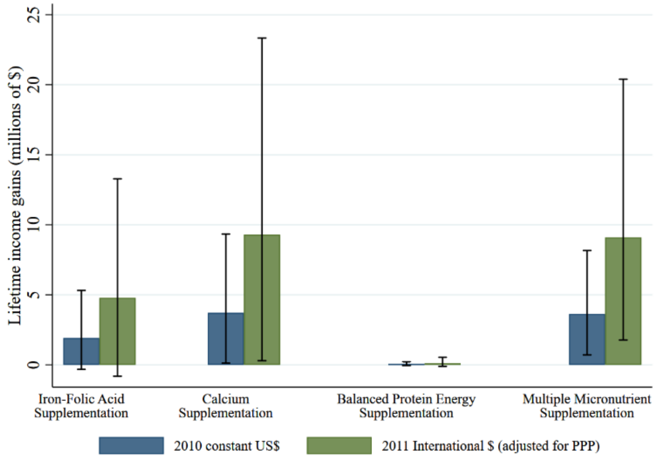

Figure: Benefits by birth cohort size for estimated gains in lifetime wages attributable to scaling up nutrition interventions to 90% target coverage

Table: Impact of maternal prenatal nutrition intervention on human capital and labour market outcomes, through improvements in low birthweight or preterm birth and schooling in Macedonia.

Intervention	Target Coverage (%)	Absolute reduction in birth outcome (%)	Benefits by cohorts: School years gained (in 1000 s)	No. of additional students completing secondary school	Benefits by cohorts: Lifetime wages		Returns in lifetime earnings per child born to a targeted pregnant woman	
					in US $ millions	in International $ millions	in US $	in International $
Iron/Folic Acid Suppl.	90%	1.08 (-0.20, 2.15)	0.31 (-0.06, 0.83)	14 (-3, 37)	1.91 (-0.32, 5.31)	4.78 (-0.81, 13.28)	18.80 (-3.18, 52.24)	47.00 (-7.94, 130.58)
Calcium Suppl.	50%	1.04 (0.23, 1.81)	0.34 (0.01, 0.83)	15 (0, 37)	2.07 (0.07, 5.19)	5.16 (0.17, 12.96)	36.55 (1.18, 91.80)	91.36 (2.95, 229.46)
	90%	1.87 (0.41, 3.26)	0.61 (0.02, 1.49)	27 (1, 66)	3.72 (0.12, 9.34)	9.29 (0.30, 23.34)	36.55 (1.18, 91.80)	91.36 (2.95, 229.46)
Multiple Micronutrient Suppl.	50%	0.96 (0.45, 1.38)	0.29 (0.06, 0.62)	13 (3, 27)	1.80 (0.37, 3.97)	4.51 (0.93, 9.91)	31.92 (6.61, 70.20)	79.79 (16.53, 175.48)
	90%	1.96 (0.82, 2.90)	0.59 (0.12, 1.26)	26 (5, 56)	3.64 (0.71, 8.16)	9.09 (1.77, 20.40)	35.78 (6.96, 80.24)	89.43 (17.41, 200.58)
Balanced Protein Suppl.	50%	0.02 (-0.01, 0.05)	0.00 (-0.00, 0.02)	0 (-0, 1)	0.03 (-0.02, 0.12)	0.07 (-0.06, 0.30)	23.43 (-22.99, 74.12)	58.56 (-57.46, 185.28)
	90%	0.03 (-0.02, 0.10)	0.01 (-0.01, 0.03)	0 (-0, 2)	0.05 (-0.04, 0.22)	0.12 (-0.11, 0.54)	23.43 (-22.99, 74.12)	58.56 (-57.46, 185.28)

References for Data Inputs

[1] Blencowe H, Krasevec J, Onis M De, et al. Articles National , regional , and worldwide estimates of low birthweight in 2015 , with trends from 2000: a systematic analysis. Lancet Glob Heal. 2019;(18):1-12.
[2] Chawanpaiboon S, Vogel JP, Moller AB, et al. Global, regional, and national estimates of levels of preterm birth in 2014: a systematic review and modelling analysis. Lancet Glob Heal. 2019;7(1):e37-e46.
[3] United National Population Division World Population Prospects 2019.
[4] Fink G, Peet E, Danaei G, et al. Schooling and wage income losses due to early-childhood growth faltering in developing countries: National, regional, and global estimates. Am J Clin Nutr. 2016;104(1):104-112.
[5] Country specific annual wage data from World Indicators Database. Average yearly wage was estimated to be 2/3 of the gross domestic product in 2010 constant US dollars and 2011 International dollars, adjusted for purchasing power parity.
[6] NCD Risk Factor Collaboration. Trends in adult body-mass index in 200 countries from 1975 to 2014: a pooled analysis of 1698 population-based measurement studies with 19·2 million participants. Lancet. 2016;387(10026):1377-1396.
[7] Stevens GA, Finucane MM, De-Regil LM, et al. Global, regional, and national trends in haemoglobin concentration and prevalence of total and severe anaemia in children and pregnant and non-pregnant women for 1995-2011: A systematic analysis of population-representative data. Lancet Glob Heal. 2013;1(1):16-25.
[8] Coverage of iron-folic acid supplementation abstracted from the most recent Demographic Health Survey or imputed based on sub-regional average. Indicator used: % women in the past five years who took iron tablets or syrup for >90 days.

Madagascar

Region: Sub-Saharan Africa**; Sub-region:** Eastern Sub-Saharan Africa

Low birthweight prevalence[1]: 17.1% (95% CI: 11.7, 22.9)

Preterm birth prevalence[2]: 12.0% (95% CI: 8.6, 16.7)

Number of births[3]: 4,260,000

Returns to education[4]: 11.3% (95% CI: 9.7, 12.9)

GDP per capita 2010 US$ (estimated annual wage)[5]: $410 ($273/year)

GDP per capita 2011 International $ (estimated annual wage)[5]: $1377 ($918/year)

Prevalence of low BMI[6]: 14.6% (95% CI: 8.1, 22.6)

Prevalence of anemia[7]: 38.6% (95% CI: 30.8, 46.9)

Baseline coverage of IFA[8]: 19.2%

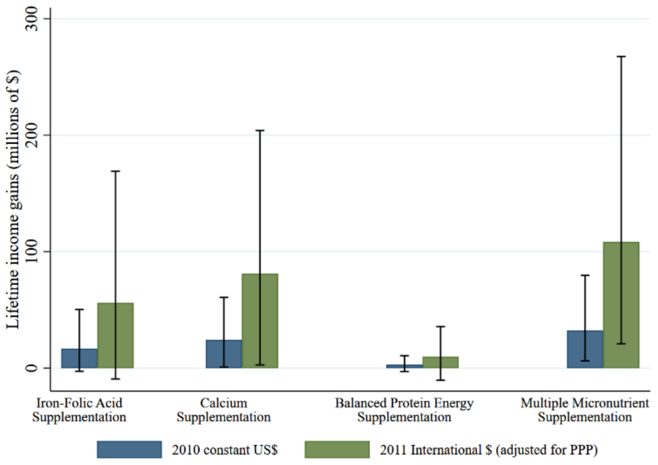

Figure: Benefits by birth cohort size for estimated gains in lifetime wages attributable to scaling up nutrition interventions to 90% target coverage

Table: Impact of maternal prenatal nutrition intervention on human capital and labour market outcomes, through improvements in low birthweight or preterm birth and schooling in Madagascar.

Intervention	Target Coverage (%)	Absolute reduction in birth outcome (%)	Benefits by cohorts: School years gained (in 1000 s)	No. of additional students completing secondary school	Benefits by cohorts: Lifetime wages		Returns in lifetime earnings per child born to a targeted pregnant woman	
					in US $ millions	in International $ millions	in US $	in International $
**Iron/Folic Acid Suppl.**	90%	1.98 (-0.40, 4.35)	19.99 (-3.13, 59.44)	548 (-86, 1630)	16.68 (-2.80, 50.35)	56.00 (-9.38, 169.06)	4.35 (-0.73, 13.13)	14.61 (-2.45, 44.09)
**Calcium Suppl.**	50%	1.40 (0.32, 2.67)	16.12 (0.52, 40.16)	442 (14, 1101)	13.42 (0.46, 33.76)	45.07 (1.53, 113.33)	6.30 (0.21, 15.85)	21.16 (0.72, 53.21)
	90%	2.52 (0.58, 4.81)	29.02 (0.93, 72.29)	796 (26, 1982)	24.16 (0.82, 60.76)	81.12 (2.75, 204.00)	6.30 (0.21, 15.85)	21.16 (0.72, 53.21)
**Multiple Micronutrient Suppl.**	50%	1.80 (0.81, 2.96)	19.21 (3.89, 44.96)	527 (107, 1233)	15.95 (3.25, 38.08)	53.55 (10.92, 127.84)	7.49 (1.53, 17.88)	25.14 (5.13, 60.02)
	90%	3.66 (1.47, 6.19)	38.92 (7.71, 94.45)	1067 (211, 2590)	32.28 (6.20, 79.66)	108.38 (20.81, 267.44)	8.42 (1.62, 20.78)	28.27 (5.43, 69.75)
**Balanced Protein Suppl.**	50%	0.20 (-0.16, 0.57)	1.95 (-1.99, 7.05)	54 (-55, 193)	1.62 (-1.71, 5.90)	5.44 (-5.75, 19.82)	5.51 (-5.32, 17.94)	18.49 (-17.85, 60.23)
	90%	0.36 (-0.29, 1.02)	3.51 (-3.58, 12.68)	96 (-98, 348)	2.92 (-3.08, 10.62)	9.79 (-10.35, 35.67)	5.51 (-5.32, 17.94)	18.49 (-17.85, 60.23)

References for Data Inputs
[1] Blencowe H, Krasevec J, Onis M De, et al. Articles National , regional , and worldwide estimates of low birthweight in 2015 , with trends from 2000: a systematic analysis. Lancet Glob Heal. 2019;(18):1-12.
[2] Chawanpaiboon S, Vogel JP, Moller AB, et al. Global, regional, and national estimates of levels of preterm birth in 2014: a systematic review and modelling analysis. Lancet Glob Heal. 2019;7(1):e37-e46.
[3] United National Population Division World Population Prospects 2019.
[4] Fink G, Peet E, Danaei G, et al. Schooling and wage income losses due to early-childhood growth faltering in developing countries: National, regional, and global estimates. Am J Clin Nutr. 2016;104(1):104-112.
[5] Country specific annual wage data from World Indicators Database. Average yearly wage was estimated to be 2/3 of the gross domestic product in 2010 constant US dollars and 2011 International dollars, adjusted for purchasing power parity.
[6] NCD Risk Factor Collaboration. Trends in adult body-mass index in 200 countries from 1975 to 2014: a pooled analysis of 1698 population-based measurement studies with 19·2 million participants. Lancet. 2016;387(10026):1377-1396.
[7] Stevens GA, Finucane MM, De-Regil LM, et al. Global, regional, and national trends in haemoglobin concentration and prevalence of total and severe anaemia in children and pregnant and non-pregnant women for 1995-2011: A systematic analysis of population-representative data. Lancet Glob Heal. 2013;1(1):16-25.
[8] Coverage of iron-folic acid supplementation abstracted from the most recent Demographic Health Survey or imputed based on sub-regional average. Indicator used: % women in the past five years who took iron tablets or syrup for >90 days.

Malawi

Region: Sub-Saharan Africa**; Sub-region:** Eastern Sub-Saharan Africa

Low birthweight prevalence[1]: 14.5% (95% CI: 11.3, 18.5)

Preterm birth prevalence[2]: 10.5% (95% CI: 7.4, 14.3)

Number of births[3]: 3,072,000

Returns to education[4]: 12.2% (95% CI: 10.6, 13.7)

GDP per capita 2010 US$ (estimated annual wage)[5]: $508 ($338/year)

GDP per capita 2011 International $ (estimated annual wage)[5]: $1143 ($762/year)

Prevalence of low BMI[6]: 9.0% (95% CI: 4.6, 14.9)

Prevalence of anemia[7]: 27.0% (95% CI: 12.1, 51.2)

Baseline coverage of IFA[8]: 33.4%

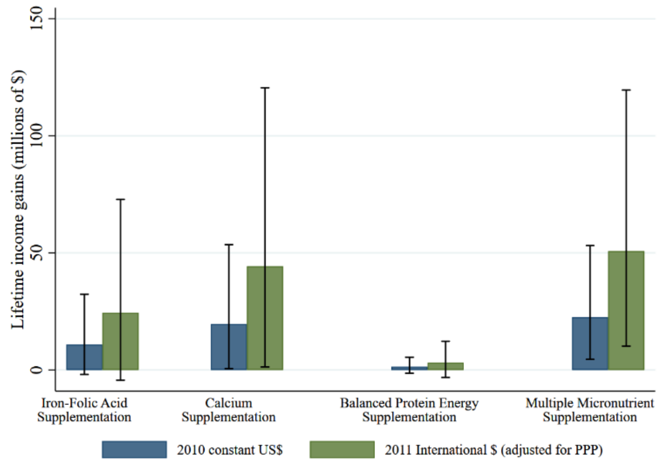

Figure: Benefits by birth cohort size for estimated gains in lifetime wages attributable to scaling up nutrition interventions to 90% target coverage

Table: Impact of maternal prenatal nutrition intervention on human capital and labour market outcomes, through improvements in low birthweight or preterm birth and schooling in Malawi.

Intervention	Target Coverage (%)	Absolute reduction in birth outcome (%)	Benefits by cohorts: School years gained (in 1000 s)	No. of additional students completing secondary school	Benefits by cohorts: Lifetime wages		Returns in lifetime earnings per child born to a targeted pregnant woman	
					in US $ millions	in International $ millions	in US $	in International $
Iron/Folic Acid Suppl.	90%	1.37 (-0.26, 2.91)	9.97 (-1.74, 27.96)	305 (-53, 856)	10.86 (-1.93, 32.34)	24.44 (-4.35, 72.80)	3.93 (-0.70, 11.70)	8.84 (-1.57, 26.33)
Calcium Suppl.	50%	1.23 (0.24, 2.34)	9.98 (0.29, 26.43)	305 (9, 809)	10.93 (0.31, 29.74)	24.60 (0.70, 66.96)	7.11 (0.20, 19.36)	16.01 (0.45, 43.59)
	90%	2.21 (0.44, 4.21)	17.97 (0.52, 47.57)	550 (16, 1456)	19.67 (0.56, 53.54)	44.28 (1.25, 120.52)	7.11 (0.20, 19.36)	16.01 (0.45, 43.59)
Multiple Micronutrient Suppl.	50%	1.13 (0.59, 1.74)	8.71 (2.04, 19.40)	266 (62, 594)	9.64 (2.19, 21.77)	21.70 (4.94, 49.00)	6.28 (1.43, 14.17)	14.13 (3.21, 31.90)
	90%	2.72 (1.19, 4.38)	20.39 (4.04, 47.03)	624 (123, 1439)	22.54 (4.53, 53.09)	50.74 (10.20, 119.52)	8.15 (1.64, 19.20)	18.35 (3.69, 43.23)
Balanced Protein Suppl.	50%	0.10 (-0.10, 0.29)	0.70 (-0.71, 2.66)	21 (-22, 81)	0.78 (-0.79, 3.02)	1.75 (-1.78, 6.81)	6.06 (-5.84, 19.65)	13.64 (-13.15, 44.23)
	90%	0.18 (-0.17, 0.53)	1.26 (-1.27, 4.79)	38 (-39, 147)	1.40 (-1.43, 5.44)	3.15 (-3.21, 12.25)	6.06 (-5.84, 19.65)	13.64 (-13.15, 44.23)

References for Data Inputs

[1] Blencowe H, Krasevec J, Onis M De, et al. Articles National , regional , and worldwide estimates of low birthweight in 2015 , with trends from 2000: a systematic analysis. Lancet Glob Heal. 2019;(18):1-12.
[2] Chawanpaiboon S, Vogel JP, Moller AB, et al. Global, regional, and national estimates of levels of preterm birth in 2014: a systematic review and modelling analysis. Lancet Glob Heal. 2019;7(1):e37-e46.
[3] United National Population Division World Population Prospects 2019.
[4] Fink G, Peet E, Danaei G, et al. Schooling and wage income losses due to early-childhood growth faltering in developing countries: National, regional, and global estimates. Am J Clin Nutr. 2016;104(1):104-112.
[5] Country specific annual wage data from World Indicators Database. Average yearly wage was estimated to be 2/3 of the gross domestic product in 2010 constant US dollars and 2011 International dollars, adjusted for purchasing power parity.
[6] NCD Risk Factor Collaboration. Trends in adult body-mass index in 200 countries from 1975 to 2014: a pooled analysis of 1698 population-based measurement studies with 19·2 million participants. Lancet. 2016;387(10026):1377-1396.
[7] Stevens GA, Finucane MM, De-Regil LM, et al. Global, regional, and national trends in haemoglobin concentration and prevalence of total and severe anaemia in children and pregnant and non-pregnant women for 1995-2011: A systematic analysis of population-representative data. Lancet Glob Heal. 2013;1(1):16-25.
[8] Coverage of iron-folic acid supplementation abstracted from the most recent Demographic Health Survey or imputed based on sub-regional average. Indicator used: % women in the past five years who took iron tablets or syrup for >90 days.

Malaysia

Region: Southeast Asia, East Asia, and Oceania**; Sub-region:** Southeast Asia

Low birthweight prevalence[1]: 11.3% (95% CI: 11.1, 11.6)

Preterm birth prevalence[2]: 10.4% (95% CI: 8.7, 11.9)

Number of births[3]: 2,636,000

Returns to education[4]: 6.5% (95% CI: 2.1, 10.9)

GDP per capita 2010 US$ (estimated annual wage)[5]: $10912 ($7275/year)

GDP per capita 2011 International $ (estimated annual wage)[5]: $25390 ($16927/year)

Prevalence of low BMI[6]: 6.9% (95% CI: 4.3, 10.3)

Prevalence of anemia[7]: 40.3% (95% CI: 21.9, 63.1)

Baseline coverage of IFA[8]: 46.2%

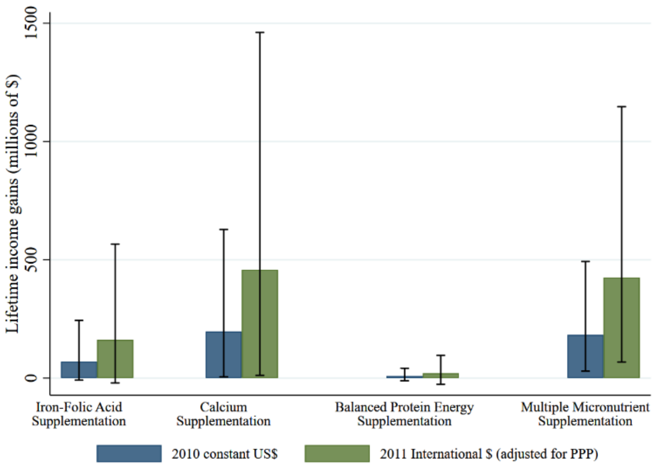

Figure: Benefits by birth cohort size for estimated gains in lifetime wages attributable to scaling up nutrition interventions to 90% target coverage

Table: Impact of maternal prenatal nutrition intervention on human capital and labour market outcomes, through improvements in low birthweight or preterm birth and schooling in Malaysia.

Intervention	Target Coverage (%)	Absolute reduction in birth outcome (%)	Benefits by cohorts: School years gained (in 1000 s)	No. of additional students completing secondary school	Benefits by cohorts: Lifetime wages in US $ millions	Benefits by cohorts: Lifetime wages in International $ millions	Returns in lifetime earnings per child born to a targeted pregnant woman in US $	Returns in lifetime earnings per child born to a targeted pregnant woman in International $
Iron/Folic Acid Suppl.	90%	0.86 (-0.15, 1.78)	5.89 (-1.00, 16.11)	105 (-18, 287)	69.47 (-8.97, 243.34)	161.65 (-20.87, 566.20)	29.28 (-3.78, 102.57)	68.14 (-8.80, 238.66)
Calcium Suppl.	50%	1.24 (0.26, 2.14)	9.37 (0.32, 23.13)	167 (6, 412)	109.29 (2.65, 348.87)	254.29 (6.17, 811.75)	82.92 (2.01, 264.70)	192.94 (4.68, 615.90)
	90%	2.23 (0.47, 3.85)	16.87 (0.57, 41.63)	300 (10, 741)	196.72 (4.77, 627.97)	457.73 (11.11, 1461.16)	82.92 (2.01, 264.70)	192.94 (4.68, 615.90)
Multiple Micronutrient Suppl.	50%	0.74 (0.43, 1.02)	5.46 (1.14, 11.38)	97 (20, 203)	66.52 (8.93, 172.97)	154.78 (20.79, 402.47)	50.47 (6.78, 131.24)	117.43 (15.77, 305.36)
	90%	2.06 (1.04, 2.98)	14.73 (3.27, 32.39)	262 (58, 577)	182.70 (28.99, 492.93)	425.11 (67.44, 1146.94)	77.01 (12.22, 207.78)	179.19 (28.43, 483.45)
Balanced Protein Suppl.	50%	0.06 (-0.06, 0.17)	0.42 (-0.45, 1.53)	7 (-8, 27)	4.83 (-6.28, 22.84)	11.24 (-14.62, 53.15)	55.49 (-65.53, 228.16)	129.10 (-152.48, 530.88)
	90%	0.12 (-0.10, 0.30)	0.75 (-0.82, 2.75)	13 (-15, 49)	8.69 (-11.31, 41.11)	20.22 (-26.31, 95.66)	55.49 (-65.53, 228.16)	129.10 (-152.48, 530.88)

References for Data Inputs

[1] Blencowe H, Krasevec J, Onis M De, et al. Articles National , regional , and worldwide estimates of low birthweight in 2015 , with trends from 2000: a systematic analysis. Lancet Glob Heal. 2019;(18):1-12.
[2] Chawanpaiboon S, Vogel JP, Moller AB, et al. Global, regional, and national estimates of levels of preterm birth in 2014: a systematic review and modelling analysis. Lancet Glob Heal. 2019;7(1):e37-e46.
[3] United National Population Division World Population Prospects 2019.
[4] Fink G, Peet E, Danaei G, et al. Schooling and wage income losses due to early-childhood growth faltering in developing countries: National, regional, and global estimates. Am J Clin Nutr. 2016;104(1):104-112.
[5] Country specific annual wage data from World Indicators Database. Average yearly wage was estimated to be 2/3 of the gross domestic product in 2010 constant US dollars and 2011 International dollars, adjusted for purchasing power parity.
[6] NCD Risk Factor Collaboration. Trends in adult body-mass index in 200 countries from 1975 to 2014: a pooled analysis of 1698 population-based measurement studies with 19·2 million participants. Lancet. 2016;387(10026):1377-1396.
[7] Stevens GA, Finucane MM, De-Regil LM, et al. Global, regional, and national trends in haemoglobin concentration and prevalence of total and severe anaemia in children and pregnant and non-pregnant women for 1995-2011: A systematic analysis of population-representative data. Lancet Glob Heal. 2013;1(1):16-25.
[8] Coverage of iron-folic acid supplementation abstracted from the most recent Demographic Health Survey or imputed based on sub-regional average. Indicator used: % women in the past five years who took iron tablets or syrup for >90 days.

Maldives

Region: Southeast Asia, East Asia, and Oceania**; Sub-region:** Southeast Asia

Low birthweight prevalence[1]: 11.7% (95% CI: 8.0, 17.9)

Preterm birth prevalence[2]: 6.2% (95% CI: 3.7, 9.8)

Number of births[3]: 36,000

Returns to education[4]: 6.5% (95% CI: 2.1, 10.9)

GDP per capita 2010 US$ (estimated annual wage)[5]: $7502 ($5001/year)

GDP per capita 2011 International $ (estimated annual wage)[5]: $12684 ($8456/year)

Prevalence of low BMI[6]: 8.4% (95% CI: 4.0, 14.5)

Prevalence of anemia[7]: 61.6% (95% CI: 50.7, 69.2)

Baseline coverage of IFA[8]: 46.3%

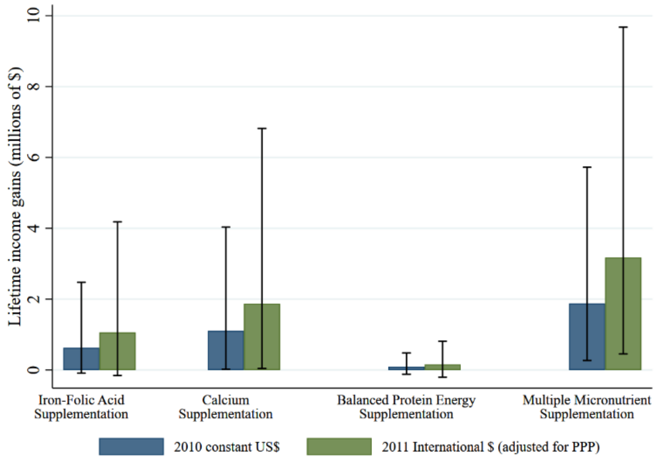

Figure: Benefits by birth cohort size for estimated gains in lifetime wages attributable to scaling up nutrition interventions to 90% target coverage

Table: Impact of maternal prenatal nutrition intervention on human capital and labour market outcomes, through improvements in low birthweight or preterm birth and schooling in Maldives.

Intervention	Target Coverage (%)	Absolute reduction in birth outcome (%)	Benefits by cohorts: School years gained (in 1000 s)	No. of additional students completing secondary school	Benefits by cohorts: Lifetime wages		Returns in lifetime earnings per child born to a targeted pregnant woman	
					in US $ millions	in International $ millions	in US $	in International $
**Iron/Folic Acid Suppl.**	90%	0.85 (-0.16, 1.99)	0.08 (-0.01, 0.24)	10 (-2, 32)	0.63 (-0.09, 2.47)	1.06 (-0.15, 4.18)	19.36 (-2.78, 76.38)	32.74 (-4.70, 129.15)
**Calcium Suppl.**	50%	0.72 (0.13, 1.43)	0.07 (0.00, 0.21)	10 (0, 27)	0.61 (0.01, 2.24)	1.04 (0.02, 3.79)	34.13 (0.77, 124.47)	57.71 (1.30, 210.45)
	90%	1.30 (0.24, 2.57)	0.13 (0.00, 0.37)	17 (0, 48)	1.11 (0.02, 4.03)	1.87 (0.04, 6.82)	34.13 (0.77, 124.47)	57.71 (1.30, 210.45)
**Multiple Micronutrient Suppl.**	50%	0.85 (0.42, 1.41)	0.09 (0.02, 0.21)	11 (2, 28)	0.72 (0.10, 2.18)	1.22 (0.16, 3.69)	40.09 (5.35, 121.27)	67.79 (9.05, 205.04)
	90%	2.27 (1.09, 3.73)	0.22 (0.04, 0.55)	29 (6, 72)	1.88 (0.27, 5.72)	3.17 (0.46, 9.68)	57.93 (8.32, 176.62)	97.95 (14.06, 298.64)
**Balanced Protein Suppl.**	50%	0.07 (-0.07, 0.24)	0.01 (-0.01, 0.03)	1 (-1, 3)	0.05 (-0.07, 0.27)	0.09 (-0.11, 0.45)	37.38 (-39.13, 160.06)	63.20 (-66.16, 270.64)
	90%	0.13 (-0.12, 0.43)	0.01 (-0.01, 0.05)	2 (-2, 6)	0.09 (-0.12, 0.48)	0.16 (-0.20, 0.81)	37.38 (-39.13, 160.06)	63.20 (-66.16, 270.64)

References for Data Inputs

[1] Blencowe H, Krasevec J, Onis M De, et al. Articles National , regional , and worldwide estimates of low birthweight in 2015 , with trends from 2000: a systematic analysis. Lancet Glob Heal. 2019;(18):1-12.
[2] Chawanpaiboon S, Vogel JP, Moller AB, et al. Global, regional, and national estimates of levels of preterm birth in 2014: a systematic review and modelling analysis. Lancet Glob Heal. 2019;7(1):e37-e46.
[3] United National Population Division World Population Prospects 2019.
[4] Fink G, Peet E, Danaei G, et al. Schooling and wage income losses due to early-childhood growth faltering in developing countries: National, regional, and global estimates. Am J Clin Nutr. 2016;104(1):104-112.
[5] Country specific annual wage data from World Indicators Database. Average yearly wage was estimated to be 2/3 of the gross domestic product in 2010 constant US dollars and 2011 International dollars, adjusted for purchasing power parity.
[6] NCD Risk Factor Collaboration. Trends in adult body-mass index in 200 countries from 1975 to 2014: a pooled analysis of 1698 population-based measurement studies with 19·2 million participants. Lancet. 2016;387(10026):1377-1396.
[7] Stevens GA, Finucane MM, De-Regil LM, et al. Global, regional, and national trends in haemoglobin concentration and prevalence of total and severe anaemia in children and pregnant and non-pregnant women for 1995-2011: A systematic analysis of population-representative data. Lancet Glob Heal. 2013;1(1):16-25.
[8] Coverage of iron-folic acid supplementation abstracted from the most recent Demographic Health Survey or imputed based on sub-regional average. Indicator used: % women in the past five years who took iron tablets or syrup for >90 days.

Mali

Region: Sub-Saharan Africa**; Sub-region:** Western Sub-Saharan Africa

Low birthweight prevalence[1]: 18.7% (95% CI: 13.4, 24.1)

Preterm birth prevalence[2]: 12.0% (95% CI: 8.6, 16.7)

Number of births[3]: 3,937,000

Returns to education[4]: 6.3% (95% CI: 4.5, 8.1)

GDP per capita 2010 US$ (estimated annual wage)[5]: $727 ($485/year)

GDP per capita 2011 International $ (estimated annual wage)[5]: $1922 ($1282/year)

Prevalence of low BMI[6]: 9.6% (95% CI: 4.9, 15.8)

Prevalence of anemia[7]: 28.2% (95% CI: 10.2, 55.3)

Baseline coverage of IFA[8]: 18.3%

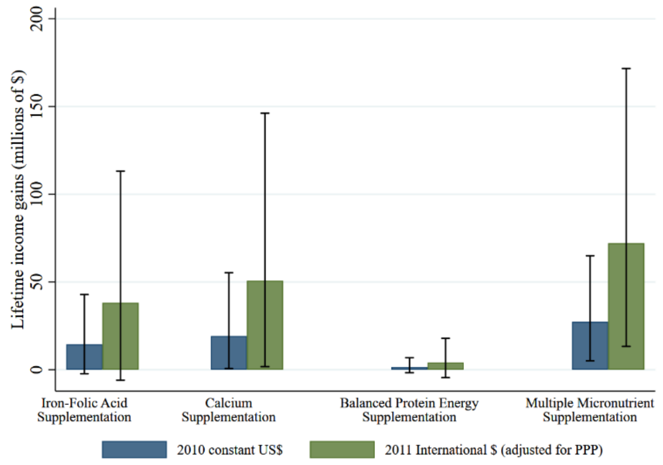

Figure: Benefits by birth cohort size for estimated gains in lifetime wages attributable to scaling up nutrition interventions to 90% target coverage

Table: Impact of maternal prenatal nutrition intervention on human capital and labour market outcomes, through improvements in low birthweight or preterm birth and schooling in Mali.

Intervention	Target Coverage (%)	Absolute reduction in birth outcome (%)	Benefits by cohorts: School years gained (in 1000 s)	No. of additional students completing secondary school	Benefits by cohorts: Lifetime wages		Returns in lifetime earnings per child born to a targeted pregnant woman	
					in US $ millions	in International $ millions	in US $	in International $
Iron/Folic Acid Suppl.	90%	2.14 (-0.40, 4.57)	18.28 (-3.35, 51.72)	355 (-65, 1003)	14.45 (-2.25, 42.83)	38.18 (-5.95, 113.18)	4.08 (-0.64, 12.09)	10.78 (-1.68, 31.94)
Calcium Suppl.	50%	1.40 (0.30, 2.57)	13.28 (0.41, 36.04)	258 (8, 699)	10.66 (0.36, 30.72)	28.17 (0.96, 81.18)	5.41 (0.19, 15.61)	14.31 (0.49, 41.24)
	90%	2.52 (0.53, 4.62)	23.90 (0.74, 64.87)	464 (14, 1259)	19.18 (0.66, 55.30)	50.70 (1.73, 146.13)	5.41 (0.19, 15.61)	14.31 (0.49, 41.24)
Multiple Micronutrient Suppl.	50%	1.90 (0.79, 3.03)	16.90 (3.52, 36.88)	328 (68, 716)	13.64 (2.81, 31.71)	36.05 (7.41, 83.79)	6.93 (1.43, 16.11)	18.31 (3.77, 42.57)
	90%	3.85 (1.47, 6.36)	34.07 (6.45, 75.38)	661 (125, 1462)	27.27 (5.04, 64.94)	72.06 (13.32, 171.61)	7.70 (1.42, 18.33)	20.34 (3.76, 48.43)
Balanced Protein Suppl.	50%	0.13 (-0.11, 0.41)	1.10 (-1.12, 4.53)	21 (-22, 88)	0.88 (-0.95, 3.77)	2.32 (-2.51, 9.95)	5.13 (-5.53, 17.72)	13.56 (-14.63, 46.83)
	90%	0.24 (-0.20, 0.75)	1.98 (-2.02, 8.16)	38 (-39, 158)	1.58 (-1.71, 6.78)	4.17 (-4.53, 17.92)	5.13 (-5.53, 17.72)	13.56 (-14.63, 46.83)

References for Data Inputs

[1] Blencowe H, Krasevec J, Onis M De, et al. Articles National , regional , and worldwide estimates of low birthweight in 2015 , with trends from 2000: a systematic analysis. Lancet Glob Heal. 2019;(18):1-12.

[2] Chawanpaiboon S, Vogel JP, Moller AB, et al. Global, regional, and national estimates of levels of preterm birth in 2014: a systematic review and modelling analysis. Lancet Glob Heal. 2019;7(1):e37-e46.

[3] United National Population Division World Population Prospects 2019.

[4] Fink G, Peet E, Danaei G, et al. Schooling and wage income losses due to early-childhood growth faltering in developing countries: National, regional, and global estimates. Am J Clin Nutr. 2016;104(1):104-112.

[5] Country specific annual wage data from World Indicators Database. Average yearly wage was estimated to be 2/3 of the gross domestic product in 2010 constant US dollars and 2011 International dollars, adjusted for purchasing power parity.

[6] NCD Risk Factor Collaboration. Trends in adult body-mass index in 200 countries from 1975 to 2014: a pooled analysis of 1698 population-based measurement studies with 19·2 million participants. Lancet. 2016;387(10026):1377-1396.

[7] Stevens GA, Finucane MM, De-Regil LM, et al. Global, regional, and national trends in haemoglobin concentration and prevalence of total and severe anaemia in children and pregnant and non-pregnant women for 1995-2011: A systematic analysis of population-representative data. Lancet Glob Heal. 2013;1(1):16-25.

[8] Coverage of iron-folic acid supplementation abstracted from the most recent Demographic Health Survey or imputed based on sub-regional average. Indicator used: % women in the past five years who took iron tablets or syrup for >90 days.

Mauritania

Region: Sub-Saharan Africa**; Sub-region:** Western Sub-Saharan Africa

Low birthweight prevalence[1]: 33.7% (95% CI: 28.4, 39.0)

Preterm birth prevalence[2]: 12.0% (95% CI: 8.6, 16.7)

Number of births[3]: 736,000

Returns to education[4]: 6.3% (95% CI: 4.5, 8.1)

GDP per capita 2010 US$ (estimated annual wage)[5]: $1349 ($899/year)

GDP per capita 2011 International $ (estimated annual wage)[5]: $3723 ($2482/year)

Prevalence of low BMI[6]: 7.6% (95% CI: 3.3, 13.8)

Prevalence of anemia[7]: 31.3% (95% CI: 15.9, 51.6)

Baseline coverage of IFA[8]: 36.8%

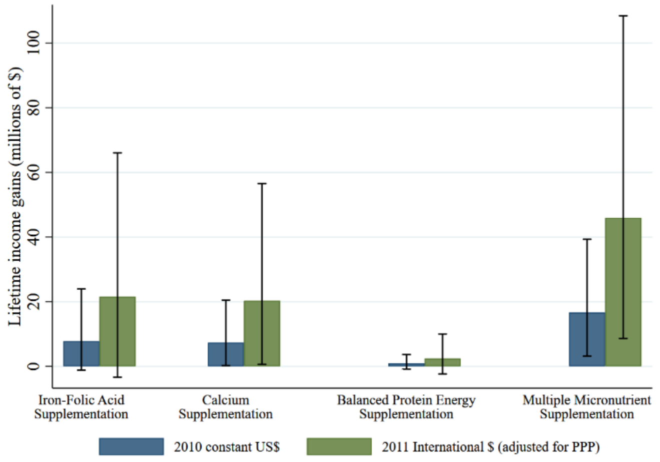

Figure: Benefits by birth cohort size for estimated gains in lifetime wages attributable to scaling up nutrition interventions to 90% target coverage

Table: Impact of maternal prenatal nutrition intervention on human capital and labour market outcomes, through improvements in low birthweight or preterm birth and schooling in Mauritania.

Intervention	Target Coverage (%)	Absolute reduction in birth outcome (%)	Benefits by cohorts: School years gained (in 1000 s)	No. of additional students completing secondary school	Benefits by cohorts: Lifetime wages		Returns in lifetime earnings per child born to a targeted pregnant woman	
					in US $ millions	in International $ millions	in US $	in International $
Iron/Folic Acid Suppl.	90%	3.10 (-0.53, 6.36)	5.21 (-0.86, 14.29)	91 (-15, 249)	7.80 (-1.22, 23.93)	21.53 (-3.36, 66.06)	11.78 (-1.84, 36.13)	32.51 (-5.07, 99.72)
Calcium Suppl.	50%	1.42 (0.29, 2.63)	2.71 (0.08, 7.30)	47 (1, 127)	4.08 (0.12, 11.38)	11.27 (0.33, 31.42)	11.10 (0.33, 30.93)	30.63 (0.91, 85.38)
	90%	2.56 (0.52, 4.73)	4.88 (0.15, 13.13)	85 (3, 229)	7.35 (0.22, 20.49)	20.29 (0.60, 56.55)	11.10 (0.33, 30.93)	30.63 (0.91, 85.38)
Multiple Micronutrient Suppl.	50%	2.56 (1.41, 3.60)	4.68 (1.01, 9.70)	81 (18, 169)	6.99 (1.50, 15.91)	19.30 (4.14, 43.92)	19.00 (4.08, 43.24)	52.44 (11.25, 119.35)
	90%	6.34 (2.75, 9.60)	11.19 (2.31, 24.54)	195 (40, 427)	16.65 (3.13, 39.30)	45.96 (8.64, 108.48)	25.14 (4.72, 59.33)	69.38 (13.04, 163.76)
Balanced Protein Suppl.	50%	0.20 (-0.15, 0.58)	0.32 (-0.29, 1.25)	6 (-5, 22)	0.48 (-0.48, 2.01)	1.33 (-1.31, 5.56)	19.04 (-19.36, 61.33)	52.54 (-53.44, 169.27)
	90%	0.35 (-0.27, 1.05)	0.58 (-0.52, 2.25)	10 (-9, 39)	0.86 (-0.86, 3.62)	2.39 (-2.37, 10.00)	19.04 (-19.36, 61.33)	52.54 (-53.44, 169.27)

References for Data Inputs

[1] Blencowe H, Krasevec J, Onis M De, et al. Articles National , regional , and worldwide estimates of low birthweight in 2015 , with trends from 2000: a systematic analysis. Lancet Glob Heal. 2019;(18):1-12.

[2] Chawanpaiboon S, Vogel JP, Moller AB, et al. Global, regional, and national estimates of levels of preterm birth in 2014: a systematic review and modelling analysis. Lancet Glob Heal. 2019;7(1):e37-e46.

[3] United National Population Division World Population Prospects 2019.

[4] Fink G, Peet E, Danaei G, et al. Schooling and wage income losses due to early-childhood growth faltering in developing countries: National, regional, and global estimates. Am J Clin Nutr. 2016;104(1):104-112.

[5] Country specific annual wage data from World Indicators Database. Average yearly wage was estimated to be 2/3 of the gross domestic product in 2010 constant US dollars and 2011 International dollars, adjusted for purchasing power parity.

[6] NCD Risk Factor Collaboration. Trends in adult body-mass index in 200 countries from 1975 to 2014: a pooled analysis of 1698 population-based measurement studies with 19·2 million participants. Lancet. 2016;387(10026):1377-1396.

[7] Stevens GA, Finucane MM, De-Regil LM, et al. Global, regional, and national trends in haemoglobin concentration and prevalence of total and severe anaemia in children and pregnant and non-pregnant women for 1995-2011: A systematic analysis of population-representative data. Lancet Glob Heal. 2013;1(1):16-25.

[8] Coverage of iron-folic acid supplementation abstracted from the most recent Demographic Health Survey or imputed based on sub-regional average. Indicator used: % women in the past five years who took iron tablets or syrup for >90 days.

Mauritius

Region: Southeast Asia, East Asia, and Oceania**; Sub-region:** Southeast Asia

Low birthweight prevalence[1]: 13.9% (95% CI: 8.0, 19.9)

Preterm birth prevalence[2]: 12.0% (95% CI: 8.6, 16.7)

Number of births[3]: 65,000

Returns to education[4]: 6.5% (95% CI: 2.1, 10.9)

GDP per capita 2010 US$ (estimated annual wage)[5]: $9477 ($6318/year)

GDP per capita 2011 International $ (estimated annual wage)[5]: $18879 ($12586/year)

Prevalence of low BMI[6]: 7.0% (95% CI: 3.4, 12.0)

Prevalence of anemia[7]: 21.9% (95% CI: 14.8, 30.6)

Baseline coverage of IFA[8]: 46.2%

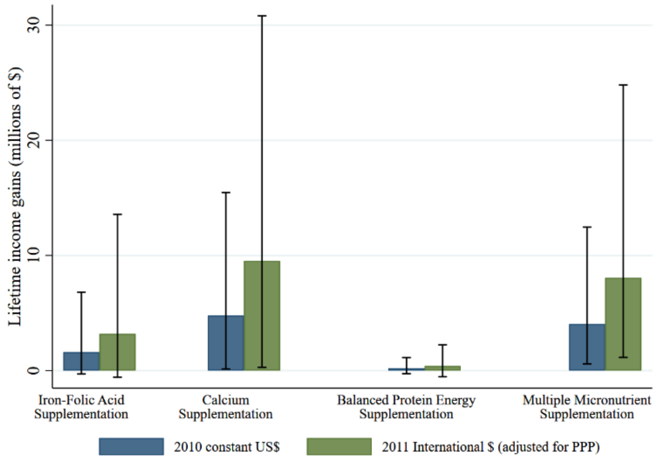

Figure: Benefits by birth cohort size for estimated gains in lifetime wages attributable to scaling up nutrition interventions to 90% target coverage

Table: Impact of maternal prenatal nutrition intervention on human capital and labour market outcomes, through improvements in low birthweight or preterm birth and schooling in Mauritius.

Intervention	Target Coverage (%)	Absolute reduction in birth outcome (%)	Benefits by cohorts: School years gained (in 1000 s)	No. of additional students completing secondary school	Benefits by cohorts: Lifetime wages		Returns in lifetime earnings per child born to a targeted pregnant woman	
					in US $ millions	in International $ millions	in US $	in International $
Iron/Folic Acid Suppl.	90%	1.03 (-0.19, 2.42)	0.17 (-0.02, 0.49)	5 (-1, 14)	1.60 (-0.29, 6.81)	3.20 (-0.57, 13.57)	27.43 (-4.89, 116.43)	54.65 (-9.75, 231.96)
Calcium Suppl.	50%	1.40 (0.28, 2.64)	0.26 (0.01, 0.68)	8 (0, 20)	2.66 (0.08, 8.59)	5.29 (0.16, 17.12)	81.71 (2.43, 264.42)	162.79 (4.84, 526.79)
	90%	2.53 (0.51, 4.76)	0.48 (0.02, 1.22)	14 (0, 36)	4.78 (0.14, 15.47)	9.52 (0.28, 30.82)	81.71 (2.43, 264.42)	162.79 (4.84, 526.79)
Multiple Micronutrient Suppl.	50%	0.78 (0.34, 1.29)	0.14 (0.03, 0.32)	4 (1, 9)	1.41 (0.21, 4.10)	2.80 (0.41, 8.17)	43.24 (6.33, 126.18)	86.15 (12.62, 251.37)
	90%	2.25 (0.90, 4.00)	0.39 (0.08, 0.93)	11 (2, 27)	4.04 (0.58, 12.45)	8.06 (1.15, 24.81)	69.14 (9.84, 212.90)	137.74 (19.61, 424.14)
Balanced Protein Suppl.	50%	0.07 (-0.06, 0.24)	0.01 (-0.01, 0.05)	0 (-0, 1)	0.11 (-0.15, 0.63)	0.22 (-0.29, 1.25)	55.49 (-59.52, 241.18)	110.54 (-118.58, 480.49)
	90%	0.12 (-0.12, 0.43)	0.02 (-0.02, 0.09)	1 (-1, 3)	0.20 (-0.27, 1.13)	0.40 (-0.53, 2.24)	55.49 (-59.52, 241.18)	110.54 (-118.58, 480.49)

References for Data Inputs

[1] Blencowe H, Krasevec J, Onis M De, et al. Articles National , regional , and worldwide estimates of low birthweight in 2015 , with trends from 2000: a systematic analysis. Lancet Glob Heal. 2019;(18):1-12.

[2] Chawanpaiboon S, Vogel JP, Moller AB, et al. Global, regional, and national estimates of levels of preterm birth in 2014: a systematic review and modelling analysis. Lancet Glob Heal. 2019;7(1):e37-e46.

[3] United National Population Division World Population Prospects 2019.

[4] Fink G, Peet E, Danaei G, et al. Schooling and wage income losses due to early-childhood growth faltering in developing countries: National, regional, and global estimates. Am J Clin Nutr. 2016;104(1):104-112.

[5] Country specific annual wage data from World Indicators Database. Average yearly wage was estimated to be 2/3 of the gross domestic product in 2010 constant US dollars and 2011 International dollars, adjusted for purchasing power parity.

[6] NCD Risk Factor Collaboration. Trends in adult body-mass index in 200 countries from 1975 to 2014: a pooled analysis of 1698 population-based measurement studies with 19·2 million participants. Lancet. 2016;387(10026):1377-1396.

[7] Stevens GA, Finucane MM, De-Regil LM, et al. Global, regional, and national trends in haemoglobin concentration and prevalence of total and severe anaemia in children and pregnant and non-pregnant women for 1995-2011: A systematic analysis of population-representative data. Lancet Glob Heal. 2013;1(1):16-25.

[8] Coverage of iron-folic acid supplementation abstracted from the most recent Demographic Health Survey or imputed based on sub-regional average. Indicator used: % women in the past five years who took iron tablets or syrup for >90 days.

Mexico

Region: Latin America and Caribbean**; Sub-region:** Central Latin America

Low birthweight prevalence[1]: 7.9% (95% CI: 6.2, 10.2)

Preterm birth prevalence[2]: 7.0% (95% CI: 5.6, 8.7)

Number of births[3]: 11,120,000

Returns to education[4]: 14.5% (95% CI: 8.1, 20.8)

GDP per capita 2010 US$ (estimated annual wage)[5]: $10037 ($6691/year)

GDP per capita 2011 International $ (estimated annual wage)[5]: $17495 ($11663/year)

Prevalence of low BMI[6]: 1.6% (95% CI: 0.8, 2.5)

Prevalence of anemia[7]: 27.0% (95% CI: 8.8, 53.9)

Baseline coverage of IFA[8]: 33.3%

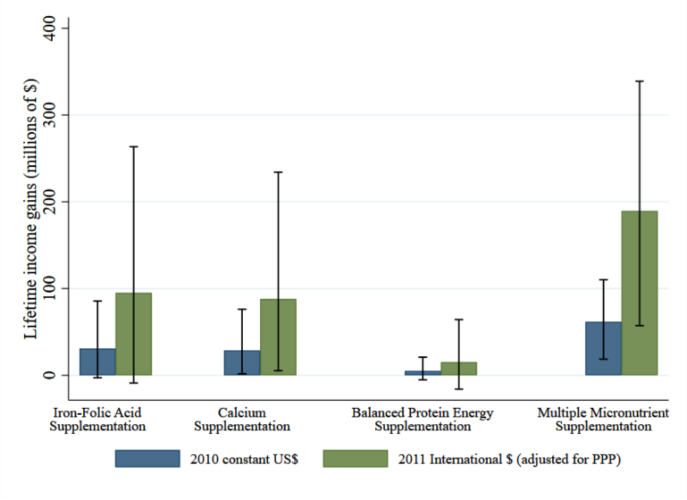

Figure: Benefits by birth cohort size for estimated gains in lifetime wages attributable to scaling up nutrition interventions to 90% target coverage

Table: Impact of maternal prenatal nutrition intervention on human capital and labour market outcomes, through improvements in low birthweight or preterm birth and schooling in Mexico.

Intervention	Target Coverage (%)	Absolute reduction in birth outcome (%)	Benefits by cohorts: School years gained (in 1000 s)	No. of additional students completing secondary school	Benefits by cohorts: Lifetime wages		Returns in lifetime earnings per child born to a targeted pregnant woman	
					in US $ millions	in International $ millions	in US $	in International $
Iron/Folic Acid Suppl.	90%	0.76 (-0.13, 1.57)	21.05 (-4.09, 59.57)	1301 (-253, 3682)	543.16 (-88.40, 1720)	946.74 (-154.09, 2997)	54.27 (-8.83, 171.82)	94.60 (-15.40, 299)
Calcium Suppl.	50%	0.83 (0.18, 1.47)	25.97 (0.86, 65.06)	1605 (53, 4021)	667.91 (29.69, 1778)	1164.18 (51.76, 3099)	120.13 (5.34, 319.77)	209.39 (9.31, 557)
	90%	1.49 (0.32, 2.65)	46.75 (1.55, 117.12)	2889 (96, 7238)	1202.23 (53.45, 3200)	2095.53 (93.17, 5578)	120.13 (5.34, 319.77)	209.39 (9.31, 557)
Multiple Micronutrient Suppl.	50%	0.62 (0.32, 0.95)	18.80 (3.82, 39.60)	1162 (236, 2448)	471.07 (95.18, 1245)	821.09 (165.90, 2170)	84.72 (17.12, 223.90)	147.68 (29.84, 390)
	90%	1.45 (0.59, 2.37)	43.52 (8.00, 98.74)	2690 (495, 6102)	1097.99 (194.81, 2923)	1913.82 (339.55, 5094)	109.71 (19.47, 292.02)	191.23 (33.93, 509)
Balanced Protein Suppl.	50%	0.01 (-0.01, 0.03)	0.26 (-0.27, 0.97)	16 (-17, 60)	6.35 (-7.45, 29.06)	11.07 (-12.98, 50.65)	79.83 (-79.40, 301.22)	139.15 (-138.40, 525)
	90%	0.02 (-0.02, 0.05)	0.46 (-0.49, 1.75)	28 (-30, 108)	11.43 (-13.40, 52.30)	19.93 (-23.36, 91.16)	79.83 (-79.40, 301.22)	139.15 (-138.40, 525)

References for Data Inputs

[1] Blencowe H, Krasevec J, Onis M De, et al. Articles National , regional , and worldwide estimates of low birthweight in 2015 , with trends from 2000: a systematic analysis. Lancet Glob Heal. 2019;(18):1-12.
[2] Chawanpaiboon S, Vogel JP, Moller AB, et al. Global, regional, and national estimates of levels of preterm birth in 2014: a systematic review and modelling analysis. Lancet Glob Heal. 2019;7(1):e37-e46.
[3] United National Population Division World Population Prospects 2019.
[4] Fink G, Peet E, Danaei G, et al. Schooling and wage income losses due to early-childhood growth faltering in developing countries: National, regional, and global estimates. Am J Clin Nutr. 2016;104(1):104-112.
[5] Country specific annual wage data from World Indicators Database. Average yearly wage was estimated to be 2/3 of the gross domestic product in 2010 constant US dollars and 2011 International dollars, adjusted for purchasing power parity.
[6] NCD Risk Factor Collaboration. Trends in adult body-mass index in 200 countries from 1975 to 2014: a pooled analysis of 1698 population-based measurement studies with 19·2 million participants. Lancet. 2016;387(10026):1377-1396.
[7] Stevens GA, Finucane MM, De-Regil LM, et al. Global, regional, and national trends in haemoglobin concentration and prevalence of total and severe anaemia in children and pregnant and non-pregnant women for 1995-2011: A systematic analysis of population-representative data. Lancet Glob Heal. 2013;1(1):16-25.
[8] Coverage of iron-folic acid supplementation abstracted from the most recent Demographic Health Survey or imputed based on sub-regional average. Indicator used: % women in the past five years who took iron tablets or syrup for >90 days.

Micronesia

Region: Southeast Asia, East Asia, and Oceania**; Sub-region:** Oceania

Low birthweight prevalence[1]: 11.1% (95% CI: 8.4, 13.8)

Preterm birth prevalence[2]: 10.0% (95% CI: 7.9, 12.7)

Number of births[3]: 13,000

Returns to education[4]: 6.1% (95% CI: 2.7, 9.6)

GDP per capita 2010 US$ (estimated annual wage)[5]: $2723 ($1815/year)

GDP per capita 2011 International $ (estimated annual wage)[5]: $3137 ($2091/year)

Prevalence of low BMI[6]: 1.1% (95% CI: 0.4, 2.2)

Prevalence of anemia[7]: 29.8% (95% CI: 6.3, 53.3)

Baseline coverage of IFA[8]: 44.2%

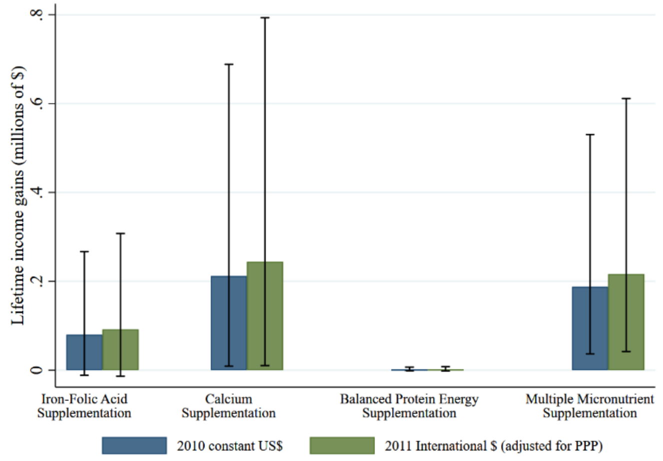

Figure: Benefits by birth cohort size for estimated gains in lifetime wages attributable to scaling up nutrition interventions to 90% target coverage

Table: Impact of maternal prenatal nutrition intervention on human capital and labour market outcomes, through improvements in low birthweight or preterm birth and schooling in Micronesia.

Intervention	Target Coverage (%)	Absolute reduction in birth outcome (%)	Benefits by cohorts: School years gained (in 1000 s)	No. of additional students completing secondary school	Benefits by cohorts: Lifetime wages		Returns in lifetime earnings per child born to a targeted pregnant woman	
					in US $ millions	in International $ millions	in US $	in International $
Iron/Folic Acid Suppl.	90%	0.86 (-0.15, 1.87)	0.03 (-0.00, 0.08)	1 (-0, 2)	0.08 (-0.01, 0.27)	0.09 (-0.01, 0.31)	6.82 (-1.00, 22.82)	7.86 (-1.15, 26.29)
Calcium Suppl.	50%	1.18 (0.27, 2.15)	0.04 (0.00, 0.11)	1 (0, 2)	0.12 (0.00, 0.38)	0.14 (0.01, 0.44)	18.11 (0.77, 58.84)	20.87 (0.89, 67.80)
	90%	2.12 (0.48, 3.87)	0.08 (0.00, 0.19)	2 (0, 4)	0.21 (0.01, 0.69)	0.24 (0.01, 0.79)	18.11 (0.77, 58.84)	20.87 (0.89, 67.80)
Multiple Micronutrient Suppl.	50%	0.71 (0.36, 1.06)	0.02 (0.01, 0.05)	1 (0, 1)	0.07 (0.01, 0.19)	0.08 (0.02, 0.22)	10.82 (2.04, 28.94)	12.47 (2.35, 33.34)
	90%	1.93 (0.85, 3.03)	0.07 (0.01, 0.14)	1 (0, 3)	0.19 (0.04, 0.53)	0.22 (0.04, 0.61)	16.04 (3.12, 45.34)	18.48 (3.59, 52.24)
Balanced Protein Suppl.	50%	0.01 (-0.01, 0.03)	0.00 (-0.00, 0.00)	0 (-0, 0)	0.00 (-0.00, 0.00)	0.00 (-0.00, 0.00)	12.13 (-11.59, 48.76)	13.98 (-13.35, 56.18)
	90%	0.01 (-0.01, 0.06)	0.00 (-0.00, 0.00)	0 (-0, 0)	0.00 (-0.00, 0.01)	0.00 (-0.00, 0.01)	12.13 (-11.59, 48.76)	13.98 (-13.35, 56.18)

References for Data Inputs

[1] Blencowe H, Krasevec J, Onis M De, et al. Articles National , regional , and worldwide estimates of low birthweight in 2015 , with trends from 2000: a systematic analysis. Lancet Glob Heal. 2019;(18):1-12.

[2] Chawanpaiboon S, Vogel JP, Moller AB, et al. Global, regional, and national estimates of levels of preterm birth in 2014: a systematic review and modelling analysis. Lancet Glob Heal. 2019;7(1):e37-e46.

[3] United National Population Division World Population Prospects 2019.

[4] Fink G, Peet E, Danaei G, et al. Schooling and wage income losses due to early-childhood growth faltering in developing countries: National, regional, and global estimates. Am J Clin Nutr. 2016;104(1):104-112.

[5] Country specific annual wage data from World Indicators Database. Average yearly wage was estimated to be 2/3 of the gross domestic product in 2010 constant US dollars and 2011 International dollars, adjusted for purchasing power parity.

[6] NCD Risk Factor Collaboration. Trends in adult body-mass index in 200 countries from 1975 to 2014: a pooled analysis of 1698 population-based measurement studies with 19·2 million participants. Lancet. 2016;387(10026):1377-1396.

[7] Stevens GA, Finucane MM, De-Regil LM, et al. Global, regional, and national trends in haemoglobin concentration and prevalence of total and severe anaemia in children and pregnant and non-pregnant women for 1995-2011: A systematic analysis of population-representative data. Lancet Glob Heal. 2013;1(1):16-25.

[8] Coverage of iron-folic acid supplementation abstracted from the most recent Demographic Health Survey or imputed based on sub-regional average. Indicator used: % women in the past five years who took iron tablets or syrup for >90 days.

Moldova

Region: Central Europe, Eastern Europe, Central Asia**; Sub-region:** Eastern Europe

Low birthweight prevalence[1]: 5.0% (95% CI: 4.0, 6.3)

Preterm birth prevalence[2]: 6.4% (95% CI: 2.7, 10.1)

Number of births[3]: 206,000

Returns to education[4]: 6.7% (95% CI: 5.3, 8.1)

GDP per capita 2010 US$ (estimated annual wage)[5]: $2356 ($1570/year)

GDP per capita 2011 International $ (estimated annual wage)[5]: $5696 ($3797/year)

Prevalence of low BMI[6]: 2.4% (95% CI: 0.9, 4.9)

Prevalence of anemia[7]: 27.8% (95% CI: 17.3, 43.4)

Baseline coverage of IFA[8]: 18.5%

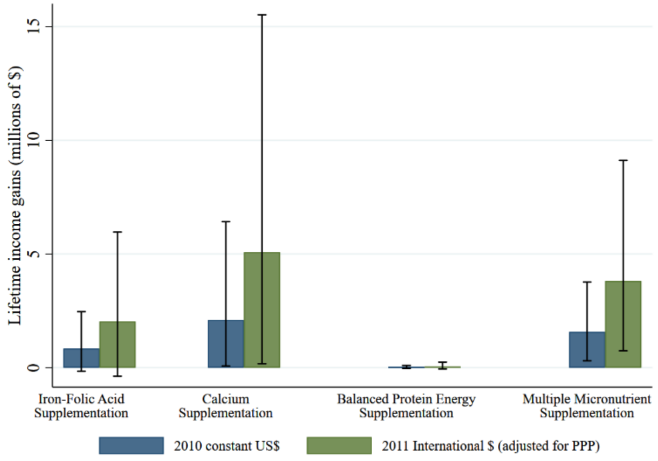

Figure: Benefits by birth cohort size for estimated gains in lifetime wages attributable to scaling up nutrition interventions to 90% target coverage

Table: Impact of maternal prenatal nutrition intervention on human capital and labour market outcomes, through improvements in low birthweight or preterm birth and schooling in Moldova.

Intervention	Target Coverage (%)	Absolute reduction in birth outcome (%)	Benefits by cohorts: School years gained (in 1000 s)	No. of additional students completing secondary school	Benefits by cohorts: Lifetime wages		Returns in lifetime earnings per child born to a targeted pregnant woman	
					in US $ millions	in International $ millions	in US $	in International $
Iron/Folic Acid Suppl.	90%	0.58 (-0.11, 1.21)	0.30 (-0.05, 0.84)	14 (-2, 38)	0.84 (-0.15, 2.47)	2.04 (-0.37, 5.97)	4.54 (-0.82, 13.32)	10.98 (-1.99, 32.20)
Calcium Suppl.	50%	0.71 (0.12, 1.55)	0.41 (0.01, 1.19)	19 (1, 54)	1.17 (0.04, 3.57)	2.82 (0.10, 8.62)	11.34 (0.40, 34.63)	27.41 (0.97, 83.73)
	90%	1.28 (0.21, 2.80)	0.74 (0.03, 2.14)	34 (1, 98)	2.10 (0.07, 6.42)	5.08 (0.18, 15.52)	11.34 (0.40, 34.63)	27.41 (0.97, 83.73)
Multiple Micronutrient Suppl.	50%	0.50 (0.21, 0.78)	0.28 (0.06, 0.63)	13 (3, 29)	0.77 (0.17, 1.82)	1.87 (0.40, 4.40)	7.51 (1.61, 17.65)	18.17 (3.90, 42.67)
	90%	1.02 (0.37, 1.64)	0.56 (0.11, 1.28)	26 (5, 58)	1.58 (0.31, 3.77)	3.81 (0.74, 9.12)	8.51 (1.66, 20.34)	20.57 (4.02, 49.17)
Balanced Protein Suppl.	50%	0.01 (-0.01, 0.03)	0.00 (-0.00, 0.02)	0 (-0, 1)	0.01 (-0.01, 0.06)	0.03 (-0.03, 0.14)	5.79 (-5.34, 18.97)	13.99 (-12.92, 45.86)
	90%	0.02 (-0.01, 0.06)	0.01 (-0.01, 0.04)	0 (-0, 2)	0.02 (-0.02, 0.10)	0.06 (-0.06, 0.25)	5.79 (-5.34, 18.97)	13.99 (-12.92, 45.86)

References for Data Inputs

[1] Blencowe H, Krasevec J, Onis M De, et al. Articles National , regional , and worldwide estimates of low birthweight in 2015 , with trends from 2000: a systematic analysis. Lancet Glob Heal. 2019;(18):1-12.
[2] Chawanpaiboon S, Vogel JP, Moller AB, et al. Global, regional, and national estimates of levels of preterm birth in 2014: a systematic review and modelling analysis. Lancet Glob Heal. 2019;7(1):e37-e46.
[3] United National Population Division World Population Prospects 2019.
[4] Fink G, Peet E, Danaei G, et al. Schooling and wage income losses due to early-childhood growth faltering in developing countries: National, regional, and global estimates. Am J Clin Nutr. 2016;104(1):104-112.
[5] Country specific annual wage data from World Indicators Database. Average yearly wage was estimated to be 2/3 of the gross domestic product in 2010 constant US dollars and 2011 International dollars, adjusted for purchasing power parity.
[6] NCD Risk Factor Collaboration. Trends in adult body-mass index in 200 countries from 1975 to 2014: a pooled analysis of 1698 population-based measurement studies with 19·2 million participants. Lancet. 2016;387(10026):1377-1396.
[7] Stevens GA, Finucane MM, De-Regil LM, et al. Global, regional, and national trends in haemoglobin concentration and prevalence of total and severe anaemia in children and pregnant and non-pregnant women for 1995-2011: A systematic analysis of population-representative data. Lancet Glob Heal. 2013;1(1):16-25.
[8] Coverage of iron-folic acid supplementation abstracted from the most recent Demographic Health Survey or imputed based on sub-regional average. Indicator used: % women in the past five years who took iron tablets or syrup for >90 days.

Mongolia

Region: Central Europe, Eastern Europe, Central Asia**; Sub-region:** Central Asia

Low birthweight prevalence[1]: 5.4% (95% CI: 4.2, 6.9)

Preterm birth prevalence[2]: 10.4% (95% CI: 8.7, 11.9)

Number of births[3]: 384,000

Returns to education[4]: 5.1% (95% CI: 2.4, 7.8)

GDP per capita 2010 US$ (estimated annual wage)[5]: $3895 ($2597/year)

GDP per capita 2011 International $ (estimated annual wage)[5]: $11330 ($7553/year)

Prevalence of low BMI[6]: 2.7% (95% CI: 1.2, 5.0)

Prevalence of anemia[7]: 24.8% (95% CI: 9.9, 48.5)

Baseline coverage of IFA[8]: 3.0%

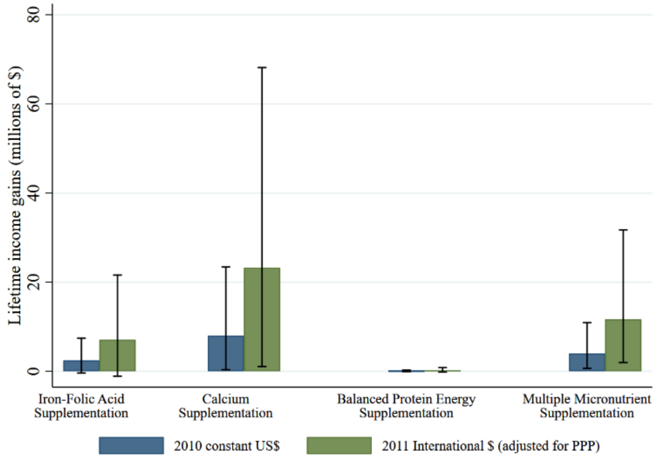

Figure: Benefits by birth cohort size for estimated gains in lifetime wages attributable to scaling up nutrition interventions to 90% target coverage

Table: Impact of maternal prenatal nutrition intervention on human capital and labour market outcomes, through improvements in low birthweight or preterm birth and schooling in Mongolia.

Intervention	Target Coverage (%)	Absolute reduction in birth outcome (%)	Benefits by cohorts: School years gained (in 1000 s)	No. of additional students completing secondary school	Benefits by cohorts: Lifetime wages		Returns in lifetime earnings per child born to a targeted pregnant woman	
					in US $ millions	in International $ millions	in US $	in International $
Iron/Folic Acid Suppl.	90%	0.74 (-0.13, 1.47)	0.72 (-0.14, 1.96)	14 (-3, 38)	2.43 (-0.38, 7.42)	7.07 (-1.09, 21.58)	7.04 (-1.09, 21.47)	20.47 (-3.17, 62.43)
Calcium Suppl.	50%	1.25 (0.29, 2.10)	1.34 (0.04, 3.26)	26 (1, 63)	4.44 (0.20, 13.01)	12.92 (0.59, 37.85)	23.14 (1.06, 67.78)	67.30 (3.09, 197.15)
	90%	2.26 (0.51, 3.78)	2.42 (0.08, 5.87)	47 (1, 114)	8.00 (0.37, 23.43)	23.26 (1.07, 68.14)	23.14 (1.06, 67.78)	67.30 (3.09, 197.15)
Multiple Micronutrient Suppl.	50%	0.65 (0.22, 1.07)	0.65 (0.12, 1.51)	13 (2, 29)	2.19 (0.38, 5.94)	6.38 (1.10, 17.28)	11.42 (1.97, 30.94)	33.22 (5.73, 90.00)
	90%	1.19 (0.39, 1.97)	1.19 (0.21, 2.78)	23 (4, 54)	4.01 (0.67, 10.91)	11.65 (1.96, 31.74)	11.59 (1.95, 31.57)	33.72 (5.67, 91.83)
Balanced Protein Suppl.	50%	0.01 (-0.01, 0.03)	0.01 (-0.01, 0.04)	0 (-0, 1)	0.03 (-0.04, 0.15)	0.10 (-0.11, 0.45)	7.10 (-6.64, 26.34)	20.64 (-19.30, 76.61)
	90%	0.02 (-0.02, 0.06)	0.02 (-0.02, 0.08)	0 (-0, 1)	0.06 (-0.07, 0.28)	0.17 (-0.19, 0.81)	7.10 (-6.64, 26.34)	20.64 (-19.30, 76.61)

References for Data Inputs

[1] Blencowe H, Krasevec J, Onis M De, et al. Articles National , regional , and worldwide estimates of low birthweight in 2015 , with trends from 2000: a systematic analysis. Lancet Glob Heal. 2019;(18):1-12.

[2] Chawanpaiboon S, Vogel JP, Moller AB, et al. Global, regional, and national estimates of levels of preterm birth in 2014: a systematic review and modelling analysis. Lancet Glob Heal. 2019;7(1):e37-e46.

[3] United National Population Division World Population Prospects 2019.

[4] Fink G, Peet E, Danaei G, et al. Schooling and wage income losses due to early-childhood growth faltering in developing countries: National, regional, and global estimates. Am J Clin Nutr. 2016;104(1):104-112.

[5] Country specific annual wage data from World Indicators Database. Average yearly wage was estimated to be 2/3 of the gross domestic product in 2010 constant US dollars and 2011 International dollars, adjusted for purchasing power parity.

[6] NCD Risk Factor Collaboration. Trends in adult body-mass index in 200 countries from 1975 to 2014: a pooled analysis of 1698 population-based measurement studies with 19·2 million participants. Lancet. 2016;387(10026):1377-1396.

[7] Stevens GA, Finucane MM, De-Regil LM, et al. Global, regional, and national trends in haemoglobin concentration and prevalence of total and severe anaemia in children and pregnant and non-pregnant women for 1995-2011: A systematic analysis of population-representative data. Lancet Glob Heal. 2013;1(1):16-25.

[8] Coverage of iron-folic acid supplementation abstracted from the most recent Demographic Health Survey or imputed based on sub-regional average. Indicator used: % women in the past five years who took iron tablets or syrup for >90 days.

Montenegro

Region: Central Europe, Eastern Europe, Central Asia**; Sub-region:** Central Europe

Low birthweight prevalence[1]: 5.5% (95% CI: 5.2, 5.7)

Preterm birth prevalence[2]: 8.7% (95% CI: 6.3, 13.3)

Number of births[3]: 37,000

Returns to education[4]: 6.7% (95% CI: 5.3, 8.1)

GDP per capita 2010 US$ (estimated annual wage)[5]: $7280 ($4853/year)

GDP per capita 2011 International $ (estimated annual wage)[5]: $15290 ($10193/year)

Prevalence of low BMI[6]: 2.2% (95% CI: 0.7, 5.2)

Prevalence of anemia[7]: 27.2% (95% CI: 8.4, 46.0)

Baseline coverage of IFA[8]: 18.5%

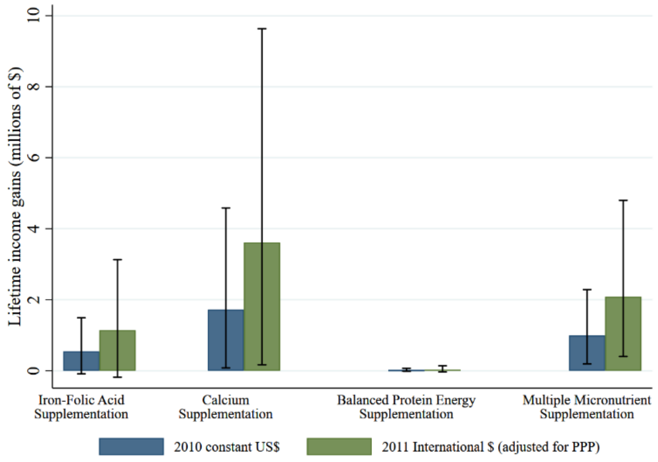

Figure: Benefits by birth cohort size for estimated gains in lifetime wages attributable to scaling up nutrition interventions to 90% target coverage

Table: Impact of maternal prenatal nutrition intervention on human capital and labour market outcomes, through improvements in low birthweight or preterm birth and schooling in Montenegro.

Intervention	Target Coverage (%)	Absolute reduction in birth outcome (%)	Benefits by cohorts: School years gained (in 1000 s)	No. of additional students completing secondary school	Benefits by cohorts: Lifetime wages		Returns in lifetime earnings per child born to a targeted pregnant woman	
					in US $ millions	in International $ millions	in US $	in International $
Iron/Folic Acid Suppl.	90%	0.65 (-0.12, 1.28)	0.06 (-0.01, 0.17)	3 (-1, 7)	0.54 (-0.09, 1.49)	1.14 (-0.18, 3.13)	16.33 (-2.59, 44.73)	34.30 (-5.43, 93.95)
Calcium Suppl.	50%	1.02 (0.22, 1.91)	0.11 (0.00, 0.29)	5 (0, 13)	0.96 (0.04, 2.55)	2.01 (0.09, 5.35)	51.66 (2.42, 137.73)	108.50 (5.09, 289.26)
	90%	1.83 (0.39, 3.44)	0.20 (0.01, 0.52)	9 (0, 23)	1.72 (0.08, 4.59)	3.61 (0.17, 9.63)	51.66 (2.42, 137.73)	108.50 (5.09, 289.26)
Multiple Micronutrient Suppl.	50%	0.57 (0.25, 0.83)	0.06 (0.01, 0.12)	2 (1, 5)	0.49 (0.11, 1.12)	1.04 (0.22, 2.35)	26.75 (5.72, 60.61)	56.18 (12.02, 127.29)
	90%	1.15 (0.45, 1.75)	0.11 (0.02, 0.25)	5 (1, 11)	0.99 (0.19, 2.29)	2.08 (0.40, 4.80)	29.79 (5.72, 68.65)	62.58 (12.02, 144.18)
Balanced Protein Suppl.	50%	0.01 (-0.01, 0.03)	0.00 (-0.00, 0.00)	0 (-0, 0)	0.01 (-0.01, 0.04)	0.01 (-0.02, 0.08)	20.48 (-20.10, 65.54)	43.00 (-42.21, 137.64)
	90%	0.02 (-0.02, 0.06)	0.00 (-0.00, 0.01)	0 (-0, 0)	0.01 (-0.02, 0.07)	0.03 (-0.03, 0.14)	20.48 (-20.10, 65.54)	43.00 (-42.21, 137.64)

References for Data Inputs

[1] Blencowe H, Krasevec J, Onis M De, et al. Articles National , regional , and worldwide estimates of low birthweight in 2015 , with trends from 2000: a systematic analysis. Lancet Glob Heal. 2019;(18):1-12.

[2] Chawanpaiboon S, Vogel JP, Moller AB, et al. Global, regional, and national estimates of levels of preterm birth in 2014: a systematic review and modelling analysis. Lancet Glob Heal. 2019;7(1):e37-e46.

[3] United National Population Division World Population Prospects 2019.

[4] Fink G, Peet E, Danaei G, et al. Schooling and wage income losses due to early-childhood growth faltering in developing countries: National, regional, and global estimates. Am J Clin Nutr. 2016;104(1):104-112.

[5] Country specific annual wage data from World Indicators Database. Average yearly wage was estimated to be 2/3 of the gross domestic product in 2010 constant US dollars and 2011 International dollars, adjusted for purchasing power parity.

[6] NCD Risk Factor Collaboration. Trends in adult body-mass index in 200 countries from 1975 to 2014: a pooled analysis of 1698 population-based measurement studies with 19·2 million participants. Lancet. 2016;387(10026):1377-1396.

[7] Stevens GA, Finucane MM, De-Regil LM, et al. Global, regional, and national trends in haemoglobin concentration and prevalence of total and severe anaemia in children and pregnant and non-pregnant women for 1995-2011: A systematic analysis of population-representative data. Lancet Glob Heal. 2013;1(1):16-25.

[8] Coverage of iron-folic acid supplementation abstracted from the most recent Demographic Health Survey or imputed based on sub-regional average. Indicator used: % women in the past five years who took iron tablets or syrup for >90 days.

Morocco

Region: North Africa and Middle East**; Sub-region:** North Africa and Middle East

Low birthweight prevalence[1]: 17.3% (95% CI: 13.9, 21.9)

Preterm birth prevalence[2]: 13.4% (95% CI: 6.3, 30.9)

Number of births[3]: 3,409,000

Returns to education[4]: 15.8% (95% CI: 14.8, 16.8)

GDP per capita 2010 US$ (estimated annual wage)[5]: $3222 ($2148/year)

GDP per capita 2011 International $ (estimated annual wage)[5]: $7325 ($4883/year)

Prevalence of low BMI[6]: 3.1% (95% CI: 1.2, 6.2)

Prevalence of anemia[7]: 32.6% (95% CI: 18.1, 52.0)

Baseline coverage of IFA[8]: 24.3%

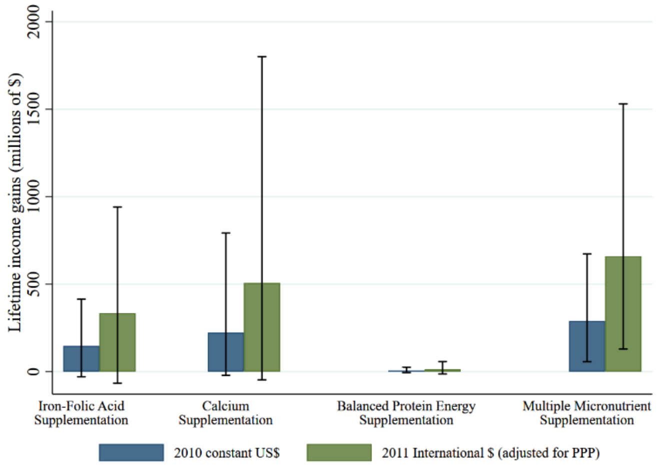

Figure: Benefits by birth cohort size for estimated gains in lifetime wages attributable to scaling up nutrition interventions to 90% target coverage

Table: Impact of maternal prenatal nutrition intervention on human capital and labour market outcomes, through improvements in low birthweight or preterm birth and schooling in Morocco.

Intervention	Target Coverage (%)	Absolute reduction in birth outcome (%)	Benefits by cohorts: School years gained (in 1000 s)	No. of additional students completing secondary school	Benefits by cohorts: Lifetime wages		Returns in lifetime earnings per child born to a targeted pregnant woman	
					in US $ millions	in International $ millions	in US $	in International $
Iron/Folic Acid Suppl.	90%	1.87 (-0.36, 3.93)	16.03 (-3.13, 45.61)	353 (-69, 1004)	147.28 (-28.95, 413.80)	334.82 (-65.81, 940.73)	48.00 (-9.43, 134.87)	109.13 (-21.45, 306.62)
Calcium Suppl.	50%	1.43 (-0.09, 3.73)	13.45 (-1.24, 48.74)	296 (-27, 1072)	123.87 (-11.44, 439.93)	281.60 (-26.01, 1000.14)	72.67 (-6.71, 258.10)	165.21 (-15.26, 586.76)
	90%	2.57 (-0.17, 6.71)	24.22 (-2.23, 87.73)	533 (-49, 1930)	222.96 (-20.59, 791.88)	506.88 (-46.81, 1800.24)	72.67 (-6.71, 258.10)	165.21 (-15.26, 586.76)
Multiple Micronutrient Suppl.	50%	1.65 (0.78, 2.52)	15.09 (3.17, 33.63)	332 (70, 740)	138.03 (28.80, 305.77)	313.80 (65.47, 695.13)	80.98 (16.89, 179.39)	184.10 (38.41, 407.82)
	90%	3.50 (1.44, 5.69)	31.95 (6.41, 72.58)	703 (141, 1597)	290.21 (57.06, 673.05)	659.75 (129.73, 1530.11)	94.59 (18.60, 219.37)	215.04 (42.28, 498.71)
Balanced Protein Suppl.	50%	0.04 (-0.03, 0.13)	0.35 (-0.35, 1.53)	8 (-8, 34)	3.16 (-3.13, 14.07)	7.19 (-7.11, 31.99)	66.02 (-62.57, 209.47)	150.10 (-142.25, 476.20)
	90%	0.08 (-0.06, 0.24)	0.62 (-0.62, 2.76)	14 (-14, 61)	5.69 (-5.63, 25.33)	12.94 (-12.80, 57.58)	66.02 (-62.57, 209.47)	150.10 (-142.25, 476.20)

References for Data Inputs

[1] Blencowe H, Krasevec J, Onis M De, et al. Articles National , regional , and worldwide estimates of low birthweight in 2015 , with trends from 2000: a systematic analysis. Lancet Glob Heal. 2019;(18):1-12.
[2] Chawanpaiboon S, Vogel JP, Moller AB, et al. Global, regional, and national estimates of levels of preterm birth in 2014: a systematic review and modelling analysis. Lancet Glob Heal. 2019;7(1):e37-e46.
[3] United National Population Division World Population Prospects 2019.
[4] Fink G, Peet E, Danaei G, et al. Schooling and wage income losses due to early-childhood growth faltering in developing countries: National, regional, and global estimates. Am J Clin Nutr. 2016;104(1):104-112.
[5] Country specific annual wage data from World Indicators Database. Average yearly wage was estimated to be 2/3 of the gross domestic product in 2010 constant US dollars and 2011 International dollars, adjusted for purchasing power parity.
[6] NCD Risk Factor Collaboration. Trends in adult body-mass index in 200 countries from 1975 to 2014: a pooled analysis of 1698 population-based measurement studies with 19·2 million participants. Lancet. 2016;387(10026):1377-1396.
[7] Stevens GA, Finucane MM, De-Regil LM, et al. Global, regional, and national trends in haemoglobin concentration and prevalence of total and severe anaemia in children and pregnant and non-pregnant women for 1995-2011: A systematic analysis of population-representative data. Lancet Glob Heal. 2013;1(1):16-25.
[8] Coverage of iron-folic acid supplementation abstracted from the most recent Demographic Health Survey or imputed based on sub-regional average. Indicator used: % women in the past five years who took iron tablets or syrup for >90 days.

Mozambique

Region: Sub-Saharan Africa**; Sub-region:** Eastern Sub-Saharan Africa

Low birthweight prevalence[1]: 13.8% (95% CI: 10.9, 17.5)

Preterm birth prevalence[2]: 12.0% (95% CI: 8.6, 16.7)

Number of births[3]: 5,495,000

Returns to education[4]: 11.3% (95% CI: 9.7, 12.9)

GDP per capita 2010 US$ (estimated annual wage)[5]: $529 ($353/year)

GDP per capita 2011 International $ (estimated annual wage)[5]: $1158 ($772/year)

Prevalence of low BMI[6]: 9.8% (95% CI: 5.1, 15.7)

Prevalence of anemia[7]: 48.5% (95% CI: 38.8, 57.9)

Baseline coverage of IFA[8]: 25.9%

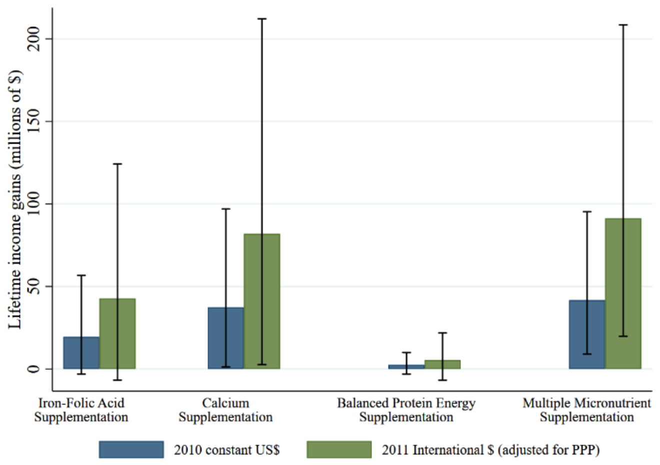

Figure: Benefits by birth cohort size for estimated gains in lifetime wages attributable to scaling up nutrition interventions to 90% target coverage

Table: Impact of maternal prenatal nutrition intervention on human capital and labour market outcomes, through improvements in low birthweight or preterm birth and schooling in Mozambique.

Intervention	Target Coverage (%)	Absolute reduction in birth outcome (%)	Benefits by cohorts: School years gained (in 1000 s)	No. of additional students completing secondary school	Benefits by cohorts: Lifetime wages		Returns in lifetime earnings per child born to a targeted pregnant woman	
					in US $ millions	in International $ millions	in US $	in International $
Iron/Folic Acid Suppl.	90%	1.45 (-0.27, 3.15)	18.48 (-2.95, 52.99)	137 (-22, 392)	19.49 (-3.06, 56.72)	42.67 (-6.70, 124.17)	3.94 (-0.62, 11.47)	8.63 (-1.35, 25.11)
Calcium Suppl.	50%	1.41 (0.30, 2.55)	19.42 (0.69, 49.59)	144 (5, 367)	20.79 (0.68, 53.86)	45.50 (1.49, 117.90)	7.57 (0.25, 19.60)	16.56 (0.54, 42.91)
	90%	2.53 (0.53, 4.58)	34.96 (1.24, 89.26)	259 (9, 661)	37.42 (1.23, 96.94)	81.91 (2.69, 212.22)	7.57 (0.25, 19.60)	16.56 (0.54, 42.91)
Multiple Micronutrient Suppl.	50%	1.37 (0.74, 2.09)	18.30 (4.10, 39.96)	135 (30, 296)	19.59 (4.26, 43.58)	42.88 (9.32, 95.41)	7.13 (1.55, 15.86)	15.61 (3.39, 34.73)
	90%	2.93 (1.38, 4.69)	38.39 (7.95, 88.11)	284 (59, 652)	41.70 (9.04, 95.26)	91.28 (19.80, 208.54)	8.43 (1.83, 19.26)	18.46 (4.00, 42.17)
Balanced Protein Suppl.	50%	0.11 (-0.10, 0.31)	1.27 (-1.64, 5.10)	9 (-12, 38)	1.37 (-1.72, 5.54)	3.00 (-3.76, 12.14)	5.35 (-5.22, 17.58)	11.72 (-11.43, 38.49)
	90%	0.19 (-0.18, 0.55)	2.28 (-2.94, 9.17)	17 (-22, 68)	2.47 (-3.09, 9.98)	5.40 (-6.76, 21.85)	5.35 (-5.22, 17.58)	11.72 (-11.43, 38.49)

References for Data Inputs

[1] Blencowe H, Krasevec J, Onis M De, et al. Articles National , regional , and worldwide estimates of low birthweight in 2015 , with trends from 2000: a systematic analysis. Lancet Glob Heal. 2019;(18):1-12.
[2] Chawanpaiboon S, Vogel JP, Moller AB, et al. Global, regional, and national estimates of levels of preterm birth in 2014: a systematic review and modelling analysis. Lancet Glob Heal. 2019;7(1):e37-e46.
[3] United National Population Division World Population Prospects 2019.
[4] Fink G, Peet E, Danaei G, et al. Schooling and wage income losses due to early-childhood growth faltering in developing countries: National, regional, and global estimates. Am J Clin Nutr. 2016;104(1):104-112.
[5] Country specific annual wage data from World Indicators Database. Average yearly wage was estimated to be 2/3 of the gross domestic product in 2010 constant US dollars and 2011 International dollars, adjusted for purchasing power parity.
[6] NCD Risk Factor Collaboration. Trends in adult body-mass index in 200 countries from 1975 to 2014: a pooled analysis of 1698 population-based measurement studies with 19·2 million participants. Lancet. 2016;387(10026):1377-1396.
[7] Stevens GA, Finucane MM, De-Regil LM, et al. Global, regional, and national trends in haemoglobin concentration and prevalence of total and severe anaemia in children and pregnant and non-pregnant women for 1995-2011: A systematic analysis of population-representative data. Lancet Glob Heal. 2013;1(1):16-25.
[8] Coverage of iron-folic acid supplementation abstracted from the most recent Demographic Health Survey or imputed based on sub-regional average. Indicator used: % women in the past five years who took iron tablets or syrup for >90 days.

Myanmar

Region: Southeast Asia, East Asia, and Oceania**; Sub-region:** Southeast Asia

Low birthweight prevalence[1]: 12.3% (95% CI: 8.5, 15.9)

Preterm birth prevalence[2]: 10.4% (95% CI: 8.7, 11.9)

Number of births[3]: 4,738,000

Returns to education[4]: 6.5% (95% CI: 2.1, 10.9)

GDP per capita 2010 US$ (estimated annual wage)[5]: $1335 ($890/year)

GDP per capita 2011 International $ (estimated annual wage)[5]: $5030 ($3354/year)

Prevalence of low BMI[6]: 14.4% (95% CI: 8.8, 21.1)

Prevalence of anemia[7]: 34.0% (95% CI: 20.0, 54.7)

Baseline coverage of IFA[8]: 59.3%

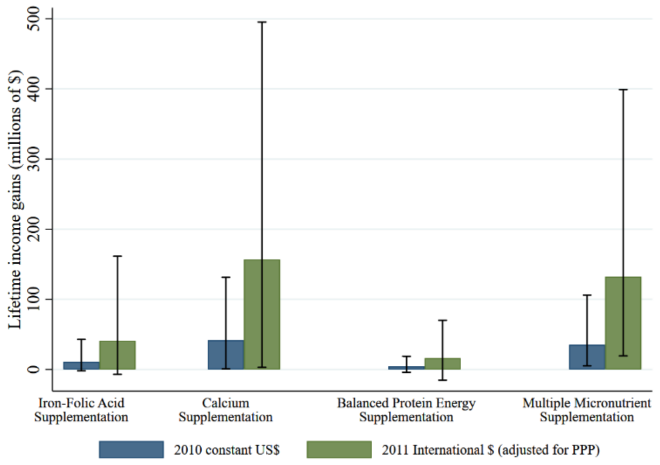

Figure: Benefits by birth cohort size for estimated gains in lifetime wages attributable to scaling up nutrition interventions to 90% target coverage

Table: Impact of maternal prenatal nutrition intervention on human capital and labour market outcomes, through improvements in low birthweight or preterm birth and schooling in Myanmar.

Intervention	Target Coverage (%)	Absolute reduction in birth outcome (%)	Benefits by cohorts: School years gained (in 1000 s)	No. of additional students completing secondary school	Benefits by cohorts: Lifetime wages		Returns in lifetime earnings per child born to a targeted pregnant woman	
					in US $ millions	in International $ millions	in US $	in International $
Iron/Folic Acid Suppl.	90%	0.67 (-0.10, 1.55)	7.34 (-1.17, 21.32)	50 (-8, 145)	10.88 (-1.86, 42.85)	40.97 (-7.00, 161.43)	2.55 (-0.44, 10.05)	9.61 (-1.64, 37.86)
Calcium Suppl.	50%	1.25 (0.26, 2.11)	15.66 (0.49, 38.33)	106 (3, 261)	23.13 (0.44, 73.04)	87.15 (1.67, 275.16)	9.76 (0.19, 30.83)	36.79 (0.71, 116.15)
	90%	2.24 (0.47, 3.79)	28.19 (0.88, 68.99)	192 (6, 469)	41.64 (0.80, 131.47)	156.87 (3.01, 495.29)	9.76 (0.19, 30.83)	36.79 (0.71, 116.15)
Multiple Micronutrient Suppl.	50%	0.76 (0.37, 1.19)	9.14 (1.85, 21.43)	62 (13, 146)	13.91 (1.88, 43.36)	52.39 (7.10, 163.34)	5.87 (0.80, 18.30)	22.11 (3.00, 68.95)
	90%	1.91 (0.92, 3.05)	23.33 (4.96, 52.07)	159 (34, 354)	35.14 (5.14, 105.94)	132.37 (19.37, 399.13)	8.24 (1.21, 24.84)	31.04 (4.54, 93.60)
Balanced Protein Suppl.	50%	0.15 (-0.11, 0.40)	1.62 (-1.46, 5.49)	11 (-10, 37)	2.38 (-2.25, 10.34)	8.97 (-8.49, 38.95)	7.21 (-6.41, 27.23)	27.16 (-24.15, 102.57)
	90%	0.27 (-0.20, 0.72)	2.92 (-2.63, 9.89)	20 (-18, 67)	4.28 (-4.06, 18.61)	16.14 (-15.28, 70.10)	7.21 (-6.41, 27.23)	27.16 (-24.15, 102.57)

References for Data Inputs

[1] Blencowe H, Krasevec J, Onis M De, et al. Articles National , regional , and worldwide estimates of low birthweight in 2015 , with trends from 2000: a systematic analysis. Lancet Glob Heal. 2019;(18):1-12.
[2] Chawanpaiboon S, Vogel JP, Moller AB, et al. Global, regional, and national estimates of levels of preterm birth in 2014: a systematic review and modelling analysis. Lancet Glob Heal. 2019;7(1):e37-e46.
[3] United National Population Division World Population Prospects 2019.
[4] Fink G, Peet E, Danaei G, et al. Schooling and wage income losses due to early-childhood growth faltering in developing countries: National, regional, and global estimates. Am J Clin Nutr. 2016;104(1):104-112.
[5] Country specific annual wage data from World Indicators Database. Average yearly wage was estimated to be 2/3 of the gross domestic product in 2010 constant US dollars and 2011 International dollars, adjusted for purchasing power parity.
[6] NCD Risk Factor Collaboration. Trends in adult body-mass index in 200 countries from 1975 to 2014: a pooled analysis of 1698 population-based measurement studies with 19·2 million participants. Lancet. 2016;387(10026):1377-1396.
[7] Stevens GA, Finucane MM, De-Regil LM, et al. Global, regional, and national trends in haemoglobin concentration and prevalence of total and severe anaemia in children and pregnant and non-pregnant women for 1995-2011: A systematic analysis of population-representative data. Lancet Glob Heal. 2013;1(1):16-25.
[8] Coverage of iron-folic acid supplementation abstracted from the most recent Demographic Health Survey or imputed based on sub-regional average. Indicator used: % women in the past five years who took iron tablets or syrup for >90 days.

Namibia

Region: Sub-Saharan Africa**; Sub-region:** Southern Sub-Saharan Africa

Low birthweight prevalence[1]: 15.5% (95% CI: 12.3, 19.7)

Preterm birth prevalence[2]: 12.0% (95% CI: 8.6, 16.7)

Number of births[3]: 350,000

Returns to education[4]: 16.1% (95% CI: 15.3, 16.8)

GDP per capita 2010 US$ (estimated annual wage)[5]: $6412 ($4275/year)

GDP per capita 2011 International $ (estimated annual wage)[5]: $10450 ($6967/year)

Prevalence of low BMI[6]: 8.3% (95% CI: 4.3, 13.7)

Prevalence of anemia[7]: 34.0% (95% CI: 16.0, 58.9)

Baseline coverage of IFA[8]: 38.5%

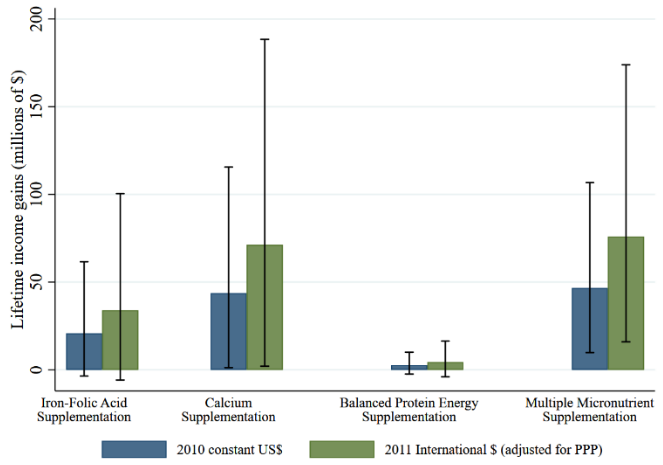

Figure: Benefits by birth cohort size for estimated gains in lifetime wages attributable to scaling up nutrition interventions to 90% target coverage

Table: Impact of maternal prenatal nutrition intervention on human capital and labour market outcomes, through improvements in low birthweight or preterm birth and schooling in Namibia.

Intervention	Target Coverage (%)	Absolute reduction in birth outcome (%)	Benefits by cohorts: School years gained (in 1000 s)	No. of additional students completing secondary school	Benefits by cohorts: Lifetime wages		Returns in lifetime earnings per child born to a targeted pregnant woman	
					in US $ millions	in International $ millions	in US $	in International $
Iron/Folic Acid Suppl.	90%	1.36 (-0.25, 2.91)	1.12 (-0.18, 3.33)	51 (-8, 152)	20.84 (-3.56, 61.63)	33.97 (-5.80, 100.44)	66.17 (-11.30, 195.65)	107.84 (-18.41, 318.85)
Calcium Suppl.	50%	1.42 (0.28, 2.59)	1.31 (0.04, 3.48)	60 (2, 159)	24.30 (0.72, 64.21)	39.60 (1.17, 104.64)	138.84 (4.11, 366.91)	226.28 (6.69, 597.97)
	90%	2.56 (0.51, 4.66)	2.37 (0.07, 6.27)	108 (3, 287)	43.74 (1.29, 115.58)	71.28 (2.11, 188.36)	138.84 (4.11, 366.91)	226.28 (6.69, 597.97)
Multiple Micronutrient Suppl.	50%	1.15 (0.65, 1.74)	1.02 (0.23, 2.22)	47 (11, 102)	18.90 (4.30, 41.63)	30.81 (7.01, 67.85)	108.02 (24.57, 237.90)	176.05 (40.05, 387.72)
	90%	2.89 (1.29, 4.67)	2.51 (0.52, 5.76)	115 (24, 264)	46.63 (9.80, 106.68)	75.99 (15.98, 173.87)	148.02 (31.13, 338.68)	241.23 (50.73, 551.95)
Balanced Protein Suppl.	50%	0.10 (-0.07, 0.29)	0.08 (-0.07, 0.31)	4 (-3, 14)	1.51 (-1.34, 5.59)	2.46 (-2.19, 9.11)	109.65 (-107.05, 352.28)	178.70 (-174.46, 574.11)
	90%	0.17 (-0.13, 0.52)	0.15 (-0.13, 0.55)	7 (-6, 25)	2.71 (-2.42, 10.06)	4.42 (-3.94, 16.39)	109.65 (-107.05, 352.28)	178.70 (-174.46, 574.11)

References for Data Inputs

[1] Blencowe H, Krasevec J, Onis M De, et al. Articles National , regional , and worldwide estimates of low birthweight in 2015 , with trends from 2000: a systematic analysis. Lancet Glob Heal. 2019;(18):1-12.

[2] Chawanpaiboon S, Vogel JP, Moller AB, et al. Global, regional, and national estimates of levels of preterm birth in 2014: a systematic review and modelling analysis. Lancet Glob Heal. 2019;7(1):e37-e46.

[3] United National Population Division World Population Prospects 2019.

[4] Fink G, Peet E, Danaei G, et al. Schooling and wage income losses due to early-childhood growth faltering in developing countries: National, regional, and global estimates. Am J Clin Nutr. 2016;104(1):104-112.

[5] Country specific annual wage data from World Indicators Database. Average yearly wage was estimated to be 2/3 of the gross domestic product in 2010 constant US dollars and 2011 International dollars, adjusted for purchasing power parity.

[6] NCD Risk Factor Collaboration. Trends in adult body-mass index in 200 countries from 1975 to 2014: a pooled analysis of 1698 population-based measurement studies with 19·2 million participants. Lancet. 2016;387(10026):1377-1396.

[7] Stevens GA, Finucane MM, De-Regil LM, et al. Global, regional, and national trends in haemoglobin concentration and prevalence of total and severe anaemia in children and pregnant and non-pregnant women for 1995-2011: A systematic analysis of population-representative data. Lancet Glob Heal. 2013;1(1):16-25.

[8] Coverage of iron-folic acid supplementation abstracted from the most recent Demographic Health Survey or imputed based on sub-regional average. Indicator used: % women in the past five years who took iron tablets or syrup for >90 days.

Nepal

Region: South Asia**; Sub-region:** South Asia

Low birthweight prevalence[1]: 21.8% (95% CI: 15.2, 30.3)

Preterm birth prevalence[2]: 5.3% (95% CI: 3.6, 7.4)

Number of births[3]: 2,810,000

Returns to education[4]: 7.8% (95% CI: 7.0, 8.6)

GDP per capita 2010 US$ (estimated annual wage)[5]: $732 ($488/year)

GDP per capita 2011 International $ (estimated annual wage)[5]: $2455 ($1637/year)

Prevalence of low BMI[6]: 17.6% (95% CI: 12.0, 24.0)

Prevalence of anemia[7]: 45.0% (95% CI: 35.1, 55.3)

Baseline coverage of IFA[8]: 70.9%

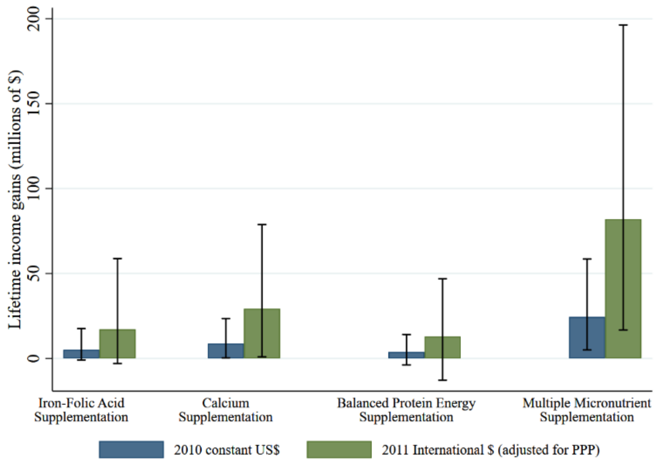

Figure: Benefits by birth cohort size for estimated gains in lifetime wages attributable to scaling up nutrition interventions to 90% target coverage

Table: Impact of maternal prenatal nutrition intervention on human capital and labour market outcomes, through improvements in low birthweight or preterm birth and schooling in Nepal.

Intervention	Target Coverage (%)	Absolute reduction in birth outcome (%)	Benefits by cohorts: School years gained (in 1000 s)	No. of additional students completing secondary school	Benefits by cohorts: Lifetime wages		Returns in lifetime earnings per child born to a targeted pregnant woman	
					in US $ millions	in International $ millions	in US $	in International $
Iron/Folic Acid Suppl.	90%	0.73 (-0.13, 1.78)	4.95 (-0.82, 16.46)	211 (-35, 701)	5.11 (-0.89, 17.54)	17.13 (-2.99, 58.82)	2.02 (-0.35, 6.94)	6.77 (-1.18, 23.26)
Calcium Suppl.	50%	0.62 (0.14, 1.18)	4.73 (0.15, 12.63)	201 (6, 538)	4.85 (0.16, 13.06)	16.28 (0.53, 43.80)	3.45 (0.11, 9.29)	11.59 (0.38, 31.17)
	90%	1.11 (0.25, 2.12)	8.51 (0.27, 22.73)	363 (11, 968)	8.74 (0.29, 23.51)	29.30 (0.96, 78.83)	3.45 (0.11, 9.29)	11.59 (0.38, 31.17)
Multiple Micronutrient Suppl.	50%	1.43 (0.70, 2.27)	10.81 (2.03, 26.69)	461 (87, 1137)	11.11 (1.98, 27.21)	37.25 (6.64, 91.26)	7.91 (1.41, 19.37)	26.52 (4.73, 64.95)
	90%	3.19 (1.70, 5.07)	23.70 (4.97, 55.73)	1010 (212, 2374)	24.45 (4.98, 58.54)	81.99 (16.71, 196.33)	9.67 (1.97, 23.15)	32.42 (6.61, 77.63)
Balanced Protein Suppl.	50%	0.32 (-0.28, 0.81)	2.10 (-2.14, 7.49)	90 (-91, 319)	2.15 (-2.12, 7.77)	7.20 (-7.10, 26.07)	8.91 (-9.25, 29.60)	29.89 (-31.01, 99.27)
	90%	0.57 (-0.50, 1.45)	3.78 (-3.86, 13.47)	161 (-164, 574)	3.86 (-3.81, 13.99)	12.95 (-12.79, 46.93)	8.91 (-9.25, 29.60)	29.89 (-31.01, 99.27)

References for Data Inputs

[1] Blencowe H, Krasevec J, Onis M De, et al. Articles National , regional , and worldwide estimates of low birthweight in 2015 , with trends from 2000: a systematic analysis. Lancet Glob Heal. 2019;(18):1-12.
[2] Chawanpaiboon S, Vogel JP, Moller AB, et al. Global, regional, and national estimates of levels of preterm birth in 2014: a systematic review and modelling analysis. Lancet Glob Heal. 2019;7(1):e37-e46.
[3] United National Population Division World Population Prospects 2019.
[4] Fink G, Peet E, Danaei G, et al. Schooling and wage income losses due to early-childhood growth faltering in developing countries: National, regional, and global estimates. Am J Clin Nutr. 2016;104(1):104-112.
[5] Country specific annual wage data from World Indicators Database. Average yearly wage was estimated to be 2/3 of the gross domestic product in 2010 constant US dollars and 2011 International dollars, adjusted for purchasing power parity.
[6] NCD Risk Factor Collaboration. Trends in adult body-mass index in 200 countries from 1975 to 2014: a pooled analysis of 1698 population-based measurement studies with 19·2 million participants. Lancet. 2016;387(10026):1377-1396.
[7] Stevens GA, Finucane MM, De-Regil LM, et al. Global, regional, and national trends in haemoglobin concentration and prevalence of total and severe anaemia in children and pregnant and non-pregnant women for 1995-2011: A systematic analysis of population-representative data. Lancet Glob Heal. 2013;1(1):16-25.
[8] Coverage of iron-folic acid supplementation abstracted from the most recent Demographic Health Survey or imputed based on sub-regional average. Indicator used: % women in the past five years who took iron tablets or syrup for >90 days.

Nicaragua

Region: Latin America and Caribbean**; Sub-region:** Central Latin America

Low birthweight prevalence[1]: 10.7% (95% CI: 8.4, 13.2)

Preterm birth prevalence[2]: 9.8% (95% CI: 8.6, 11.3)

Number of births[3]: 670,000

Returns to education[4]: 7.1% (95% CI: 6.5, 7.7)

GDP per capita 2010 US$ (estimated annual wage)[5]: $1836 ($1224/year)

GDP per capita 2011 International $ (estimated annual wage)[5]: $4846 ($3230/year)

Prevalence of low BMI[6]: 2.2% (95% CI: 0.9, 4.3)

Prevalence of anemia[7]: 20.5% (95% CI: 6.6, 41.7)

Baseline coverage of IFA[8]: 33.3%

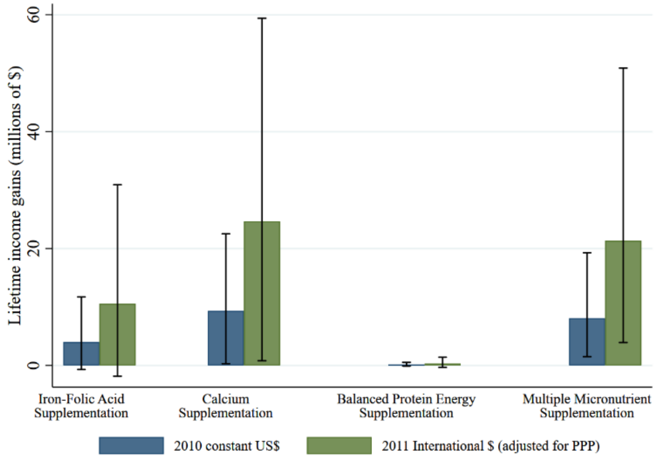

Figure: Benefits by birth cohort size for estimated gains in lifetime wages attributable to scaling up nutrition interventions to 90% target coverage

Table: Impact of maternal prenatal nutrition intervention on human capital and labour market outcomes, through improvements in low birthweight or preterm birth and schooling in Nicaragua.

Intervention	Target Coverage (%)	Absolute reduction in birth outcome (%)	Benefits by cohorts: School years gained (in 1000 s)	No. of additional students completing secondary school	Benefits by cohorts: Lifetime wages		Returns in lifetime earnings per child born to a targeted pregnant woman	
					in US $ millions	in International $ millions	in US $	in International $
**Iron/Folic Acid Suppl.**	90%	1.01 (-0.18, 2.09)	1.68 (-0.28, 4.93)	74 (-12, 216)	4.00 (-0.70, 11.71)	10.55 (-1.84, 30.90)	6.63 (-1.16, 19.42)	17.50 (-3.05, 51.24)
**Calcium Suppl.**	50%	1.18 (0.26, 1.98)	2.19 (0.07, 5.29)	96 (3, 232)	5.19 (0.17, 12.50)	13.70 (0.45, 33.00)	15.49 (0.51, 37.32)	40.88 (1.34, 98.49)
	90%	2.12 (0.47, 3.56)	3.94 (0.13, 9.52)	173 (6, 417)	9.34 (0.31, 22.50)	24.65 (0.81, 59.39)	15.49 (0.51, 37.32)	40.88 (1.34, 98.49)
**Multiple Micronutrient Suppl.**	50%	0.81 (0.41, 1.25)	1.47 (0.31, 3.31)	64 (14, 145)	3.48 (0.72, 7.81)	9.18 (1.89, 20.60)	10.38 (2.14, 23.30)	27.40 (5.64, 61.51)
	90%	1.96 (0.77, 3.16)	3.39 (0.65, 7.97)	149 (29, 349)	8.08 (1.49, 19.27)	21.33 (3.92, 50.87)	13.40 (2.46, 31.96)	35.37 (6.50, 84.36)
**Balanced Protein Suppl.**	50%	0.02 (-0.02, 0.06)	0.03 (-0.03, 0.12)	1 (-1, 5)	0.07 (-0.07, 0.29)	0.17 (-0.19, 0.78)	10.20 (-9.51, 31.83)	26.93 (-25.10, 84.01)
	90%	0.03 (-0.03, 0.11)	0.05 (-0.06, 0.22)	2 (-2, 10)	0.12 (-0.13, 0.53)	0.31 (-0.35, 1.40)	10.20 (-9.51, 31.83)	26.93 (-25.10, 84.01)

References for Data Inputs
[1] Blencowe H, Krasevec J, Onis M De, et al. Articles National , regional , and worldwide estimates of low birthweight in 2015 , with trends from 2000: a systematic analysis. Lancet Glob Heal. 2019;(18):1-12.
[2] Chawanpaiboon S, Vogel JP, Moller AB, et al. Global, regional, and national estimates of levels of preterm birth in 2014: a systematic review and modelling analysis. Lancet Glob Heal. 2019;7(1):e37-e46.
[3] United National Population Division World Population Prospects 2019.
[4] Fink G, Peet E, Danaei G, et al. Schooling and wage income losses due to early-childhood growth faltering in developing countries: National, regional, and global estimates. Am J Clin Nutr. 2016;104(1):104-112.
[5] Country specific annual wage data from World Indicators Database. Average yearly wage was estimated to be 2/3 of the gross domestic product in 2010 constant US dollars and 2011 International dollars, adjusted for purchasing power parity.
[6] NCD Risk Factor Collaboration. Trends in adult body-mass index in 200 countries from 1975 to 2014: a pooled analysis of 1698 population-based measurement studies with 19·2 million participants. Lancet. 2016;387(10026):1377-1396.
[7] Stevens GA, Finucane MM, De-Regil LM, et al. Global, regional, and national trends in haemoglobin concentration and prevalence of total and severe anaemia in children and pregnant and non-pregnant women for 1995-2011: A systematic analysis of population-representative data. Lancet Glob Heal. 2013;1(1):16-25.
[8] Coverage of iron-folic acid supplementation abstracted from the most recent Demographic Health Survey or imputed based on sub-regional average. Indicator used: % women in the past five years who took iron tablets or syrup for >90 days.

Niger

Region: Sub-Saharan Africa**; Sub-region:** Western Sub-Saharan Africa

Low birthweight prevalence[1]: 26.7% (95% CI: 21.4, 32.0)

Preterm birth prevalence[2]: 12.0% (95% CI: 8.6, 16.7)

Number of births[3]: 5,114,000

Returns to education[4]: 8.5% (95% CI: 7.2, 9.8)

GDP per capita 2010 US$ (estimated annual wage)[5]: $386 ($258/year)

GDP per capita 2011 International $ (estimated annual wage)[5]: $903 ($602/year)

Prevalence of low BMI[6]: 12.5% (95% CI: 6.6, 19.8)

Prevalence of anemia[7]: 57.7% (95% CI: 49.3, 65.5)

Baseline coverage of IFA[8]: 28.6%

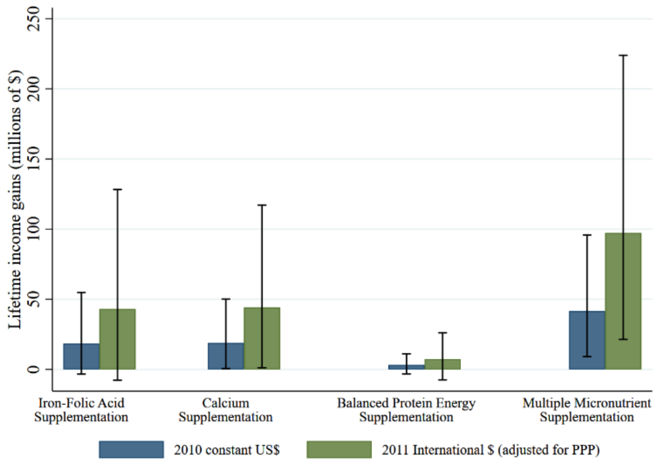

Figure: Benefits by birth cohort size for estimated gains in lifetime wages attributable to scaling up nutrition interventions to 90% target coverage

Table: Impact of maternal prenatal nutrition intervention on human capital and labour market outcomes, through improvements in low birthweight or preterm birth and schooling in Niger.

Intervention	Target Coverage (%)	Absolute reduction in birth outcome (%)	Benefits by cohorts: School years gained (in 1000 s)	No. of additional students completing secondary school	Benefits by cohorts: Lifetime wages		Returns in lifetime earnings per child born to a targeted pregnant woman	
					in US $ millions	in International $ millions	in US $	in International $
Iron/Folic Acid Suppl.	90%	2.71 (-0.52, 5.79)	31.35 (-5.78, 88.88)	176 (-32, 498)	18.43 (-3.28, 54.87)	43.11 (-7.68, 128.31)	4.00 (-0.71, 11.92)	9.37 (-1.67, 27.88)
Calcium Suppl.	50%	1.39 (0.30, 2.67)	17.73 (0.50, 47.46)	99 (3, 266)	10.50 (0.30, 27.82)	24.55 (0.69, 65.06)	4.11 (0.12, 10.88)	9.60 (0.27, 25.45)
	90%	2.50 (0.53, 4.81)	31.92 (0.90, 85.42)	179 (5, 478)	18.90 (0.53, 50.08)	44.19 (1.25, 117.12)	4.11 (0.12, 10.88)	9.60 (0.27, 25.45)
Multiple Micronutrient Suppl.	50%	2.63 (1.58, 3.86)	32.62 (7.64, 74.37)	183 (43, 416)	19.39 (4.39, 43.44)	45.34 (10.27, 101.58)	7.58 (1.72, 16.99)	17.73 (4.02, 39.73)
	90%	5.72 (2.86, 9.08)	70.25 (15.50, 161.49)	393 (87, 904)	41.60 (9.13, 95.75)	97.29 (21.34, 223.92)	9.04 (1.98, 20.80)	21.14 (4.64, 48.65)
Balanced Protein Suppl.	50%	0.27 (-0.24, 0.72)	2.94 (-2.90, 10.54)	16 (-16, 59)	1.75 (-1.77, 6.20)	4.09 (-4.13, 14.49)	5.83 (-5.02, 18.08)	13.63 (-11.75, 42.27)
	90%	0.48 (-0.43, 1.29)	5.29 (-5.22, 18.97)	30 (-29, 106)	3.15 (-3.18, 11.15)	7.36 (-7.44, 26.09)	5.83 (-5.02, 18.08)	13.63 (-11.75, 42.27)

References for Data Inputs
[1] Blencowe H, Krasevec J, Onis M De, et al. Articles National , regional , and worldwide estimates of low birthweight in 2015 , with trends from 2000: a systematic analysis. Lancet Glob Heal. 2019;(18):1-12.
[2] Chawanpaiboon S, Vogel JP, Moller AB, et al. Global, regional, and national estimates of levels of preterm birth in 2014: a systematic review and modelling analysis. Lancet Glob Heal. 2019;7(1):e37-e46.
[3] United National Population Division World Population Prospects 2019.
[4] Fink G, Peet E, Danaei G, et al. Schooling and wage income losses due to early-childhood growth faltering in developing countries: National, regional, and global estimates. Am J Clin Nutr. 2016;104(1):104-112.
[5] Country specific annual wage data from World Indicators Database. Average yearly wage was estimated to be 2/3 of the gross domestic product in 2010 constant US dollars and 2011 International dollars, adjusted for purchasing power parity.
[6] NCD Risk Factor Collaboration. Trends in adult body-mass index in 200 countries from 1975 to 2014: a pooled analysis of 1698 population-based measurement studies with 19·2 million participants. Lancet. 2016;387(10026):1377-1396.
[7] Stevens GA, Finucane MM, De-Regil LM, et al. Global, regional, and national trends in haemoglobin concentration and prevalence of total and severe anaemia in children and pregnant and non-pregnant women for 1995-2011: A systematic analysis of population-representative data. Lancet Glob Heal. 2013;1(1):16-25.
[8] Coverage of iron-folic acid supplementation abstracted from the most recent Demographic Health Survey or imputed based on sub-regional average. Indicator used: % women in the past five years who took iron tablets or syrup for >90 days.

Nigeria

Region: Sub-Saharan Africa**; Sub-region:** Western Sub-Saharan Africa

Low birthweight prevalence[1]: 11.7% (95% CI: 6.3, 17.1)

Preterm birth prevalence[2]: 11.4% (95% CI: 8.0, 15.7)

Number of births[3]: 36,886,000

Returns to education[4]: 4.1% (95% CI: 3.3, 4.9)

GDP per capita 2010 US$ (estimated annual wage)[5]: $2563 ($1709/year)

GDP per capita 2011 International $ (estimated annual wage)[5]: $5686 ($3791/year)

Prevalence of low BMI[6]: 9.5% (95% CI: 5.9, 13.9)

Prevalence of anemia[7]: 58.4% (95% CI: 38.7, 71.0)

Baseline coverage of IFA[8]: 20.5%

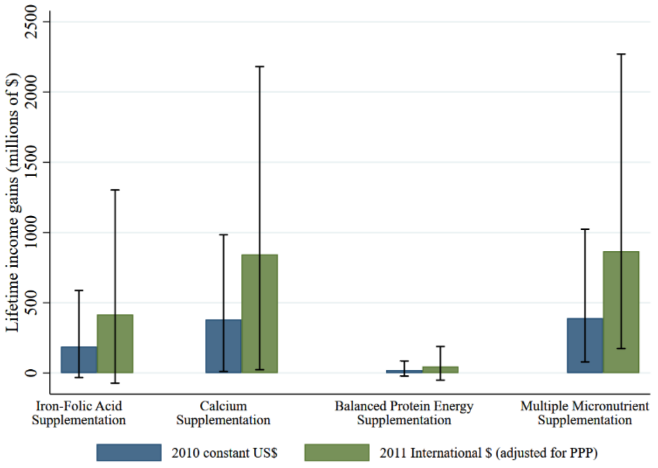

Figure: Benefits by birth cohort size for estimated gains in lifetime wages attributable to scaling up nutrition interventions to 90% target coverage

Table: Impact of maternal prenatal nutrition intervention on human capital and labour market outcomes, through improvements in low birthweight or preterm birth and schooling in Nigeria.

Intervention	Target Coverage (%)	Absolute reduction in birth outcome (%)	Benefits by cohorts: School years gained (in 1000 s)	No. of additional students completing secondary school	Benefits by cohorts: Lifetime wages		Returns in lifetime earnings per child born to a targeted pregnant woman	
					in US $ millions	in International $ millions	in US $	in International $
Iron/Folic Acid Suppl.	90%	1.30 (-0.25, 3.14)	99.45 (-18.35, 313.28)	259 (-48, 815)	187.74 (-32.61, 587.15)	416.47 (-72.33, 1302.50)	5.66 (-0.98, 17.69)	12.55 (-2.18, 39.24)
Calcium Suppl.	50%	1.32 (0.27, 2.46)	112.59 (3.59, 293.29)	293 (9, 763)	211.43 (5.79, 546.22)	469.03 (12.83, 1211.69)	11.46 (0.31, 29.62)	25.43 (0.70, 65.70)
	90%	2.38 (0.49, 4.43)	202.66 (6.45, 527.92)	527 (17, 1373)	380.58 (10.41, 983.19)	844.25 (23.10, 2181.05)	11.46 (0.31, 29.62)	25.43 (0.70, 65.70)
Multiple Micronutrient Suppl.	50%	1.31 (0.58, 2.18)	103.14 (21.03, 253.03)	268 (55, 658)	192.87 (38.93, 491.91)	427.84 (86.35, 1091.22)	10.46 (2.11, 26.67)	23.20 (4.68, 59.17)
	90%	2.66 (1.10, 4.66)	208.09 (43.30, 520.22)	541 (113, 1353)	390.36 (78.15, 1023.27)	865.95 (173.37, 2269.96)	11.76 (2.35, 30.82)	26.08 (5.22, 68.38)
Balanced Protein Suppl.	50%	0.09 (-0.08, 0.26)	6.05 (-7.08, 24.76)	16 (-18, 64)	11.67 (-12.68, 47.19)	25.88 (-28.13, 104.67)	6.99 (-7.08, 25.77)	15.52 (-15.70, 57.17)
	90%	0.15 (-0.14, 0.47)	10.89 (-12.74, 44.56)	28 (-33, 116)	21.00 (-22.83, 84.93)	46.59 (-50.64, 188.41)	6.99 (-7.08, 25.77)	15.52 (-15.70, 57.17)

References for Data Inputs

[1] Blencowe H, Krasevec J, Onis M De, et al. Articles National , regional , and worldwide estimates of low birthweight in 2015 , with trends from 2000: a systematic analysis. Lancet Glob Heal. 2019;(18):1-12.

[2] Chawanpaiboon S, Vogel JP, Moller AB, et al. Global, regional, and national estimates of levels of preterm birth in 2014: a systematic review and modelling analysis. Lancet Glob Heal. 2019;7(1):e37-e46.

[3] United National Population Division World Population Prospects 2019.

[4] Fink G, Peet E, Danaei G, et al. Schooling and wage income losses due to early-childhood growth faltering in developing countries: National, regional, and global estimates. Am J Clin Nutr. 2016;104(1):104-112.

[5] Country specific annual wage data from World Indicators Database. Average yearly wage was estimated to be 2/3 of the gross domestic product in 2010 constant US dollars and 2011 International dollars, adjusted for purchasing power parity.

[6] NCD Risk Factor Collaboration. Trends in adult body-mass index in 200 countries from 1975 to 2014: a pooled analysis of 1698 population-based measurement studies with 19·2 million participants. Lancet. 2016;387(10026):1377-1396.

[7] Stevens GA, Finucane MM, De-Regil LM, et al. Global, regional, and national trends in haemoglobin concentration and prevalence of total and severe anaemia in children and pregnant and non-pregnant women for 1995-2011: A systematic analysis of population-representative data. Lancet Glob Heal. 2013;1(1):16-25.

[8] Coverage of iron-folic acid supplementation abstracted from the most recent Demographic Health Survey or imputed based on sub-regional average. Indicator used: % women in the past five years who took iron tablets or syrup for >90 days.

Pakistan

Region: South Asia**; Sub-region:** South Asia

Low birthweight prevalence[1]: 26.0% (95% CI: 16.6, 35.5)

Preterm birth prevalence[2]: 8.4% (95% CI: 5.6, 11.9)

Number of births[3]: 29,970,000

Returns to education[4]: 8.5% (95% CI: 7.6, 9.4)

GDP per capita 2010 US$ (estimated annual wage)[5]: $1083 ($722/year)

GDP per capita 2011 International $ (estimated annual wage)[5]: $4459 ($2973/year)

Prevalence of low BMI[6]: 14.7% (95% CI: 9.0, 21.5)

Prevalence of anemia[7]: 49.7% (95% CI: 40.5, 57.0)

Baseline coverage of IFA[8]: 22.1%

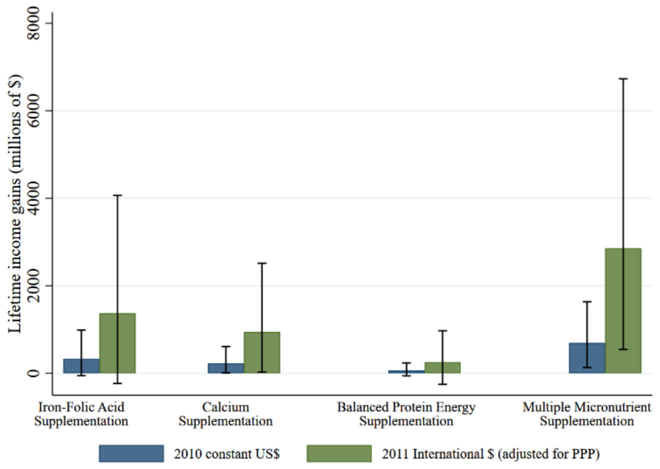

Figure: Benefits by birth cohort size for estimated gains in lifetime wages attributable to scaling up nutrition interventions to 90% target coverage

Table: Impact of maternal prenatal nutrition intervention on human capital and labour market outcomes, through improvements in low birthweight or preterm birth and schooling in Pakistan.

Intervention	Target Coverage (%)	Absolute reduction in birth outcome (%)	Benefits by cohorts: School years gained (in 1000 s)	No. of additional students completing secondary school	Benefits by cohorts: Lifetime wages		Returns in lifetime earnings per child born to a targeted pregnant woman	
					in US $ millions	in International $ millions	in US $	in International $
Iron/Folic Acid Suppl.	90%	2.90 (-0.55, 6.45)	202.29 (-35.12, 602.89)	4895 (-850, 14590)	333.23 (-56.81, 987.56)	1372.35 (-233.94, 4067.06)	12.35 (-2.11, 36.61)	50.88 (-8.67, 150.78)
Calcium Suppl.	50%	0.97 (0.19, 1.84)	77.88 (2.08, 207.94)	1885 (50, 5032)	127.16 (3.64, 339.00)	523.68 (14.98, 1396.11)	8.49 (0.24, 22.62)	34.95 (1.00, 93.17)
	90%	1.75 (0.34, 3.30)	140.19 (3.75, 374.30)	3393 (91, 9058)	228.89 (6.55, 610.20)	942.62 (26.96, 2513.00)	8.49 (0.24, 22.62)	34.95 (1.00, 93.17)
Multiple Micronutrient Suppl.	50%	2.75 (1.22, 4.45)	203.57 (39.38, 476.40)	4926 (953, 11529)	335.19 (65.43, 771.64)	1380.39 (269.45, 3177.83)	22.37 (4.37, 51.49)	92.12 (17.98, 212.07)
	90%	5.64 (2.29, 9.63)	422.31 (80.24, 989.81)	10220 (1942, 23953)	693.65 (131.93, 1634.07)	2856.65 (543.31, 6729.58)	25.72 (4.89, 60.58)	105.91 (20.14, 249.49)
Balanced Protein Suppl.	50%	0.30 (-0.26, 0.84)	20.92 (-21.27, 75.68)	506 (-515, 1831)	34.62 (-33.82, 131.08)	142.57 (-139.29, 539.84)	16.12 (-15.17, 53.85)	66.37 (-62.46, 221.78)
	90%	0.55 (-0.46, 1.51)	37.66 (-38.29, 136.22)	911 (-927, 3296)	62.31 (-60.88, 235.95)	256.63 (-250.73, 971.70)	16.12 (-15.17, 53.85)	66.37 (-62.46, 221.78)

References for Data Inputs

[1] Blencowe H, Krasevec J, Onis M De, et al. Articles National , regional , and worldwide estimates of low birthweight in 2015 , with trends from 2000: a systematic analysis. Lancet Glob Heal. 2019;(18):1-12.
[2] Chawanpaiboon S, Vogel JP, Moller AB, et al. Global, regional, and national estimates of levels of preterm birth in 2014: a systematic review and modelling analysis. Lancet Glob Heal. 2019;7(1):e37-e46.
[3] United National Population Division World Population Prospects 2019.
[4] Fink G, Peet E, Danaei G, et al. Schooling and wage income losses due to early-childhood growth faltering in developing countries: National, regional, and global estimates. Am J Clin Nutr. 2016;104(1):104-112.
[5] Country specific annual wage data from World Indicators Database. Average yearly wage was estimated to be 2/3 of the gross domestic product in 2010 constant US dollars and 2011 International dollars, adjusted for purchasing power parity.
[6] NCD Risk Factor Collaboration. Trends in adult body-mass index in 200 countries from 1975 to 2014: a pooled analysis of 1698 population-based measurement studies with 19·2 million participants. Lancet. 2016;387(10026):1377-1396.
[7] Stevens GA, Finucane MM, De-Regil LM, et al. Global, regional, and national trends in haemoglobin concentration and prevalence of total and severe anaemia in children and pregnant and non-pregnant women for 1995-2011: A systematic analysis of population-representative data. Lancet Glob Heal. 2013;1(1):16-25.
[8] Coverage of iron-folic acid supplementation abstracted from the most recent Demographic Health Survey or imputed based on sub-regional average. Indicator used: % women in the past five years who took iron tablets or syrup for >90 days.

Palestine

Region: North Africa and Middle East**; Sub-region:** North Africa and Middle East

Low birthweight prevalence[1]: 10.9% (95% CI: 6.9, 14.9)

Preterm birth prevalence[2]: 10.3% (95% CI: 9.1, 11.6)

Number of births[3]: 708,000

Returns to education[4]: 2.7% (95% CI: 2.5, 2.8)

GDP per capita 2010 US$ (estimated annual wage)[5]: $2632 ($1755/year)

GDP per capita 2011 International $ (estimated annual wage)[5]: $4494 ($2996/year)

Prevalence of low BMI[6]: 3.8% (95% CI: 1.9, 5.8)

Prevalence of anemia[7]: 31.0% (95% CI: 9.6, 52.7)

Baseline coverage of IFA[8]: 24.3%

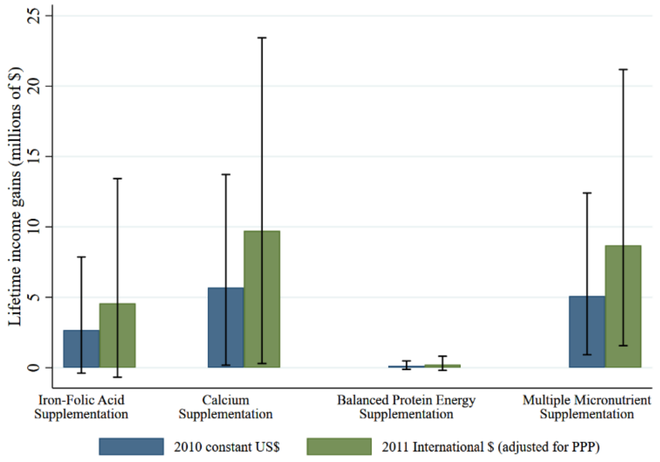

Figure: Benefits by birth cohort size for estimated gains in lifetime wages attributable to scaling up nutrition interventions to 90% target coverage

Table: Impact of maternal prenatal nutrition intervention on human capital and labour market outcomes, through improvements in low birthweight or preterm birth and schooling in Palestine.

Intervention	Target Coverage (%)	Absolute reduction in birth outcome (%)	Benefits by cohorts: School years gained (in 1000 s)	No. of additional students completing secondary school	Benefits by cohorts: Lifetime wages		Returns in lifetime earnings per child born to a targeted pregnant woman	
					in US $ millions	in International $ millions	in US $	in International $
Iron/Folic Acid Suppl.	90%	1.19 (-0.20, 2.61)	2.12 (-0.30, 6.36)	72 (-10, 216)	2.67 (-0.39, 7.87)	4.57 (-0.67, 13.43)	4.20 (-0.62, 12.35)	7.16 (-1.06, 21.08)
Calcium Suppl.	50%	1.25 (0.29, 2.06)	2.48 (0.08, 5.93)	84 (3, 202)	3.16 (0.10, 7.62)	5.40 (0.16, 13.02)	8.94 (0.27, 21.53)	15.26 (0.46, 36.77)
	90%	2.24 (0.52, 3.71)	4.46 (0.14, 10.68)	152 (5, 363)	5.69 (0.17, 13.72)	9.72 (0.30, 23.43)	8.94 (0.27, 21.53)	15.26 (0.46, 36.77)
Multiple Micronutrient Suppl.	50%	1.01 (0.44, 1.69)	1.91 (0.36, 4.56)	65 (12, 155)	2.42 (0.45, 5.75)	4.12 (0.78, 9.81)	6.82 (1.28, 16.23)	11.65 (2.19, 27.71)
	90%	2.16 (0.79, 3.74)	4.02 (0.69, 9.74)	137 (24, 331)	5.09 (0.91, 12.40)	8.68 (1.56, 21.18)	7.98 (1.43, 19.47)	13.63 (2.45, 33.24)
Balanced Protein Suppl.	50%	0.03 (-0.02, 0.09)	0.05 (-0.05, 0.20)	2 (-2, 7)	0.07 (-0.06, 0.26)	0.12 (-0.11, 0.45)	5.41 (-5.14, 18.70)	9.23 (-8.78, 31.93)
	90%	0.06 (-0.04, 0.17)	0.10 (-0.09, 0.36)	3 (-3, 12)	0.12 (-0.11, 0.47)	0.21 (-0.19, 0.81)	5.41 (-5.14, 18.70)	9.23 (-8.78, 31.93)

References for Data Inputs

[1] Blencowe H, Krasevec J, Onis M De, et al. Articles National , regional , and worldwide estimates of low birthweight in 2015 , with trends from 2000: a systematic analysis. Lancet Glob Heal. 2019;(18):1-12.

[2] Chawanpaiboon S, Vogel JP, Moller AB, et al. Global, regional, and national estimates of levels of preterm birth in 2014: a systematic review and modelling analysis. Lancet Glob Heal. 2019;7(1):e37-e46.

[3] United National Population Division World Population Prospects 2019.

[4] Fink G, Peet E, Danaei G, et al. Schooling and wage income losses due to early-childhood growth faltering in developing countries: National, regional, and global estimates. Am J Clin Nutr. 2016;104(1):104-112.

[5] Country specific annual wage data from World Indicators Database. Average yearly wage was estimated to be 2/3 of the gross domestic product in 2010 constant US dollars and 2011 International dollars, adjusted for purchasing power parity.

[6] NCD Risk Factor Collaboration. Trends in adult body-mass index in 200 countries from 1975 to 2014: a pooled analysis of 1698 population-based measurement studies with 19·2 million participants. Lancet. 2016;387(10026):1377-1396.

[7] Stevens GA, Finucane MM, De-Regil LM, et al. Global, regional, and national trends in haemoglobin concentration and prevalence of total and severe anaemia in children and pregnant and non-pregnant women for 1995-2011: A systematic analysis of population-representative data. Lancet Glob Heal. 2013;1(1):16-25.

[8] Coverage of iron-folic acid supplementation abstracted from the most recent Demographic Health Survey or imputed based on sub-regional average. Indicator used: % women in the past five years who took iron tablets or syrup for >90 days.

Papua New Guinea

Region: Southeast Asia, East Asia, and Oceania**; Sub-region:** Oceania

Low birthweight prevalence[1]: 10.0% (95% CI: 7.3, 12.7)

Preterm birth prevalence[2]: 10.0% (95% CI: 7.9, 12.7)

Number of births[3]: 1,161,000

Returns to education[4]: 6.1% (95% CI: 2.7, 9.6)

GDP per capita 2010 US$ (estimated annual wage)[5]: $2400 ($1600/year)

GDP per capita 2011 International $ (estimated annual wage)[5]: $3820 ($2547/year)

Prevalence of low BMI[6]: 2.9% (95% CI: 0.9, 6.6)

Prevalence of anemia[7]: 37.3% (95% CI: 17.7, 62.0)

Baseline coverage of IFA[8]: 44.2%

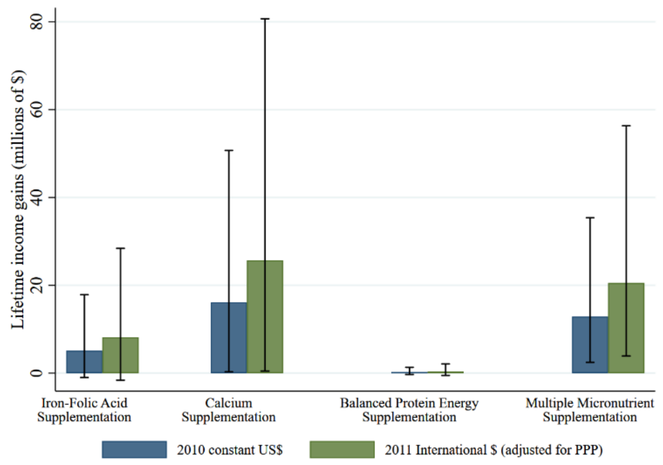

Figure: Benefits by birth cohort size for estimated gains in lifetime wages attributable to scaling up nutrition interventions to 90% target coverage

Table: Impact of maternal prenatal nutrition intervention on human capital and labour market outcomes, through improvements in low birthweight or preterm birth and schooling in Papua New Guinea.

Intervention	Target Coverage (%)	Absolute reduction in birth outcome (%)	Benefits by cohorts: School years gained (in 1000 s)	No. of additional students completing secondary school	Benefits by cohorts: Lifetime wages		Returns in lifetime earnings per child born to a targeted pregnant woman	
					in US $ millions	in International $ millions	in US $	in International $
Iron/Folic Acid Suppl.	90%	0.77 (-0.14, 1.68)	2.15 (-0.39, 6.27)	24 (-4, 69)	5.12 (-1.00, 17.85)	8.16 (-1.60, 28.41)	4.90 (-0.96, 17.08)	7.81 (-1.53, 27.19)
Calcium Suppl.	50%	1.19 (0.27, 2.11)	3.67 (0.11, 9.11)	40 (1, 100)	8.96 (0.17, 28.16)	14.26 (0.27, 44.83)	15.43 (0.29, 48.51)	24.56 (0.47, 77.23)
	90%	2.15 (0.49, 3.80)	6.60 (0.19, 16.40)	73 (2, 180)	16.12 (0.31, 50.69)	25.66 (0.49, 80.70)	15.43 (0.29, 48.51)	24.56 (0.47, 77.23)
Multiple Micronutrient Suppl.	50%	0.67 (0.37, 1.00)	2.02 (0.44, 4.31)	22 (5, 47)	4.83 (0.84, 13.46)	7.69 (1.34, 21.42)	8.32 (1.45, 23.18)	13.25 (2.30, 36.90)
	90%	1.80 (0.84, 2.79)	5.21 (1.13, 12.00)	57 (12, 132)	12.90 (2.45, 35.37)	20.53 (3.90, 56.31)	12.34 (2.34, 33.85)	19.65 (3.73, 53.89)
Balanced Protein Suppl.	50%	0.02 (-0.02, 0.08)	0.06 (-0.07, 0.26)	1 (-1, 3)	0.13 (-0.18, 0.73)	0.21 (-0.28, 1.16)	9.28 (-9.71, 38.03)	14.77 (-15.45, 60.54)
	90%	0.04 (-0.04, 0.14)	0.10 (-0.12, 0.46)	1 (-1, 5)	0.24 (-0.32, 1.31)	0.39 (-0.51, 2.09)	9.28 (-9.71, 38.03)	14.77 (-15.45, 60.54)

References for Data Inputs

[1] Blencowe H, Krasevec J, Onis M De, et al. Articles National , regional , and worldwide estimates of low birthweight in 2015 , with trends from 2000: a systematic analysis. Lancet Glob Heal. 2019;(18):1-12.

[2] Chawanpaiboon S, Vogel JP, Moller AB, et al. Global, regional, and national estimates of levels of preterm birth in 2014: a systematic review and modelling analysis. Lancet Glob Heal. 2019;7(1):e37-e46.

[3] United National Population Division World Population Prospects 2019.

[4] Fink G, Peet E, Danaei G, et al. Schooling and wage income losses due to early-childhood growth faltering in developing countries: National, regional, and global estimates. Am J Clin Nutr. 2016;104(1):104-112.

[5] Country specific annual wage data from World Indicators Database. Average yearly wage was estimated to be 2/3 of the gross domestic product in 2010 constant US dollars and 2011 International dollars, adjusted for purchasing power parity.

[6] NCD Risk Factor Collaboration. Trends in adult body-mass index in 200 countries from 1975 to 2014: a pooled analysis of 1698 population-based measurement studies with 19·2 million participants. Lancet. 2016;387(10026):1377-1396.

[7] Stevens GA, Finucane MM, De-Regil LM, et al. Global, regional, and national trends in haemoglobin concentration and prevalence of total and severe anaemia in children and pregnant and non-pregnant women for 1995-2011: A systematic analysis of population-representative data. Lancet Glob Heal. 2013;1(1):16-25.

[8] Coverage of iron-folic acid supplementation abstracted from the most recent Demographic Health Survey or imputed based on sub-regional average. Indicator used: % women in the past five years who took iron tablets or syrup for >90 days.

Paraguay

Region: Latin America and Caribbean**; Sub-region:** Tropical Latin America

Low birthweight prevalence[1]: 8.1% (95% CI: 6.5, 10.6)

Preterm birth prevalence[2]: 8.1% (95% CI: 6.4, 9.9)

Number of births[3]: 716,000

Returns to education[4]: 13.3% (95% CI: 11.6, 15.0)

GDP per capita 2010 US$ (estimated annual wage)[5]: $4944 ($3296/year)

GDP per capita 2011 International $ (estimated annual wage)[5]: $11056 ($7371/year)

Prevalence of low BMI[6]: 2.2% (95% CI: 0.6, 5.3)

Prevalence of anemia[7]: 31.5% (95% CI: 12.6, 59.2)

Baseline coverage of IFA[8]: 34.2%

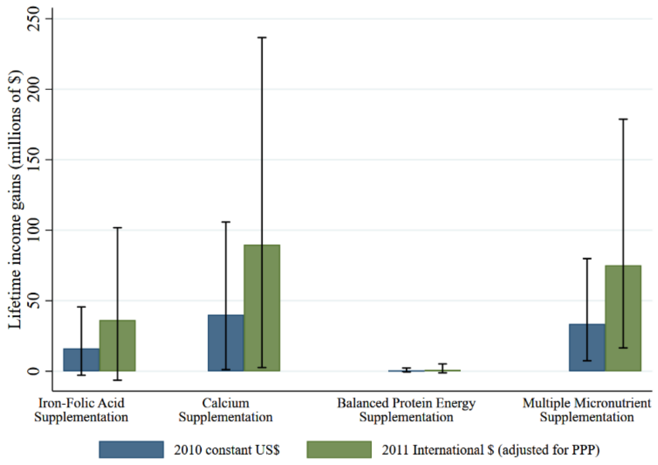

Figure: Benefits by birth cohort size for estimated gains in lifetime wages attributable to scaling up nutrition interventions to 90% target coverage

Table: Impact of maternal prenatal nutrition intervention on human capital and labour market outcomes, through improvements in low birthweight or preterm birth and schooling in Paraguay.

Intervention	Target Coverage (%)	Absolute reduction in birth outcome (%)	Benefits by cohorts: School years gained (in 1000 s)	No. of additional students completing secondary school	Benefits by cohorts: Lifetime wages		Returns in lifetime earnings per child born to a targeted pregnant woman	
					in US $ millions	in International $ millions	in US $	in International $
Iron/Folic Acid Suppl.	90%	0.76 (-0.15, 1.60)	1.37 (-0.23, 3.87)	45 (-8, 129)	16.21 (-2.83, 45.55)	36.25 (-6.32, 101.85)	25.16 (-4.39, 70.68)	56.26 (-9.81, 158.05)
Calcium Suppl.	50%	0.96 (0.19, 1.64)	1.91 (0.06, 4.79)	63 (2, 159)	22.29 (0.66, 58.79)	49.83 (1.47, 131.47)	62.25 (1.84, 164.22)	139.20 (4.11, 367.23)
	90%	1.73 (0.35, 2.95)	3.44 (0.10, 8.63)	114 (3, 286)	40.11 (1.19, 105.83)	89.70 (2.65, 236.65)	62.25 (1.84, 164.22)	139.20 (4.11, 367.23)
Multiple Micronutrient Suppl.	50%	0.64 (0.34, 0.99)	1.21 (0.27, 2.72)	40 (9, 90)	14.40 (3.40, 32.28)	32.20 (7.61, 72.19)	40.22 (9.51, 90.18)	89.93 (21.26, 201.66)
	90%	1.52 (0.67, 2.48)	2.85 (0.60, 6.68)	95 (20, 222)	33.60 (7.41, 79.92)	75.14 (16.57, 178.72)	52.15 (11.50, 124.02)	116.61 (25.71, 277.34)
Balanced Protein Suppl.	50%	0.01 (-0.01, 0.05)	0.02 (-0.03, 0.11)	1 (-1, 4)	0.24 (-0.29, 1.28)	0.54 (-0.65, 2.87)	37.95 (-36.12, 120.69)	84.87 (-80.76, 269.89)
	90%	0.02 (-0.02, 0.09)	0.04 (-0.05, 0.20)	1 (-2, 7)	0.44 (-0.52, 2.31)	0.98 (-1.16, 5.16)	37.95 (-36.12, 120.69)	84.87 (-80.76, 269.89)

References for Data Inputs

[1] Blencowe H, Krasevec J, Onis M De, et al. Articles National , regional , and worldwide estimates of low birthweight in 2015 , with trends from 2000: a systematic analysis. Lancet Glob Heal. 2019;(18):1-12.
[2] Chawanpaiboon S, Vogel JP, Moller AB, et al. Global, regional, and national estimates of levels of preterm birth in 2014: a systematic review and modelling analysis. Lancet Glob Heal. 2019;7(1):e37-e46.
[3] United National Population Division World Population Prospects 2019.
[4] Fink G, Peet E, Danaei G, et al. Schooling and wage income losses due to early-childhood growth faltering in developing countries: National, regional, and global estimates. Am J Clin Nutr. 2016;104(1):104-112.
[5] Country specific annual wage data from World Indicators Database. Average yearly wage was estimated to be 2/3 of the gross domestic product in 2010 constant US dollars and 2011 International dollars, adjusted for purchasing power parity.
[6] NCD Risk Factor Collaboration. Trends in adult body-mass index in 200 countries from 1975 to 2014: a pooled analysis of 1698 population-based measurement studies with 19·2 million participants. Lancet. 2016;387(10026):1377-1396.
[7] Stevens GA, Finucane MM, De-Regil LM, et al. Global, regional, and national trends in haemoglobin concentration and prevalence of total and severe anaemia in children and pregnant and non-pregnant women for 1995-2011: A systematic analysis of population-representative data. Lancet Glob Heal. 2013;1(1):16-25.
[8] Coverage of iron-folic acid supplementation abstracted from the most recent Demographic Health Survey or imputed based on sub-regional average. Indicator used: % women in the past five years who took iron tablets or syrup for >90 days.

Peru

Region: Latin America and Caribbean**; Sub-region:** Andean Latin America

Low birthweight prevalence[1]: 9.4% (95% CI: 7.2, 12.2)

Preterm birth prevalence[2]: 8.8% (95% CI: 6.9, 11.0)

Number of births[3]: 2,870,000

Returns to education[4]: 6.8% (95% CI: 0.9, 12.6)

GDP per capita 2010 US$ (estimated annual wage)[5]: $6114 ($4076/year)

GDP per capita 2011 International $ (estimated annual wage)[5]: $12121 ($8081/year)

Prevalence of low BMI[6]: 1.4% (95% CI: 0.8, 2.2)

Prevalence of anemia[7]: 24.4% (95% CI: 20.3, 29.1)

Baseline coverage of IFA[8]: 44.7%

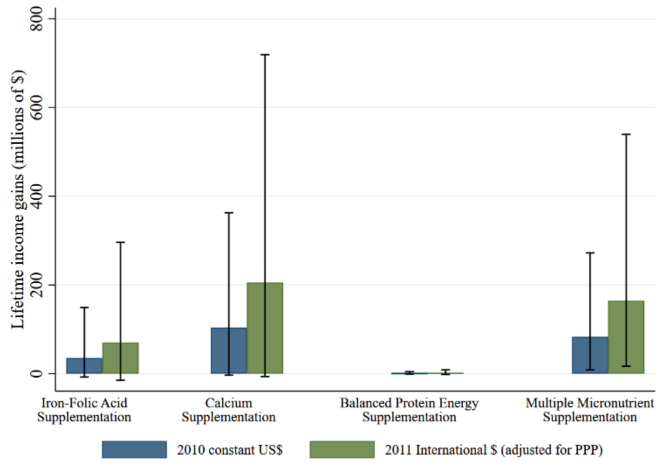

Figure: Benefits by birth cohort size for estimated gains in lifetime wages attributable to scaling up nutrition interventions to 90% target coverage

Table: Impact of maternal prenatal nutrition intervention on human capital and labour market outcomes, through improvements in low birthweight or preterm birth and schooling in Peru.

Intervention	Target Coverage (%)	Absolute reduction in birth outcome (%)	Benefits by cohorts: School years gained (in 1000 s)	No. of additional students completing secondary school	Benefits by cohorts: Lifetime wages		Returns in lifetime earnings per child born to a targeted pregnant woman	
					in US $ millions	in International $ millions	in US $	in International $
Iron/Folic Acid Suppl.	90%	0.74 (-0.13, 1.63)	5.31 (-0.92, 15.63)	74 (-13, 219)	35.33 (-7.37, 149.32)	70.03 (-14.62, 296.01)	13.68 (-2.85, 57.81)	27.11 (-5.66, 114.60)
Calcium Suppl.	50%	1.04 (0.22, 1.85)	8.38 (0.30, 21.42)	117 (4, 300)	57.56 (-1.81, 201.45)	114.10 (-3.59, 399.36)	40.11 (-1.26, 140.39)	79.51 (-2.51, 278.30)
	90%	1.88 (0.39, 3.34)	15.08 (0.55, 38.56)	211 (8, 540)	103.60 (-3.26, 362.62)	205.38 (-6.47, 718.85)	40.11 (-1.26, 140.39)	79.51 (-2.51, 278.30)
Multiple Micronutrient Suppl.	50%	0.56 (0.30, 0.84)	4.46 (0.87, 9.51)	62 (12, 133)	29.92 (3.09, 94.33)	59.31 (6.14, 187.00)	20.85 (2.16, 65.74)	41.33 (4.28, 130.32)
	90%	1.60 (0.71, 2.58)	12.30 (2.58, 27.52)	172 (36, 385)	82.91 (8.58, 272.14)	164.36 (17.01, 539.49)	32.10 (3.32, 105.36)	63.63 (6.59, 208.86)
Balanced Protein Suppl.	50%	0.01 (-0.01, 0.03)	0.07 (-0.07, 0.28)	1 (-1, 4)	0.45 (-0.51, 2.41)	0.89 (-1.01, 4.77)	23.89 (-25.31, 113.75)	47.35 (-50.17, 225.50)
	90%	0.02 (-0.02, 0.05)	0.13 (-0.12, 0.50)	2 (-2, 7)	0.81 (-0.92, 4.33)	1.61 (-1.82, 8.59)	23.89 (-25.31, 113.75)	47.35 (-50.17, 225.50)

References for Data Inputs
[1] Blencowe H, Krasevec J, Onis M De, et al. Articles National , regional , and worldwide estimates of low birthweight in 2015 , with trends from 2000: a systematic analysis. Lancet Glob Heal. 2019;(18):1-12.
[2] Chawanpaiboon S, Vogel JP, Moller AB, et al. Global, regional, and national estimates of levels of preterm birth in 2014: a systematic review and modelling analysis. Lancet Glob Heal. 2019;7(1):e37-e46.
[3] United National Population Division World Population Prospects 2019.
[4] Fink G, Peet E, Danaei G, et al. Schooling and wage income losses due to early-childhood growth faltering in developing countries: National, regional, and global estimates. Am J Clin Nutr. 2016;104(1):104-112.
[5] Country specific annual wage data from World Indicators Database. Average yearly wage was estimated to be 2/3 of the gross domestic product in 2010 constant US dollars and 2011 International dollars, adjusted for purchasing power parity.
[6] NCD Risk Factor Collaboration. Trends in adult body-mass index in 200 countries from 1975 to 2014: a pooled analysis of 1698 population-based measurement studies with 19·2 million participants. Lancet. 2016;387(10026):1377-1396.
[7] Stevens GA, Finucane MM, De-Regil LM, et al. Global, regional, and national trends in haemoglobin concentration and prevalence of total and severe anaemia in children and pregnant and non-pregnant women for 1995-2011: A systematic analysis of population-representative data. Lancet Glob Heal. 2013;1(1):16-25.
[8] Coverage of iron-folic acid supplementation abstracted from the most recent Demographic Health Survey or imputed based on sub-regional average. Indicator used: % women in the past five years who took iron tablets or syrup for >90 days.

Philippines

Region: Southeast Asia, East Asia, and Oceania**; Sub-region:** Southeast Asia

Low birthweight prevalence[1]: 20.1% (95% CI: 14.2, 26.1)

Preterm birth prevalence[2]: 13.3% (95% CI: 9.1, 18.5)

Number of births[3]: 10,889,000

Returns to education[4]: 9.1% (95% CI: 5.3, 12.9)

GDP per capita 2010 US$ (estimated annual wage)[5]: $2605 ($1737/year)

GDP per capita 2011 International $ (estimated annual wage)[5]: $6848 ($4565/year)

Prevalence of low BMI[6]: 13.6% (95% CI: 8.4, 19.7)

Prevalence of anemia[7]: 33.6% (95% CI: 20.8, 50.2)

Baseline coverage of IFA[8]: 50.6%

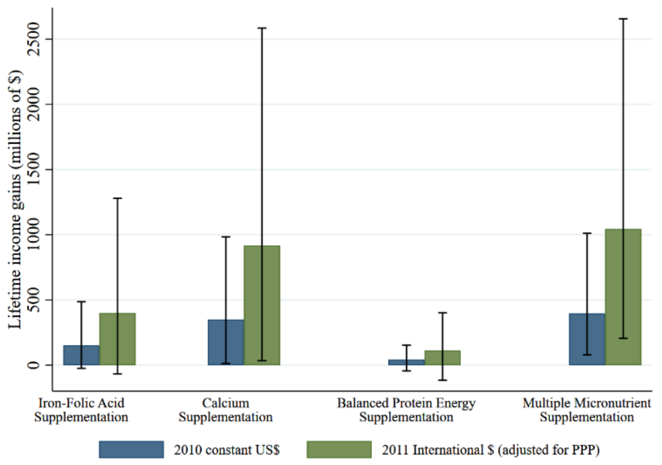

Figure: Benefits by birth cohort size for estimated gains in lifetime wages attributable to scaling up nutrition interventions to 90% target coverage

Table: Impact of maternal prenatal nutrition intervention on human capital and labour market outcomes, through improvements in low birthweight or preterm birth and schooling in Philippines.

Intervention	Target Coverage (%)	Absolute reduction in birth outcome (%)	Benefits by cohorts: School years gained (in 1000 s)	No. of additional students completing secondary school	Benefits by cohorts: Lifetime wages		Returns in lifetime earnings per child born to a targeted pregnant woman	
					in US $ millions	in International $ millions	in US $	in International $
Iron/Folic Acid Suppl.	90%	1.37 (-0.25, 3.04)	37.16 (-5.39, 107.02)	30 (-4, 86)	151.92 (-25.16, 486.88)	399.28 (-66.12, 1279.65)	15.50 (-2.57, 49.68)	40.74 (-6.75, 130.58)
Calcium Suppl.	50%	1.52 (0.32, 2.93)	46.60 (1.59, 123.53)	37 (1, 99)	194.23 (7.25, 546.49)	510.48 (19.05, 1436.31)	35.67 (1.33, 100.38)	93.76 (3.50, 263.81)
	90%	2.73 (0.58, 5.27)	83.88 (2.86, 222.35)	67 (2, 178)	349.61 (13.05, 983.69)	918.86 (34.29, 2585.36)	35.67 (1.33, 100.38)	93.76 (3.50, 263.81)
Multiple Micronutrient Suppl.	50%	1.22 (0.60, 1.93)	35.20 (6.59, 81.19)	28 (5, 65)	147.49 (26.03, 380.05)	387.63 (68.42, 998.87)	27.09 (4.78, 69.80)	71.20 (12.57, 183.46)
	90%	3.36 (1.58, 5.46)	96.33 (20.14, 219.77)	77 (16, 176)	397.49 (78.32, 1010.86)	1044.71 (205.84, 2656.79)	40.56 (7.99, 103.15)	106.60 (21.00, 271.10)
Balanced Protein Suppl.	50%	0.22 (-0.18, 0.58)	5.80 (-6.04, 19.57)	5 (-5, 16)	23.75 (-24.32, 84.91)	62.41 (-63.92, 223.16)	33.49 (-29.36, 110.93)	88.02 (-77.17, 291.54)
	90%	0.40 (-0.33, 1.05)	10.44 (-10.88, 35.23)	8 (-9, 28)	42.75 (-43.78, 152.83)	112.35 (-115.05, 401.68)	33.49 (-29.36, 110.93)	88.02 (-77.17, 291.54)

References for Data Inputs

[1] Blencowe H, Krasevec J, Onis M De, et al. Articles National , regional , and worldwide estimates of low birthweight in 2015 , with trends from 2000: a systematic analysis. Lancet Glob Heal. 2019;(18):1-12.
[2] Chawanpaiboon S, Vogel JP, Moller AB, et al. Global, regional, and national estimates of levels of preterm birth in 2014: a systematic review and modelling analysis. Lancet Glob Heal. 2019;7(1):e37-e46.
[3] United National Population Division World Population Prospects 2019.
[4] Fink G, Peet E, Danaei G, et al. Schooling and wage income losses due to early-childhood growth faltering in developing countries: National, regional, and global estimates. Am J Clin Nutr. 2016;104(1):104-112.
[5] Country specific annual wage data from World Indicators Database. Average yearly wage was estimated to be 2/3 of the gross domestic product in 2010 constant US dollars and 2011 International dollars, adjusted for purchasing power parity.
[6] NCD Risk Factor Collaboration. Trends in adult body-mass index in 200 countries from 1975 to 2014: a pooled analysis of 1698 population-based measurement studies with 19·2 million participants. Lancet. 2016;387(10026):1377-1396.
[7] Stevens GA, Finucane MM, De-Regil LM, et al. Global, regional, and national trends in haemoglobin concentration and prevalence of total and severe anaemia in children and pregnant and non-pregnant women for 1995-2011: A systematic analysis of population-representative data. Lancet Glob Heal. 2013;1(1):16-25.
[8] Coverage of iron-folic acid supplementation abstracted from the most recent Demographic Health Survey or imputed based on sub-regional average. Indicator used: % women in the past five years who took iron tablets or syrup for >90 days.

Romania

Region: Central Europe, Eastern Europe, Central Asia**; Sub-region:** Central Europe

Low birthweight prevalence[1]: 8.2% (95% CI: 6.5, 10.5)

Preterm birth prevalence[2]: 8.7% (95% CI: 6.3, 13.3)

Number of births[3]: 958,000

Returns to education[4]: 6.7% (95% CI: 5.3, 8.1)

GDP per capita 2010 US$ (estimated annual wage)[5]: $9712 ($6475/year)

GDP per capita 2011 International $ (estimated annual wage)[5]: $20666 ($13777/year)

Prevalence of low BMI[6]: 2.1% (95% CI: 0.9, 3.9)

Prevalence of anemia[7]: 26.2% (95% CI: 12.6, 48.7)

Baseline coverage of IFA[8]: 18.5%

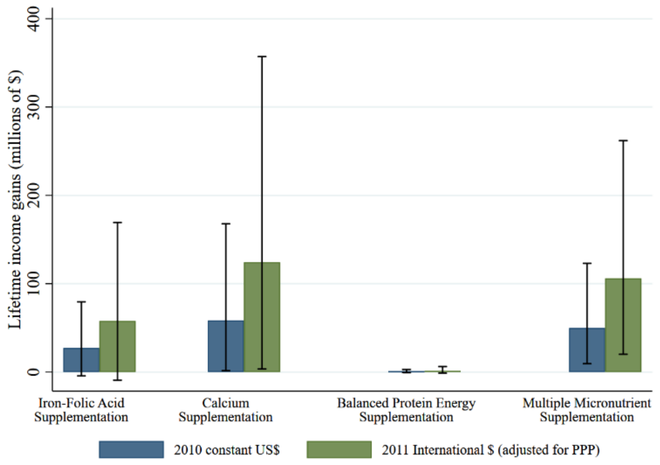

Figure: Benefits by birth cohort size for estimated gains in lifetime wages attributable to scaling up nutrition interventions to 90% target coverage

Table: Impact of maternal prenatal nutrition intervention on human capital and labour market outcomes, through improvements in low birthweight or preterm birth and schooling in Romania.

Intervention	Target Coverage (%)	Absolute reduction in birth outcome (%)	Benefits by cohorts: School years gained (in 1000 s)	No. of additional students completing secondary school	Benefits by cohorts: Lifetime wages		Returns in lifetime earnings per child born to a targeted pregnant woman	
					in US $ millions	in International $ millions	in US $	in International $
Iron/Folic Acid Suppl.	90%	0.95 (-0.19, 2.00)	2.32 (-0.33, 6.48)	82 (-12, 230)	27.20 (-4.31, 79.53)	57.88 (-9.17, 169.22)	31.55 (-5.00, 92.24)	67.13 (-10.64, 196.27)
Calcium Suppl.	50%	1.02 (0.20, 2.00)	2.80 (0.08, 7.53)	99 (3, 266)	32.44 (0.91, 93.23)	69.03 (1.94, 198.37)	67.72 (1.91, 194.62)	144.11 (4.06, 414.12)
	90%	1.83 (0.36, 3.60)	5.04 (0.15, 13.55)	178 (5, 480)	58.39 (1.64, 167.81)	124.25 (3.50, 357.06)	67.72 (1.91, 194.62)	144.11 (4.06, 414.12)
Multiple Micronutrient Suppl.	50%	0.84 (0.35, 1.32)	2.15 (0.44, 4.85)	76 (16, 172)	24.32 (5.16, 59.61)	51.75 (10.97, 126.85)	50.77 (10.77, 124.45)	108.03 (22.91, 264.81)
	90%	1.71 (0.64, 2.75)	4.33 (0.82, 10.01)	153 (29, 354)	49.79 (9.47, 123.13)	105.95 (20.14, 261.99)	57.75 (10.98, 142.81)	122.89 (23.36, 303.86)
Balanced Protein Suppl.	50%	0.01 (-0.01, 0.04)	0.03 (-0.03, 0.14)	1 (-1, 5)	0.35 (-0.33, 1.63)	0.74 (-0.71, 3.47)	39.22 (-37.80, 135.12)	83.46 (-80.44, 287.51)
	90%	0.02 (-0.02, 0.07)	0.05 (-0.05, 0.25)	2 (-2, 9)	0.62 (-0.60, 2.93)	1.33 (-1.28, 6.24)	39.22 (-37.80, 135.12)	83.46 (-80.44, 287.51)

References for Data Inputs

[1] Blencowe H, Krasevec J, Onis M De, et al. Articles National , regional , and worldwide estimates of low birthweight in 2015 , with trends from 2000: a systematic analysis. Lancet Glob Heal. 2019;(18):1-12.
[2] Chawanpaiboon S, Vogel JP, Moller AB, et al. Global, regional, and national estimates of levels of preterm birth in 2014: a systematic review and modelling analysis. Lancet Glob Heal. 2019;7(1):e37-e46.
[3] United National Population Division World Population Prospects 2019.
[4] Fink G, Peet E, Danaei G, et al. Schooling and wage income losses due to early-childhood growth faltering in developing countries: National, regional, and global estimates. Am J Clin Nutr. 2016;104(1):104-112.
[5] Country specific annual wage data from World Indicators Database. Average yearly wage was estimated to be 2/3 of the gross domestic product in 2010 constant US dollars and 2011 International dollars, adjusted for purchasing power parity.
[6] NCD Risk Factor Collaboration. Trends in adult body-mass index in 200 countries from 1975 to 2014: a pooled analysis of 1698 population-based measurement studies with 19·2 million participants. Lancet. 2016;387(10026):1377-1396.
[7] Stevens GA, Finucane MM, De-Regil LM, et al. Global, regional, and national trends in haemoglobin concentration and prevalence of total and severe anaemia in children and pregnant and non-pregnant women for 1995-2011: A systematic analysis of population-representative data. Lancet Glob Heal. 2013;1(1):16-25.
[8] Coverage of iron-folic acid supplementation abstracted from the most recent Demographic Health Survey or imputed based on sub-regional average. Indicator used: % women in the past five years who took iron tablets or syrup for >90 days.

Russia

Region: Central Europe, Eastern Europe, Central Asia**; Sub-region:** Eastern Europe

Low birthweight prevalence[1]: 5.8% (95% CI: 5.5, 6.1)

Preterm birth prevalence[2]: 8.6% (95% CI: 2.2, 22.4)

Number of births[3]: 9,288,000

Returns to education[4]: 6.7% (95% CI: 5.3, 8.1)

GDP per capita 2010 US$ (estimated annual wage)[5]: $11282 ($7521/year)

GDP per capita 2011 International $ (estimated annual wage)[5]: $24517 ($16344/year)

Prevalence of low BMI[6]: 2.1% (95% CI: 1.1, 3.5)

Prevalence of anemia[7]: 23.4% (95% CI: 9.7, 43.8)

Baseline coverage of IFA[8]: 18.5%

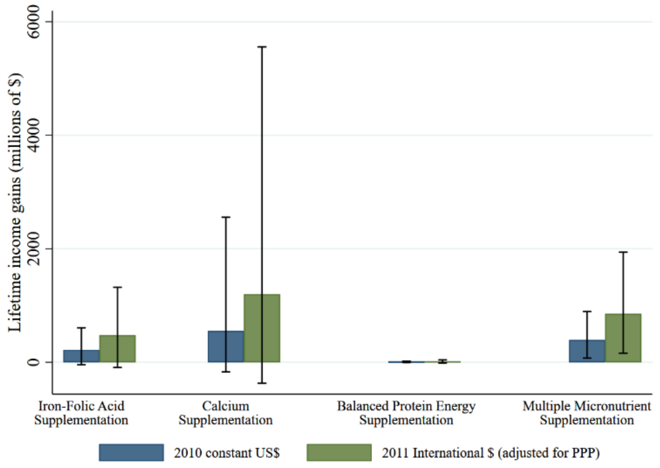

Figure: Benefits by birth cohort size for estimated gains in lifetime wages attributable to scaling up nutrition interventions to 90% target coverage

Table: Impact of maternal prenatal nutrition intervention on human capital and labour market outcomes, through improvements in low birthweight or preterm birth and schooling in Russia.

Intervention	Target Coverage (%)	Absolute reduction in birth outcome (%)	Benefits by cohorts: School years gained (in 1000 s)	No. of additional students completing secondary school	Benefits by cohorts: Lifetime wages		Returns in lifetime earnings per child born to a targeted pregnant woman	
					in US $ millions	in International $ millions	in US $	in International $
Iron/Folic Acid Suppl.	90%	0.69 (-0.12, 1.36)	16.20 (-3.07, 43.49)	217 (-41, 583)	219.94 (-41.17, 607.87)	477.95 (-89.47, 1321)	26.31 (-4.93, 72.72)	57.18 (-10.70, 158)
Calcium Suppl.	50%	0.92 (-0.22, 2.87)	22.19 (-7.79, 105.07)	297 (-104, 1408)	306.22 (-93.94, 1420.41)	665.44 (-204.15, 3087)	65.94 (-20.23, 305.86)	143.29 (-43.96, 665)
	90%	1.65 (-0.40, 5.16)	39.95 (-14.02, 189.13)	535 (-188, 2534)	551.20 (-169.10, 2556.74)	1197.80 (-367.46, 5556)	65.94 (-20.23, 305.86)	143.29 (-43.96, 665)
Multiple Micronutrient Suppl.	50%	0.59 (0.25, 0.87)	14.36 (2.81, 31.45)	192 (38, 421)	196.71 (41.64, 434.23)	427.47 (90.49, 944)	42.36 (8.97, 93.50)	92.05 (19.49, 203)
	90%	1.20 (0.46, 1.82)	29.32 (5.64, 64.87)	393 (76, 869)	393.46 (73.61, 893.21)	855.01 (159.96, 1941)	47.07 (8.81, 106.85)	102.28 (19.14, 232)
Balanced Protein Suppl.	50%	0.01 (-0.01, 0.03)	0.22 (-0.22, 0.82)	3 (-3, 11)	3.00 (-3.00, 11.14)	6.51 (-6.53, 24.22)	33.29 (-32.38, 103.58)	72.35 (-70.37, 225)
	90%	0.02 (-0.01, 0.05)	0.40 (-0.40, 1.48)	5 (-5, 20)	5.39 (-5.41, 20.06)	11.72 (-11.75, 43.59)	33.29 (-32.38, 103.58)	72.35 (-70.37, 225)

References for Data Inputs

[1] Blencowe H, Krasevec J, Onis M De, et al. Articles National , regional , and worldwide estimates of low birthweight in 2015 , with trends from 2000: a systematic analysis. Lancet Glob Heal. 2019;(18):1-12.
[2] Chawanpaiboon S, Vogel JP, Moller AB, et al. Global, regional, and national estimates of levels of preterm birth in 2014: a systematic review and modelling analysis. Lancet Glob Heal. 2019;7(1):e37-e46.
[3] United National Population Division World Population Prospects 2019.
[4] Fink G, Peet E, Danaei G, et al. Schooling and wage income losses due to early-childhood growth faltering in developing countries: National, regional, and global estimates. Am J Clin Nutr. 2016;104(1):104-112.
[5] Country specific annual wage data from World Indicators Database. Average yearly wage was estimated to be 2/3 of the gross domestic product in 2010 constant US dollars and 2011 International dollars, adjusted for purchasing power parity.
[6] NCD Risk Factor Collaboration. Trends in adult body-mass index in 200 countries from 1975 to 2014: a pooled analysis of 1698 population-based measurement studies with 19·2 million participants. Lancet. 2016;387(10026):1377-1396.
[7] Stevens GA, Finucane MM, De-Regil LM, et al. Global, regional, and national trends in haemoglobin concentration and prevalence of total and severe anaemia in children and pregnant and non-pregnant women for 1995-2011: A systematic analysis of population-representative data. Lancet Glob Heal. 2013;1(1):16-25.
[8] Coverage of iron-folic acid supplementation abstracted from the most recent Demographic Health Survey or imputed based on sub-regional average. Indicator used: % women in the past five years who took iron tablets or syrup for >90 days.

Rwanda

Region: Sub-Saharan Africa**; Sub-region:** Eastern Sub-Saharan Africa

Low birthweight prevalence[1]: 7.9% (95% CI: 6.2, 10.1)

Preterm birth prevalence[2]: 12.0% (95% CI: 8.6, 16.7)

Number of births[3]: 1,951,000

Returns to education[4]: 11.3% (95% CI: 9.7, 12.9)

GDP per capita 2010 US$ (estimated annual wage)[5]: $732 ($488/year)

GDP per capita 2011 International $ (estimated annual wage)[5]: $1774 ($1183/year)

Prevalence of low BMI[6]: 7.6% (95% CI: 3.6, 13.1)

Prevalence of anemia[7]: 20.0% (95% CI: 15.5, 25.5)

Baseline coverage of IFA[8]: 3.4%

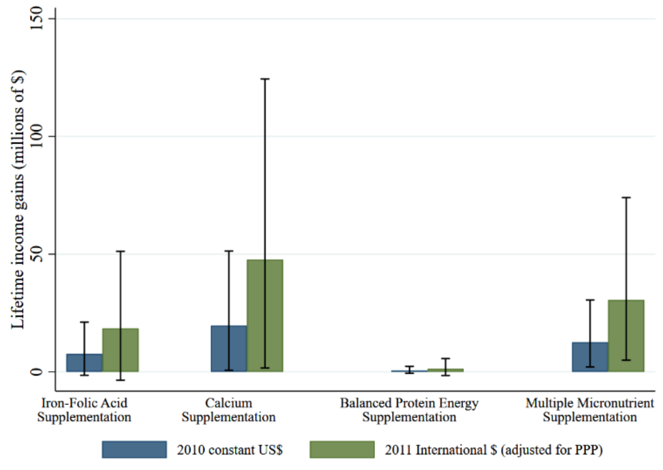

Figure: Benefits by birth cohort size for estimated gains in lifetime wages attributable to scaling up nutrition interventions to 90% target coverage

Table: Impact of maternal prenatal nutrition intervention on human capital and labour market outcomes, through improvements in low birthweight or preterm birth and schooling in Rwanda.

Intervention	Target Coverage (%)	Absolute reduction in birth outcome (%)	Benefits by cohorts: School years gained (in 1000 s)	No. of additional students completing secondary school	Benefits by cohorts: Lifetime wages		Returns in lifetime earnings per child born to a targeted pregnant woman	
					in US $ millions	in International $ millions	in US $	in International $
Iron/Folic Acid Suppl.	90%	1.08 (-0.23, 2.31)	5.17 (-0.90, 14.16)	54 (-9, 147)	7.63 (-1.45, 21.12)	18.50 (-3.52, 51.19)	4.35 (-0.83, 12.03)	10.54 (-2.00, 29.15)
Calcium Suppl.	50%	1.39 (0.28, 2.62)	7.41 (0.26, 19.09)	77 (3, 199)	10.94 (0.38, 28.52)	26.51 (0.92, 69.13)	11.21 (0.39, 29.23)	27.17 (0.95, 70.86)
	90%	2.50 (0.51, 4.71)	13.34 (0.47, 34.36)	139 (5, 357)	19.68 (0.69, 51.33)	47.72 (1.66, 124.43)	11.21 (0.39, 29.23)	27.17 (0.95, 70.86)
Multiple Micronutrient Suppl.	50%	0.95 (0.32, 1.60)	4.64 (0.80, 10.58)	48 (8, 110)	6.85 (1.13, 16.53)	16.61 (2.75, 40.08)	7.02 (1.16, 16.95)	17.02 (2.82, 41.09)
	90%	1.74 (0.57, 2.96)	8.49 (1.42, 19.43)	88 (15, 202)	12.60 (2.06, 30.53)	30.55 (4.99, 74.01)	7.18 (1.17, 17.39)	17.40 (2.84, 42.15)
Balanced Protein Suppl.	50%	0.05 (-0.04, 0.14)	0.21 (-0.23, 0.86)	2 (-2, 9)	0.31 (-0.35, 1.30)	0.76 (-0.85, 3.15)	4.60 (-4.42, 14.44)	11.15 (-10.71, 35.01)
	90%	0.08 (-0.07, 0.25)	0.38 (-0.42, 1.55)	4 (-4, 16)	0.57 (-0.63, 2.34)	1.37 (-1.52, 5.67)	4.60 (-4.42, 14.44)	11.15 (-10.71, 35.01)

References for Data Inputs

[1] Blencowe H, Krasevec J, Onis M De, et al. Articles National , regional , and worldwide estimates of low birthweight in 2015 , with trends from 2000: a systematic analysis. Lancet Glob Heal. 2019;(18):1-12.
[2] Chawanpaiboon S, Vogel JP, Moller AB, et al. Global, regional, and national estimates of levels of preterm birth in 2014: a systematic review and modelling analysis. Lancet Glob Heal. 2019;7(1):e37-e46.
[3] United National Population Division World Population Prospects 2019.
[4] Fink G, Peet E, Danaei G, et al. Schooling and wage income losses due to early-childhood growth faltering in developing countries: National, regional, and global estimates. Am J Clin Nutr. 2016;104(1):104-112.
[5] Country specific annual wage data from World Indicators Database. Average yearly wage was estimated to be 2/3 of the gross domestic product in 2010 constant US dollars and 2011 International dollars, adjusted for purchasing power parity.
[6] NCD Risk Factor Collaboration. Trends in adult body-mass index in 200 countries from 1975 to 2014: a pooled analysis of 1698 population-based measurement studies with 19·2 million participants. Lancet. 2016;387(10026):1377-1396.
[7] Stevens GA, Finucane MM, De-Regil LM, et al. Global, regional, and national trends in haemoglobin concentration and prevalence of total and severe anaemia in children and pregnant and non-pregnant women for 1995-2011: A systematic analysis of population-representative data. Lancet Glob Heal. 2013;1(1):16-25.
[8] Coverage of iron-folic acid supplementation abstracted from the most recent Demographic Health Survey or imputed based on sub-regional average. Indicator used: % women in the past five years who took iron tablets or syrup for >90 days.

Samoa

Region: Southeast Asia, East Asia, and Oceania**; Sub-region:** Oceania

Low birthweight prevalence[1]: 1.2% (95% CI: -1.5, 3.9)

Preterm birth prevalence[2]: 10.0% (95% CI: 7.9, 12.7)

Number of births[3]: 24,000

Returns to education[4]: 6.1% (95% CI: 2.7, 9.6)

GDP per capita 2010 US$ (estimated annual wage)[5]: $3560 ($2373/year)

GDP per capita 2011 International $ (estimated annual wage)[5]: $5567 ($3711/year)

Prevalence of low BMI[6]: 0.4% (95% CI: 0.1, 0.9)

Prevalence of anemia[7]: 26.5% (95% CI: 9.2, 52.3)

Baseline coverage of IFA[8]: 44.2%

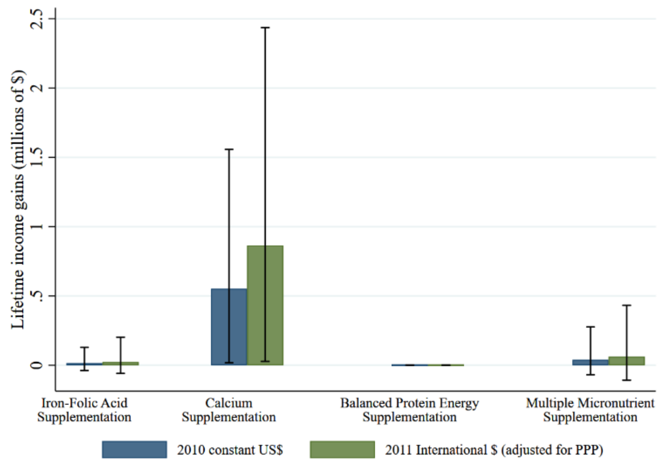

Figure: Benefits by birth cohort size for estimated gains in lifetime wages attributable to scaling up nutrition interventions to 90% target coverage

Table: Impact of maternal prenatal nutrition intervention on human capital and labour market outcomes, through improvements in low birthweight or preterm birth and schooling in Samoa.

Intervention	Target Coverage (%)	Absolute reduction in birth outcome (%)	Benefits by cohorts: School years gained (in 1000 s)	No. of additional students completing secondary school	Benefits by cohorts: Lifetime wages		Returns in lifetime earnings per child born to a targeted pregnant woman	
					in US $ millions	in International $ millions	in US $	in International $
Iron/Folic Acid Suppl.	90%	0.07 (-0.13, 0.43)	0.00 (-0.01, 0.03)	0 (-0, 1)	0.01 (-0.04, 0.13)	0.02 (-0.06, 0.20)	0.69 (-1.73, 5.99)	1.08 (-2.71, 9.37)
Calcium Suppl.	50%	1.17 (0.26, 2.06)	0.08 (0.00, 0.20)	2 (0, 5)	0.31 (0.01, 0.87)	0.48 (0.02, 1.35)	25.60 (0.84, 72.12)	40.02 (1.31, 112.77)
	90%	2.11 (0.46, 3.72)	0.14 (0.00, 0.36)	3 (0, 8)	0.55 (0.02, 1.56)	0.86 (0.03, 2.44)	25.60 (0.84, 72.12)	40.02 (1.31, 112.77)
Multiple Micronutrient Suppl.	50%	0.07 (-0.09, 0.28)	0.00 (-0.01, 0.02)	0 (-0, 1)	0.01 (-0.02, 0.10)	0.02 (-0.04, 0.16)	1.19 (-2.06, 8.41)	1.86 (-3.22, 13.16)
	90%	0.19 (-0.24, 0.74)	0.01 (-0.02, 0.07)	0 (-0, 2)	0.04 (-0.07, 0.28)	0.06 (-0.11, 0.43)	1.86 (-3.18, 12.79)	2.91 (-4.97, 20.00)
Balanced Protein Suppl.	50%	0.00 (-0.00, 0.00)	0.00 (-0.00, 0.00)	0 (-0, 0)	0.00 (-0.00, 0.00)	0.00 (-0.00, 0.00)	1.18 (-3.25, 12.14)	1.85 (-5.09, 18.99)
	90%	0.00 (-0.00, 0.00)	0.00 (-0.00, 0.00)	0 (-0, 0)	0.00 (-0.00, 0.00)	0.00 (-0.00, 0.00)	1.18 (-3.25, 12.14)	1.85 (-5.09, 18.99)

References for Data Inputs

[1] Blencowe H, Krasevec J, Onis M De, et al. Articles National , regional , and worldwide estimates of low birthweight in 2015 , with trends from 2000: a systematic analysis. Lancet Glob Heal. 2019;(18):1-12.
[2] Chawanpaiboon S, Vogel JP, Moller AB, et al. Global, regional, and national estimates of levels of preterm birth in 2014: a systematic review and modelling analysis. Lancet Glob Heal. 2019;7(1):e37-e46.
[3] United National Population Division World Population Prospects 2019.
[4] Fink G, Peet E, Danaei G, et al. Schooling and wage income losses due to early-childhood growth faltering in developing countries: National, regional, and global estimates. Am J Clin Nutr. 2016;104(1):104-112.
[5] Country specific annual wage data from World Indicators Database. Average yearly wage was estimated to be 2/3 of the gross domestic product in 2010 constant US dollars and 2011 International dollars, adjusted for purchasing power parity.
[6] NCD Risk Factor Collaboration. Trends in adult body-mass index in 200 countries from 1975 to 2014: a pooled analysis of 1698 population-based measurement studies with 19·2 million participants. Lancet. 2016;387(10026):1377-1396.
[7] Stevens GA, Finucane MM, De-Regil LM, et al. Global, regional, and national trends in haemoglobin concentration and prevalence of total and severe anaemia in children and pregnant and non-pregnant women for 1995-2011: A systematic analysis of population-representative data. Lancet Glob Heal. 2013;1(1):16-25.
[8] Coverage of iron-folic acid supplementation abstracted from the most recent Demographic Health Survey or imputed based on sub-regional average. Indicator used: % women in the past five years who took iron tablets or syrup for >90 days.

Sao Tome and Principe

Region: Sub-Saharan Africa**; Sub-region:** Western Sub-Saharan Africa

Low birthweight prevalence[1]: 6.6% (95% CI: 5.0, 8.6)

Preterm birth prevalence[2]: 12.0% (95% CI: 8.6, 16.7)

Number of births[3]: 33,000

Returns to education[4]: 6.3% (95% CI: 4.5, 8.1)

GDP per capita 2010 US$ (estimated annual wage)[5]: $1236 ($824/year)

GDP per capita 2011 International $ (estimated annual wage)[5]: $2890 ($1926/year)

Prevalence of low BMI[6]: 7.4% (95% CI: 3.2, 13.6)

Prevalence of anemia[7]: 48.3% (95% CI: 36.7, 60.4)

Baseline coverage of IFA[8]: 36.8%

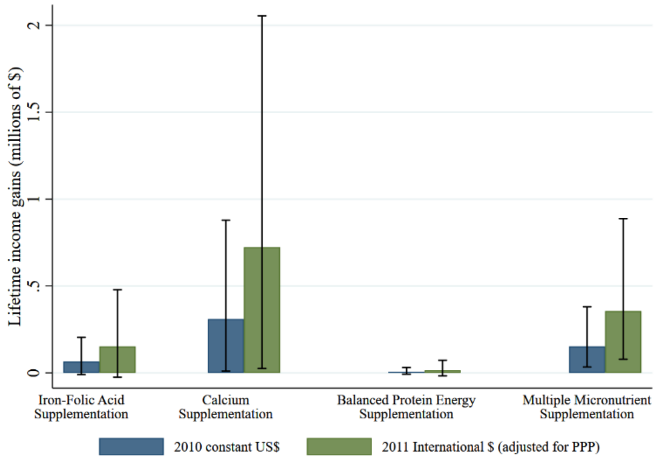

Figure: Benefits by birth cohort size for estimated gains in lifetime wages attributable to scaling up nutrition interventions to 90% target coverage

Table: Impact of maternal prenatal nutrition intervention on human capital and labour market outcomes, through improvements in low birthweight or preterm birth and schooling in Sao Tome and Principe.

Intervention	Target Coverage (%)	Absolute reduction in birth outcome (%)	Benefits by cohorts: School years gained (in 1000 s)	No. of additional students completing secondary school	Benefits by cohorts: Lifetime wages		Returns in lifetime earnings per child born to a targeted pregnant woman	
					in US $ millions	in International $ millions	in US $	in International $
Iron/Folic Acid Suppl.	90%	0.59 (-0.11, 1.30)	0.05 (-0.01, 0.14)	4 (-1, 12)	0.06 (-0.01, 0.20)	0.15 (-0.02, 0.48)	2.19 (-0.36, 6.90)	5.12 (-0.83, 16.12)
Calcium Suppl.	50%	1.42 (0.28, 2.61)	0.13 (0.00, 0.33)	11 (0, 29)	0.17 (0.01, 0.49)	0.40 (0.01, 1.14)	10.42 (0.36, 29.60)	24.36 (0.83, 69.20)
	90%	2.56 (0.51, 4.69)	0.23 (0.01, 0.60)	20 (1, 51)	0.31 (0.01, 0.88)	0.72 (0.02, 2.06)	10.42 (0.36, 29.60)	24.36 (0.83, 69.20)
Multiple Micronutrient Suppl.	50%	0.54 (0.31, 0.79)	0.05 (0.01, 0.10)	4 (1, 9)	0.07 (0.01, 0.16)	0.15 (0.03, 0.36)	3.98 (0.88, 9.46)	9.31 (2.05, 22.11)
	90%	1.30 (0.61, 2.02)	0.11 (0.02, 0.26)	10 (2, 22)	0.15 (0.03, 0.38)	0.36 (0.08, 0.89)	5.12 (1.13, 12.79)	11.97 (2.63, 29.90)
Balanced Protein Suppl.	50%	0.04 (-0.03, 0.11)	0.00 (-0.00, 0.01)	0 (-0, 1)	0.00 (-0.00, 0.02)	0.01 (-0.01, 0.04)	3.52 (-3.75, 12.24)	8.22 (-8.77, 28.63)
	90%	0.06 (-0.05, 0.20)	0.00 (-0.01, 0.02)	0 (-0, 2)	0.01 (-0.01, 0.03)	0.02 (-0.02, 0.07)	3.52 (-3.75, 12.24)	8.22 (-8.77, 28.63)

References for Data Inputs

[1] Blencowe H, Krasevec J, Onis M De, et al. Articles National , regional , and worldwide estimates of low birthweight in 2015 , with trends from 2000: a systematic analysis. Lancet Glob Heal. 2019;(18):1-12.
[2] Chawanpaiboon S, Vogel JP, Moller AB, et al. Global, regional, and national estimates of levels of preterm birth in 2014: a systematic review and modelling analysis. Lancet Glob Heal. 2019;7(1):e37-e46.
[3] United National Population Division World Population Prospects 2019.
[4] Fink G, Peet E, Danaei G, et al. Schooling and wage income losses due to early-childhood growth faltering in developing countries: National, regional, and global estimates. Am J Clin Nutr. 2016;104(1):104-112.
[5] Country specific annual wage data from World Indicators Database. Average yearly wage was estimated to be 2/3 of the gross domestic product in 2010 constant US dollars and 2011 International dollars, adjusted for purchasing power parity.
[6] NCD Risk Factor Collaboration. Trends in adult body-mass index in 200 countries from 1975 to 2014: a pooled analysis of 1698 population-based measurement studies with 19·2 million participants. Lancet. 2016;387(10026):1377-1396.
[7] Stevens GA, Finucane MM, De-Regil LM, et al. Global, regional, and national trends in haemoglobin concentration and prevalence of total and severe anaemia in children and pregnant and non-pregnant women for 1995-2011: A systematic analysis of population-representative data. Lancet Glob Heal. 2013;1(1):16-25.
[8] Coverage of iron-folic acid supplementation abstracted from the most recent Demographic Health Survey or imputed based on sub-regional average. Indicator used: % women in the past five years who took iron tablets or syrup for >90 days.

Senegal

Region: Sub-Saharan Africa**; Sub-region:** Western Sub-Saharan Africa

Low birthweight prevalence[1]: 18.5% (95% CI: 14.1, 24.0)

Preterm birth prevalence[2]: 12.0% (95% CI: 8.6, 16.7)

Number of births[3]: 2,720,000

Returns to education[4]: 6.3% (95% CI: 4.5, 8.1)

GDP per capita 2010 US$ (estimated annual wage)[5]: $1383 ($922/year)

GDP per capita 2011 International $ (estimated annual wage)[5]: $3002 ($2001/year)

Prevalence of low BMI[6]: 10.4% (95% CI: 5.3, 17.2)

Prevalence of anemia[7]: 63.8% (95% CI: 58.0, 68.6)

Baseline coverage of IFA[8]: 63.1%

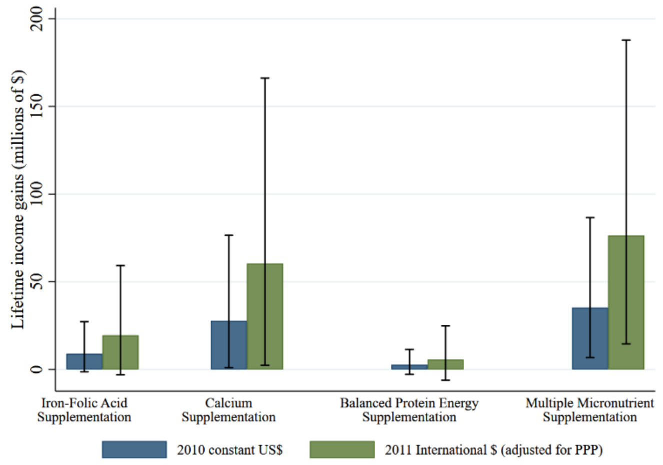

Figure: Benefits by birth cohort size for estimated gains in lifetime wages attributable to scaling up nutrition interventions to 90% target coverage

Table: Impact of maternal prenatal nutrition intervention on human capital and labour market outcomes, through improvements in low birthweight or preterm birth and schooling in Senegal.

Intervention	Target Coverage (%)	Absolute reduction in birth outcome (%)	Benefits by cohorts: School years gained (in 1000 s)	No. of additional students completing secondary school	Benefits by cohorts: Lifetime wages		Returns in lifetime earnings per child born to a targeted pregnant woman	
					in US $ millions	in International $ millions	in US $	in International $
Iron/Folic Acid Suppl.	90%	0.88 (-0.14, 2.01)	5.89 (-0.92, 17.47)	163 (-25, 482)	8.95 (-1.41, 27.28)	19.42 (-3.05, 59.20)	3.65 (-0.57, 11.14)	7.93 (-1.25, 24.18)
Calcium Suppl.	50%	1.38 (0.30, 2.64)	10.11 (0.34, 27.01)	279 (9, 745)	15.46 (0.58, 42.52)	33.55 (1.27, 92.28)	11.37 (0.43, 31.27)	24.67 (0.93, 67.86)
	90%	2.48 (0.54, 4.76)	18.20 (0.62, 48.62)	502 (17, 1342)	27.83 (1.05, 76.54)	60.39 (2.28, 166.11)	11.37 (0.43, 31.27)	24.67 (0.93, 67.86)
Multiple Micronutrient Suppl.	50%	1.40 (0.78, 2.06)	10.07 (1.79, 22.96)	278 (49, 634)	15.29 (2.81, 37.71)	33.18 (6.09, 81.83)	11.24 (2.06, 27.72)	24.40 (4.48, 60.17)
	90%	3.25 (1.85, 4.79)	23.08 (4.25, 52.20)	637 (117, 1441)	35.19 (6.68, 86.54)	76.38 (14.49, 187.81)	14.38 (2.73, 35.35)	31.20 (5.92, 76.72)
Balanced Protein Suppl.	50%	0.15 (-0.13, 0.46)	0.95 (-1.03, 3.85)	26 (-29, 106)	1.45 (-1.57, 6.36)	3.14 (-3.41, 13.81)	11.00 (-10.34, 37.45)	23.88 (-22.43, 81.29)
	90%	0.27 (-0.24, 0.82)	1.72 (-1.86, 6.92)	47 (-51, 191)	2.61 (-2.83, 11.45)	5.65 (-6.14, 24.86)	11.00 (-10.34, 37.45)	23.88 (-22.43, 81.29)

References for Data Inputs

[1] Blencowe H, Krasevec J, Onis M De, et al. Articles National , regional , and worldwide estimates of low birthweight in 2015 , with trends from 2000: a systematic analysis. Lancet Glob Heal. 2019;(18):1-12.

[2] Chawanpaiboon S, Vogel JP, Moller AB, et al. Global, regional, and national estimates of levels of preterm birth in 2014: a systematic review and modelling analysis. Lancet Glob Heal. 2019;7(1):e37-e46.

[3] United National Population Division World Population Prospects 2019.

[4] Fink G, Peet E, Danaei G, et al. Schooling and wage income losses due to early-childhood growth faltering in developing countries: National, regional, and global estimates. Am J Clin Nutr. 2016;104(1):104-112.

[5] Country specific annual wage data from World Indicators Database. Average yearly wage was estimated to be 2/3 of the gross domestic product in 2010 constant US dollars and 2011 International dollars, adjusted for purchasing power parity.

[6] NCD Risk Factor Collaboration. Trends in adult body-mass index in 200 countries from 1975 to 2014: a pooled analysis of 1698 population-based measurement studies with 19·2 million participants. Lancet. 2016;387(10026):1377-1396.

[7] Stevens GA, Finucane MM, De-Regil LM, et al. Global, regional, and national trends in haemoglobin concentration and prevalence of total and severe anaemia in children and pregnant and non-pregnant women for 1995-2011: A systematic analysis of population-representative data. Lancet Glob Heal. 2013;1(1):16-25.

[8] Coverage of iron-folic acid supplementation abstracted from the most recent Demographic Health Survey or imputed based on sub-regional average. Indicator used: % women in the past five years who took iron tablets or syrup for >90 days.

Serbia

Region: Central Europe, Eastern Europe, Central Asia**; Sub-region:** Central Europe

Low birthweight prevalence[1]: 4.5% (95% CI: 3.6, 5.7)

Preterm birth prevalence[2]: 12.0% (95% CI: 2.1, 34.0)

Number of births[3]: 421,000

Returns to education[4]: 7.8% (95% CI: 7.4, 8.1)

GDP per capita 2010 US$ (estimated annual wage)[5]: $6155 ($4104/year)

GDP per capita 2011 International $ (estimated annual wage)[5]: $14346 ($9564/year)

Prevalence of low BMI[6]: 2.6% (95% CI: 1.2, 4.6)

Prevalence of anemia[7]: 27.0% (95% CI: 14.9, 48.3)

Baseline coverage of IFA[8]: 18.5%

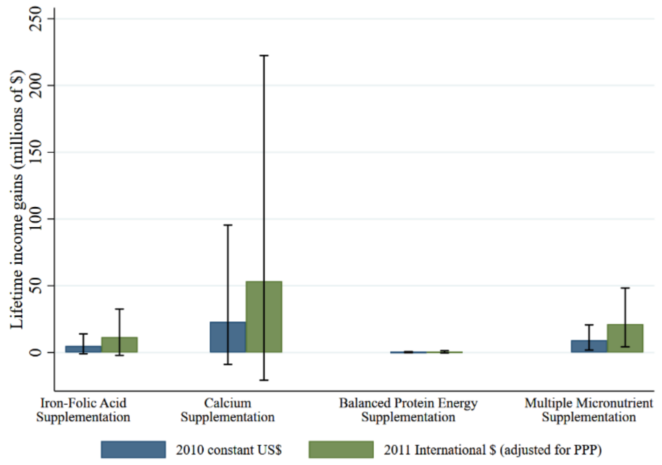

Figure: Benefits by birth cohort size for estimated gains in lifetime wages attributable to scaling up nutrition interventions to 90% target coverage

Table: Impact of maternal prenatal nutrition intervention on human capital and labour market outcomes, through improvements in low birthweight or preterm birth and schooling in Serbia.

Intervention	Target Coverage (%)	Absolute reduction in birth outcome (%)	Benefits by cohorts: School years gained (in 1000 s)	No. of additional students completing secondary school	Benefits by cohorts: Lifetime wages		Returns in lifetime earnings per child born to a targeted pregnant woman	
					in US $ millions	in International $ millions	in US $	in International $
Iron/Folic Acid Suppl.	90%	0.53 (-0.10, 1.09)	0.57 (-0.11, 1.63)	25 (-5, 72)	4.94 (-0.90, 13.98)	11.50 (-2.10, 32.58)	13.03 (-2.38, 36.89)	30.36 (-5.54, 85.97)
Calcium Suppl.	50%	1.27 (-0.44, 4.06)	1.48 (-0.59, 6.17)	66 (-26, 273)	12.74 (-4.94, 53.03)	29.69 (-11.50, 123.60)	60.52 (-23.45, 251.94)	141.04 (-54.65, 587.16)
	90%	2.28 (-0.78, 7.32)	2.67 (-1.05, 11.10)	118 (-47, 491)	22.93 (-8.88, 95.46)	53.44 (-20.71, 222.48)	60.52 (-23.45, 251.94)	141.04 (-54.65, 587.16)
Multiple Micronutrient Suppl.	50%	0.46 (0.20, 0.73)	0.52 (0.11, 1.17)	23 (5, 52)	4.47 (0.92, 10.02)	10.42 (2.15, 23.34)	21.24 (4.39, 47.58)	49.51 (10.23, 110.90)
	90%	0.93 (0.36, 1.54)	1.06 (0.21, 2.40)	47 (10, 106)	9.13 (1.81, 20.73)	21.29 (4.22, 48.31)	24.10 (4.77, 54.71)	56.18 (11.13, 127.51)
Balanced Protein Suppl.	50%	0.01 (-0.01, 0.03)	0.01 (-0.01, 0.04)	0 (-0, 2)	0.08 (-0.09, 0.33)	0.19 (-0.21, 0.78)	16.34 (-14.95, 50.45)	38.08 (-34.84, 117.58)
	90%	0.02 (-0.01, 0.05)	0.02 (-0.02, 0.07)	1 (-1, 3)	0.14 (-0.16, 0.60)	0.33 (-0.37, 1.40)	16.34 (-14.95, 50.45)	38.08 (-34.84, 117.58)

References for Data Inputs

[1] Blencowe H, Krasevec J, Onis M De, et al. Articles National , regional , and worldwide estimates of low birthweight in 2015 , with trends from 2000: a systematic analysis. Lancet Glob Heal. 2019;(18):1-12.
[2] Chawanpaiboon S, Vogel JP, Moller AB, et al. Global, regional, and national estimates of levels of preterm birth in 2014: a systematic review and modelling analysis. Lancet Glob Heal. 2019;7(1):e37-e46.
[3] United National Population Division World Population Prospects 2019.
[4] Fink G, Peet E, Danaei G, et al. Schooling and wage income losses due to early-childhood growth faltering in developing countries: National, regional, and global estimates. Am J Clin Nutr. 2016;104(1):104-112.
[5] Country specific annual wage data from World Indicators Database. Average yearly wage was estimated to be 2/3 of the gross domestic product in 2010 constant US dollars and 2011 International dollars, adjusted for purchasing power parity.
[6] NCD Risk Factor Collaboration. Trends in adult body-mass index in 200 countries from 1975 to 2014: a pooled analysis of 1698 population-based measurement studies with 19·2 million participants. Lancet. 2016;387(10026):1377-1396.
[7] Stevens GA, Finucane MM, De-Regil LM, et al. Global, regional, and national trends in haemoglobin concentration and prevalence of total and severe anaemia in children and pregnant and non-pregnant women for 1995-2011: A systematic analysis of population-representative data. Lancet Glob Heal. 2013;1(1):16-25.
[8] Coverage of iron-folic acid supplementation abstracted from the most recent Demographic Health Survey or imputed based on sub-regional average. Indicator used: % women in the past five years who took iron tablets or syrup for >90 days.

Sierra Leone

Region: Sub-Saharan Africa**; Sub-region:** Western Sub-Saharan Africa

Low birthweight prevalence[1]: 14.4% (95% CI: 11.4, 18.1)

Preterm birth prevalence[2]: 12.0% (95% CI: 8.6, 16.7)

Number of births[3]: 1,277,000

Returns to education[4]: 6.3% (95% CI: 4.5, 8.1)

GDP per capita 2010 US$ (estimated annual wage)[5]: $441 ($294/year)

GDP per capita 2011 International $ (estimated annual wage)[5]: $1326 ($884/year)

Prevalence of low BMI[6]: 9.5% (95% CI: 4.6, 15.8)

Prevalence of anemia[7]: 46.5% (95% CI: 34.2, 60.2)

Baseline coverage of IFA[8]: 30.0%

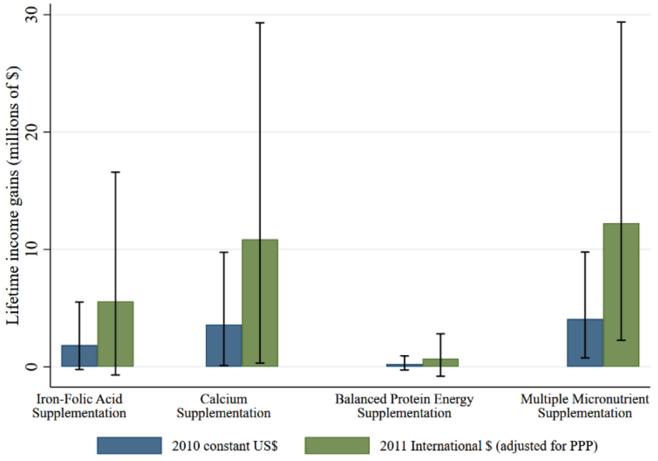

Figure: Benefits by birth cohort size for estimated gains in lifetime wages attributable to scaling up nutrition interventions to 90% target coverage

Table: Impact of maternal prenatal nutrition intervention on human capital and labour market outcomes, through improvements in low birthweight or preterm birth and schooling in Sierra Leone.

Intervention	Target Coverage (%)	Absolute reduction in birth outcome (%)	Benefits by cohorts: School years gained (in 1000 s)	No. of additional students completing secondary school	Benefits by cohorts: Lifetime wages		Returns in lifetime earnings per child born to a targeted pregnant woman	
					in US $ millions	in International $ millions	in US $	in International $
Iron/Folic Acid Suppl.	90%	1.44 (-0.23, 2.97)	3.79 (-0.55, 10.99)	227 (-33, 657)	1.85 (-0.23, 5.52)	5.58 (-0.70, 16.58)	1.61 (-0.20, 4.80)	4.85 (-0.61, 14.43)
Calcium Suppl.	50%	1.40 (0.30, 2.62)	4.08 (0.11, 10.95)	244 (7, 655)	2.01 (0.06, 5.42)	6.03 (0.17, 16.28)	3.14 (0.09, 8.48)	9.44 (0.27, 25.50)
	90%	2.51 (0.53, 4.72)	7.35 (0.20, 19.70)	439 (12, 1178)	3.61 (0.10, 9.75)	10.85 (0.31, 29.31)	3.14 (0.09, 8.48)	9.44 (0.27, 25.50)
Multiple Micronutrient Suppl.	50%	1.34 (0.73, 1.93)	3.81 (0.77, 8.20)	228 (46, 490)	1.85 (0.39, 4.20)	5.57 (1.16, 12.62)	2.90 (0.61, 6.57)	8.73 (1.82, 19.76)
	90%	2.98 (1.37, 4.53)	8.36 (1.58, 18.96)	500 (95, 1134)	4.07 (0.75, 9.77)	12.25 (2.26, 29.37)	3.54 (0.65, 8.50)	10.65 (1.97, 25.55)
Balanced Protein Suppl.	50%	0.11 (-0.09, 0.31)	0.27 (-0.28, 1.05)	16 (-17, 63)	0.13 (-0.15, 0.52)	0.39 (-0.44, 1.56)	2.29 (-2.22, 7.79)	6.89 (-6.68, 23.43)
	90%	0.19 (-0.16, 0.55)	0.49 (-0.51, 1.89)	29 (-30, 113)	0.23 (-0.27, 0.93)	0.70 (-0.80, 2.81)	2.29 (-2.22, 7.79)	6.89 (-6.68, 23.43)

References for Data Inputs

[1] Blencowe H, Krasevec J, Onis M De, et al. Articles National , regional , and worldwide estimates of low birthweight in 2015 , with trends from 2000: a systematic analysis. Lancet Glob Heal. 2019;(18):1-12.
[2] Chawanpaiboon S, Vogel JP, Moller AB, et al. Global, regional, and national estimates of levels of preterm birth in 2014: a systematic review and modelling analysis. Lancet Glob Heal. 2019;7(1):e37-e46.
[3] United National Population Division World Population Prospects 2019.
[4] Fink G, Peet E, Danaei G, et al. Schooling and wage income losses due to early-childhood growth faltering in developing countries: National, regional, and global estimates. Am J Clin Nutr. 2016;104(1):104-112.
[5] Country specific annual wage data from World Indicators Database. Average yearly wage was estimated to be 2/3 of the gross domestic product in 2010 constant US dollars and 2011 International dollars, adjusted for purchasing power parity.
[6] NCD Risk Factor Collaboration. Trends in adult body-mass index in 200 countries from 1975 to 2014: a pooled analysis of 1698 population-based measurement studies with 19·2 million participants. Lancet. 2016;387(10026):1377-1396.
[7] Stevens GA, Finucane MM, De-Regil LM, et al. Global, regional, and national trends in haemoglobin concentration and prevalence of total and severe anaemia in children and pregnant and non-pregnant women for 1995-2011: A systematic analysis of population-representative data. Lancet Glob Heal. 2013;1(1):16-25.
[8] Coverage of iron-folic acid supplementation abstracted from the most recent Demographic Health Survey or imputed based on sub-regional average. Indicator used: % women in the past five years who took iron tablets or syrup for >90 days.

Solomon Islands

Region: Southeast Asia, East Asia, and Oceania**; Sub-region:** Oceania

Low birthweight prevalence[1]: 12.5% (95% CI: 9.8, 15.2)

Preterm birth prevalence[2]: 10.0% (95% CI: 7.9, 12.7)

Number of births[3]: 105,000

Returns to education[4]: 6.1% (95% CI: 2.7, 9.6)

GDP per capita 2010 US$ (estimated annual wage)[5]: $1444 ($963/year)

GDP per capita 2011 International $ (estimated annual wage)[5]: $2094 ($1396/year)

Prevalence of low BMI[6]: 2.0% (95% CI: 0.8, 4.1)

Prevalence of anemia[7]: 33.1% (95% CI: 14.6, 59.4)

Baseline coverage of IFA[8]: 44.2%

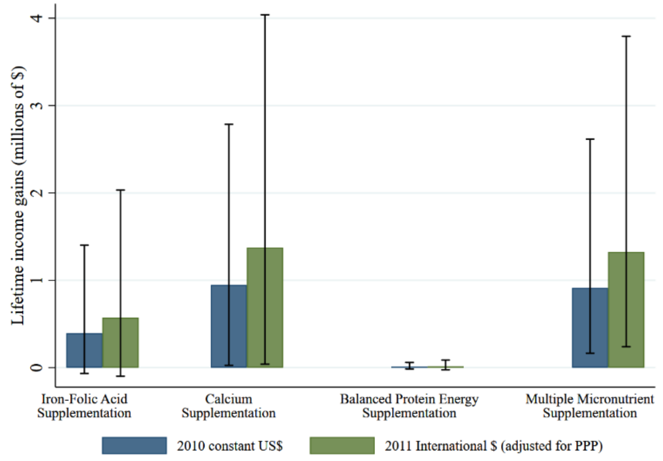

Figure: Benefits by birth cohort size for estimated gains in lifetime wages attributable to scaling up nutrition interventions to 90% target coverage

Table: Impact of maternal prenatal nutrition intervention on human capital and labour market outcomes, through improvements in low birthweight or preterm birth and schooling in Solomon Islands.

Intervention	Target Coverage (%)	Absolute reduction in birth outcome (%)	Benefits by cohorts: School years gained (in 1000 s)	No. of additional students completing secondary school	Benefits by cohorts: Lifetime wages		Returns in lifetime earnings per child born to a targeted pregnant woman	
					in US $ millions	in International $ millions	in US $	in International $
Iron/Folic Acid Suppl.	90%	0.97 (-0.14, 2.10)	0.26 (-0.04, 0.76)	6 (-1, 17)	0.39 (-0.07, 1.40)	0.57 (-0.10, 2.03)	4.17 (-0.71, 14.84)	6.05 (-1.03, 21.52)
Calcium Suppl.	50%	1.17 (0.25, 2.08)	0.35 (0.01, 0.86)	8 (0, 19)	0.53 (0.02, 1.55)	0.76 (0.02, 2.24)	10.03 (0.29, 29.48)	14.54 (0.42, 42.75)
	90%	2.11 (0.44, 3.74)	0.62 (0.02, 1.55)	14 (0, 35)	0.95 (0.03, 2.79)	1.37 (0.04, 4.04)	10.03 (0.29, 29.48)	14.54 (0.42, 42.75)
Multiple Micronutrient Suppl.	50%	0.81 (0.45, 1.20)	0.23 (0.05, 0.51)	5 (1, 12)	0.35 (0.06, 0.92)	0.50 (0.09, 1.34)	6.62 (1.19, 17.60)	9.60 (1.73, 25.53)
	90%	2.22 (0.96, 3.50)	0.61 (0.12, 1.39)	14 (3, 31)	0.91 (0.17, 2.62)	1.32 (0.24, 3.79)	9.67 (1.75, 27.68)	14.02 (2.54, 40.14)
Balanced Protein Suppl.	50%	0.02 (-0.02, 0.06)	0.00 (-0.01, 0.02)	0 (-0, 0)	0.01 (-0.01, 0.03)	0.01 (-0.01, 0.05)	7.62 (-8.40, 26.36)	11.05 (-12.18, 38.23)
	90%	0.03 (-0.03, 0.11)	0.01 (-0.01, 0.04)	0 (-0, 1)	0.01 (-0.02, 0.06)	0.02 (-0.02, 0.09)	7.62 (-8.40, 26.36)	11.05 (-12.18, 38.23)

References for Data Inputs

[1] Blencowe H, Krasevec J, Onis M De, et al. Articles National , regional , and worldwide estimates of low birthweight in 2015 , with trends from 2000: a systematic analysis. Lancet Glob Heal. 2019;(18):1-12.

[2] Chawanpaiboon S, Vogel JP, Moller AB, et al. Global, regional, and national estimates of levels of preterm birth in 2014: a systematic review and modelling analysis. Lancet Glob Heal. 2019;7(1):e37-e46.

[3] United National Population Division World Population Prospects 2019.

[4] Fink G, Peet E, Danaei G, et al. Schooling and wage income losses due to early-childhood growth faltering in developing countries: National, regional, and global estimates. Am J Clin Nutr. 2016;104(1):104-112.

[5] Country specific annual wage data from World Indicators Database. Average yearly wage was estimated to be 2/3 of the gross domestic product in 2010 constant US dollars and 2011 International dollars, adjusted for purchasing power parity.

[6] NCD Risk Factor Collaboration. Trends in adult body-mass index in 200 countries from 1975 to 2014: a pooled analysis of 1698 population-based measurement studies with 19·2 million participants. Lancet. 2016;387(10026):1377-1396.

[7] Stevens GA, Finucane MM, De-Regil LM, et al. Global, regional, and national trends in haemoglobin concentration and prevalence of total and severe anaemia in children and pregnant and non-pregnant women for 1995-2011: A systematic analysis of population-representative data. Lancet Glob Heal. 2013;1(1):16-25.

[8] Coverage of iron-folic acid supplementation abstracted from the most recent Demographic Health Survey or imputed based on sub-regional average. Indicator used: % women in the past five years who took iron tablets or syrup for >90 days.

Somalia

Region: Sub-Saharan Africa**; Sub-region:** Eastern Sub-Saharan Africa

Low birthweight prevalence[1]: 11.2% (95% CI: 5.6, 16.8)

Preterm birth prevalence[2]: 12.0% (95% CI: 8.6, 16.7)

Number of births[3]: 3,108,000

Returns to education[4]: 11.3% (95% CI: 9.7, 12.9)

GDP per capita 2010 US$ (estimated annual wage)[5]: $269 ($179/year)

GDP per capita 2011 International $ (estimated annual wage)[5]: $544 ($363/year)

Prevalence of low BMI[6]: 9.3% (95% CI: 3.6, 17.8)

Prevalence of anemia[7]: 45.9% (95% CI: 33.5, 58.4)

Baseline coverage of IFA[8]: 19.2%

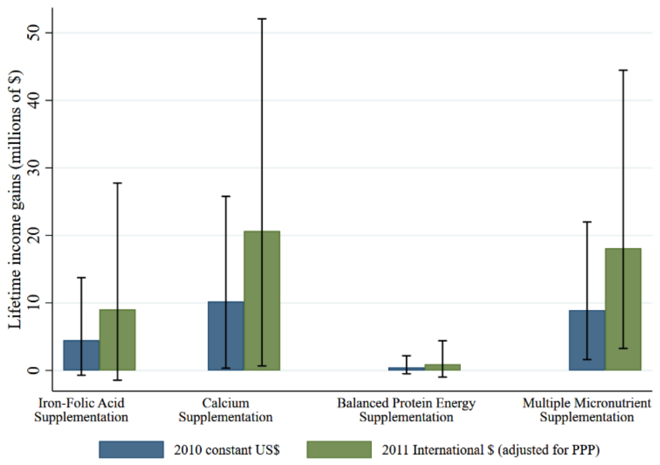

Figure: Benefits by birth cohort size for estimated gains in lifetime wages attributable to scaling up nutrition interventions to 90% target coverage

Table: Impact of maternal prenatal nutrition intervention on human capital and labour market outcomes, through improvements in low birthweight or preterm birth and schooling in Somalia.

Intervention	Target Coverage (%)	Absolute reduction in birth outcome (%)	Benefits by cohorts: School years gained (in 1000 s)	No. of additional students completing secondary school	Benefits by cohorts: Lifetime wages		Returns in lifetime earnings per child born to a targeted pregnant woman	
					in US $ millions	in International $ millions	in US $	in International $
Iron/Folic Acid Suppl.	90%	1.23 (-0.21, 3.08)	8.19 (-1.34, 25.10)	225 (-37, 688)	4.47 (-0.71, 13.73)	9.04 (-1.44, 27.76)	1.60 (-0.25, 4.91)	3.23 (-0.51, 9.92)
Calcium Suppl.	50%	1.41 (0.28, 2.63)	10.22 (0.36, 26.08)	280 (10, 715)	5.67 (0.19, 14.32)	11.47 (0.38, 28.94)	3.65 (0.12, 9.21)	7.38 (0.24, 18.62)
	90%	2.55 (0.50, 4.74)	18.40 (0.65, 46.94)	504 (18, 1287)	10.21 (0.33, 25.77)	20.64 (0.68, 52.09)	3.65 (0.12, 9.21)	7.38 (0.24, 18.62)
Multiple Micronutrient Suppl.	50%	1.19 (0.47, 2.12)	8.19 (1.49, 20.15)	225 (41, 553)	4.43 (0.83, 10.76)	8.96 (1.67, 21.74)	2.85 (0.53, 6.92)	5.77 (1.08, 13.99)
	90%	2.40 (0.89, 4.49)	16.52 (2.92, 41.72)	453 (80, 1144)	8.95 (1.61, 21.99)	18.09 (3.25, 44.45)	3.20 (0.58, 7.86)	6.47 (1.16, 15.89)
Balanced Protein Suppl.	50%	0.07 (-0.06, 0.28)	0.45 (-0.48, 2.10)	12 (-13, 58)	0.25 (-0.27, 1.20)	0.51 (-0.54, 2.44)	2.06 (-1.99, 6.97)	4.17 (-4.03, 14.09)
	90%	0.13 (-0.11, 0.50)	0.81 (-0.87, 3.79)	22 (-24, 104)	0.46 (-0.48, 2.17)	0.92 (-0.97, 4.38)	2.06 (-1.99, 6.97)	4.17 (-4.03, 14.09)

References for Data Inputs

[1] Blencowe H, Krasevec J, Onis M De, et al. Articles National , regional , and worldwide estimates of low birthweight in 2015 , with trends from 2000: a systematic analysis. Lancet Glob Heal. 2019;(18):1-12.

[2] Chawanpaiboon S, Vogel JP, Moller AB, et al. Global, regional, and national estimates of levels of preterm birth in 2014: a systematic review and modelling analysis. Lancet Glob Heal. 2019;7(1):e37-e46.

[3] United National Population Division World Population Prospects 2019.

[4] Fink G, Peet E, Danaei G, et al. Schooling and wage income losses due to early-childhood growth faltering in developing countries: National, regional, and global estimates. Am J Clin Nutr. 2016;104(1):104-112.

[5] Country specific annual wage data from World Indicators Database. Average yearly wage was estimated to be 2/3 of the gross domestic product in 2010 constant US dollars and 2011 International dollars, adjusted for purchasing power parity.

[6] NCD Risk Factor Collaboration. Trends in adult body-mass index in 200 countries from 1975 to 2014: a pooled analysis of 1698 population-based measurement studies with 19·2 million participants. Lancet. 2016;387(10026):1377-1396.

[7] Stevens GA, Finucane MM, De-Regil LM, et al. Global, regional, and national trends in haemoglobin concentration and prevalence of total and severe anaemia in children and pregnant and non-pregnant women for 1995-2011: A systematic analysis of population-representative data. Lancet Glob Heal. 2013;1(1):16-25.

[8] Coverage of iron-folic acid supplementation abstracted from the most recent Demographic Health Survey or imputed based on sub-regional average. Indicator used: % women in the past five years who took iron tablets or syrup for >90 days.

South Africa

Region: Sub-Saharan Africa**; Sub-region:** Southern Sub-Saharan Africa

Low birthweight prevalence[1]: 14.2% (95% CI: 11.1, 18.6)

Preterm birth prevalence[2]: 12.4% (95% CI: 8.6, 17.1)

Number of births[3]: 5,925,000

Returns to education[4]: 18.3% (95% CI: 13.2, 23.5)

GDP per capita 2010 US$ (estimated annual wage)[5]: $7557 ($5038/year)

GDP per capita 2011 International $ (estimated annual wage)[5]: $12346 ($8231/year)

Prevalence of low BMI[6]: 2.8% (95% CI: 1.6, 4.4)

Prevalence of anemia[7]: 30.0% (95% CI: 14.9, 52.3)

Baseline coverage of IFA[8]: 50.5%

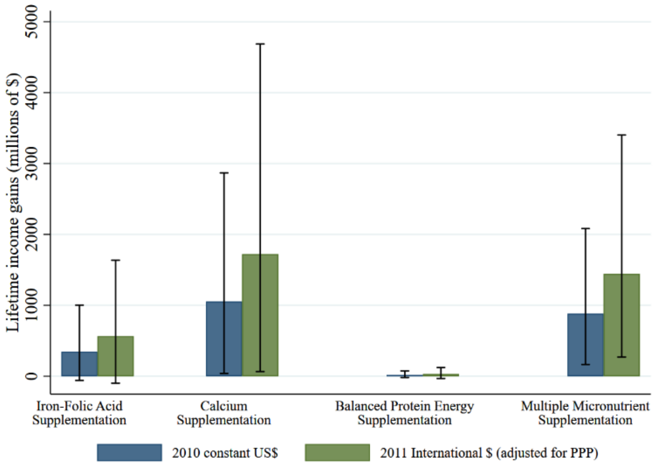

Figure: Benefits by birth cohort size for estimated gains in lifetime wages attributable to scaling up nutrition interventions to 90% target coverage

Table: Impact of maternal prenatal nutrition intervention on human capital and labour market outcomes, through improvements in low birthweight or preterm birth and schooling in South Africa.

Intervention	Target Coverage (%)	Absolute reduction in birth outcome (%)	Benefits by cohorts: School years gained (in 1000 s)	No. of additional students completing secondary school	Benefits by cohorts: Lifetime wages		Returns in lifetime earnings per child born to a targeted pregnant woman	
					in US $ millions	in International $ millions	in US $	in International $
Iron/Folic Acid Suppl.	90%	0.96 (-0.18, 2.16)	14.05 (-2.24, 39.64)	421 (-67, 1189)	346.22 (-59.74, 1001.70)	565.64 (-97.61, 1636.53)	64.93 (-11.20, 187.85)	106.07 (-18.30, 307)
Calcium Suppl.	50%	1.47 (0.31, 2.79)	23.53 (0.79, 62.44)	706 (24, 1873)	585.23 (22.12, 1593.15)	956.12 (36.14, 2602.82)	197.55 (7.47, 537.77)	322.74 (12.20, 879)
	90%	2.64 (0.56, 5.01)	42.35 (1.41, 112.39)	1270 (42, 3372)	1053.41 (39.82, 2867.67)	1721.02 (65.06, 4685.08)	197.55 (7.47, 537.77)	322.74 (12.20, 879)
Multiple Micronutrient Suppl.	50%	0.84 (0.38, 1.32)	12.98 (2.41, 29.29)	389 (72, 879)	321.12 (59.67, 768.44)	524.64 (97.48, 1255.44)	108.40 (20.14, 259.39)	177.09 (32.91, 424)
	90%	2.34 (1.05, 3.77)	36.03 (7.77, 79.98)	1081 (233, 2399)	883.38 (166.15, 2082.12)	1443.22 (271.45, 3401.68)	165.66 (31.16, 390.46)	270.65 (50.91, 638)
Balanced Protein Suppl.	50%	0.03 (-0.03, 0.09)	0.44 (-0.42, 1.66)	13 (-13, 50)	10.57 (-10.69, 42.04)	17.27 (-17.46, 68.68)	140.05 (-128.06, 440.14)	228.81 (-209.23, 719)
	90%	0.06 (-0.05, 0.17)	0.79 (-0.75, 2.98)	24 (-23, 89)	19.02 (-19.24, 75.67)	31.08 (-31.43, 123.62)	140.05 (-128.06, 440.14)	228.81 (-209.23, 719)

References for Data Inputs

[1] Blencowe H, Krasevec J, Onis M De, et al. Articles National , regional , and worldwide estimates of low birthweight in 2015 , with trends from 2000: a systematic analysis. Lancet Glob Heal. 2019;(18):1-12.
[2] Chawanpaiboon S, Vogel JP, Moller AB, et al. Global, regional, and national estimates of levels of preterm birth in 2014: a systematic review and modelling analysis. Lancet Glob Heal. 2019;7(1):e37-e46.
[3] United National Population Division World Population Prospects 2019.
[4] Fink G, Peet E, Danaei G, et al. Schooling and wage income losses due to early-childhood growth faltering in developing countries: National, regional, and global estimates. Am J Clin Nutr. 2016;104(1):104-112.
[5] Country specific annual wage data from World Indicators Database. Average yearly wage was estimated to be 2/3 of the gross domestic product in 2010 constant US dollars and 2011 International dollars, adjusted for purchasing power parity.
[6] NCD Risk Factor Collaboration. Trends in adult body-mass index in 200 countries from 1975 to 2014: a pooled analysis of 1698 population-based measurement studies with 19·2 million participants. Lancet. 2016;387(10026):1377-1396.
[7] Stevens GA, Finucane MM, De-Regil LM, et al. Global, regional, and national trends in haemoglobin concentration and prevalence of total and severe anaemia in children and pregnant and non-pregnant women for 1995-2011: A systematic analysis of population-representative data. Lancet Glob Heal. 2013;1(1):16-25.
[8] Coverage of iron-folic acid supplementation abstracted from the most recent Demographic Health Survey or imputed based on sub-regional average. Indicator used: % women in the past five years who took iron tablets or syrup for >90 days.

South Sudan

Region: Sub-Saharan Africa**; Sub-region:** Eastern Sub-Saharan Africa

Low birthweight prevalence[1]: 15.4% (95% CI: 9.9, 20.9)

Preterm birth prevalence[2]: 12.0% (95% CI: 8.6, 16.7)

Number of births[3]: 1,928,000

Returns to education[4]: 11.3% (95% CI: 9.7, 12.9)

GDP per capita 2010 US$ (estimated annual wage)[5]: $787 ($525/year)

GDP per capita 2011 International $ (estimated annual wage)[5]: $1910 ($1273/year)

Prevalence of low BMI[6]: 10.3% (95% CI: 2.5, 18.2)

Prevalence of anemia[7]: 36.0% (95% CI: 12.5, 59.5)

Baseline coverage of IFA[8]: 19.2%

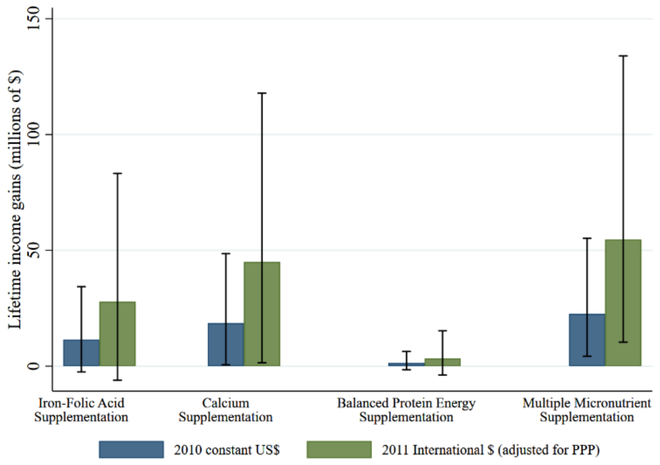

Figure: Benefits by birth cohort size for estimated gains in lifetime wages attributable to scaling up nutrition interventions to 90% target coverage

Table: Impact of maternal prenatal nutrition intervention on human capital and labour market outcomes, through improvements in low birthweight or preterm birth and schooling in South Sudan.

Intervention	Target Coverage (%)	Absolute reduction in birth outcome (%)	Benefits by cohorts: School years gained (in 1000 s)	No. of additional students completing secondary school	Benefits by cohorts: Lifetime wages		Returns in lifetime earnings per child born to a targeted pregnant woman	
					in US $ millions	in International $ millions	in US $	in International $
Iron/Folic Acid Suppl.	90%	1.76 (-0.34, 3.94)	7.24 (-1.47, 22.10)	198 (-40, 606)	11.46 (-2.50, 34.30)	27.80 (-6.05, 83.21)	6.60 (-1.44, 19.77)	16.02 (-3.49, 47.95)
Calcium Suppl.	50%	1.39 (0.29, 2.56)	6.40 (0.19, 16.37)	175 (5, 449)	10.30 (0.33, 26.99)	25.00 (0.81, 65.47)	10.69 (0.34, 27.99)	25.93 (0.84, 67.91)
	90%	2.51 (0.53, 4.60)	11.51 (0.34, 29.47)	316 (9, 808)	18.55 (0.60, 48.58)	44.99 (1.45, 117.84)	10.69 (0.34, 27.99)	25.93 (0.84, 67.91)
Multiple Micronutrient Suppl.	50%	1.60 (0.64, 2.62)	7.08 (1.28, 16.53)	194 (35, 453)	11.25 (2.05, 27.30)	27.29 (4.98, 66.23)	11.67 (2.13, 28.32)	28.31 (5.17, 68.70)
	90%	3.25 (1.17, 5.56)	14.24 (2.62, 34.30)	390 (72, 940)	22.54 (4.25, 55.22)	54.67 (10.32, 133.96)	12.99 (2.45, 31.82)	31.51 (5.95, 77.20)
Balanced Protein Suppl.	50%	0.12 (-0.11, 0.42)	0.47 (-0.54, 2.07)	13 (-15, 57)	0.75 (-0.88, 3.50)	1.81 (-2.14, 8.50)	8.36 (-8.05, 28.57)	20.27 (-19.53, 69.31)
	90%	0.21 (-0.19, 0.75)	0.84 (-0.97, 3.73)	23 (-27, 102)	1.34 (-1.59, 6.31)	3.26 (-3.85, 15.30)	8.36 (-8.05, 28.57)	20.27 (-19.53, 69.31)

References for Data Inputs
[1] Blencowe H, Krasevec J, Onis M De, et al. Articles National , regional , and worldwide estimates of low birthweight in 2015 , with trends from 2000: a systematic analysis. Lancet Glob Heal. 2019;(18):1-12.
[2] Chawanpaiboon S, Vogel JP, Moller AB, et al. Global, regional, and national estimates of levels of preterm birth in 2014: a systematic review and modelling analysis. Lancet Glob Heal. 2019;7(1):e37-e46.
[3] United National Population Division World Population Prospects 2019.
[4] Fink G, Peet E, Danaei G, et al. Schooling and wage income losses due to early-childhood growth faltering in developing countries: National, regional, and global estimates. Am J Clin Nutr. 2016;104(1):104-112.
[5] Country specific annual wage data from World Indicators Database. Average yearly wage was estimated to be 2/3 of the gross domestic product in 2010 constant US dollars and 2011 International dollars, adjusted for purchasing power parity.
[6] NCD Risk Factor Collaboration. Trends in adult body-mass index in 200 countries from 1975 to 2014: a pooled analysis of 1698 population-based measurement studies with 19·2 million participants. Lancet. 2016;387(10026):1377-1396.
[7] Stevens GA, Finucane MM, De-Regil LM, et al. Global, regional, and national trends in haemoglobin concentration and prevalence of total and severe anaemia in children and pregnant and non-pregnant women for 1995-2011: A systematic analysis of population-representative data. Lancet Glob Heal. 2013;1(1):16-25.
[8] Coverage of iron-folic acid supplementation abstracted from the most recent Demographic Health Survey or imputed based on sub-regional average. Indicator used: % women in the past five years who took iron tablets or syrup for >90 days.

Sri Lanka

Region: Southeast Asia, East Asia, and Oceania**; Sub-region:** Southeast Asia

Low birthweight prevalence[1]: 15.9% (95% CI: 15.6, 16.1)

Preterm birth prevalence[2]: 7.0% (95% CI: 4.2, 10.7)

Number of births[3]: 1,695,000

Returns to education[4]: 1.9% (95% CI: 1.7, 2.2)

GDP per capita 2010 US$ (estimated annual wage)[5]: $3647 ($2432/year)

GDP per capita 2011 International $ (estimated annual wage)[5]: $11078 ($7385/year)

Prevalence of low BMI[6]: 12.8% (95% CI: 7.6, 19.4)

Prevalence of anemia[7]: 25.9% (95% CI: 16.0, 40.4)

Baseline coverage of IFA[8]: 46.2%

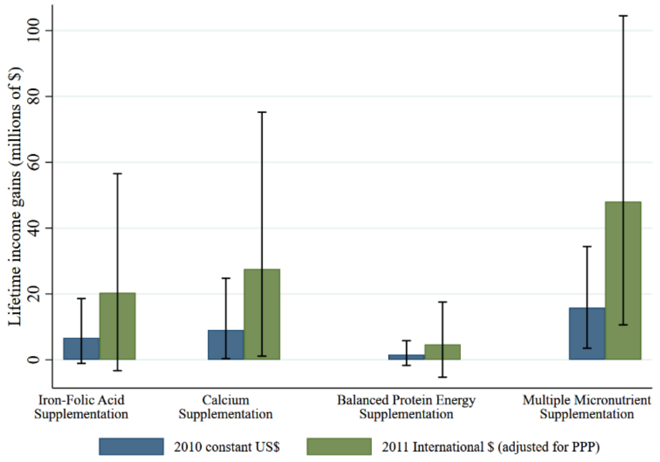

Figure: Benefits by birth cohort size for estimated gains in lifetime wages attributable to scaling up nutrition interventions to 90% target coverage

Table: Impact of maternal prenatal nutrition intervention on human capital and labour market outcomes, through improvements in low birthweight or preterm birth and schooling in Sri Lanka.

Intervention	Target Coverage (%)	Absolute reduction in birth outcome (%)	Benefits by cohorts: School years gained (in 1000 s)	No. of additional students completing secondary school	Benefits by cohorts: Lifetime wages		Returns in lifetime earnings per child born to a targeted pregnant woman	
					in US $ millions	in International $ millions	in US $	in International $
Iron/Folic Acid Suppl.	90%	1.21 (-0.22, 2.51)	5.26 (-0.91, 14.66)	581 (-101, 1619)	6.70 (-1.09, 18.61)	20.34 (-3.30, 56.53)	4.39 (-0.71, 12.20)	13.33 (-2.17, 37.06)
Calcium Suppl.	50%	0.80 (0.14, 1.59)	3.94 (0.15, 10.49)	435 (16, 1158)	5.05 (0.20, 13.76)	15.32 (0.60, 41.78)	5.95 (0.23, 16.23)	18.08 (0.71, 49.30)
	90%	1.45 (0.26, 2.85)	7.09 (0.26, 18.88)	783 (29, 2085)	9.08 (0.36, 24.76)	27.58 (1.08, 75.21)	5.95 (0.23, 16.23)	18.08 (0.71, 49.30)
Multiple Micronutrient Suppl.	50%	0.94 (0.50, 1.32)	4.41 (1.00, 9.35)	487 (110, 1032)	5.62 (1.14, 11.66)	17.08 (3.46, 35.42)	6.63 (1.34, 13.76)	20.15 (4.08, 41.79)
	90%	2.71 (1.25, 4.06)	12.44 (2.73, 26.43)	1373 (301, 2918)	15.83 (3.49, 34.42)	48.07 (10.61, 104.54)	10.38 (2.29, 22.56)	31.51 (6.96, 68.53)
Balanced Protein Suppl.	50%	0.17 (-0.16, 0.44)	0.67 (-0.79, 2.51)	74 (-87, 277)	0.86 (-0.97, 3.21)	2.60 (-2.94, 9.74)	8.46 (-7.86, 25.20)	25.70 (-23.86, 76.53)
	90%	0.30 (-0.29, 0.80)	1.21 (-1.42, 4.51)	134 (-157, 498)	1.54 (-1.74, 5.77)	4.68 (-5.29, 17.54)	8.46 (-7.86, 25.20)	25.70 (-23.86, 76.53)

References for Data Inputs

[1] Blencowe H, Krasevec J, Onis M De, et al. Articles National , regional , and worldwide estimates of low birthweight in 2015 , with trends from 2000: a systematic analysis. Lancet Glob Heal. 2019;(18):1-12.

[2] Chawanpaiboon S, Vogel JP, Moller AB, et al. Global, regional, and national estimates of levels of preterm birth in 2014: a systematic review and modelling analysis. Lancet Glob Heal. 2019;7(1):e37-e46.

[3] United National Population Division World Population Prospects 2019.

[4] Fink G, Peet E, Danaei G, et al. Schooling and wage income losses due to early-childhood growth faltering in developing countries: National, regional, and global estimates. Am J Clin Nutr. 2016;104(1):104-112.

[5] Country specific annual wage data from World Indicators Database. Average yearly wage was estimated to be 2/3 of the gross domestic product in 2010 constant US dollars and 2011 International dollars, adjusted for purchasing power parity.

[6] NCD Risk Factor Collaboration. Trends in adult body-mass index in 200 countries from 1975 to 2014: a pooled analysis of 1698 population-based measurement studies with 19·2 million participants. Lancet. 2016;387(10026):1377-1396.

[7] Stevens GA, Finucane MM, De-Regil LM, et al. Global, regional, and national trends in haemoglobin concentration and prevalence of total and severe anaemia in children and pregnant and non-pregnant women for 1995-2011: A systematic analysis of population-representative data. Lancet Glob Heal. 2013;1(1):16-25.

[8] Coverage of iron-folic acid supplementation abstracted from the most recent Demographic Health Survey or imputed based on sub-regional average. Indicator used: % women in the past five years who took iron tablets or syrup for >90 days.

Saint Lucia

Region: Latin America and Caribbean**; Sub-region:** Caribbean

Low birthweight prevalence[1]: 10.1% (95% CI: 6.3, 13.9)

Preterm birth prevalence[2]: 9.8% (95% CI: 8.6, 11.3)

Number of births[3]: 11,000

Returns to education[4]: 9.8% (95% CI: 8.1, 11.5)

GDP per capita 2010 US$ (estimated annual wage)[5]: $7969 ($5313/year)

GDP per capita 2011 International $ (estimated annual wage)[5]: $11568 ($7712/year)

Prevalence of low BMI[6]: 3.6% (95% CI: 1.4, 7.2)

Prevalence of anemia[7]: 32.2% (95% CI: 15.8, 56.7)

Baseline coverage of IFA[8]: 62.3%

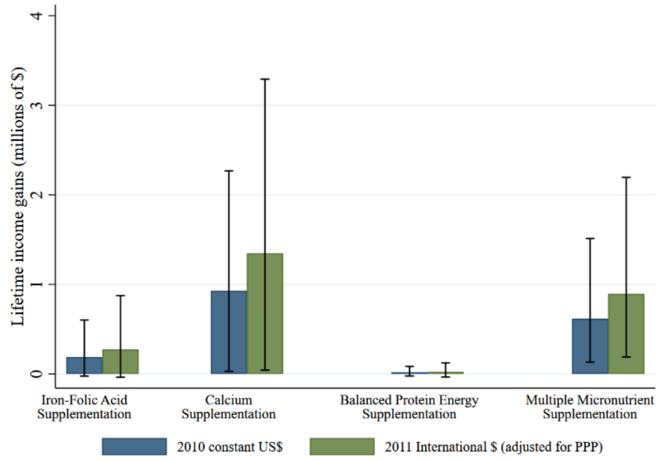

Figure: Benefits by birth cohort size for estimated gains in lifetime wages attributable to scaling up nutrition interventions to 90% target coverage

Table: Impact of maternal prenatal nutrition intervention on human capital and labour market outcomes, through improvements in low birthweight or preterm birth and schooling in Saint Lucia.

Intervention	Target Coverage (%)	Absolute reduction in birth outcome (%)	Benefits by cohorts: School years gained (in 1000 s)	No. of additional students completing secondary school	Benefits by cohorts: Lifetime wages		Returns in lifetime earnings per child born to a targeted pregnant woman	
					in US $ millions	in International $ millions	in US $	in International $
Iron/Folic Acid Suppl.	90%	0.48 (-0.08, 1.18)	0.01 (-0.00, 0.04)	1 (-0, 2)	0.19 (-0.02, 0.60)	0.27 (-0.04, 0.88)	18.97 (-2.48, 60.94)	27.53 (-3.61, 88.46)
Calcium Suppl.	50%	1.17 (0.26, 1.94)	0.04 (0.00, 0.09)	2 (0, 5)	0.52 (0.02, 1.26)	0.75 (0.02, 1.83)	93.73 (2.90, 229.06)	136.06 (4.21, 332.50)
	90%	2.11 (0.47, 3.49)	0.07 (0.00, 0.16)	3 (0, 8)	0.93 (0.03, 2.27)	1.35 (0.04, 3.29)	93.73 (2.90, 229.06)	136.06 (4.21, 332.50)
Multiple Micronutrient Suppl.	50%	0.61 (0.27, 1.02)	0.02 (0.00, 0.04)	1 (0, 2)	0.25 (0.04, 0.62)	0.36 (0.06, 0.90)	45.68 (7.95, 113.25)	66.31 (11.54, 164.39)
	90%	1.50 (0.74, 2.47)	0.04 (0.01, 0.10)	2 (0, 5)	0.62 (0.13, 1.51)	0.89 (0.19, 2.20)	62.18 (13.23, 152.83)	90.27 (19.21, 221.84)
Balanced Protein Suppl.	50%	0.03 (-0.03, 0.09)	0.00 (-0.00, 0.00)	0 (-0, 0)	0.01 (-0.01, 0.05)	0.01 (-0.02, 0.07)	57.94 (-55.90, 198.49)	84.10 (-81.14, 288.13)
	90%	0.05 (-0.05, 0.17)	0.00 (-0.00, 0.01)	0 (-0, 0)	0.02 (-0.02, 0.09)	0.03 (-0.03, 0.12)	57.94 (-55.90, 198.49)	84.10 (-81.14, 288.13)

References for Data Inputs

[1] Blencowe H, Krasevec J, Onis M De, et al. Articles National , regional , and worldwide estimates of low birthweight in 2015 , with trends from 2000: a systematic analysis. Lancet Glob Heal. 2019;(18):1-12.

[2] Chawanpaiboon S, Vogel JP, Moller AB, et al. Global, regional, and national estimates of levels of preterm birth in 2014: a systematic review and modelling analysis. Lancet Glob Heal. 2019;7(1):e37-e46.

[3] United National Population Division World Population Prospects 2019.

[4] Fink G, Peet E, Danaei G, et al. Schooling and wage income losses due to early-childhood growth faltering in developing countries: National, regional, and global estimates. Am J Clin Nutr. 2016;104(1):104-112.

[5] Country specific annual wage data from World Indicators Database. Average yearly wage was estimated to be 2/3 of the gross domestic product in 2010 constant US dollars and 2011 International dollars, adjusted for purchasing power parity.

[6] NCD Risk Factor Collaboration. Trends in adult body-mass index in 200 countries from 1975 to 2014: a pooled analysis of 1698 population-based measurement studies with 19·2 million participants. Lancet. 2016;387(10026):1377-1396.

[7] Stevens GA, Finucane MM, De-Regil LM, et al. Global, regional, and national trends in haemoglobin concentration and prevalence of total and severe anaemia in children and pregnant and non-pregnant women for 1995-2011: A systematic analysis of population-representative data. Lancet Glob Heal. 2013;1(1):16-25.

[8] Coverage of iron-folic acid supplementation abstracted from the most recent Demographic Health Survey or imputed based on sub-regional average. Indicator used: % women in the past five years who took iron tablets or syrup for >90 days.

Saint Vincent and the Grenadines

Region: Latin America and Caribbean**; Sub-region:** Caribbean

Low birthweight prevalence[1]: 7.9% (95% CI: 4.2, 11.7)

Preterm birth prevalence[2]: 9.8% (95% CI: 8.6, 11.3)

Number of births[3]: 8,000

Returns to education[4]: 9.8% (95% CI: 8.1, 11.5)

GDP per capita 2010 US$ (estimated annual wage)[5]: $6582 ($4388/year)

GDP per capita 2011 International $ (estimated annual wage)[5]: $10472 ($6981/year)

Prevalence of low BMI[6]: 3.3% (95% CI: 1.0, 7.4)

Prevalence of anemia[7]: 30.7% (95% CI: 14.3, 54.1)

Baseline coverage of IFA[8]: 62.3%

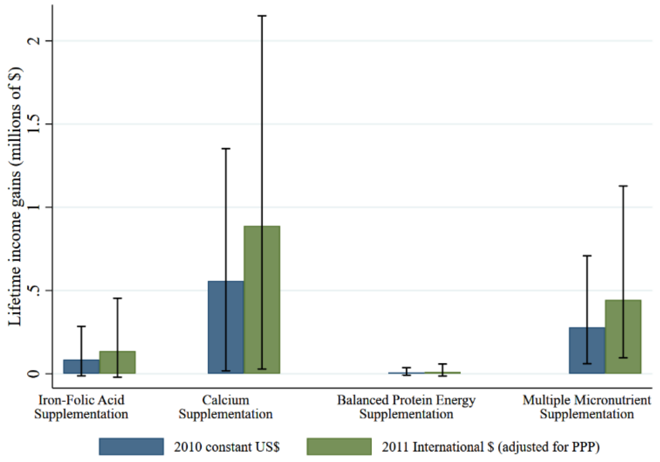

Figure: Benefits by birth cohort size for estimated gains in lifetime wages attributable to scaling up nutrition interventions to 90% target coverage

Table: Impact of maternal prenatal nutrition intervention on human capital and labour market outcomes, through improvements in low birthweight or preterm birth and schooling in Saint Vincent and the Grenadines.

Intervention	Target Coverage (%)	Absolute reduction in birth outcome (%)	Benefits by cohorts: School years gained (in 1000 s)	No. of additional students completing secondary school	Benefits by cohorts: Lifetime wages		Returns in lifetime earnings per child born to a targeted pregnant woman	
					in US $ millions	in International $ millions	in US $	in International $
Iron/Folic Acid Suppl.	90%	0.37 (-0.07, 0.95)	0.01 (-0.00, 0.02)	0 (-0, 1)	0.09 (-0.01, 0.29)	0.14 (-0.02, 0.45)	11.84 (-1.79, 39.60)	18.84 (-2.84, 63.01)
Calcium Suppl.	50%	1.18 (0.26, 1.98)	0.03 (0.00, 0.06)	1 (0, 3)	0.31 (0.01, 0.75)	0.49 (0.02, 1.20)	77.59 (2.49, 187.78)	123.46 (3.96, 298.78)
	90%	2.12 (0.46, 3.57)	0.05 (0.00, 0.11)	2 (0, 6)	0.56 (0.02, 1.35)	0.89 (0.03, 2.15)	77.59 (2.49, 187.78)	123.46 (3.96, 298.78)
Multiple Micronutrient Suppl.	50%	0.45 (0.17, 0.84)	0.01 (0.00, 0.02)	0 (0, 1)	0.11 (0.02, 0.29)	0.18 (0.03, 0.47)	27.79 (5.25, 73.22)	44.22 (8.35, 116.50)
	90%	1.14 (0.48, 2.05)	0.02 (0.01, 0.06)	1 (0, 3)	0.28 (0.06, 0.71)	0.44 (0.10, 1.13)	38.77 (8.35, 98.46)	61.69 (13.29, 156.66)
Balanced Protein Suppl.	50%	0.02 (-0.02, 0.07)	0.00 (-0.00, 0.00)	0 (-0, 0)	0.00 (-0.00, 0.02)	0.01 (-0.01, 0.03)	33.69 (-32.44, 131.82)	53.61 (-51.62, 209.74)
	90%	0.03 (-0.04, 0.13)	0.00 (-0.00, 0.00)	0 (-0, 0)	0.01 (-0.01, 0.04)	0.01 (-0.01, 0.06)	33.69 (-32.44, 131.82)	53.61 (-51.62, 209.74)

References for Data Inputs
[1] Blencowe H, Krasevec J, Onis M De, et al. Articles National , regional , and worldwide estimates of low birthweight in 2015 , with trends from 2000: a systematic analysis. Lancet Glob Heal. 2019;(18):1-12.
[2] Chawanpaiboon S, Vogel JP, Moller AB, et al. Global, regional, and national estimates of levels of preterm birth in 2014: a systematic review and modelling analysis. Lancet Glob Heal. 2019;7(1):e37-e46.
[3] United National Population Division World Population Prospects 2019.
[4] Fink G, Peet E, Danaei G, et al. Schooling and wage income losses due to early-childhood growth faltering in developing countries: National, regional, and global estimates. Am J Clin Nutr. 2016;104(1):104-112.
[5] Country specific annual wage data from World Indicators Database. Average yearly wage was estimated to be 2/3 of the gross domestic product in 2010 constant US dollars and 2011 International dollars, adjusted for purchasing power parity.
[6] NCD Risk Factor Collaboration. Trends in adult body-mass index in 200 countries from 1975 to 2014: a pooled analysis of 1698 population-based measurement studies with 19·2 million participants. Lancet. 2016;387(10026):1377-1396.
[7] Stevens GA, Finucane MM, De-Regil LM, et al. Global, regional, and national trends in haemoglobin concentration and prevalence of total and severe anaemia in children and pregnant and non-pregnant women for 1995-2011: A systematic analysis of population-representative data. Lancet Glob Heal. 2013;1(1):16-25.
[8] Coverage of iron-folic acid supplementation abstracted from the most recent Demographic Health Survey or imputed based on sub-regional average. Indicator used: % women in the past five years who took iron tablets or syrup for >90 days.

Sudan

Region: Sub-Saharan Africa**; Sub-region:** Eastern Sub-Saharan Africa

Low birthweight prevalence[1]: 30.7% (95% CI: 25.1, 36.3)

Preterm birth prevalence[2]: 13.4% (95% CI: 6.3, 30.9)

Number of births[3]: 6,695,000

Returns to education[4]: 11.3% (95% CI: 9.7, 12.9)

GDP per capita 2010 US$ (estimated annual wage)[5]: $1870 ($1246/year)

GDP per capita 2011 International $ (estimated annual wage)[5]: $4262 ($2841/year)

Prevalence of low BMI[6]: 8.1% (95% CI: 3.2, 15.3)

Prevalence of anemia[7]: 35.1% (95% CI: 19.3, 55.7)

Baseline coverage of IFA[8]: 19.2%

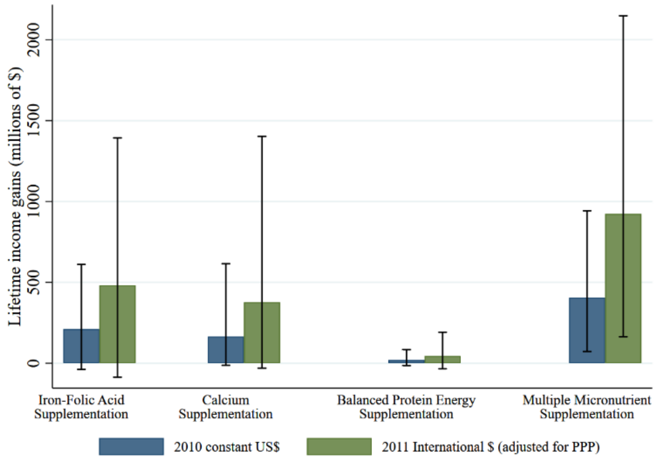

Figure: Benefits by birth cohort size for estimated gains in lifetime wages attributable to scaling up nutrition interventions to 90% target coverage

Table: Impact of maternal prenatal nutrition intervention on human capital and labour market outcomes, through improvements in low birthweight or preterm birth and schooling in Sudan.

Intervention	Target Coverage (%)	Absolute reduction in birth outcome (%)	Benefits by cohorts: School years gained (in 1000 s)	No. of additional students completing secondary school	Benefits by cohorts: Lifetime wages		Returns in lifetime earnings per child born to a targeted pregnant woman	
					in US $ millions	in International $ millions	in US $	in International $
Iron/Folic Acid Suppl.	90%	3.52 (-0.59, 7.46)	55.34 (-9.84, 152.40)	520 (-92, 1433)	211.28 (-37.88, 610.98)	481.68 (-86.36, 1392.92)	35.06 (-6.29, 101.40)	79.94 (-14.33, 231.17)
Calcium Suppl.	50%	1.46 (-0.06, 3.79)	24.40 (-1.92, 89.07)	229 (-18, 837)	91.79 (-7.51, 341.74)	209.27 (-17.12, 779.11)	27.42 (-2.24, 102.09)	62.51 (-5.11, 232.74)
	90%	2.62 (-0.11, 6.83)	43.93 (-3.46, 160.32)	413 (-33, 1507)	165.22 (-13.52, 615.14)	376.68 (-30.81, 1402.40)	27.42 (-2.24, 102.09)	62.51 (-5.11, 232.74)
Multiple Micronutrient Suppl.	50%	3.20 (1.50, 4.84)	52.07 (10.91, 117.11)	489 (103, 1101)	198.81 (42.95, 449.48)	453.26 (97.92, 1024.73)	59.39 (12.83, 134.27)	135.40 (29.25, 306.12)
	90%	6.54 (2.59, 10.20)	106.15 (20.14, 238.03)	998 (189, 2238)	405.17 (71.83, 942.61)	923.71 (163.77, 2148.97)	67.24 (11.92, 156.44)	153.30 (27.18, 356.65)
Balanced Protein Suppl.	50%	0.19 (-0.15, 0.63)	2.85 (-2.27, 12.58)	27 (-21, 118)	10.92 (-8.49, 46.67)	24.90 (-19.36, 106.41)	43.23 (-40.96, 142.24)	98.57 (-93.37, 324.28)
	90%	0.34 (-0.26, 1.13)	5.13 (-4.08, 22.65)	48 (-38, 213)	19.66 (-15.29, 84.01)	44.82 (-34.85, 191.53)	43.23 (-40.96, 142.24)	98.57 (-93.37, 324.28)

References for Data Inputs
[1] Blencowe H, Krasevec J, Onis M De, et al. Articles National , regional , and worldwide estimates of low birthweight in 2015 , with trends from 2000: a systematic analysis. Lancet Glob Heal. 2019;(18):1-12.
[2] Chawanpaiboon S, Vogel JP, Moller AB, et al. Global, regional, and national estimates of levels of preterm birth in 2014: a systematic review and modelling analysis. Lancet Glob Heal. 2019;7(1):e37-e46.
[3] United National Population Division World Population Prospects 2019.
[4] Fink G, Peet E, Danaei G, et al. Schooling and wage income losses due to early-childhood growth faltering in developing countries: National, regional, and global estimates. Am J Clin Nutr. 2016;104(1):104-112.
[5] Country specific annual wage data from World Indicators Database. Average yearly wage was estimated to be 2/3 of the gross domestic product in 2010 constant US dollars and 2011 International dollars, adjusted for purchasing power parity.
[6] NCD Risk Factor Collaboration. Trends in adult body-mass index in 200 countries from 1975 to 2014: a pooled analysis of 1698 population-based measurement studies with 19·2 million participants. Lancet. 2016;387(10026):1377-1396.
[7] Stevens GA, Finucane MM, De-Regil LM, et al. Global, regional, and national trends in haemoglobin concentration and prevalence of total and severe anaemia in children and pregnant and non-pregnant women for 1995-2011: A systematic analysis of population-representative data. Lancet Glob Heal. 2013;1(1):16-25.
[8] Coverage of iron-folic acid supplementation abstracted from the most recent Demographic Health Survey or imputed based on sub-regional average. Indicator used: % women in the past five years who took iron tablets or syrup for >90 days.

Suriname

Region: Latin America and Caribbean**; Sub-region:** Caribbean

Low birthweight prevalence[1]: 14.7% (95% CI: 11.4, 18.7)

Preterm birth prevalence[2]: 9.8% (95% CI: 8.6, 11.3)

Number of births[3]: 53,000

Returns to education[4]: 9.8% (95% CI: 8.1, 11.5)

GDP per capita 2010 US$ (estimated annual wage)[5]: $8465 ($5643/year)

GDP per capita 2011 International $ (estimated annual wage)[5]: $14488 ($9659/year)

Prevalence of low BMI[6]: 2.8% (95% CI: 1.0, 5.7)

Prevalence of anemia[7]: 31.0% (95% CI: 14.6, 55.1)

Baseline coverage of IFA[8]: 62.3%

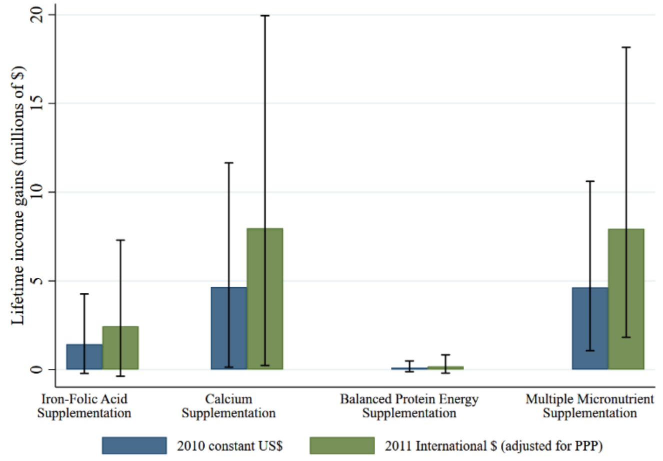

Figure: Benefits by birth cohort size for estimated gains in lifetime wages attributable to scaling up nutrition interventions to 90% target coverage

Table: Impact of maternal prenatal nutrition intervention on human capital and labour market outcomes, through improvements in low birthweight or preterm birth and schooling in Suriname.

Intervention	Target Coverage (%)	Absolute reduction in birth outcome (%)	Benefits by cohorts: School years gained (in 1000 s)	No. of additional students completing secondary school	Benefits by cohorts: Lifetime wages		Returns in lifetime earnings per child born to a targeted pregnant woman	
					in US $ millions	in International $ millions	in US $	in International $
**Iron/Folic Acid Suppl.**	90%	0.72 (-0.12, 1.63)	0.10 (-0.02, 0.28)	5 (-1, 15)	1.43 (-0.22, 4.26)	2.45 (-0.37, 7.29)	29.95 (-4.53, 89.33)	51.26 (-7.76, 152.89)
**Calcium Suppl.**	50%	1.17 (0.25, 2.00)	0.18 (0.00, 0.43)	9 (0, 22)	2.58 (0.08, 6.47)	4.42 (0.13, 11.08)	97.53 (2.88, 244.28)	166.93 (4.93, 418.12)
	90%	2.10 (0.46, 3.59)	0.32 (0.01, 0.78)	16 (0, 40)	4.65 (0.14, 11.65)	7.96 (0.24, 19.94)	97.53 (2.88, 244.28)	166.93 (4.93, 418.12)
**Multiple Micronutrient Suppl.**	50%	0.88 (0.42, 1.34)	0.13 (0.03, 0.29)	7 (1, 15)	1.90 (0.38, 4.40)	3.25 (0.65, 7.54)	71.64 (14.29, 166.19)	122.62 (24.45, 284.46)
	90%	2.21 (1.16, 3.43)	0.31 (0.07, 0.70)	16 (4, 36)	4.63 (1.06, 10.61)	7.93 (1.82, 18.16)	97.13 (22.32, 222.45)	166.25 (38.19, 380.74)
**Balanced Protein Suppl.**	50%	0.03 (-0.03, 0.11)	0.00 (-0.00, 0.02)	0 (-0, 1)	0.06 (-0.06, 0.27)	0.10 (-0.11, 0.46)	89.26 (-83.25, 283.12)	152.78 (-142.50, 484.58)
	90%	0.06 (-0.05, 0.19)	0.01 (-0.01, 0.03)	0 (-0, 2)	0.11 (-0.12, 0.48)	0.18 (-0.20, 0.83)	89.26 (-83.25, 283.12)	152.78 (-142.50, 484.58)

References for Data Inputs

[1] Blencowe H, Krasevec J, Onis M De, et al. Articles National , regional , and worldwide estimates of low birthweight in 2015 , with trends from 2000: a systematic analysis. Lancet Glob Heal. 2019;(18):1-12.
[2] Chawanpaiboon S, Vogel JP, Moller AB, et al. Global, regional, and national estimates of levels of preterm birth in 2014: a systematic review and modelling analysis. Lancet Glob Heal. 2019;7(1):e37-e46.
[3] United National Population Division World Population Prospects 2019.
[4] Fink G, Peet E, Danaei G, et al. Schooling and wage income losses due to early-childhood growth faltering in developing countries: National, regional, and global estimates. Am J Clin Nutr. 2016;104(1):104-112.
[5] Country specific annual wage data from World Indicators Database. Average yearly wage was estimated to be 2/3 of the gross domestic product in 2010 constant US dollars and 2011 International dollars, adjusted for purchasing power parity.
[6] NCD Risk Factor Collaboration. Trends in adult body-mass index in 200 countries from 1975 to 2014: a pooled analysis of 1698 population-based measurement studies with 19·2 million participants. Lancet. 2016;387(10026):1377-1396.
[7] Stevens GA, Finucane MM, De-Regil LM, et al. Global, regional, and national trends in haemoglobin concentration and prevalence of total and severe anaemia in children and pregnant and non-pregnant women for 1995-2011: A systematic analysis of population-representative data. Lancet Glob Heal. 2013;1(1):16-25.
[8] Coverage of iron-folic acid supplementation abstracted from the most recent Demographic Health Survey or imputed based on sub-regional average. Indicator used: % women in the past five years who took iron tablets or syrup for >90 days.

Syria

Region: North Africa and Middle East**; Sub-region:** North Africa and Middle East

Low birthweight prevalence[1]: 9.4% (95% CI: 5.4, 13.4)

Preterm birth prevalence[2]: 10.4% (95% CI: 8.7, 11.9)

Number of births[3]: 2,130,000

Returns to education[4]: 6.7% (95% CI: 4.1, 9.4)

GDP per capita 2010 US$ (estimated annual wage)[5]: $1864 ($1243/year)

GDP per capita 2011 International $ (estimated annual wage)[5]: $4685 ($3123/year)

Prevalence of low BMI[6]: 2.5% (95% CI: 0.9, 5.4)

Prevalence of anemia[7]: 30.2% (95% CI: 14.6, 54.4)

Baseline coverage of IFA[8]: 24.3%

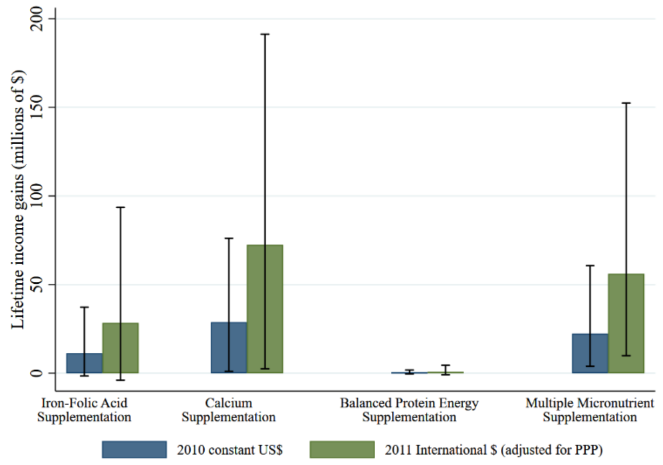

Figure: Benefits by birth cohort size for estimated gains in lifetime wages attributable to scaling up nutrition interventions to 90% target coverage

Table: Impact of maternal prenatal nutrition intervention on human capital and labour market outcomes, through improvements in low birthweight or preterm birth and schooling in Syria.

Intervention	Target Coverage (%)	Absolute reduction in birth outcome (%)	Benefits by cohorts: School years gained (in 1000 s)	No. of additional students completing secondary school	Benefits by cohorts: Lifetime wages		Returns in lifetime earnings per child born to a targeted pregnant woman	
					in US $ millions	in International $ millions	in US $	in International $
Iron/Folic Acid Suppl.	90%	0.98 (-0.18, 2.33)	5.21 (-0.75, 15.46)	147 (-21, 436)	11.29 (-1.56, 37.24)	28.38 (-3.92, 93.58)	5.89 (-0.81, 19.43)	14.80 (-2.04, 48.82)
Calcium Suppl.	50%	1.24 (0.27, 2.10)	7.32 (0.22, 17.15)	207 (6, 484)	16.01 (0.55, 42.29)	40.23 (1.39, 106.27)	15.03 (0.52, 39.71)	37.77 (1.31, 99.79)
	90%	2.23 (0.49, 3.79)	13.18 (0.40, 30.86)	372 (11, 870)	28.82 (1.00, 76.12)	72.41 (2.50, 191.29)	15.03 (0.52, 39.71)	37.77 (1.31, 99.79)
Multiple Micronutrient Suppl.	50%	0.87 (0.37, 1.53)	4.81 (0.91, 11.43)	136 (26, 322)	10.58 (1.91, 28.82)	26.59 (4.80, 72.43)	9.94 (1.80, 27.06)	24.97 (4.51, 68.01)
	90%	1.86 (0.70, 3.40)	10.17 (1.75, 25.02)	287 (49, 706)	22.31 (3.95, 60.66)	56.07 (9.92, 152.43)	11.64 (2.06, 31.64)	29.25 (5.18, 79.52)
Balanced Protein Suppl.	50%	0.02 (-0.02, 0.07)	0.08 (-0.10, 0.42)	2 (-3, 12)	0.18 (-0.20, 0.98)	0.45 (-0.49, 2.47)	8.25 (-7.17, 29.73)	20.73 (-18.02, 74.71)
	90%	0.03 (-0.03, 0.12)	0.14 (-0.17, 0.76)	4 (-5, 22)	0.32 (-0.35, 1.77)	0.81 (-0.89, 4.45)	8.25 (-7.17, 29.73)	20.73 (-18.02, 74.71)

References for Data Inputs

[1] Blencowe H, Krasevec J, Onis M De, et al. Articles National , regional , and worldwide estimates of low birthweight in 2015 , with trends from 2000: a systematic analysis. Lancet Glob Heal. 2019;(18):1-12.
[2] Chawanpaiboon S, Vogel JP, Moller AB, et al. Global, regional, and national estimates of levels of preterm birth in 2014: a systematic review and modelling analysis. Lancet Glob Heal. 2019;7(1):e37-e46.
[3] United National Population Division World Population Prospects 2019.
[4] Fink G, Peet E, Danaei G, et al. Schooling and wage income losses due to early-childhood growth faltering in developing countries: National, regional, and global estimates. Am J Clin Nutr. 2016;104(1):104-112.
[5] Country specific annual wage data from World Indicators Database. Average yearly wage was estimated to be 2/3 of the gross domestic product in 2010 constant US dollars and 2011 International dollars, adjusted for purchasing power parity.
[6] NCD Risk Factor Collaboration. Trends in adult body-mass index in 200 countries from 1975 to 2014: a pooled analysis of 1698 population-based measurement studies with 19·2 million participants. Lancet. 2016;387(10026):1377-1396.
[7] Stevens GA, Finucane MM, De-Regil LM, et al. Global, regional, and national trends in haemoglobin concentration and prevalence of total and severe anaemia in children and pregnant and non-pregnant women for 1995-2011: A systematic analysis of population-representative data. Lancet Glob Heal. 2013;1(1):16-25.
[8] Coverage of iron-folic acid supplementation abstracted from the most recent Demographic Health Survey or imputed based on sub-regional average. Indicator used: % women in the past five years who took iron tablets or syrup for >90 days.

Tajikistan

Region: Central Europe, Eastern Europe, Central Asia**; Sub-region:** Central Asia

Low birthweight prevalence[1]: 5.6% (95% CI: 4.4, 7.3)

Preterm birth prevalence[2]: 10.4% (95% CI: 8.7, 11.9)

Number of births[3]: 1,404,000

Returns to education[4]: 3.2% (95% CI: 0.8, 5.5)

GDP per capita 2010 US$ (estimated annual wage)[5]: $936 ($624/year)

GDP per capita 2011 International $ (estimated annual wage)[5]: $2670 ($1780/year)

Prevalence of low BMI[6]: 4.6% (95% CI: 2.0, 8.7)

Prevalence of anemia[7]: 28.6% (95% CI: 16.7, 47.8)

Baseline coverage of IFA[8]: 2.2%

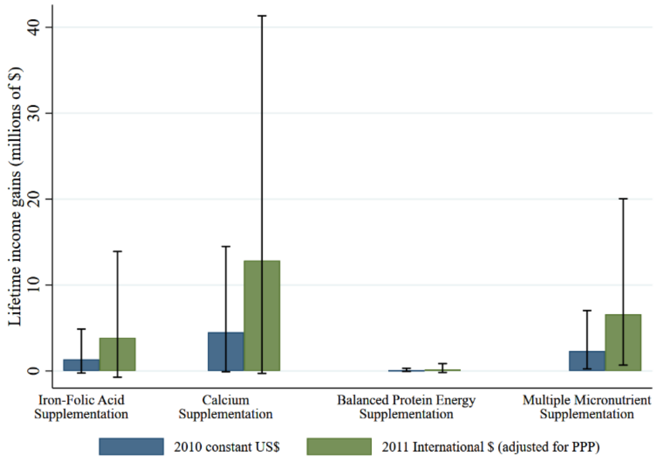

Figure: Benefits by birth cohort size for estimated gains in lifetime wages attributable to scaling up nutrition interventions to 90% target coverage

Table: Impact of maternal prenatal nutrition intervention on human capital and labour market outcomes, through improvements in low birthweight or preterm birth and schooling in Tajikistan.

Intervention	Target Coverage (%)	Absolute reduction in birth outcome (%)	Benefits by cohorts: School years gained (in 1000 s)	No. of additional students completing secondary school	Benefits by cohorts: Lifetime wages		Returns in lifetime earnings per child born to a targeted pregnant woman	
					in US $ millions	in International $ millions	in US $	in International $
Iron/Folic Acid Suppl.	90%	0.79 (-0.16, 1.61)	2.74 (-0.53, 7.41)	103 (-20, 277)	1.35 (-0.25, 4.88)	3.86 (-0.72, 13.91)	1.07 (-0.20, 3.86)	3.06 (-0.57, 11.01)
Calcium Suppl.	50%	1.25 (0.27, 2.11)	4.94 (0.15, 12.13)	185 (6, 454)	2.50 (-0.06, 8.05)	7.14 (-0.16, 22.97)	3.56 (-0.08, 11.47)	10.17 (-0.23, 32.73)
	90%	2.25 (0.48, 3.79)	8.89 (0.27, 21.84)	332 (10, 817)	4.50 (-0.10, 14.50)	12.85 (-0.29, 41.35)	3.56 (-0.08, 11.47)	10.17 (-0.23, 32.73)
Multiple Micronutrient Suppl.	50%	0.70 (0.24, 1.13)	2.54 (0.43, 5.71)	95 (16, 214)	1.27 (0.13, 3.85)	3.63 (0.37, 10.99)	1.81 (0.19, 5.49)	5.17 (0.53, 15.65)
	90%	1.27 (0.43, 2.06)	4.62 (0.79, 10.41)	173 (30, 389)	2.31 (0.24, 7.03)	6.60 (0.68, 20.05)	1.83 (0.19, 5.56)	5.22 (0.54, 15.87)
Balanced Protein Suppl.	50%	0.02 (-0.02, 0.06)	0.06 (-0.07, 0.27)	2 (-2, 10)	0.03 (-0.03, 0.17)	0.09 (-0.10, 0.48)	1.16 (-1.17, 4.49)	3.31 (-3.34, 12.80)
	90%	0.03 (-0.03, 0.11)	0.12 (-0.12, 0.49)	4 (-4, 18)	0.05 (-0.06, 0.30)	0.16 (-0.18, 0.86)	1.16 (-1.17, 4.49)	3.31 (-3.34, 12.80)

References for Data Inputs

[1] Blencowe H, Krasevec J, Onis M De, et al. Articles National , regional , and worldwide estimates of low birthweight in 2015 , with trends from 2000: a systematic analysis. Lancet Glob Heal. 2019;(18):1-12.
[2] Chawanpaiboon S, Vogel JP, Moller AB, et al. Global, regional, and national estimates of levels of preterm birth in 2014: a systematic review and modelling analysis. Lancet Glob Heal. 2019;7(1):e37-e46.
[3] United National Population Division World Population Prospects 2019.
[4] Fink G, Peet E, Danaei G, et al. Schooling and wage income losses due to early-childhood growth faltering in developing countries: National, regional, and global estimates. Am J Clin Nutr. 2016;104(1):104-112.
[5] Country specific annual wage data from World Indicators Database. Average yearly wage was estimated to be 2/3 of the gross domestic product in 2010 constant US dollars and 2011 International dollars, adjusted for purchasing power parity.
[6] NCD Risk Factor Collaboration. Trends in adult body-mass index in 200 countries from 1975 to 2014: a pooled analysis of 1698 population-based measurement studies with 19·2 million participants. Lancet. 2016;387(10026):1377-1396.
[7] Stevens GA, Finucane MM, De-Regil LM, et al. Global, regional, and national trends in haemoglobin concentration and prevalence of total and severe anaemia in children and pregnant and non-pregnant women for 1995-2011: A systematic analysis of population-representative data. Lancet Glob Heal. 2013;1(1):16-25.
[8] Coverage of iron-folic acid supplementation abstracted from the most recent Demographic Health Survey or imputed based on sub-regional average. Indicator used: % women in the past five years who took iron tablets or syrup for >90 days.

Tanzania

Region: Sub-Saharan Africa**; Sub-region:** Eastern Sub-Saharan Africa

Low birthweight prevalence[1]: 10.5% (95% CI: 8.1, 13.4)

Preterm birth prevalence[2]: 16.6% (95% CI: 6.5, 33.4)

Number of births[3]: 10,260,000

Returns to education[4]: 8.2% (95% CI: 5.8, 10.7)

GDP per capita 2010 US$ (estimated annual wage)[5]: $872 ($581/year)

GDP per capita 2011 International $ (estimated annual wage)[5]: $2613 ($1742/year)

Prevalence of low BMI[6]: 9.8% (95% CI: 5.8, 14.8)

Prevalence of anemia[7]: 51.4% (95% CI: 42.8, 58.7)

Baseline coverage of IFA[8]: 21.4%

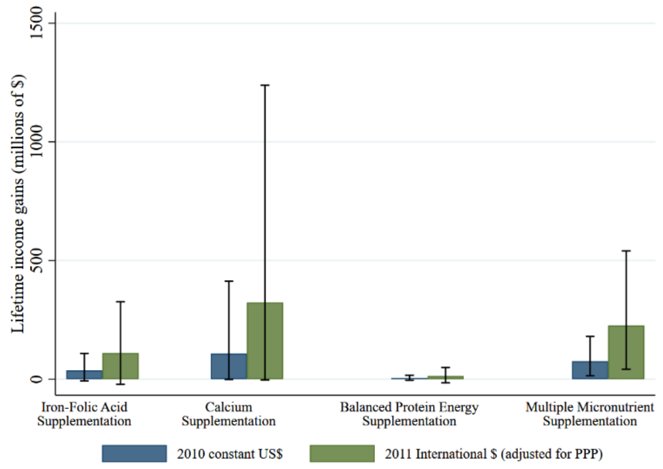

Figure: Benefits by birth cohort size for estimated gains in lifetime wages attributable to scaling up nutrition interventions to 90% target coverage

Table: Impact of maternal prenatal nutrition intervention on human capital and labour market outcomes, through improvements in low birthweight or preterm birth and schooling in Tanzania.

Intervention	Target Coverage (%)	Absolute reduction in birth outcome (%)	Benefits by cohorts: School years gained (in 1000 s)	No. of additional students completing secondary school	Benefits by cohorts: Lifetime wages		Returns in lifetime earnings per child born to a targeted pregnant woman	
					in US $ millions	in International $ millions	in US $	in International $
Iron/Folic Acid Suppl.	90%	1.18 (-0.22, 2.59)	28.85 (-5.06, 81.70)	664 (-116, 1879)	36.73 (-7.13, 108.83)	110.07 (-21.37, 326.16)	3.98 (-0.77, 11.79)	11.92 (-2.31, 35.32)
Calcium Suppl.	50%	1.80 (0.10, 4.50)	47.25 (-0.69, 164.99)	1087 (-16, 3795)	59.92 (-0.70, 229.58)	179.57 (-2.09, 688.04)	11.68 (-0.14, 44.75)	35.00 (-0.41, 134.12)
	90%	3.24 (0.18, 8.09)	85.06 (-1.24, 296.99)	1956 (-28, 6831)	107.85 (-1.25, 413.24)	323.22 (-3.75, 1238.48)	11.68 (-0.14, 44.75)	35.00 (-0.41, 134.12)
Multiple Micronutrient Suppl.	50%	1.14 (0.59, 1.72)	29.21 (6.35, 66.03)	672 (146, 1519)	37.00 (7.06, 87.11)	110.89 (21.16, 261.05)	7.21 (1.38, 16.98)	21.62 (4.13, 50.89)
	90%	2.34 (1.06, 3.73)	58.61 (12.37, 138.22)	1348 (285, 3179)	75.43 (13.93, 180.35)	226.07 (41.76, 540.51)	8.17 (1.51, 19.53)	24.48 (4.52, 58.53)
Balanced Protein Suppl.	50%	0.08 (-0.07, 0.22)	1.93 (-2.06, 6.71)	44 (-47, 154)	2.53 (-2.80, 9.17)	7.58 (-8.39, 27.48)	5.13 (-4.97, 16.88)	15.37 (-14.90, 50.58)
	90%	0.15 (-0.13, 0.40)	3.48 (-3.70, 12.08)	80 (-85, 278)	4.55 (-5.04, 16.51)	13.64 (-15.09, 49.47)	5.13 (-4.97, 16.88)	15.37 (-14.90, 50.58)

References for Data Inputs

[1] Blencowe H, Krasevec J, Onis M De, et al. Articles National , regional , and worldwide estimates of low birthweight in 2015 , with trends from 2000: a systematic analysis. Lancet Glob Heal. 2019;(18):1-12.
[2] Chawanpaiboon S, Vogel JP, Moller AB, et al. Global, regional, and national estimates of levels of preterm birth in 2014: a systematic review and modelling analysis. Lancet Glob Heal. 2019;7(1):e37-e46.
[3] United National Population Division World Population Prospects 2019.
[4] Fink G, Peet E, Danaei G, et al. Schooling and wage income losses due to early-childhood growth faltering in developing countries: National, regional, and global estimates. Am J Clin Nutr. 2016;104(1):104-112.
[5] Country specific annual wage data from World Indicators Database. Average yearly wage was estimated to be 2/3 of the gross domestic product in 2010 constant US dollars and 2011 International dollars, adjusted for purchasing power parity.
[6] NCD Risk Factor Collaboration. Trends in adult body-mass index in 200 countries from 1975 to 2014: a pooled analysis of 1698 population-based measurement studies with 19·2 million participants. Lancet. 2016;387(10026):1377-1396.
[7] Stevens GA, Finucane MM, De-Regil LM, et al. Global, regional, and national trends in haemoglobin concentration and prevalence of total and severe anaemia in children and pregnant and non-pregnant women for 1995-2011: A systematic analysis of population-representative data. Lancet Glob Heal. 2013;1(1):16-25.
[8] Coverage of iron-folic acid supplementation abstracted from the most recent Demographic Health Survey or imputed based on sub-regional average. Indicator used: % women in the past five years who took iron tablets or syrup for >90 days.

Thailand

Region: Southeast Asia, East Asia, and Oceania**; Sub-region:** Southeast Asia

Low birthweight prevalence[1]: 10.5% (95% CI: 10.3, 10.8)

Preterm birth prevalence[2]: 12.7% (95% CI: 10.1, 15.6)

Number of births[3]: 3,623,000

Returns to education[4]: 10.9% (95% CI: 10.2, 11.7)

GDP per capita 2010 US$ (estimated annual wage)[5]: $5741 ($3828/year)

GDP per capita 2011 International $ (estimated annual wage)[5]: $15256 ($10171/year)

Prevalence of low BMI[6]: 8.1% (95% CI: 4.4, 13.1)

Prevalence of anemia[7]: 29.9% (95% CI: 13.2, 63.2)

Baseline coverage of IFA[8]: 46.2%

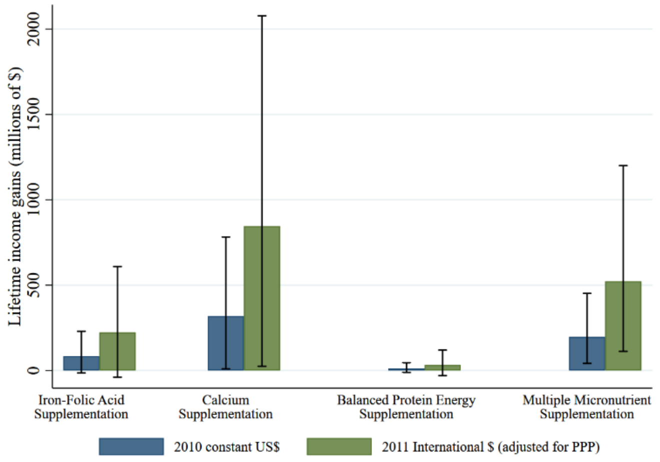

Figure: Benefits by birth cohort size for estimated gains in lifetime wages attributable to scaling up nutrition interventions to 90% target coverage

Table: Impact of maternal prenatal nutrition intervention on human capital and labour market outcomes, through improvements in low birthweight or preterm birth and schooling in Thailand.

Intervention	Target Coverage (%)	Absolute reduction in birth outcome (%)	Benefits by cohorts: School years gained (in 1000 s)	No. of additional students completing secondary school	Benefits by cohorts: Lifetime wages		Returns in lifetime earnings per child born to a targeted pregnant woman	
					in US $ millions	in International $ millions	in US $	in International $
Iron/Folic Acid Suppl.	90%	0.80 (-0.14, 1.65)	7.44 (-1.27, 20.39)	503 (-86, 1378)	83.85 (-14.74, 229.13)	222.82 (-39.17, 608.88)	25.72 (-4.52, 70.27)	68.33 (-12.01, 186.73)
Calcium Suppl.	50%	1.49 (0.34, 2.61)	15.58 (0.46, 37.99)	1053 (31, 2568)	176.75 (5.17, 434.55)	469.68 (13.74, 1154.73)	97.57 (2.86, 239.89)	259.28 (7.59, 637.44)
	90%	2.68 (0.61, 4.69)	28.05 (0.83, 68.38)	1896 (56, 4622)	318.15 (9.31, 782.19)	845.43 (24.74, 2078.52)	97.57 (2.86, 239.89)	259.28 (7.59, 637.44)
Multiple Micronutrient Suppl.	50%	0.64 (0.33, 0.92)	6.35 (1.28, 14.11)	429 (86, 954)	71.66 (14.42, 157.36)	190.42 (38.31, 418.15)	39.56 (7.96, 86.87)	105.12 (21.15, 230.83)
	90%	1.82 (0.89, 2.67)	17.47 (3.55, 39.05)	1181 (240, 2640)	196.69 (42.04, 451.52)	522.67 (111.72, 1199.81)	60.32 (12.89, 138.47)	160.29 (34.26, 367.96)
Balanced Protein Suppl.	50%	0.07 (-0.06, 0.19)	0.60 (-0.57, 2.19)	41 (-38, 148)	6.79 (-6.24, 24.91)	18.05 (-16.57, 66.18)	49.18 (-45.90, 150.73)	130.68 (-121.98, 400.53)
	90%	0.12 (-0.10, 0.35)	1.08 (-1.02, 3.94)	73 (-69, 266)	12.22 (-11.23, 44.83)	32.48 (-29.83, 119.13)	49.18 (-45.90, 150.73)	130.68 (-121.98, 400.53)

References for Data Inputs
[1] Blencowe H, Krasevec J, Onis M De, et al. Articles National , regional , and worldwide estimates of low birthweight in 2015 , with trends from 2000: a systematic analysis. Lancet Glob Heal. 2019;(18):1-12.
[2] Chawanpaiboon S, Vogel JP, Moller AB, et al. Global, regional, and national estimates of levels of preterm birth in 2014: a systematic review and modelling analysis. Lancet Glob Heal. 2019;7(1):e37-e46.
[3] United National Population Division World Population Prospects 2019.
[4] Fink G, Peet E, Danaei G, et al. Schooling and wage income losses due to early-childhood growth faltering in developing countries: National, regional, and global estimates. Am J Clin Nutr. 2016;104(1):104-112.
[5] Country specific annual wage data from World Indicators Database. Average yearly wage was estimated to be 2/3 of the gross domestic product in 2010 constant US dollars and 2011 International dollars, adjusted for purchasing power parity.
[6] NCD Risk Factor Collaboration. Trends in adult body-mass index in 200 countries from 1975 to 2014: a pooled analysis of 1698 population-based measurement studies with 19·2 million participants. Lancet. 2016;387(10026):1377-1396.
[7] Stevens GA, Finucane MM, De-Regil LM, et al. Global, regional, and national trends in haemoglobin concentration and prevalence of total and severe anaemia in children and pregnant and non-pregnant women for 1995-2011: A systematic analysis of population-representative data. Lancet Glob Heal. 2013;1(1):16-25.
[8] Coverage of iron-folic acid supplementation abstracted from the most recent Demographic Health Survey or imputed based on sub-regional average. Indicator used: % women in the past five years who took iron tablets or syrup for >90 days.

Timor-Leste

Region: Southeast Asia, East Asia, and Oceania**; Sub-region:** Southeast Asia

Low birthweight prevalence[1]: 12.0% (95% CI: 6.0, 17.9)

Preterm birth prevalence[2]: 10.4% (95% CI: 8.7, 11.9)

Number of births[3]: 187,000

Returns to education[4]: 4.8% (95% CI: 0.9, 8.7)

GDP per capita 2010 US$ (estimated annual wage)[5]: $3109 ($2073/year)

GDP per capita 2011 International $ (estimated annual wage)[5]: $7657 ($5105/year)

Prevalence of low BMI[6]: 17.9% (95% CI: 10.8, 26.1)

Prevalence of anemia[7]: 24.7% (95% CI: 18.1, 33.8)

Baseline coverage of IFA[8]: 12.8%

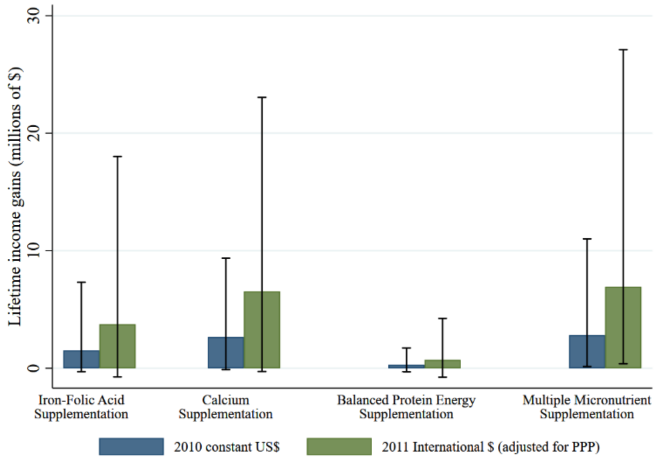

Figure: Benefits by birth cohort size for estimated gains in lifetime wages attributable to scaling up nutrition interventions to 90% target coverage

Table: Impact of maternal prenatal nutrition intervention on human capital and labour market outcomes, through improvements in low birthweight or preterm birth and schooling in Timor-Leste.

Intervention	Target Coverage (%)	Absolute reduction in birth outcome (%)	Benefits by cohorts: School years gained (in 1000 s)	No. of additional students completing secondary school	Benefits by cohorts: Lifetime wages		Returns in lifetime earnings per child born to a targeted pregnant woman	
					in US $ millions	in International $ millions	in US $	in International $
**Iron/Folic Acid Suppl.**	90%	1.45 (-0.27, 3.44)	0.66 (-0.11, 2.08)	12 (-2, 38)	1.52 (-0.30, 7.32)	3.75 (-0.73, 18.02)	9.05 (-1.77, 43.48)	22.28 (-4.35, 107.07)
**Calcium Suppl.**	50%	1.24 (0.28, 2.11)	0.64 (0.02, 1.51)	12 (0, 28)	1.48 (-0.06, 5.20)	3.63 (-0.16, 12.81)	15.79 (-0.68, 55.63)	38.87 (-1.68, 136.99)
	90%	2.24 (0.50, 3.80)	1.15 (0.04, 2.72)	21 (1, 50)	2.66 (-0.12, 9.36)	6.54 (-0.28, 23.05)	15.79 (-0.68, 55.63)	38.87 (-1.68, 136.99)
**Multiple Micronutrient Suppl.**	50%	1.28 (0.40, 2.37)	0.61 (0.09, 1.60)	11 (2, 29)	1.43 (0.08, 5.63)	3.52 (0.20, 13.87)	15.31 (0.85, 60.23)	37.70 (2.09, 148.31)
	90%	2.47 (0.72, 4.70)	1.19 (0.17, 3.12)	22 (3, 57)	2.81 (0.15, 11.01)	6.93 (0.38, 27.12)	16.72 (0.91, 65.43)	41.18 (2.23, 161.12)
**Balanced Protein Suppl.**	50%	0.16 (-0.13, 0.50)	0.07 (-0.08, 0.29)	1 (-1, 5)	0.16 (-0.17, 0.96)	0.40 (-0.42, 2.36)	10.57 (-11.58, 54.87)	26.03 (-28.52, 135.11)
	90%	0.29 (-0.24, 0.90)	0.13 (-0.14, 0.51)	2 (-3, 9)	0.29 (-0.31, 1.72)	0.72 (-0.76, 4.24)	10.57 (-11.58, 54.87)	26.03 (-28.52, 135.11)

References for Data Inputs

[1] Blencowe H, Krasevec J, Onis M De, et al. Articles National , regional , and worldwide estimates of low birthweight in 2015 , with trends from 2000: a systematic analysis. Lancet Glob Heal. 2019;(18):1-12.
[2] Chawanpaiboon S, Vogel JP, Moller AB, et al. Global, regional, and national estimates of levels of preterm birth in 2014: a systematic review and modelling analysis. Lancet Glob Heal. 2019;7(1):e37-e46.
[3] United National Population Division World Population Prospects 2019.
[4] Fink G, Peet E, Danaei G, et al. Schooling and wage income losses due to early-childhood growth faltering in developing countries: National, regional, and global estimates. Am J Clin Nutr. 2016;104(1):104-112.
[5] Country specific annual wage data from World Indicators Database. Average yearly wage was estimated to be 2/3 of the gross domestic product in 2010 constant US dollars and 2011 International dollars, adjusted for purchasing power parity.
[6] NCD Risk Factor Collaboration. Trends in adult body-mass index in 200 countries from 1975 to 2014: a pooled analysis of 1698 population-based measurement studies with 19·2 million participants. Lancet. 2016;387(10026):1377-1396.
[7] Stevens GA, Finucane MM, De-Regil LM, et al. Global, regional, and national trends in haemoglobin concentration and prevalence of total and severe anaemia in children and pregnant and non-pregnant women for 1995-2011: A systematic analysis of population-representative data. Lancet Glob Heal. 2013;1(1):16-25.
[8] Coverage of iron-folic acid supplementation abstracted from the most recent Demographic Health Survey or imputed based on sub-regional average. Indicator used: % women in the past five years who took iron tablets or syrup for >90 days.

Togo

Region: Sub-Saharan Africa**; Sub-region:** Western Sub-Saharan Africa

Low birthweight prevalence[1]: 16.1% (95% CI: 12.8, 20.3)

Preterm birth prevalence[2]: 12.0% (95% CI: 8.6, 16.7)

Number of births[3]: 1,299,000

Returns to education[4]: 6.3% (95% CI: 4.5, 8.1)

GDP per capita 2010 US$ (estimated annual wage)[5]: $630 ($420/year)

GDP per capita 2011 International $ (estimated annual wage)[5]: $1467 ($978/year)

Prevalence of low BMI[6]: 8.8% (95% CI: 4.2, 14.8)

Prevalence of anemia[7]: 58.9% (95% CI: 38.6, 71.6)

Baseline coverage of IFA[8]: 37.1%

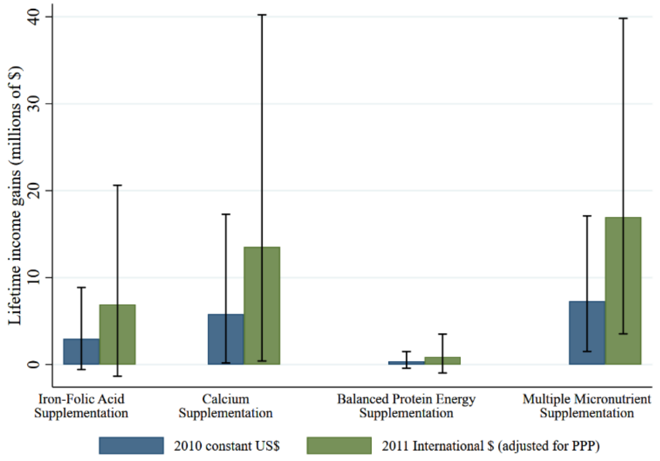

Figure: Benefits by birth cohort size for estimated gains in lifetime wages attributable to scaling up nutrition interventions to 90% target coverage

Table: Impact of maternal prenatal nutrition intervention on human capital and labour market outcomes, through improvements in low birthweight or preterm birth and schooling in Togo.

Intervention	Target Coverage (%)	Absolute reduction in birth outcome (%)	Benefits by cohorts: School years gained (in 1000 s)	No. of additional students completing secondary school	Benefits by cohorts: Lifetime wages		Returns in lifetime earnings per child born to a targeted pregnant woman	
					in US $ millions	in International $ millions	in US $	in International $
**Iron/Folic Acid Suppl.**	90%	1.46 (-0.26, 3.10)	4.24 (-0.75, 11.81)	283 (-50, 789)	2.97 (-0.57, 8.86)	6.92 (-1.33, 20.63)	2.54 (-0.49, 7.58)	5.92 (-1.14, 17.64)
**Calcium Suppl.**	50%	1.41 (0.32, 2.61)	4.51 (0.15, 12.47)	301 (10, 833)	3.23 (0.10, 9.60)	7.52 (0.23, 22.36)	4.97 (0.15, 14.79)	11.58 (0.35, 34.42)
	90%	2.54 (0.58, 4.70)	8.12 (0.27, 22.45)	543 (18, 1500)	5.81 (0.18, 17.29)	13.53 (0.41, 40.24)	4.97 (0.15, 14.79)	11.58 (0.35, 34.42)
**Multiple Micronutrient Suppl.**	50%	1.40 (0.84, 1.97)	4.39 (0.91, 9.41)	293 (61, 629)	3.08 (0.62, 7.05)	7.17 (1.44, 16.40)	4.74 (0.95, 10.85)	11.04 (2.22, 25.26)
	90%	3.36 (1.68, 5.05)	10.21 (2.09, 22.88)	682 (140, 1528)	7.29 (1.52, 17.10)	16.97 (3.53, 39.81)	6.23 (1.30, 14.63)	14.51 (3.02, 34.06)
**Balanced Protein Suppl.**	50%	0.11 (-0.10, 0.32)	0.30 (-0.29, 1.13)	20 (-19, 75)	0.21 (-0.23, 0.84)	0.50 (-0.54, 1.95)	4.10 (-3.92, 13.40)	9.55 (-9.12, 31.19)
	90%	0.20 (-0.18, 0.57)	0.55 (-0.52, 2.03)	37 (-35, 136)	0.39 (-0.41, 1.51)	0.90 (-0.96, 3.50)	4.10 (-3.92, 13.40)	9.55 (-9.12, 31.19)

References for Data Inputs

[1] Blencowe H, Krasevec J, Onis M De, et al. Articles National , regional , and worldwide estimates of low birthweight in 2015 , with trends from 2000: a systematic analysis. Lancet Glob Heal. 2019;(18):1-12.
[2] Chawanpaiboon S, Vogel JP, Moller AB, et al. Global, regional, and national estimates of levels of preterm birth in 2014: a systematic review and modelling analysis. Lancet Glob Heal. 2019;7(1):e37-e46.
[3] United National Population Division World Population Prospects 2019.
[4] Fink G, Peet E, Danaei G, et al. Schooling and wage income losses due to early-childhood growth faltering in developing countries: National, regional, and global estimates. Am J Clin Nutr. 2016;104(1):104-112.
[5] Country specific annual wage data from World Indicators Database. Average yearly wage was estimated to be 2/3 of the gross domestic product in 2010 constant US dollars and 2011 International dollars, adjusted for purchasing power parity.
[6] NCD Risk Factor Collaboration. Trends in adult body-mass index in 200 countries from 1975 to 2014: a pooled analysis of 1698 population-based measurement studies with 19·2 million participants. Lancet. 2016;387(10026):1377-1396.
[7] Stevens GA, Finucane MM, De-Regil LM, et al. Global, regional, and national trends in haemoglobin concentration and prevalence of total and severe anaemia in children and pregnant and non-pregnant women for 1995-2011: A systematic analysis of population-representative data. Lancet Glob Heal. 2013;1(1):16-25.
[8] Coverage of iron-folic acid supplementation abstracted from the most recent Demographic Health Survey or imputed based on sub-regional average. Indicator used: % women in the past five years who took iron tablets or syrup for >90 days.

Tonga

Region: Southeast Asia, East Asia, and Oceania**; Sub-region:** Oceania

Low birthweight prevalence[1]: 3.0% (95% CI: 0.3, 5.7)

Preterm birth prevalence[2]: 10.0% (95% CI: 7.9, 12.7)

Number of births[3]: 13,000

Returns to education[4]: 6.1% (95% CI: 2.7, 9.6)

GDP per capita 2010 US$ (estimated annual wage)[5]: $3899 ($2599/year)

GDP per capita 2011 International $ (estimated annual wage)[5]: $5477 ($3652/year)

Prevalence of low BMI[6]: 0.3% (95% CI: 0.1, 0.7)

Prevalence of anemia[7]: 25.8% (95% CI: 7.9, 54.8)

Baseline coverage of IFA[8]: 44.2%

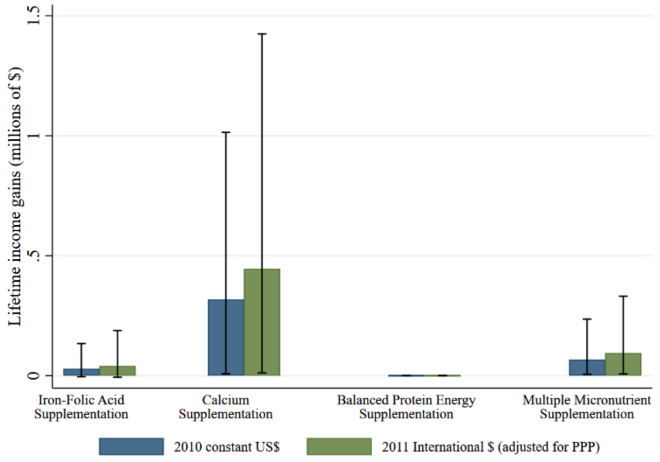

Figure: Benefits by birth cohort size for estimated gains in lifetime wages attributable to scaling up nutrition interventions to 90% target coverage

Table: Impact of maternal prenatal nutrition intervention on human capital and labour market outcomes, through improvements in low birthweight or preterm birth and schooling in Tonga.

Intervention	Target Coverage (%)	Absolute reduction in birth outcome (%)	Benefits by cohorts: School years gained (in 1000 s)	No. of additional students completing secondary school	Benefits by cohorts: Lifetime wages		Returns in lifetime earnings per child born to a targeted pregnant woman	
					in US $ millions	in International $ millions	in US $	in International $
Iron/Folic Acid Suppl.	90%	0.21 (-0.04, 0.72)	0.01 (-0.00, 0.03)	0 (-0, 1)	0.03 (-0.00, 0.13)	0.04 (-0.01, 0.19)	2.46 (-0.38, 11.45)	3.45 (-0.53, 16.09)
Calcium Suppl.	50%	1.19 (0.26, 2.15)	0.04 (0.00, 0.11)	2 (0, 5)	0.18 (0.00, 0.56)	0.25 (0.01, 0.79)	27.14 (0.70, 86.66)	38.13 (0.98, 121.74)
	90%	2.15 (0.48, 3.88)	0.08 (0.00, 0.20)	3 (0, 8)	0.32 (0.01, 1.01)	0.45 (0.01, 1.42)	27.14 (0.70, 86.66)	38.13 (0.98, 121.74)
Multiple Micronutrient Suppl.	50%	0.18 (0.03, 0.39)	0.01 (0.00, 0.02)	0 (0, 1)	0.02 (0.00, 0.08)	0.03 (0.00, 0.12)	3.78 (0.27, 12.96)	5.31 (0.38, 18.21)
	90%	0.50 (0.08, 1.13)	0.02 (0.00, 0.05)	1 (0, 2)	0.07 (0.01, 0.24)	0.09 (0.01, 0.33)	5.73 (0.46, 20.13)	8.05 (0.65, 28.28)
Balanced Protein Suppl.	50%	0.00 (-0.00, 0.00)	0.00 (-0.00, 0.00)	0 (-0, 0)	0.00 (-0.00, 0.00)	0.00 (-0.00, 0.00)	4.47 (-5.05, 23.09)	6.28 (-7.09, 32.43)
	90%	0.00 (-0.00, 0.01)	0.00 (-0.00, 0.00)	0 (-0, 0)	0.00 (-0.00, 0.00)	0.00 (-0.00, 0.00)	4.47 (-5.05, 23.09)	6.28 (-7.09, 32.43)

References for Data Inputs

[1] Blencowe H, Krasevec J, Onis M De, et al. Articles National , regional , and worldwide estimates of low birthweight in 2015 , with trends from 2000: a systematic analysis. Lancet Glob Heal. 2019;(18):1-12.
[2] Chawanpaiboon S, Vogel JP, Moller AB, et al. Global, regional, and national estimates of levels of preterm birth in 2014: a systematic review and modelling analysis. Lancet Glob Heal. 2019;7(1):e37-e46.
[3] United National Population Division World Population Prospects 2019.
[4] Fink G, Peet E, Danaei G, et al. Schooling and wage income losses due to early-childhood growth faltering in developing countries: National, regional, and global estimates. Am J Clin Nutr. 2016;104(1):104-112.
[5] Country specific annual wage data from World Indicators Database. Average yearly wage was estimated to be 2/3 of the gross domestic product in 2010 constant US dollars and 2011 International dollars, adjusted for purchasing power parity.
[6] NCD Risk Factor Collaboration. Trends in adult body-mass index in 200 countries from 1975 to 2014: a pooled analysis of 1698 population-based measurement studies with 19·2 million participants. Lancet. 2016;387(10026):1377-1396.
[7] Stevens GA, Finucane MM, De-Regil LM, et al. Global, regional, and national trends in haemoglobin concentration and prevalence of total and severe anaemia in children and pregnant and non-pregnant women for 1995-2011: A systematic analysis of population-representative data. Lancet Glob Heal. 2013;1(1):16-25.
[8] Coverage of iron-folic acid supplementation abstracted from the most recent Demographic Health Survey or imputed based on sub-regional average. Indicator used: % women in the past five years who took iron tablets or syrup for >90 days.

Tunisia

Region: North Africa and Middle East**; Sub-region:** North Africa and Middle East

Low birthweight prevalence[1]: 7.5% (95% CI: 5.9, 9.9)

Preterm birth prevalence[2]: 13.4% (95% CI: 6.3, 30.9)

Number of births[3]: 1,018,000

Returns to education[4]: 6.7% (95% CI: 4.1, 9.4)

GDP per capita 2010 US$ (estimated annual wage)[5]: $4308 ($2872/year)

GDP per capita 2011 International $ (estimated annual wage)[5]: $10861 ($7240/year)

Prevalence of low BMI[6]: 3.0% (95% CI: 1.3, 5.7)

Prevalence of anemia[7]: 29.1% (95% CI: 13.8, 52.5)

Baseline coverage of IFA[8]: 24.3%

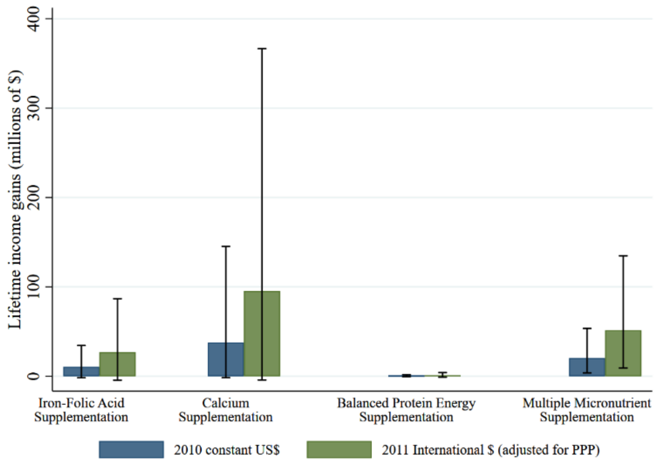

Figure: Benefits by birth cohort size for estimated gains in lifetime wages attributable to scaling up nutrition interventions to 90% target coverage

Table: Impact of maternal prenatal nutrition intervention on human capital and labour market outcomes, through improvements in low birthweight or preterm birth and schooling in Tunisia.

Intervention	Target Coverage (%)	Absolute reduction in birth outcome (%)	Benefits by cohorts: School years gained (in 1000 s)	No. of additional students completing secondary school	Benefits by cohorts: Lifetime wages		Returns in lifetime earnings per child born to a targeted pregnant woman	
					in US $ millions	in International $ millions	in US $	in International $
Iron/Folic Acid Suppl.	90%	0.80 (-0.15, 1.75)	2.13 (-0.38, 6.14)	134 (-24, 387)	10.65 (-1.73, 34.43)	26.85 (-4.35, 86.78)	11.63 (-1.88, 37.57)	29.30 (-4.75, 94.72)
Calcium Suppl.	50%	1.44 (0.01, 3.88)	4.04 (-0.19, 14.63)	255 (-12, 922)	21.01 (-0.90, 80.76)	52.95 (-2.28, 203.57)	41.27 (-1.77, 158.66)	104.04 (-4.47, 399.94)
	90%	2.59 (0.02, 6.98)	7.27 (-0.35, 26.34)	458 (-22, 1659)	37.81 (-1.62, 145.36)	95.32 (-4.10, 366.42)	41.27 (-1.77, 158.66)	104.04 (-4.47, 399.94)
Multiple Micronutrient Suppl.	50%	0.70 (0.32, 1.09)	1.86 (0.42, 4.45)	117 (27, 280)	9.58 (1.90, 24.86)	24.14 (4.78, 62.67)	18.81 (3.73, 48.84)	47.43 (9.40, 123.11)
	90%	1.49 (0.56, 2.47)	3.99 (0.76, 9.71)	252 (48, 612)	20.45 (3.67, 53.46)	51.55 (9.25, 134.75)	22.32 (4.01, 58.35)	56.27 (10.10, 147.08)
Balanced Protein Suppl.	50%	0.02 (-0.01, 0.05)	0.04 (-0.04, 0.17)	3 (-3, 11)	0.21 (-0.21, 0.92)	0.52 (-0.53, 2.32)	15.36 (-14.53, 54.50)	38.71 (-36.63, 137.38)
	90%	0.03 (-0.02, 0.10)	0.08 (-0.07, 0.31)	5 (-5, 19)	0.37 (-0.38, 1.66)	0.94 (-0.96, 4.18)	15.36 (-14.53, 54.50)	38.71 (-36.63, 137.38)

References for Data Inputs

[1] Blencowe H, Krasevec J, Onis M De, et al. Articles National , regional , and worldwide estimates of low birthweight in 2015 , with trends from 2000: a systematic analysis. Lancet Glob Heal. 2019;(18):1-12.
[2] Chawanpaiboon S, Vogel JP, Moller AB, et al. Global, regional, and national estimates of levels of preterm birth in 2014: a systematic review and modelling analysis. Lancet Glob Heal. 2019;7(1):e37-e46.
[3] United National Population Division World Population Prospects 2019.
[4] Fink G, Peet E, Danaei G, et al. Schooling and wage income losses due to early-childhood growth faltering in developing countries: National, regional, and global estimates. Am J Clin Nutr. 2016;104(1):104-112.
[5] Country specific annual wage data from World Indicators Database. Average yearly wage was estimated to be 2/3 of the gross domestic product in 2010 constant US dollars and 2011 International dollars, adjusted for purchasing power parity.
[6] NCD Risk Factor Collaboration. Trends in adult body-mass index in 200 countries from 1975 to 2014: a pooled analysis of 1698 population-based measurement studies with 19·2 million participants. Lancet. 2016;387(10026):1377-1396.
[7] Stevens GA, Finucane MM, De-Regil LM, et al. Global, regional, and national trends in haemoglobin concentration and prevalence of total and severe anaemia in children and pregnant and non-pregnant women for 1995-2011: A systematic analysis of population-representative data. Lancet Glob Heal. 2013;1(1):16-25.
[8] Coverage of iron-folic acid supplementation abstracted from the most recent Demographic Health Survey or imputed based on sub-regional average. Indicator used: % women in the past five years who took iron tablets or syrup for >90 days.

Turkey

Region: North Africa and Middle East**; Sub-region:** North Africa and Middle East

Low birthweight prevalence[1]: 11.4% (95% CI: 9.0, 14.5)

Preterm birth prevalence[2]: 12.4% (95% CI: 8.7, 17.0)

Number of births[3]: 6,588,000

Returns to education[4]: 8.5% (95% CI: 6.6, 10.3)

GDP per capita 2010 US$ (estimated annual wage)[5]: $13853 ($9235/year)

GDP per capita 2011 International $ (estimated annual wage)[5]: $23312 ($15541/year)

Prevalence of low BMI[6]: 1.5% (95% CI: 0.8, 2.5)

Prevalence of anemia[7]: 28.1% (95% CI: 13.2, 50.9)

Baseline coverage of IFA[8]: 24.3%

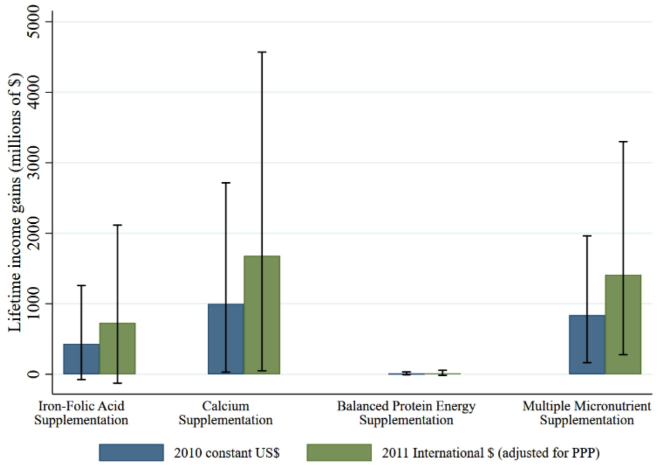

Figure: Benefits by birth cohort size for estimated gains in lifetime wages attributable to scaling up nutrition interventions to 90% target coverage

Table: Impact of maternal prenatal nutrition intervention on human capital and labour market outcomes, through improvements in low birthweight or preterm birth and schooling in Turkey.

Intervention	Target Coverage (%)	Absolute reduction in birth outcome (%)	Benefits by cohorts: School years gained (in 1000 s)	No. of additional students completing secondary school	Benefits by cohorts: Lifetime wages		Returns in lifetime earnings per child born to a targeted pregnant woman	
					in US $ millions	in International $ millions	in US $	in International $
Iron/Folic Acid Suppl.	90%	1.22 (-0.23, 2.64)	20.92 (-3.54, 57.43)	712 (-120, 1954)	433.18 (-75.12, 1257.45)	728.94 (-126.41, 2116)	73.06 (-12.67, 212.08)	122.94 (-21.32, 357)
Calcium Suppl.	50%	1.46 (0.33, 2.71)	26.62 (1.01, 70.91)	905 (34, 2412)	554.32 (16.75, 1508.46)	932.79 (28.18, 2538)	168.28 (5.08, 457.94)	283.18 (8.55, 771)
	90%	2.63 (0.59, 4.88)	47.91 (1.81, 127.63)	1630 (62, 4341)	997.77 (30.14, 2715.23)	1679.02 (50.72, 4569)	168.28 (5.08, 457.94)	283.18 (8.55, 771)
Multiple Micronutrient Suppl.	50%	1.06 (0.49, 1.67)	18.84 (4.13, 40.77)	641 (140, 1387)	393.88 (80.41, 901.25)	662.82 (135.31, 1517)	119.58 (24.41, 273.60)	201.22 (41.08, 460)
	90%	2.28 (0.86, 3.78)	39.83 (7.72, 89.23)	1355 (263, 3035)	839.67 (164.16, 1961.39)	1412.98 (276.24, 3301)	141.62 (27.69, 330.80)	238.31 (46.59, 557)
Balanced Protein Suppl.	50%	0.01 (-0.01, 0.04)	0.22 (-0.23, 0.84)	7 (-8, 29)	4.63 (-4.99, 18.51)	7.80 (-8.40, 31.15)	97.49 (-97.01, 327.80)	164.06 (-163.25, 552)
	90%	0.02 (-0.02, 0.07)	0.40 (-0.42, 1.51)	13 (-14, 52)	8.34 (-8.98, 33.32)	14.03 (-15.12, 56.07)	97.49 (-97.01, 327.80)	164.06 (-163.25, 552)

References for Data Inputs

[1] Blencowe H, Krasevec J, Onis M De, et al. Articles National , regional , and worldwide estimates of low birthweight in 2015 , with trends from 2000: a systematic analysis. Lancet Glob Heal. 2019;(18):1-12.
[2] Chawanpaiboon S, Vogel JP, Moller AB, et al. Global, regional, and national estimates of levels of preterm birth in 2014: a systematic review and modelling analysis. Lancet Glob Heal. 2019;7(1):e37-e46.
[3] United National Population Division World Population Prospects 2019.
[4] Fink G, Peet E, Danaei G, et al. Schooling and wage income losses due to early-childhood growth faltering in developing countries: National, regional, and global estimates. Am J Clin Nutr. 2016;104(1):104-112.
[5] Country specific annual wage data from World Indicators Database. Average yearly wage was estimated to be 2/3 of the gross domestic product in 2010 constant US dollars and 2011 International dollars, adjusted for purchasing power parity.
[6] NCD Risk Factor Collaboration. Trends in adult body-mass index in 200 countries from 1975 to 2014: a pooled analysis of 1698 population-based measurement studies with 19·2 million participants. Lancet. 2016;387(10026):1377-1396.
[7] Stevens GA, Finucane MM, De-Regil LM, et al. Global, regional, and national trends in haemoglobin concentration and prevalence of total and severe anaemia in children and pregnant and non-pregnant women for 1995-2011: A systematic analysis of population-representative data. Lancet Glob Heal. 2013;1(1):16-25.
[8] Coverage of iron-folic acid supplementation abstracted from the most recent Demographic Health Survey or imputed based on sub-regional average. Indicator used: % women in the past five years who took iron tablets or syrup for >90 days.

Turkmenistan

Region: Central Europe, Eastern Europe, Central Asia**; Sub-region:** Central Asia

Low birthweight prevalence[1]: 4.9% (95% CI: 3.8, 6.2)

Preterm birth prevalence[2]: 10.4% (95% CI: 8.7, 11.9)

Number of births[3]: 695,000

Returns to education[4]: 5.1% (95% CI: 2.4, 7.8)

GDP per capita 2010 US$ (estimated annual wage)[5]: $6694 ($4463/year)

GDP per capita 2011 International $ (estimated annual wage)[5]: $14992 ($9995/year)

Prevalence of low BMI[6]: 3.7% (95% CI: 1.5, 7.4)

Prevalence of anemia[7]: 29.6% (95% CI: 15.0, 52.9)

Baseline coverage of IFA[8]: 3.0%

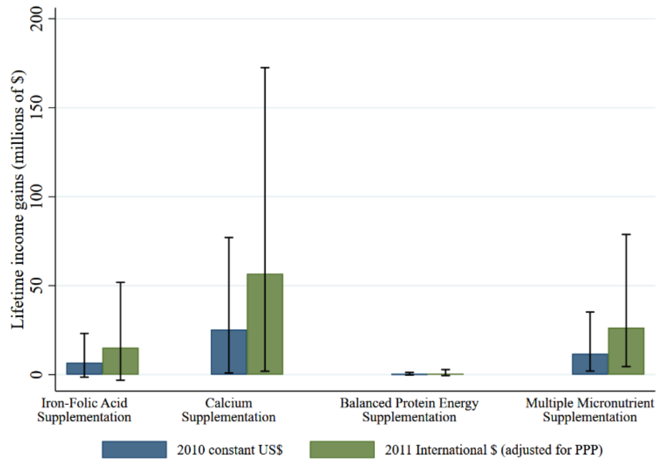

Figure: Benefits by birth cohort size for estimated gains in lifetime wages attributable to scaling up nutrition interventions to 90% target coverage

Table: Impact of maternal prenatal nutrition intervention on human capital and labour market outcomes, through improvements in low birthweight or preterm birth and schooling in Turkmenistan.

Intervention	Target Coverage (%)	Absolute reduction in birth outcome (%)	Benefits by cohorts: School years gained (in 1000 s)	No. of additional students completing secondary school	Benefits by cohorts: Lifetime wages		Returns in lifetime earnings per child born to a targeted pregnant woman	
					in US $ millions	in International $ millions	in US $	in International $
**Iron/Folic Acid Suppl.**	90%	0.68 (-0.14, 1.41)	1.15 (-0.20, 3.28)	36 (-6, 103)	6.76 (-1.39, 23.13)	15.14 (-3.10, 51.80)	10.81 (-2.22, 36.98)	24.20 (-4.96, 82.82)
**Calcium Suppl.**	50%	1.24 (0.28, 2.11)	2.34 (0.08, 5.61)	74 (2, 177)	14.07 (0.48, 42.79)	31.52 (1.07, 95.84)	40.50 (1.37, 123.14)	90.71 (3.08, 275.80)
	90%	2.23 (0.50, 3.79)	4.21 (0.14, 10.10)	133 (4, 318)	25.33 (0.86, 77.03)	56.74 (1.92, 172.51)	40.50 (1.37, 123.14)	90.71 (3.08, 275.80)
**Multiple Micronutrient Suppl.**	50%	0.61 (0.21, 1.03)	1.07 (0.19, 2.54)	34 (6, 80)	6.43 (1.13, 19.09)	14.40 (2.54, 42.75)	18.51 (3.26, 54.92)	41.45 (7.31, 123.01)
	90%	1.12 (0.38, 1.90)	1.96 (0.33, 4.67)	62 (10, 147)	11.77 (2.01, 35.17)	26.36 (4.51, 78.78)	18.82 (3.22, 56.23)	42.14 (7.21, 125.94)
**Balanced Protein Suppl.**	50%	0.01 (-0.01, 0.05)	0.02 (-0.02, 0.10)	1 (-1, 3)	0.13 (-0.13, 0.71)	0.28 (-0.28, 1.59)	11.24 (-10.35, 44.18)	25.17 (-23.19, 98.95)
	90%	0.02 (-0.02, 0.08)	0.04 (-0.04, 0.18)	1 (-1, 6)	0.23 (-0.23, 1.28)	0.50 (-0.51, 2.86)	11.24 (-10.35, 44.18)	25.17 (-23.19, 98.95)

References for Data Inputs

[1] Blencowe H, Krasevec J, Onis M De, et al. Articles National , regional , and worldwide estimates of low birthweight in 2015 , with trends from 2000: a systematic analysis. Lancet Glob Heal. 2019;(18):1-12.
[2] Chawanpaiboon S, Vogel JP, Moller AB, et al. Global, regional, and national estimates of levels of preterm birth in 2014: a systematic review and modelling analysis. Lancet Glob Heal. 2019;7(1):e37-e46.
[3] United National Population Division World Population Prospects 2019.
[4] Fink G, Peet E, Danaei G, et al. Schooling and wage income losses due to early-childhood growth faltering in developing countries: National, regional, and global estimates. Am J Clin Nutr. 2016;104(1):104-112.
[5] Country specific annual wage data from World Indicators Database. Average yearly wage was estimated to be 2/3 of the gross domestic product in 2010 constant US dollars and 2011 International dollars, adjusted for purchasing power parity.
[6] NCD Risk Factor Collaboration. Trends in adult body-mass index in 200 countries from 1975 to 2014: a pooled analysis of 1698 population-based measurement studies with 19·2 million participants. Lancet. 2016;387(10026):1377-1396.
[7] Stevens GA, Finucane MM, De-Regil LM, et al. Global, regional, and national trends in haemoglobin concentration and prevalence of total and severe anaemia in children and pregnant and non-pregnant women for 1995-2011: A systematic analysis of population-representative data. Lancet Glob Heal. 2013;1(1):16-25.
[8] Coverage of iron-folic acid supplementation abstracted from the most recent Demographic Health Survey or imputed based on sub-regional average. Indicator used: % women in the past five years who took iron tablets or syrup for >90 days.

Uganda

Region: Sub-Saharan Africa**; Sub-region:** Eastern Sub-Saharan Africa

Low birthweight prevalence[1]: 9.3% (95% CI: 3.7, 14.9)

Preterm birth prevalence[2]: 6.6% (95% CI: 4.1, 10.0)

Number of births[3]: 8,069,000

Returns to education[4]: 12.0% (95% CI: 11.2, 12.8)

GDP per capita 2010 US$ (estimated annual wage)[5]: $687 ($458/year)

GDP per capita 2011 International $ (estimated annual wage)[5]: $1750 ($1166/year)

Prevalence of low BMI[6]: 10.1% (95% CI: 5.6, 16.0)

Prevalence of anemia[7]: 36.2% (95% CI: 29.0, 45.1)

Baseline coverage of IFA[8]: 22.6%

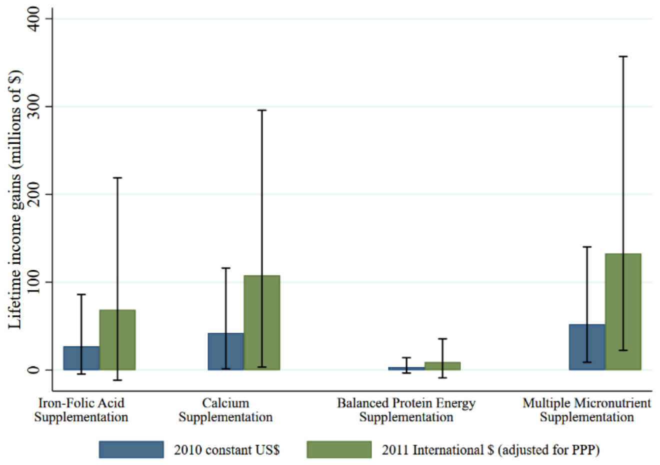

Figure: Benefits by birth cohort size for estimated gains in lifetime wages attributable to scaling up nutrition interventions to 90% target coverage

Table: Impact of maternal prenatal nutrition intervention on human capital and labour market outcomes, through improvements in low birthweight or preterm birth and schooling in Uganda.

Intervention	Target Coverage (%)	Absolute reduction in birth outcome (%)	Benefits by cohorts: School years gained (in 1000 s)	No. of additional students completing secondary school	Benefits by cohorts: Lifetime wages		Returns in lifetime earnings per child born to a targeted pregnant woman	
					in US $ millions	in International $ millions	in US $	in International $
Iron/Folic Acid Suppl.	90%	0.94 (-0.16, 2.45)	18.20 (-2.94, 58.26)	1023 (-165, 3274)	26.93 (-4.53, 85.90)	68.59 (-11.53, 218.78)	3.71 (-0.62, 11.83)	9.45 (-1.59, 30.13)
Calcium Suppl.	50%	0.76 (0.14, 1.50)	15.93 (0.51, 42.56)	895 (28, 2392)	23.52 (0.71, 64.50)	59.90 (1.81, 164.28)	5.83 (0.18, 15.99)	14.85 (0.45, 40.72)
	90%	1.36 (0.25, 2.71)	28.67 (0.91, 76.61)	1611 (51, 4305)	42.33 (1.28, 116.11)	107.81 (3.26, 295.70)	5.83 (0.18, 15.99)	14.85 (0.45, 40.72)
Multiple Micronutrient Suppl.	50%	0.91 (0.29, 1.66)	16.87 (3.14, 44.41)	948 (176, 2496)	25.28 (4.65, 66.25)	64.37 (11.83, 168.73)	6.26 (1.15, 16.42)	15.96 (2.93, 41.82)
	90%	1.90 (0.55, 3.65)	35.14 (5.98, 94.10)	1975 (336, 5288)	52.10 (8.79, 140.12)	132.70 (22.38, 356.85)	7.17 (1.21, 19.29)	18.27 (3.08, 49.14)
Balanced Protein Suppl.	50%	0.07 (-0.07, 0.22)	1.33 (-1.33, 5.09)	75 (-75, 286)	1.97 (-1.96, 7.75)	5.01 (-4.99, 19.73)	4.89 (-6.00, 18.28)	12.46 (-15.29, 46.56)
	90%	0.13 (-0.13, 0.40)	2.40 (-2.40, 9.16)	135 (-135, 515)	3.54 (-3.53, 13.95)	9.01 (-8.98, 35.52)	4.89 (-6.00, 18.28)	12.46 (-15.29, 46.56)

References for Data Inputs

[1] Blencowe H, Krasevec J, Onis M De, et al. Articles National , regional , and worldwide estimates of low birthweight in 2015 , with trends from 2000: a systematic analysis. Lancet Glob Heal. 2019;(18):1-12.
[2] Chawanpaiboon S, Vogel JP, Moller AB, et al. Global, regional, and national estimates of levels of preterm birth in 2014: a systematic review and modelling analysis. Lancet Glob Heal. 2019;7(1):e37-e46.
[3] United National Population Division World Population Prospects 2019.
[4] Fink G, Peet E, Danaei G, et al. Schooling and wage income losses due to early-childhood growth faltering in developing countries: National, regional, and global estimates. Am J Clin Nutr. 2016;104(1):104-112.
[5] Country specific annual wage data from World Indicators Database. Average yearly wage was estimated to be 2/3 of the gross domestic product in 2010 constant US dollars and 2011 International dollars, adjusted for purchasing power parity.
[6] NCD Risk Factor Collaboration. Trends in adult body-mass index in 200 countries from 1975 to 2014: a pooled analysis of 1698 population-based measurement studies with 19·2 million participants. Lancet. 2016;387(10026):1377-1396.
[7] Stevens GA, Finucane MM, De-Regil LM, et al. Global, regional, and national trends in haemoglobin concentration and prevalence of total and severe anaemia in children and pregnant and non-pregnant women for 1995-2011: A systematic analysis of population-representative data. Lancet Glob Heal. 2013;1(1):16-25.
[8] Coverage of iron-folic acid supplementation abstracted from the most recent Demographic Health Survey or imputed based on sub-regional average. Indicator used: % women in the past five years who took iron tablets or syrup for >90 days.

Ukraine

Region: Central Europe, Eastern Europe, Central Asia**; Sub-region:** Eastern Europe

Low birthweight prevalence[1]: 5.6% (95% CI: 5.4, 5.9)

Preterm birth prevalence[2]: 8.7% (95% CI: 6.3, 13.3)

Number of births[3]: 2,129,000

Returns to education[4]: 6.7% (95% CI: 5.3, 8.1)

GDP per capita 2010 US$ (estimated annual wage)[5]: $2829 ($1886/year)

GDP per capita 2011 International $ (estimated annual wage)[5]: $7465 ($4977/year)

Prevalence of low BMI[6]: 1.9% (95% CI: 0.5, 4.6)

Prevalence of anemia[7]: 24.6% (95% CI: 11.0, 45.6)

Baseline coverage of IFA[8]: 18.5%

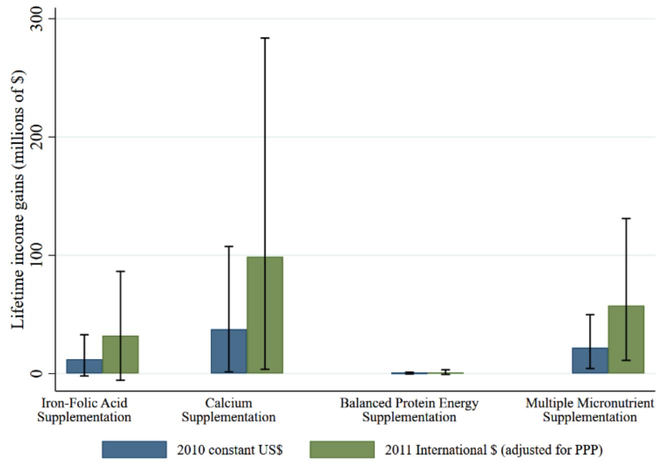

Figure: Benefits by birth cohort size for estimated gains in lifetime wages attributable to scaling up nutrition interventions to 90% target coverage

Table: Impact of maternal prenatal nutrition intervention on human capital and labour market outcomes, through improvements in low birthweight or preterm birth and schooling in Ukraine.

Intervention	Target Coverage (%)	Absolute reduction in birth outcome (%)	Benefits by cohorts: School years gained (in 1000 s)	No. of additional students completing secondary school	Benefits by cohorts: Lifetime wages		Returns in lifetime earnings per child born to a targeted pregnant woman	
					in US $ millions	in International $ millions	in US $	in International $
**Iron/Folic Acid Suppl.**	90%	0.67 (-0.12, 1.31)	3.61 (-0.67, 9.57)	28 (-5, 75)	12.12 (-2.13, 32.71)	31.99 (-5.61, 86.33)	6.33 (-1.11, 17.07)	16.70 (-2.93, 45.05)
**Calcium Suppl.**	50%	1.01 (0.21, 1.89)	6.13 (0.20, 17.08)	48 (2, 133)	20.80 (0.76, 59.70)	54.88 (2.01, 157.55)	19.54 (0.72, 56.09)	51.55 (1.89, 148.00)
	90%	1.82 (0.39, 3.39)	11.03 (0.35, 30.75)	86 (3, 240)	37.43 (1.37, 107.47)	98.78 (3.63, 283.59)	19.54 (0.72, 56.09)	51.55 (1.89, 148.00)
**Multiple Micronutrient Suppl.**	50%	0.57 (0.25, 0.86)	3.24 (0.66, 6.91)	25 (5, 54)	10.59 (2.24, 24.28)	27.95 (5.92, 64.08)	9.95 (2.11, 22.81)	26.26 (5.56, 60.19)
	90%	1.16 (0.47, 1.80)	6.55 (1.23, 14.07)	51 (10, 110)	21.81 (4.22, 49.67)	57.54 (11.13, 131.07)	11.38 (2.20, 25.92)	30.03 (5.81, 68.41)
**Balanced Protein Suppl.**	50%	0.01 (-0.01, 0.03)	0.04 (-0.05, 0.19)	0 (-0, 1)	0.13 (-0.17, 0.66)	0.34 (-0.46, 1.74)	7.80 (-7.35, 25.23)	20.58 (-19.40, 66.58)
	90%	0.01 (-0.01, 0.05)	0.07 (-0.09, 0.33)	1 (-1, 3)	0.23 (-0.31, 1.19)	0.61 (-0.82, 3.13)	7.80 (-7.35, 25.23)	20.58 (-19.40, 66.58)

References for Data Inputs

[1] Blencowe H, Krasevec J, Onis M De, et al. Articles National , regional , and worldwide estimates of low birthweight in 2015 , with trends from 2000: a systematic analysis. Lancet Glob Heal. 2019;(18):1-12.
[2] Chawanpaiboon S, Vogel JP, Moller AB, et al. Global, regional, and national estimates of levels of preterm birth in 2014: a systematic review and modelling analysis. Lancet Glob Heal. 2019;7(1):e37-e46.
[3] United National Population Division World Population Prospects 2019.
[4] Fink G, Peet E, Danaei G, et al. Schooling and wage income losses due to early-childhood growth faltering in developing countries: National, regional, and global estimates. Am J Clin Nutr. 2016;104(1):104-112.
[5] Country specific annual wage data from World Indicators Database. Average yearly wage was estimated to be 2/3 of the gross domestic product in 2010 constant US dollars and 2011 International dollars, adjusted for purchasing power parity.
[6] NCD Risk Factor Collaboration. Trends in adult body-mass index in 200 countries from 1975 to 2014: a pooled analysis of 1698 population-based measurement studies with 19·2 million participants. Lancet. 2016;387(10026):1377-1396.
[7] Stevens GA, Finucane MM, De-Regil LM, et al. Global, regional, and national trends in haemoglobin concentration and prevalence of total and severe anaemia in children and pregnant and non-pregnant women for 1995-2011: A systematic analysis of population-representative data. Lancet Glob Heal. 2013;1(1):16-25.
[8] Coverage of iron-folic acid supplementation abstracted from the most recent Demographic Health Survey or imputed based on sub-regional average. Indicator used: % women in the past five years who took iron tablets or syrup for >90 days.

Uzbekistan

Region: Central Europe, Eastern Europe, Central Asia**; Sub-region:** Central Asia

Low birthweight prevalence[1]: 5.3% (95% CI: 4.1, 6.7)

Preterm birth prevalence[2]: 10.4% (95% CI: 8.7, 11.9)

Number of births[3]: 3,513,000

Returns to education[4]: 5.1% (95% CI: 2.4, 7.8)

GDP per capita 2010 US$ (estimated annual wage)[5]: $1831 ($1221/year)

GDP per capita 2011 International $ (estimated annual wage)[5]: $5639 ($3759/year)

Prevalence of low BMI[6]: 3.9% (95% CI: 1.6, 7.7)

Prevalence of anemia[7]: 35.3% (95% CI: 19.6, 60.2)

Baseline coverage of IFA[8]: 3.0%

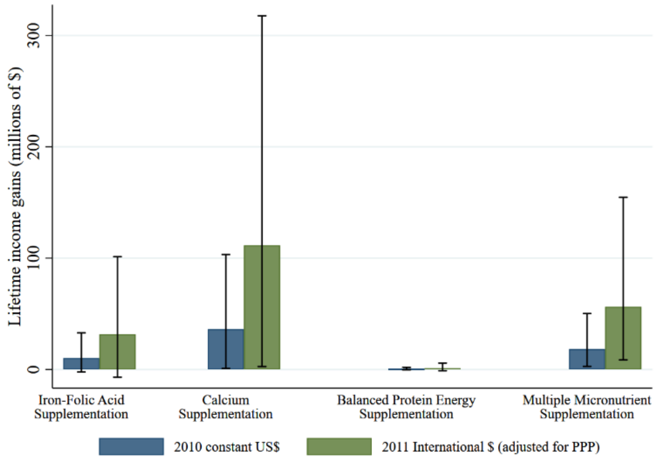

Figure: Benefits by birth cohort size for estimated gains in lifetime wages attributable to scaling up nutrition interventions to 90% target coverage

Table: Impact of maternal prenatal nutrition intervention on human capital and labour market outcomes, through improvements in low birthweight or preterm birth and schooling in Uzbekistan.

Intervention	Target Coverage (%)	Absolute reduction in birth outcome (%)	Benefits by cohorts: School years gained (in 1000 s)	No. of additional students completing secondary school	Benefits by cohorts: Lifetime wages		Returns in lifetime earnings per child born to a targeted pregnant woman	
					in US $ millions	in International $ millions	in US $	in International $
Iron/Folic Acid Suppl.	90%	0.72 (-0.14, 1.48)	6.51 (-1.31, 17.97)	205 (-41, 566)	10.26 (-2.25, 32.89)	31.59 (-6.92, 101.28)	3.24 (-0.71, 10.40)	9.99 (-2.19, 32.03)
Calcium Suppl.	50%	1.25 (0.29, 2.05)	12.27 (0.34, 29.80)	386 (11, 939)	20.11 (0.47, 57.33)	61.91 (1.46, 176.54)	11.45 (0.27, 32.64)	35.25 (0.83, 100.51)
	90%	2.25 (0.51, 3.69)	22.08 (0.62, 53.64)	695 (19, 1690)	36.19 (0.85, 103.20)	111.44 (2.63, 317.77)	11.45 (0.27, 32.64)	35.25 (0.83, 100.51)
Multiple Micronutrient Suppl.	50%	0.66 (0.25, 1.08)	6.18 (1.13, 13.92)	195 (35, 438)	9.97 (1.53, 27.40)	30.70 (4.72, 84.39)	5.68 (0.87, 15.60)	17.48 (2.69, 48.04)
	90%	1.21 (0.45, 1.98)	11.28 (2.06, 25.59)	355 (65, 806)	18.28 (2.80, 50.23)	56.28 (8.61, 154.68)	5.78 (0.88, 15.89)	17.80 (2.72, 48.92)
Balanced Protein Suppl.	50%	0.01 (-0.01, 0.05)	0.13 (-0.12, 0.57)	4 (-4, 18)	0.20 (-0.20, 1.04)	0.62 (-0.62, 3.20)	3.40 (-3.40, 12.82)	10.46 (-10.47, 39.48)
	90%	0.03 (-0.02, 0.09)	0.23 (-0.22, 1.02)	7 (-7, 32)	0.36 (-0.36, 1.87)	1.11 (-1.11, 5.76)	3.40 (-3.40, 12.82)	10.46 (-10.47, 39.48)

References for Data Inputs
[1] Blencowe H, Krasevec J, Onis M De, et al. Articles National , regional , and worldwide estimates of low birthweight in 2015 , with trends from 2000: a systematic analysis. Lancet Glob Heal. 2019;(18):1-12.
[2] Chawanpaiboon S, Vogel JP, Moller AB, et al. Global, regional, and national estimates of levels of preterm birth in 2014: a systematic review and modelling analysis. Lancet Glob Heal. 2019;7(1):e37-e46.
[3] United National Population Division World Population Prospects 2019.
[4] Fink G, Peet E, Danaei G, et al. Schooling and wage income losses due to early-childhood growth faltering in developing countries: National, regional, and global estimates. Am J Clin Nutr. 2016;104(1):104-112.
[5] Country specific annual wage data from World Indicators Database. Average yearly wage was estimated to be 2/3 of the gross domestic product in 2010 constant US dollars and 2011 International dollars, adjusted for purchasing power parity.
[6] NCD Risk Factor Collaboration. Trends in adult body-mass index in 200 countries from 1975 to 2014: a pooled analysis of 1698 population-based measurement studies with 19·2 million participants. Lancet. 2016;387(10026):1377-1396.
[7] Stevens GA, Finucane MM, De-Regil LM, et al. Global, regional, and national trends in haemoglobin concentration and prevalence of total and severe anaemia in children and pregnant and non-pregnant women for 1995-2011: A systematic analysis of population-representative data. Lancet Glob Heal. 2013;1(1):16-25.
[8] Coverage of iron-folic acid supplementation abstracted from the most recent Demographic Health Survey or imputed based on sub-regional average. Indicator used: % women in the past five years who took iron tablets or syrup for >90 days.

Vanuatu

Region: Southeast Asia, East Asia, and Oceania**; Sub-region:** Oceania

Low birthweight prevalence[1]: 10.9% (95% CI: 8.6, 13.9)

Preterm birth prevalence[2]: 10.0% (95% CI: 7.9, 12.7)

Number of births[3]: 43,000

Returns to education[4]: 6.1% (95% CI: 2.7, 9.6)

GDP per capita 2010 US$ (estimated annual wage)[5]: $2781 ($1854/year)

GDP per capita 2011 International $ (estimated annual wage)[5]: $2953 ($1969/year)

Prevalence of low BMI[6]: 2.2% (95% CI: 0.8, 4.7)

Prevalence of anemia[7]: 27.3% (95% CI: 14.9, 46.8)

Baseline coverage of IFA[8]: 44.2%

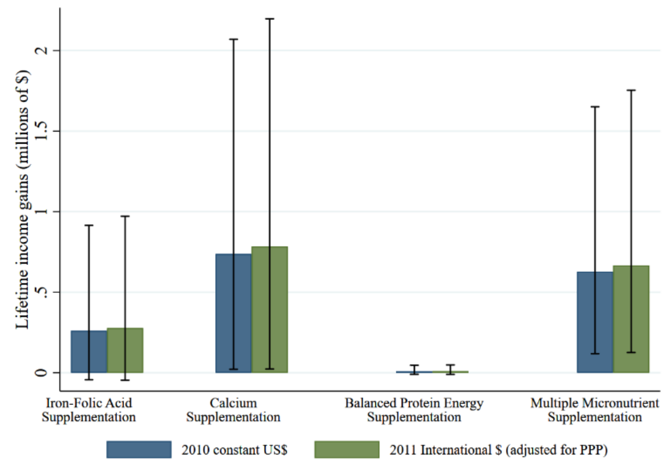

Figure: Benefits by birth cohort size for estimated gains in lifetime wages attributable to scaling up nutrition interventions to 90% target coverage

Table: Impact of maternal prenatal nutrition intervention on human capital and labour market outcomes, through improvements in low birthweight or preterm birth and schooling in Vanuatu.

Intervention	Target Coverage (%)	Absolute reduction in birth outcome (%)	Benefits by cohorts: School years gained (in 1000 s)	No. of additional students completing secondary school	Benefits by cohorts: Lifetime wages		Returns in lifetime earnings per child born to a targeted pregnant woman	
					in US $ millions	in International $ millions	in US $	in International $
Iron/Folic Acid Suppl.	90%	0.86 (-0.16, 1.89)	0.09 (-0.02, 0.27)	2 (-0, 6)	0.26 (-0.04, 0.91)	0.28 (-0.05, 0.97)	6.75 (-1.12, 23.63)	7.17 (-1.19, 25.09)
Calcium Suppl.	50%	1.18 (0.24, 2.09)	0.14 (0.00, 0.35)	3 (0, 8)	0.41 (0.01, 1.15)	0.44 (0.01, 1.22)	19.06 (0.57, 53.47)	20.24 (0.60, 56.78)
	90%	2.13 (0.44, 3.76)	0.25 (0.01, 0.63)	6 (0, 14)	0.74 (0.02, 2.07)	0.78 (0.02, 2.20)	19.06 (0.57, 53.47)	20.24 (0.60, 56.78)
Multiple Micronutrient Suppl.	50%	0.69 (0.38, 0.99)	0.08 (0.02, 0.17)	2 (0, 4)	0.23 (0.04, 0.59)	0.25 (0.05, 0.63)	10.87 (2.00, 27.62)	11.54 (2.12, 29.34)
	90%	1.89 (0.83, 2.99)	0.21 (0.05, 0.48)	5 (1, 11)	0.63 (0.12, 1.65)	0.67 (0.13, 1.75)	16.22 (3.06, 42.66)	17.22 (3.25, 45.30)
Balanced Protein Suppl.	50%	0.02 (-0.02, 0.06)	0.00 (-0.00, 0.01)	0 (-0, 0)	0.01 (-0.01, 0.03)	0.01 (-0.01, 0.03)	12.52 (-11.48, 44.17)	13.30 (-12.19, 46.90)
	90%	0.03 (-0.03, 0.11)	0.00 (-0.00, 0.01)	0 (-0, 0)	0.01 (-0.01, 0.05)	0.01 (-0.01, 0.05)	12.52 (-11.48, 44.17)	13.30 (-12.19, 46.90)

References for Data Inputs

[1] Blencowe H, Krasevec J, Onis M De, et al. Articles National , regional , and worldwide estimates of low birthweight in 2015 , with trends from 2000: a systematic analysis. Lancet Glob Heal. 2019;(18):1-12.
[2] Chawanpaiboon S, Vogel JP, Moller AB, et al. Global, regional, and national estimates of levels of preterm birth in 2014: a systematic review and modelling analysis. Lancet Glob Heal. 2019;7(1):e37-e46.
[3] United National Population Division World Population Prospects 2019.
[4] Fink G, Peet E, Danaei G, et al. Schooling and wage income losses due to early-childhood growth faltering in developing countries: National, regional, and global estimates. Am J Clin Nutr. 2016;104(1):104-112.
[5] Country specific annual wage data from World Indicators Database. Average yearly wage was estimated to be 2/3 of the gross domestic product in 2010 constant US dollars and 2011 International dollars, adjusted for purchasing power parity.
[6] NCD Risk Factor Collaboration. Trends in adult body-mass index in 200 countries from 1975 to 2014: a pooled analysis of 1698 population-based measurement studies with 19·2 million participants. Lancet. 2016;387(10026):1377-1396.
[7] Stevens GA, Finucane MM, De-Regil LM, et al. Global, regional, and national trends in haemoglobin concentration and prevalence of total and severe anaemia in children and pregnant and non-pregnant women for 1995-2011: A systematic analysis of population-representative data. Lancet Glob Heal. 2013;1(1):16-25.
[8] Coverage of iron-folic acid supplementation abstracted from the most recent Demographic Health Survey or imputed based on sub-regional average. Indicator used: % women in the past five years who took iron tablets or syrup for >90 days.

Venezuela

Region: Latin America and Caribbean**; Sub-region:** Central Latin America

Low birthweight prevalence[1]: 9.1% (95% CI: 8.9, 9.3)

Preterm birth prevalence[2]: 9.7% (95% CI: 7.7, 12.1)

Number of births[3]: 2,638,000

Returns to education[4]: 8.5% (95% CI: 7.6, 9.4)

GDP per capita 2010 US$ (estimated annual wage)[5]: $13825 ($9217/year)

GDP per capita 2011 International $ (estimated annual wage)[5]: $16887 ($11258/year)

Prevalence of low BMI[6]: 1.6% (95% CI: 0.7, 3.0)

Prevalence of anemia[7]: 27.4% (95% CI: 12.2, 51.2)

Baseline coverage of IFA[8]: 33.3%

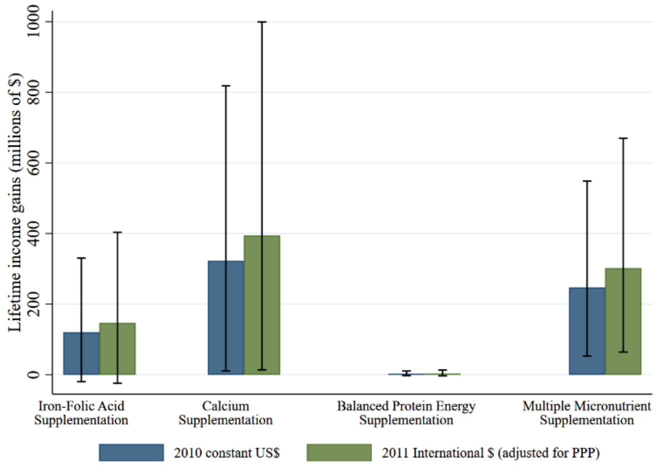

Figure: Benefits by birth cohort size for estimated gains in lifetime wages attributable to scaling up nutrition interventions to 90% target coverage

Table: Impact of maternal prenatal nutrition intervention on human capital and labour market outcomes, through improvements in low birthweight or preterm birth and schooling in Venezuela.

Intervention	Target Coverage (%)	Absolute reduction in birth outcome (%)	Benefits by cohorts: School years gained (in 1000 s)	No. of additional students completing secondary school	Benefits by cohorts: Lifetime wages		Returns in lifetime earnings per child born to a targeted pregnant woman	
					in US $ millions	in International $ millions	in US $	in International $
Iron/Folic Acid Suppl.	90%	0.88 (-0.16, 1.78)	5.70 (-1.00, 15.48)	124 (-22, 338)	120.11 (-19.54, 330.43)	146.71 (-23.87, 403.61)	50.59 (-8.23, 139.18)	61.79 (-10.05, 170.00)
Calcium Suppl.	50%	1.15 (0.26, 2.07)	8.37 (0.29, 21.73)	182 (6, 474)	179.32 (6.20, 454.56)	219.03 (7.57, 555.23)	135.95 (4.70, 344.62)	166.06 (5.74, 420.94)
	90%	2.06 (0.46, 3.72)	15.06 (0.53, 39.12)	328 (12, 853)	322.77 (11.15, 818.21)	394.26 (13.62, 999.41)	135.95 (4.70, 344.62)	166.06 (5.74, 420.94)
Multiple Micronutrient Suppl.	50%	0.73 (0.39, 1.01)	4.99 (1.19, 10.50)	109 (26, 229)	105.03 (24.31, 222.46)	128.28 (29.69, 271.73)	79.63 (18.43, 168.66)	97.26 (22.51, 206.01)
	90%	1.73 (0.73, 2.57)	11.63 (2.32, 25.34)	253 (51, 552)	247.09 (52.57, 548.38)	301.82 (64.22, 669.82)	104.08 (22.14, 230.97)	127.12 (27.05, 282.13)
Balanced Protein Suppl.	50%	0.01 (-0.01, 0.03)	0.07 (-0.07, 0.29)	2 (-2, 6)	1.50 (-1.47, 5.95)	1.83 (-1.79, 7.27)	78.73 (-74.29, 234.06)	96.17 (-90.74, 285.89)
	90%	0.02 (-0.02, 0.06)	0.13 (-0.13, 0.52)	3 (-3, 11)	2.69 (-2.64, 10.71)	3.29 (-3.23, 13.08)	78.73 (-74.29, 234.06)	96.17 (-90.74, 285.89)

References for Data Inputs

[1] Blencowe H, Krasevec J, Onis M De, et al. Articles National , regional , and worldwide estimates of low birthweight in 2015 , with trends from 2000: a systematic analysis. Lancet Glob Heal. 2019;(18):1-12.
[2] Chawanpaiboon S, Vogel JP, Moller AB, et al. Global, regional, and national estimates of levels of preterm birth in 2014: a systematic review and modelling analysis. Lancet Glob Heal. 2019;7(1):e37-e46.
[3] United National Population Division World Population Prospects 2019.
[4] Fink G, Peet E, Danaei G, et al. Schooling and wage income losses due to early-childhood growth faltering in developing countries: National, regional, and global estimates. Am J Clin Nutr. 2016;104(1):104-112.
[5] Country specific annual wage data from World Indicators Database. Average yearly wage was estimated to be 2/3 of the gross domestic product in 2010 constant US dollars and 2011 International dollars, adjusted for purchasing power parity.
[6] NCD Risk Factor Collaboration. Trends in adult body-mass index in 200 countries from 1975 to 2014: a pooled analysis of 1698 population-based measurement studies with 19·2 million participants. Lancet. 2016;387(10026):1377-1396.
[7] Stevens GA, Finucane MM, De-Regil LM, et al. Global, regional, and national trends in haemoglobin concentration and prevalence of total and severe anaemia in children and pregnant and non-pregnant women for 1995-2011: A systematic analysis of population-representative data. Lancet Glob Heal. 2013;1(1):16-25.
[8] Coverage of iron-folic acid supplementation abstracted from the most recent Demographic Health Survey or imputed based on sub-regional average. Indicator used: % women in the past five years who took iron tablets or syrup for >90 days.

Vietnam

Region: Southeast Asia, East Asia, and Oceania**; Sub-region:** Southeast Asia

Low birthweight prevalence[1]: 8.2% (95% CI: 5.8, 10.6)

Preterm birth prevalence[2]: 6.5% (95% CI: 4.3, 9.3)

Number of births[3]: 8,050,000

Returns to education[4]: 4.8% (95% CI: 3.7, 5.9)

GDP per capita 2010 US$ (estimated annual wage)[5]: $1667 ($1111/year)

GDP per capita 2011 International $ (estimated annual wage)[5]: $5608 ($3739/year)

Prevalence of low BMI[6]: 18.4% (95% CI: 12.8, 24.4)

Prevalence of anemia[7]: 24.4% (95% CI: 13.9, 38.4)

Baseline coverage of IFA[8]: 46.2%

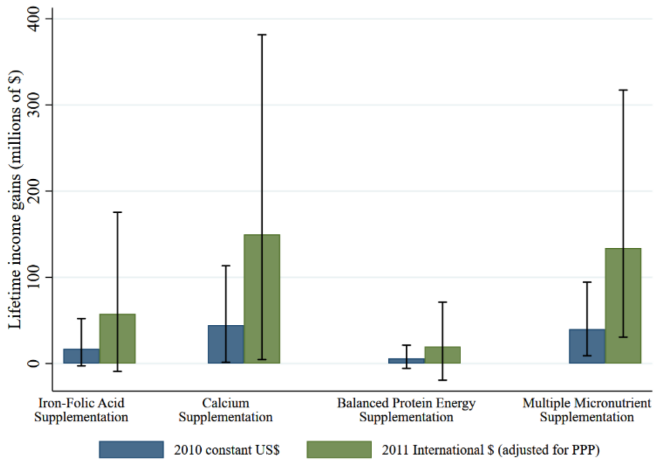

Figure: Benefits by birth cohort size for estimated gains in lifetime wages attributable to scaling up nutrition interventions to 90% target coverage

Table: Impact of maternal prenatal nutrition intervention on human capital and labour market outcomes, through improvements in low birthweight or preterm birth and schooling in Vietnam.

Intervention	Target Coverage (%)	Absolute reduction in birth outcome (%)	Benefits by cohorts: School years gained (in 1000 s)	No. of additional students completing secondary school	Benefits by cohorts: Lifetime wages		Returns in lifetime earnings per child born to a targeted pregnant woman	
					in US $ millions	in International $ millions	in US $	in International $
Iron/Folic Acid Suppl.	90%	0.60 (-0.12, 1.35)	12.02 (-1.85, 36.34)	873 (-134, 2638)	17.12 (-2.75, 52.15)	57.61 (-9.26, 175.42)	2.36 (-0.38, 7.20)	7.95 (-1.28, 24.21)
Calcium Suppl.	50%	0.76 (0.16, 1.45)	17.25 (0.54, 43.05)	1252 (39, 3125)	24.72 (0.75, 62.98)	83.17 (2.53, 211.86)	6.14 (0.19, 15.65)	20.66 (0.63, 52.64)
	90%	1.37 (0.28, 2.60)	31.05 (0.98, 77.48)	2254 (71, 5625)	44.50 (1.35, 113.36)	149.70 (4.55, 381.35)	6.14 (0.19, 15.65)	20.66 (0.63, 52.64)
Multiple Micronutrient Suppl.	50%	0.46 (0.24, 0.73)	10.02 (2.26, 22.70)	727 (164, 1648)	14.29 (2.92, 34.03)	48.07 (9.81, 114.47)	3.55 (0.72, 8.45)	11.94 (2.44, 28.44)
	90%	1.35 (0.60, 2.11)	28.31 (6.30, 65.86)	2056 (457, 4781)	39.80 (9.09, 94.29)	133.90 (30.57, 317.20)	5.49 (1.25, 13.01)	18.48 (4.22, 43.78)
Balanced Protein Suppl.	50%	0.12 (-0.10, 0.31)	2.30 (-2.18, 7.72)	167 (-158, 560)	3.24 (-3.19, 11.74)	10.90 (-10.73, 39.48)	4.53 (-4.43, 15.77)	15.24 (-14.89, 53.04)
	90%	0.22 (-0.19, 0.56)	4.14 (-3.93, 13.90)	301 (-285, 1009)	5.83 (-5.74, 21.12)	19.62 (-19.32, 71.06)	4.53 (-4.43, 15.77)	15.24 (-14.89, 53.04)

References for Data Inputs

[1] Blencowe H, Krasevec J, Onis M De, et al. Articles National , regional , and worldwide estimates of low birthweight in 2015 , with trends from 2000: a systematic analysis. Lancet Glob Heal. 2019;(18):1-12.
[2] Chawanpaiboon S, Vogel JP, Moller AB, et al. Global, regional, and national estimates of levels of preterm birth in 2014: a systematic review and modelling analysis. Lancet Glob Heal. 2019;7(1):e37-e46.
[3] United National Population Division World Population Prospects 2019.
[4] Fink G, Peet E, Danaei G, et al. Schooling and wage income losses due to early-childhood growth faltering in developing countries: National, regional, and global estimates. Am J Clin Nutr. 2016;104(1):104-112.
[5] Country specific annual wage data from World Indicators Database. Average yearly wage was estimated to be 2/3 of the gross domestic product in 2010 constant US dollars and 2011 International dollars, adjusted for purchasing power parity.
[6] NCD Risk Factor Collaboration. Trends in adult body-mass index in 200 countries from 1975 to 2014: a pooled analysis of 1698 population-based measurement studies with 19·2 million participants. Lancet. 2016;387(10026):1377-1396.
[7] Stevens GA, Finucane MM, De-Regil LM, et al. Global, regional, and national trends in haemoglobin concentration and prevalence of total and severe anaemia in children and pregnant and non-pregnant women for 1995-2011: A systematic analysis of population-representative data. Lancet Glob Heal. 2013;1(1):16-25.
[8] Coverage of iron-folic acid supplementation abstracted from the most recent Demographic Health Survey or imputed based on sub-regional average. Indicator used: % women in the past five years who took iron tablets or syrup for >90 days.

Yemen

Region: North Africa and Middle East**; Sub-region:** North Africa and Middle East

Low birthweight prevalence[1]: 23.0% (95% CI: 19.0, 27.0)

Preterm birth prevalence[2]: 10.4% (95% CI: 8.7, 11.9)

Number of births[3]: 4,324,000

Returns to education[4]: 6.7% (95% CI: 4.1, 9.4)

GDP per capita 2010 US$ (estimated annual wage)[5]: $909 ($606/year)

GDP per capita 2011 International $ (estimated annual wage)[5]: $3109 ($2073/year)

Prevalence of low BMI[6]: 7.8% (95% CI: 4.2, 12.8)

Prevalence of anemia[7]: 36.7% (95% CI: 18.6, 60.6)

Baseline coverage of IFA[8]: 5.5%

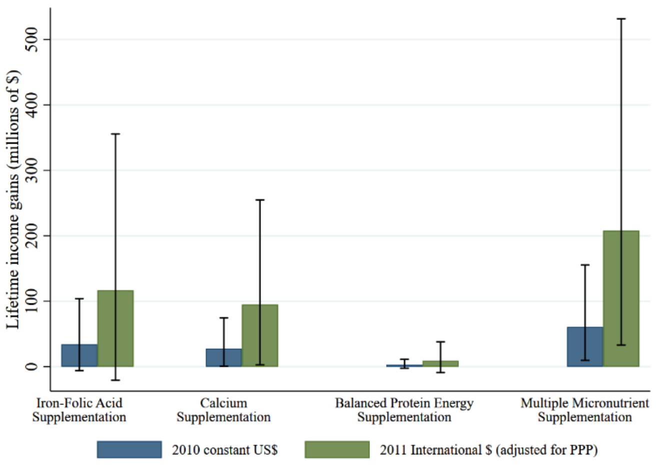

Figure: Benefits by birth cohort size for estimated gains in lifetime wages attributable to scaling up nutrition interventions to 90% target coverage

Table: Impact of maternal prenatal nutrition intervention on human capital and labour market outcomes, through improvements in low birthweight or preterm birth and schooling in Yemen.

Intervention	Target Coverage (%)	Absolute reduction in birth outcome (%)	Benefits by cohorts: School years gained (in 1000 s)	No. of additional students completing secondary school	Benefits by cohorts: Lifetime wages		Returns in lifetime earnings per child born to a targeted pregnant woman	
					in US $ millions	in International $ millions	in US $	in International $
**Iron/Folic Acid Suppl.**	90%	3.08 (-0.55, 6.24)	32.25 (-5.55, 83.64)	1206 (-208, 3128)	34.14 (-6.04, 103.99)	116.79 (-20.66, 355.75)	8.77 (-1.55, 26.72)	30.01 (-5.31, 91.41)
**Calcium Suppl.**	50%	1.25 (0.28, 2.09)	14.17 (0.44, 34.28)	530 (16, 1282)	15.43 (0.46, 41.39)	52.77 (1.57, 141.58)	7.13 (0.21, 19.14)	24.41 (0.73, 65.49)
	90%	2.25 (0.51, 3.77)	25.51 (0.79, 61.70)	954 (29, 2308)	27.77 (0.83, 74.50)	94.98 (2.83, 254.85)	7.13 (0.21, 19.14)	24.41 (0.73, 65.49)
**Multiple Micronutrient Suppl.**	50%	2.80 (1.12, 4.46)	30.21 (5.65, 69.94)	1130 (211, 2616)	32.74 (5.53, 83.51)	112.01 (18.93, 285.69)	15.14 (2.56, 38.63)	51.81 (8.75, 132.14)
	90%	5.21 (2.00, 8.32)	56.18 (10.22, 129.81)	2101 (382, 4855)	60.79 (9.63, 155.46)	207.97 (32.96, 531.83)	15.62 (2.48, 39.95)	53.44 (8.47, 136.66)
**Balanced Protein Suppl.**	50%	0.14 (-0.11, 0.40)	1.40 (-1.32, 4.94)	52 (-49, 185)	1.49 (-1.46, 6.17)	5.09 (-4.98, 21.10)	9.44 (-9.82, 33.13)	32.28 (-33.59, 113.33)
	90%	0.25 (-0.21, 0.71)	2.53 (-2.38, 8.90)	94 (-89, 333)	2.68 (-2.62, 11.10)	9.16 (-8.97, 37.97)	9.44 (-9.82, 33.13)	32.28 (-33.59, 113.33)

References for Data Inputs

[1] Blencowe H, Krasevec J, Onis M De, et al. Articles National , regional , and worldwide estimates of low birthweight in 2015 , with trends from 2000: a systematic analysis. Lancet Glob Heal. 2019;(18):1-12.

[2] Chawanpaiboon S, Vogel JP, Moller AB, et al. Global, regional, and national estimates of levels of preterm birth in 2014: a systematic review and modelling analysis. Lancet Glob Heal. 2019;7(1):e37-e46.

[3] United National Population Division World Population Prospects 2019.

[4] Fink G, Peet E, Danaei G, et al. Schooling and wage income losses due to early-childhood growth faltering in developing countries: National, regional, and global estimates. Am J Clin Nutr. 2016;104(1):104-112.

[5] Country specific annual wage data from World Indicators Database. Average yearly wage was estimated to be 2/3 of the gross domestic product in 2010 constant US dollars and 2011 International dollars, adjusted for purchasing power parity.

[6] NCD Risk Factor Collaboration. Trends in adult body-mass index in 200 countries from 1975 to 2014: a pooled analysis of 1698 population-based measurement studies with 19·2 million participants. Lancet. 2016;387(10026):1377-1396.

[7] Stevens GA, Finucane MM, De-Regil LM, et al. Global, regional, and national trends in haemoglobin concentration and prevalence of total and severe anaemia in children and pregnant and non-pregnant women for 1995-2011: A systematic analysis of population-representative data. Lancet Glob Heal. 2013;1(1):16-25.

[8] Coverage of iron-folic acid supplementation abstracted from the most recent Demographic Health Survey or imputed based on sub-regional average. Indicator used: % women in the past five years who took iron tablets or syrup for >90 days.

Zambia

Region: Sub-Saharan Africa**; Sub-region:** Eastern Sub-Saharan Africa

Low birthweight prevalence[1]: 11.6% (95% CI: 9.2, 14.8)

Preterm birth prevalence[2]: 12.0% (95% CI: 8.6, 16.7)

Number of births[3]: 3,108,000

Returns to education[4]: 11.3% (95% CI: 9.7, 12.9)

GDP per capita 2010 US$ (estimated annual wage)[5]: $1641 ($1094/year)

GDP per capita 2011 International $ (estimated annual wage)[5]: $3678 ($2452/year)

Prevalence of low BMI[6]: 8.6% (95% CI: 4.5, 14.1)

Prevalence of anemia[7]: 36.4% (95% CI: 21.1, 54.4)

Baseline coverage of IFA[8]: 59.1%

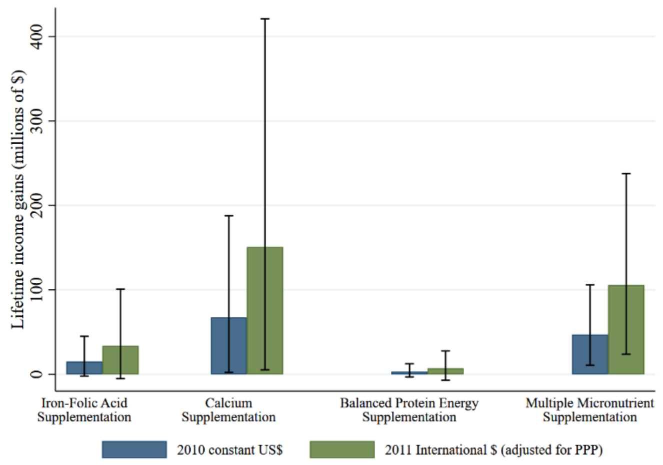

Figure: Benefits by birth cohort size for estimated gains in lifetime wages attributable to scaling up nutrition interventions to 90% target coverage

Table: Impact of maternal prenatal nutrition intervention on human capital and labour market outcomes, through improvements in low birthweight or preterm birth and schooling in Zambia.

Intervention	Target Coverage (%)	Absolute reduction in birth outcome (%)	Benefits by cohorts: School years gained (in 1000 s)	No. of additional students completing secondary school	Benefits by cohorts: Lifetime wages		Returns in lifetime earnings per child born to a targeted pregnant woman	
					in US $ millions	in International $ millions	in US $	in International $
Iron/Folic Acid Suppl.	90%	0.62 (-0.11, 1.40)	4.60 (-0.67, 13.32)	307 (-45, 890)	15.08 (-2.25, 45.01)	33.79 (-5.03, 100.88)	5.39 (-0.80, 16.09)	12.08 (-1.80, 36.07)
Calcium Suppl.	50%	1.37 (0.35, 2.70)	11.56 (0.43, 30.69)	772 (29, 2050)	37.40 (1.29, 104.41)	83.83 (2.88, 233.99)	24.07 (0.83, 67.19)	53.94 (1.85, 150.57)
	90%	2.47 (0.63, 4.86)	20.81 (0.77, 55.25)	1390 (51, 3690)	67.33 (2.31, 187.93)	150.89 (5.18, 421.19)	24.07 (0.83, 67.19)	53.94 (1.85, 150.57)
Multiple Micronutrient Suppl.	50%	0.71 (0.38, 1.10)	5.76 (1.22, 12.99)	384 (82, 868)	18.98 (3.98, 43.47)	42.55 (8.93, 97.43)	12.22 (2.56, 27.98)	27.38 (5.74, 62.70)
	90%	1.82 (0.92, 2.79)	14.34 (3.30, 30.94)	958 (221, 2066)	47.15 (10.61, 106.09)	105.66 (23.79, 237.77)	16.85 (3.79, 37.93)	37.77 (8.50, 85.00)
Balanced Protein Suppl.	50%	0.08 (-0.06, 0.22)	0.53 (-0.51, 2.03)	36 (-34, 136)	1.76 (-1.72, 6.88)	3.94 (-3.86, 15.42)	14.23 (-14.29, 47.07)	31.89 (-32.03, 105.48)
	90%	0.14 (-0.12, 0.40)	0.96 (-0.92, 3.66)	64 (-61, 244)	3.16 (-3.10, 12.38)	7.09 (-6.95, 27.76)	14.23 (-14.29, 47.07)	31.89 (-32.03, 105.48)

References for Data Inputs

[1] Blencowe H, Krasevec J, Onis M De, et al. Articles National , regional , and worldwide estimates of low birthweight in 2015 , with trends from 2000: a systematic analysis. Lancet Glob Heal. 2019;(18):1-12.
[2] Chawanpaiboon S, Vogel JP, Moller AB, et al. Global, regional, and national estimates of levels of preterm birth in 2014: a systematic review and modelling analysis. Lancet Glob Heal. 2019;7(1):e37-e46.
[3] United National Population Division World Population Prospects 2019.
[4] Fink G, Peet E, Danaei G, et al. Schooling and wage income losses due to early-childhood growth faltering in developing countries: National, regional, and global estimates. Am J Clin Nutr. 2016;104(1):104-112.
[5] Country specific annual wage data from World Indicators Database. Average yearly wage was estimated to be 2/3 of the gross domestic product in 2010 constant US dollars and 2011 International dollars, adjusted for purchasing power parity.
[6] NCD Risk Factor Collaboration. Trends in adult body-mass index in 200 countries from 1975 to 2014: a pooled analysis of 1698 population-based measurement studies with 19·2 million participants. Lancet. 2016;387(10026):1377-1396.
[7] Stevens GA, Finucane MM, De-Regil LM, et al. Global, regional, and national trends in haemoglobin concentration and prevalence of total and severe anaemia in children and pregnant and non-pregnant women for 1995-2011: A systematic analysis of population-representative data. Lancet Glob Heal. 2013;1(1):16-25.
[8] Coverage of iron-folic acid supplementation abstracted from the most recent Demographic Health Survey or imputed based on sub-regional average. Indicator used: % women in the past five years who took iron tablets or syrup for >90 days.

Zimbabwe

Region: Sub-Saharan Africa**; Sub-region:** Southern Sub-Saharan Africa

Low birthweight prevalence[1]: 12.6% (95% CI: 10.0, 16.5)

Preterm birth prevalence[2]: 12.0% (95% CI: 8.6, 16.7)

Number of births[3]: 2,211,000

Returns to education[4]: 16.1% (95% CI: 15.3, 16.8)

GDP per capita 2010 US$ (estimated annual wage)[5]: $1234 ($823/year)

GDP per capita 2011 International $ (estimated annual wage)[5]: $2509 ($1673/year)

Prevalence of low BMI[6]: 4.7% (95% CI: 2.2, 8.6)

Prevalence of anemia[7]: 34.4% (95% CI: 26.8, 44.0)

Baseline coverage of IFA[8]: 39.7%

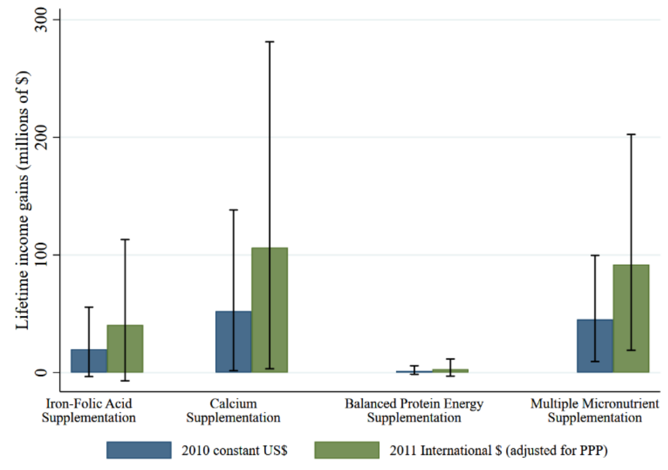

Figure: Benefits by birth cohort size for estimated gains in lifetime wages attributable to scaling up nutrition interventions to 90% target coverage

Table: Impact of maternal prenatal nutrition intervention on human capital and labour market outcomes, through improvements in low birthweight or preterm birth and schooling in Zimbabwe.

Intervention	Target Coverage (%)	Absolute reduction in birth outcome (%)	Benefits by cohorts: School years gained (in 1000 s)	No. of additional students completing secondary school	Benefits by cohorts: Lifetime wages		Returns in lifetime earnings per child born to a targeted pregnant woman	
					in US $ millions	in International $ millions	in US $	in International $
Iron/Folic Acid Suppl.	90%	1.06 (-0.19, 2.35)	5.65 (-0.93, 15.84)	627 (-103, 1758)	19.95 (-3.41, 55.65)	40.56 (-6.94, 113.14)	10.03 (-1.72, 27.97)	20.39 (-3.49, 56.86)
Calcium Suppl.	50%	1.41 (0.28, 2.52)	8.11 (0.25, 21.36)	901 (28, 2371)	29.04 (0.89, 76.84)	59.05 (1.81, 156.21)	26.27 (0.81, 69.50)	53.41 (1.64, 141.31)
	90%	2.55 (0.51, 4.53)	14.60 (0.46, 38.45)	1621 (51, 4267)	52.28 (1.61, 138.31)	106.28 (3.27, 281.18)	26.27 (0.81, 69.50)	53.41 (1.64, 141.31)
Multiple Micronutrient Suppl.	50%	0.90 (0.53, 1.33)	5.20 (1.13, 10.79)	577 (126, 1198)	18.36 (4.08, 38.18)	37.33 (8.30, 77.63)	16.61 (3.69, 34.54)	33.77 (7.51, 70.22)
	90%	2.31 (1.11, 3.63)	12.71 (2.63, 28.05)	1410 (292, 3114)	45.21 (9.34, 99.57)	91.92 (18.98, 202.44)	22.72 (4.69, 50.04)	46.20 (9.54, 101.73)
Balanced Protein Suppl.	50%	0.05 (-0.04, 0.14)	0.23 (-0.23, 0.90)	26 (-25, 100)	0.82 (-0.81, 3.17)	1.66 (-1.65, 6.44)	16.62 (-16.93, 55.70)	33.79 (-34.41, 113.24)
	90%	0.08 (-0.07, 0.25)	0.42 (-0.41, 1.62)	47 (-45, 180)	1.47 (-1.46, 5.70)	3.00 (-2.97, 11.60)	16.62 (-16.93, 55.70)	33.79 (-34.41, 113.24)

References for Data Inputs

[1] Blencowe H, Krasevec J, Onis M De, et al. Articles National , regional , and worldwide estimates of low birthweight in 2015 , with trends from 2000: a systematic analysis. Lancet Glob Heal. 2019;(18):1-12.
[2] Chawanpaiboon S, Vogel JP, Moller AB, et al. Global, regional, and national estimates of levels of preterm birth in 2014: a systematic review and modelling analysis. Lancet Glob Heal. 2019;7(1):e37-e46.
[3] United National Population Division World Population Prospects 2019.
[4] Fink G, Peet E, Danaei G, et al. Schooling and wage income losses due to early-childhood growth faltering in developing countries: National, regional, and global estimates. Am J Clin Nutr. 2016;104(1):104-112.
[5] Country specific annual wage data from World Indicators Database. Average yearly wage was estimated to be 2/3 of the gross domestic product in 2010 constant US dollars and 2011 International dollars, adjusted for purchasing power parity.
[6] NCD Risk Factor Collaboration. Trends in adult body-mass index in 200 countries from 1975 to 2014: a pooled analysis of 1698 population-based measurement studies with 19·2 million participants. Lancet. 2016;387(10026):1377-1396.
[7] Stevens GA, Finucane MM, De-Regil LM, et al. Global, regional, and national trends in haemoglobin concentration and prevalence of total and severe anaemia in children and pregnant and non-pregnant women for 1995-2011: A systematic analysis of population-representative data. Lancet Glob Heal. 2013;1(1):16-25.
[8] Coverage of iron-folic acid supplementation abstracted from the most recent Demographic Health Survey or imputed based on sub-regional average. Indicator used: % women in the past five years who took iron tablets or syrup for >90 days.
